# Synthetic Zwitterionic *Streptococcus pneumoniae* Type 1 Oligosaccharides Carrying Labile *O*‐Acetyl Esters

**DOI:** 10.1002/anie.202211940

**Published:** 2022-12-01

**Authors:** Zhen Wang, Ana Gimeno, Marta G. Lete, Herman S. Overkleeft, Gijsbert A. van der Marel, Fabrizio Chiodo, Jesús Jiménez‐Barbero, Jeroen D. C. Codée

**Affiliations:** ^1^ Leiden Institute of Chemistry Leiden University Einsteinweg 55 2333 CC Leiden (The Netherlands; ^2^ CIC bioGUNE Bizkaia Technology Park, Building 801A 48170 Derio Spain; ^3^ Institute of Biomolecular Chemistry National Research Council (CNR) Pozzuoli, Napoli Italy; ^4^ Amsterdam Infection and Immunity Institute Department of Molecular Cell Biology and Immunology Amsterdam UMC, Location VUmc 1007 MB Amsterdam (The Netherlands; ^5^ Ikerbasque Basque Foundation for Science Plaza Euskadi 5 48009 Bilbao, Bizkaia Spain; ^6^ Department of Organic Chemistry II Faculty of Science and Technology, EHU-UPV 48940 Leioa Spain; ^7^ Centro de Investigación Biomédica En Red de Enfermedades Respiratorias Madrid Spain

**Keywords:** Acyl Migration, Bacterial Oligosaccharides, Glycosylation, Helical Structure, Stereoselectivity

## Abstract

We herein report the first total synthesis of the *Streptococcus pneumoniae* serotype 1 (Sp1) oligosaccharide, a unique zwitterionic capsular polysaccharide carrying labile *O*‐acetyl esters. The target oligosaccharides, featuring rare α‐2,4‐diamino‐2,4,6‐trideoxy galactose (AAT) and α‐galacturonic acids, were assembled up to the 9‐mer level, in a highly stereoselective manner using trisaccharide building blocks. The lability of the *O*‐acetyl esters imposed a careful deprotection scheme to prevent migration and hydrolysis. The migration was investigated in detail at various pD values using NMR spectroscopy, to show that migration and hydrolysis of the C‐3‐*O*‐acetyl esters readily takes place under neutral conditions. Structural investigation showed the oligomers to adopt a right‐handed helical structure with the acetyl esters exposed on the periphery of the helix in close proximity of the neighboring AAT residues, thereby imposing conformational restrictions on the AATα1‐4GalA(3OAc) glycosidic linkages, supporting the helical shape of the polysaccharide, that has been proposed to be critical for its unique biological activity.

## Graphical abstract

The highly convergent and stereoselective total synthesis of zwitterionic *Streptococcus pneumoniae* serotype 1 (Sp1) oligosaccharides, carrying labile *O*‐acetyl esters, is described. Structural studies show the nonasaccharide to adopt a right‐handed helical structure in which the acetyl groups decorating the periphery stabilize the structure.

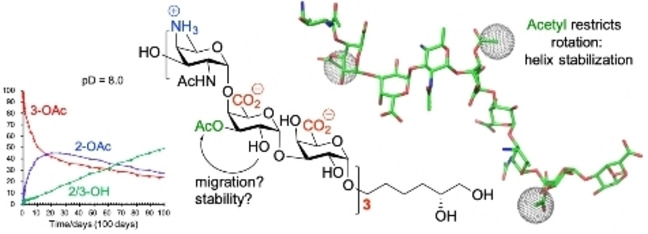

## Introduction

**Figure 1:**
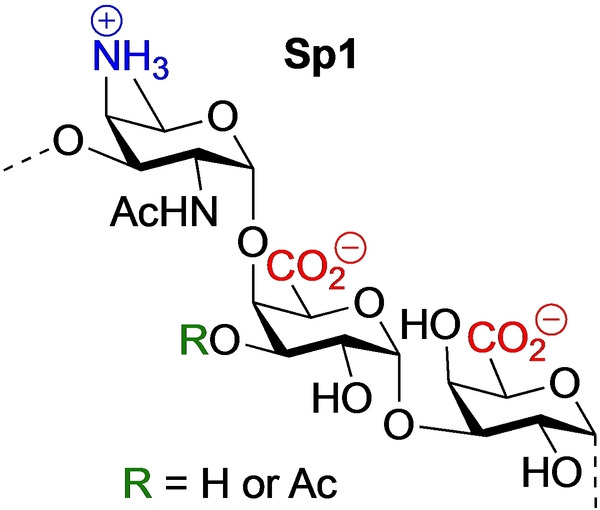
The structure of Sp1.

Bacterial cell‐surface carbohydrates play a significant role in binding events with components of the host immune system,[^1^, ^2^] and bacterial capsular polysaccharides (CPS) can effectively be used in the generation of carbohydrate‐based antibacterial vaccines. *Streptococcus pneumoniae* (or *pneumococcus*) is a dreaded alpha‐hemolytic Gram‐positive pathogen that can cause various types of potentially lethal infections, including pneumonia, septicemia, meningitis, leading to high morbidity and mortality rates worldwide.[^3^, ^4^, ^5^] Although antibiotic treatment for most pneumococcal infections is effective, it does induce the evolution of drug‐resistant pneumococcal bacteria. As early as 1946, the first pneumococcal polysaccharide vaccine was licensed^[6]^ and currently a 23‐valent CPS vaccine and 20‐valent glycoconjugate vaccine are available to offer protection against the most prevalent serotypes.^[7]^ However, even though these vaccines have been very successful, the immunological mechanism of the different serotypes is not well understood.[^8^, ^9^, ^10^, ^11^, ^12^] *S. pneumoniae* type 1 is a highly virulent strain, and it is characterized by a unique CPS, Sp1, that is built up from trisaccharide repeating units, that in turn are composed of the rare α‐2,4‐di‐amino‐2,4,6‐tri‐deoxygalactose (D‐AAT) and two α‐D‐galacturonic acid residues (Figure 1).[^13^, ^14^, ^15^] It has been reported that the polysaccharide can carry *O*‐acetyl groups at the C‐2 or C‐3 positions of the 4‐linked galacturonic acid residues and that approximately two third of the repeating units carry an acetyl ester.^[14]^ *O*‐acetylation is commonly encountered in bacterial polysaccharides, and the role of this modification can vary.[^15^, ^16^] It may be an important structural element for recognition by opsonic antibodies but can also shield immunogenic epitopes. It has been proposed that acetylation of Sp1 can impact the conformation and flexibility of the polysaccharide chains although previous models have fallen short in explaining the structural role of these labile functional groups.[^17^, ^18^, ^19^, ^20^, ^21^] The lability of the *O*‐acetyl groups complicates the isolation of well‐defined and pure CPS fragments for structural studies and the establishment of structure‐activity relationships at the molecular level. Migration of *O*‐acetyl groups has been observed between different hydroxyl groups within carbohydrate residues of polysaccharides but also between neighboring carbohydrate rings.[^22^, ^23^, ^24^] While biosynthesis enzymes can transfer acetyl esters in a highly regioselective manner, spontaneous migration events can lead to highly heterogeneous sequences.

In contrast to naturally sourced oligo/polysaccharides, synthetic oligosaccharides are well defined, uniform molecules, of a single length with predefined substitution patterns, making them expertly suited for structural studies and detailed interaction studies with binding partners.[^25^, ^26^, ^27^] Multiple synthetic efforts have been directed at the assembly of Sp1 oligosaccharides, but none have addressed the challenging incorporation of the labile *O*‐acetyl esters.[^28^, ^29^] The assembly of the Sp1 trisaccharide repeating unit has been reported by multiple groups[^30^, ^31^, ^32^] and Bundle and co‐workers were the first to report on the synthesis of Sp1 oligomers comprising two trisaccharide repeats.^[33]^ We have reported on the assembly of Sp1‐oligosaccharides, up to the dodecasaccharide level and we showed through molecular dynamics (MD) simulations and NMR spectroscopy studies that the oligomers adopt a right‐handed helical structure with the nonasaccharide completing a full turn.^[34]^ Kasper and co‐workers have linked the helical structure of zwitterionic polysaccharides to their unique capacity to bind to MHC‐II molecules, eliciting a T‐cell mediated immune response.^[35]^

The Sp1 polysaccharide represents major synthetic challenges, amongst which are the presence of the rare 2‐acetamido‐4‐amino‐2,4,6‐trideoxy‐D‐galactose (D‐AAT), the galacturonic acids, which are well known to be significantly less reactive in glycosylation reactions than their non‐oxidized galactose counterparts, and the *cis*‐glycosidic linkages that connect all monosaccharides. Although the presence of the *O*‐acetyl esters represents only a small structural modification, the incorporation of these labile esters presents an additional major hurdle, because of the migratory aptitude of these groups[^22^, ^23^, ^24^] and because their presence significantly limits protecting group manipulations as well as options for final deprotection chemistries.

We here describe the development of a synthetic route to generate acetylated Sp1 fragments ranging in length from a trisaccharide to a nonasaccharide, which have been synthesized in multi‐milligram amounts to allow for detailed structural studies. Molecular dynamics (MD) simulations and NMR spectroscopy were used to probe the 3‐dimensional structure of the Sp1 oligosaccharides. These revealed that the oligomers adopt a stable helical structure with the C‐3‐*O*‐acetyl groups being positioned on the outside of the helix and close enough to the neighboring AAT‐residues to provide an additional barrier for restricting the rotation of the AATα1‐4GalA(3OAc) glycosidic linkages. The stability of the *O*‐acetyl groups has been assessed by NMR spectroscopy at different pH values to show that they migrate readily at neutral and slightly basic pH, while being stable for prolonged period of time at slightly acidic pH (>1 year).

## Results and Discussion

**Scheme 1:**
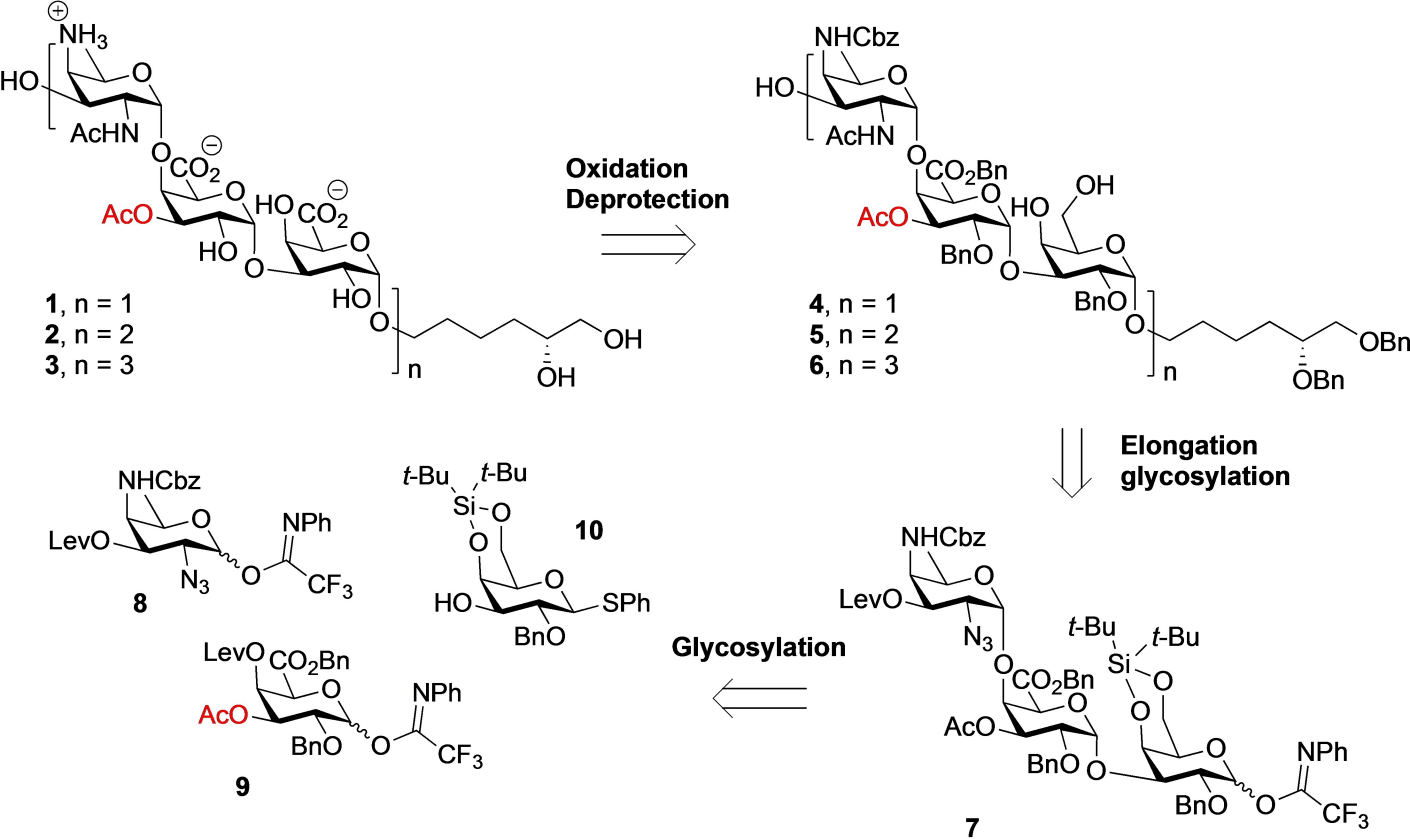
Retrosynthetic analysis for the assembly of target compounds **1**–**3**. Bn=benzyl, Cbz=carbobenzyloxy.

The acetylated Sp1‐fragments targeted in this study (trimer **1**, hexamer **2** and nonamer **3**) are shown in Scheme 1. We previously reported a route towards the de‐OAc‐Sp1 oligosaccharides, combining a pre‐glycosylation oxidation strategy with a post‐glycosylation oxidation approach, to minimize the difficult oxidation events required on large oligosaccharides while enabling a robust and highly stereoselective glycosylation protocol.^[34]^ A Birch‐type reduction was employed at the end of the synthesis to unmask all benzyl‐type protecting groups. Taking the acetyl groups in the target compounds **1**–**3** into consideration, this global deprotection scheme can obviously not be used and therefore a hydrogenation step will be required at the end of the synthesis. We decided to incorporate a vicinal diol terminated spacer to enable future conjugation chemistry after a selective oxidation by a Malaprade reaction.[^36^, ^37^] Building on our previously synthetic strategy, we set out to use trisaccharide **7** as the key building block (Scheme 1). This building block features a silylidene protected galactose donor moiety, that allows for the reliable, stereoselective formation of the required *cis*‐glycosidic linkages. To minimize oxidation events in the oligosaccharide stage the building block contains a galacturonic acid in the middle. The galacturonic acid also carries a C‐3‐*O*‐acetyl group that has to be maintained through the assembly. Trisaccharide **7** can be obtained from the monomeric building blocks **8**–**10**.

**Scheme 2:**
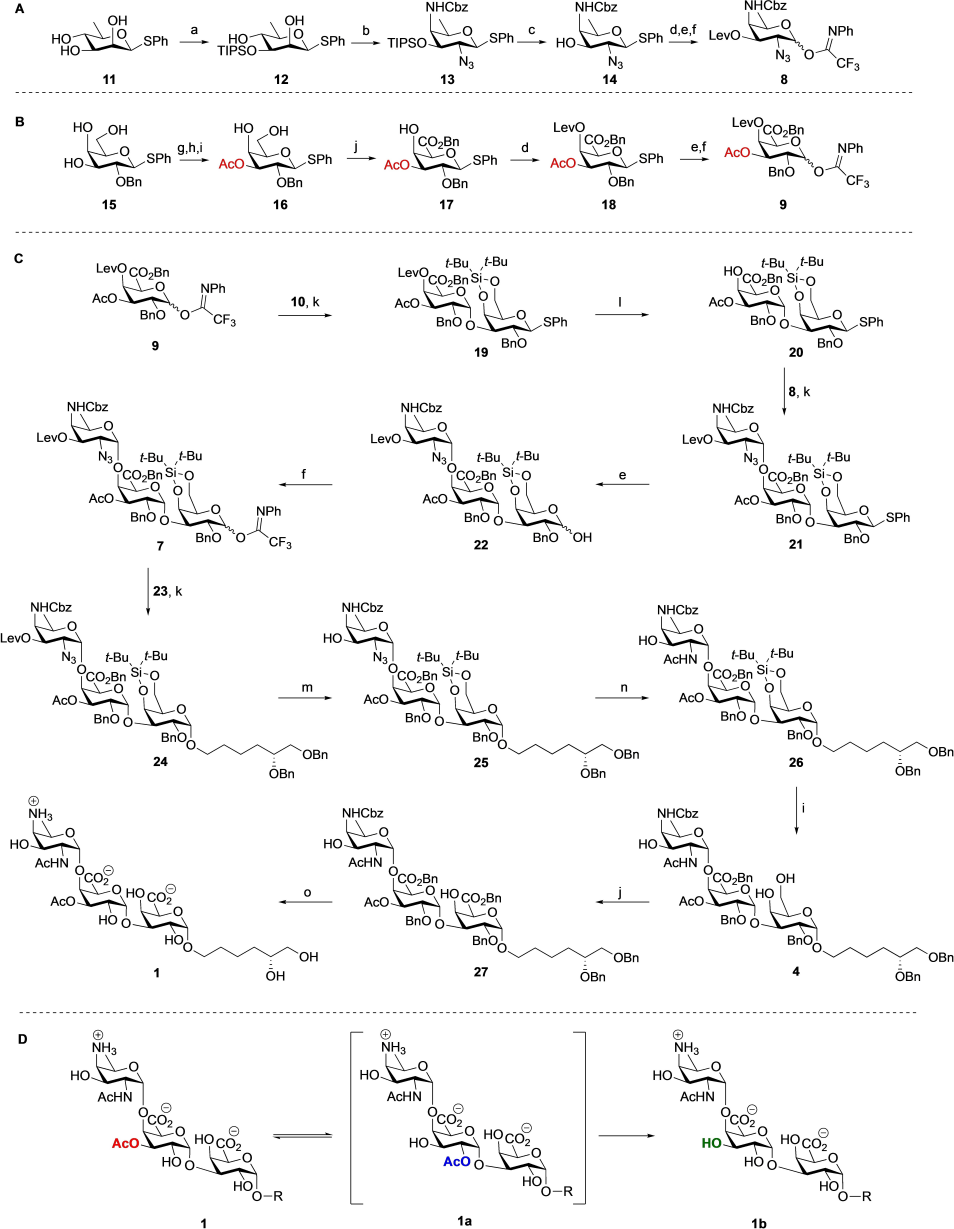
A) Synthesis of D‐AAT building block **8**. B) Synthesis of the central C‐3‐*O*‐acetyl galacturonic acid building block **9**. C) Assembly of trisaccharide **1**. D) Migration and hydrolysis of the C‐3′‐*O*‐acetyl ester. Reagents and conditions: a) TIPSCl, imidazole, DMF, 0 °C to rt, 88 %; b) i, Tf_2_O, Py, DMAP, DCM, −10 °C to 10 °C; ii, TBAN_3_, MeCN, −30 °C→−20 °C; iii, 7 N NH_3_ in MeOH; iv, CbzCl, Na_2_CO_3_, THF, H_2_O, 0 °C to rt; four steps 62 %; c) TBAF, AcOH, THF, quant.; d) LevOH, EDCI, DMAP, DCM, **14**, 91 %; **18**, 99 %; e) NIS, TFA, DCM, 0 °C, **8 b**, quant.; **9 a**, 96 %; **22**, 95 %; f) *N*‐phenyltrifluoroacetimidoyl chloride, Cs_2_CO_3_, acetone, **8**, 79 %; **9**, 91 %; **7**, 94 %; g) TBDPSCl, imidazole, DMF, 96 %; h) AcCl, Me_2_SnCl_2_, DIPEA, ThF; i) HF−Py, THF, Py, 0 °C, 85 % (two steps); j) i, TEMPO, BAIB, DCM, *t*‐BuOH, H_2_O; ii, BnBr, Cs_2_CO_3_, DMF, **17**, 77 %; **27**, 81 % (over two steps); k) TBSOTf, DCM, 5Å MS, 0 °C, **19**, 75 %; **21**, 82 % (α anomer, 68 %; β anomer, 14 %); **24**, 85 %; l) N_2_H_4_⋅AcOH, AcOH, THF, MeOH, 0 °C, 95 %; m) N_2_H_4_⋅H_2_O, pyridine, AcOH, 0 °C→RT, 95 %; n) i, PPh_3_, pyridine, H_2_O, THF, 70 °C, 7 h; ii, Ac_2_O, NaHCO_3_, THF, H_2_O, quantitative; o) Pd(OH)_2_/C, H_2_, *t*‐BuOH, H_2_O, 3 days, quantitative. BAIB=bis(acetoxy)iodobenzene, DCM=dichloromethane, DIPEA=*N*,*N*‐diisopropylethylamine, DMAP=4‐dimethylaminopyridine, DMF=*N*,*N*‐dimethylformamide, EDCI=1‐(3‐dimethylaminopropyl)‐3‐ethylcarbodiimide, NIS=*N*‐iodosuccinimide, Py=pyridine, TBAF=tetrabutylammonium fluoride, TBAN_3_=tetrabutylammonium azide, TBDPS=*tert*‐butyldiphenylsilyl, TBS=*tert*‐butyldimethylsilyl, TEMPO=2,2,6,6‐tetramethylpiperidin‐1‐oxyl, Tf=trifluoromethanesulfonyl, TFA=trifluoroacetic acid, TIPS=triisopropylsilyl.

We started our synthesis with the assembly of the required monomeric building blocks **8**–**10** as depicted in Scheme 2. The synthetic route to provide the rare 2‐acetamido‐4‐amino‐2,4,6‐trideoxy‐D‐galactose (D‐AAT) building block **8**,[^38^, ^39^] was optimized and started from 6‐deoxy mannose **11**[^34^, ^39^] (Scheme 2A). We installed a bulky TIPS group at the C‐3‐OH, after which the diol was triflated and the C‐2‐ and C‐4‐*O*‐triflates were subsequently substituted with an azide and ammonia respectively. After Cbz‐protection of the C‐4‐amine, AAT building block **13** was obtained in 62 % from diol **12**. For comparison, our old route using a C‐3‐*O*‐acetyl and potassium phthalimide instead of ammonia, provide the corresponding C‐3‐*O*‐acetyl AAT building block in only 21 % yield.^[34]^ Desilylation, levulinoylation and transformation of the thiophenol group into the corresponding *N*‐phenyl trifluoroacetimidate then provided building block **8**.

The required galacturonic acid synthon **9** was obtained from known C‐2‐*O*‐benzyl galactose **15** ^[34]^ by silylation of the primary alcohol, after which the C‐3‐OH was regioselectively acetylated by treating the diol with a catalytic amount of dimethyltin dichloride, di‐*iso*‐propyl ethylamine (DiPEA) and acetylchloride (Scheme 2B).[^40^, ^41^] After treatment of the resulting alcohol with HF‐pyridine, diol **16** was obtained that was regio‐ and chemoselectively oxidized using the TEMPO/BAIB combination. Benzylation of the so‐formed carboxylic acid delivered galacturonic acid **17** of which the C‐4‐OH was masked with a levulinoyl ester. Transformation of the thiophenol **18** into the *N*‐phenyl trifluoroacetimidate completed the synthesis of the central galacturonic acid building block **9**. The silylidene‐protected galactose building block **10** was assembled as previously described.^[34]^

With all building blocks in hand, the synthesis of the oligosaccharides commenced with the assembly of trisaccharide target **1** (Scheme 2C). First, the glycosylation between imidate galacturonic acid donor **9** and galactose acceptor **10** was performed in the presence of TBSOTf to provide disaccharide **19** in 75 % yield with excellent stereoselectivity. Subsequently, selective deprotection of the levulinoyl protecting group was effected by treatment of the disaccharide with hydrazine acetate under acidic conditions to prevent migration of the C‐3′‐acetate, to deliver the C4′‐OH disaccharide **20** in 95 % yield. Next, the glycosylation between acceptor **20** and AAT building block **8** was carried out under the promotion of TBSOTf to provide **21** in 82 % yield and a 5/1 α/β‐ratio. The anomers could be separated at this stage and the synthesis was continued with the hydrolysis of the thioglycoside moiety using NIS‐TFA,^[42]^ to provide the corresponding hemiacetal **22**, which was followed by installation of the imidate moiety delivering the pivotal trisaccharide imidate donor **7**. Next, the spacer, *R*‐5,6‐bis(benzyloxy)hexan‐1‐ol **23**, prepared according to literature procedures,[^43^, ^44^] was attached to the trisaccharide. Under control of the bulky silylidene, the glycosylation proceeded smoothly to furnish **24** in 85 % yield with complete stereoselectivity. Subsequently, the levulinoyl group was cleaved by using hydrazine monohydrate to provide the trisaccharide **25** in excellent yield. Reduction of the azide group using a Staudinger reaction, was followed by selective *N*‐acetylation to provide acetamide **26** in quantitative yield. Triol intermediate **4** could then be prepared in 95 % yield from **26** by treatment with hydrogen fluoride in pyridine. The regioselective oxidation to provide the carboxylic moiety proceeded uneventfully and was achieved using the TEMPO‐BAIB oxidation system in a *tert*‐butanol‐DCM‐water mixture at 4 °C, which was followed by benzylation using BnBr in DMF to provide trisaccharide **27** in 81 % yield. To complete the synthesis of the trisaccharide target **1**, all benzyl ethers, the benzyl esters and the benzyl carbamate were removed through a hydrogenation reaction using Pd(OH)_2_ as a catalyst. After purification by gel filtration column, however, the product proved to be impure and NMR analysis indicated the presence of side products, in which the acetyl group had migrated (**1 a**) or was hydrolyzed (**1 b**) (see Scheme 2D). The labile acetyl group in the trisaccharide apparently cannot withstand the slightly basic conditions used for the size exclusion chromatography for which an aqueous NH_4_OAc solution was used as eluent.[^22^, ^23^, ^24^] Therefore, we checked the integrity and purity of the trisaccharide immediately after the hydrogenation reaction. After filtration of the Pd‐catalyst and concentration, the trisaccharide proved to be pure and no sign of acetyl migration was detected by NMR spectroscopy.

**Scheme 3:**
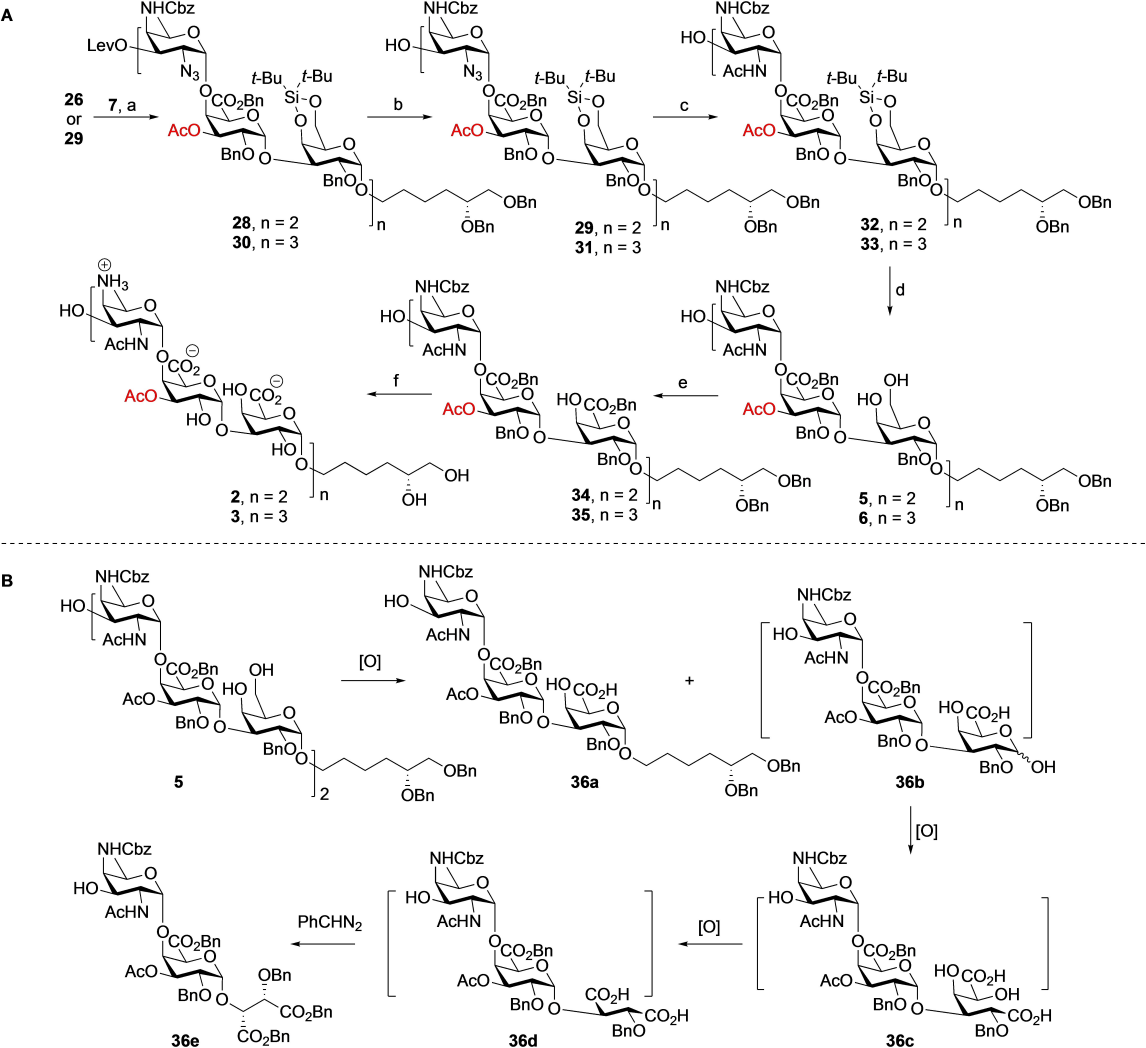
A) Assembly of targets **2** and **3**. B) Oxidative cleavage leading to fragmentation of the oligosaccharides. Reagents and conditions: a) TBSOTf, DCM, 5Å MS, 0 °C, **28**, 83 %; **30**, 85 %; b) N_2_H_4_⋅H_2_O, pyridine, AcOH, 0 °C→RT, **29**, 94 %; **31**, 89 %; c) i, PPh_3_, pyridine, H_2_O, THF, 70 °C, 7 h; ii, Ac_2_O, NaHCO_3_, THF, H_2_O, **32**, 88 %; **33**, 99 %; d) HF⋅Py, THF, pyridine, 0 °C, **5**, 92 %; **6**, 96 %; e) i, TEMPO, BAIB, *t*‐BuOH, H_2_O, NaHCO_3_, EtOAc or MeCN, 4 °C, 1 day; ii, PhCHN_2_, DCM, **34**, 58 %; **35**, 66 % (over two steps); f) Pd(OH)_2_/C, H_2_, *t*‐BuOH, 0.1 % AcOH in H_2_O, 3 days, **2**, 91 %; **3**, 90 %.

Having successfully assembled the trisaccharide, we moved to the preparation of longer oligomers (See Scheme 3A). Hexasaccharide **28** was synthesized using **26** and **7** in a TBSOTf mediated [3+3] glycosylation in 83 % yield as a single diastereoisomer. Removal of the levulinoyl group using hydrazine monohydrate, as described for the synthesis of **25**, furnished hexasaccharide **29**. Subsequently, a [3+6] glycosylation and delevulination cycle was carried out to uneventfully provide the nonasaccharide **31** in excellent yield. Following the synthetic approach for trisaccharide target **1**, a similar functionalization and deprotection sequence was performed with hexasaccharide **29** and nonasaccharide **31** to provide the partially protected hexasaccharide **5** and nonasaccharide **6**, respectively. Previously we noticed that the regioselective oxidation of multiple primary alcohols in the oligosaccharides became increasingly difficult with increasing substrate length. When the oxidation conditions, as previously optimized,^[34]^ were applied on hexasaccharide **5**, containing five free hydroxyls, the required product was formed in moderate yield and a major side product was isolated. The structure of this compound was elucidated using NMR and HRMS analysis and proved to be truncated disaccharide **36 e**. A possible mechanism for the formation of this trisaccharide is shown in Scheme 3B.^[45]^ Apparently, the oxidative conditions led to cleavage of the glycosidic bond at the junction of the trisaccharide repeating units. This would lead to two trisaccharide fragments **36 a**, and **36 b**. The hemiacetal **36 b** can undergo a further oxidation to the di‐acid **36 c**, the diol of which can be oxidatively cleaved to provide, after another oxidation, di‐acid **36 d**, which upon benzylation with phenyldiazomethane then provides **36 e**. The formation of this side product indicated that the long reaction time (3 days) and the large excess of oxidants used for the oxidation were too harsh for the substrate. Therefore, shorter reaction times were explored and the reaction was carefully monitored by thin‐layer chromatography from 10 h to 3 days. This indicated a reaction of 24 h to be optimal for the conversion of **5** into **36 a**. This way, hexasaccharide **34** was obtained in 58 % yield after a reaction with the TEMPO/BAIB reagent combination for 24 h at 4 °C in a *t*‐BuOH‐water‐EtOAc solvent system, followed by a benzylation using phenyldiazomethane. Similarly, the three primary alcohols of the nonasaccharide **6** were oxidized and benzylated to provide the corresponding nonamer **35** in 66 % yield over two steps. Cleavage of the nonasaccharide under the oxidative conditions could not be completely prevented as revealed by LC–MS analysis of the reaction mixture, which showed the formation of tri‐ and hexasaccharide fragments, including **27** and **34**. The formation of these side products confirms the regioselectivity of the cleavage reactions, taking place at the anomeric center of the galactose residues that have to be oxidized. The mechanism for the oxidative glycosidic bond cleavage remains to be established.

Unfortunately, the deprotection procedure used to prepare trisaccharide **1** from **27** was not suitable for the hexasaccharide, and acetyl migration and hydrolysis were detected by NMR and LC–MS analysis. After careful optimization of the solvent systems used and closely monitoring the pH of the reaction mixture, we managed to achieve the global deprotection by performing the hydrogenation under mild acidic conditions to provide the target hexasaccharide **2** in 91 % and nonasaccharide **3** in 90 % yield (Scheme 3A).

**Figure 2:**
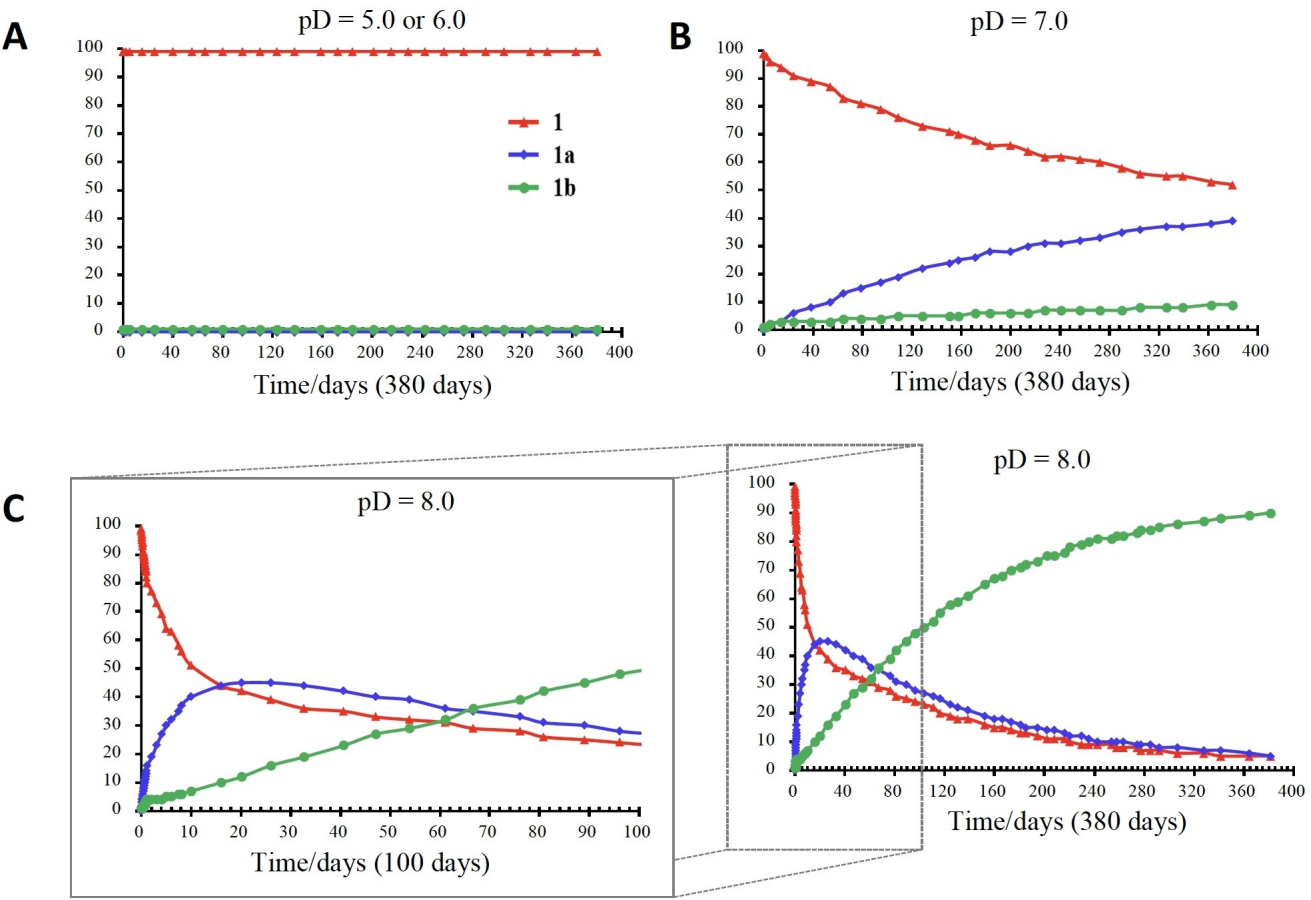
*O*‐Acetyl migration and hydrolysis in trisaccharide **1** at pD=5.0, 6.0 (A), 7.0 (B), or 8.0 (C).

The syntheses of the acetylated oligomers clearly indicated the C‐3‐*O*‐acetyl to be very labile. To evaluate the stability of the acetyl group more accurately, a set of NMR experiments was set up. NMR analyses were performed on samples of **1** in deuterated phosphate buffers at different pD values (pD=pH+0.4), ranging from pD 5.0 to pD 8.0 using the diagnostic peaks for H3 and H2 of the acetylated GalA residue (**1**: δ=5.33 ppm H3(OAc); **1 a**: δ=5.01 ppm H2(OAc); **1 b**: δ=4.10 H3(OH)). Trisaccharide **1 a** was formed as an intermediate during these experiments and was not isolated. The assignment of this structure was based on the characteristic chemical shifts of the mentioned protons and comparison to literature values^[14]^ (See Supporting Information for full details). As shown in Figure 2 and Scheme 2D, the acetyl group can migrate and hydrolyze from **1** to **1 a** and **1 b** under slightly basic conditions (pD=8.0), and migration was also possible, albeit very slowly, at pD 7.0, indicating the acetyl to be labile under basic, neutral and slightly acidic (pH 6.6) conditions. Of note, we did not observe any migration to the AAT nitrogen atom. At pD=8.0 the 3‐OAc↔2‐OAc migration was fast (Figure 2), with more than 50 % of the 3‐OAc migrating in 15 days.[^23^, ^24^] After 380 days the ratio of **1**, **1 a** and **1 b** was approximately 5 : 5 : 90. At lower pD (pD=5.0 and 6.0) no migration or hydrolysis was observed over a period as long as 380 days. These migration and cleavage studies underpin the requirement for slightly acidic conditions during the deprotection of the oligosaccharides. They also indicate that care should be taken when interaction studies are performed with these synthetic fragments or during isolation and manipulation of naturally sourced Sp1‐polysaccharides.

## Structural studies

**Figure 3:**
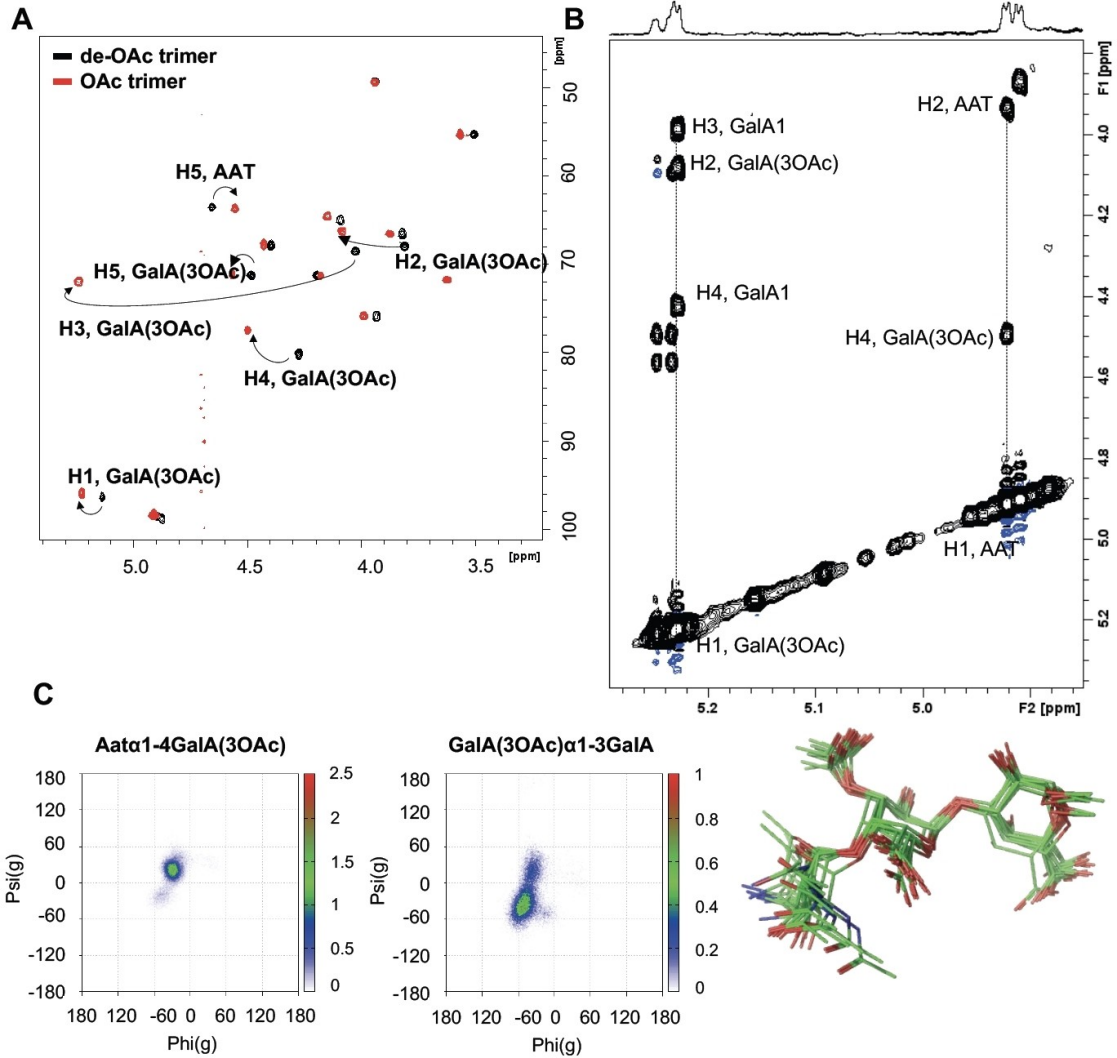
A) Superimposition of the HSQC spectra of the *O*‐acetylated Sp1 trimer (in black) and the de‐*O*‐acetylated homologue (in red). B) Expansion of the NOESY spectrum of trisaccharide **1**. Key cross‐peaks defining the conformation around the glycosidic linkages are indicated. C) 10 snapshot superimposition and Φ/Ψ maps of the conformations explored along the 500 ns MD simulation of trisaccharide **1**.

With the three target oligosaccharides in hand, we set out to probe their structure. All ^1^H NMR resonances of trimer **1** were assigned through standard TOCSY, NOESY and HSQC experiments. Notably, all ring protons of acetylated GalA residue showed downfield‐shifted resonances compared with the de‐*O*‐acetylated Sp1 oligomers we previously assembled. In addition, the NOESY spectrum of the *O*‐acetylated trimer showed key inter‐residue cross‐peaks which allowed us to unequivocally define the conformation of the trimer (Figure 3A). The NOEs for the H1_AAT_‐H4_GalA(3OAc),_ H1_GalA(3OAc)_‐H3_GalA_, and H1_GalA(3OAc)_‐H4_GalA_ proton pairs defined exo‐*syn*‐Φ/*syn*‐Ψ conformations around the glycosidic linkages. Fittingly, MD simulations predicted exo‐*syn*‐Φ/*syn*(+)‐Ψ and exo‐*syn*‐Φ/*syn*(−)‐Ψ conformations for the AAT‐(α1–4)‐GalA(3OAc) and GalA(3OAc)‐(α1–3)‐GalA glycosidic linkages, respectively, and they remained considerably stable along all the trajectory (Figure 3B and C). The proton signal of H5 of AAT shifted upfield due to the presence of the OAc group, while the AAT C6‐CH_3_ group appeared to be more deshielded appearing at a higher chemical shift. The MD simulation revealed that the AAT‐(α1‐4)‐GalA(3OAc) glycosidic linkage showed rather low flexibility while the GalA(3OAc)–(α1‐3)‐GalA linkage was more flexible, with continuous transitions between *syn*(−)‐Ψ and *syn*(+)‐Ψ conformers occurring along the entire simulation. The NOE‐estimated inter residual proton‐proton distances matched well with the average distances derived from the MD simulation.

**Figure 4:**
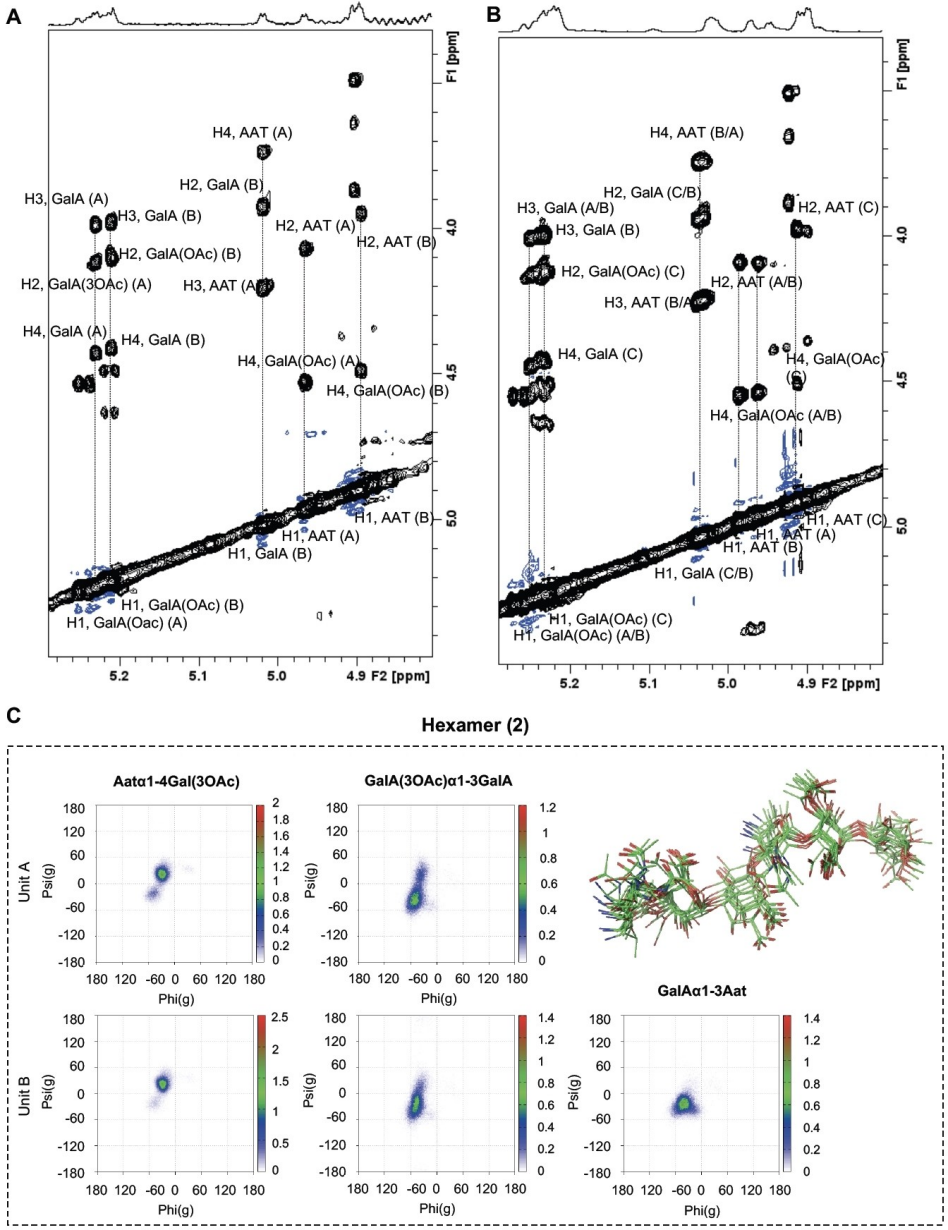
A) Expansion of NOESY spectrum of hexamer **2**. B) Expansion of the NOESY spectrum of nonamer **3**. Key cross‐peaks defining the conformation around the glycosidic linkages were indicated. C) Φ/Ψ maps and superimposition of representative structures explored along the 500 ns MD simulation of hexamer **2**.

**Figure 5:**
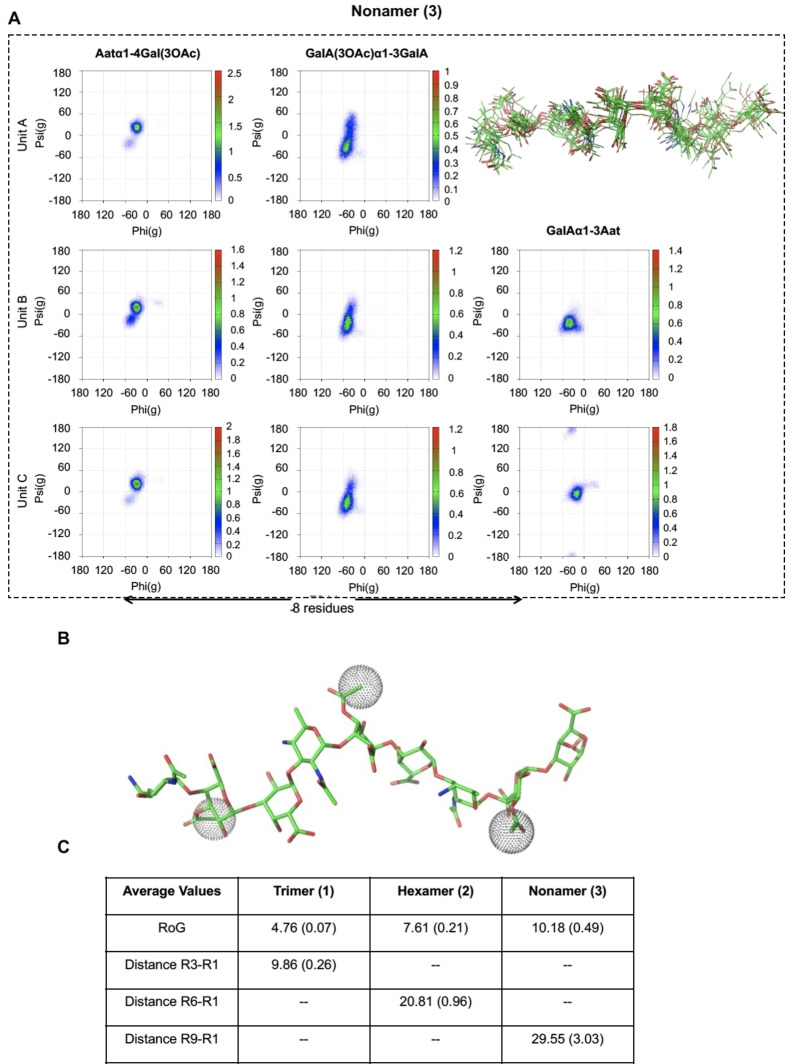
A) Φ/Ψ maps and superimposition of representative structures explored along the 500 ns MD simulation of and nonamer **3**. B) Molecular model of nonamer **3** where the acetyl groups were highlighted as balls. C) Average values of RoG and inter‐residue distances R_*n*_‐R_1_ for the three oligomers, measured from the center of mass of every residue. The standard deviation is indicated in parentheses. R_1_ is the residue at the reducing end.

Next, a similar analysis was performed for the hexasaccharide **2** and nonasaccharide **3** using NOESY experiments in combination with MD simulations to analyze their conformational features. Noteworthy, the H1‐H4 proton signals of all GalA(3OAc) residues were shifted downfield, highlighting the perturbation upon the introduction of the 3‐OAc groups. As observed for the trimer, the AAT H5 protons were shifted to higher field, while all AAT C6‐CH_3_ signals appeared at a higher chemical shift as compared to the non‐acetylated structures. NOESY experiments indicated that the exo‐syn‐Φ/syn‐Ψ conformations around AAT‐(α1‐4)‐Gal(3OAc), Gala(3OAc)‐(α1‐3)‐GalA, and GalA‐(α1‐3)‐AAT glycosidic linkages were the most populated (Figure 4A,B). 500 ns MD simulations supported these results and described rather similar conformational features for the longer oligosaccharides with exo‐syn‐Φ/syn(+)‐Ψ, exo‐syn‐Φ/syn(±)‐Ψ, and exo‐syn‐Φ/syn(−)‐Ψ conformations for the AAT‐(α1‐4)‐GalA(3OAc), Gala(3OAc)‐(α1‐3)‐GalA, and GalA‐(α1–3)‐AAT linkages, respectively (Figure 4C, 5A). Thus, as observed for the de‐*O*‐acetylated Sp1 fragments, the addition of repeating units does not alter the conformational flexibility around each glycosidic linkage. The MD simulations allowed us to analyze the global structure of the oligosaccharides revealing them to adopt an extended conformation, where on average the nonamer is three times as long as the trimer as defined by the inter‐residue distances (Figure 5C). Interestingly, nonamer **3** displayed a right‐handed helical structure, with eight residues completing a full turn (spanning ca. 25 Å, figure 5B). This structure is almost identical to the one described for its non‐acetylated counterpart and no major conformational changes could be observed. The GalA(3OAc) acetyl groups are solvent‐exposed and in close proximity to the C6‐CH_3_ groups of the neighboring AAT residues, explaining the deshielding effects observed by NMR. Indeed, the vicinity of the *O*‐acetyl groups to the AAT methyl group may provide an additional barrier for the free rotation around the AAT‐(α1‐4)‐Gal(3OAc) glycosidic linkage, then hampering the access to other (higher‐energy) conformations and thus stabilizing the helix. The close proximity of the methyl groups of the GalA(3OAc) acetyl and C6‐CH_3_ groups of the neighboring AAT residues creates hydrophobic patches on the periphery of the OAc‐Sp1 helix, which may play a role in the interaction with anti‐Sp1 antibodies.

## Conclusion

In conclusion, three *O*‐acetylated Sp1 fragments, a trisaccharide, hexasaccharide and nonasaccharide were successfully assembled, building on a synthesis approach, which strategically combined pre‐ and post‐glycosylation oxidation events. The regioselective oxidation of multiple primary alcohols in the complex oligosaccharide was accomplished using a modified TEMPO‐BAIB oxidation, which was devised because the original protocol led to oxidative cleavage of the oligosaccharide chain. The syntheses revealed the lability of the C‐3‐*O*‐acetyl esters and detailed stability studies indicated that migration and hydrolysis of this group can take place under neutral conditions. It is not unlikely that the mixed acetylation pattern observed in Sp1 polysaccharides, isolated from the bacteria, also originates from the migration of acetyl esters that are mounted on the polysaccharide in an initially regioselective manner by the bacterial *O*‐acetyl transferase. The synthetic fragments were used for structural studies, which revealed the oligomers to adopt a helical structure, with the ninemer completing a full turn. The helical structure of zwitterionic polysaccharides has been linked to the unique immunomodulatory properties of these polysaccharides, with the positively and negatively charged groups positioned properly in space to interact with the MHC‐II binding groove. With the set of well‐defined Sp1 oligomers now available, these interactions may now be evaluated in atomic detail. The assembled structures will also be used to probe their antigenicity and the installed spacer will allow for the attachment of the structures to carrier proteins to generate synthetic vaccines.

## Supporting Information

Detailed description of all experimental details and full characterization of all new compounds, including ^1^H and ^13^C spectra. Structural studies including NMR analyses and MD simulations.

## Conflict of interest

The authors declare no conflict of interest.

1

## Supporting information

As a service to our authors and readers, this journal provides supporting information supplied by the authors. Such materials are peer reviewed and may be re‐organized for online delivery, but are not copy‐edited or typeset. Technical support issues arising from supporting information (other than missing files) should be addressed to the authors.

Supporting Information

## Data Availability

The data that support the findings of this study are available in the Supporting Information of this article.
