# Supplementary material for: Synthetic Zwitterionic Streptococcus pneumoniae Type 1 Oligosaccharides Carrying Labile O‐Acetyl Esters
Source: Angew Chem Int Ed Engl. 2022 Dec 1;62(1):e202211940. doi: 10.1002/anie.202211940 (PMC10107948; doi:10.1002/anie.202211940)
Supplement: Supplementary file 1 — Supporting Information [file ANIE-62-0-s001.pdf]

Angewandte Chemie
Eine Zeitschrift der Gesellschaft Deutscher Chemiker

Supporting Information

Synthetic Zwitterionic *Streptococcus pneumoniae* Type 1 Oligosaccharides Carrying Labile *O*-Acetyl Esters

*Z. Wang, A. Gimeno, M. G. Lete, H. S. Overkleeft, G. A. van der Marel, F. Chiodo, J. Jiménez-Barbero, J. D. C. Codée**

Supporting Information

Table of Contents

Experimental Section

1. Oligosaccharide synthesis procedures

2. Structural studies

3. References

4. NMR spectra

Oligosaccharide synthesis procedures

General experimental procedures

All reagents were of commercial grade and used as received. All moisture sensitive reactions were performed under an argon or nitrogen atmosphere, at ambient temperature, unless stated otherwise. DCM used in the glycosylation reactions was dried with flamed 4Å or 5Å molecular sieves before being used. Reactions were monitored by TLC analysis with detection by UV (Merck, silica gel 60, F245) with detection by UV absorption (254 nm) and where applicable by spraying with 20% sulfuric acid in EtOH or with a solution of $(NH_4)_6Mo_7O_{24}\cdot 4H_2O$ (25 g/L) and $(NH_4)_4Ce(SO_4)_4\cdot 2H_2O$ (10 g/L) in 10% sulfuric acid (aq.) followed by charring at ~150 °C. Flash column chromatography was performed on silica gel (40-63µm). ^{1}H and ^{13}C spectra were recorded on a Bruker AV 400 or Bruker AV 500 or Bruker AV 600 and Bruker AV 850 in $CDCl_3$ or D_2O. Chemical shifts (δ) are given in ppm relative to tetramethylsilane as internal standard (^{1}H NMR in $CDCl_3$) or the residual signal of the deuterated solvent. Coupling constants (*J*) are given in Hz. All ^{13}C spectra are proton decoupled. NMR peak assignments were made using COSY and HSQC experiments, where applicable Clean TOCSY, HMBC and GATED experiments were used to further elucidate the structure. The anomeric product ratios were analyzed through integration of proton NMR signals. High-resolution mass (HRMS) was performed on a Thermo Finnigan LTQ Orbitrap mass spectrometer equipped with an electrospray ion source in positive ion mode (source voltage 3.5 kV, sheath gas flow 10, capillary temperature 275 °C) resolution R = 60.000 at m/z 400 (mass range of 150–4000) and dioctylphthalate(m/z = 391.28428) as lock mass, or on a Waters Spynat G2-Si(OTF) equipped with an electrospray ion source in positive mode (source voltage 3.5 kV) and LeuEnk (m/z = 556.2771). Optical rotation measurements ($[\alpha]_D^{20}$) were performed on an Anton Paar Modular Circular Polarimeter MCP 100/150 with a concentration of 10 mg/mL (c 1), unless stated otherwise.

Experimental procedures and characterization of products

Phenyl 6-deoxy-3-*O*-triisopropylsilyl-1-thio-β-D-mannopyranoside (12)

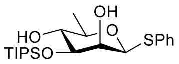

Phenyl 6-deoxy-1-thio-β-D-mannopyranoside **11**[1,2] (19.7 g, 76.9 mmol, 1.0 eq) was dissolved in DMF (154 mL) and cooled to 0 °C. Triisopropylsilyl chloride (TIPSCl) (33 mL, 153.8 mmol, 2.0 eq) and imidazole (31 g, 455 mmol, 6.0 eq) were added at 0 °C. The mixture

was stirred at RT for 24 hours and checked by TLC. After complete consumption of the starting material, the mixture was diluted with EtOAc, and washed with water and brine. The organic layer was dried with anhydrous $MgSO_4$, filtered, and concentrated *in vacuo*. The compound was purified by flash chromatography (PE/EA 20:1 - 8:1) to yield compound **12** (28 g, 67.9 mmol, 88%). ^{1}H NMR (500 MHz, Chloroform-*d*) δ 7.55 – 7.49 (m, 2H), 7.34 – 7.23 (m, 3H), 4.89 – 4.81 (m, 1H, H-1), 4.16 – 4.08 (m, 1H, H-2), 3.74 (dd, *J* = 8.8, 3.5 Hz, 1H, H-3), 3.63 – 3.54 (m, 1H, H-4), 3.41 – 3.29 (m, 1H, H-5), 2.66 (t, *J* = 1.8 Hz, 1H, 2-OH), 2.08 (d, *J* = 3.4 Hz, 1H, 4-OH), 1.41 (d, *J* = 6.1 Hz, 3H, H-6), 1.17 – 1.07 (m, 21H, TIPS). ^{13}C NMR (126 MHz, $CDCl_3$) δ 130.9, 129.0, 127.3, 86.4 (C-1), 76.6 (C-3), 75.9 (C-5), 73.3, 73.3 (C-2, C-4), 18.2, 18.1, 18.1 (C-6), 12.6. HR-MS: Calculated for $C_{21}H_{36}O_4SSi$ $[M+Na]^+$: 435.19958, found: 435.19957. $[\alpha]^{20}_D$ = - 40.0º (c = 1, $CHCl_3$). TLC: Rf = 0.5 (PE/EA = 4/1, v/v).

Phenyl 2-azido-4-*N*-benzyloxycarbonyl-6-deoxy-3-*O*-triisopropylsilyl-1-thio-β-D-galactopyranoside (13)

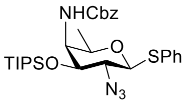

Compound **12** (1.57 g, 3.81 mmol, 1.0 eq) was dissolved in DCM (54 ml) with pyridine (4 mL, 50 mmol, 13.0 eq) and DMAP (466 mg, 3.81 mmol, 1.0 eq), then Tf_2O (3.8 mL, 22.9 mmol, 6.0 eq) was added to the reaction mixture at -10 °C, and the mixture was slowly warmed up to 10 °C in 2 h. After TLC showed complete consumption of the starting material, the reaction mixture was diluted with DCM and washed with 1M HCl solution and saturated aqueous sodium bicarbonate. The organic layer was dried over Na_2SO_4 and concentrated *in vacuo*. The residue was dissolved in dry CH_3CN (50 mL), $TBAN_3$ (1.11 g, 3.90 mmol, 1.02 eq) solution in CH_3CN (5 mL) was slowly added to the reaction mixture at -30 °C and the reaction was stirred for one day. The reaction was warmed slowly to -20°C and stirred for additional 2 days. After TLC showed complete consumption of the starting material, 7N NH_3 in methanol (10 mL) was added at -20°C. The reaction was slowly warmed to 5 °C and stirred for 3 days. After TLC showed complete consumption of the starting material, the mixture was concentrated *in vacuo*. The residue was dissolved in THF (28 mL) and water (19 mL), and sodium bicarbonate (1.28 g, 15.2 mmol, 4.0 eq) were added and the mixture was cooled to 0 °C. After addition of benzyl chloroformate (CbzCl) (1.1 mL, 7.6 mmol, 2.0 eq) the mixture was stirred overnight at room temperature. After TLC showed complete consumption of the starting material, the reaction was quenched with saturated aqueous

sodium bicarbonate and diluted with EtOAc. The mixture was washed with water (2x) and brine. The aqueous layer was extracted with EA (3x), dried with $MgSO_4$, filtered, and concentrated *in vacuo*. The compound was purified by flash chromatography (PE/EA 5:1 - 3:1) to yield compound **13** (1.36 g, 2.38 mmol, 62%). ^{1}H NMR (400 MHz, Chloroform-*d*) δ 7.59 – 7.53 (m, 2H), 7.40 – 7.26 (m, 8H), 5.08 (q, *J* = 12.2 Hz, 2H, Cbz), 4.79 (d, *J* = 10.0 Hz, 1H, N*H*Cbz), 4.42 (d, *J* = 10.2 Hz, 1H, H-1), 4.01 – 3.93 (m, 1H, H-4), 3.77 (dd, *J* = 9.5, 4.4 Hz, 1H, H-3), 3.63 – 3.53 (m, 1H, H-5), 3.09 (t, *J* = 9.8 Hz, 1H, H-2), 1.24 (d, *J* = 6.3 Hz, 3H, H-6), 1.15 – 1.00 (m, 21H, TIPS). ^{13}C NMR (101 MHz, $CDCl_3$) δ 156.8 (Cbz), 133.1, 129.2, 128.6, 128.5, 128.2, 128.2, 87.4 (C-1), 74.6 (C-5), 73.9 (C-3), 67.0 (Cbz), 64.7 (C-2), 55.6 (C-4), 18.1, 18.0, 17.2 (C-6), 12.8. HR-MS: Calculated for $C_{29}H_{42}N_4O_4SSi$ [M+H$^+$]: 571.27688, found: 571.27703. $[\alpha]^{20}_D$ = + 0.6° (c = 1, $CHCl_3$). TLC: Rf = 0.3 (PE/EA = 20/1, v/v).

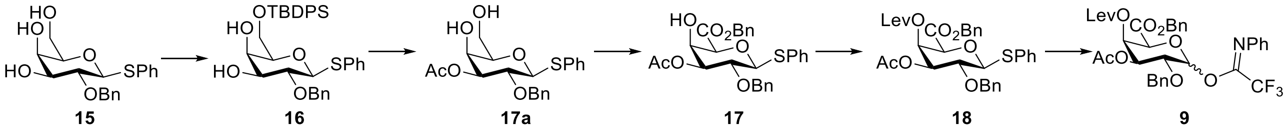

Scheme 1. Synthesis of the galacturonic acid building block.

Phenyl 2-*O*-benzyl-6-*O*-*tert*-butyldiphenylsilyl-1-thio-β-D-galactopyranoside (16)

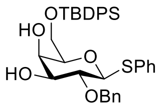

Phenyl 2-*O*-benzyl-1-thio-β-D-galactopyranoside **15**[3] (16.4 g, 45.3 mmol, 1.0 eq) was dissolved in DMF (91 mL) and cooled to 0 °C. *tert*-Butyl(chloro)diphenylsilane (TBDPSCl) (14.2 mL, 54.5 mmol, 1.2 eq) and imidazole (4.7 g, 69.0 mmol, 1.5 eq) were added at 0 °C. The mixture was stirred at RT for 4 hours and checked by TLC. After complete consumption of the starting material, the mixture was diluted with EtOAc, and washed with water and brine. The organic layer was dried with anhydrous $MgSO_4$, filtered, and concentrated *in vacuo*. The compound was purified by flash chromatography (PE/EA 6:1 - 3:1) to yield compound **16** (26 g, 43.3 mmol, 96%). ^{1}H NMR (400 MHz, Chloroform-*d*) δ 7.76 – 7.64 (m, 4H), 7.59 – 7.51 (m, 2H), 7.46 – 7.16 (m, 14H), 4.89 (d, *J* = 10.9 Hz, 1H, C*H*$_2$), 4.69 (d, *J* = 10.9 Hz, 1H, C*H*$_2$), 4.64 – 4.55 (m, 1H, H-1), 4.06 – 4.01 (m, 1H, H-4), 3.98 – 3.87 (m, 2H, H-6), 3.66 – 3.57 (m, 2H, H-3, H-2), 3.49 – 3.42 (m, 1H, H-5), 2.74 (s, 1H), 1.06 (s, 9H, TBDPS). ^{13}C NMR (101 MHz, $CDCl_3$) δ 138.2, 135.7, 135.6, 134.2, 132.8, 132.7, 131.3, 129.9, 128.9, 128.5, 128.3, 128.0, 127.8, 127.8, 127.2, 87.6 (C-1), 78.1 (C-3), 77.8 (C-5), 75.4, 75.2 (C-2), 69.6 (C-4), 63.9 (C-6), 26.8, 19.1. HR-MS: Calculated for

$C_{35}H_{40}O_5SSi$ [M+Na$^+$]: 623.2258, found: 623.2256. $[\alpha]^{20}_D$ = + 6.8° (c = 1, $CHCl_3$). TLC: Rf = 0.4 (PE/EA = 3/1, v/v).

Phenyl 2-*O*-benzyl-3-*O*-acetyl-1-thio-β-D-galactopyranoside (17a)

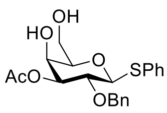

Compound **16** (2.5 g, 4.24 mmol, 1.0 eq) was dissolved in dry THF (22 mL). DIPEA (1.5 mL, 8.5 mmol, 2.0 eq), and Me_2SnCl_2 (50 mg, 0.23 mmol, 0.05 eq) were added and stirred for 15 min. Then acetyl chloride (362 μL, 5.07 mmol, 1.2 eq) was added and the reaction was stirred at RT overnight. After TLC showed complete consumption of the starting material, the reaction was quenched with 3% HCl solution and washed with H_2O (2x), brine. The organic phase was dried with $MgSO_4$, filtered, and concentrated *in vacuo*. The residue was dissolved in THF (30 mL) and pyridine (30 mL), then cooled to 0 °C and hydrogen fluoride (HF)/pyridine (70%) (3 mL) was added dropwise. The solution was stirred overnight. After TLC showed complete consumption of the starting material, the reaction was quenched with saturated aqueous sodium bicarbonate slowly and diluted with EtOAc. The solution was washed with water (2x) and brine. The aqueous layer was extracted with EtOAc (3x), dried with $MgSO_4$, filtered, and concentrated *in vacuo*. The compound was purified by flash chromatography (DCM/Acetone 20:1 - 5:1) to yield compound **17a** (1.5 g, 3.6 mmol, 85%). ^{1}H NMR (400 MHz, Chloroform-*d*) δ 7.58 – 7.52 (m, 2H), 7.37 – 7.23 (m, 8H), 4.92 (dd, J = 9.5, 3.1 Hz, 1H, H-3), 4.86 (d, J = 10.9 Hz, 1H, CH_2), 4.73 (d, J = 9.7 Hz, 1H, H-1), 4.58 (d, J = 11.0 Hz, 1H, CH_2), 4.19 (dd, J = 3.1, 1.0 Hz, 1H, H-4), 3.92 – 3.80 (m, 3H, H-2, H-6), 3.59 – 3.52 (m, 1H, H-5), 2.85 (s, 2H), 2.02 (s, 3H, OAc). ^{13}C NMR (101 MHz, $CDCl_3$) δ 170.4 (OAc), 138.0, 133.5, 131.8, 129.2, 128.5, 128.0, 128.0, 127.8, 88.0 (C-1), 77.4 (C-5), 76.8 (C-3), 75.6 (CH_2), 75.4 (C-2), 68.8 (C-4), 63.0 (C-6), 21.1 (OAc). HR-MS: Calculated for $C_{21}H_{24}O_6S$ [M+Na$^+$]: 427.1186, found: 427.1185. $[\alpha]^{20}_D$ = + 21.1° (c = 1, $CHCl_3$). TLC: Rf = 0.5 (DCM/Acetone = 4/1, v/v).

Benzyl phenyl 3-*O*-acetyl-2-*O*-benzyl-1-thio-β-D-galactopyranosyl uronate (17)

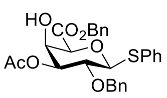

Compound **17a** (7.8 g, 19.3 mmol, 1.0 eq) was dissolved in DCM/*tert*-BuOH/H_2O (146 mL, 4/4/1, v/v/v). The mixture was cooled to 0 °C and treated with TEMPO (608 mg, 3.9 mmol, 0.2 eq) and BAIB (16 g, 48.2 mmol, 2.5 eq). After stirring overnight at 4 °C TLC showed complete consumption of the starting material and saturated aqueous sodium thiosulphate was added and the mixture was diluted with EtOAc and washed with brine. The organic phase was dried over Na_2SO_4 and concentrated *in vacuo*. The crude residue was dissolved in DMF (77 mL),

followed by addition of Cs_2CO_3 (6.4 g, 19.6 mmol, 1.0 eq) and BnBr (4.6 mL, 38.5 mmol, 2.0 eq) at 0 °C. The mixture was allowed to stir overnight at RT, and then diluted with EtOAc and washed with brine. The organic phase was dried over Na_2SO_4 and concentrated *in vacuo*. Purification by column chromatography (silica gel, pentane/DCM/EA, 7/2/1) yielded **17** (7.6 g, 14.9 mmol, 77%). ^{1}H NMR (400 MHz, Chloroform-*d*) δ 7.68 – 7.60 (m, 2H), 7.41 – 7.21 (m, 13H), 5.28 – 5.19 (m, 2H, C*H*2), 4.97 (dd, *J* = 9.6, 3.1 Hz, 1H, H-3), 4.88 (d, *J* = 11.0 Hz, 1H, C*H*2), 4.67 (d, *J* = 9.7 Hz, 1H, H-1), 4.59 (d, *J* = 11.0 Hz, 1H, C*H*2), 4.44 (dd, *J* = 3.1, 1.2 Hz, 1H, H-4), 4.19 (d, *J* = 1.2 Hz, 1H, H-5), 3.81 (t, *J* = 9.6 Hz, 1H, H-2), 2.23 (s, 1H), 2.01 (s, 3H, OAc). ^{13}C NMR (101 MHz, $CDCl_3$) δ 170.1 (OAc), 167.2, 137.9, 135.1, 133.2, 132.6, 129.1, 128.7, 128.7, 128.6, 128.5, 128.4, 128.2, 128.0, 128.0, 88.0 (C-1), 76.8 (C-5), 75.8 (C-3), 75.7 (*C*H2), 75.0 (C-2), 68.5 (C-4), 67.5 (*C*H2), 21.0 (OAc). HR-MS: Calculated for $C_{28}H_{28}O_7S$ [M+Na$^+$]: 531.1448, found: 531.1448. $[\alpha]^{20}_D$ = + 4.5° (c = 1, $CHCl_3$). TLC: Rf = 0.1 (PE/DCM/EA = 7/2/1, v/v/v).

Benzyl phenyl 3-*O*-acetyl-2-*O*-benzyl-4-*O*-levulinoyl-1-thio-β-D-galactopyranosyl uronate (18)

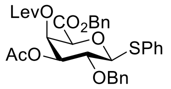

Compound **17** (1.05 g, 2.1 mmol, 1.0 eq) was co-evaporated with anhydrous toluene three times and dissolved in DCM (20 mL). At 0 °C, levulinic acid (668 mg, 5.8 mmol, 2.8 eq), 1-ethyl-3-(3-dimethylaminopropyl) carbodiimide (EDCI) (640 mg, 4.1 mmol, 2.0 eq) and 4-dimethylaminopyridine (DMAP) (50 mg, 0.41 mmol, 0.2 eq) were added. The reaction was stirred for 2 days at RT. The reaction was diluted with DCM and washed with saturated aqueous sodium bicarbonate and brine. The organic phase was dried with $MgSO_4$, filtered and concentrated *in vacuo*. The compound was purified by flash chromatography (Tol/EA 20:1 - 10:1) to yield compound **18** (1.21 g, 2.0 mmol, 99%). ^{1}H NMR (400 MHz, Chloroform-*d*) δ 7.72 – 7.62 (m, 2H), 7.41 – 7.23 (m, 13H), 5.76 – 5.71 (m, 1H, H-4), 5.23 (d, *J* = 11.9 Hz, 1H, C*H*2), 5.12 (d, *J* = 11.9 Hz, 1H, C*H*2), 5.02 (dd, *J* = 9.6, 3.4 Hz, 1H, H-3), 4.85 (d, *J* = 10.9 Hz, 1H, C*H*2), 4.69 (d, *J* = 9.7 Hz, 1H, C*H*2), 4.59 (d, *J* = 10.9 Hz, 1H, C*H*2), 4.28 (d, *J* = 1.4 Hz, 1H, H-5), 3.70 (t, *J* = 9.7 Hz, 1H, H-2), 2.64 – 2.44 (m, 3H, Lev), 2.34 – 2.23 (m, 1H, Lev), 2.16 (s, 3H, Lev), 1.92 (s, 3H, OAc). ^{13}C NMR (101 MHz, $CDCl_3$) δ 206.1 (Lev), 171.5 (Lev), 170.2 (OAc), 165.9 (CO_2Bn), 137.9, 135.1, 133.3, 132.8, 129.1, 129.0, 128.8, 128.7, 128.7, 128.5, 128.3, 128.2, 128.0, 128.0, 87.9 (C-1), 75.7, 75.4 (C-5), 74.8 (C-2), 73.9 (C-3), 69.0 (C-4), 67.6 (CO_2Bn), 37.7, 30.0 (Lev), 27.7, 20.7 (OAc). HR-MS: Calculated for $C_{33}H_{34}O_9S$ [M+Na$^+$]: 629.18157, found: 629.18103. $[\alpha]^{20}_D$ = + 4.6° (c = 1, $CHCl_3$). TLC: Rf = 0.2

(Tol/EA = 9/1, v/v).

Benzyl 3-*O*-acetyl-2-*O*-benzyl-4-*O*-levulinoyl-α/β-D-galactopyranosyl uronate (9a)

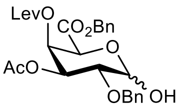

Compound **18** (490 mg, 0.79 mmol, 1.0 eq) was dissolved in DCM (8 mL) and cooled to 0 °C. NIS (195 mg, 0.87 mmol, 1.1 eq) and TFA (67 µL, 0.87 mmol, 1.1 eq) were added and the solution stirred for 2 hours. After analysis by TLC showed complete consumption of the starting material, the reaction was quenched with triethyl amine and saturated aqueous sodium thiosulphate. The solution was diluted with DCM and washed with brine (3x). The organic phase was dried with $MgSO_4$, filtered, and concentrated *in vacuo*. The compound was purified by flash chromatography (PE/EA 2:1 - 1:1) to yield the titled compound (390 mg, 0.76 mmol, 96%). ^{1}H NMR (400 MHz, Chloroform-*d*) δ 7.43 – 7.16 (m, 10H), 5.82 – 5.63 (m, 1H, H-4), 5.45 – 5.32 (m, 2H, H-1, H-3), 5.29 – 5.19 (m, 1H, C*H*2), 5.13 – 5.00 (m, 1H, C*H*2), 4.88 – 4.80 (m, 1H, H-5), 4.74 – 4.56 (m, 2H, C*H*2), 3.93 – 3.65 (m, 2H, H-2), 2.66 – 2.35 (m, 3H, Lev), 2.31 – 2.09 (m, 4H, Lev), 2.01 – 1.89 (m, 3H, OAc). ^{13}C NMR (101 MHz, $CDCl_3$) δ 206.3, 206.2 (Lev), 171.5 (Lev), 170.4, 170.3 (OAc), 167.4, 166.6 (CO_2Bn), 138.3, 137.7, 135.0, 134.9, 129.2, 128.7, 128.7, 128.7, 128.6, 128.4, 128.2, 128.0, 127.9, 127.8, 97.5, 92.0 (C-1), 77.0, 74.9, 73.5, 73.2 (C-2), 72.1, 71.9, 69.6 (C-4), 69.2 (C-3), 68.7, 68.5 (C-5), 67.8, 67.6 (C-6), 37.7, 37.7, 29.9, 27.6, 20.8 (OAc). HR-MS: Calculated for $C_{27}H_{30}O_{10}$ $[M+NH_4]^+$: 537.17312, found: 537.17302. TLC: Rf = 0.3 (PE/EA = 10/1, v/v).

Benzyl *N*-phenyl-trifluoroacetimidate 3-*O*-acetyl-2-*O*-benzyl-4-*O*-levulinoyl-α/β-D-galactopyranosyl uronate (9)

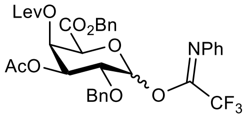

The hemiacetal **9a** (5.5 g, 10.7 mmol, 1.0 eq) was dissolved in acetone (110 mL) and cooled to 0 °C. Cesium carbonate (3.3 g, 12.9 mmol, 1.2 eq) was added. After 15 min, *N*-phenyl trifluoroacetimidoyl chloride (3.3 g, 15.9 mmol, 1.5 eq) was added, and then the reaction was allowed to stir overnight at RT. After analysis by TLC showed complete consumption of the starting material, the reaction was quenched by triethyl amine, the mixture filtered and concentrated *in vacuo*, and the product purified by column chromatography (PE/EA 5:1 – 2/1) to yield compound **9** (6.62 g, 9.66 mmol, 91%). ^{1}H NMR (400 MHz, Chloroform-*d*) δ 7.41 – 7.01 (m, 13H), 6.85 – 6.68 (m, 2H), 5.95 – 5.30 (m, 2H, H-4, H-3), 5.29 – 5.19 (m, 1H, C*H*2), 5.15 – 4.88 (m, 2H, H-5, C*H*2), 4.86 – 4.76 (m, 1H, C*H*2), 4.73 – 4.64 (m, 1H, C*H*2), 3.96 –

3.73 (m, 1H, H-2), 2.68 – 2.43 (m, 3H, Lev), 2.33 – 2.24 (m, 1H, Lev), 2.20 – 2.13 (m, 3H, Lev), 2.01 – 1.82 (m, 3H, OAc). ^{13}C NMR (101 MHz, $CDCl_3$) δ 206.1 (Lev), 171.4 (Lev), 170.1 (OAc), 165.3 (CO_2Bn), 137.4, 134.9, 129.3, 129.3, 128.9, 128.8, 128.7, 128.7, 128.7, 128.6, 128.6, 128.2, 128.1, 124.5, 119.4, 96.6 (C-1), 75.4, 74.9, 72.8, 71.9, 68.4, 67.8, 37.6, 29.9, 27.6, 20.6. HR-MS: Calculated for $C_{35}H_{34}F_3NO_{10}$ [M+Na$^+$]: 708.20270, found: 708.20297. TLC: Rf = 0.2 (PE/EA = 7/3, v/v).

(*R*)-5,6-bis(benzyloxy)hexan-1-ol (23)

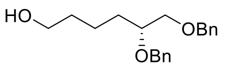

AD-mix-β (28.5 g) was dissolved in *tert*-BuOH/H_2O (192 mL, 1/1, v/v). The mixture was cooled to 0 °C and 2-((hex-5-en-1-yloxy)methyl)naphthalene[4] (4.88 g, 20.3 mmol, 1.0 eq) was added and stirred for overnight. After TLC showed complete consumption of the starting material, solid sodium sulfite (25 g) was added slowly at 0 °C. The resulting suspension was allowed to warm to room temperature, stirred for an additional 1 h, and diluted with DCM. diluted with EtOAc, washed with brine. The organic phase was dried over Na_2SO_4 and concentrated *in vacuo*. The aqueous phase was extracted with DCM, and the combined organic extracts were dried over MgSO4, filtered, and concentrated.[5] The residue was dissolved in DMF (32 mL), and sodium hydride (3.3 g, 81.2 mmol, 4.0 eq) was added and stirred for 15 min. Benzyl bromide (7.3 mL, 60.9 mmol, 3.0 eq) was added at 0 °C. The mixture was slowly warmed to RT and stirred overnight. After analysis by TLC showed complete consumption of the starting material, the reaction was quenched with MeOH and water. The mixture was diluted with EtOAc and washed with water and brine. The organic layer was dried with anhydrous $MgSO_4$, filtered, and concentrated *in vacuo*. The crude was dissolved in DCM (400 mL) and water (40 mL). After cooling to 0 °C, 2,3-dichloro-5,6-dicyano-*p*-benzoquinone (DDQ) (5.5 g, 24.2 mmol, 1.2 eq) was added. The reaction was stirred at RT for 7 hours. After analysis by TLC showed complete consumption of the starting material, the reaction was quenched by saturated aqueous sodium thiosulphate, extracted with DCM and washed with water and brine. The organic layer was dried with anhydrous $MgSO_4$, filtered and concentrated *in vacuo*, and the product purified by column chromatography (PE/EA 10:1 – 4:1) to yield compound **23** (3.1 g, 9.9 mmol, 49%). ^{1}H NMR (400 MHz, Chloroform-*d*) δ 7.56 – 7.10 (m, 10H), 4.69 (d, *J* = 11.6 Hz, 1H, Bn), 4.59 – 4.52 (m, 3H, Bn), 3.65 – 3.47 (m, 5H), 1.64 – 1.31 (m, 6H). ^{13}C NMR (101 MHz, $CDCl_3$) δ 138.9, 138.4, 128.5, 128.4, 127.9, 127.7, 127.7, 127.6, 78.1 (CH), 73.5, 72.9, 72.1, 62.8, 32.8, 31.8,

21.7. HR-MS: Calculated for $C_{20}H_{26}O_3$ [M+Na$^+$]: 337.1774, found: 337.1781. $[\alpha]^{20}_D$ = + 13.7° (c = 1, $CHCl_3$). TLC: Rf = 0.1 (PE/EA = 4/1, v/v).

Phenyl 2-*O*-benzyl-3-*O*-(benzyl 3-*O*-acetyl-2-*O*-benzyl-4-*O*-levulinoyl-α-D-galactopyranosyl uronate)-4,6-*O*-di-*tert*-butylsilylidene-1-thio-β-D-galactopyranoside (19)

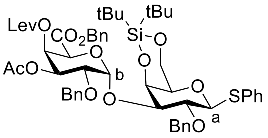

Donor **9** (3.1 g, 4.52 mmol, 1.1 eq) and acceptor **10** (2.0 g, 3.98 mmol, 1.0 eq) were co-evaporated with anhydrous toluene three times under nitrogen. Dry DCM (35 mL) and 5Å molecular sieves were added and then the solution was stirred for 20 minutes at RT. The reaction was cooled to -70 °C and *tert*-butyldimethylsilyl trifluoromethanesulfonate (TBSOTf) (182 µL, 0.79 mmol, 0.2 eq) was added. After stirring overnight TLC showed complete consumption of the starting material, and the reaction was quenched with saturated aqueous sodium bicarbonate and diluted with DCM. The solution was washed with water (2x) and brine. The aqueous layer was extracted with DCM (3x), dried with $MgSO_4$, filtered, and concentrated *in vacuo*. The compound was purified by flash chromatography (PE/EA 5:1 - 3:1) to yield compound **19** (3.0 g, 3.0 mmol, 75%). ^{1}H NMR (500 MHz, Chloroform-*d*) δ 7.59 – 7.48 (m, 4H), 7.35 – 7.23 (m, 13H), 7.22 – 7.17 (m, 2H), 7.17 – 7.10 (m, 1H), 5.63 – 5.58 (m, 1H, H-4b), 5.56 (dd, *J* = 10.5, 3.4 Hz, 1H, H-3b), 5.47 (d, *J* = 3.4 Hz, 1H, H-1b), 5.14 (d, *J* = 12.0 Hz, 1H, CH_2), 5.06 (d, *J* = 9.7 Hz, 1H, CH_2), 4.83 – 4.71 (m, 4H, H-5b, CH_2), 4.70 – 4.65 (m, 2H, H-1a, H-4a), 4.62 (d, *J* = 12.0 Hz, 1H, CH_2), 4.26 – 4.14 (m, 2H, H-6a), 3.96 (dd, *J* = 10.5, 3.3 Hz, 1H, H-2b), 3.89 (t, *J* = 9.5 Hz, 1H, H-2a), 3.71 (dd, *J* = 9.4, 2.9 Hz, 1H, H-3a), 3.32 (d, *J* = 2.3 Hz, 1H, H-5a), 2.55 – 2.32 (m, 3H, Lev), 2.16 – 2.07 (m, 4H, Lev), 1.94 (s, 3H, OAc), 1.09 (s, 9H), 1.02 (s, 9H). ^{13}C NMR (126 MHz, $CDCl_3$) δ 205.9 (Lev), 171.3 (Lev), 170.1 (OAc), 166.9 (CO_2Bn), 137.8, 137.5, 135.0, 132.0, 129.0, 128.9, 128.7, 128.6, 128.5, 128.4, 128.3, 128.0, 127.7, 127.7, 127.5, 92.9 (C-1b, J_{CH} = 171.0 Hz), 88.9 (C-1a, J_{CH} = 157.2 Hz), 77.8 (C-3a), 76.4, 76.1 (C-2a), 74.5 (C-5a), 72.1, 71.9 (C-2b), 69.5 (C-4b), 69.0 (C-3b), 68.6 (C-5b), 68.2 (C-4a), 67.3, 67.2, 37.6, 29.8, 27.7, 27.7, 27.7, 27.5, 23.3, 20.7 (OAc). HR-MS: Calculated for $C_{54}H_{66}O_{14}SSi$ [M+Na$^+$]: 1021.3835, found: 1021.3843. $[\alpha]^{20}_D$ = + 94.3° (c = 1, $CHCl_3$). TLC: Rf = 0.3 (PE/EA = 7/3, v/v).

Phenyl 2-*O*-benzyl-3-*O*-(benzyl 3-*O*-acetyl-2-*O*-benzyl-α-D-galactopyranosyl uronate)-4,6-*O*-di-*tert*-butylsilylidene-1-thio-β-D-galactopyranoside (20)

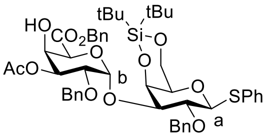

Compound **19** (3.0 g, 3.0 mmol, 1.0 eq) was dissolved in THF (30 mL), MeOH (3 mL) and acetic acid (3 mL). After cooling to 0 °C, hydrazine acetate (N_2H_4 • AcOH) (830 mg, 9.01 mmol, 3.0 eq) was added. After stirring 2 hours at RT, TLC showed complete consumption of the starting material, and the reaction was quenched by acetone. The solution was diluted by EtOAc and then washed with water (2x) and brine. The aqueous layer was extracted with EtOAc (3x), dried with $MgSO_4$, filtered, and concentrated *in vacuo*. The compound was purified by flash chromatography (PE/EA/DCM 10:1:1 – 5:1:1) to yield compound **20** (2.6 g, 2.89 mmol, 95%). ^{1}H NMR (400 MHz, Chloroform-*d*) δ 7.58 – 7.52 (m, 2H), 7.52 – 7.46 (m, 2H), 7.36 – 7.15 (m, 16H), 5.54 (d, *J* = 3.5 Hz, 1H, H-1b), 5.49 (dd, *J* = 10.4, 3.2 Hz, 1H, H-3b), 5.18 (d, *J* = 12.4 Hz, 1H, CH_2), 5.03 (d, *J* = 9.9 Hz, 1H, CH_2), 4.95 (d, *J* = 12.3 Hz, 1H, CH_2), 4.87 – 4.79 (m, 2H, CH_2), 4.75 – 4.65 (m, 3H, H-1a, H-4a, H-5b), 4.56 (d, *J* = 12.1 Hz, 1H, CH_2), 4.32 – 4.26 (m, 1H, H-4b), 4.26 – 4.14 (m, 2H, H-6a), 4.06 (dd, *J* = 10.4, 3.4 Hz, 1H, H-2b), 3.89 (t, *J* = 9.5 Hz, 1H, H-2a), 3.73 (dd, *J* = 9.3, 2.9 Hz, 1H, H-3a), 3.32 (d, *J* = 2.2 Hz, 1H, H-5a), 2.06 (s, 3H, OAc), 1.08 (s, 9H), 1.02 (s, 9H). ^{13}C NMR (101 MHz, $CDCl_3$) δ 167.0(OAc), 168.1 (CO_2Bn), 138.0, 137.8, 132.0, 129.1, 128.9, 128.6, 128.5, 128.5, 128.4, 128.3, 128.1, 127.9, 127.7, 127.5, 127.4, 125.4, 92.4 (C-1b, J_{CH} = 171.0 Hz), 88.8 (C-1a, J_{CH} = 157.0 Hz), 77.7 (C-3a), 76.3, 76.1 (C-2a), 74.5 (C-5a), 72.1 (C-2b), 71.7, 71.2 (C-3b), 69.9 (C-5b), 68.9 (C-4b), 68.2 (C-4a), 67.3 (C-6a), 67.0, 27.7, 27.7, 23.3, 21.0 (OAc), 20.7. HR-MS: Calculated for $C_{49}H_{60}O_{12}SSi$ [M+Na$^+$]: 923.3467, found: 923.3487. $[\alpha]^{20}_D$ = + 104.6° (c = 1, $CHCl_3$). TLC: Rf = 0.3 (PE/DCM/EA = 3/1/1, v/v/v).

Phenyl 2-*O*-benzyl-3-*O*-(benzyl 3-*O*-acetyl-2-*O*-benzyl-4-*O*-(2-azido-3-*O*-levulinoyl-4-*N*-benzyloxycarbonyl-6-deoxy-α-D-galactopyranosyl)-α-D-galactopyranosyl uronate)-4,6-*O*-di-*tert*-butylsilylidene-1-thio-β-D-galactopyranoside (21)

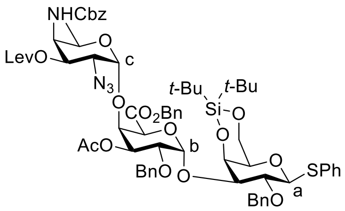

Donor **8**[3] (1.6 g, 2.7 mmol, 1.5 eq) and th acceptor **20** (1.64 g, 1.82 mmol, 1.0 eq) were co-evaporated with anhydrous toluene three times under nitrogen. Dry DCM (18 mL) and 5Å molecular sieves were added and then the solution was stirred for 20 minutes at RT. The reaction was cooled to 0 °C and *tert*-butyldimethylsilyl trifluoromethanesulfonate (TBSOTf) (84 µL, 0.37 mmol, 0.2 eq) was added. After stirring 2 hours TLC showed complete consumption of the starting material, and the reaction was quenched with saturated aqueous sodium bicarbonate and diluted with DCM.

The solution was washed with water (2x) and brine. The aqueous layer was extracted with DCM (3x), dried with $MgSO_4$, filtered, and concentrated *in vacuo*. The compound was purified by flash chromatography (PE/EA/DCM 5:1:1 - 3:1:1) to yield desired α anomer compound **21** (1.6 g, 1.23 mmol, 68%) and byproduct β anomer (332 mg, 0.25 mmol, 14%). α anome: ^{1}H NMR (500 MHz, Chloroform-*d*) δ 7.57 – 7.53 (m, 2H), 7.51 – 7.46 (m, 2H), 7.41 – 7.20 (m, 21H), 5.57 (d, *J* = 3.6 Hz, 1H, H-1b), 5.45 (dd, *J* = 10.7, 2.9 Hz, 1H, H-3b), 5.24 – 5.12 (m, 2H, C*H*2), 5.09 – 4.97 (m, 3H, H-3c, C*H*2), 4.89 – 4.79 (m, 4H, C*H*2), 4.73 (d, *J* = 3.0 Hz, 1H, H-4a), 4.66 (d, *J* = 9.8 Hz, 1H, H-1a), 4.61 (s, 1H, H-5b), 4.54 (d, *J* = 3.9 Hz, 1H, H-1c), 4.49 (d, *J* = 11.9 Hz, 1H, C*H*2), 4.38 (d, *J* = 2.9 Hz, 1H, H-4b), 4.26 – 4.16 (m, 2H, H-6a), 4.16 – 4.10 (m, 1H, H-4c), 4.08 – 4.00 (m, 2H, H-2b, H-5c), 3.89 (t, *J* = 9.6 Hz, 1H, H-2a), 3.73 (dd, *J* = 9.4, 2.9 Hz, 1H, H-3a), 3.29 (d, *J* = 2.2 Hz, 1H, H-5a), 3.11 (dd, *J* = 11.3, 3.9 Hz, 1H, H-2c), 2.84 – 2.37 (m, 4H, Lev), 2.17 (s, 3H, Lev), 1.99 (s, 3H, OAc), 1.06 (d, *J* = 21.5 Hz, 21H, H-6c). ^{13}C NMR (126 MHz, $CDCl_3$) δ 206.4 (Lev), 172.0 (Lev), 170.2 (OAc), 167.2 (CO_2Bn), 156.6 (Cbz), 137.9, 132.0, 128.9, 128.7, 128.7, 128.6, 128.5, 128.5, 128.4, 128.3, 128.1, 127.9, 127.5, 98.5 (C-1c, J_{CH} = 170.8 Hz), 91.9 (C-1b, J_{CH} = 171.5 Hz), 88.9 (C-1a, J_{CH} = 158.0 Hz), 77.6 (C-3a), 76.8 (C-4b), 76.3, 76.0 (C-2a), 74.6 (C-5a), 71.9, 71.7 (C-2b), 70.3 (C-3b), 70.2 (C-3c), 69.6 (C-5b), 68.1 (C-4a), 67.4, 67.3, 67.2, 64.8 (C-5c), 57.6 (C-2c), 52.5 (C-4c), 38.0, 29.9, 28.0, 27.8, 27.7, 23.4, 21.4 (OAc), 20.7, 16.7 (C-6c). HR-MS: Calculated for $C_{68}H_{82}N_4O_{18}SSi$ [M+Na$^+$]: 1325.50063, found: 1325.50063. $[\alpha]^{20}_D$ = + 122.2° (c = 1, $CHCl_3$). TLC: Rf = 0.3 (PE/EA/DCM = 3:1:1, v/v).

Phenyl 2-*O*-benzyl-3-*O*-(benzyl 3-*O*-acetyl-2-*O*-benzyl-4-*O*-(2-azido-3-*O*-levulinoyl-4-*N*-benzyloxycarbonyl-6-deoxy-β-D-galactopyranosyl)-α-D-galactopyranosyl uronate)-4,6-*O*-di-*tert*-butylsilylidene-1-thio-β-D-galactopyranoside (β anomer)

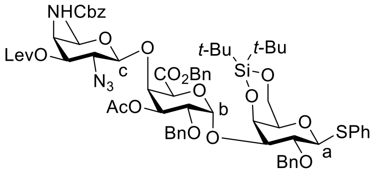

β anomer: ^{1}H NMR (400 MHz, Chloroform-*d*) δ 7.58 – 7.46 (m, 4H), 7.41 – 7.19 (m, 20H), 7.16 – 7.10 (m, 1H), 5.59 (d, *J* = 3.6 Hz, 1H, H-1b), 5.46 (dd, *J* = 10.3, 3.1 Hz, 1H, H-3b), 5.21 – 5.11 (m, 2H, C*H*2), 5.11 – 4.99 (m, 2H, C*H*2), 4.99 – 4.78 (m, 4H, C*H*2), 4.78 – 4.48 (m, 6H, H-5b, H-4a, H-1a, H-3c, H-4b, C*H*2), 4.28 (dd, *J* = 10.4, 3.5 Hz, 1H, H-2b), 4.25 – 4.13 (m, 3H, H-1c, H-6a), 4.09 – 3.98 (m, 1H, H-4c), 3.88 (t, *J* = 9.5 Hz, 1H, H-2a), 3.66 (dd, *J* = 9.3, 2.9 Hz, 1H, H-3a), 3.56 – 3.41 (m, 2H, H-5c, H-2c), 3.33 – 3.24 (m, 1H, H-5a), 2.84 – 2.28 (m, 4H), 2.15

(d, J = 2.0 Hz, 3H, Lev), 2.10 (s, 3H, OAc), 1.15 – 0.98 (m, 21H, H-6c). ^{13}C NMR (126 MHz, $CDCl_3$) δ 206.6 (Lev), 172.2 (Lev), 170.6 (OAc), 167.3 (CO_2Bn), 156.7 (Cbz), 138.1, 137.9, 135.0, 134.9, 132.1, 129.1, 128.9, 128.8, 128.8, 128.7, 128.7, 128.6, 128.5, 128.5, 128.4, 128.4, 128.1, 128.0, 127.8, 127.5, 127.5, 124.9, 103.2 (C-1c, J_{CH} = 163.8 Hz), 92.9 (C-1b, J_{CH} = 172.0 Hz), 88.8 (C-1a, J_{CH} = 157.0 Hz), 78.4 (C-3a), 76.9 (C-4b), 76.4, 76.2 (C-2a), 74.6 (C-5a), 73.1 (C-3c), 73.0 (C-2b), 72.3, 71.5 (C-3b), 69.9 (C-5b), 69.4 (C-5c), 68.5 (C-4a), 67.4, 67.2, 67.0, 61.4 (C-2c), 51.8 (C-4c), 37.9, 30.5, 29.8, 29.8, 27.8, 27.7, 23.5, 22.8, 21.0 (OAc), 20.8, 16.7 (C-6c). HR-MS: Calculated for $C_{68}H_{82}N_4O_{18}SSi$ [M+Na$^+$]: 1325.5006, found: 1325.5015. $[\alpha]^{20}_D$ = + 118.6° (c = 1, $CHCl_3$). TLC: Rf = 0.25 (PE/EA/DCM = 3:1:1, v/v).

2-*O*-benzyl-3-*O*-(benzyl 3-*O*-acetyl-2-*O*-benzyl-4-*O*-(2-azido-3-*O*-levulinoyl-4-*N*-benzyloxycarbonyl-6-deoxy-α-D-galactopyranosyl)-α-D-galactopyranosyl uronate)-4,6-*O*-di-*tert*-butylsilylidene-α/β-D-galactopyranoside (22)

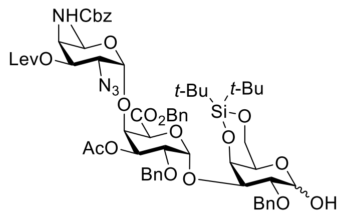

Compound **21** (1.56 g, 1.2 mmol, 1.0 eq) was dissolved in DCM (15 mL) and cooled to 0 °C. NIS (405 mg, 1.8 mmol, 1.5 eq) and TFA (111 μL, 1.44 mmol, 1.2 eq) were added and the solution stirred for 2 hours. After analysis by TLC showed complete consumption of the starting material, the reaction was quenched with triethyl amine and saturated aqueous sodium thiosulphate. The solution was diluted with DCM and washed with brine (3x). The organic phase was dried with $MgSO_4$, filtered, and concentrated *in vacuo*. The compound was purified by flash chromatography (PE/EA 2:1 - 1:1) to yield compound **22** (1.38 g, 1.14 mmol, 95%). ^{1}H NMR (500 MHz, Chloroform-*d*) δ 7.45 – 7.15 (m, 20H), 5.57 – 5.50 (m, 1H), 5.50 – 5.39 (m, 1H), 5.31 – 5.21 (m, 1H), 5.21 – 5.13 (m, 2H), 5.10 – 4.96 (m, 3H), 4.94 – 4.84 (m, 1H), 4.84 – 4.75 (m, 2H), 4.74 – 4.61 (m, 4H), 4.56 – 4.43 (m, 2H), 4.24 – 4.08 (m, 4H), 4.07 – 3.94 (m, 3H), 3.88 – 3.65 (m, 1H), 3.20 – 3.08 (m, 1H), 3.04 (s, 1H), 2.86 – 2.37 (m, 4H), 2.21 – 2.14 (m, 3H), 2.02 – 1.95 (m, 3H), 1.08 – 0.88 (m, 21H). ^{13}C NMR (126 MHz, $CDCl_3$) δ 206.5, 172.0, 170.3, 167.6, 167.4, 156.6, 138.1, 137.8, 137.7, 137.6, 136.3, 134.9, 134.8, 128.8, 128.8, 128.7, 128.7, 128.7, 128.6, 128.4, 128.4, 128.2, 128.2, 128.1, 128.1, 128.0, 128.0, 127.9, 127.8, 98.5, 98.5, 97.9, 92.2, 91.8, 78.0, 76.8, 76.8, 75.8, 75.5, 73.7, 73.6, 72.7, 72.1, 71.9, 71.8, 71.4, 70.3, 70.3, 70.2, 70.2, 69.7, 69.5, 69.2, 68.2, 67.4, 67.3, 67.2, 67.2, 64.8, 57.7, 57.6, 52.5, 52.5, 38.0, 28.0, 27.8, 27.5, 27.3, 23.3, 21.4, 20.7, 16.7. HR-MS: Calculated for $C_{62}H_{78}N_4O_{19}Si$

[M+Na+]: 1233.4922, found: 1233.4943. TLC: Rf = 0.2 (PE/EA/DCM = 1/1/1, v/v/v).

***N*-phenyl-trifluoroacetimidate 2-*O*-benzyl-3-*O*-(benzyl 3-*O*-acetyl-2-*O*-benzyl-4-*O*-(2-azido-3-*O*-levulinoyl-4-*N*-benzyloxycarbonyl-6-deoxy-α-D-galactopyranosyl)-α-D-galactopyranosyl uronate)-4,6-*O*-di-*tert*-butylsilylidene-α/β-D-galactopyranoside (7)**

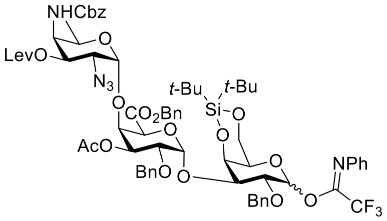

Hemiacetal **22** (1.53 g, 1.26 mmol, 1.0 eq) was dissolved in acetone (13 mL) and cooled to 0 °C. Cesium carbonate (617 mg, 1.89 mmol, 1.5 eq) was added. After 15 min, *N*-phenyl trifluoroacetimidoyl chloride (524 mg, 2.5 mmol, 2.0 eq) was added, and then the reaction was allowed to stir overnight at RT. After analysis by TLC showed complete consumption of the starting material, the reaction was quenched with triethyl amine, and the mixture was filtered and concentrated *in vacuo*, after which the product was purified by column chromatography (PE/EA 4:1 – 3/1) to yield compound **7** (1.64 mg, 1.19 mmol, 94%). ^{1}H NMR (400 MHz, Acetone-d_6) δ 7.49 – 7.21 (m, 22H), 7.19 – 7.10 (m, 1H), 6.95 – 6.76 (m, 2H), 6.48 – 6.36 (m, 1H), 5.60 – 5.46 (m, 2H), 5.30 – 4.75 (m, 12H), 4.70 – 4.52 (m, 2H), 4.41 – 4.09 (m, 7H), 3.71 (dd, J = 11.5, 3.8 Hz, 1H), 2.87 – 2.33 (m, 4H), 2.16 – 2.12 (m, 3H), 2.09 (s, 3H), 1.20 – 1.13 (m, 3H), 1.12 – 0.92 (m, 18H). ^{13}C NMR (101 MHz, Acetone) δ 206.2 206.0, 172.3, 170.6, 170.5, 168.3, 168.1, 157.8, 144.5, 144.3, 139.1, 139.0, 138.6, 138.6, 138.1, 136.2, 136.2, 129.5, 129.5, 129.4, 129.3, 129.2, 129.2, 129.1, 129.1, 129.0, 129.0, 128.9, 128.9, 128.9, 128.8, 128.7, 128.7, 128.7, 128.6, 128.5, 128.4, 128.3, 128.3, 125.1, 120.1, 119.9, 99.6, 99.5, 93.8, 93.7, 77.1, 76.8, 76.5, 75.9, 73.8, 73.3, 73.2, 73.0, 72.9, 72.6, 72.4, 72.4, 71.0, 71.0, 70.3, 70.2, 70.0, 70.0, 69.3, 67.6, 67.4, 67.2, 66.8, 65.8, 58.0, 58.0, 55.3, 53.5, 38.0, 29.8, 29.6, 28.6, 28.0, 27.8, 27.7, 23.6, 23.6, 21.4, 21.3, 21.1, 21.1, 17.0. HR-MS: Calculated for $C_{70}H_{82}F_3N_5O_{19}Si$ [M+Na$^+$]: 1404.5218, found: 1404.5259. TLC: Rf = 0.2 (PE/EA = 3/1, v/v).

(*R*)-5,6-bis(benzyloxy)hexyl 2-*O*-benzyl-3-*O*-(benzyl 3-*O*-acetyl-2-*O*-benzyl-4-*O*-(2-azido-3-*O*-levulinoyl-4-*N*-benzyloxycarbonyl-6-deoxy-α-D-galactopyranosyl)-α-D-galactopyranosyl uronate)-4,6-*O*-di-*tert*-butylsilylidene-α-D-galactopyranoside (24)

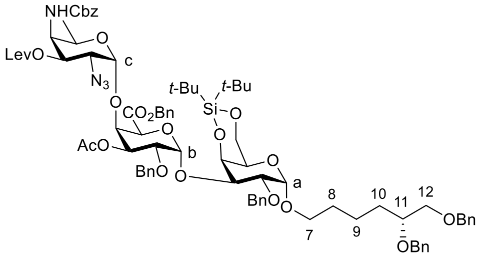

Donor **7** (540 mg, 0.39 mmol, 1.0 eq) and linker acceptor **23** (368 mg, 1.17 mmol, 3.0 eq) were co-evaporated with anhydrous toluene three times under nitrogen. Dry DCM (5 mL) and 4Å molecular sieves were added and then the solution stirred for 20 minutes at RT. The reaction was cooled to 0 °C and *tert*-butyldimethylsilyl trifluoromethanesulfonate (TBSOTf) (18 μL, 0.08 mmol, 0.2 eq) was added. After stirring 2 hours TLC showed complete consumption of the starting material, and the reaction was quenched with saturated aqueous sodium bicarbonate and diluted with DCM. The solution was washed with water (2x) and brine. The aqueous layer was extracted with DCM (3x), dried with $MgSO_4$, filtered, and concentrated *in vacuo*. The compound was purified by flash chromatography (PE/EA/DCM 8:1:1 - 4:1:1) to yield desired α anomer compound **24** (501 mg, 0.33 mmol, 85%). ^{1}H NMR (500 MHz, Chloroform-*d*) δ 7.45 – 7.17 (m, 30H), 5.59 (d, *J* = 3.5 Hz, 1H, H-1b), 5.51 (dd, *J* = 10.7, 2.9 Hz, 1H, H-3b), 5.31 – 5.23 (m, 1H, CH_2), 5.16 (d, *J* = 12.3 Hz, 1H, CH_2), 5.11 – 4.98 (m, 3H, H-3c, CH_2), 4.87 – 4.74 (m, 4H, CH_2, H-5b), 4.72 – 4.60 (m, 5H, CH_2, H-4a, H-1a, H-1c), 4.59 – 4.44 (m, 5H, CH_2, H-4b), 4.19 – 4.09 (m, 3H, CH_2, H-4c, H-5c), 4.09 – 3.99 (m, 3H, CH_2, H-3a, H-2b), 3.95 (dd, *J* = 10.2, 3.7 Hz, 1H, H-2a), 3.61 – 3.47 (m, 5H, H-5a, H-7, H-12, H-11), 3.43 – 3.35 (m, 1H, H-7), 3.16 – 3.10 (m, 1H, H-2c), 2.86 – 2.39 (m, 4H, Lev), 2.18 (s, 3H, Lev), 1.97 (s, 3H, OAc), 1.63 – 1.28 (m, 6H, H-8, H-9, H-10), 1.07 – 0.97 (m, 12H, H-6c), 0.87 (s, 9H). ^{13}C NMR (126 MHz, $CDCl_3$) δ 206.5 (Lev), 172.1 (Lev), 170.2 (OAc), 167.6 (CO_2Bn), 156.7 (Cbz), 139.0, 138.5, 138.4, 138.1, 136.4, 134.9, 128.9, 128.8, 128.6, 128.6, 128.5, 128.5, 128.5, 128.4, 128.1, 127.9, 127.9, 127.9, 127.9, 127.8, 127.8, 127.7, 127.7, 127.6, 98.6 (C-1c, J_{CH} = 171.5 Hz), 97.7 (C-1a, J_{CH} = 167.6 Hz), 92.1 (C-1b, J_{CH} = 171.0 Hz), 78.2 (C-11), 77.0 (C-4b), 73.7, 73.5, 73.2 (C-2a), 73.0 (C-3a), 73.0 (C-12), 72.4 (C-2b), 72.2, 71.7, 70.3 (C-3c), 70.2 (C-3b), 70.0 (C-5b), 69.5 (C-4a), 68.1 (C-7), 67.5 (CO_2Bn), 67.3 (Cbz), 66.9 (C-5a), 64.8 (C-5c), 57.7 (C-2c), 52.6 (C-4c), 38.1 (Lev), 31.9, 29.9 (Lev), 29.5, 28.1 (Lev), 27.9, 27.3, 23.4, 22.0, 21.4 (OAc), 20.7, 16.8 (C-6c). HR-MS: Calculated for $C_{82}H_{102}N_4O_{21}Si$ [M+NH_4^+]: 1524.71440, found: 1524.71125. $[\alpha]^{20}_D$ = + 140.4º (c = 1, $CHCl_3$). TLC: Rf = 0.1 (PE/EA/DCM = 4:1:1, v/v).

(*R*)-5,6-bis(benzyloxy)hexyl 2-*O*-benzyl-3-*O*-(benzyl 3-*O*-acetyl-2-*O*-benzyl-4-*O*-(2-azido-4-*N*-benzyloxycarbonyl-6-deoxy-α-D-galactopyranosyl)-α-D-galactopyranosyl uronate)-4,6-*O*-di-

***tert*-butylsilylidene-α-D-galactopyranoside (25)**

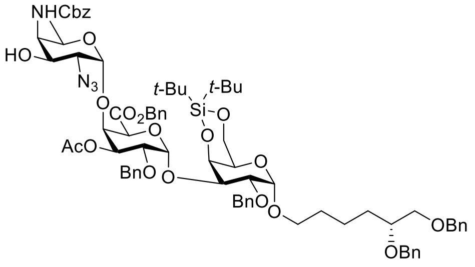

Compound **24** (356.6 mg, 0.237 mmol, 1.0 eq) was dissolved in pyridine (4 mL) and acetic acid (1 mL). After cooling to 0 °C, hydrazine hydrate ($N_2H_4 \cdot H_2O$ 50-60 %) (57 μL, 1.18 mmol, 5.0 eq) was added slowly. After stirring for 20 min at RT, TLC showed complete consumption of the starting material, the reaction was quenched by acetone. The solution was washed with water (2x) and brine. The aqueous layer was extracted with EtOAc (3x), dried with $MgSO_4$, filtered, and concentrated *in vacuo*. The compound was purified by flash chromatography (PE/EA/DCM 10:1:1 – 6:1:1) to yield compound **25** (317 mg, 0.225 mmol, 95%). ^{1}H NMR (400 MHz, Chloroform-*d*) δ 7.41 – 7.14 (m, 30H), 5.60 (d, *J* = 3.6 Hz, 1H, H-1b), 5.54 (dd, *J* = 10.7, 2.8 Hz, 1H), 5.30 – 5.19 (m, 1H), 5.18 – 4.98 (m, 4H), 4.87 – 4.74 (m, 3H), 4.73 – 4.57 (m, 5H, H-1a, H-1c), 4.57 – 4.41 (m, 5H), 4.19 – 3.85 (m, 8H), 3.64 – 3.33 (m, 7H), 2.95 (dd, *J* = 10.7, 3.8 Hz, 1H), 2.00 (s, 3H), 1.65 – 1.28 (m, 6H), 1.06 (d, *J* = 6.5 Hz, 3H), 1.01 (d, *J* = 5.7 Hz, 9H), 0.89 (s, 9H). ^{13}C NMR (101 MHz, $CDCl_3$) δ 170.0, 167.5, 158.1, 138.8, 138.3, 138.2, 138.0, 135.9, 134.8, 128.6, 128.6, 128.5, 128.5, 128.4, 128.3, 128.3, 128.3, 128.3, 128.2, 128.2, 127.7, 127.7, 127.7, 127.6, 127.6, 127.6, 127.5, 127.5, 98.8 (C-1), 97.5 (C-1), 91.9 (C-1), 78.0, 76.9, 73.4, 73.3, 73.0, 72.9, 72.8, 72.1, 72.1, 71.2, 70.2, 69.9, 69.4, 68.2, 67.9, 67.5, 67.3, 67.1, 66.8, 65.0, 60.4, 55.8, 31.7, 29.3, 27.7, 27.2, 23.2, 21.9, 21.3, 20.6, 16.8. HR-MS: Calculated for $C_{77}H_{96}N_4O_{19}Si$ [$M+NH_4^+$]: 1426.67763, found: 1426.67712. $[\alpha]^{20}_D$ = + 120.5° (c = 1, $CHCl_3$). TLC: Rf = 0.25 (PE/DCM/EA = 6/1/1, v/v/v).

(*R*)-5,6-bis(benzyloxy)hexyl 2-*O*-benzyl-3-*O*-(benzyl 3-*O*-acetyl-2-*O*-benzyl-4-*O*-(2-acetylamino-4-*N*-benzyloxycarbonyl-6-deoxy-α-D-galactopyranosyl)-α-D-galactopyranosyl uronate)-4,6-*O*-di-*tert*-butylsilylidene-α-D-galactopyranoside (26)

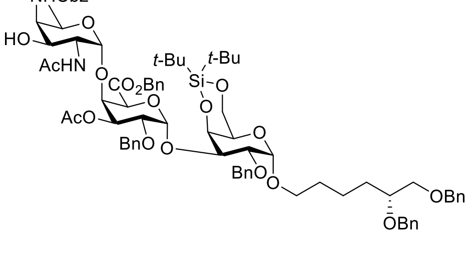

Compound **25** (97 mg, 0.07 mmol, 1.0 eq) was dissolved in THF (2 mL) and water (20 μL). Pyridine (88 μL, 1.1 mmol, 15 eq) and Ph_3P (72 mg, 0.27 mmol, 4.0 eq) were added and the reaction was allowed to stir for 7 h at 70 °C. After TLC showed complete consumption of the starting material, the reaction mixture was concentrated *in vacuo* and co-evaporated with toluene. The residue was

dissolved in THF (2 ml) and water (0.5 mL), then sodium bicarbonate (24 mg, 0.29 mmol, 4.0 eq) and acetic anhydride (14 µL, 0.15 mmol, 2.0 eq) were added and stirred overnight. After TLC showed complete consumption of the starting material, the reaction mixture was diluted with EtOAc and then washed with saturated aqueous sodium bicarbonate and brine. The aqueous layer was extracted with EtOAc (3x), dried with $MgSO_4$, filtered, and concentrated *in vacuo*. The compound was purified by flash chromatography (DCM/Acetone 10:1 – 5:1) to yield compound **26** (98 mg, 0.07 mmol, quantitative). ^{1}H NMR (500 MHz, Chloroform-*d*) δ 7.41 – 7.19 (m, 30H), 5.98 (d, *J* = 8.6 Hz, 1H), 5.61 – 5.50 (m, 2H), 5.41 – 5.17 (m, 2H), 5.15 – 4.89 (m, 3H), 4.88 – 4.72 (m, 3H), 4.72 – 4.59 (m, 4H), 4.58 – 4.43 (m, 4H), 4.38 – 4.20 (m, 2H), 4.18 – 4.10 (m, 1H), 4.09 – 3.78 (m, 8H), 3.76 – 3.68 (m, 1H), 3.65 – 3.47 (m, 5H), 3.45 – 3.36 (m, 1H), 2.14 – 1.97 (m, 6H), 1.65 – 1.28 (m, 6H), 1.12 – 0.83 (m, 21H). ^{13}C NMR (126 MHz, $CDCl_3$) δ 172.6, 170.1, 168.3, 157.6, 138.9, 138.4, 138.3, 137.8, 136.3, 134.0, 129.1, 129.0, 128.9, 128.6, 128.6, 128.5, 128.5, 128.4, 128.4, 128.3, 128.2, 128.2, 127.9, 127.8, 127.8, 127.8, 127.7, 127.6, 98.5, 97.6, 92.3, 78.1, 76.4, 73.6, 73.4, 73.3, 73.1, 72.9, 72.2, 72.1, 71.5, 70.1, 69.9, 69.5, 69.1, 68.1, 67.7, 67.2, 67.2, 66.8, 66.0, 55.4, 50.8, 31.8, 29.4, 27.9, 27.3, 23.4, 23.4, 22.0, 21.4, 20.7, 17.0. HR-MS: Calculated for $C_{79}H_{100}N_2O_{20}Si$ [M+H$^+$]: 1425.67115, found: 1425.67113. $[\alpha]^{20}_D$ = + 104.0° (c = 1, $CHCl_3$). TLC: Rf = 0.4 (DCM/Acetone = 4/1, v/v).

(*R*)-5,6-bis(benzyloxy)hexyl 2-*O*-benzyl-3-*O*-(benzyl 3-*O*-acetyl-2-*O*-benzyl-4-*O*-(2-acetylamino-4-*N*-benzyloxycarbonyl-6-deoxy-α-D-galactopyranosyl)-α-D-galactopyranosyl uronate)-α-D-galactopyranoside (4)

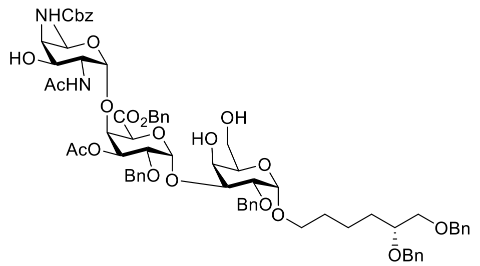

Compound **26** (94 mg, 0.066 mmol, 1.0 eq) was dissolved in THF (1 mL) and pyridine (1 mL), cooled to 0 °C and hydrogen fluoride (HF)/pyridine (70%) (0.1 mL) was added dropwise. The solution was stirred overnight. After TLC showed complete consumption of the starting material, the reaction was quenched with saturated aqueous sodium bicarbonate slowly and diluted with EtOAc. The solution was washed with water (2x) and brine. The aqueous layer was extracted with EtOAc (3x), dried with $MgSO_4$, filtered, and concentrated *in vacuo*. The compound was purified by flash chromatography (DCM/Acetone 3:1 - 2:1) to yield compound **4** (80 mg, 0.062 mmol, 94%). ^{1}H NMR (400 MHz, Chloroform-*d*) δ 7.43 – 7.09 (m, 30H), 5.97 (dd, *J* = 8.2, 4.1 Hz, 1H), 5.37 – 5.26 (m, 2H),

5.21 (d, J = 12.2 Hz, 1H), 5.10 – 4.97 (m, 3H), 4.90 – 4.78 (m, 2H), 4.76 – 4.45 (m, 9H), 4.29 – 4.23 (m, 1H), 4.16 – 4.00 (m, 4H), 3.99 – 3.46 (m, 13H), 3.41 – 3.29 (m, 2H), 2.82 – 2.68 (m, 1H), 2.22 – 1.94 (m, 6H), 1.76 – 1.28 (m, 6H), 1.12 – 0.95 (m, 3H). ^{13}C NMR (101 MHz, $CDCl_3$) δ 173.0, 170.4, 168.0, 157.8, 138.8, 138.4, 138.2, 136.7, 136.3, 134.0, 129.0, 128.9, 128.8, 128.7, 128.6, 128.5, 128.5, 128.4, 128.3, 128.2, 128.1, 127.8, 127.8, 127.8, 127.7, 127.7, 127.7, 98.3, 96.7, 94.2, 78.4, 76.1, 75.1, 74.6, 74.3, 73.4, 72.8, 72.8, 72.4, 72.3, 72.0, 70.5, 70.2, 69.2, 68.8, 67.6, 67.3, 67.1, 66.1, 62.9, 55.3, 50.7, 31.6, 29.3, 23.4, 22.0, 21.4, 17.0. HR-MS: Calculated for $C_{71}H_{84}N_2O_{20}$ [M+H$^+$]: 1285.56902, found: 1285.56928. $[\alpha]^{20}_D$ = + 105.0º (c = 1, $CHCl_3$). TLC: Rf = 0.2 (DCM/Acetone = 2/1, v/v).

Benzyl ((*R*)-5,6-bis(benzyloxy)hexyl 2-*O*-benzyl-3-*O*-(benzyl 3-*O*-acetyl-2-*O*-benzyl-4-*O*-(2-acetylamino-4-*N*-benzyloxycarbonyl-6-deoxy-α-D-galactopyranosyl)-α-D-galactopyranosyl uronate)-α-D-galactopyranosyl uronate) (27)

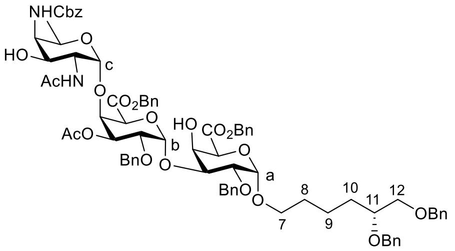

Compound **4** (62 mg, 0.05 mmol, 1.0 eq) was dissolved in DCM/*tert*-BuOH/H_2O (2.25 mL, 4/4/1, v/v/v). The mixture was cooled to 0 °C and treated with TEMPO (2.0 mg, 12.8 µmol, 0.25 eq) and BAIB (40.4 mg, 0.12 mmol, 2.5 eq). After stirring overnight at 4 °C, TLC showed complete consumption of the starting material, and saturated aqueous sodium thiosulphate was added and the mixture was diluted with EtOAc and washed with brine. The organic phase was dried over Na_2SO_4 and concentrated *in vacuo*. The crude residue was dissolved in DMF (2 mL), followed by addition of Cs_2CO_3 (19 mg, 0.06 mmol, 1.2 eq) and BnBr (12 µL, 0.1 mmol, 2.0 eq) at 0°C. After the mixture was allowed to stir overnight at rt and TLC showed complete consumption of the starting material, the reaction was diluted with EtOAc and washed with brine. The organic phase was dried over Na_2SO_4 and concentrated *in vacuo*. Purification by column chromatography (DCM/Acetone 10:1 – 5:1) yielded **27** (54.6 mg, 0.039 mmol, 81%). ^{1}H NMR (500 MHz, Chloroform-*d*) δ 7.42 – 7.08 (m, 35H), 5.90 (d, J = 7.7 Hz, 1H, N*H*Ac), 5.33 (d, J = 12.3 Hz, 1H, C*H*2), 5.27 (dd, J = 10.7, 2.7 Hz, 1H, H-3b), 5.25 – 5.16 (m, 2H, C*H*2), 5.12 (d, J = 9.8 Hz, 1H, C*H*2), 5.07 (d, J = 12.2 Hz, 1H, C*H*2), 5.01 (d, J = 11.8 Hz, 1H, C*H*2), 4.93 (d, J = 3.6 Hz, 2H, H-1b, H-1a), 4.82 (d, J = 11.9 Hz, 1H, C*H*2), 4.70 – 4.61 (m, 2H, C*H*2, H-5b), 4.61 – 4.48 (m, 7H, C*H*2), 4.40 (s, 1H, H-5a), 4.31 – 4.22 (m, 2H, H-4a, H-4b), 4.17 – 4.06 (m, 2H, H-3a, H-1c), 4.06 – 3.98 (m, 2H, H-4c, H-5c), 3.87 (dd, J = 9.8, 3.5 Hz, 1H, H-

2a), 3.81 (dd, J = 10.6, 3.6 Hz, 1H, H-2b), 3.77 – 3.64 (m, 3H, H-3c, H-2c), 3.64 – 3.57 (m, 1H, H-7), 3.57 – 3.45 (m, 3H, H-11, H-12), 3.40 – 3.29 (m, 2H, H-7), 2.11 – 1.98 (m, 6H, NHAc, OAc), 1.61 – 1.49 (m, 4H), 1.49 – 1.38 (m, 1H), 1.38 – 1.28 (m, 1H), 1.06 (d, J = 6.3 Hz, 3H, H-6c). ^{13}C NMR (126 MHz, $CDCl_3$) δ 172.8 (NHAc), 170.0 (OAc), 168.4, 168.0 (CO_2Bn), 157.6 (Cbz), 139.0, 138.5, 138.0, 136.7, 136.3, 135.5, 134.1, 129.0, 129.0, 129.0, 128.9, 128.7, 128.7, 128.6, 128.6, 128.6, 128.5, 128.5, 128.4, 128.4, 128.3, 128.2, 128.0, 127.9, 127.9, 127.9, 127.8, 127.7, 127.7, 127.6, 98.4 (C-1c), 97.1 (C-1a), 94.5 (C-1b), 78.0 (C-11), 76.2 (C-4b), 74.8 (C-3a), 74.1, 74.1 (C-2a), 73.4, 72.9, 72.8, 72.7, 72.2 (C-2b), 72.1, 70.7 (C-3b), 70.1 (C-5b), 69.5, 69.4 (C-5a, H-5c), 68.8 (C-7), 67.7, 67.3 (Cbz), 67.2, 67.1 (C-4a), 66.1 (C-5c), 55.4 (C-4c), 50.9 (C-2c), 31.9 (C-10), 29.5 (C-8), 23.3 (NHAc), 22.0 (C-9), 21.4 (OAc), 17.1 (C-6c). HR-MS: Calculated for $C_{78}H_{88}N_2O_{21}$ [M+H$^+$]: 1389.59523, found: 1389.59579. $[\alpha]^{20}_D$ = + 90.2° (c = 1, $CHCl_3$). TLC: Rf = 0.4 (DCM/Acetone = 4/1, v/v).

(*R*)-5,6-dihydoxyhexyl 3-*O*-(2-*O*-acetyl-4-*O*-(2-acetylamino-4-amino-6-deoxy-α-D-galactopyranosyl)-α-D-galactopyranosyl uronate)-α-D-galactopyranosyl uronate (1)

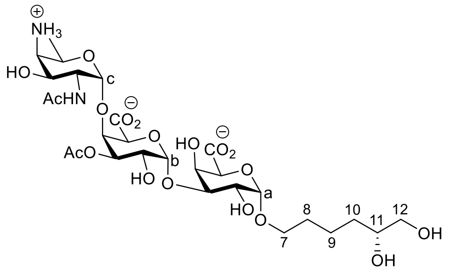

Trimer **27** (15 mg, 10.8 µmol, 1.0 eq) was dissolved in *tert*-butanol (7 mL) and water (3 mL). After $Pd(OH)_2/C$ (60 mg) was added, the reaction was stirred for 3 days under a H_2 atmosphere, after which the mixture was filtered and concentrated *in vacuo* to yield compound **1** (7.5 mg, 10.5 µmol, quantitative). ^{1}H NMR (500 MHz, Deuterium Oxide) δ 5.33 (dd, J = 10.9, 2.9 Hz, 1H, H-3b), 5.30 (d, J = 3.9 Hz, 1H, H-1b), 5.02 – 4.96 (m, 2H, H-1c, H-1a), 4.84 (s, 1H, H-5b), 4.68 – 4.59 (m, 2H, H-5c, H-4b), 4.58 – 4.55 (m, 1H, H-4a), 4.53 (d, J = 1.4 Hz, 1H, H-5a), 4.23 (dd, J = 11.4, 4.4 Hz, 1H, H-3c), 4.18 – 4.12 (m, 1H, H-2b), 4.07 (dd, J = 10.3, 3.1 Hz, 1H, H-3a), 3.98 – 3.91 (m, 2H, H-2c, H-2a), 3.73 – 3.62 (m, 3H, H-7, H-11, H-4c), 3.58 – 3.50 (m, 2H, H-7, H-12), 3.46 – 3.40 (m, 1H, H-12), 2.19 (s, 3H, OAc), 2.03 (s, 3H, NHAc), 1.71 – 1.34 (m, 6H, H-8, H-9, H-10), 1.31 (d, J = 6.7 Hz, 3H, H-6c). ^{13}C NMR (101 MHz, D_2O) δ 174.9, 173.4, 172.7, 172.0, 98.5, 97.9 (C-1a, C-1c), 95.8 (C-1b), 76.3 (C-4b), 74.9 (C-3a), 71.6 (C-11), 71.2 (C-3b), 70.2 (C-5a), 70.1 (C-5b), 68.6 (C-7), 67.0 (C-4a), 66.1 (C-2b), 66.0 (C-2a), 65.4 (C-12), 63.7 (C-3c), 63.3 (C-5c), 55.2 (C-4c), 49.2 (C-2c), 31.9, 28.5, 22.2 (NHAc), 21.4, 20.8 (OAc), 16.0 (C-6c). HR-MS: Calculated for $C_{28}H_{46}N_2O_{19}$ [M+H$^+$]: 715.27675, found: 715.27682.

(*R*)-5,6-dihydoxyhexyl 3-*O*-(4-*O*-(2-acetylamino-4-amino-6-deoxy-α-D-galactopyranosyl)-α-D-galactopyranosyl uronate)-α-D-galactopyranosyl uronate (1b)

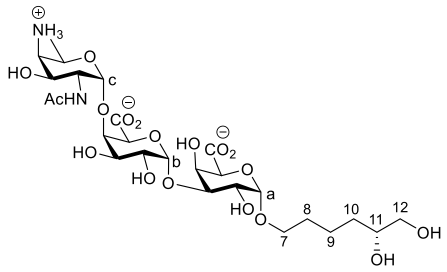

^{1}H NMR (500 MHz, Deuterium Oxide) δ 5.22 (d, *J* = 3.9 Hz, 1H, H-1b), 4.98 – 4.93 (m, 2H, H-1a, H-1c), 4.78 – 4.71 (m, 1H, H-5c), 4.56 (d, *J* = 1.3 Hz, 1H, H-5b), 4.48 (dd, *J* = 3.3, 1.4 Hz, 1H, H-4a), 4.35 (dd, *J* = 3.2, 1.2 Hz, 1H, H-4b), 4.24 (d, *J* = 1.5 Hz, 1H, H-5a), 4.18 (dd, *J* = 11.3, 4.4 Hz, 1H, H-3c), 4.10 (dd, *J* = 10.6, 3.1 Hz, 1H, H-3b), 4.05 – 3.97 (m, 2H, H-3a, H-2c), 3.93 – 3.85 (m, 2H, H-2a, H-2b), 3.74 – 3.65 (m, 2H, H-7, H-11), 3.63 – 3.51 (m, 3H, H-4c, H-12, H-7), 3.49 – 3.41 (m, 1H, H-12), 2.09 (s, 3H, NHAc), 1.73 – 1.34 (m, 6H, H-8, H-9, H-10), 1.25 (d, *J* = 6.7 Hz, 3H, H-6c). ^{13}C NMR (214 MHz, D_2O) δ 176.0, 175.8, 175.1, 99.7 (C-1c, J_{CH} = 174.0 Hz), 99.3 (C-1a, J_{CH} = 172.0 Hz), 97.2 (C-1b, J_{CH} = 170.0 Hz), 80.9 (C-4b), 76.6 (C-3a), 72.6 (C-11), 72.0 (C-5b), 71.8 (C-5a), 69.4 (C-3b), 69.3 (C-7), 68.9 (C-2b), 68.6 (C-4a), 67.4 (C-2a), 66.3 (C-12), 65.5 (C-3c), 64.2 (C-5c), 56.2 (C-4c), 50.3 (C-2c), 32.8, 29.4, 23.2, 22.3, 16.4 (C-6c). HR-MS: Calculated for $C_{26}H_{44}N_2O_{18}$ [M+H$^+$]: 673.26619, found: 673.26633.

Scheme 2. Comparison of ^{1}H NMR chemical shifts of compound **1**, **1a** and **1b** with reported data.

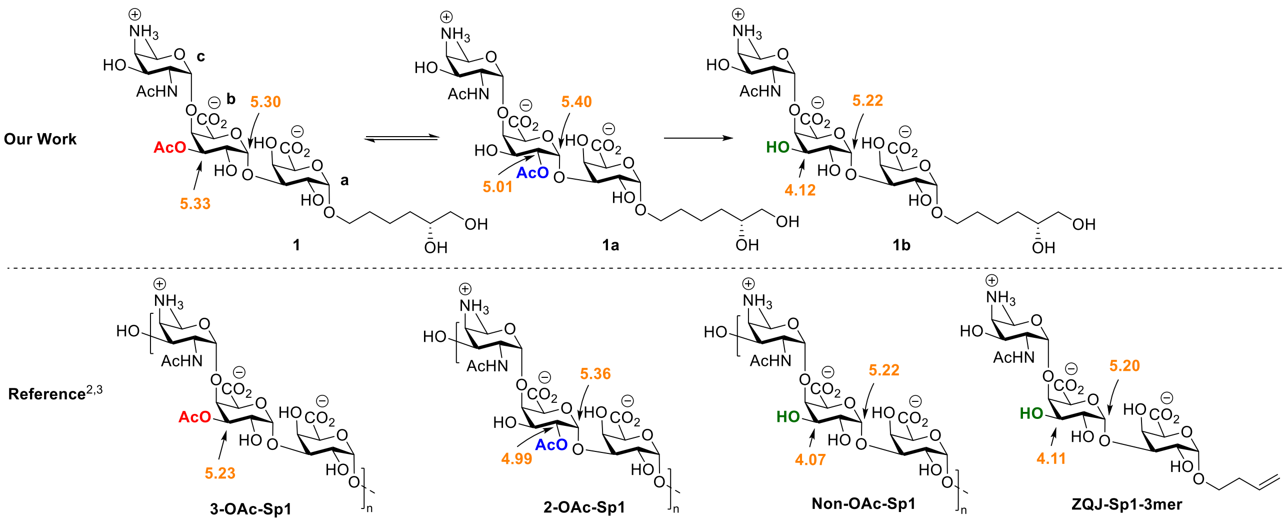

Compound **1** (1.0 mg; 1.4 µmol) was dissolved in different buffers (0.5 mL) and transferred to an NMR tube, and spectra were recorded at increasing points in time. Three representative spectra of mixture are shown in Figure SI-1. After characterization of the NMR and HRMS spectra of **1** and **1b**,

we confirmed that the peaks with chemical shifts 5.33 (dd, J = 10.9, 2.9 Hz, 1H, H-3b), 5.30 (d, J = 3.9 Hz, 1H, H-1b) belong to H-3b and H-1b of compound **1**, and the peak at 5.22 (d, J = 3.9 Hz, 1H, H-1b) belongs to H-1b of compound **1b**, which is consistent with the previously reported data. The two peaks at 5.39 (d, J = 3.9 Hz, 1H) and 5.02 (dd, J = 10.7, 3.9 Hz, 1H) in the mixture are consistent with the anomeric proton (H1b) and H-2b and of an acetylated residue, in line with the previous report. [2] This indicates migration of the 3-OAc takes place to the neighboring C-2OH. We used the integrals of the following diagnostic peaks to calculate the ratio of **1**: **1a** : **1b** (2.21 ppm, s, H-3-OAc; 2.17 ppm, s, H-2-OAc; 1.90 ppm, s, free AcOH).

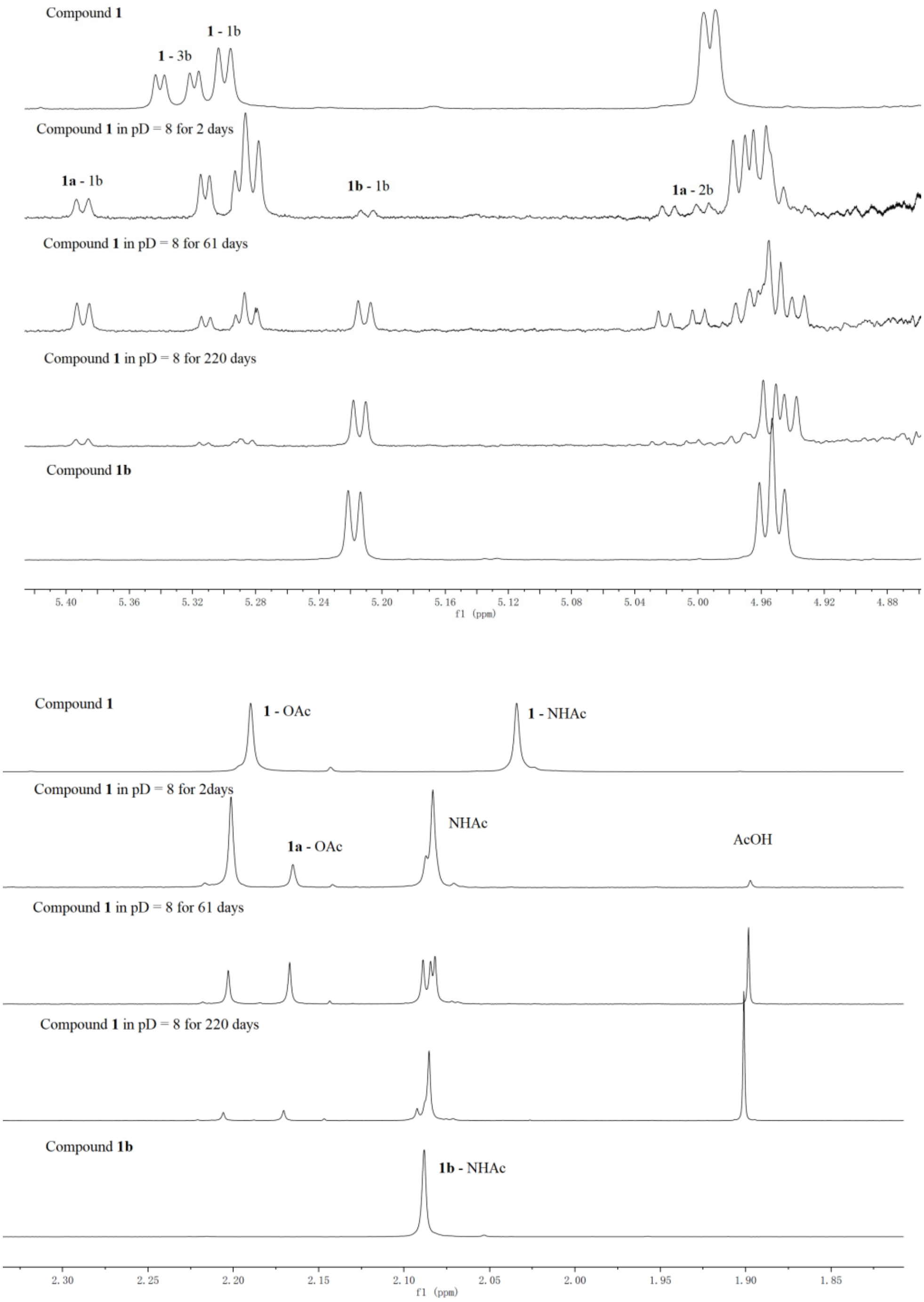

Figure SI-1. Comparison of parts of the spectra of **1** and **1b** and three spectra at intermediate time points of the migration. Top: anomeric region. Bottom: acetyl group region.

Hexamer 28

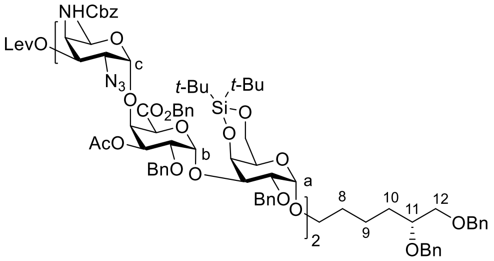

Donor **7** (194 mg, 0.14 mmol, 2.0 eq) and acceptor **26** (99 mg, 0.07 mmol, 1.0 eq) were co-evaporated with anhydrous toluene three times under nitrogen. Dry DCM (3 mL) and 4Å molecular sieves were added and then the solution was stirred for 20 minutes at RT. The reaction was cooled to 0 °C and TBSOTf (3.2 µL, 13.9 µmol, 0.2 eq) was added. After stirring 2 hours TLC showed complete consumption of the starting material, and the reaction was quenched with saturated aqueous sodium bicarbonate and diluted with DCM. The solution was washed with water (2x) and brine. The aqueous layer was extracted with DCM (3x), dried with $MgSO_4$, filtered, and concentrated *in vacuo*. The compound was purified by flash chromatography (Tol/EA 8:1 - 8:3) to yield desired α anomer compound **28** (151 mg, 58 µmol, 83%). ^{1}H NMR (500 MHz, Chloroform-*d*) δ 7.43 – 7.15 (m, 50H), 5.61 (d, J = 3.5 Hz, 1H, H-1), 5.56 (d, J = 3.5 Hz, 1H, H-1), 5.53 – 5.43 (m, 2H), 5.25 – 5.13 (m, 5H, H-1a_1), 5.12 – 4.90 (m, 6H), 4.88 – 4.62 (m, 15H, H-1a), 4.59 (dd, J = 11.5, 3.8 Hz, 2H, H-1c, H-1c_1), 4.56 – 4.40 (m, 8H), 4.29 – 4.09 (m, 7H), 4.09 – 3.97 (m, 6H), 3.97 – 3.86 (m, 2H), 3.71 (s, 1H), 3.62 – 3.44 (m, 5H), 3.43 – 3.35 (m, 1H, H-7), 3.14 (dd, J = 11.3, 4.0 Hz, 1H, H-2c_1), 3.07 (dd, J = 10.8, 3.8 Hz, 1H, H-2c), 2.86 – 2.36 (m, 4H), 2.17 (s, 3H), 2.01 (d, J = 8.7 Hz, 6H), 1.62 – 1.27 (m, 6H), 1.08 – 0.97 (m, 21H), 0.88 (s, 9H), 0.80 (s, 9H). ^{13}C NMR (126 MHz, $CDCl_3$) δ 206.4, 172.0, 170.3, 170.0, 167.4, 167.3, 156.9, 156.6, 138.9, 138.4, 138.3, 138.0, 138.0, 137.9, 136.3, 136.1, 135.0, 134.9, 128.9, 128.7, 128.7, 128.6, 128.5, 128.5, 128.5, 128.4, 128.4, 128.3, 128.3, 128.1, 128.1, 127.8, 127.8, 127.7, 127.7, 127.6, 127.6, 127.6, 127.6, 98.2 (C-1c, C-1c_1), 97.6 (C-1a), 93.5 (C-1a_1), 92.0 (C-1b), 91.7 (C-1b_1), 78.1 (C-11), 77.1, 76.7, 73.5, 73.4, 73.0, 72.9, 72.9, 72.8, 72.6, 72.3, 72.1, 71.4, 71.3, 70.5, 70.3, 70.3, 70.2, 69.9, 69.5, 69.3, 68.1, 67.4, 67.3, 67.2, 67.2, 66.9, 65.9, 64.8, 59.8 (C-2c), 57.7 (C-2c_1), 52.5 (C-4c_1), 50.4 (C-4c), 38.0, 31.8, 29.9, 29.4, 28.0, 27.9, 27.8, 27.3, 27.2, 27.1, 23.3, 21.9, 21.4, 21.3 (OAc), 20.6, 20.5, 16.7, 16.6 (C-6c, C-6c_1). HR-MS: Calculated for $C_{139}H_{172}N_8O_{37}Si_2$ [M+ NH_4^++NH_4^+]/2: 1318.60192, found: 1318.60143. $[\alpha]^{20}_D$ = + 136.5° (c = 1, $CHCl_3$). TLC: Rf = 0.4 (Tol/EA = 8:3, v/v).

Hexamer 29

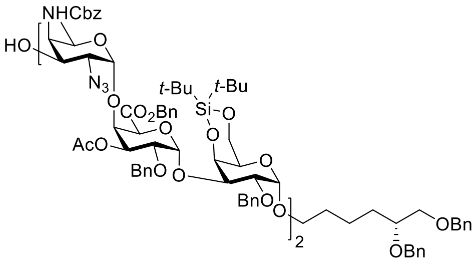

Compound **28** (585.1 mg, 0.225 mmol, 1.0 eq) was dissolved in pyridine (4 mL) and acetic acid (1 mL). After cooling to 0 °C, hydrazine hydrate ($N_2H_4 \cdot H_2O$ 50-60 %) (83 μL, 1.7 mmol, 7.5 eq) was added slowly. After stirring 20 min at RT, TLC indicated complete consumption of the starting material, and the reaction was quenched by acetone. The solution was washed with water (2x) and brine. The aqueous layer was extracted with EtOAc (3x), dried with $MgSO_4$, filtered, and concentrated *in vacuo*. The compound was purified by flash chromatography (DCM/Acetone 30:1 – 20:1) to yield compound **29** (530 mg, 0.212 mmol, 94%). ^{1}H NMR (400 MHz, Chloroform-*d*) δ 7.44 – 7.14 (m, 50H), 5.61 (d, *J* = 3.5 Hz, 1H), 5.55 (d, *J* = 3.5 Hz, 1H), 5.51 – 5.43 (m, 2H), 5.25 – 5.10 (m, 6H), 5.05 (d, *J* = 12.2 Hz, 1H), 5.03 – 4.87 (m, 3H), 4.87 – 4.39 (m, 23H), 4.30 – 4.10 (m, 6H), 4.10 – 3.86 (m, 11H), 3.72 (s, 1H), 3.62 – 3.45 (m, 5H), 3.44 – 3.34 (m, 1H), 3.08 (dd, *J* = 10.8, 3.7 Hz, 1H), 2.97 (dd, *J* = 10.6, 3.8 Hz, 1H), 2.90 (s, 1H), 2.07 – 1.97 (m, 6H), 1.62 – 1.27 (m, 6H), 1.10 – 0.98 (m, 21H), 0.92 – 0.77 (m, 18H). ^{13}C NMR (101 MHz, $CDCl_3$) δ 170.4, 170.1, 167.4, 158.3, 157.0, 139.0, 138.5, 138.3, 138.1, 138.0, 136.2, 135.9, 135.1, 134.9, 128.9, 128.8, 128.8, 128.7, 128.6, 128.6, 128.6, 128.5, 128.5, 128.4, 128.4, 128.3, 128.3, 128.2, 127.9, 127.8, 127.7, 127.7, 127.6, 127.5, 98.6, 98.3, 97.6, 93.5, 92.1, 91.7, 78.2, 76.9, 73.6, 73.4, 73.1, 72.9, 72.4, 72.2, 71.4, 71.1, 70.6, 70.4, 70.0, 69.9, 69.5, 69.4, 69.0, 68.2, 67.7, 67.5, 67.4, 67.3, 66.9, 66.0, 65.1, 60.9, 59.9, 55.8, 50.5, 31.8, 29.5, 28.0, 27.9, 27.3, 27.2, 23.4, 22.0, 21.4, 21.4, 20.7, 20.6, 16.9, 16.6. HR-MS: Calculated for $C_{134}H_{166}N_8O_{35}Si_2$ [M+H$^+$]: 2504.10669, found: 2504.10992. $[\alpha]^{20}_D$ = + 140.9° (c = 1, $CHCl_3$). TLC: Rf = 0.1 (DCM/Acetone = 20/1, v/v).

Hexamer 32

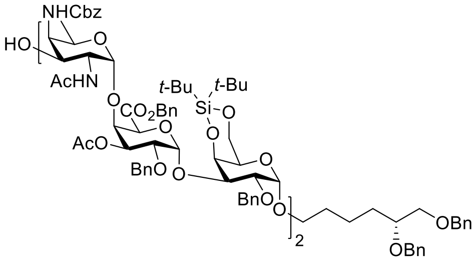

Compound **29** (85 mg, 34 μmol, 1.0 eq) was dissolved in THF (2 mL) and water (20 μL). Pyridine (42 μL, 0.5 mmol, 15 eq) and Ph_3P (37 mg, 0.14 mmol, 4.0 eq) were added and the reaction was allowed to stir for 7 h at 70 °C. After TLC showed complete consumption of the starting material, the reaction mixture was concentrated *in vacuo* and co-evaporated by toluene. The residue was dissolved

in THF (1 ml) and water (0.5 mL), then sodium bicarbonate (12 mg, 0.14 mmol, 4.0 eq) and acetic anhydride (14 µL, 0.15 mmol, 4.0 eq) were added and the mixture was stirred overnight. After TLC showed complete consumption of the starting material, the reaction mixture was diluted with EtOAc and washed with saturated aqueous sodium bicarbonate and brine. The aqueous layer was extracted with EtOAc (3x), dried with $MgSO_4$, filtered, and concentrated *in vacuo*. The compound was purified by flash chromatography (DCM/Acetone 5:1 – 4:1) to yield compound **32** (75.6 mg, 29.8 µmol, 88%). ^{1}H NMR (600 MHz, Chloroform-*d*) δ 7.51 – 6.96 (m, 50H), 6.17 – 6.05 (m, 1H), 5.63 – 5.46 (m, 5H), 5.34 – 4.88 (m, 10H), 4.88 – 4.59 (m, 13H), 4.59 – 4.41 (m, 5H), 4.40 – 4.29 (m, 2H), 4.29 – 3.81 (m, 16H), 3.78 – 3.31 (m, 11H), 2.61 (s, 1H), 2.16 – 1.92 (m, 12H), 1.65 – 1.29 (m, 6H), 1.13 – 0.74 (m, 42H). ^{13}C NMR (151 MHz, $CDCl_3$) δ 172.9, 170.3, 170.2, 169.9, 168.4, 157.6, 157.0, 139.0, 138.5, 138.4, 138.3, 138.0, 137.9, 136.8, 136.4, 134.6, 134.1, 129.2, 128.9, 128.9, 128.8, 128.6, 128.5, 128.5, 128.4, 128.4, 128.3, 128.2, 128.0, 127.9, 127.9, 127.8, 127.7, 127.6, 99.1, 98.5, 97.5, 96.4, 92.8, 91.7, 78.2, 76.6, 73.5, 73.3, 73.2, 73.0, 72.9, 72.9, 72.4, 72.2, 72.1, 71.8, 71.6, 71.2, 70.2, 70.1, 70.0, 69.7, 69.6, 69.3, 68.3, 68.2, 67.8, 67.7, 67.6, 67.3, 67.2, 67.1, 66.9, 66.7, 66.6, 66.0, 55.5, 51.7, 51.1, 48.8, 31.9, 29.5, 27.9, 27.8, 27.3, 27.2, 23.5, 23.4, 23.3, 22.0, 21.4, 21.4, 20.7, 17.0, 16.9. HR-MS: Calculated for $C_{138}H_{174}N_4O_{37}Si_2$ [$M+H^++NH_4^+$]/2: 1277.09032, found: 1277.09029. $[\alpha]^{20}_D$ = + 128.3° (c = 1, $CHCl_3$). TLC: Rf = 0.3 (DCM/Acetone = 4/1, v/v).

Hexamer 5

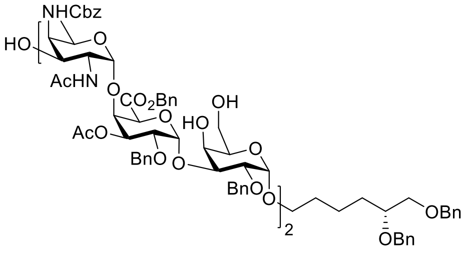

Compound **32** (73 mg, 28.8 µmol, 1.0 eq) was dissolved in THF (1 mL) and pyridine (1 mL), cooled to 0 °C and hydrogen fluoride (HF)/pyridine (70%) (0.1 mL) was added dropwise. The solution was stirred overnight. After TLC showed complete consumption of the starting material, the reaction was quenched with saturated aqueous sodium bicarbonate slowly and diluted with EtOAc. The solution was washed with water (2x) and brine. The aqueous layer was extracted with EtOAc (3x), dried with $MgSO_4$, filtered, and concentrated *in vacuo*. The compound was purified by flash chromatography (DCM/Acetone 4:1 - 1:1) to yield compound **5** (60 mg, 26.6 µmol, 92%). ^{1}H NMR (500 MHz, Chloroform-*d*) δ 7.41 – 7.09 (m, 50H), 6.09 – 5.92 (m, 2H), 5.40 – 4.86 (m, 13H), 4.86 – 4.41 (m, 17H), 4.38 (d, *J* = 3.7 Hz, 1H), 4.33 (d, *J* = 2.9 Hz, 1H), 4.25 (d, *J* = 10.8 Hz, 1H), 4.19 –

3.46 (m, 27H), 3.41 – 3.27 (m, 3H), 2.93 – 2.55 (m, 3H), 2.14 – 1.96 (m, 12H), 1.72 – 1.32 (m, 6H), 1.11 – 0.97 (m, 6H). ^{13}C NMR (126 MHz, $CDCl_3$) δ 173.0, 170.9, 170.0, 169.9, 167.7, 157.6, 156.9, 138.8, 138.3, 138.3, 138.1, 137.0, 136.7, 136.5, 136.3, 134.2, 134.0, 129.1, 128.9, 128.8, 128.8, 128.8, 128.7, 128.7, 128.6, 128.6, 128.5, 128.4, 128.4, 128.2, 128.1, 127.9, 127.7, 127.7, 127.7, 127.7, 127.3, 98.6, 98.3, 96.7, 94.4, 94.3, 78.5, 78.0, 76.2, 75.5, 75.2, 74.6, 74.3, 74.2, 74.1, 74.0, 73.4, 72.9, 72.8, 72.5, 72.3, 72.1, 72.0, 70.7, 70.4, 70.2, 70.1, 69.3, 68.8, 67.6, 67.6, 67.2, 66.9, 66.8, 66.6, 66.0, 63.3, 62.9, 55.3, 51.0, 50.9, 48.5, 31.6, 29.7, 29.3, 23.6, 23.3, 22.0, 21.4, 21.4, 17.0, 17.0. HR-MS: Calculated for $C_{122}H_{142}N_4O_{37}$ [M+2H$^+$]/2: 1128.47492, found: 1128.47435. $[\alpha]^{20}_D$ = + 137.6º (c = 1, $CHCl_3$). TLC: Rf = 0.1 (DCM/Acetone = 3/2, v/v).

Hexamer 34

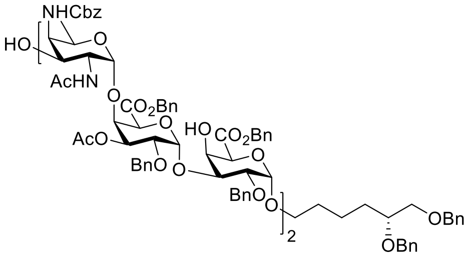

Compound **5** (11.6 mg, 5.14 μmol, 1.0 eq) was dissolved in EtOAc/*tert*-BuOH/H_2O (375 μL, 2/2/1, v/v/v). The mixture was cooled to 0 °C and treated with TEMPO (1.4 mg, 8.96 μmol, 1.7 eq), BAIB (14 mg, 42 mmol, 8 eq) and $NaHCO_3$ (4.5 mg, 53.6 μmol, 10 eq). After stirring for 24 hours at 4 °C saturated aqueous sodium thiosulphate was added and the mixture was diluted with EtOAc and washed with brine. The organic phase was dried over Na_2SO_4 and concentrated *in vacuo*. The crude residue was dissolved in DCM (2 mL), followed by addition of 0.2M phenyldiazomethane ($PhCHN_2$) in Et_2O (1 mL) at RT. After the mixture was allowed to stir overnight at rt and TLC showed complete consumption of the starting material, the reaction was diluted with EtOAc and washed with brine. The organic phase was dried over Na_2SO_4 and concentrated *in vacuo*. Purification by preparative TLC plates (Macherey-Nagel, pre-coated TLC plates SIL G-100 UV254) (DCM/Acetone/MeOH 16:4:0.4) yielded **34** (7.4 mg, 3.0 μmol, 58%). ^{1}H NMR (850 MHz, Chloroform-*d*) δ 7.41 – 7.14 (m, 60H), 5.94 (s, 1H), 5.74 (d, *J* = 9.3 Hz, 1H), 5.32 – 5.28 (m, 2H), 5.27 – 5.22 (m, 3H), 5.20 – 5.14 (m, 2H), 5.13 – 5.10 (m, 1H), 5.02 (d, *J* = 11.8 Hz, 1H), 5.00 – 4.95 (m, 2H), 4.95 – 4.90 (m, 3H), 4.88 – 4.81 (m, 2H), 4.76 (d, *J* = 11.5 Hz, 1H), 4.72 (d, *J* = 12.2 Hz, 1H), 4.67 (d, *J* = 11.7 Hz, 2H), 4.63 (s, 1H), 4.60 – 4.45 (m, 14H), 4.40 – 4.36 (m, 2H), 4.30 – 4.20 (m, 4H), 4.13 – 4.07 (m, 2H), 4.06 – 4.01 (m, 1H), 3.98 – 3.90 (m, 4H), 3.89 – 3.82 (m, 2H), 3.82 – 3.75 (m, 2H), 3.68 – 3.46 (m, 9H), 3.40 – 3.30 (m, 3H), 2.09 (s, 3H), 2.03 – 1.96 (m, 9H), 1.59 – 1.28 (m, 6H), 1.06 – 0.99 (m, 6H). ^{13}C NMR (214 MHz,

$CDCl_3$) δ 173.1, 170.9, 170.2, 169.9, 168.6, 168.5, 167.8, 167.7, 157.6, 156.8, 139.0, 138.5, 138.3, 138.0, 137.0, 136.7, 136.5, 136.2, 135.5, 135.4, 134.1, 134.1, 129.2, 129.0, 129.0, 129.0, 128.9, 128.8, 128.8, 128.8, 128.7, 128.6, 128.6, 128.6, 128.6, 128.5, 128.5, 128.5, 128.5, 128.4, 128.4, 128.1, 127.9, 127.9, 127.8, 127.7, 127.7, 127.6, 127.5, 98.6, 98.1, 97.1, 95.5, 94.5, 78.0, 76.2, 76.1, 74.8, 74.3, 74.1, 74.0, 73.9, 73.5, 73.1, 72.8, 72.8, 72.1, 72.1, 72.1, 70.6, 70.3, 70.3, 70.2, 69.9, 69.5, 68.8, 67.7, 67.6, 67.5, 67.3, 67.2, 67.1, 66.6, 65.9, 55.3, 51.0, 31.9, 29.9, 29.5, 23.5, 23.3, 22.9, 22.0, 21.5, 17.2, 17.1. HR-MS: Calculated for $C_{136}H_{150}N_4O_{39}$ [M+Na$^+$]: 2485.97694, found: 2485.97705. $[\alpha]^{20}_D$ = + 117.0º (c = 1, $CHCl_3$). TLC: Rf = 0.3 (DCM/Acetone/MeOH = 16/4/0.4, v/v/v).

Oxidation side-product 34e

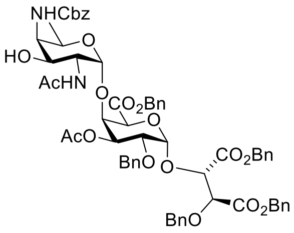

^{1}H NMR (850 MHz, Chloroform-*d*) δ 7.42 – 7.16 (m, 30H), 5.90 (d, *J* = 8.4 Hz, 1H, N*H*Ac), 5.42 (d, *J* = 3.5 Hz, 1H, H-1b), 5.24 (dd, *J* = 10.8, 2.7 Hz, 1H, H-3b), 5.17 (d, *J* = 11.8 Hz, 1H, CH_2), 5.14 – 4.99 (m, 6H, CH_2), 4.92 (d, *J* = 11.8 Hz, 1H, CH_2), 4.86 (d, *J* = 9.8 Hz, 1H, N*H*Cbz), 4.81 (d, *J* = 5.9 Hz, 1H, H-3a), 4.71 – 4.66 (m, 2H, CH_2), 4.60 (s, 1H, H-5b), 4.54 – 4.48 (m, 1H, H-2a), 4.43 (d, *J* = 11.1 Hz, 1H, CH_2), 4.35 (d, *J* = 12.3 Hz, 1H, CH_2), 4.21 – 4.17 (m, 1H, H-4b), 4.08 (d, *J* = 3.8 Hz, 1H, , H-1c), 3.92 – 3.88 (m, 2H, H-5c, H-4c), 3.82 – 3.77 (m, 1H, H-2b), 3.70 – 3.52 (m, 2H, H-3c, H-3c), 3.19 (d, *J* = 6.5 Hz, 1H, 3c-OH), 2.11 – 2.07 (m, 3H, NHAc), 2.03 (s, 3H, OAc), 1.02 (d, *J* = 6.3 Hz, 3H, H-6c). ^{13}C NMR (214 MHz, $CDCl_3$) δ 172.8 (NHAc), 170.1 (OAc), 169.2, 168.2, 168.0, 157.7 (Cbz), 137.6, 136.7, 136.3, 135.2, 134.9, 134.3, 129.0, 129.0, 128.9, 128.9, 128.8, 128.8, 128.8, 128.7, 128.7, 128.6, 128.6, 128.5, 128.5, 128.5, 128.5, 128.4, 128.4, 128.2, 128.2, 127.9, 127.8, 98.4 (C-1c), 95.7 (C-1b), 78.6 (C-2a), 76.2 (C-4b), 75.1 (C-3a), 73.7, 71.7, 71.1 (C-2b), 70.3 (C-5b), 69.8 (C-3b, C-3c), 67.7, 67.6, 67.5, 67.4, 65.9 (C-5c), 55.4 (C-4c), 51.0 (C-2c), 23.4 (NHAc), 21.5 (OAc), 17.1 (C-6c). HR-MS: Calculated for $C_{63}H_{66}N_2O_{18}$ [M+H$^+$]: 1139.43834, found: 1139.43641. $[\alpha]^{20}_D$ = + 63.0º (c = 0.1, $CHCl_3$). TLC: Rf = 0.5 (DCM/Acetone/MeOH = 16/4/0.4, v/v/v).

Hexamer 2

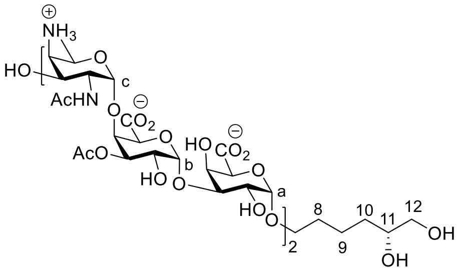

Hexamer **34** (9.8 mg, 4.3 µmol, 1.0 eq) was dissolved in *tert*-butanol (7 mL) and 0.1% AcOH in water (2 mL). After $Pd(OH)_2/C$ (60 mg) was added, the reaction was stirred for 3 days under a H_2 atmosphere, filtered and concentrated *in vacuo* to yield compound **2** (5.1 mg, 3.94 µmol, 91%). ^{1}H NMR (850 MHz, Deuterium Oxide) δ 5.30 – 5.21 (m, 4H, H-3b, H-1b, H-1b$_1$, H-3b$_1$), 5.04 (d, J = 4.2 Hz, 1H, H-1a$_1$), 5.00 (d, J = 4.0 Hz, 1H, H-1c), 4.92 (dd, J = 12.0, 3.9 Hz, 2H, H-1a, H-1c$_1$), 4.66 (s, 1H, H-5b$_1$), 4.62 – 4.49 (m, 5H, H-5c, H-5b, H-4b, H-5c$_1$, H-4b$_1$), 4.47 – 4.41 (m, 2H, H-4a, H-4a$_1$), 4.25 – 4.19 (m, 2H, H-3c, H-5a), 4.16 – 4.06 (m, 5H, H-2b, H-2b$_1$, H-3c$_1$, H-2c, H-5a$_1$), 4.04 – 3.97 (m, 3H, H-3a, H-3a$_1$, H-2c$_1$), 3.94 (dd, J = 10.3, 4.1 Hz, 1H, H-2a$_1$), 3.89 (dd, J = 10.3, 3.9 Hz, 1H, H-2a), 3.74 (s, 1H, H-4c), 3.69 – 3.63 (m, 2H, H-7, H-11), 3.55 – 3.49 (m, 2H, H-12, H-7), 3.49 – 3.43 (m, 1H, H-4c$_1$), 3.44 – 3.39 (m, 1H, H-12), 2.19 – 2.14 (m, 6H, OAc), 2.05 (s, 3H, 2c$_1$-NHAc), 1.99 (s, 3H, 2c-NHAc), 1.68 – 1.34 (m, 6H, H-8, H-9, H-10), 1.32 – 1.23 (m, 6H, H-6c, H-6c$_1$). ^{13}C NMR (214 MHz, D_2O) δ 176.5 (CO_2H), 176.0 (NHAc), 175.9 (CO_2H), 175.6 (NHAc), 175.2 (CO_2H), 175.0 (CO_2H), 174.5 (OAc), 174.4 (OAc), 99.4 (C-1a$_1$, C-1c$_1$), 99.3 (C-1a), 98.6 (C-1c), 97.1 (C-1b$_1$), 96.9 (C-1b), 78.3 (C-4b$_1$), 77.5 (C-4b), 76.8 (C-3a), 76.5 (C-3a$_1$), 74.1 (C-3c), 73.1 (C-5a$_1$), 72.9 (C-3b), 72.8 (C-3b$_1$), 72.6 (C-11), 72.1 (C-5a), 71.9 (C-5b$_1$), 71.7 (C-5b), 69.2 (C-7), 68.7 (C-4a, C-4a$_1$), 67.4 (C-2a), 67.2 (C-2b$_1$), 67.2 (C-2b), 66.6 (C-2a$_1$), 66.3 (C-12), 66.0 (C-3c$_1$), 65.3 (C-5c$_1$), 64.2 (C-5c), 55.8 (C-4c$_1$), 53.6 (C-4c), 50.2 (C-2c$_1$), 48.5 (C-2c), 32.9, 29.5, 23.3, 23.2, 22.4, 21.8, 21.7, 17.0, 17.0 (C-6c, C-6c$_1$). HR-MS: Calculated for $C_{50}H_{78}N_4O_{35}$ [M+2H$^+$]/2: 648.22961, found: 648.22942.

Different catalysts and conditions were explored for the global deprotection of the hexasaccharide as summarized below.

Entry	Catalyst	Acid	Result
**1**	$Pd(OH)_2/C$	No	Mixture
**2**	Pd black	NaHSO4 (4.0 eq)	Mixture
**3**	Pd black	HCl (4.0 eq)	Mixture
**4**	$Pd(OH)_2/C$	0.1% AcOH	Pure compound

Nonamer 30

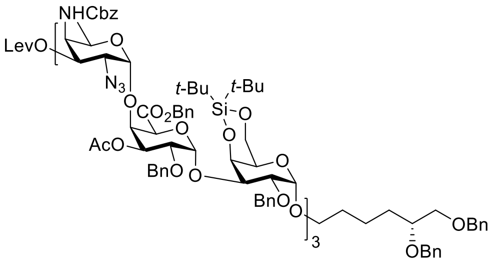

Donor **7** (248 mg, 0.18 mmol, 2.0 eq) and acceptor **29** (224 mg, 0.09 mmol, 1.0 eq) were co-evaporated with anhydrous toluene three times under nitrogen. Dry DCM (3 mL) and 4Å molecular sieves were added and then the solution was stirred for 20 minutes at RT. The reaction was cooled to 0 °C and TBSOTf (5 µL, 19.5 µmol, 0.2 eq) was added. After stirring for 2 hours TLC showed complete consumption of the starting material, and the reaction was quenched with saturated aqueous sodium bicarbonate and diluted with DCM. The solution was washed with water (2x) and brine. The aqueous layer was extracted with DCM (3x), dried with $MgSO_4$, filtered, and concentrated *in vacuo*. The compound was purified by flash chromatography (Tol/EA 8:1 - 8:3) to yield desired α anomer compound **30** (283 mg, 76.5 µmol, 85%). ^{1}H NMR (400 MHz, Chloroform-*d*) δ 7.48 – 7.12 (m, 72H), 5.66 – 5.54 (m, 3H), 5.53 – 5.42 (m, 3H), 5.26 – 4.90 (m, 14H), 4.90 – 4.62 (m, 19H), 4.62 – 4.37 (m, 12H), 4.32 – 3.86 (m, 23H), 3.71 (s, 2H), 3.63 – 3.46 (m, 5H), 3.45 – 3.35 (m, 1H), 3.20 – 3.02 (m, 3H), 2.86 – 2.36 (m, 4H), 2.16 (s, 3H), 2.08 – 1.95 (m, 9H), 1.62 – 1.26 (m, 6H), 1.12 – 0.71 (m, 63H). ^{13}C NMR (101 MHz, $CDCl_3$) δ 206.4, 172.0, 170.3, 170.2, 170.0, 167.4, 167.3, 167.1, 156.9, 156.6, 138.9, 138.4, 138.3, 138.0, 138.0, 137.9, 137.9, 136.3, 136.1, 135.0, 134.9, 134.8, 128.8, 128.7, 128.7, 128.6, 128.6, 128.5, 128.5, 128.4, 128.4, 128.3, 128.3, 128.2, 128.1, 128.0, 127.8, 127.8, 127.8, 127.7, 127.6, 127.6, 127.6, 127.4, 98.2, 98.0, 97.6, 93.5, 92.0, 91.7, 91.6, 78.1, 77.4, 77.0, 76.6, 73.5, 73.4, 73.0, 73.0, 72.9, 72.9, 72.9, 72.8, 72.6, 72.3, 72.1, 72.0, 71.4, 71.3, 71.2, 70.5, 70.3, 70.3, 70.2, 69.9, 69.5, 69.3, 68.2, 68.1, 67.4, 67.2, 67.1, 66.8, 65.9, 64.8, 59.9, 59.8, 57.7, 52.5, 50.4, 38.0, 31.8, 29.8, 29.4, 27.9, 27.9, 27.8, 27.2, 27.1, 27.1, 23.3, 23.3, 21.9, 21.4, 21.3, 20.6, 20.5, 16.7, 16.6, 16.6. HR-MS: Calculated for $C_{196}H_{242}N_{12}O_{53}Si_3$ [M+H$^+$+NH$_4^+$]/2: 1857.31645, found: 1857.32031. $[\alpha]^{20}_D$ = + 161.8° (c = 1, $CHCl_3$). TLC: Rf = 0.3 (Tol/EA = 8:3, v/v).

Nonamer 31

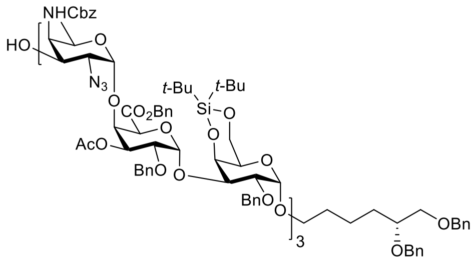

Compound **30** (128 mg, 34.6 µmol, 1.0 eq) was dissolved in pyridine (2 mL) and acetic acid (0.5 mL). After cooled to 0 °C, hydrazine hydrate ($N_2H_4 \cdot H_2O$ 50-60 %) (8.4 µL, 0.17 mmol, 5 eq) was added slowly. After stirred 20 min at RT, checked by TLC complete consumption of the starting material, quenched by acetone. The solution was washed with water (2x) and brine. The aqueous layer was extracted with EtOAc (3x), dried with $MgSO_4$, filtered, and concentrated *in vacuo*. The compound was purified by flash chromatography (DCM/Acetone 25:1 – 15:1) to yield compound **31** (111.4 mg, 30.9 µmol, 89%). ^{1}H NMR (400 MHz, Chloroform-*d*) δ 7.47 – 7.11 (m, 70H), 5.65 – 5.54 (m, 3H), 5.49 (d, *J* = 10.6, 2.2 Hz, 3H), 5.25 – 4.92 (m, 15H), 4.89 – 4.36 (m, 31H), 4.30 – 3.87 (m, 24H), 3.71 (s, 2H), 3.67 – 3.45 (m, 6H), 3.44 – 3.34 (m, 1H), 3.23 – 3.08 (m, 2H), 2.99 (dd, *J* = 10.6, 3.8 Hz, 1H), 2.10 – 1.94 (m, 9H), 1.64 – 1.28 (m, 6H), 1.12 – 0.97 (m, 36H), 0.92 – 0.73 (m, 27H). ^{13}C NMR (101 MHz, $CDCl_3$) δ 170.3, 170.2, 170.0, 167.4, 167.1, 158.2, 157.0, 152.9, 138.9, 138.4, 138.3, 138.0, 138.0, 137.9, 137.9, 136.2, 135.9, 135.0, 134.9, 134.8, 128.8, 128.7, 128.6, 128.6, 128.5, 128.5, 128.5, 128.4, 128.4, 128.4, 128.3, 128.3, 128.3, 128.2, 128.1, 127.8, 127.8, 127.8, 127.7, 127.6, 127.6, 127.6, 127.5, 127.4, 98.6, 98.3, 98.0, 97.6, 93.4, 92.0, 91.7, 91.6, 78.1, 77.4, 77.1, 76.8, 73.5, 73.4, 73.0, 73.0, 72.9, 72.9, 72.8, 72.7, 72.6, 72.4, 72.3, 72.1, 72.0, 71.4, 71.1, 71.0, 70.5, 70.3, 70.0, 69.9, 69.5, 69.4, 69.3, 68.7, 68.1, 67.6, 67.4, 67.3, 67.2, 67.1, 66.8, 65.9, 65.1, 60.8, 59.9, 59.8, 55.8, 50.4, 31.8, 29.4, 28.0, 27.9, 27.8, 27.2, 27.1, 23.3, 23.3, 21.9, 21.4, 21.3, 20.6, 20.5, 16.8, 16.6. HR-MS: Calculated for $C_{191}H_{236}N_{12}O_{51}Si_3$ [M+H$^+$+H$^+$]/2: 1799.78479, found: 1799.78658. $[\alpha]^{20}_D$ = + 139.5° (c = 1, $CHCl_3$). TLC: Rf = 0.5 (DCM/MeOH = 60/1, v/v).

Nonamer 33

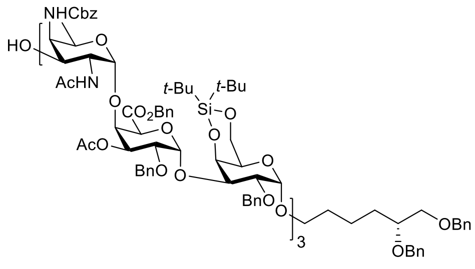

Compound **31** (105 mg, 29.2 µmol, 1.0 eq) was dissolved in THF (2 mL) and water (24 µL). Pyridine (106 µL, 1.31 mmol, 45 eq) and Ph_3P (92 mg, 0.35 mmol, 12.0 eq) were added and the reaction was allowed to stir for 7 h at 70 °C. After TLC showed complete consumption of the starting material, the reaction mixture was concentrated *in vacuo* and co-evaporated by toluene. The residue was dissolved

in THF (3 ml) and water (1 mL), then sodium bicarbonate (30 mg, 0.36 mmol, 12.0 eq) and acetic anhydride (17 µL, 0.18 mmol, 6.0 eq) were added and the mixture was stirred overnight. After TLC showed complete consumption of the starting material, the reaction mixture was diluted with EtOAc and then washed with saturated aqueous sodium bicarbonate and brine. The aqueous layer was extracted with EtOAc (3x), dried with $MgSO_4$, filtered, and concentrated *in vacuo*. The compound was purified by flash chromatography (DCM/MeOH 60:1 – 30:1) to yield compound **33** (106 mg, 29.0 µmol, 99%). ^{1}H NMR (500 MHz, Chloroform-*d*) δ 7.42 – 7.09 (m, 70H), 6.21 – 6.06 (m, 1H), 5.72 (d, *J* = 9.4 Hz, 1H), 5.67 – 5.37 (m, 9H), 5.23 – 5.13 (m, 4H), 5.13 – 4.89 (m, 9H), 4.89 – 4.72 (m, 13H), 4.72 – 4.60 (m, 6H), 4.57 – 4.42 (m, 6H), 4.36 – 4.23 (m, 5H), 4.23 – 3.86 (m, 23H), 3.78 – 3.63 (m, 4H), 3.62 – 3.46 (m, 7H), 3.45 – 3.35 (m, 1H), 2.90 (s, 2H), 2.12 (s, 3H), 2.09 – 1.93 (m, 15H), 1.64 – 1.28 (m, 6H), 1.12 – 0.73 (m, 63H). ^{13}C NMR (126 MHz, $CDCl_3$) δ 172.9, 170.7, 170.4, 170.2, 170.0, 169.8, 168.4, 168.4, 157.6, 157.1, 138.9, 138.4, 138.2, 138.1, 138.1, 137.8, 137.8, 137.8, 136.8, 136.7, 136.3, 134.5, 134.3, 133.9, 129.1, 129.0, 129.0, 129.0, 128.9, 128.9, 128.8, 128.8, 128.6, 128.6, 128.5, 128.5, 128.4, 128.4, 128.4, 128.3, 128.3, 128.2, 128.0, 128.0, 127.9, 127.8, 127.8, 127.8, 127.7, 127.7, 127.6, 127.5, 127.5, 127.4, 99.5, 99.1, 98.5, 97.4, 95.9, 92.8, 91.6, 91.3, 78.1, 77.4, 77.0, 76.7, 73.5, 73.4, 73.4, 73.3, 73.2, 72.9, 72.8, 72.8, 72.4, 72.4, 72.1, 72.1, 72.0, 71.6, 71.3, 71.1, 71.0, 70.4, 70.2, 70.1, 69.9, 69.8, 69.7, 69.2, 68.3, 68.1, 67.8, 67.7, 67.7, 67.3, 67.2, 66.9, 66.7, 66.6, 66.5, 66.0, 55.4, 51.6, 51.3, 51.0, 48.5, 31.8, 29.4, 27.9, 27.9, 27.8, 27.3, 27.1, 27.1, 23.4, 23.4, 23.3, 23.3, 22.0, 21.5, 21.4, 20.7, 20.6, 20.6, 17.0, 17.0, 17.0. HR-MS: Calculated for $C_{197}H_{248}N_6O_{54}Si_3$ $[M+H^++H^+]/2$: 1823.81489, found: 1823.81474. $[\alpha]^{20}_D$ = + 130.8° (c = 1, $CHCl_3$). TLC: Rf = 0.1 (DCM/MeOH = 50/1, v/v).

Nonamer 6

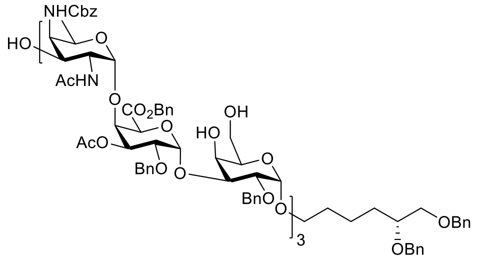

Compound **33** (102 mg, 28.0 µmol, 1.0 eq) was dissolved in THF (2 mL) and pyridine (2 mL), then cooled to 0 °C and hydrogen fluoride (HF)/pyridine (70%) (0.15 mL) was added dropwise. The solution was stirred overnight. After TLC showed complete consumption of the starting material, the reaction was quenched with saturated aqueous sodium bicarbonate slowly and diluted with EtOAc. The solution was washed with water (2x) and brine. The aqueous layer was extracted with EtOAc

(3x), dried with $MgSO_4$, filtered, and concentrated *in vacuo*. The compound was purified by flash chromatography (DCM/Acetone/MeOH 10:3:0.2 – 10:3:0.3) to yield compound **6** (86.2 mg, 26.7 μmol, 96%). ^{1}H NMR (500 MHz, Chloroform-*d*) δ 7.42 – 7.07 (m, 70H), 6.14 – 5.93 (m, 2H), 5.38 – 4.97 (m, 15H), 4.97 – 4.89 (m, 3H), 4.88 – 4.28 (m, 27H), 4.28 – 3.48 (m, 42H), 3.46 – 3.16 (m, 7H), 2.78 (d, *J* = 7.0 Hz, 1H), 2.51 (s, 1H), 2.14 – 1.94 (m, 18H, OAc, NHAc), 1.73 – 1.31 (m, 6H), 1.11 – 0.90 (m, 9H, H-6c). ^{13}C NMR (126 MHz, $CDCl_3$) δ 173.0, 171.1, 170.9, 170.0, 169.9, 169.9, 167.8, 167.7, 167.6, 157.5, 156.9, 138.8, 138.4, 138.4, 138.3, 138.1, 137.0, 136.9, 136.7, 136.4, 136.3, 134.2, 134.0, 134.0, 129.1, 129.0, 128.9, 128.9, 128.9, 128.8, 128.7, 128.7, 128.6, 128.6, 128.5, 128.5, 128.4, 128.4, 128.4, 128.4, 128.2, 128.1, 127.9, 127.7, 127.7, 127.7, 127.7, 127.2, 127.0, 98.7, 98.4, 98.2, 96.7, 95.2, 94.4, 94.2, 78.5, 78.0, 76.5, 76.2, 75.7, 75.2, 74.6, 74.3, 74.2, 74.1, 73.9, 73.4, 72.9, 72.8, 72.7, 72.6, 72.5, 72.5, 72.4, 72.0, 72.0, 71.8, 71.6, 70.8, 70.6, 70.4, 70.3, 70.1, 69.4, 68.8, 67.7, 67.6, 67.6, 67.3, 67.2, 67.0, 66.9, 66.6, 66.4, 66.0, 63.3, 63.0, 62.9, 55.3, 50.9, 48.4, 31.5, 29.7, 29.3, 23.6, 23.5, 23.3, 22.0, 21.4, 21.4, 17.0, 17.0, 16.9. HR-MS: Calculated for $C_{173}H_{200}N_6O_{54}$ [M+2H$^+$]/2: 1613.66170, found: 1613.66372. $[\alpha]^{20}_D$ = + 147.6° (c = 1, $CHCl_3$). TLC: Rf = 0.2 (DCM/Acetone/MeOH 10:3:0.3, v/v/v).

Nonamer 35

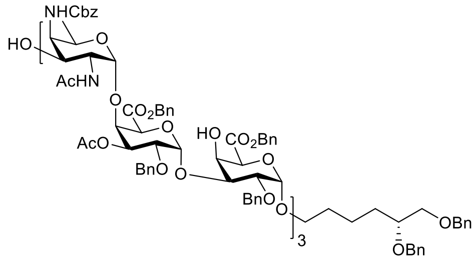

Compound **6** (16.1 mg, 4.99 μmol, 1.0 eq) was dissolved in MeCN/*tert*-BuOH/H_2O (700 μL, 4/1/2, v/v/v). The mixture was cooled to 0 °C and treated with TEMPO (1.9 mg, 12.2 μmol, 2.4 eq), BAIB (20 mg, 0.06 mmol, 12 eq) and $NaHCO_3$ (6.3 mg, 75 μmol, 15 eq). After stirring for 24 hours at 4 °C, saturated aqueous sodium thiosulphate was added and diluted with EtOAc, washed with brine. The organic phase was dried over Na_2SO_4 and concentrated *in vacuo*. The crude residue was dissolved in DCM (2 mL), followed by addition of 0.2M phenyldiazomethane ($PhCHN_2$) in Et_2O (1 mL) at RT. After the mixture was allowed to stir overnight at rt and TLC showed complete consumption of the starting material, the reaction was diluted with EtOAc and washed with brine. The organic phase was dried over Na_2SO_4 and concentrated *in vacuo*. Purification by preparative TLC plates (Macherey-Nagel, pre-coated TLC plates SIL G-100 UV254) (DCM/Acetone/MeOH 10:2:0.5) yielded **35** (11.6 mg, 3.28 μmol, 66%). ^{1}H NMR (600 MHz, Chloroform-*d*) δ 7.49 – 6.99 (m, 85H), 6.03 – 5.79 (m,

3H), 5.40 – 5.20 (m, 10H), 5.20 – 5.09 (m, 4H), 5.07 – 4.80 (m, 10H), 4.77 (d, J = 11.8 Hz, 1H), 4.74 – 4.41 (m, 19H), 4.41 – 4.33 (m, 3H), 4.31 – 4.15 (m, 7H), 4.15 – 3.72 (m, 20H), 3.71 – 3.44 (m, 14H), 3.43 – 3.27 (m, 3H), 2.09 (s, 3H), 2.05 – 1.92 (m, 15H), 1.65 – 1.36 (m, 6H), 1.07 – 0.90 (m, 9H). ^{13}C NMR (151 MHz, $CDCl_3$) δ 173.1, 171.2, 171.0, 170.2, 170.0, 169.9, 168.6, 168.5, 167.8, 167.7, 167.5, 157.6, 156.9, 139.0, 138.5, 138.2, 138.0, 137.0, 136.7, 136.6, 136.5, 136.3, 135.5, 135.4, 135.4, 134.2, 134.1, 134.0, 129.2, 129.1, 129.0, 129.0, 128.9, 128.9, 128.8, 128.8, 128.7, 128.7, 128.6, 128.6, 128.6, 128.6, 128.5, 128.5, 128.5, 128.4, 128.3, 128.1, 128.1, 127.9, 127.9, 127.9, 127.8, 127.8, 127.7, 127.7, 127.6, 127.5, 127.4, 98.7, 98.6, 98.1, 97.1, 95.5, 95.3, 94.5, 78.0, 76.3, 76.2, 76.1, 74.9, 74.3, 74.1, 74.0, 73.8, 73.7, 73.5, 73.1, 73.0, 72.8, 72.7, 72.6, 72.1, 72.1, 70.7, 70.5, 70.3, 70.2, 70.0, 69.8, 69.5, 68.8, 67.8, 67.7, 67.5, 67.3, 67.2, 67.1, 66.6, 65.9, 64.8, 55.3, 52.0, 51.3, 50.9, 48.5, 32.1, 31.9, 29.5, 29.5, 23.5, 23.3, 22.8, 22.0, 21.4, 19.3, 17.2, 17.1. HR-MS: Calculated for $C_{194}H_{212}N_6O_{57}$ $[M+NH_4^++NH_4^+]/2$: 1778.21429, found: 1778.21433. $[\alpha]^{20}_D$ = + 127.1° (c = 1, $CHCl_3$). TLC: Rf = 0.35 (DCM/Acetone/MeOH 10:2:0.5, v/v/v).

Nonamer 3

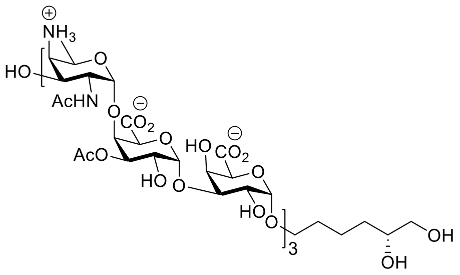

Nonamer **35** (13 mg, 3.68 µmol, 1.0 eq) was dissolved in *tert*-butanol (7 mL) and 0.1% AcOH in water (2 mL). After $Pd(OH)_2/C$ (60 mg) was added, the reaction was stirred for 3 days under a H_2 atmosphere, filtered and concentrated *in vacuo* to yield compound **3** (6.2 mg, 3.3 µmol, 90%). ^{1}H NMR (600 MHz, Deuterium Oxide) δ 5.38 – 5.26 (m, 6H), 5.13 – 5.08 (m, 2H), 5.08 – 5.02 (m, 2H), 5.02 – 4.96 (m, 2H), 4.75 – 4.69 (m, 2H), 4.68 – 4.55 (m, 7H), 4.54 – 4.47 (m, 3H), 4.34 – 4.11 (m, 11H), 4.10 – 3.92 (m, 7H), 3.85 – 3.78 (m, 2H), 3.76 – 3.68 (m, 2H), 3.62 – 3.53 (m, 3H), 3.50 – 3.44 (m, 1H), 2.27 – 2.21 (m, 9H), 2.14 – 2.09 (m, 4H), 2.08 – 2.02 (m, 5H), 1.76 – 1.45 (m, 6H), 1.39 – 1.32 (m, 9H). ^{13}C NMR (151 MHz, D_2O) δ 176.0, 175.6, 175.1, 175.0, 174.5, 174.4, 100.2, 99.8, 99.4, 99.3, 98.7, 98.6, 97.4, 97.0, 96.9, 78.3, 77.7, 77.6, 76.8, 76.5, 74.2, 73.9, 73.2, 72.9, 72.8, 72.7, 72.6, 72.1, 71.9, 71.8, 71.7, 69.2, 68.8, 68.7, 67.5, 67.2, 67.2, 66.7, 66.6, 66.3, 65.6, 64.8, 63.9, 56.0, 53.9, 53.7, 50.3, 48.5, 35.4, 32.9, 29.5, 23.3, 23.2, 22.4, 21.7, 21.7, 16.9. HR-MS: Calculated for $C_{72}H_{110}N_6O_{51}$ $[M+2H^+]/2$: 938.31720, found: 938.31596.

Structural studies

NMR Experiments. General Remarks

NMR. All NMR experiments were performed at 25 ºC on a Bruker AVANCE 800 MHz spectrometer equipped with a cryoprobe. The ^{1}H-NMR resonances of the compounds were assigned through standard TOCSY (60 and 90 ms mixing times), NOESY (50-500 ms mixing times), and HSQC experiments. 500 µL samples were prepared by dissolving the purified compound in phosphate buffered saline 1x pH 7.4 prepared in D_2O.

Molecular Modeling and MD Simulations of sugars 1-3

Molecular models for (GalNAcα1-4GalAα1-3GalAα1-4-)$_n$, where n = 1, 2 and 3 were built using the carbohydrate builder module available in the GLYCAM web portal (Glycam Biomolecule Builder), www.glycam.org. The chemical structures of OAc-Sp1 analogues **1-3** (AATα1-4GalA(3OAc)α1-3GalAα1-4-)$_n$, with n =1, 2, and 3, were produced modifying the existing residues by the use of the edit menu available in xleap. Atom type and charge of trideoxygalactosamine residue atoms were defined based on the Protonated Alpha-D-Glucosamine prep file. The MD simulations were performed using Amber16 program with the GLYCAM06j-1 and frcmod.UnsaturatedRing-ProtonatedUA force field parameters. Thereafter, the starting 3D geometries were placed into a 12Å octaedral box of explicit TIP3P waters, and counterions were added to maintain electroneutrality. Na^+ cations were introduced according to the employed experimental conditions. Two consecutive minimizations were performed: 1) involving only the water molecules and ions, and 2) involving the whole system. Molecular dynamics simulations without constraints were recorded. The system was then heated and equilibrated in two steps: 1) 20 ps of MD heating the whole system from 0 to 300 K, followed by 2) equilibration of the entire system during 100 ps at 300 K. The equilibrated structures were the starting points for MD simulations (500 ns) at constant temperature (300 K) and pressure (1 atm). A detailed analysis of each MD trajectory (for example r.m.s.d. evaluation, dihedral angles and proton-proton distances) was accomplished using the cpptraj module included in Amber-Tools 16 package. The ensemble of structures obtained from the MD simulation was in agreement with the experimental data.

O-glycoside conformation analysis

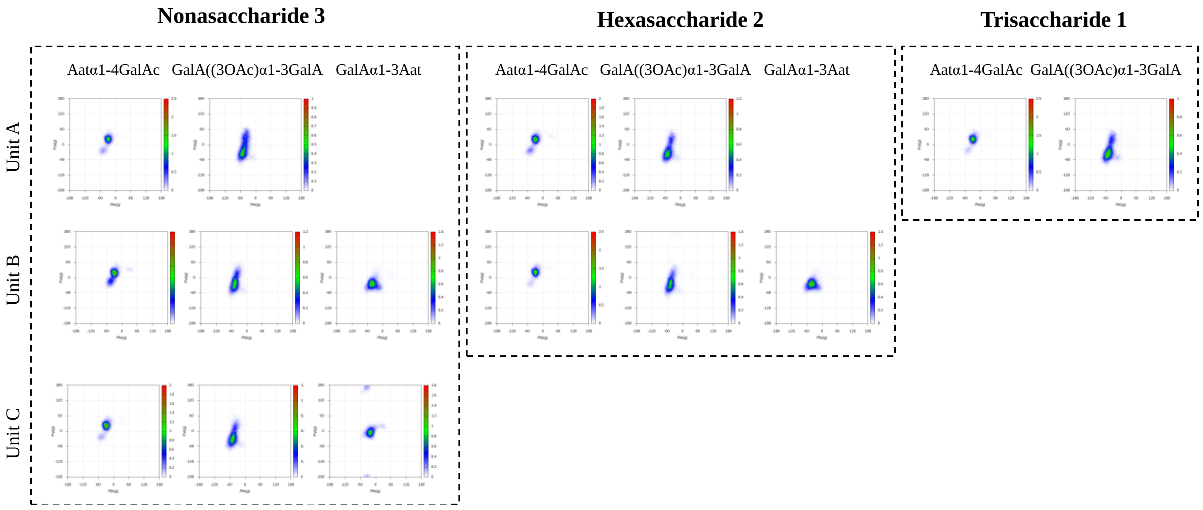

Figure SI-2. Plots of Φ/Ψ values explored along the 500 ns MD trajectory for compounds **1-3**. Points were colored as function of the population density.

Inter-residue distance and RoG analysis

A

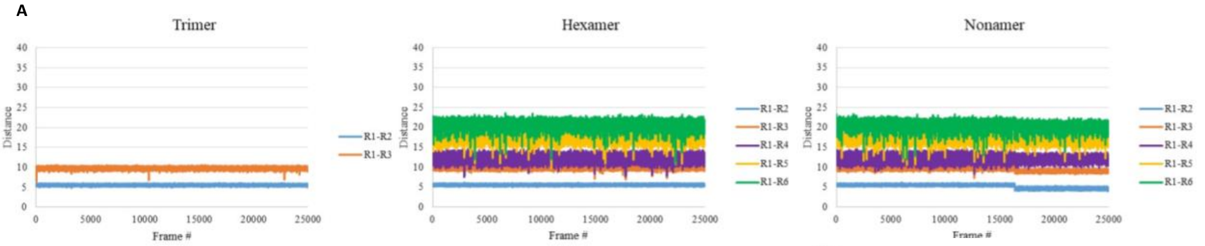

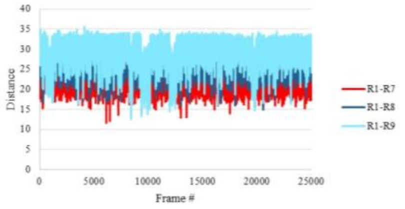

B

Average Values	Trimer (1)	Hexamer (2)	Nonamer (3)
Distance R9-R8	--	--	4.46 (0.12)
Distance R8-R7	--	--	5.68 (0.13)
Distance R7-R6	--	--	4.90 (0.12)
Distance R6-R5	--	4.46 (0.12)	4.38 (0.12)
Distance R5-R4	--	5.69 (0.13)	5.70 (0.12)
Distance R4-R3	--	4.89 (0.14)	4.90 (0.14)
Distance R3-R2	4.46 (0.12)	4.45 (0.12)	4.45 (0.12)
Distance R2-R1	5.67 (0.14)	5.68 (0.13)	5.36 (0.47)

Figure SI-3. A) Inter-residue distances (R_n-R_{n-1}, R_n-R_{n-2}…R_n-R_1, where n = 3, 6, 9) along 500 ns MD trajectories ($2.5 \cdot 10^4$ frames). B) Averaged Inter-residue distances R_n-R_{n-1} for compounds **1-3,** measured from the centre of mass of every residue. The standard deviation is indicated in parentheses. R1 is the residue at the reducing end.

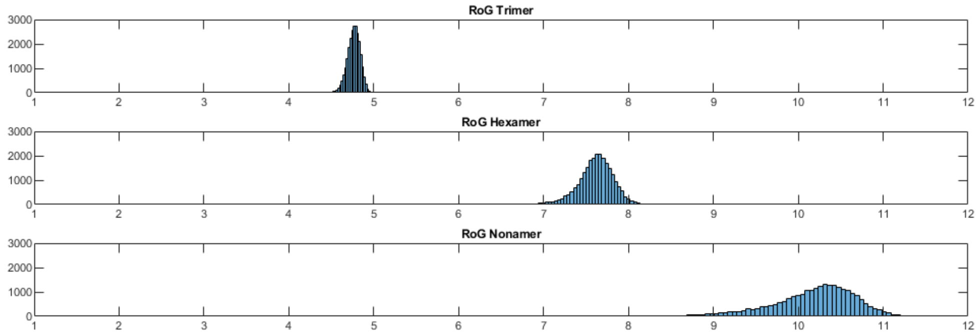

Figure SI-4. RoG histograms for compounds **1-3**.

Main Conformers of oligosaccharides 1-3

Similarly, to the features observed for deacetylated Sp1 oligosaccharides MD simulation analysis of compounds 1-3 indicated the existence of two main conformations around the glycosidic linkage GalA(3OAc)α1-3GalA. For comparison, most disparate shapes displaying all GalA(3OAc)α1-3GalA linkages with conformations exo-*syn-Φ/syn(-)*-Ψ or exo-*syn-Φ/syn(+)*-Ψ, respectively, are depicted for each oligosaccharide. Structures displaying different conformations of the GalAα1-3GalA linkage in each repeating unit were omit for simplicity.

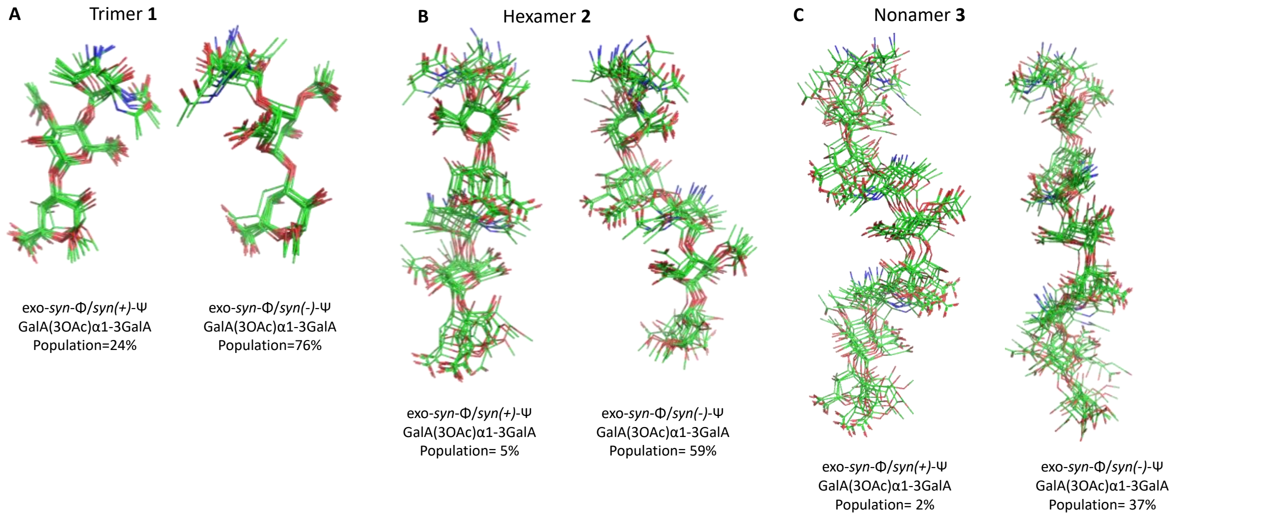

Figure SI-5. Superimposition of 10 MD frames with exo-*syn-Φ/syn(-)*-Ψ and exo-*syn-Φ/syn(+)*-Ψ conformations of the linkages GalA(3OAc)α1-3GalA for a) trimer **1**, b) hexamer **2** and c) nonamer **3**. The population of each conformer was indicated.

Conformational Analysis of compounds 1-3

Trimer 1

2D-NOESY experiments of compound **1** were acquired at 800MHz (mixing time 400ms). The trisaccharide displayed negative NOEs. NOE-derived distances for proton-proton pairs were approximately estimated following the isolated spin pair approximation and compared with the average distance predicted from 500ns MD simulations. The results are shown in table SI-1.

Table SI-1.

Proton	Proton	Rel. NOE cross-peak volume	NOE-derived distance (Å)	MD average distance (Å)
		500 ms		
**AAT**				
H1	H4, GalA(3OAc)	0.9008	2.39	2.35
	H2, AAT	1.00	2.35 (as reference)	2.35
**GalA(3OAc)**				
H1	H2, GalA(3OAc)	1.00	2.38 (as reference)	2.38
	H3, GalA	0.8258	2.46	2.69
	H4, GalA	0.7045	2.52	2.64

Hexamer 2

2D-NOESY experiments of compound **2** were acquired at 800MHz (mixing time 200ms). The hexasaccharide displayed negative NOEs. NOE-derived distances for proton-proton pairs were approximately estimated following the isolated spin pair approximation and compared with the average distance predicted from 500ns MD simulations. The results are shown in table SI-2.

Table SI-2.

Proton	Proton	Rel. NOE cross-peak volume	NOE-derived distance (Å)	MD average distance (Å)
		500 ms		
**AAT(B)**				
H1	H4, GalA(3OAc)	1.1081	2.31	2.35
	H2, AAT	1.00	2.35 (as reference)	3.11
**GalA(3OAc)(B)**				
H1	H2, GalA(3OAc)	1.00	2.38 (as reference)	2.38
	H3, GalA	0.8258	2.48	2.59
	H4, GalA	0.7045	2.60	2.79
**GalA1(B)**				
H1	H2, GalA(B)	1.00	2.36 (as reference)	2.36
	H3, AAT(A)	1.5466	2.19	2.52
	H4, AAT (A)	0.6504	2.54	2.51
**AAT(A)**				
	H2, AAT	1.00	2.35 (as reference)	2.35
	H4, GalA(3OAc)	1.1472	2.30	2.38
**GalA(3OAc)(A)**				
H1	H2, GalA(3OAc)	1.00	2.38 (as reference)	2.38
	H3, GalA	0.9855	2.39	2.58
	H4, GalA	0.8592	2.44	2.74

Nonamer 3

2D-NOESY experiments of compound **3** were acquired at 800MHz (mixing time 200ms). The nonasaccharide displayed negative NOEs. NOE-derived distances for proton-proton pairs were approximately estimated following the isolated spin pair approximation and compared with the average distance predicted from 500ns MD simulations. The results are shown in table SI-3.

Table SI-3.

Proton	Proton	Rel. NOE cross-peak volume	NOE-derived distance (Å)	MD average distance (Å)
500 ms				
**AAT(C)**				
H1	H4, GalA(3OAc)	0.4835	2.65	2.35
	H2, AAT	1.00	2.35 (as reference)	2.35
**GalA(3OAc)(C)**				
H1	H2, GalA(3OAc)	1.00 (overlapped)	2.38 (as reference)	2.38
	H3, GalA	0.7559	2.49	2.54
	H4, GalA	0.6574	2.55	2.71
**GalA(C/B)**				
H1	H2, GalA1(C/B)	1.00	2.36 (as reference)	2.36
	H3, AAT(B/A)	1.5472	2.19	2.51
	H4, AAT(B/A)	0.7529	2.47	2.54
**AAT(B)**				
	H2, AAT	1.00	2.35 (as reference)	2.35
	H4, GalA(3OAc)	1.6355	2.17	2.39
**GalA(3OAc) (B/A)**				
H1	H2, GalA(3OAc)	1.00	2.38 (as reference)	2.38
	H3, GalA	0.9372	2.41	2.51
	H4, GalA	1.0214	2.37	2.81
**AAT(A)**				
	H2, AAT	1.00	2.35 (as reference)	2.35
	H4, GalA(3OAc)	1.0891	2.35	2.39

Comparison of OAc-Sp1 and de-OAc-Sp1 structures

To compare the conformational features of the OAc- and de-OAc Sp1 oligosaccharides, the flexibility around the different glycosidic linkages was analyzed. Both oligosaccharides showed similar conformational preferences and flexibility. Below the data corresponding to non-acetylated Sp1 oligosaccharides[3] have been shown for comparison purposes (Figure SI-6A). In addition, to make the similarity between the structures more visual, a superimposition of de-OAc- and OAc-Sp1 nonamer structures has been included (see Figure SI-6B).

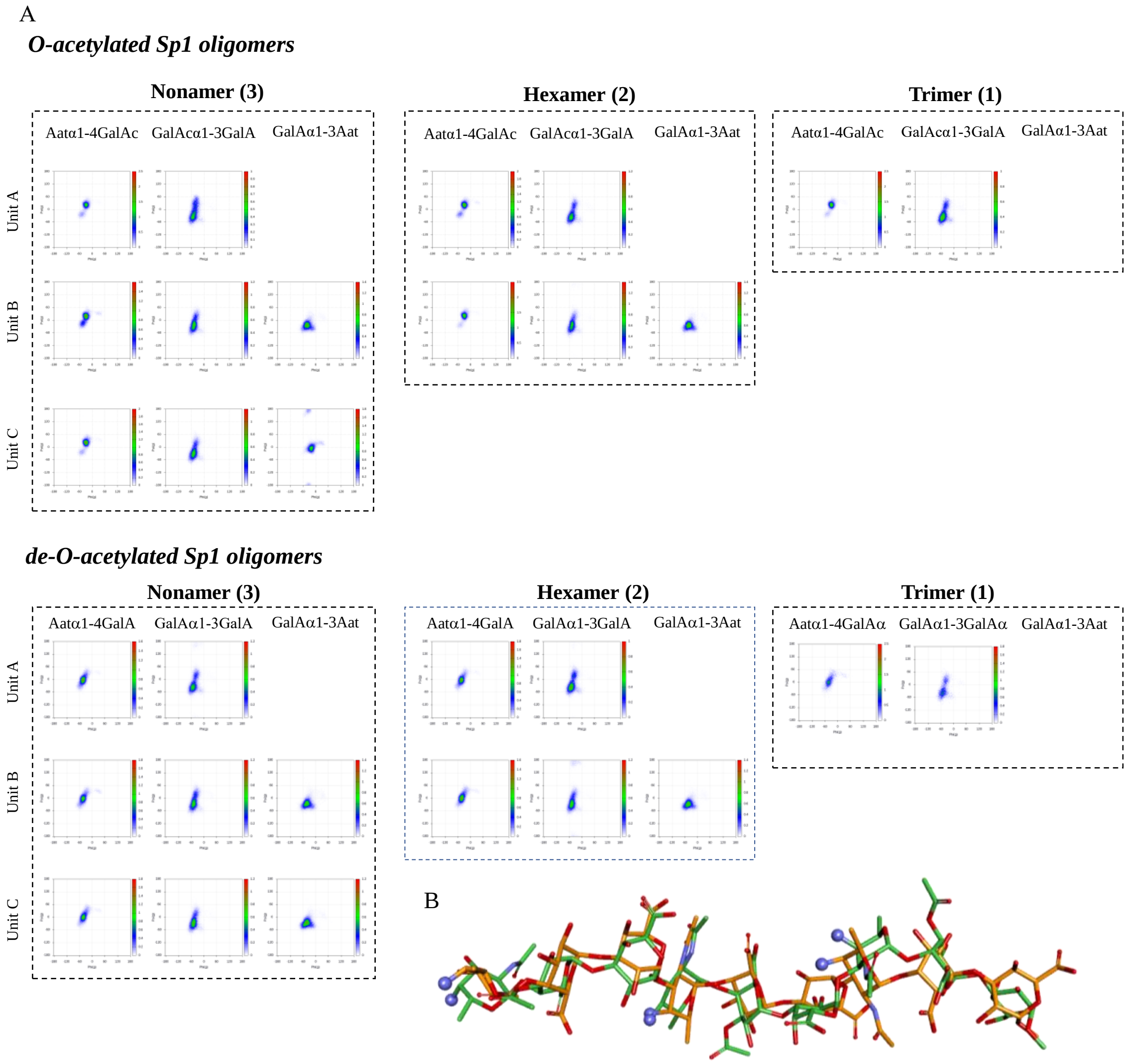

Figure SI-6. A) Plots of Φ/Ψ values explored along the 500 ns MD trajectory for the O-acetylated and de-O-acetylated Sp1 oligosaccharides. Points were colored as function of the population density. B) Superimposition of 500ns MD snapshot obtained for OAc-Sp1 nonamer (in green) and de-OAc-Sp1 nonamer (in orange). Amino groups in AAT residues have been highlighted as blue balls.

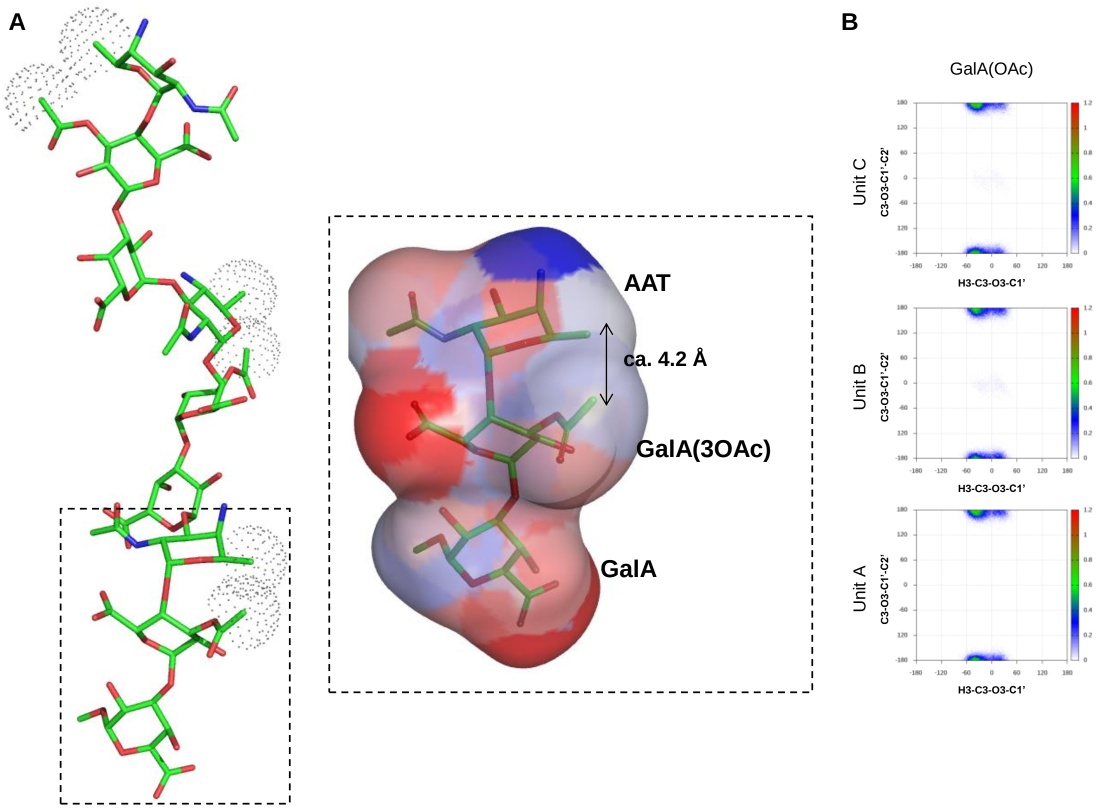

Figure SI-7. A) Molecular model of nonamer 3 where acetyl and methyl groups are highlighted as balls. Atom charge surface representation showed the existence of hydrophobic regions around O-acetyl and methyl groups of adjacent GalA and AAT residues. B) Plots of H3-C3-O3-C1' and C3-O3-C1'-C2' torsion angles defining the orientation of O-acetyl groups on GalA(3OAc) residues.

References

(1) Emmadi, M.; Kulkarni, S. S. Synthesis of orthogonally protected bacterial, rare-sugar and D-glycosamine building blocks. *Nat. Protoc.* **2013,** *8* (10), 1870.

(2) Stroop, C. J. M.; Xu, Q.; Retzlaff, M.; Abeygunawardana, C.; Bush, C. A. Structural analysis and chemical depolymerization of the capsular polysaccharide of Streptococcus pneumoniae type 1. *Carbohydr. Res.* **2002,** *337* (4), 335.

(3) Zhang, Q.; Gimeno, A.; Santana, D.; Wang, Z.; Valdes-Balbin, Y.; Rodriguez-Noda, L. M.; Hansen, T.; Kong, L.; Shen, M.; Overkleeft, H. S.et al. Synthetic, Zwitterionic Sp1 Oligosaccharides Adopt a Helical Structure Crucial for Antibody Interaction. *ACS Cent Sci* **2019,** *5* (8), 1407.

(4) Cheng, B.; Liu, W.; Lu, Z. Iron-Catalyzed Highly Enantioselective Hydrosilylation of Unactivated Terminal Alkenes. *J. Am. Chem. Soc.* **2018,** *140* (15), 5014.

(5) Smith, A. B.; Chen, S. S. Y.; Nelson, F. C.; Reichert, J. M.; Salvatore, B. A. Total Syntheses of (+)-Acutiphycin and (+)-trans-20,21-Didehydroacutiphycin. *J. Am. Chem. Soc.* **1997,** *119* (45), 10935.

zhen1910biosyn.93.fid — wz494 — bbo-h1 CDCl3 /opt/topspin2.1 nmrafd 9

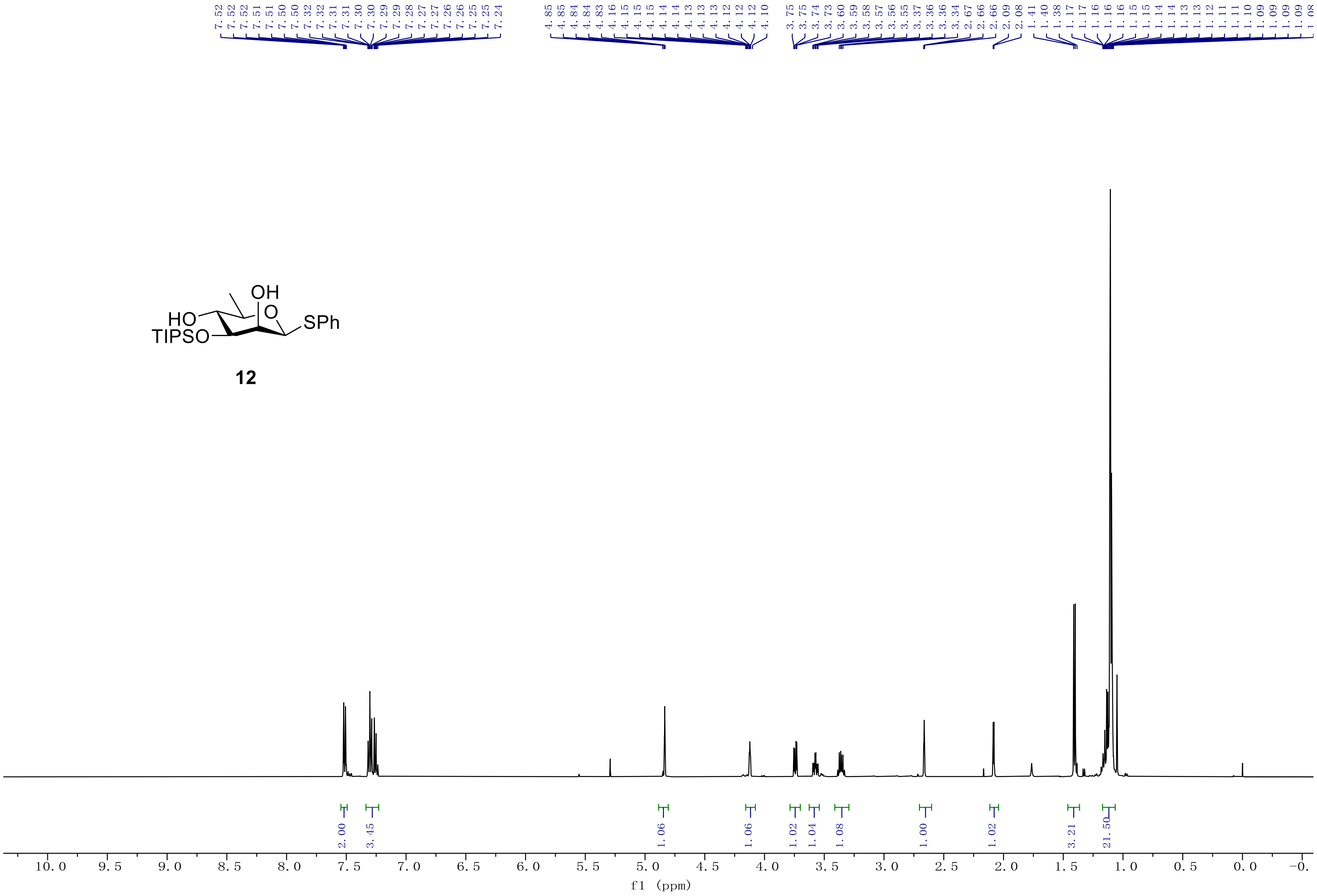

zhen1910biosyn.96.fid — wz494 — bbo-c13-APT CDCl3 /opt/topspin2.1 nmrafd 9

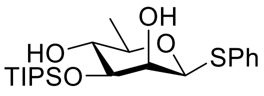

12

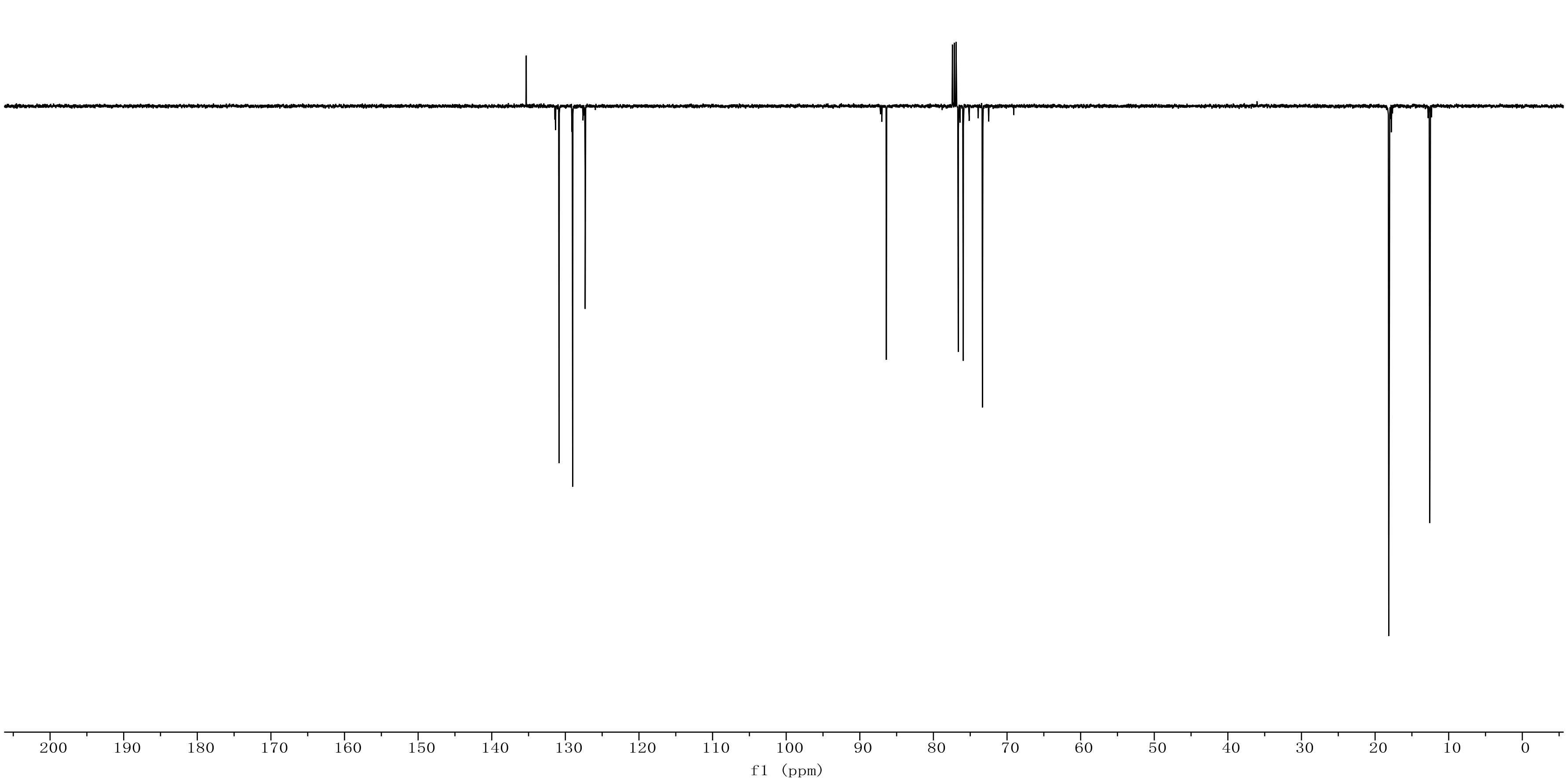

zhen1910biosyn.94.ser — wz494 — bbo-h1-cosy CDCl3 /opt/topspin2.1 nmrafd 9

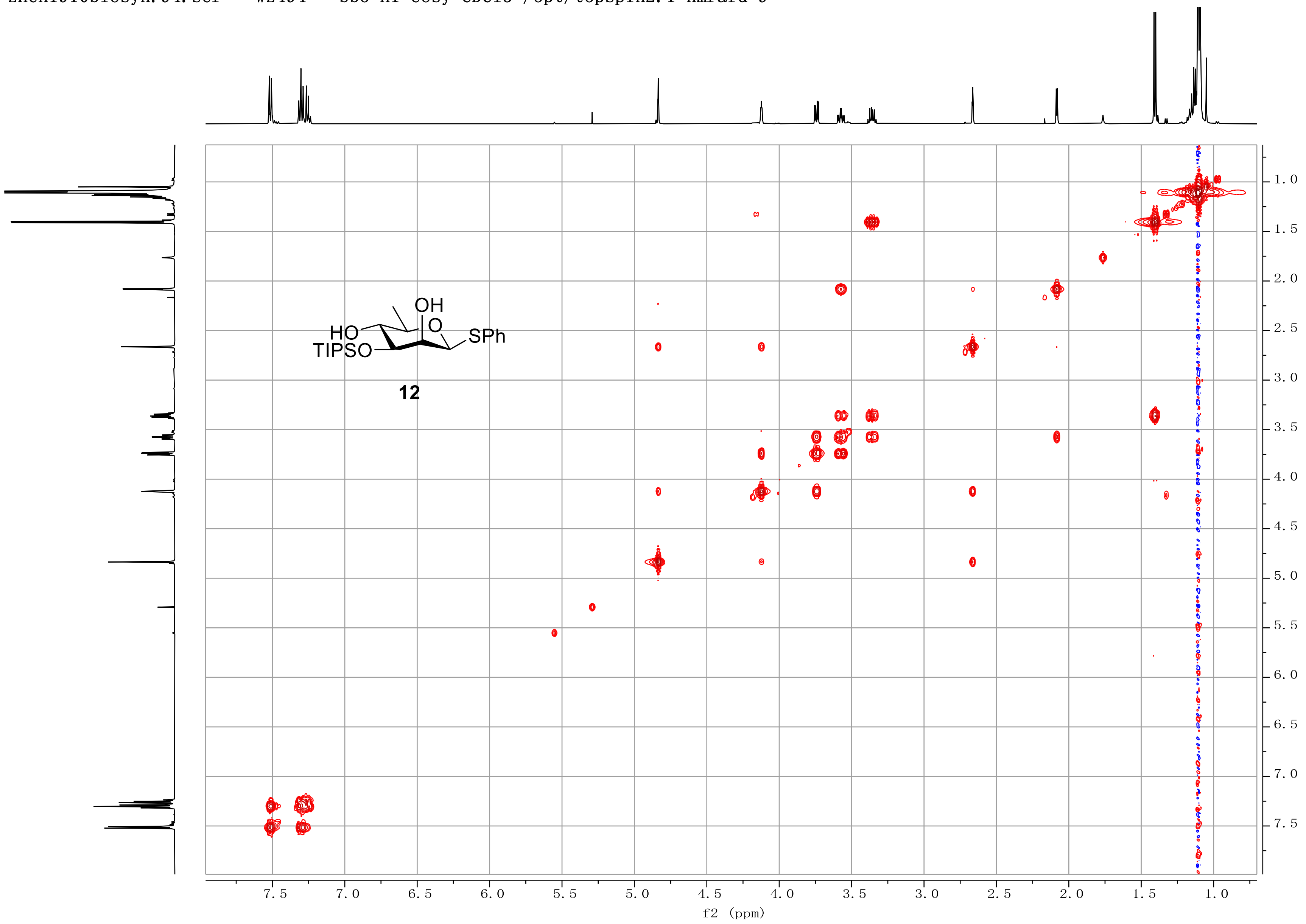

zhen1910biosyn.95.ser — wz494 — bbo-c13-HSQC CDCl3 /opt/topspin2.1 nmrafd 9

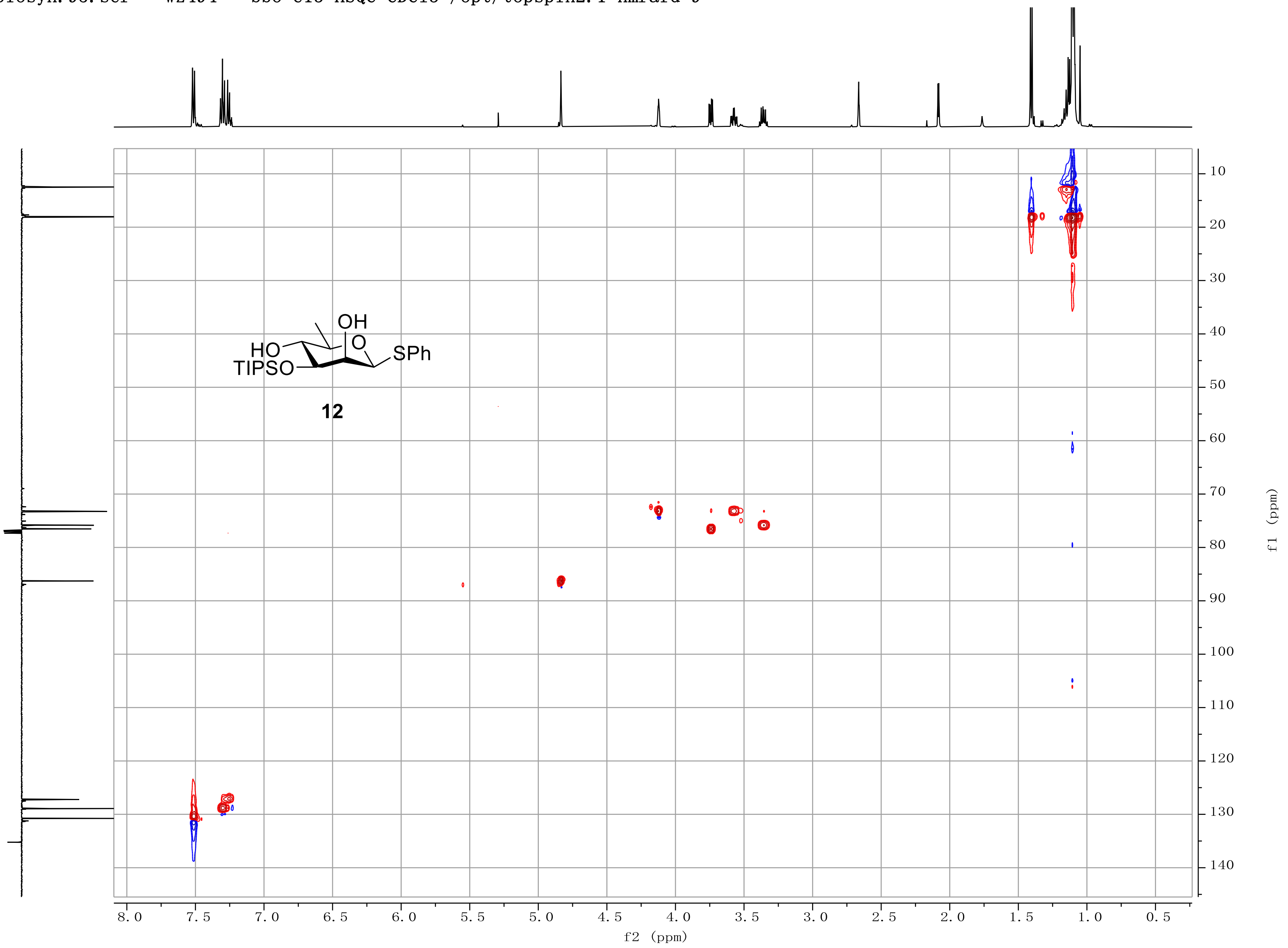

zhen1910biosyn.97.ser — wz494 — bbo-c13-HMBC CDCl3 /opt/topspin2.1 nmrafd 9

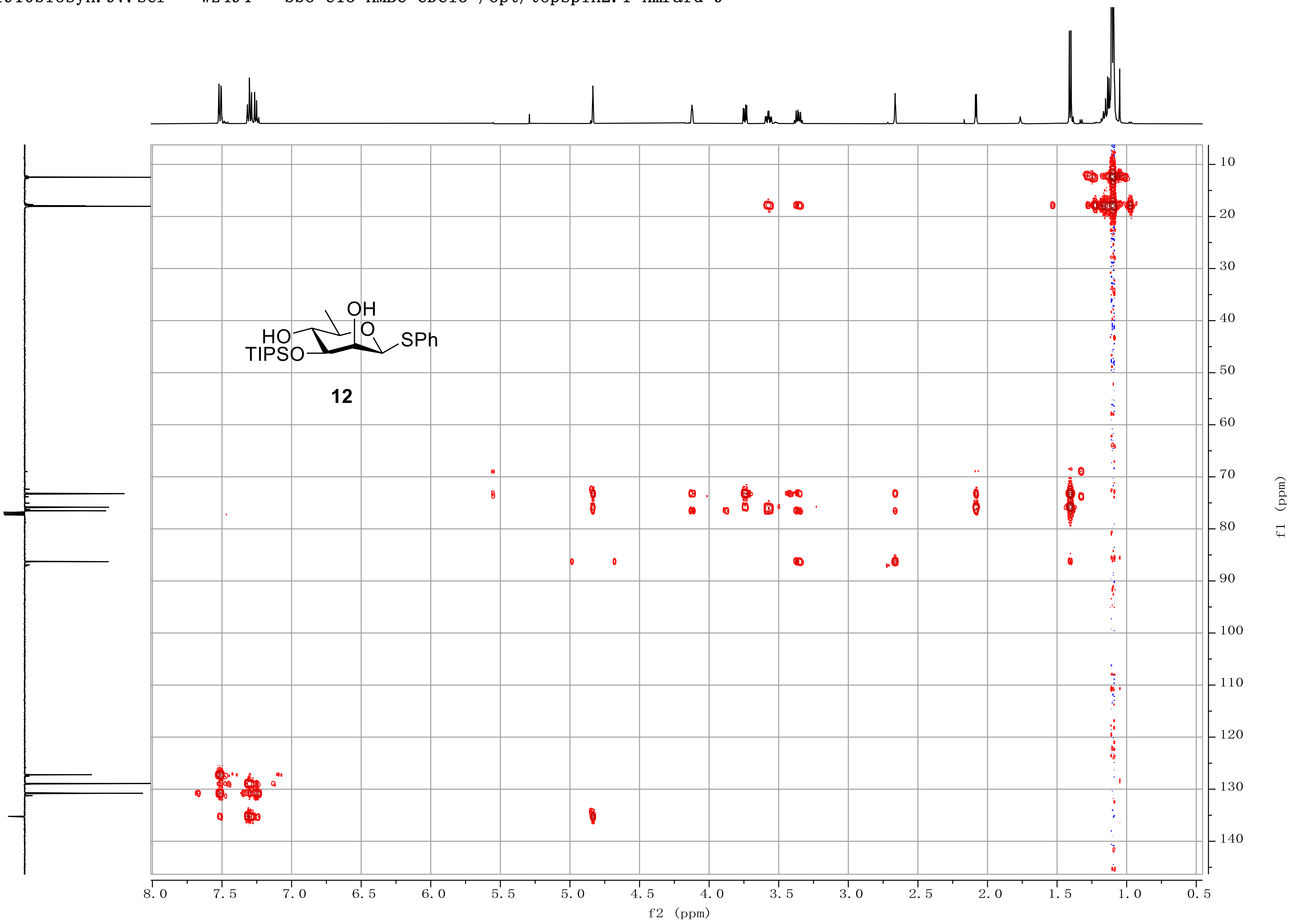

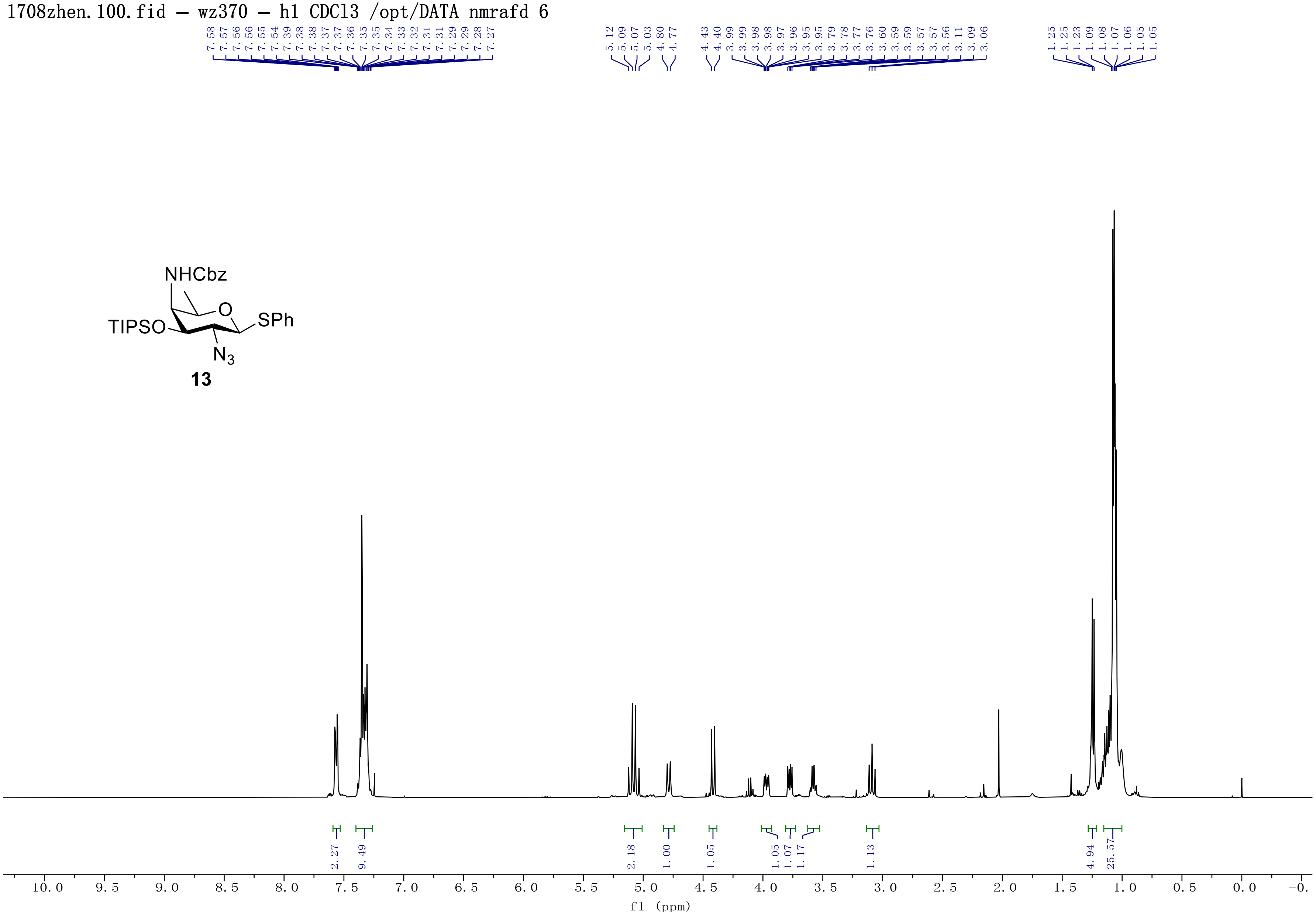
1708zhen.100.fid — wz370 — h1 CDCl3 /opt/DATA nmrafd 6
7.58 7.57 7.56 7.55 7.54 7.39 7.38 7.38 7.37 7.37 7.36 7.35 7.35 7.34 7.33 7.32 7.31 7.31 7.29 7.28 7.27
5.12 5.09 5.07 5.03 4.80 4.77
4.43 4.40 3.99 3.99 3.98 3.97 3.96 3.95 3.95 3.79 3.78 3.77 3.76 3.60 3.59 3.57 3.57 3.56 3.11 3.09 3.06
1.25 1.25 1.23 1.09 1.08 1.07 1.06 1.05 1.05
NHCbz
O
TIPSO
SPh
N3
13
2.27 9.49 2.18 1.00 1.05 1.05 1.07 1.17 1.13 4.94 25.57
10.0 9.5 9.0 8.5 8.0 7.5 7.0 6.5 6.0 5.5 5.0 4.5 4.0 3.5 3.0 2.5 2.0 1.5 1.0 0.5 0.0 -0.
f1 (ppm)

1708zhen.101.fid — wz370 — C13APT CDCl3 /opt/DATA nmrafd 6

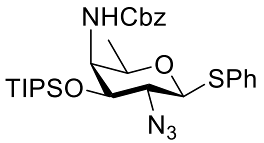

13

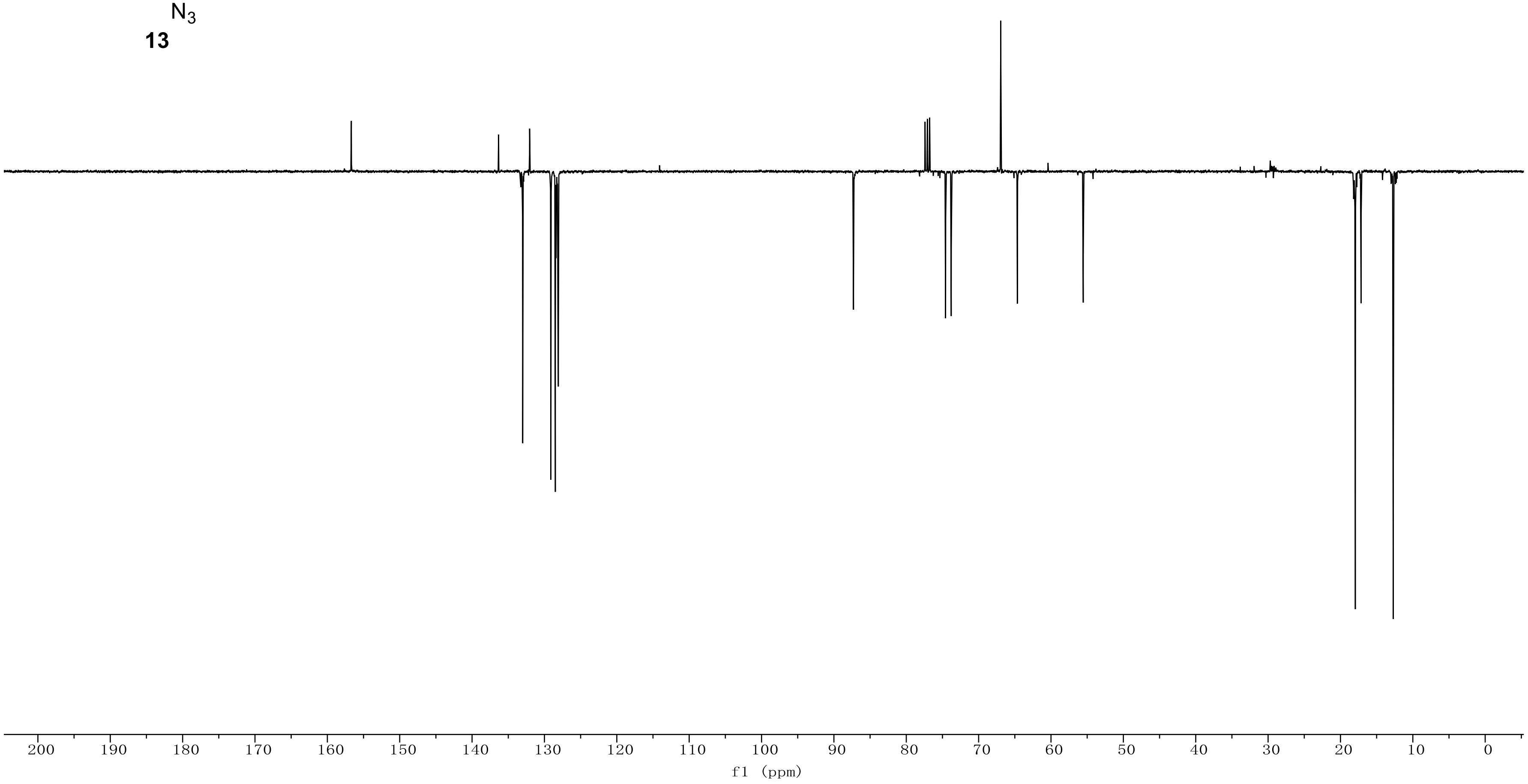

1708zhen.102.ser — wz370 — h1COSY CDCl3 /opt/DATA nmrafd 6

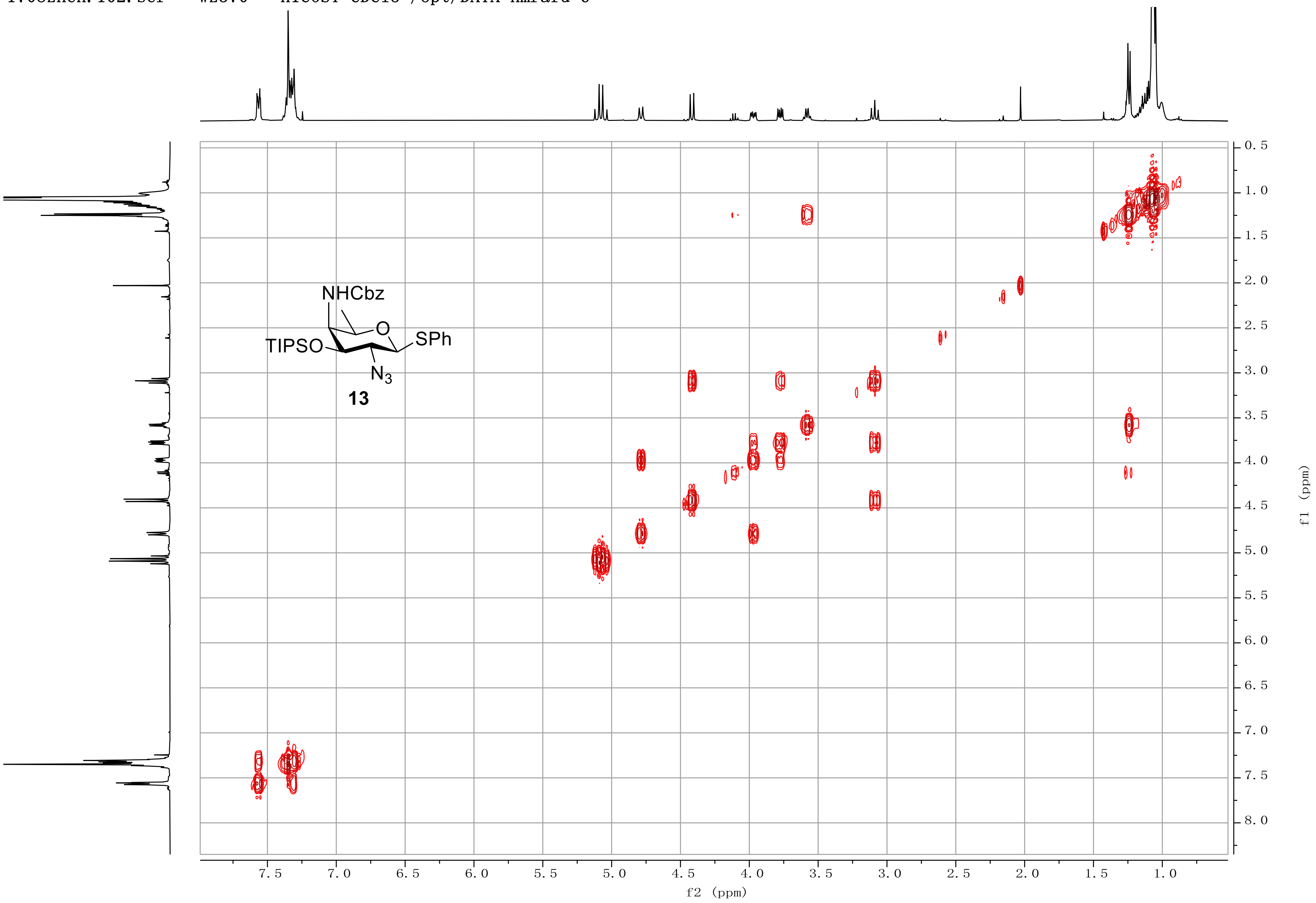

1708zhen.103.ser — wz370 — c13HSQC CDCl3 /opt/DATA nmrafd 6

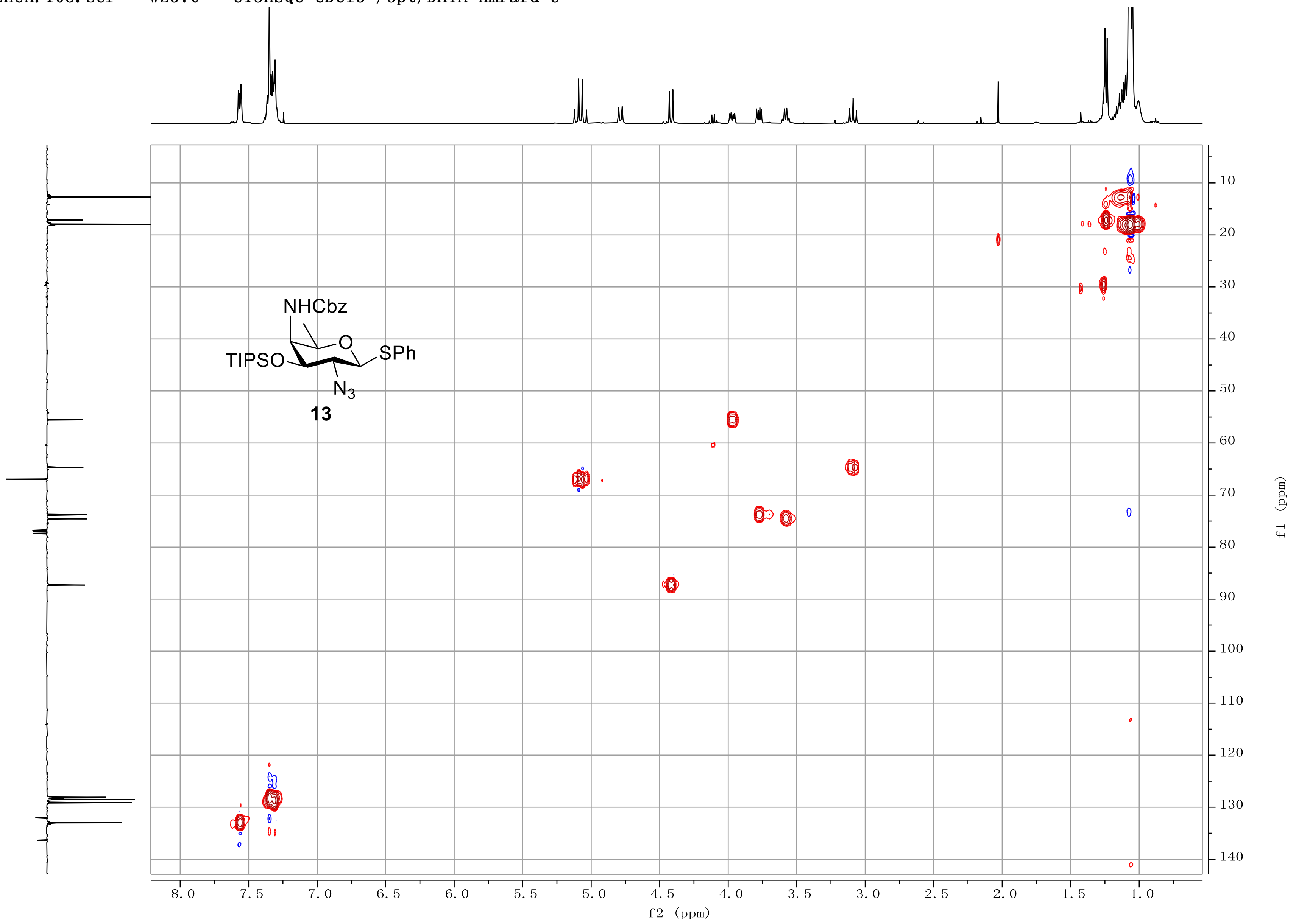

1708zhen.104.ser — wz370 — c13HMBC CDCl3 /opt/DATA nmrafd 6

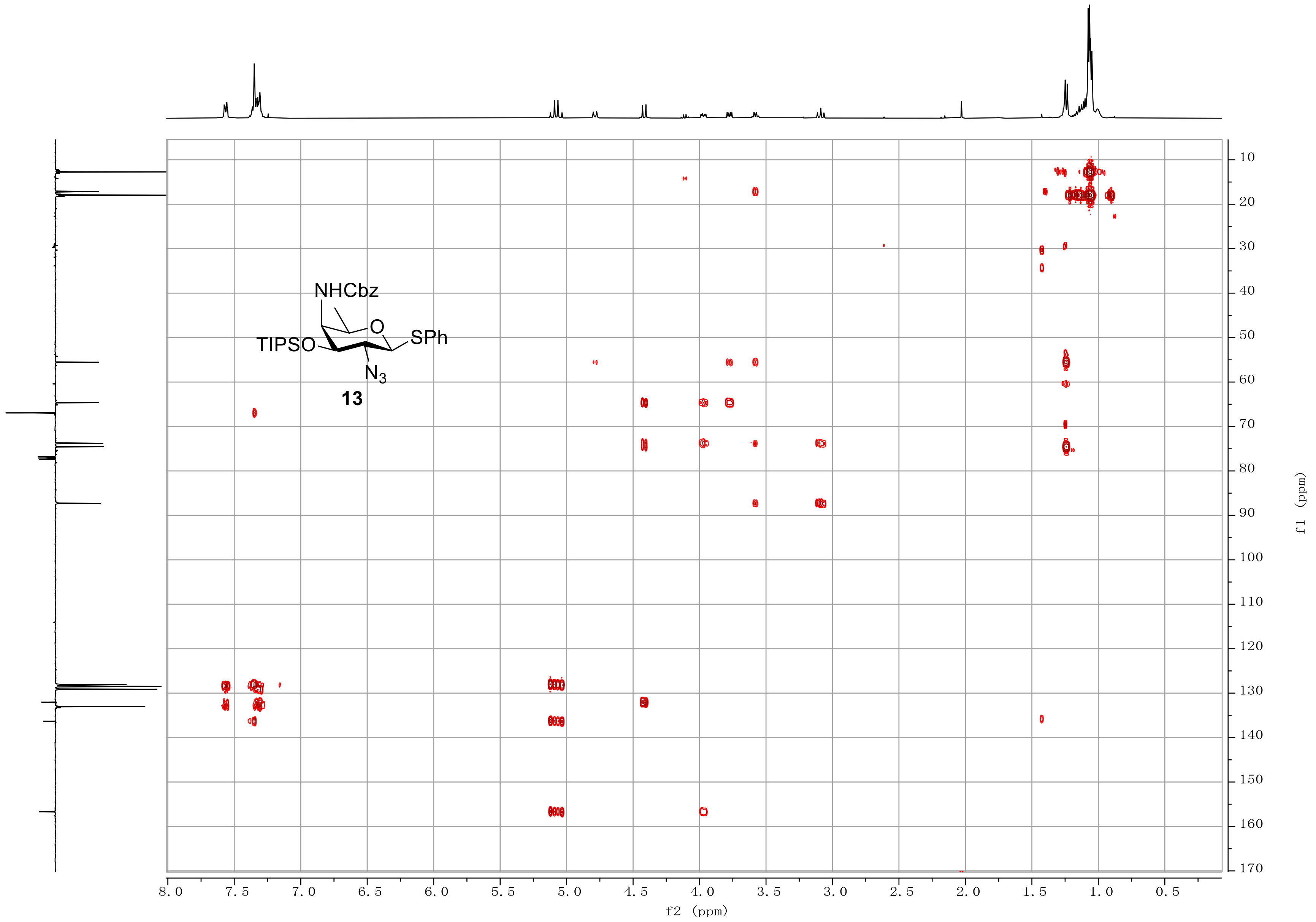

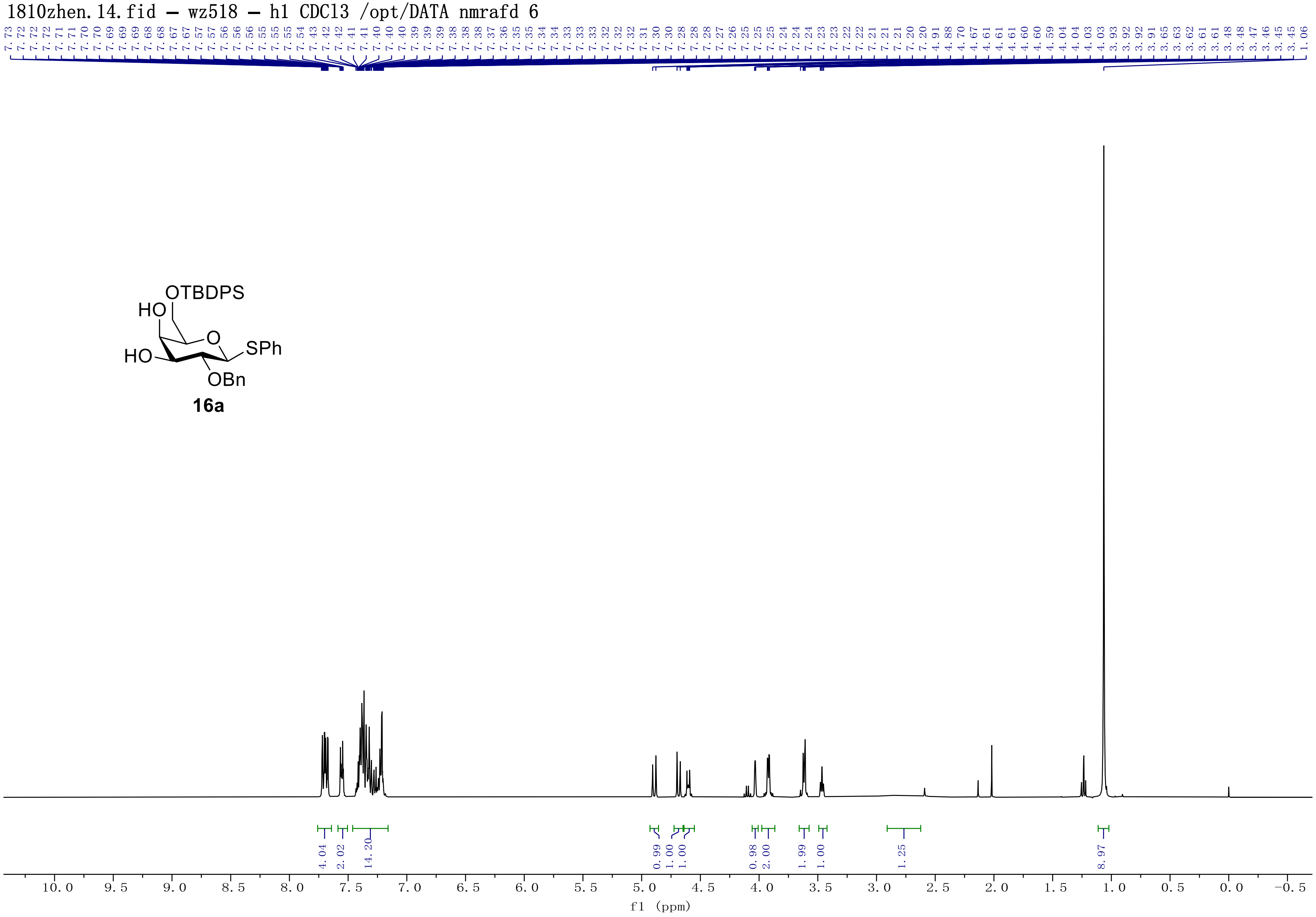
1810zhen.14.fid — wz518 — h1 CDCl3 /opt/DATA nmrafd 6
7.73 7.72 7.72 7.71 7.71 7.70 7.70 7.69 7.69 7.69 7.68 7.67 7.67 7.57 7.57 7.56 7.56 7.56 7.55 7.55 7.55 7.54 7.43 7.42 7.42 7.41 7.41 7.40 7.40 7.40 7.39 7.39 7.39 7.38 7.38 7.37 7.36 7.35 7.35 7.34 7.34 7.33 7.33 7.32 7.32 7.32 7.31 7.30 7.30 7.28 7.28 7.28 7.27 7.26 7.25 7.25 7.25 7.24 7.24 7.24 7.23 7.23 7.22 7.22 7.21 7.21 7.21 7.20 4.91 4.88 4.70 4.67 4.61 4.61 4.61 4.60 4.59 4.04 4.04 4.03 4.03 3.93 3.92 3.92 3.91 3.65 3.63 3.62 3.61 3.61 3.48 3.48 3.47 3.46 3.45 3.45 1.06
4.04 2.02 14.20 0.99 1.00 1.00 0.98 2.00 1.99 1.00 1.25 8.97
10.0 9.5 9.0 8.5 8.0 7.5 7.0 6.5 6.0 5.5 5.0 4.5 4.0 3.5 3.0 2.5 2.0 1.5 1.0 0.5 0.0 -0.5
f1 (ppm)
OTBDPS
HO
O
SPh
HO
OBn

16a

1810zhen.15.fid — wz518 — C13APT CDCl3 /opt/DATA nmrafd 6

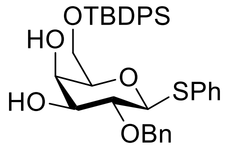

16a

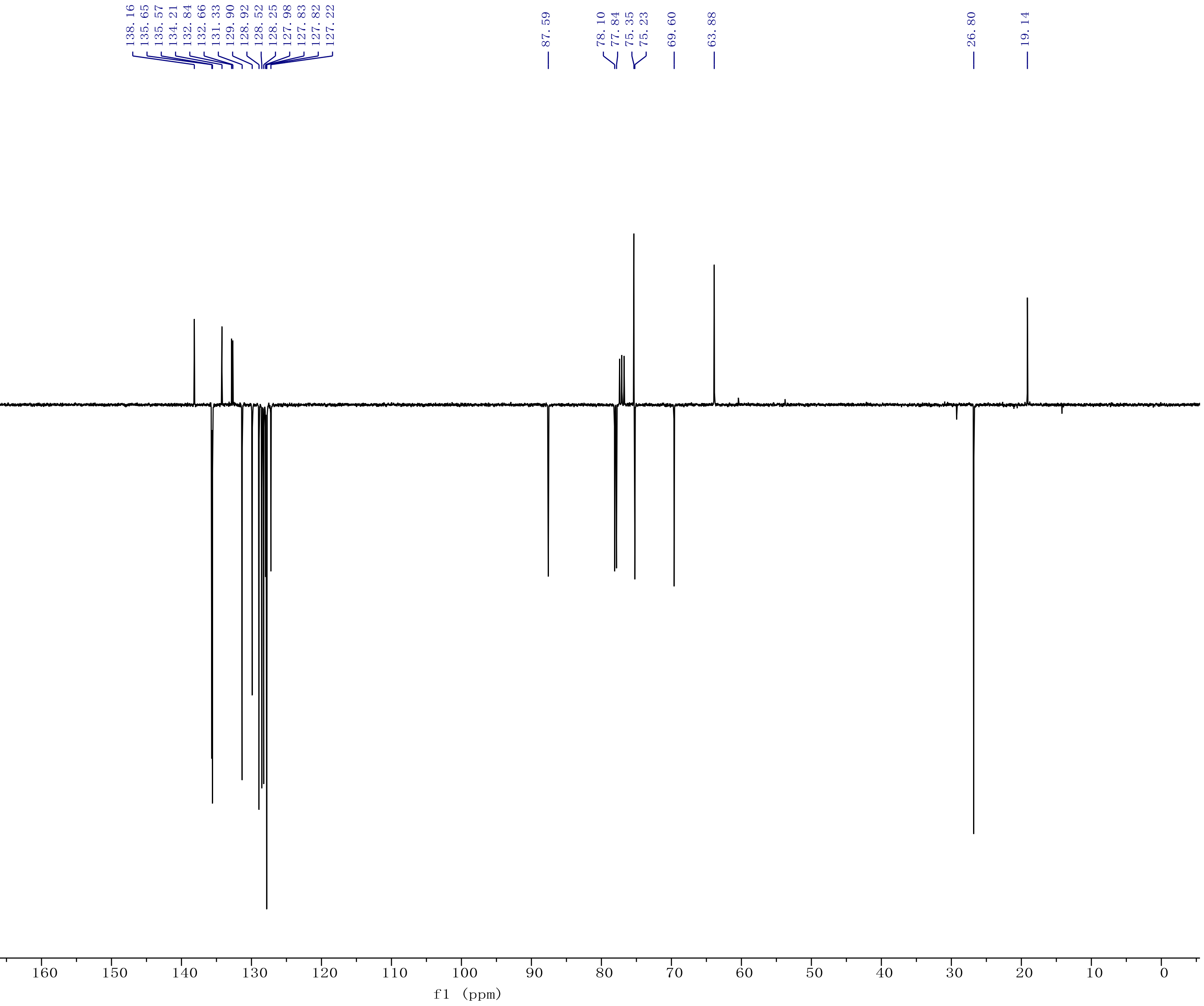

1810zhen.16.ser — wz518 — h1COSY CDCl3 /opt/DATA nmrafd 6

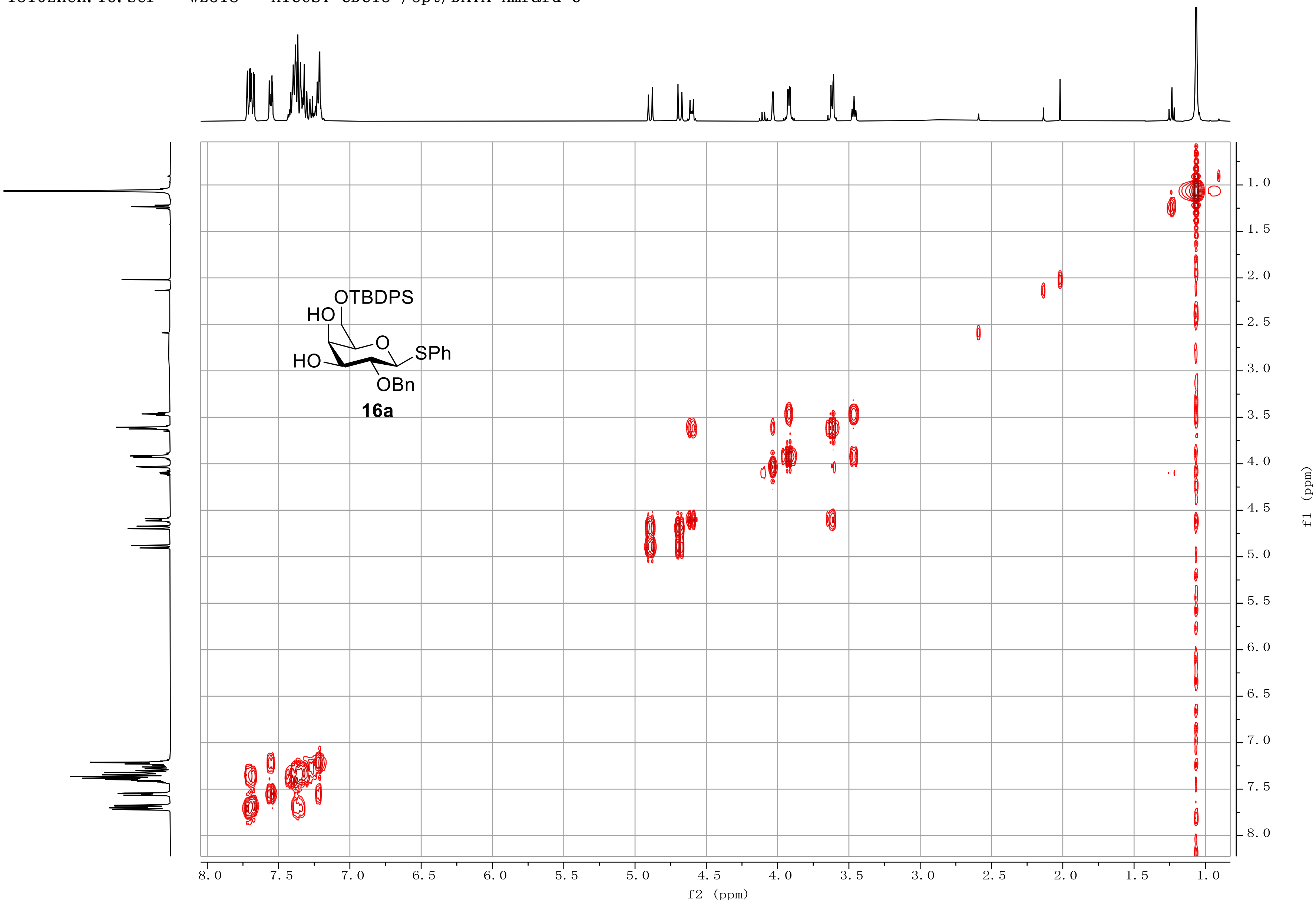

1810zhen.17.ser — wz518 — c13HSQC CDCl3 /opt/DATA nmrafd 6

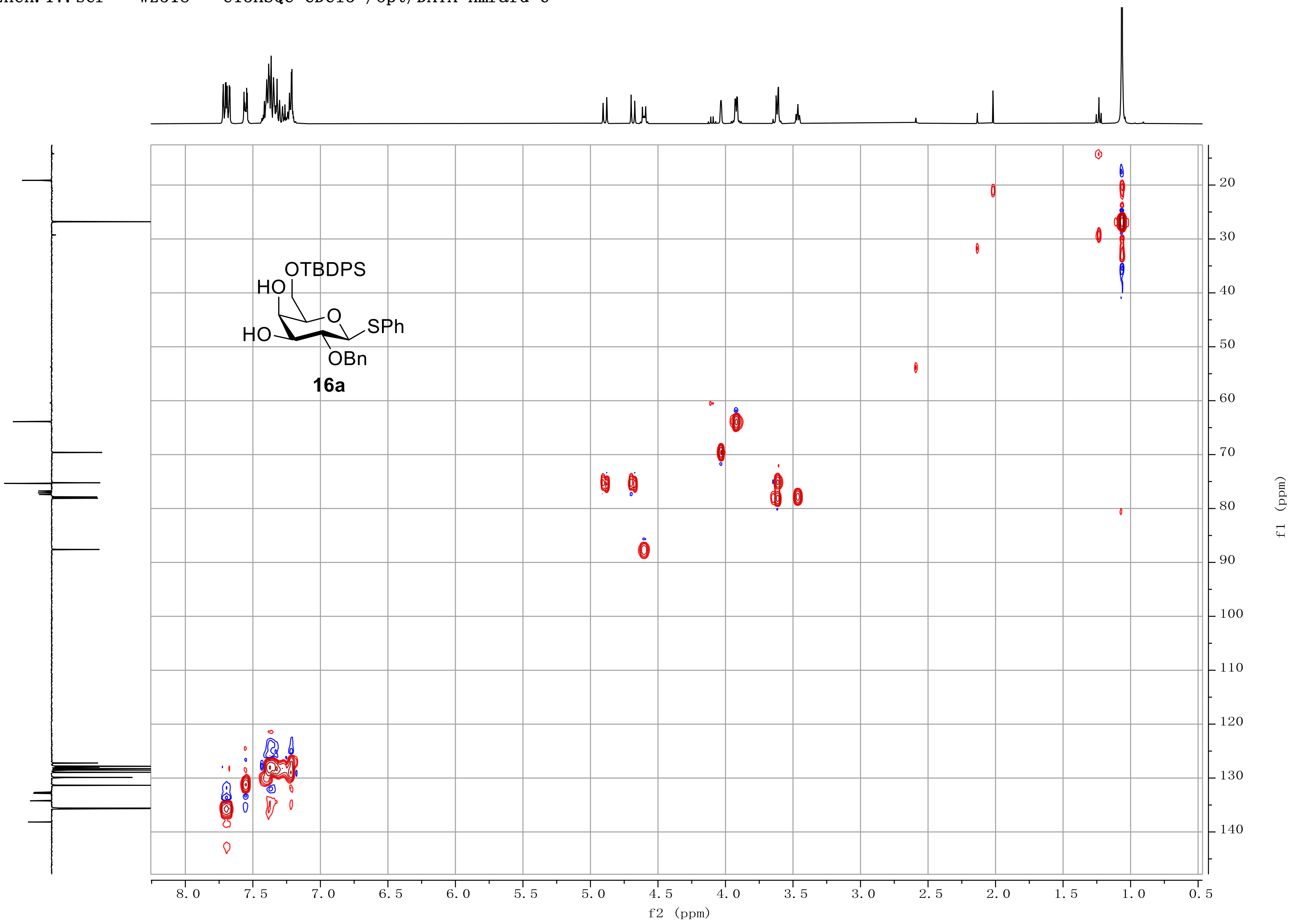

1810zhen.9.fid — wz519 — h1 CDCl3 /opt/DATA nmrafd 5

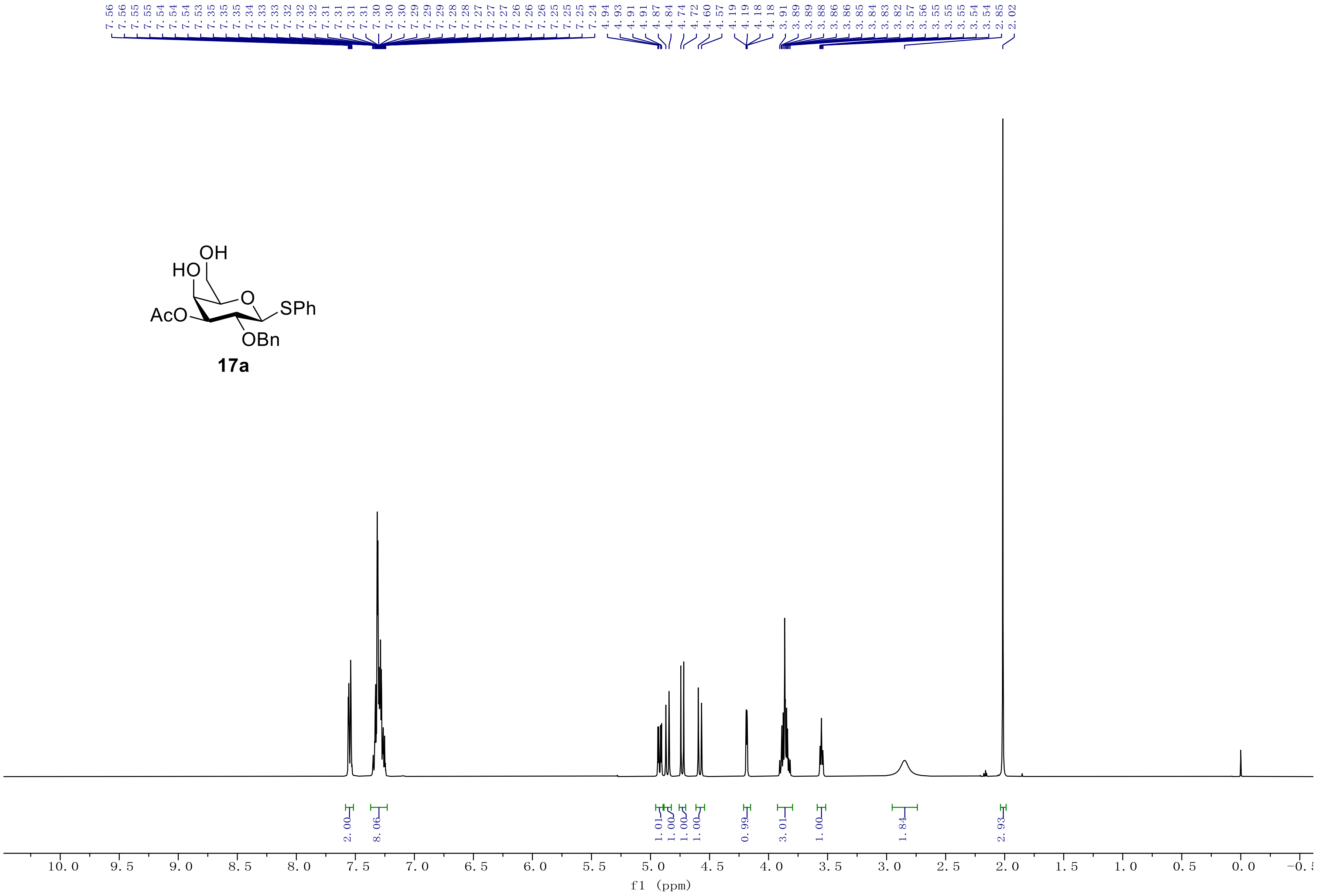

1810zhen.10.fid — wz519 — C13APT CDCl3 /opt/DATA nmrafd 5

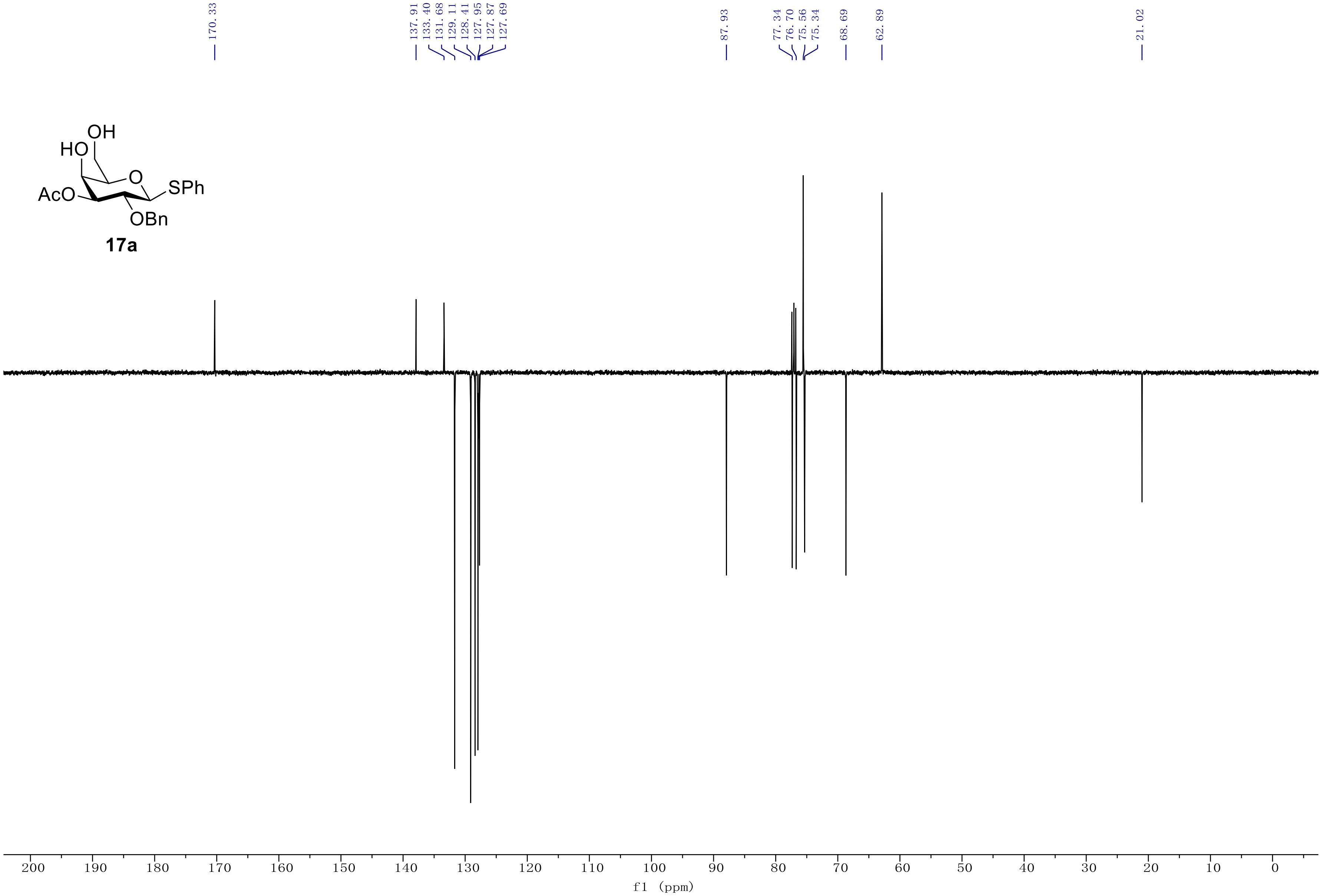

1810zhen.11.ser — wz519 — h1COSY CDCl3 /opt/DATA nmrafd 5

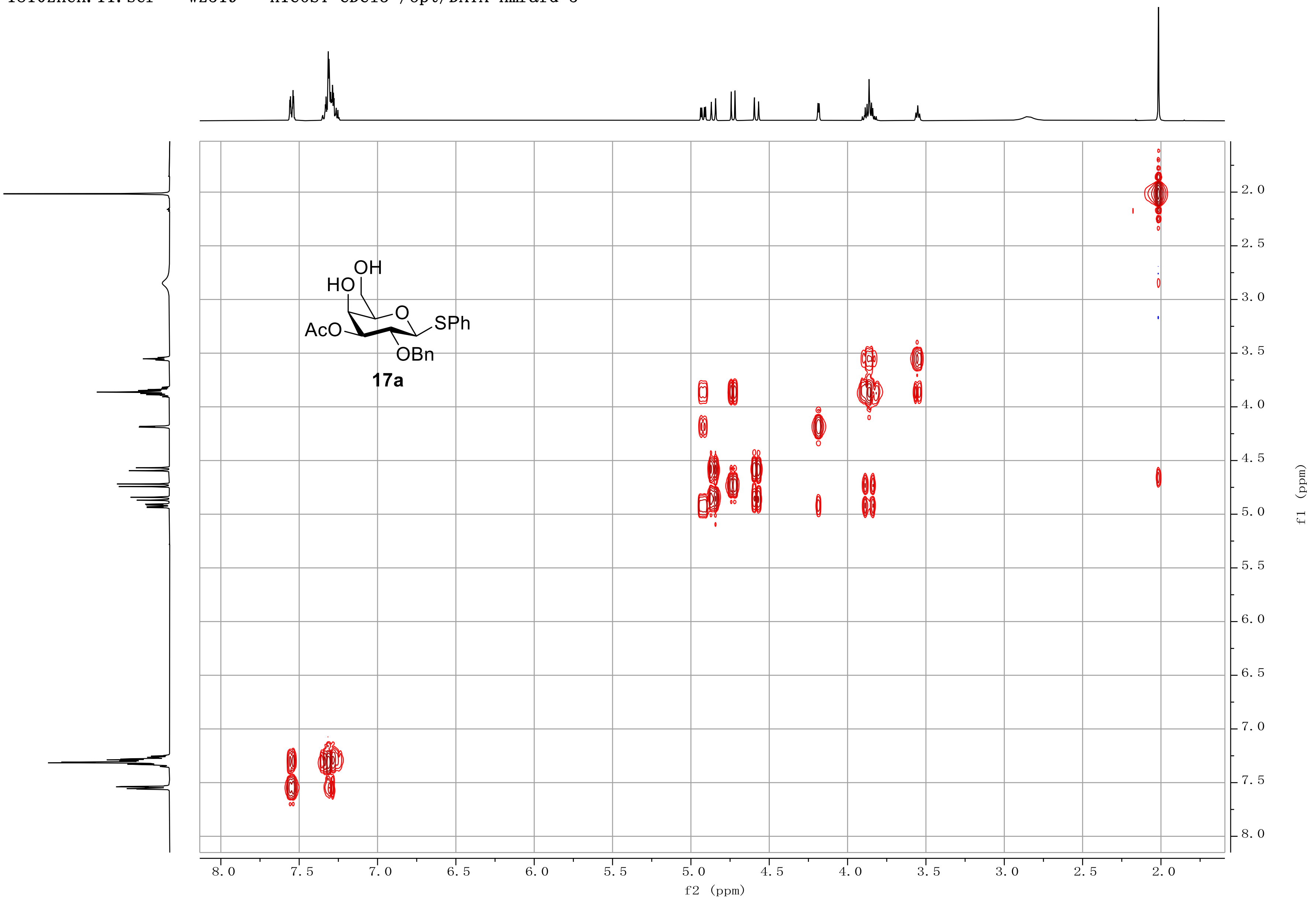

1810zhen.12.ser — wz519 — c13HSQC CDCl3 /opt/DATA nmrafd 5

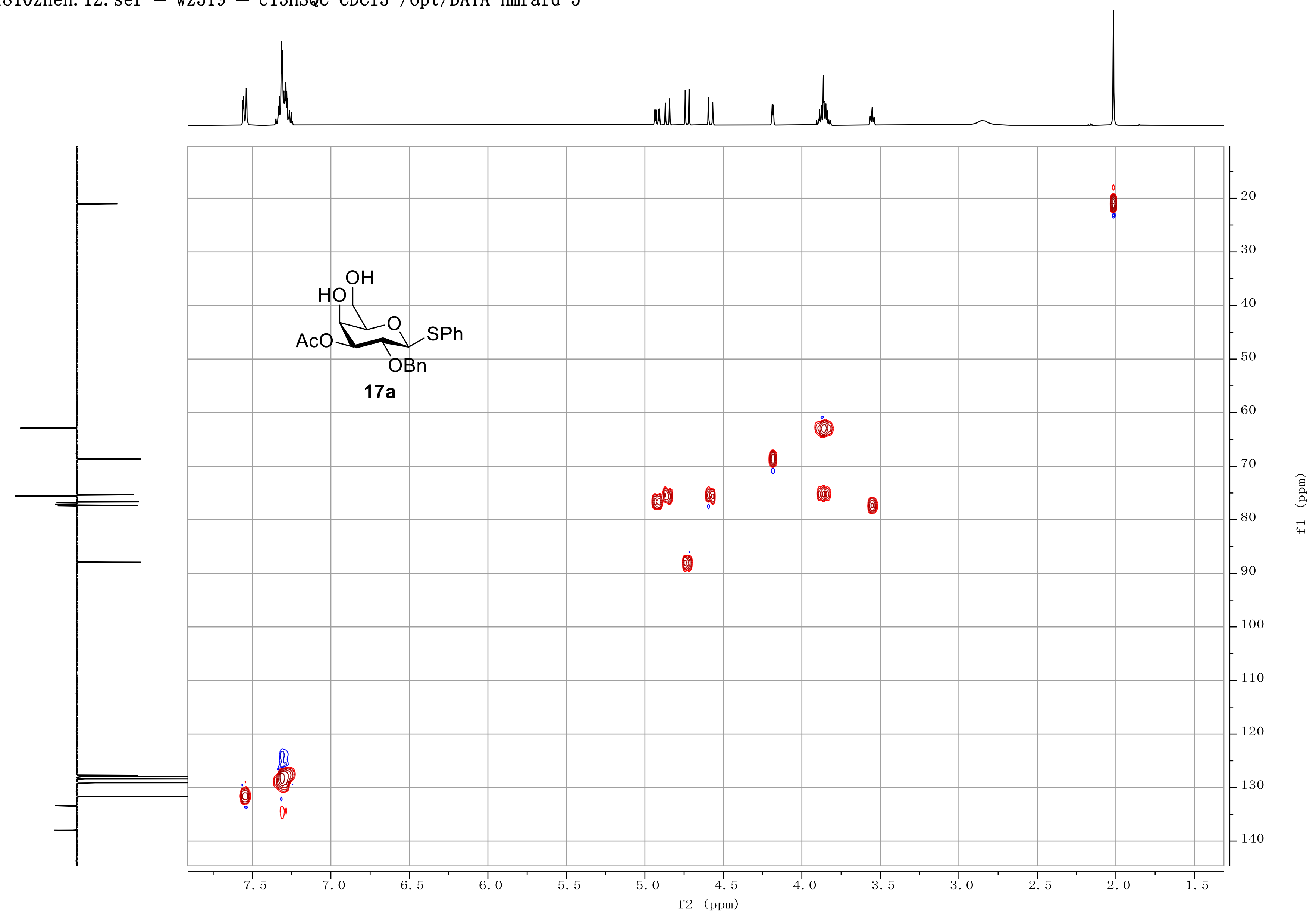

1810zhen.13.ser — wz519 — c13HMBC CDCl3 /opt/DATA nmrafd 5

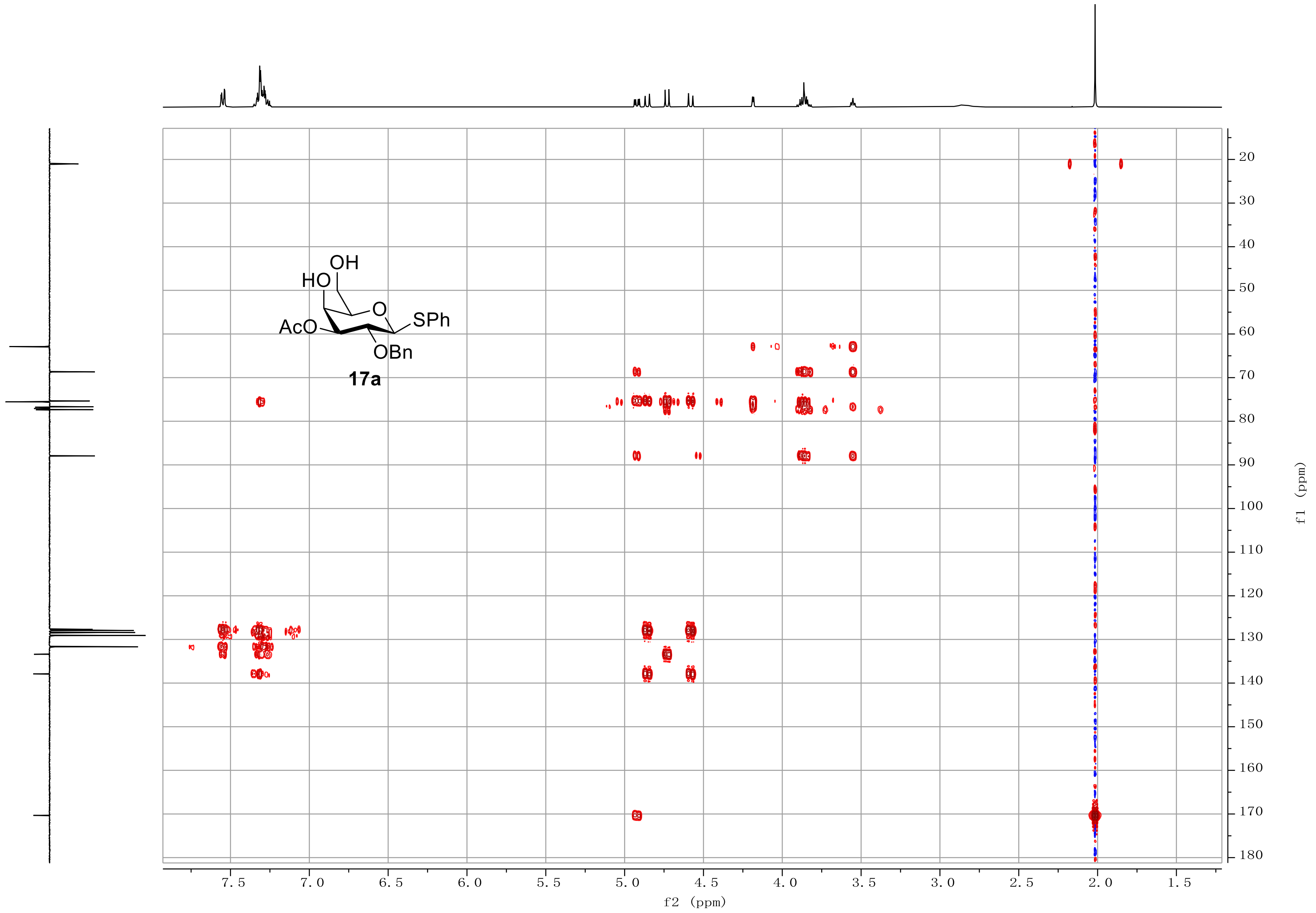

1507Qingju.41.fid — zqj-0474 — biosyn1Hfast CDCl3 /opt/DATA nmrafd 1

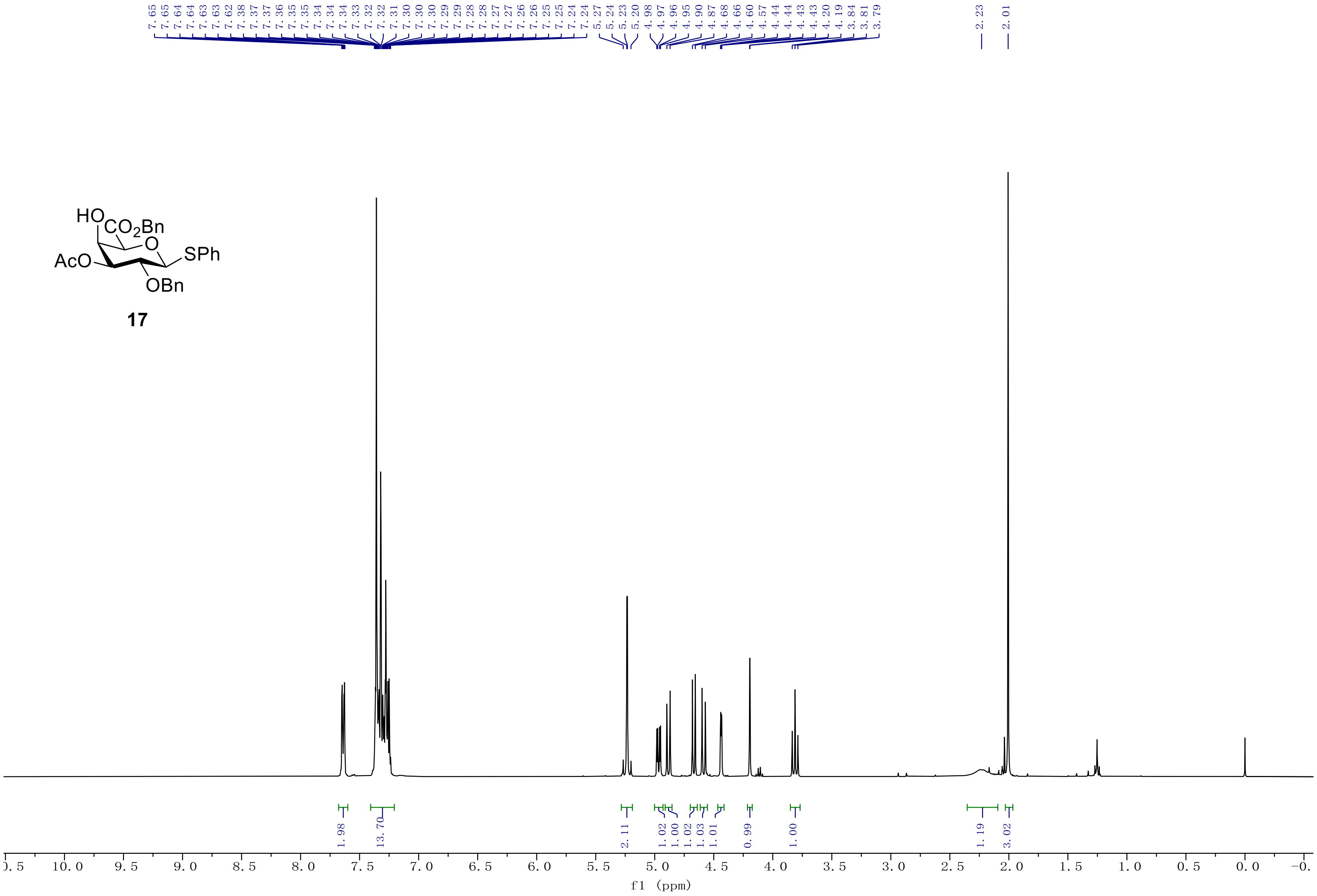

17

1507Qingju.42.fid — zqj-0474 — biosynAPTfast CDCl3 /opt/DATA nmrafd 1

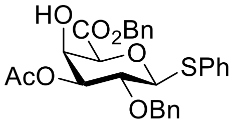

17

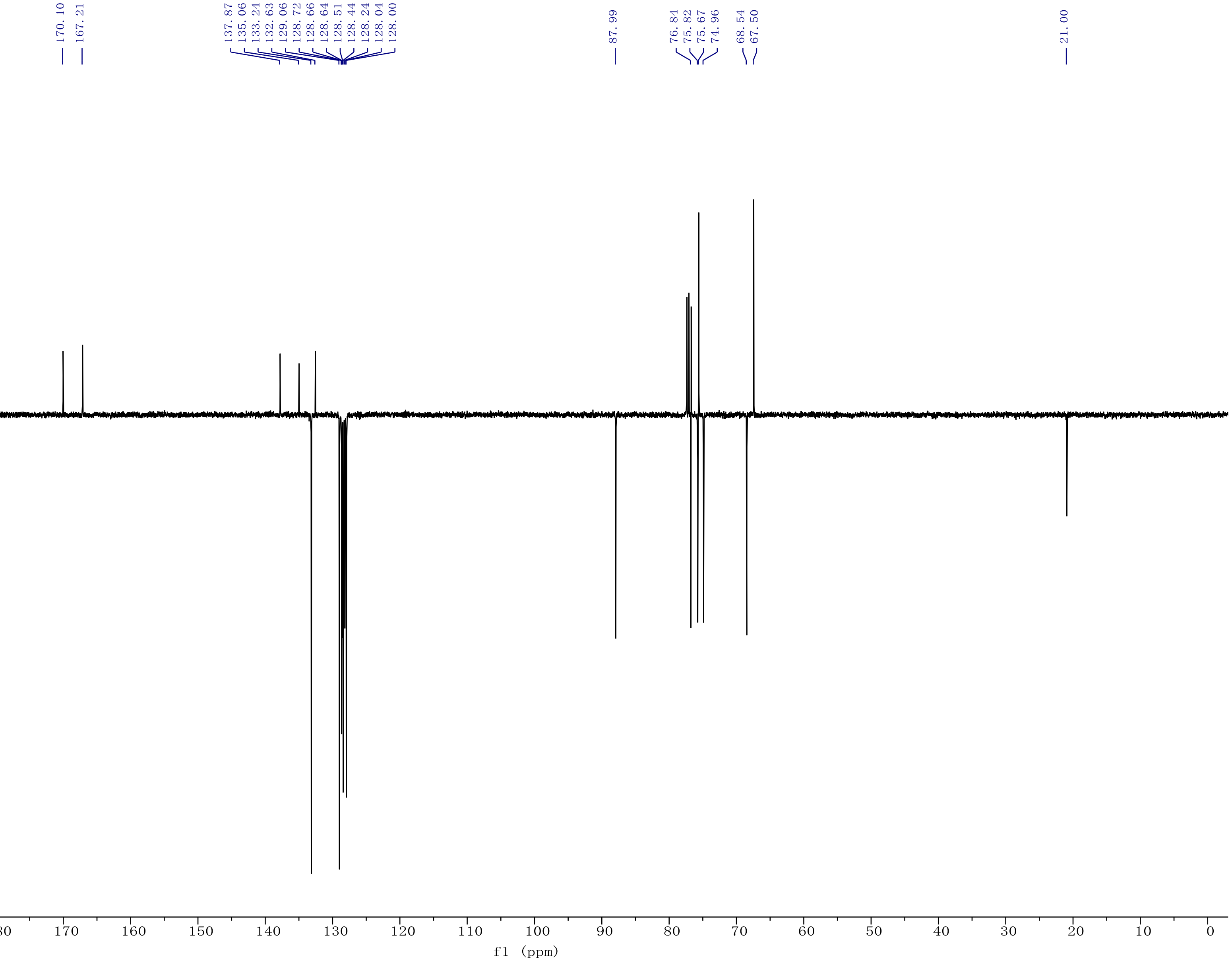

1507Qingju.43.ser — zqj-0474 — biosynCOSYfast CDCl3 /opt/DATA nmrafd 1

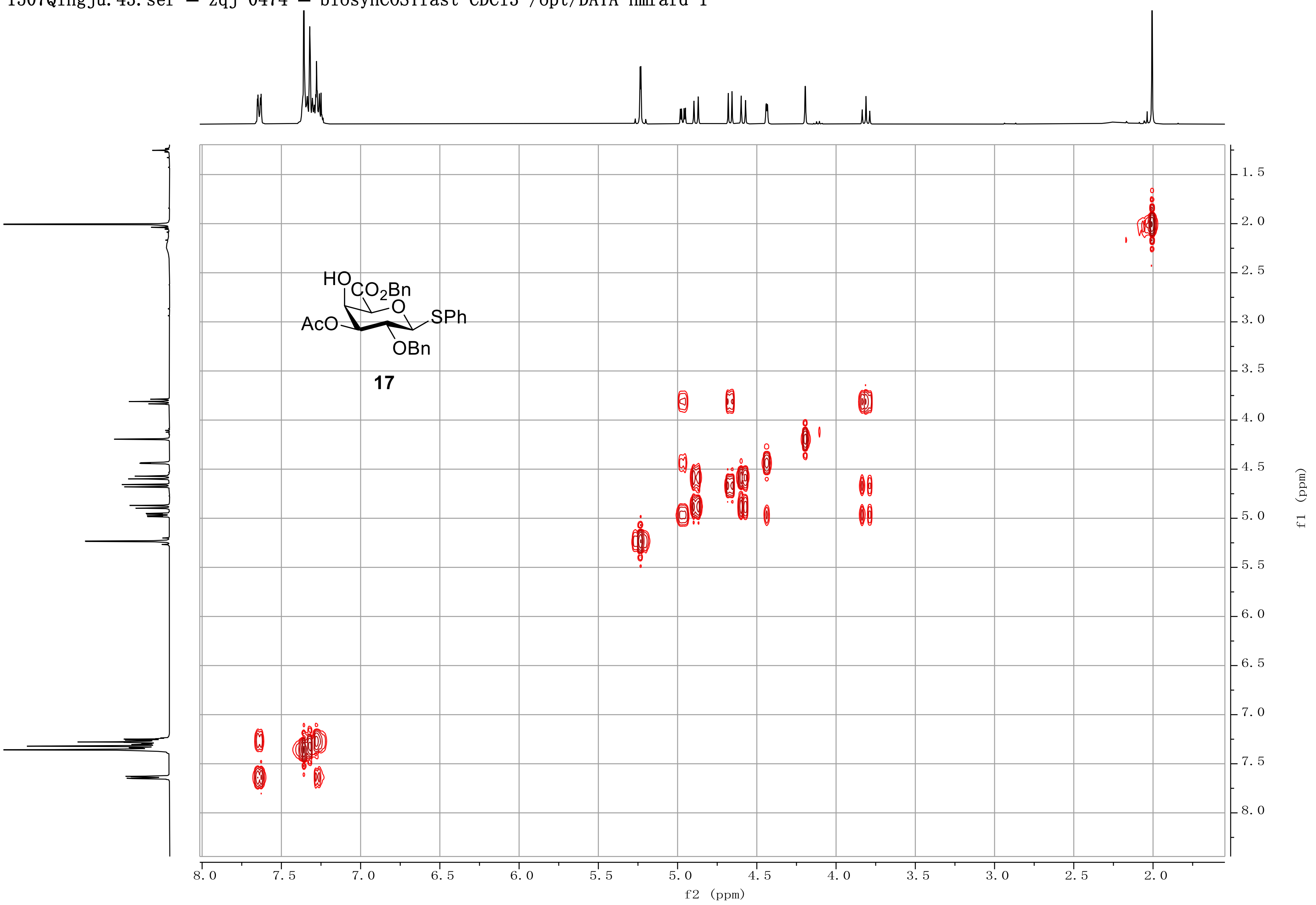

1507Qingju.44.ser — zqj-0474 — biosynHSQCfast CDCl3 /opt/DATA nmrafd 1

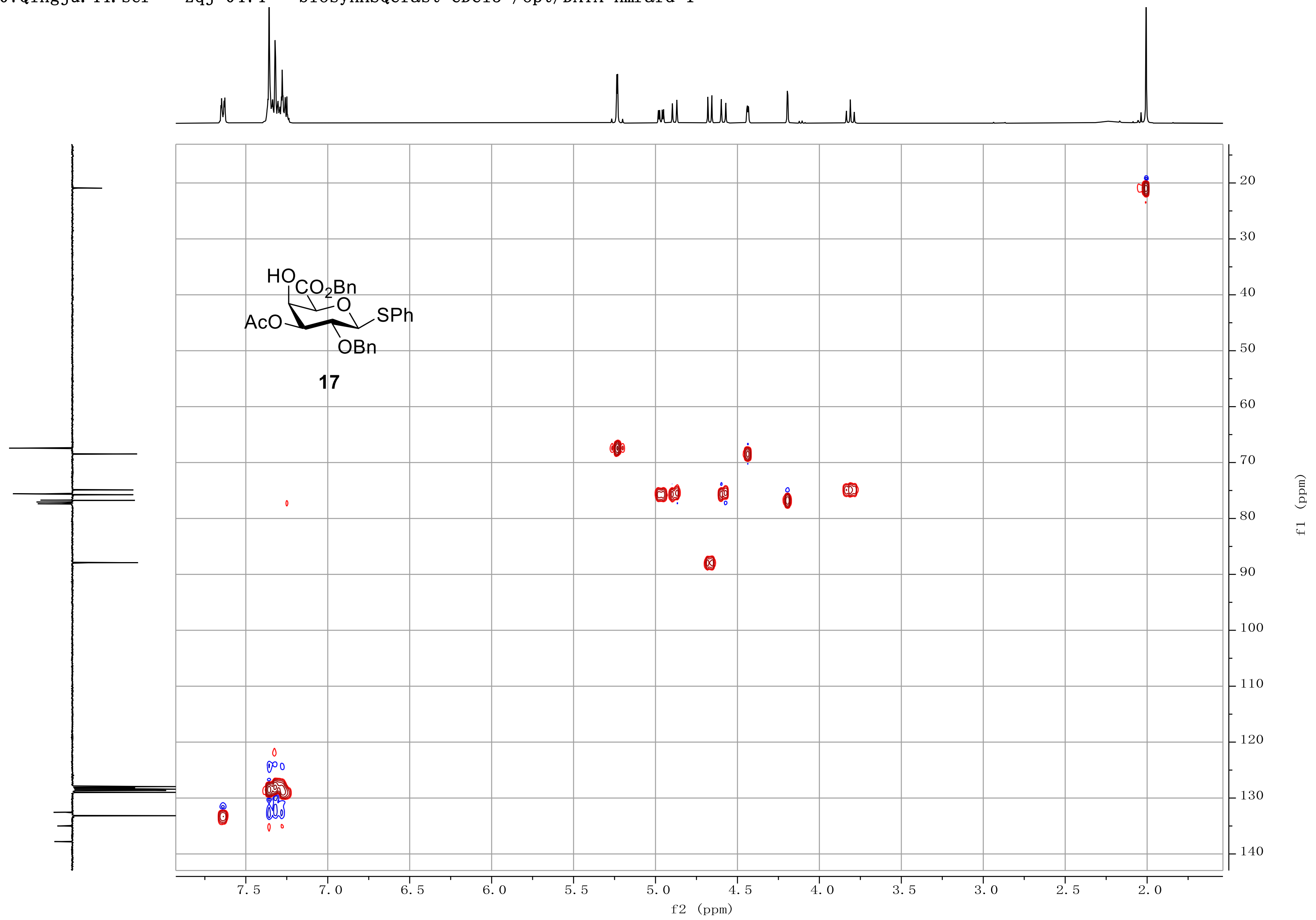

1507Qingju.45.fid — zqj-0476 — biosyn1Hfast CDCl3 /opt/DATA nmrafd 5

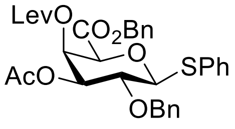

18

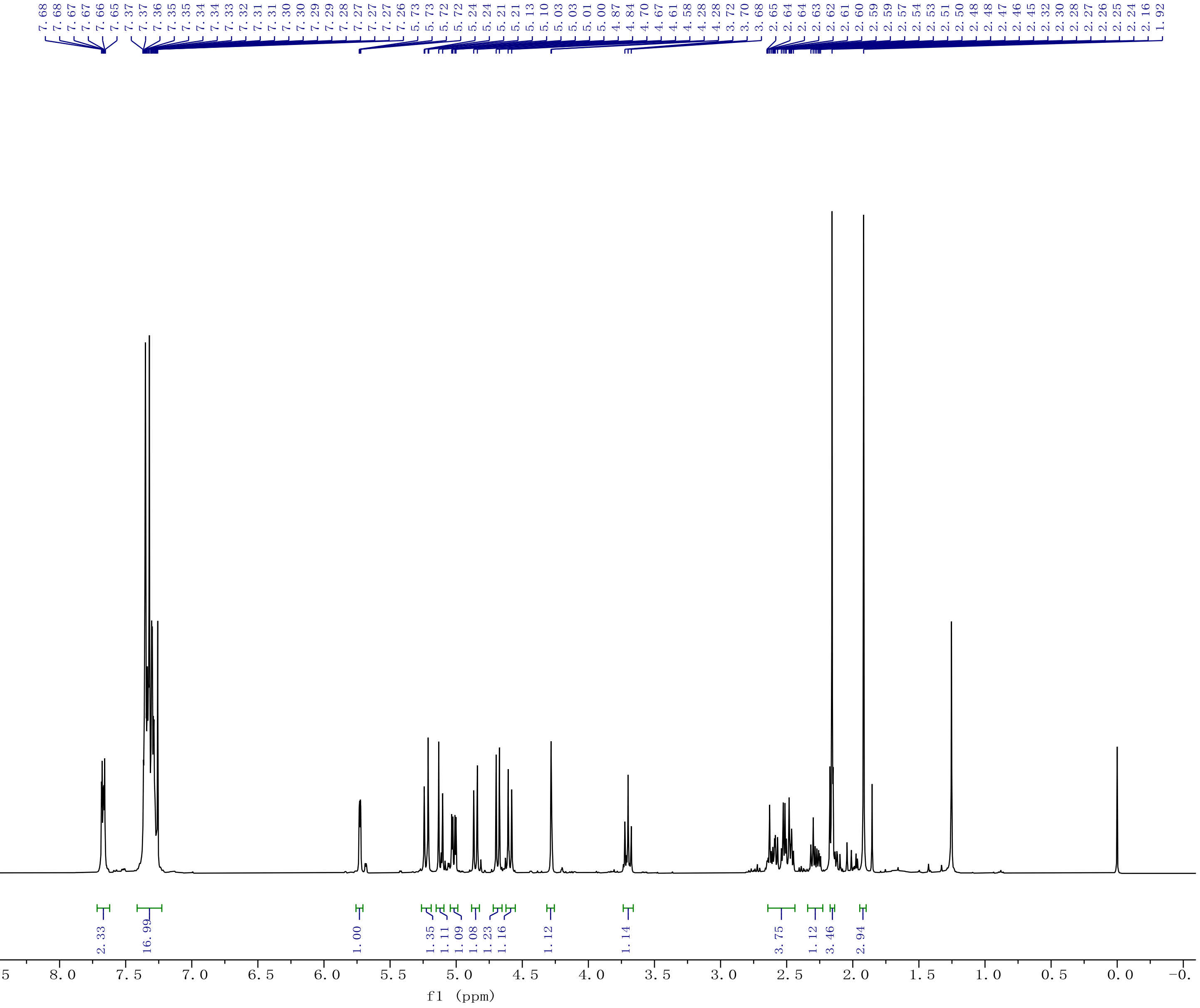

1507Qingju.46.fid — zqj-0476 — biosynAPTfast CDCl3 /opt/DATA nmrafd 5

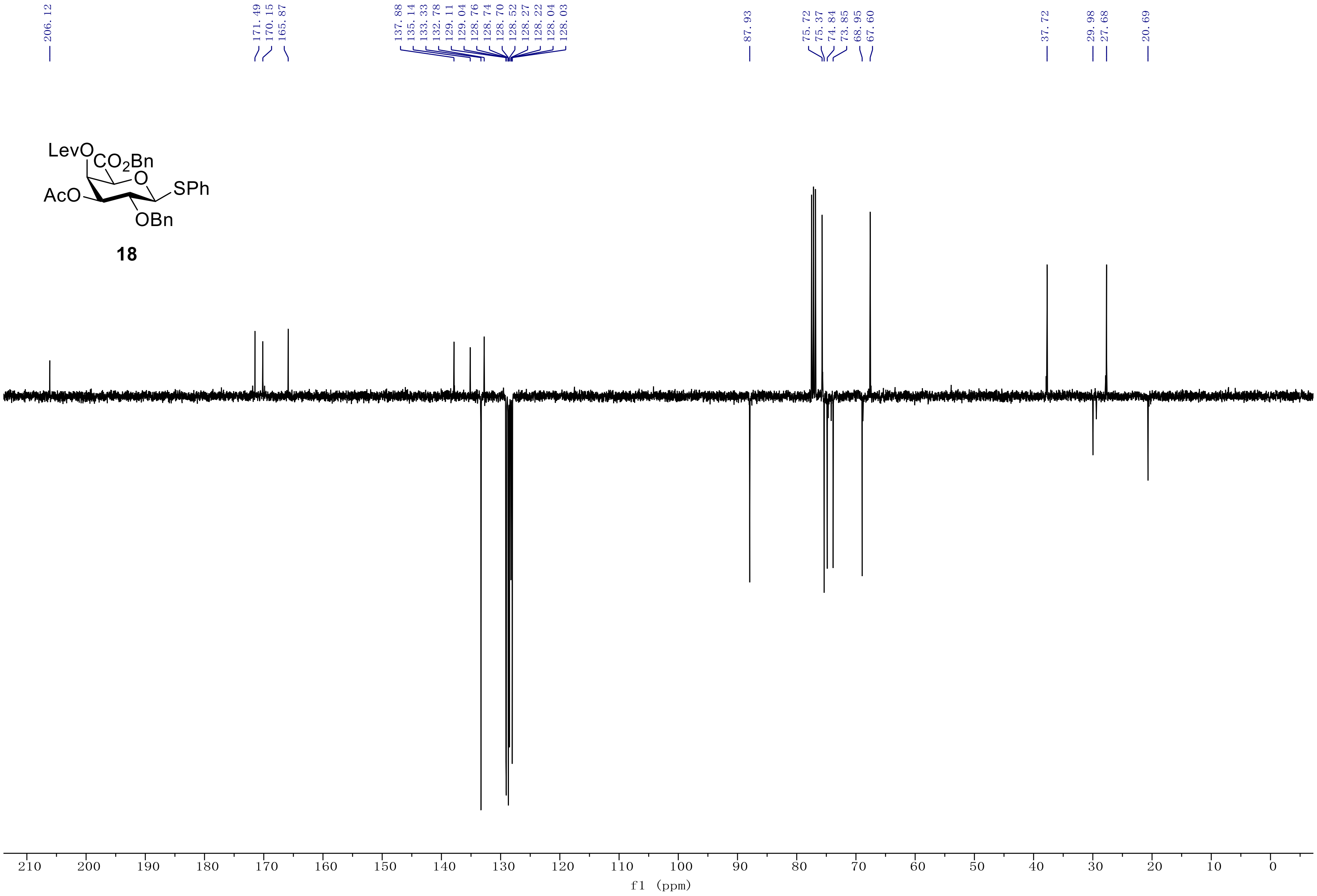

1507Qingju.47.ser — zqj-0476 — biosynCOSYfast CDCl3 /opt/DATA nmrafd 5

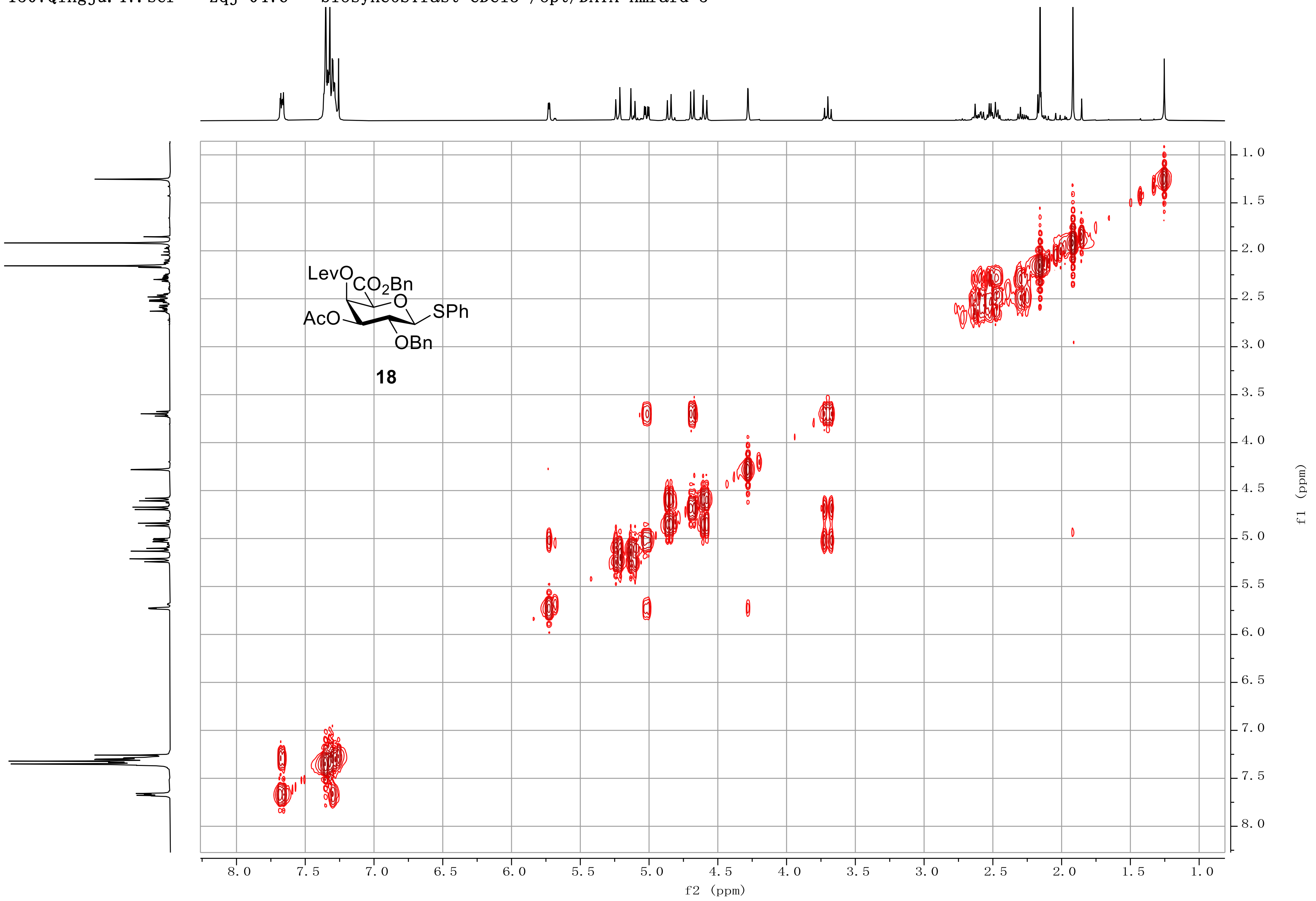

1507Qingju.48.ser — zqj-0476 — biosynHSQCfast CDCl3 /opt/DATA nmrafd 5

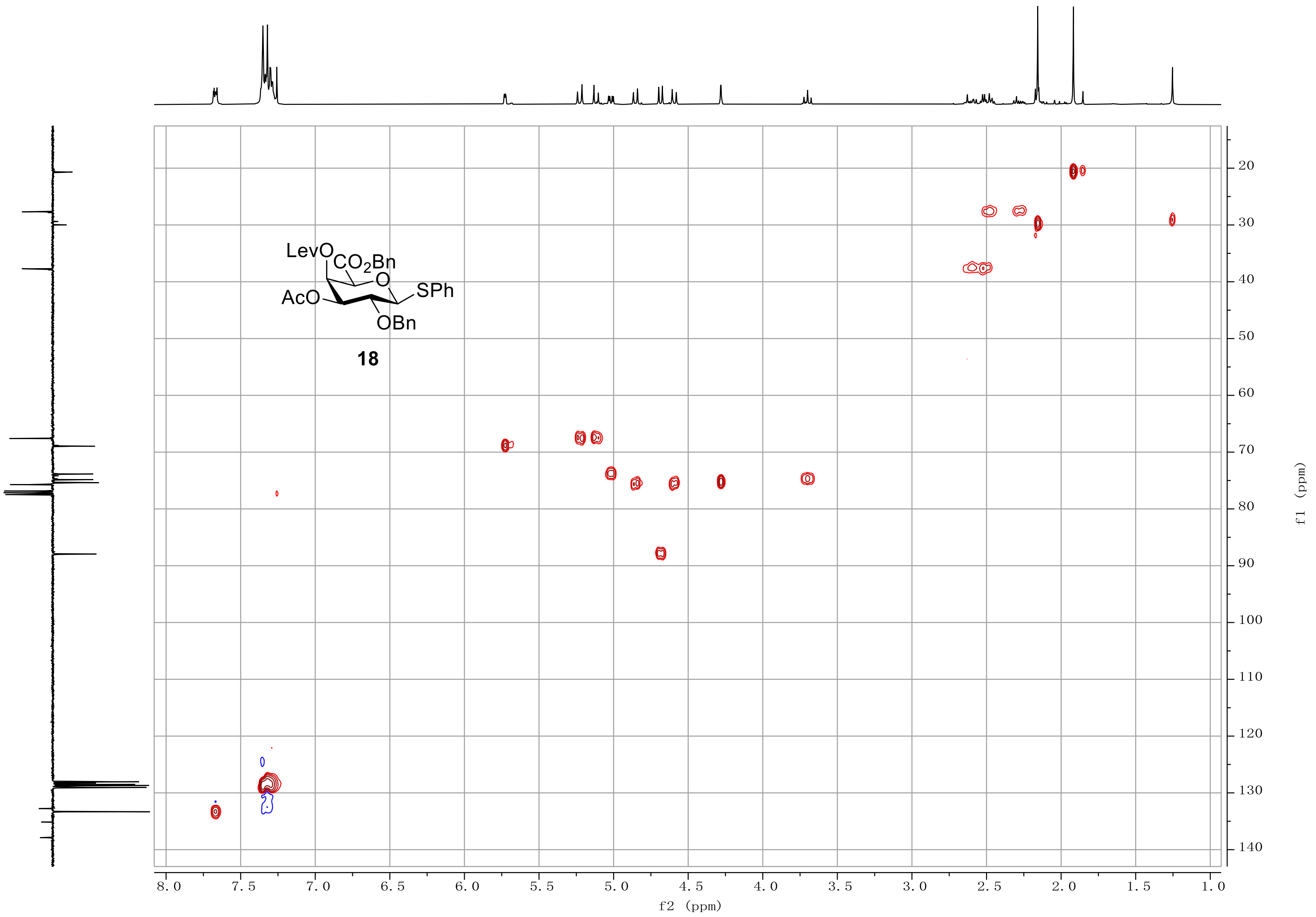

2001zhen.5.fid — wz464, size — h1 CDCl3 /opt/DATA nmrafd 14

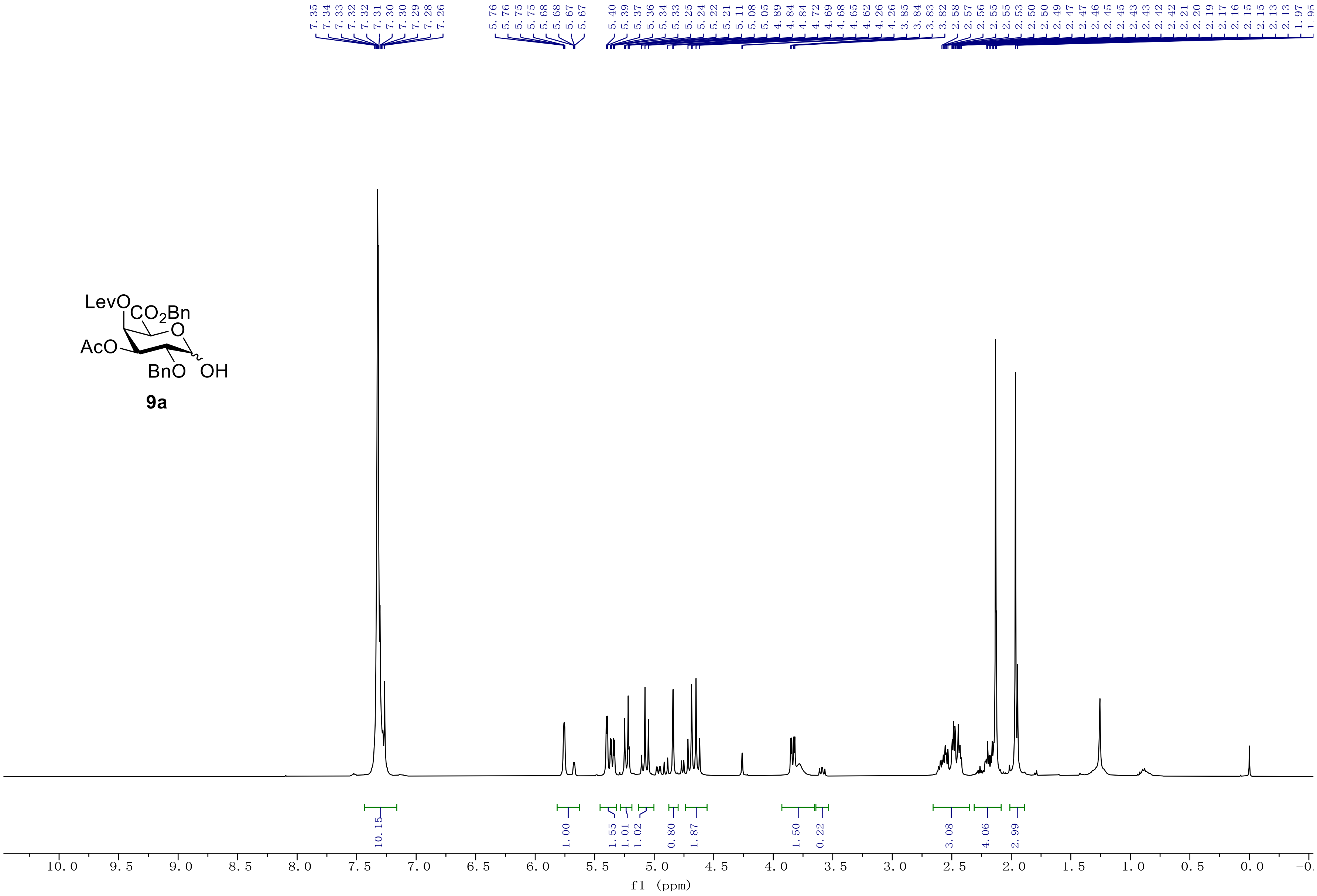

2001zhen.6.fid — wz464, size — C13APT CDCl3 /opt/DATA nmrafd 14

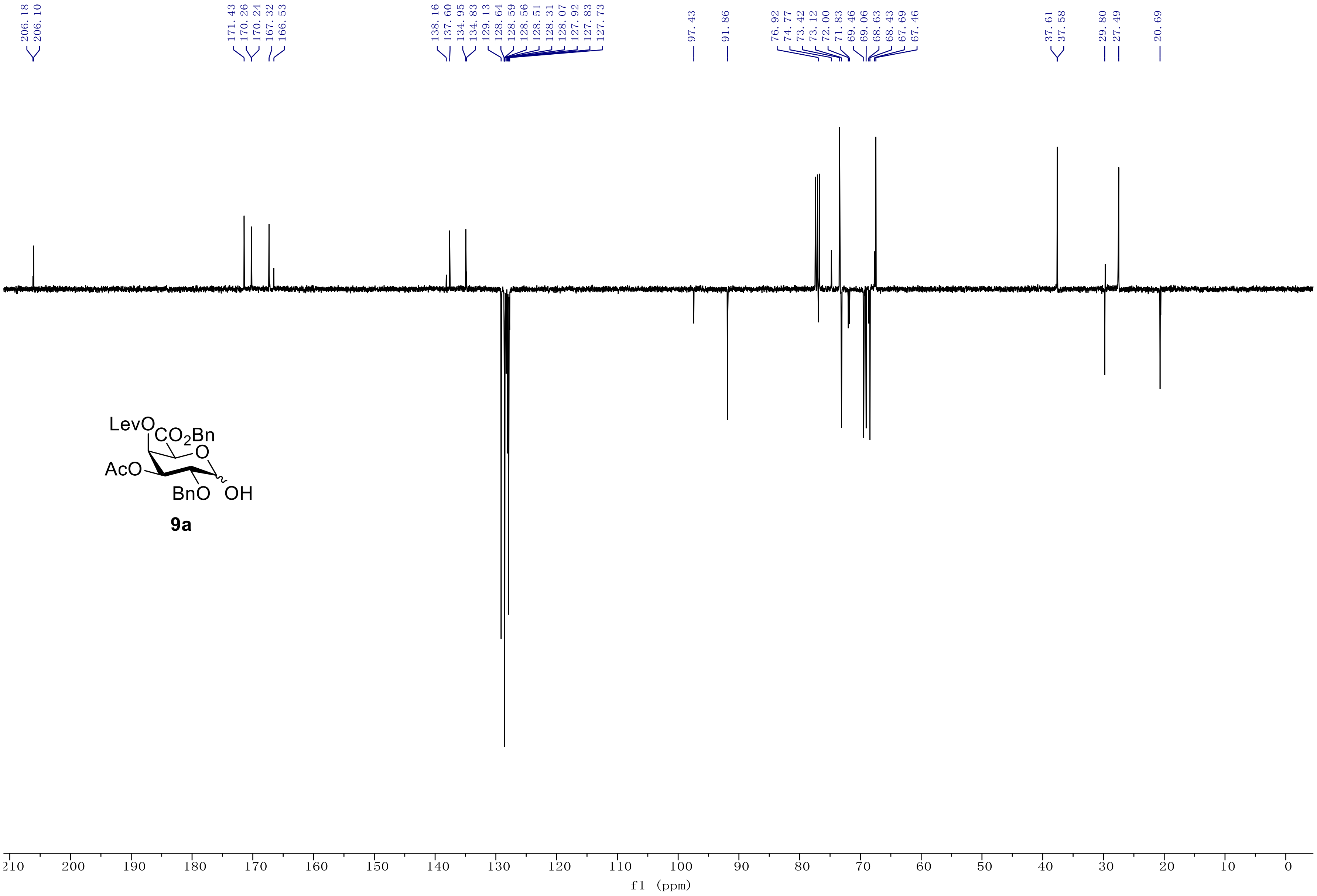

9a

2001zhen.7.ser — wz464, size — h1COSY CDCl3 /opt/DATA nmrafd 14

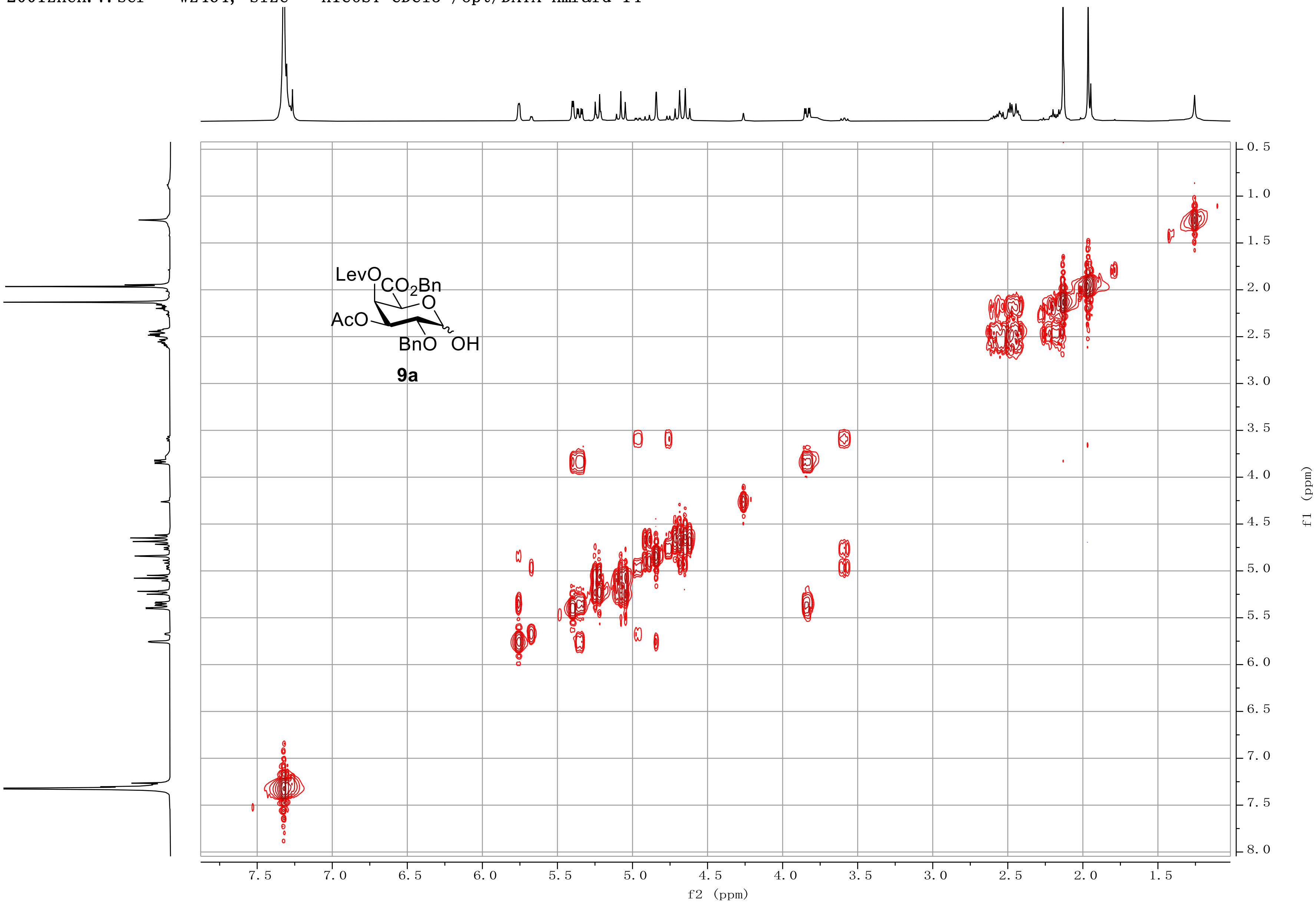

2001zhen.8.ser — wz464, size — c13HSQC CDCl3 /opt/DATA nmrafd 14

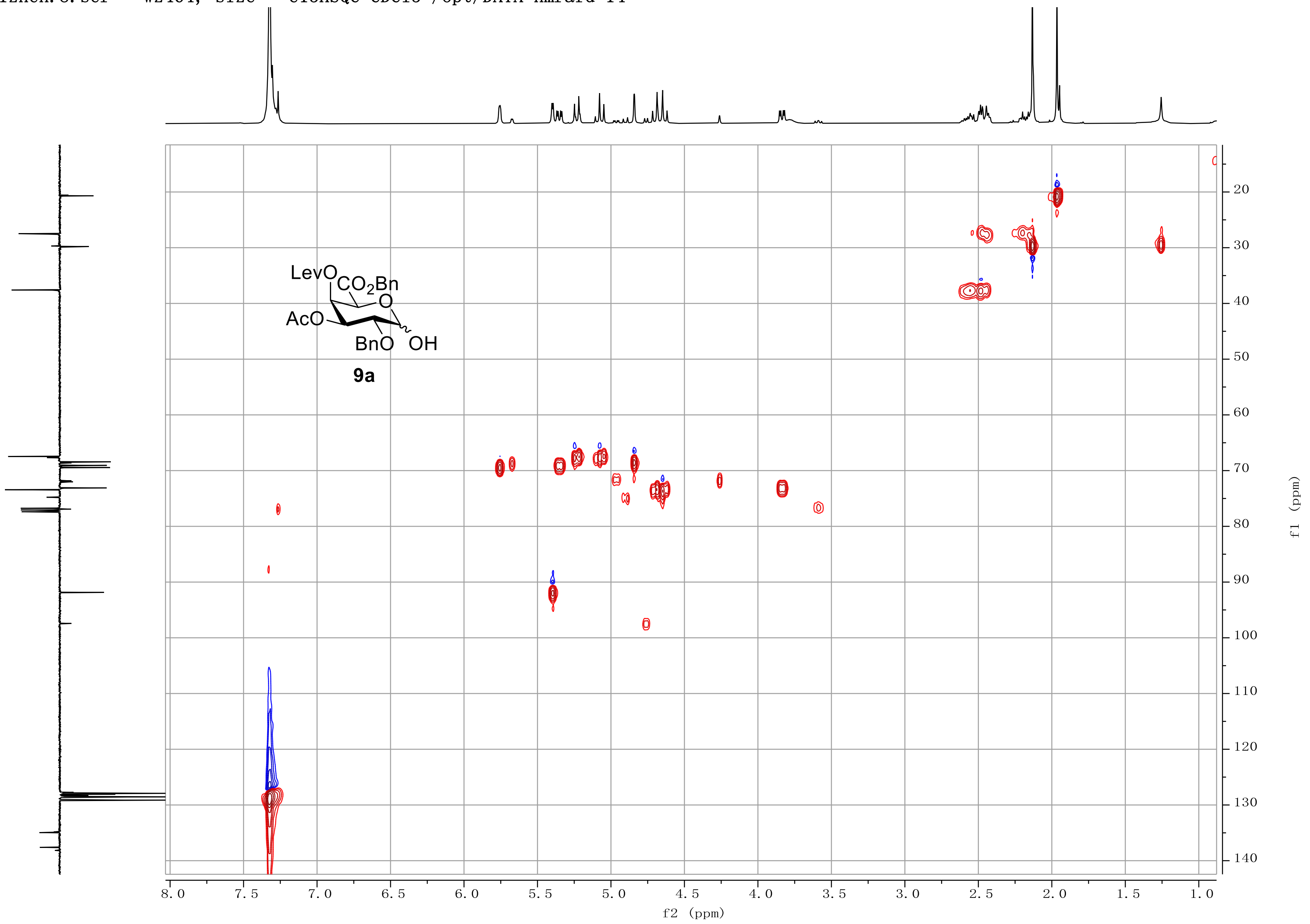

2001zhen.9.ser — wz464, size — c13HMBC CDCl3 /opt/DATA nmrafd 14

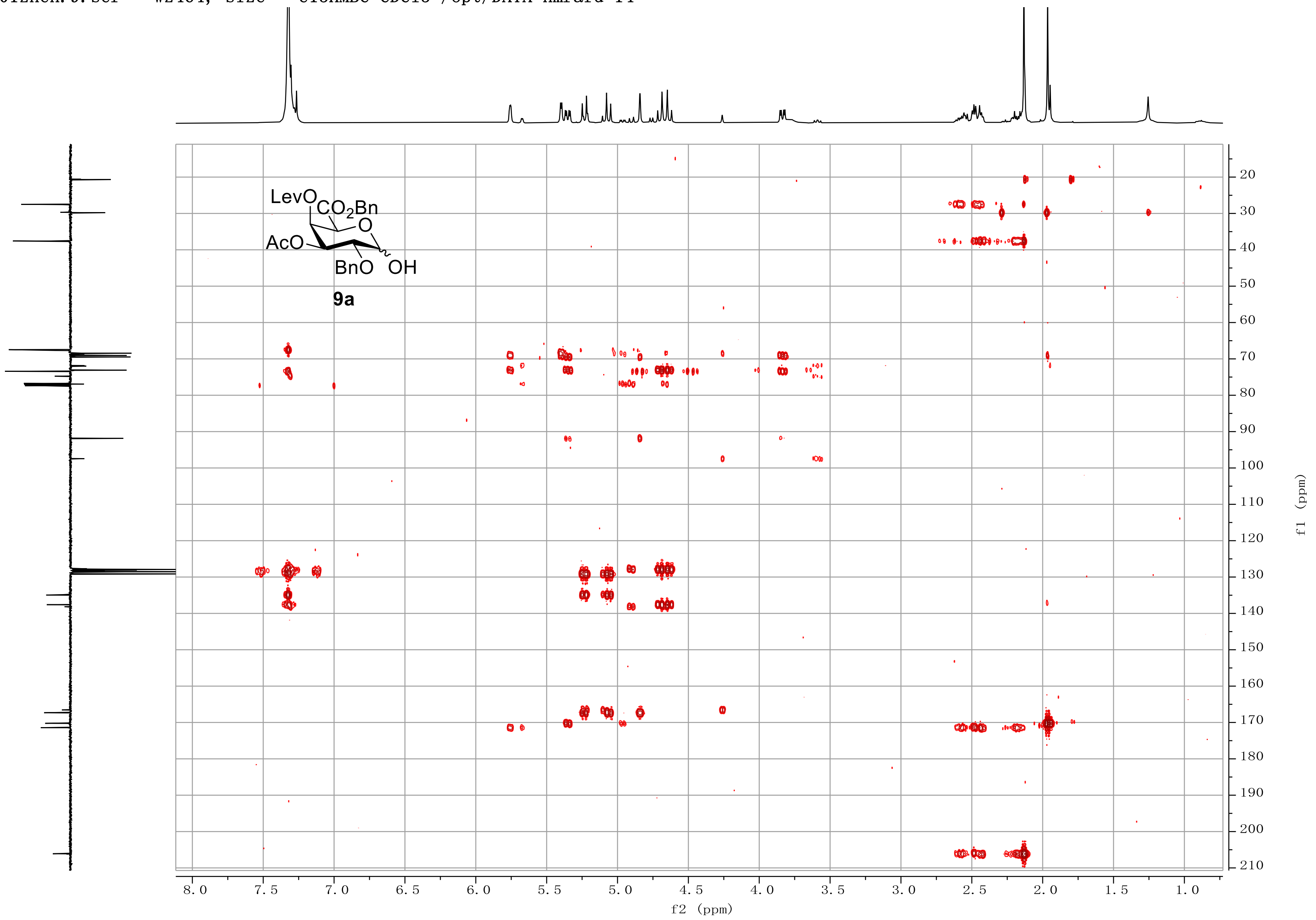

1507Qingju.49.fid — zqj-0477-2 — biosyn1Hfast CDCl3 /opt/DATA nmrafd 6

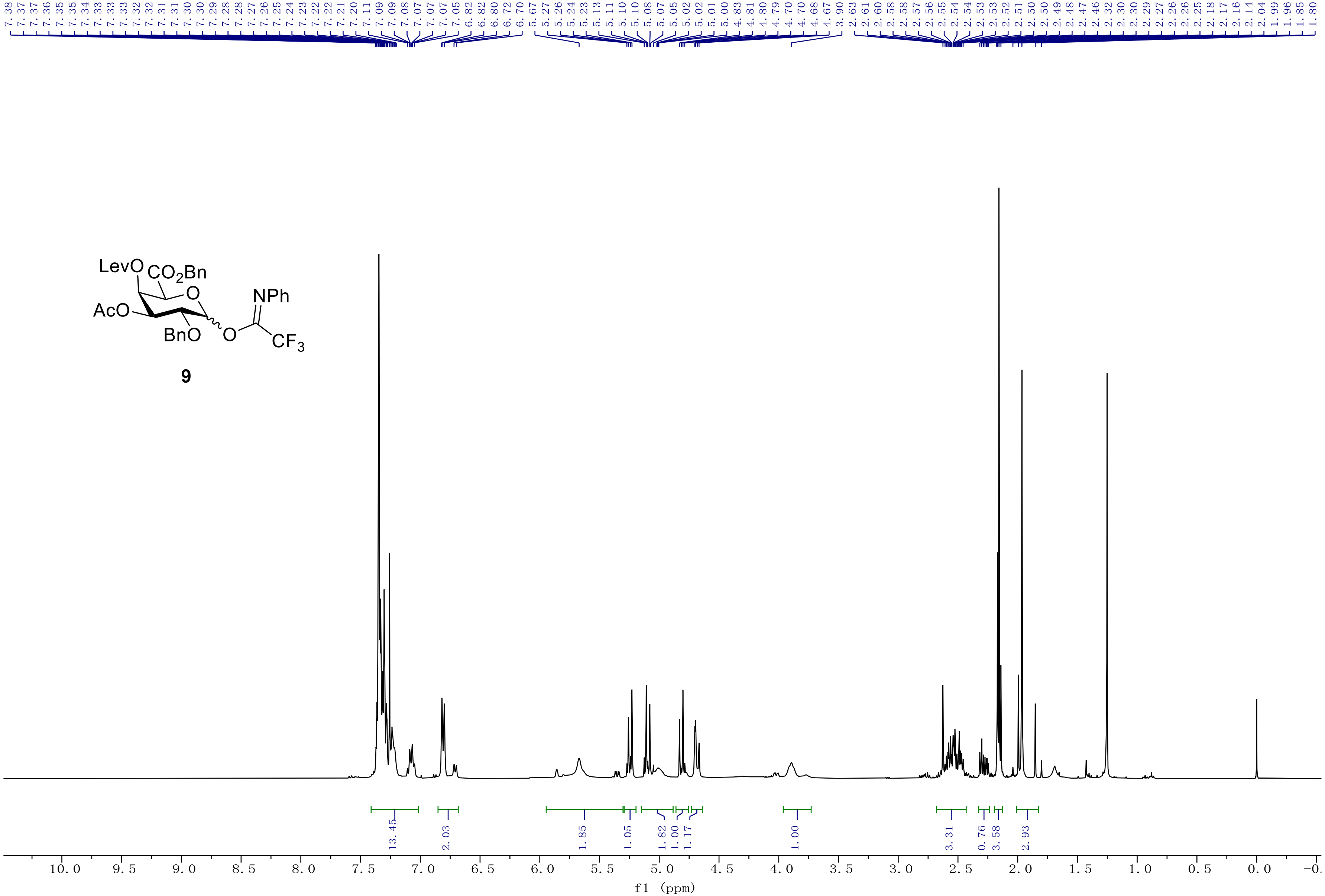

1507Qingju.50.fid — zqj-0477-2 — biosynAPTfast CDCl3 /opt/DATA nmrafd 6

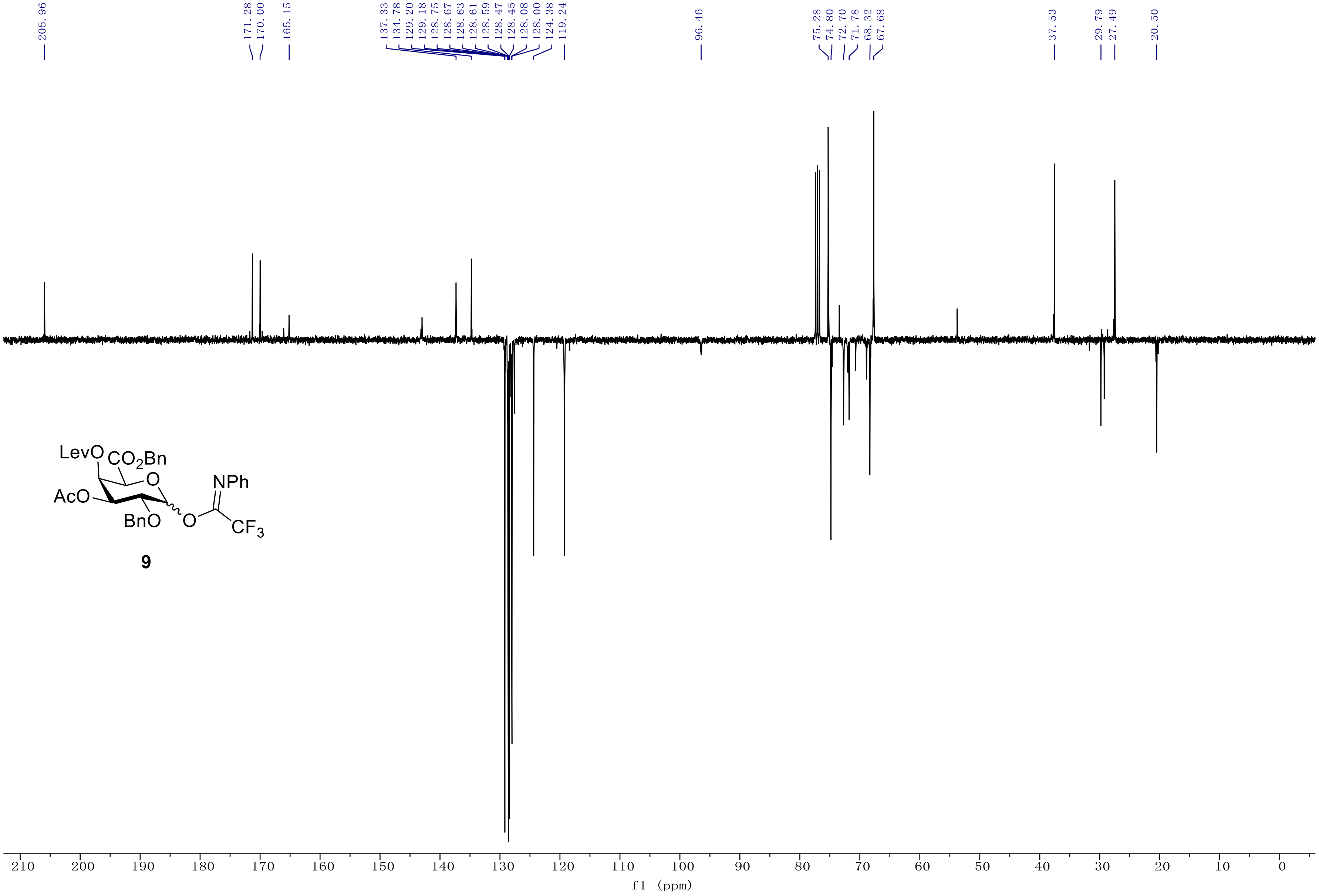

1507Qingju.51.ser — zqj-0477-2 — biosynCOSYfast CDCl3 /opt/DATA nmrafd 6

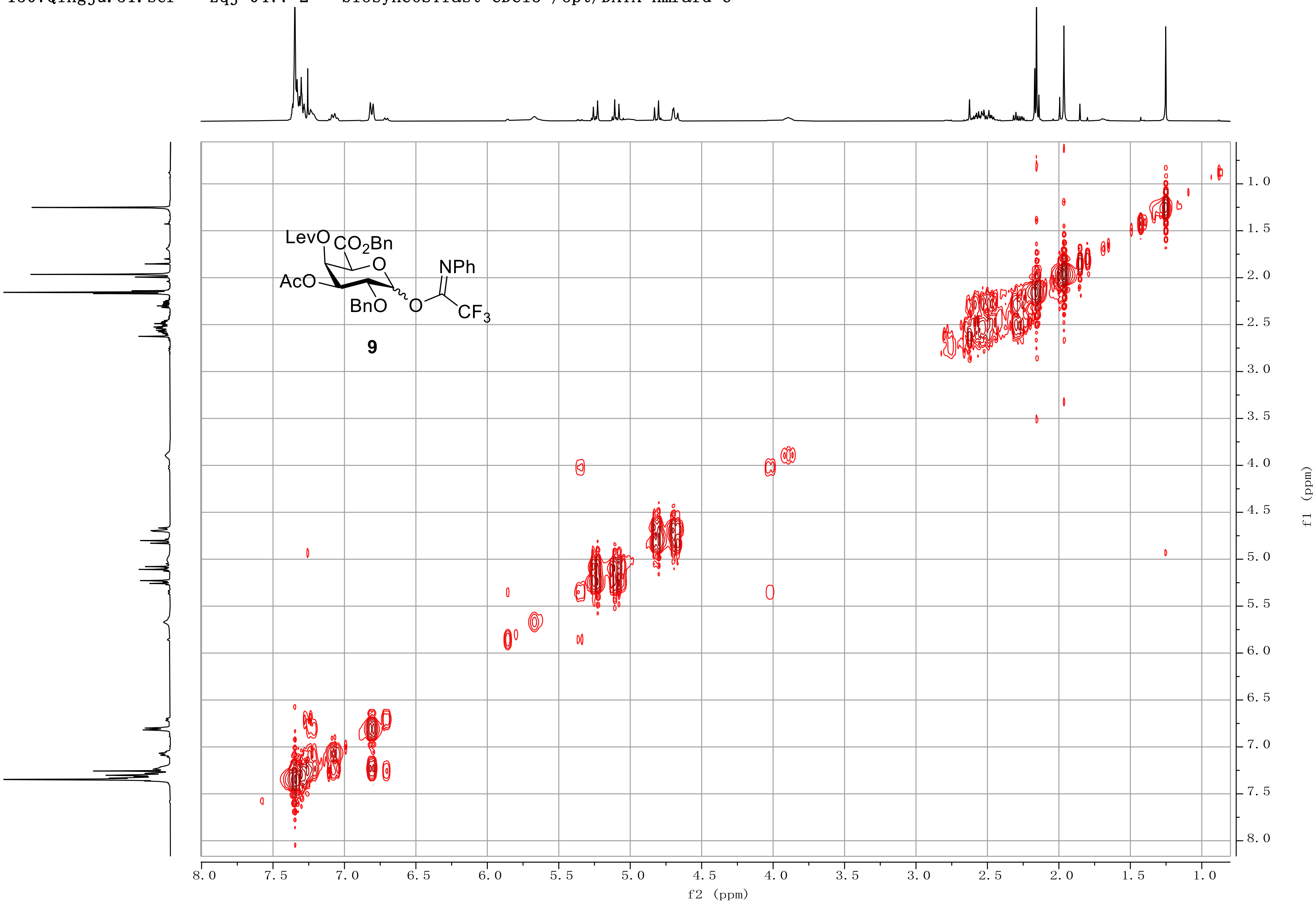

1507Qingju.52.ser — zqj-0477-2 — biosynHSQCfast CDCl3 /opt/DATA nmrafd 6

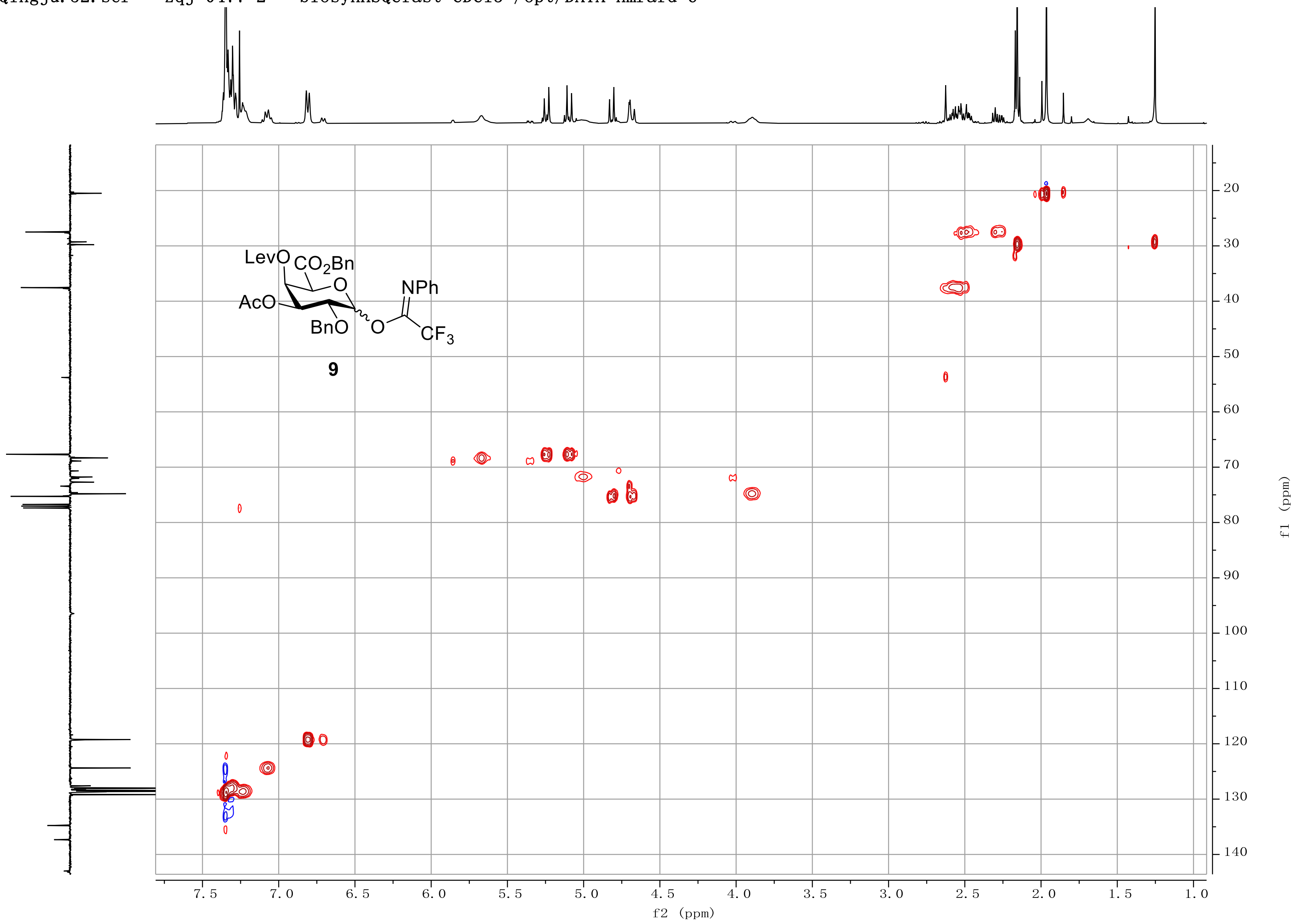

zhen1808biosyn.46.fid — wz492-size — bbo-h1 CDCl3 /opt/topspin2.1 nmrafd 5

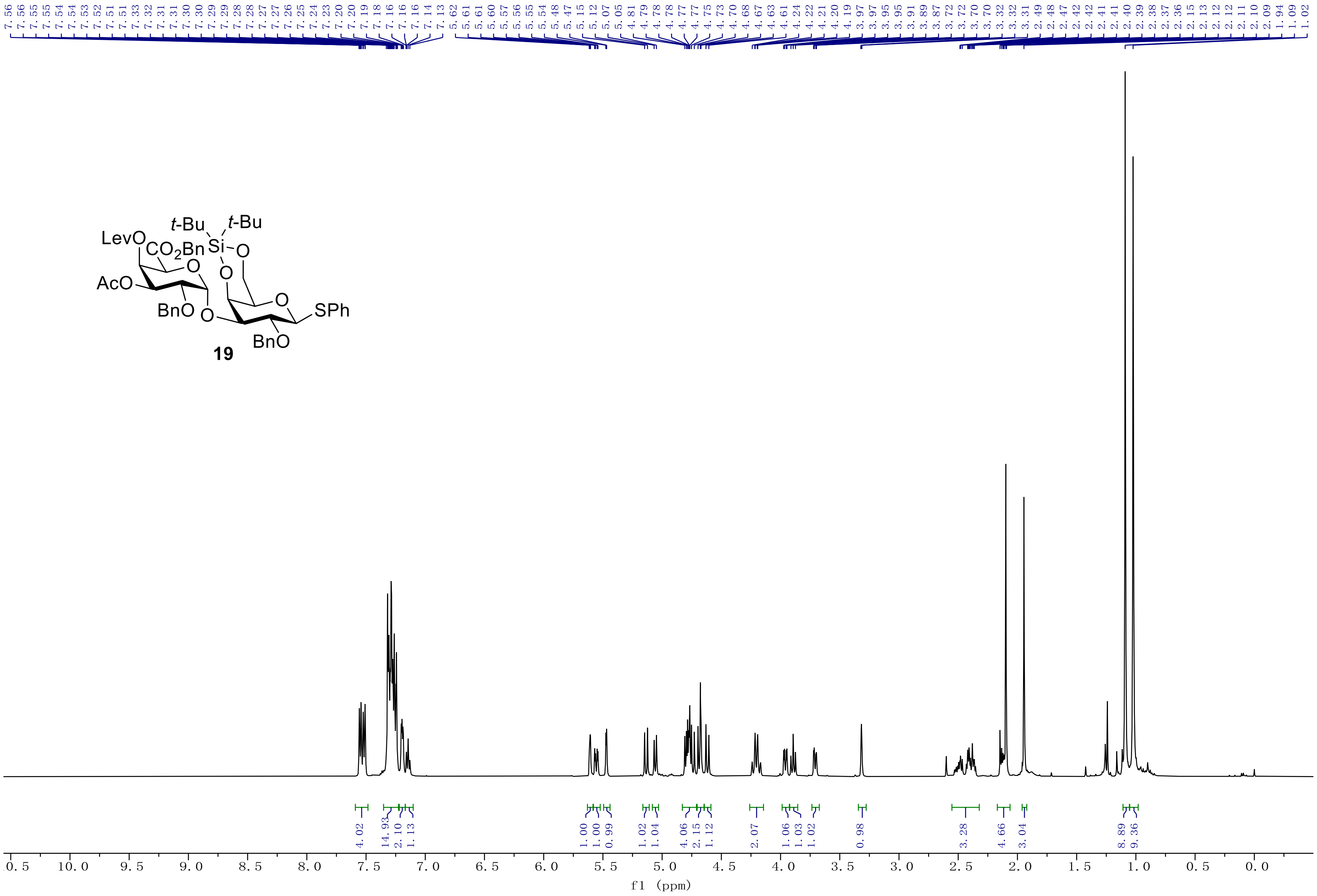

zhen1808biosyn.49.fid — wz492-size — bbo-c13-APT CDCl3 /opt/topspin2.1 nmrafd 5

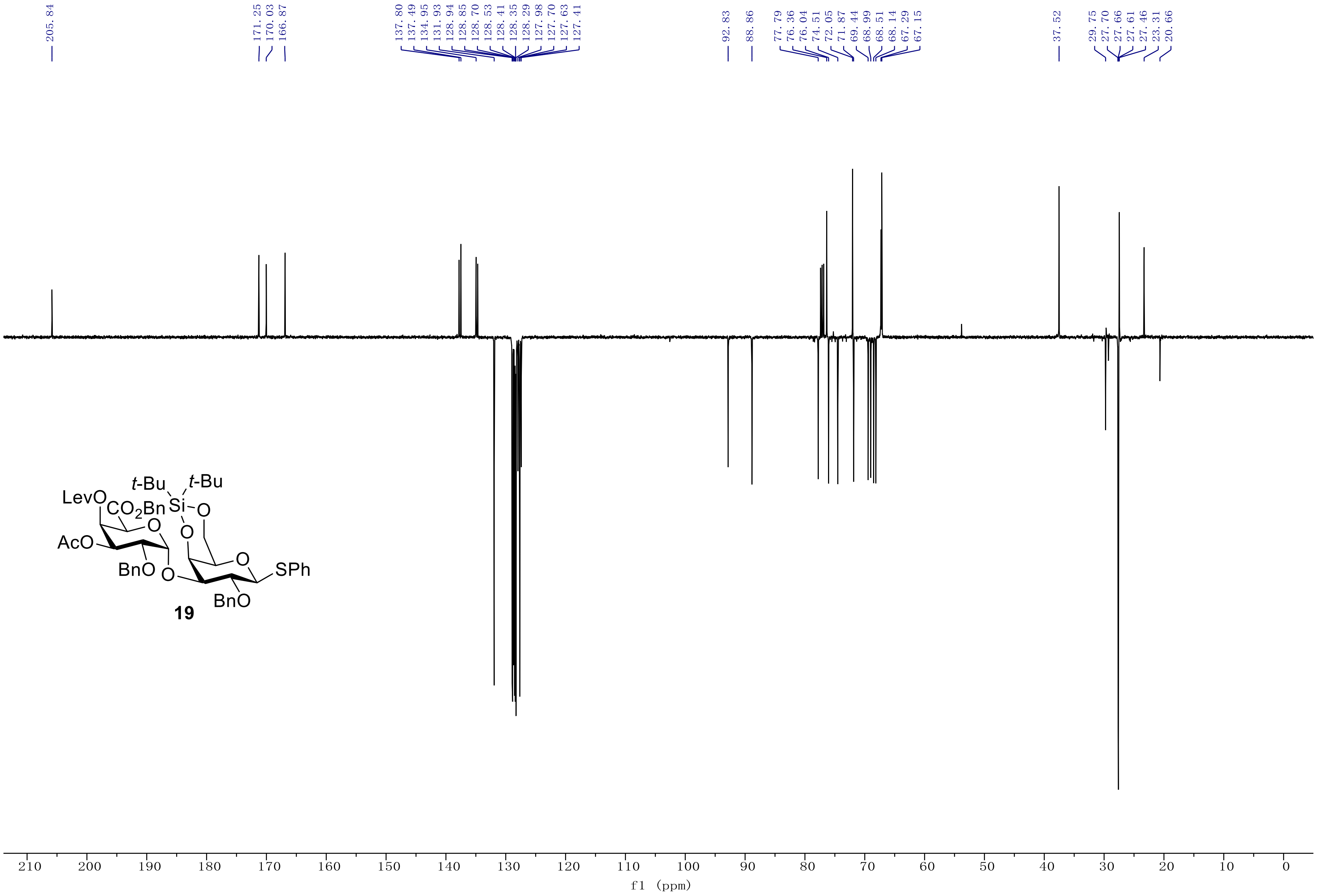

zhen1808biosyn.47.ser — wz492-size — bbo-h1-cosy CDCl3 /opt/topspin2.1 nmrafd 5

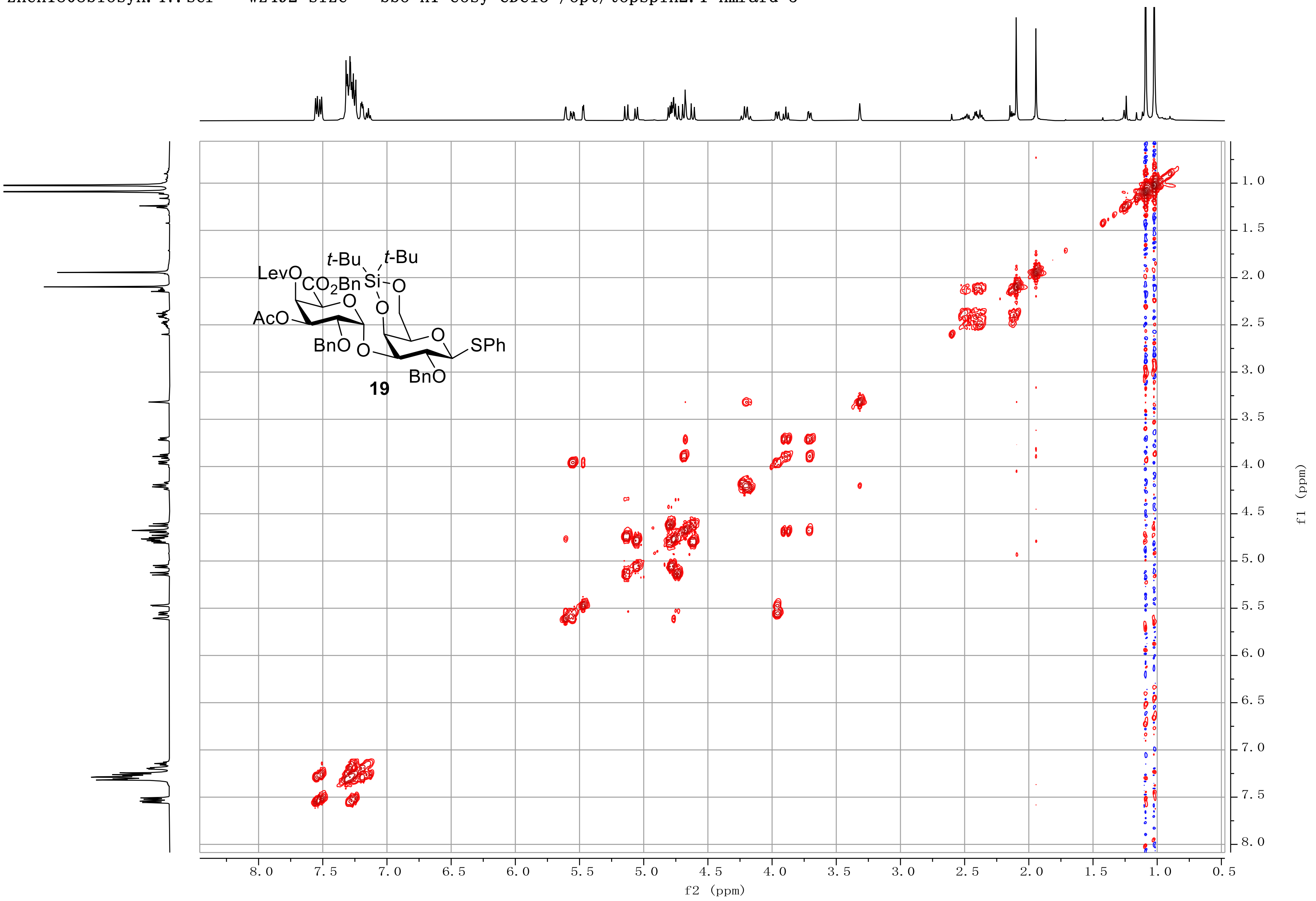

zhen1808biosyn.48.ser — wz492-size — bbo-c13-HSQC CDCl3 /opt/topspin2.1 nmrafd 5

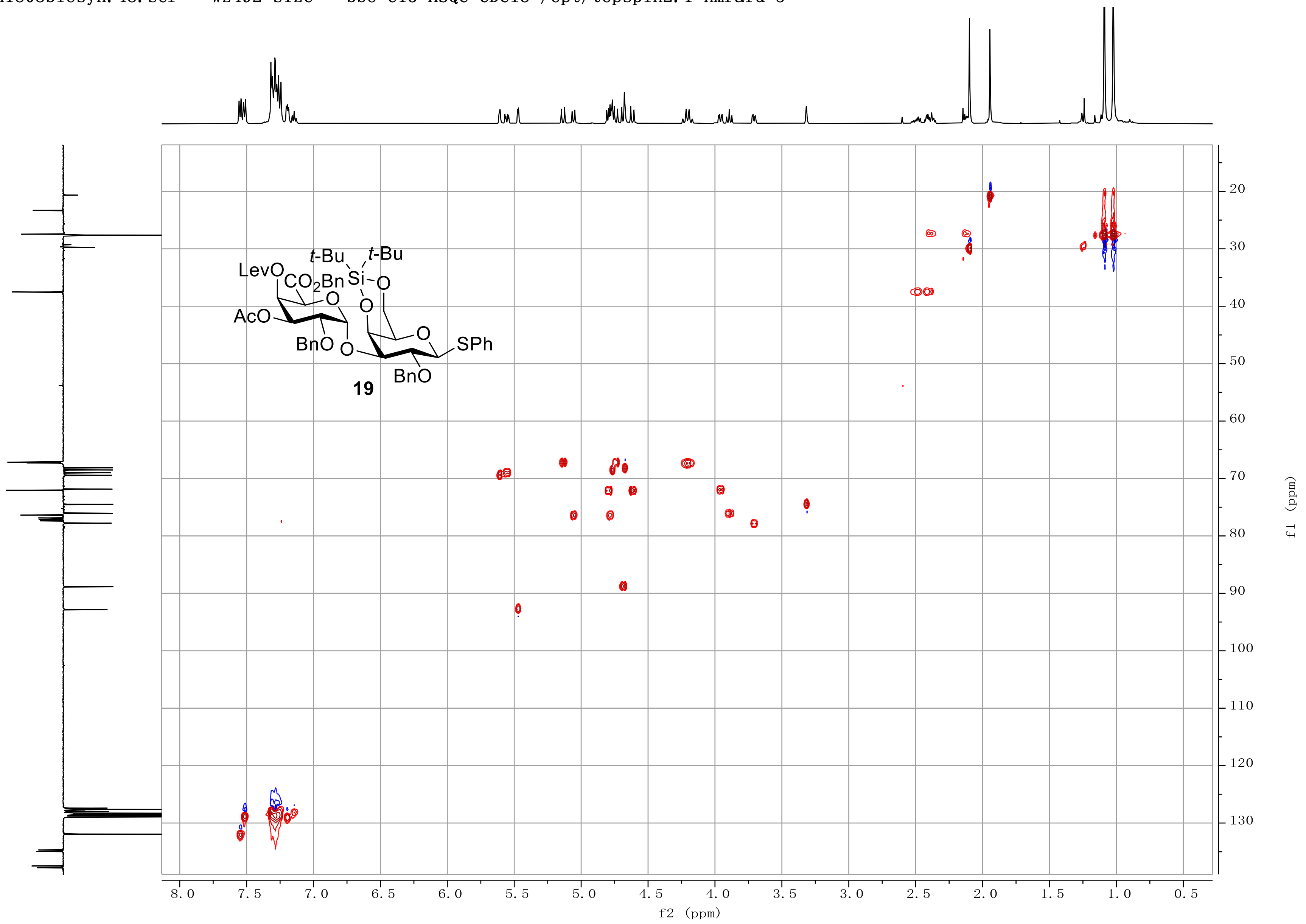

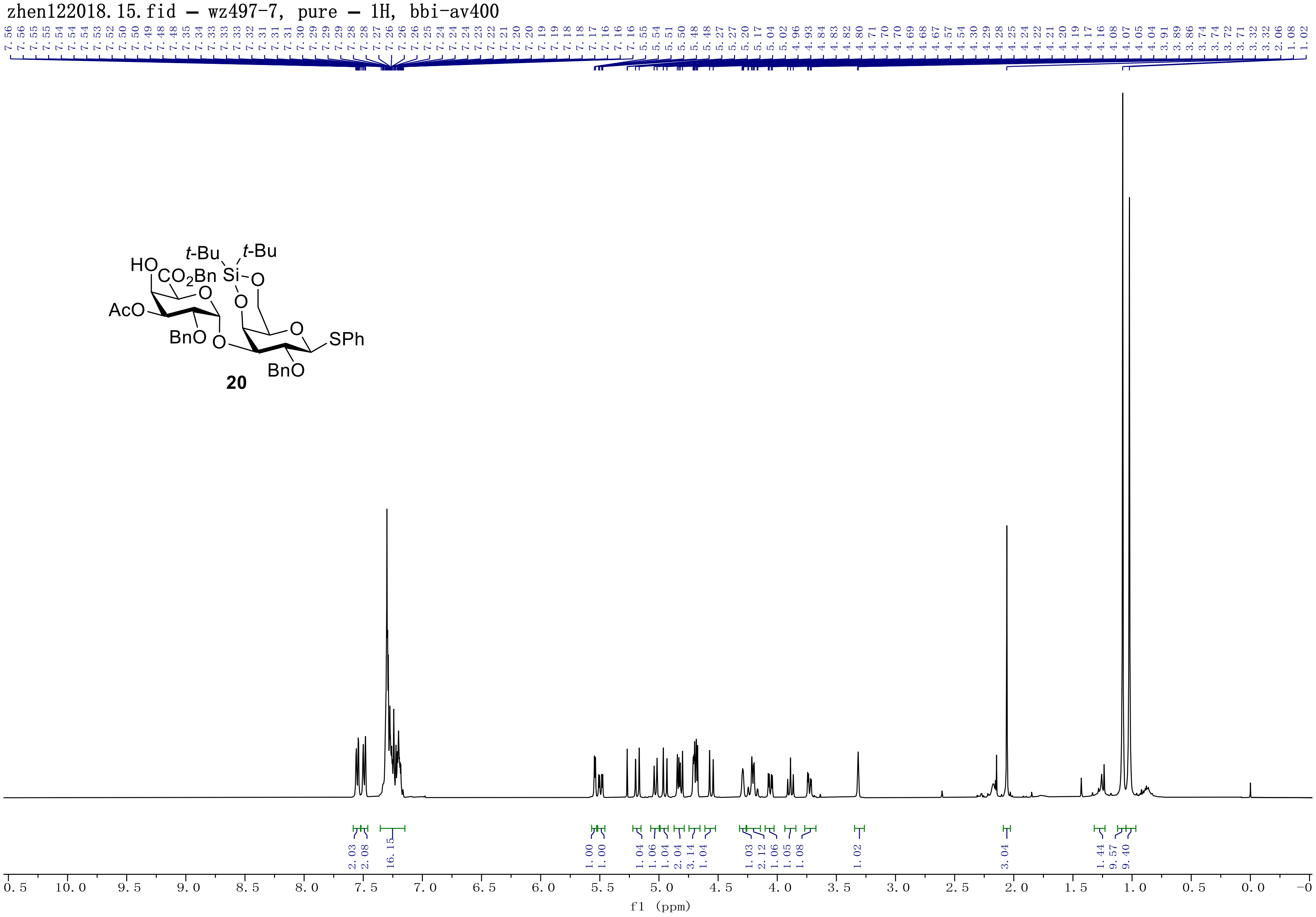
zhen122018.15.fid — wz497-7, pure — 1H, bbi-av400
20
f1 (ppm)

1807zhen.26.fid — wz497 — C13APT CDCl3 /opt/DATA nmrafd 15

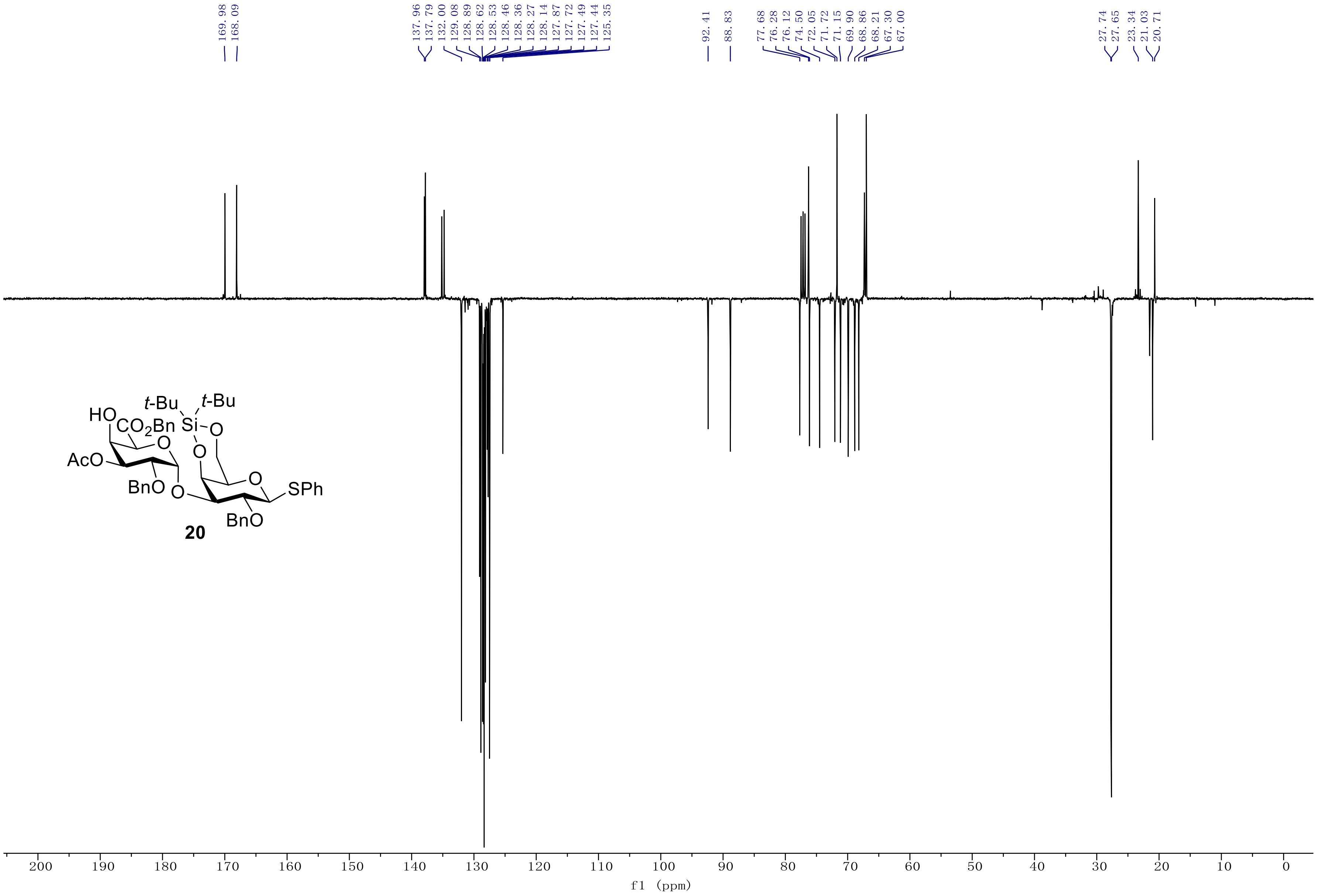

20

1808zhen.18.ser — wz497-3 — h1COSY CDCl3 /opt/DATA nmrafd 30

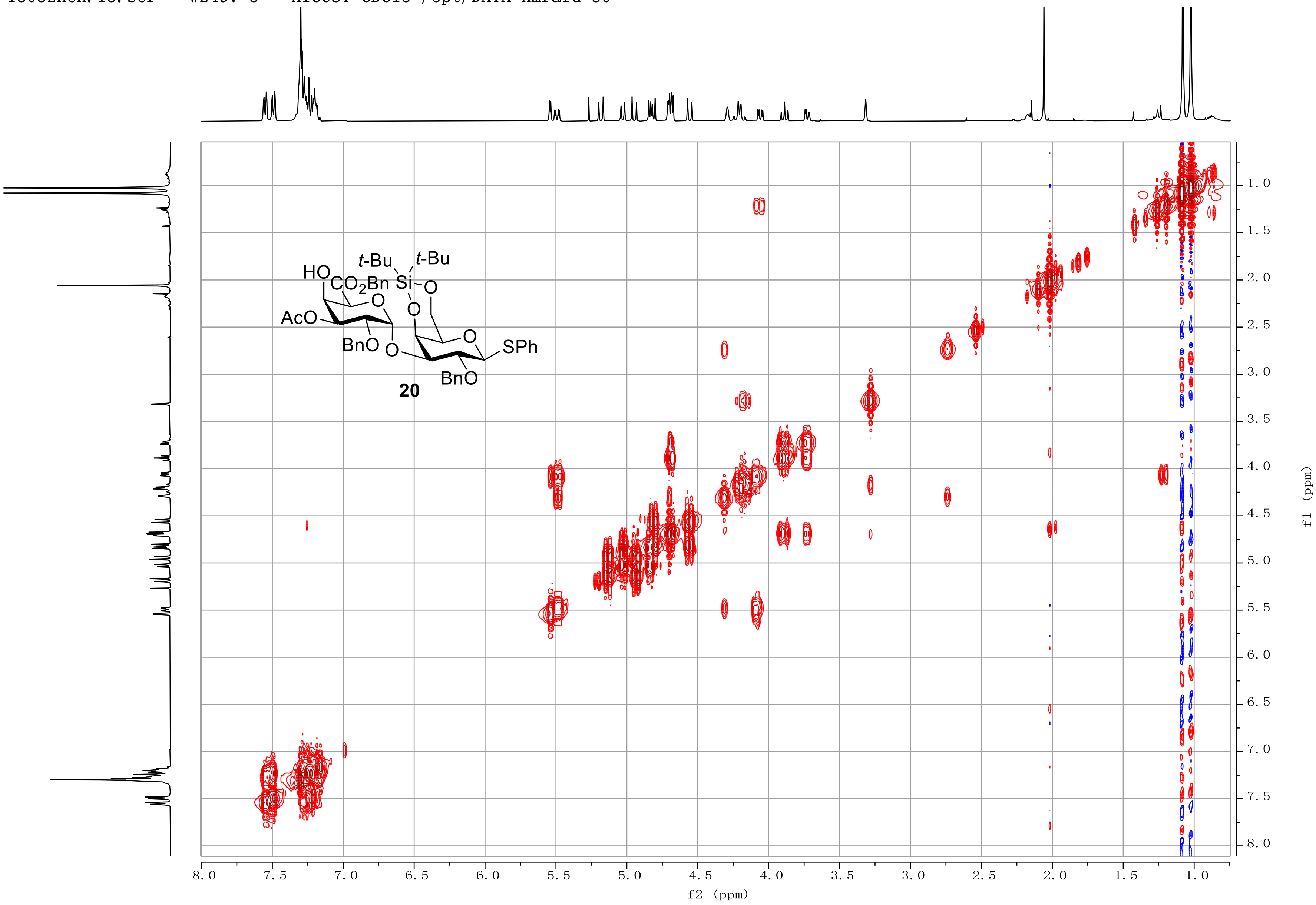

1808zhen.19.ser — wz497-3 — c13HSQC CDCl3 /opt/DATA nmrafd 30

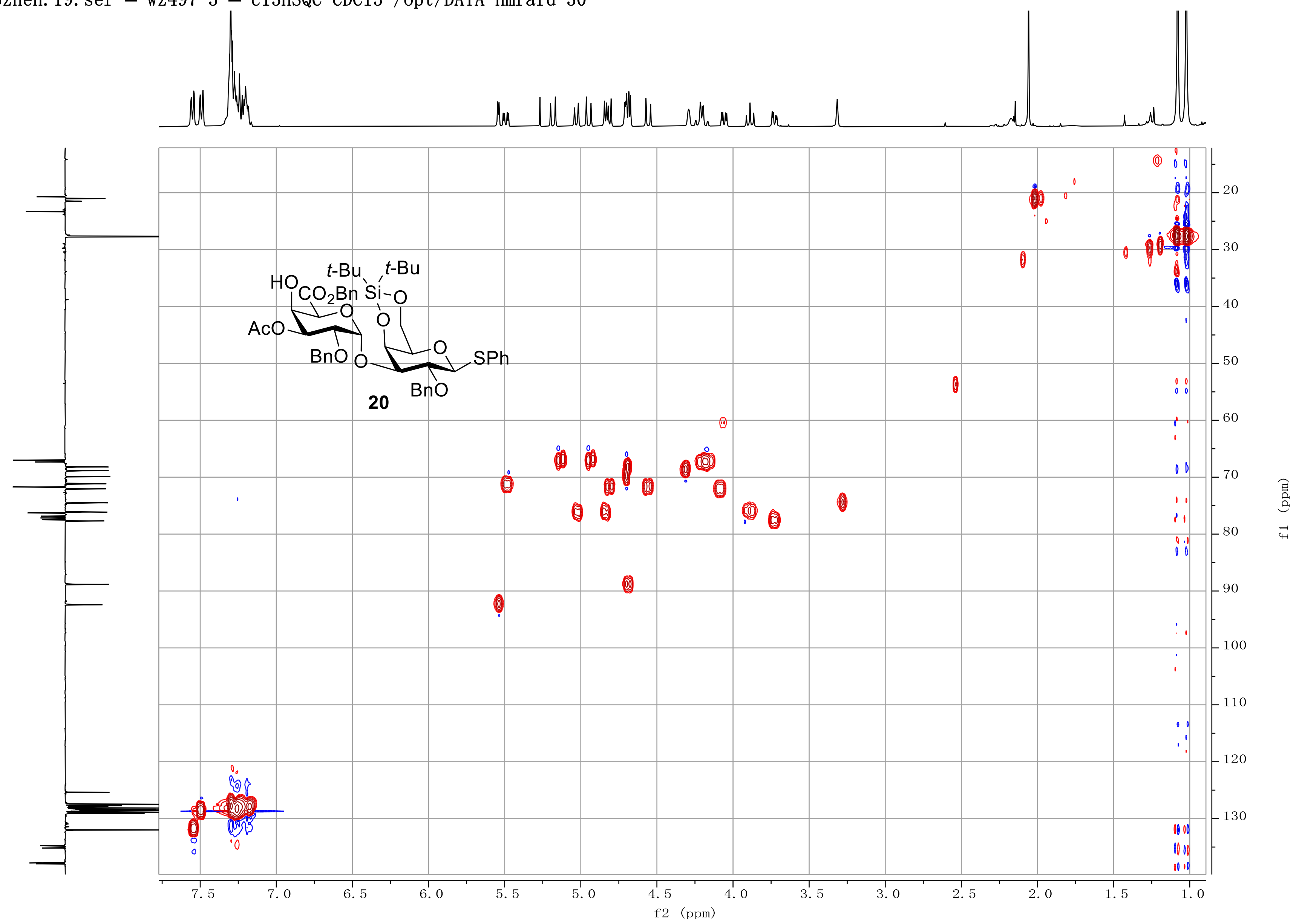

1808zhen.20.ser — wz497-3 — c13HMBC CDCl3 /opt/DATA nmrafd 30

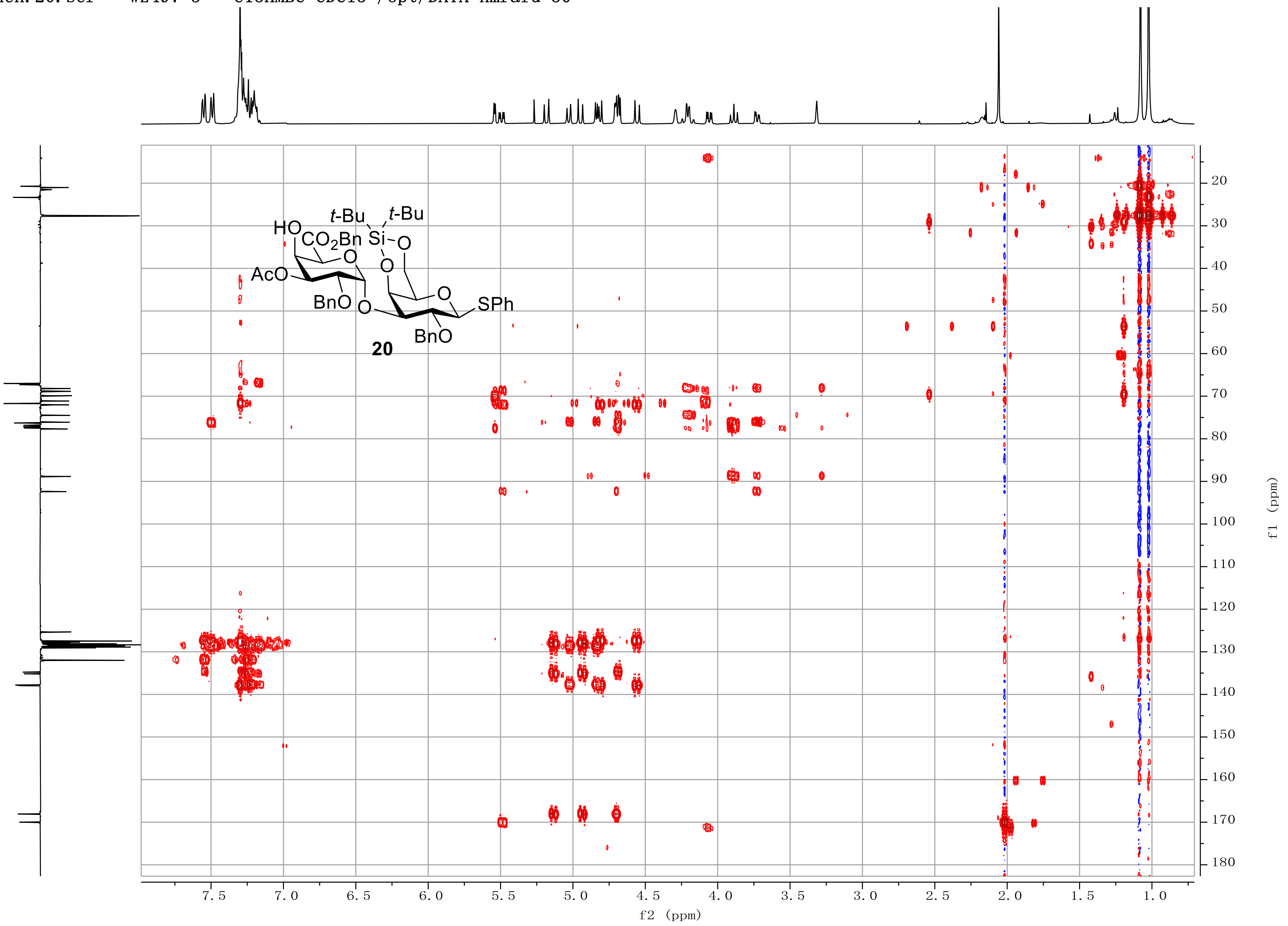

1808zhen.21.ser — wz497-3 — c13HMBCipvGATED CDCl3 /opt/DATA nmrafd 30

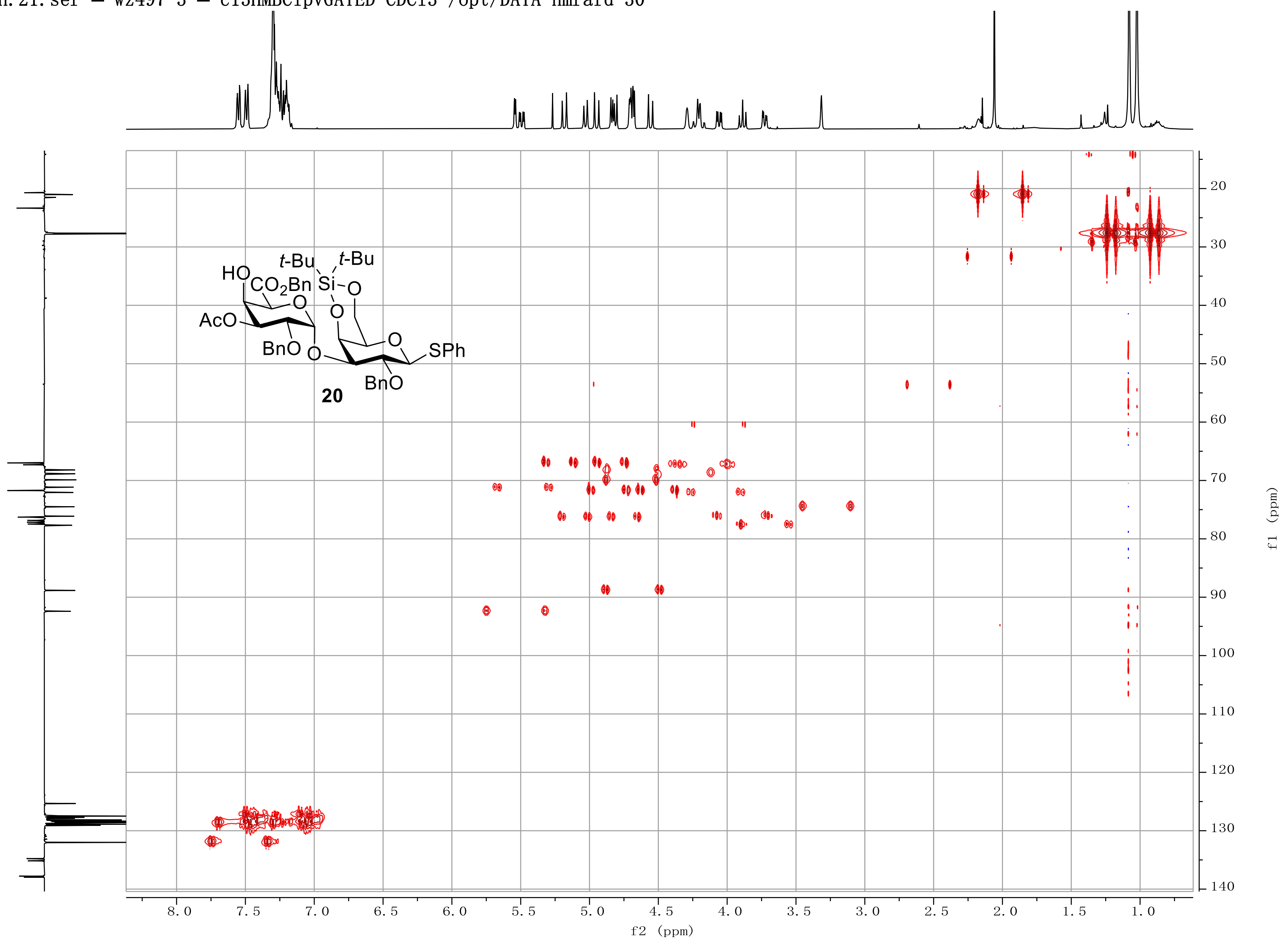

zhen1808biosyn.16.fid — wz498-4 — bbo-h1 CDCl3 /opt/topspin2.1 nmrafd 6

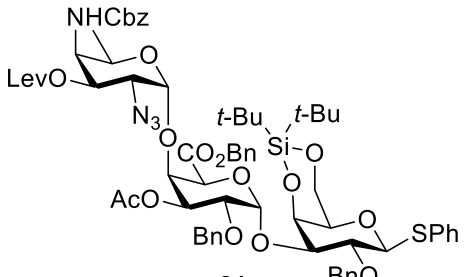

21

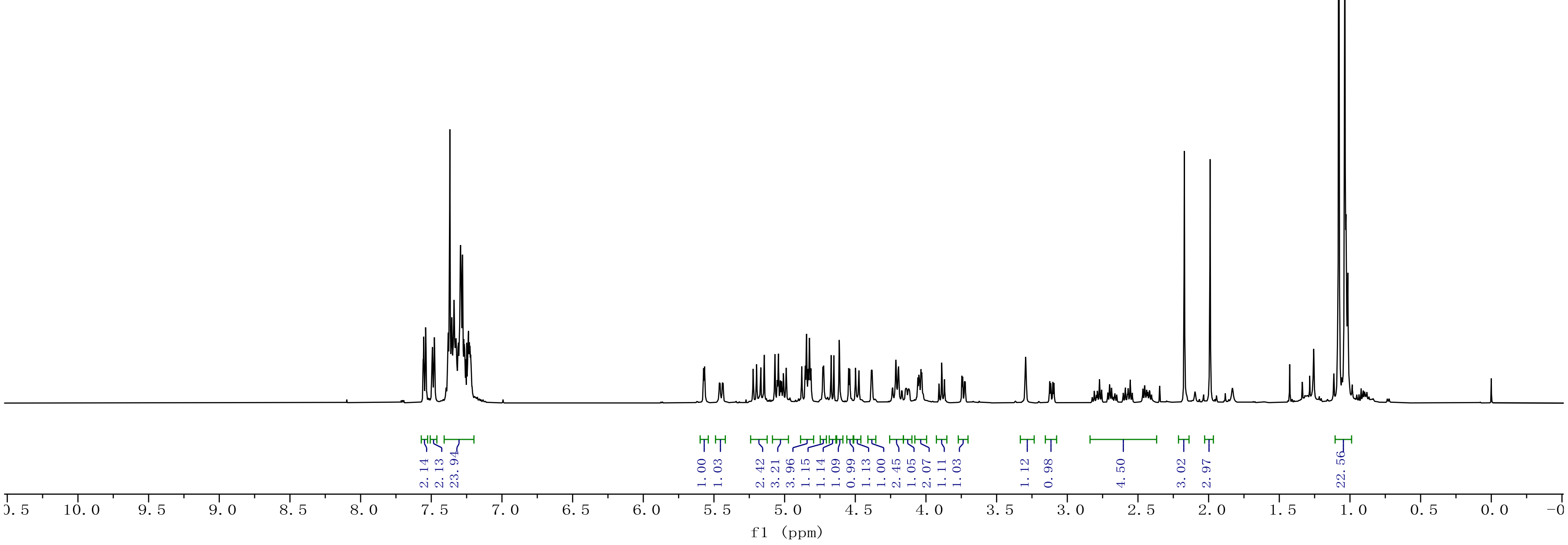

zhen1808biosyn.19.fid — wz498-4 — bbo-c13-APT CDCl3 /opt/topspin2.1 nmrafd 6

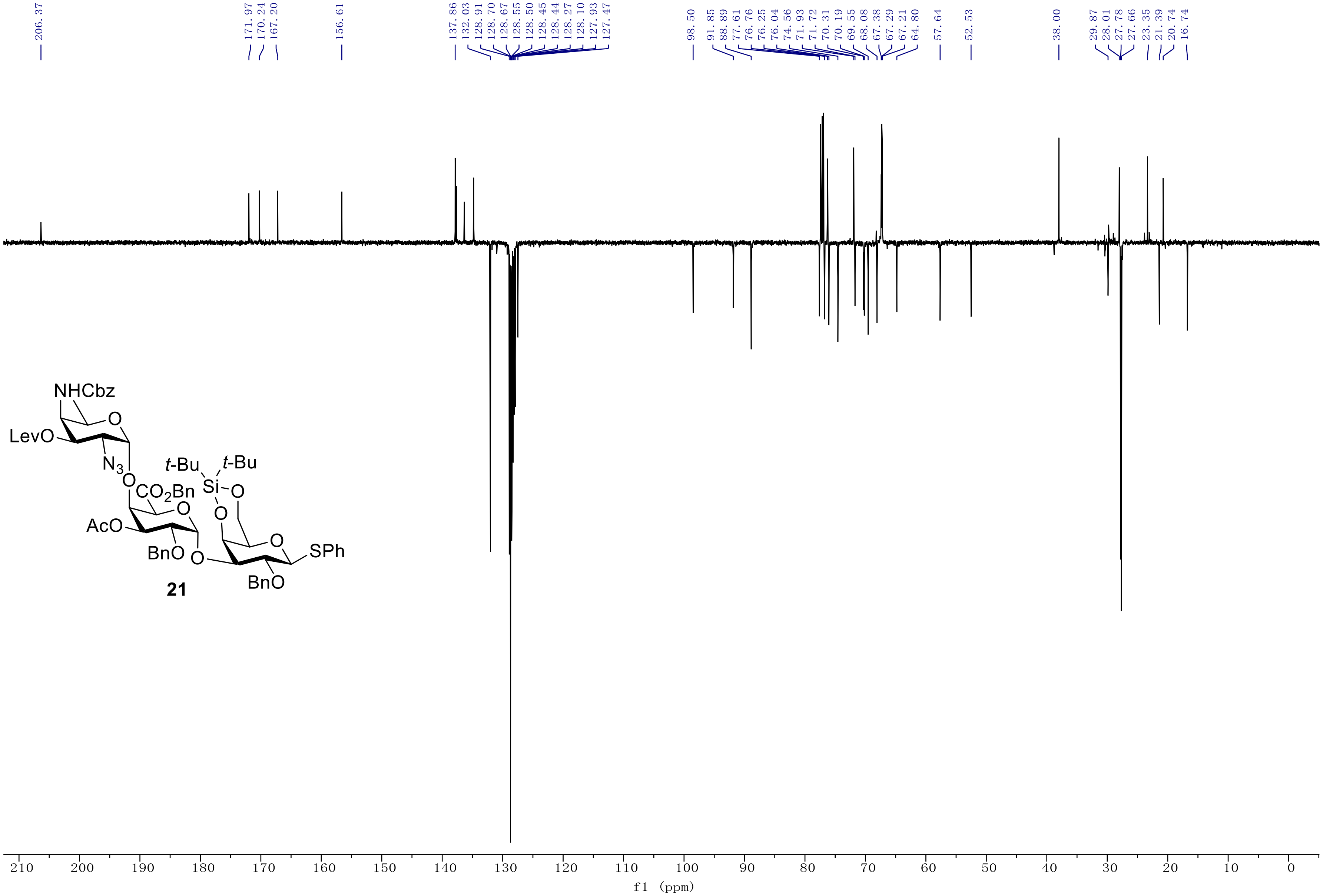

zhen1808biosyn.17.ser — wz498-4 — bbo-h1-cosy CDCl3 /opt/topspin2.1 nmrafd 6

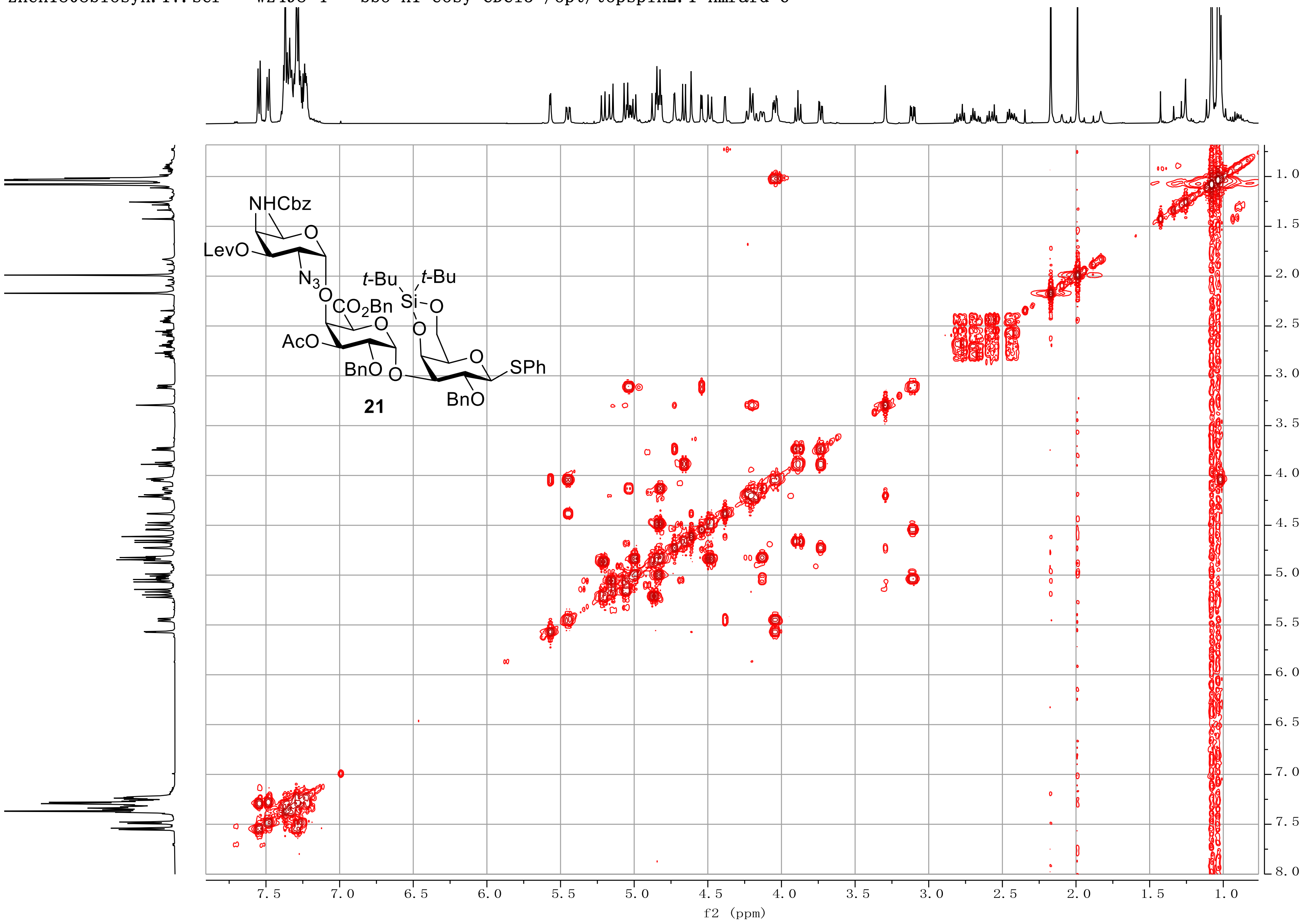

zhen1808biosyn.18.ser — wz498-4 — bbo-c13-HSQC CDCl3 /opt/topspin2.1 nmrafd 6

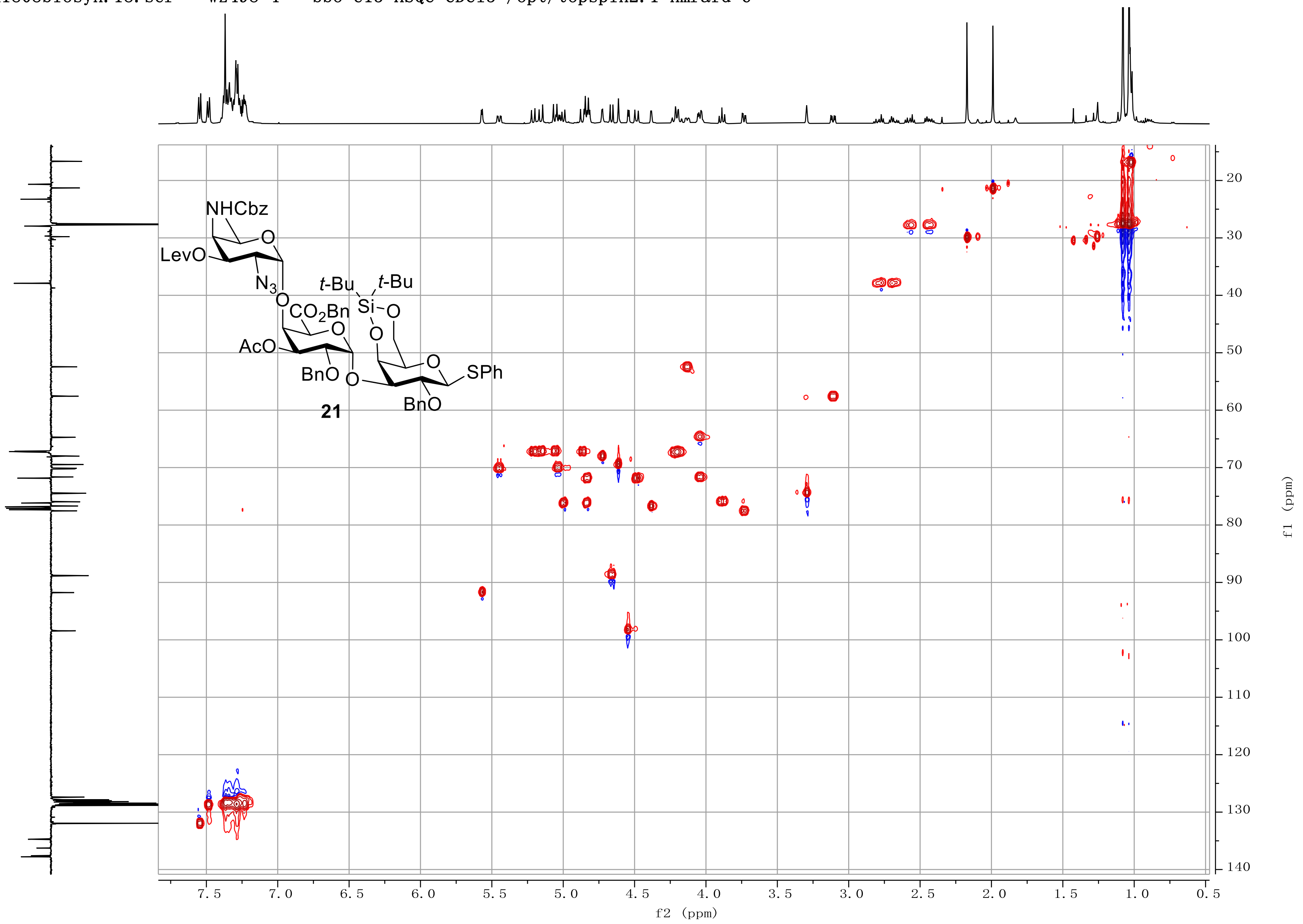

1809zhen.1.fid — wz461-2 — h1 CDCl3 /opt/DATA nmrafd 20

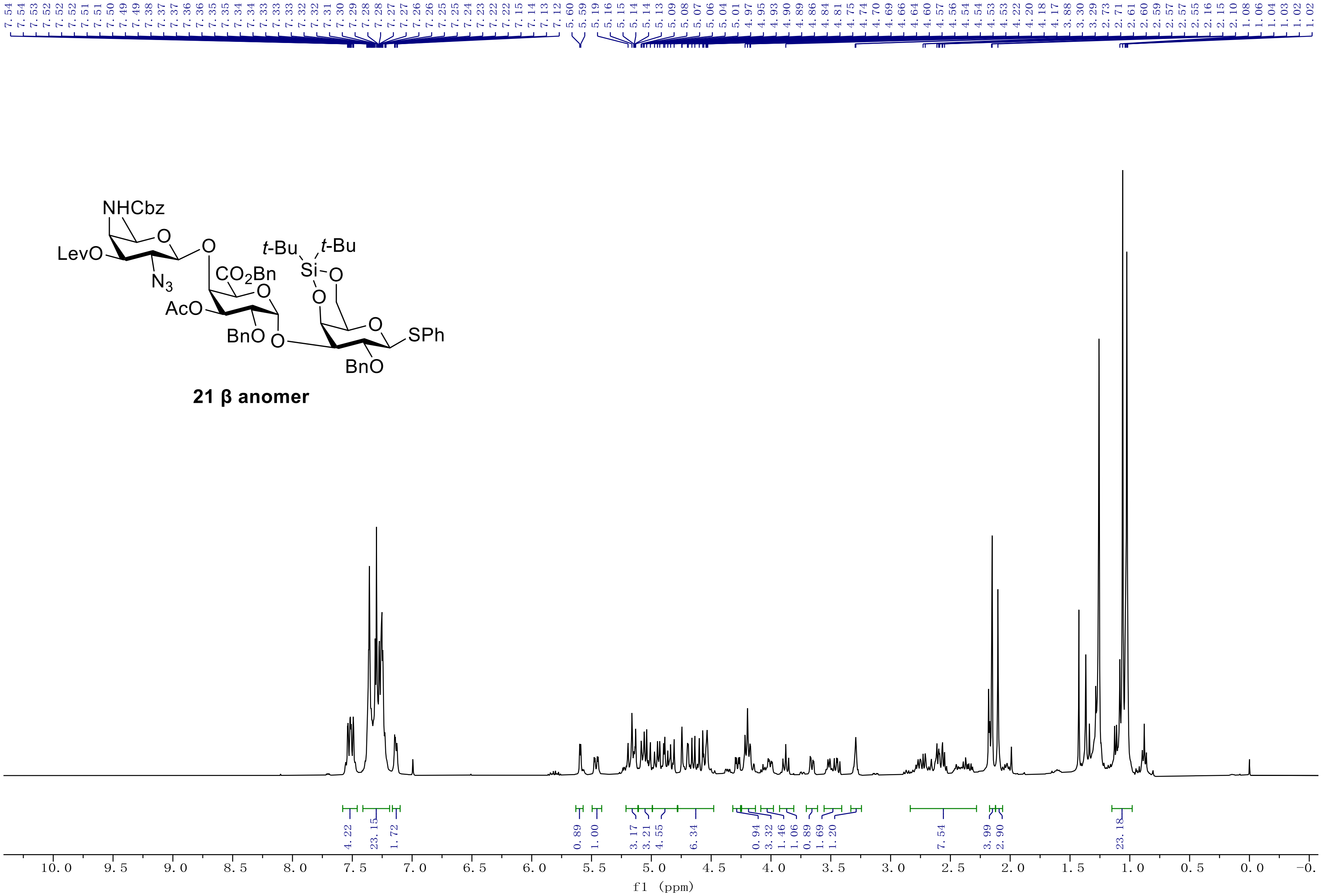

21 β anomer

zhen1808biosyn.13.fid — wz498-3 — bbo-c13-APT CDCl3 /opt/topspin2.1 nmrafd 15

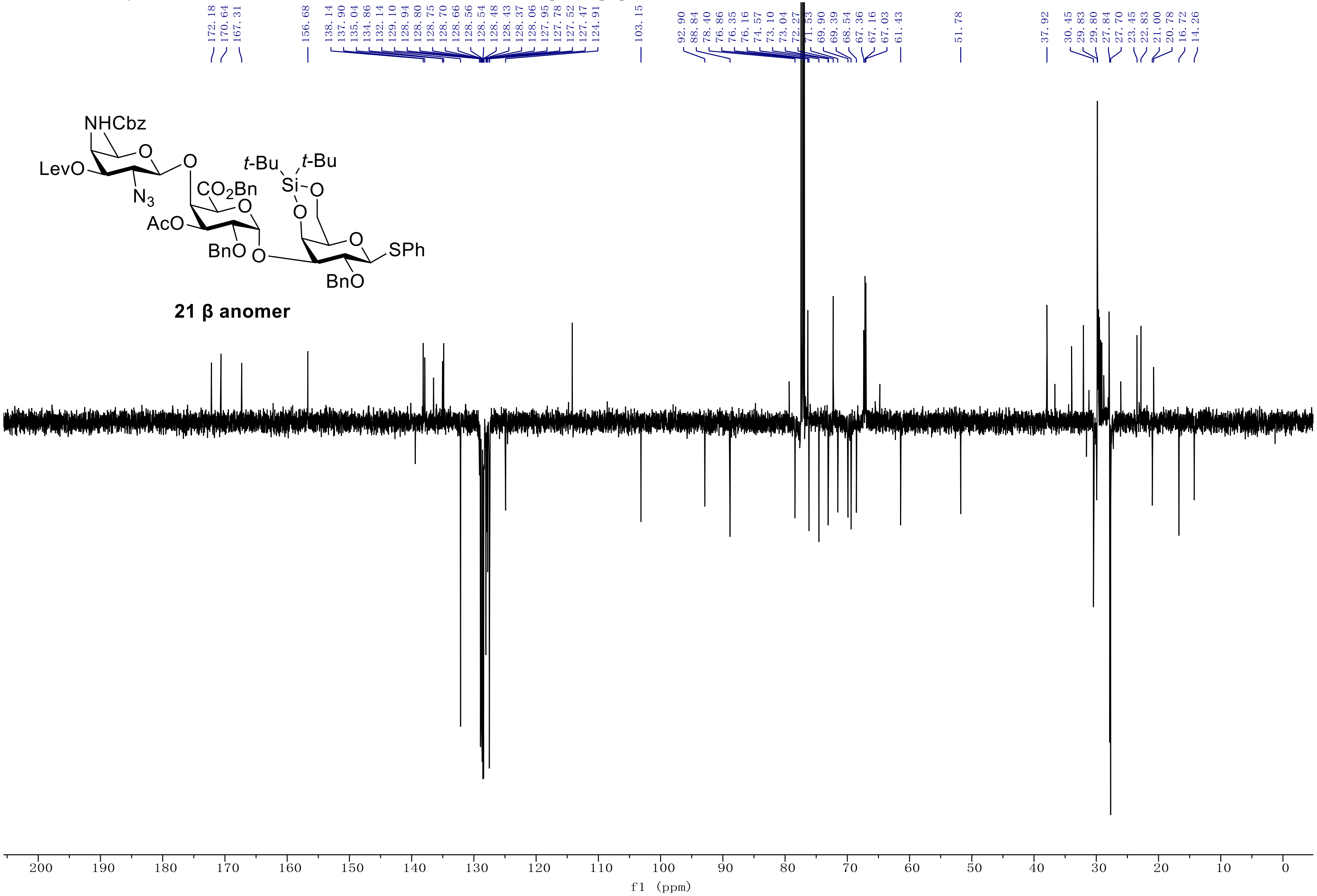

21 β anomer

1809zhen.3.ser — wz461-2 — h1COSY CDCl3 /opt/DATA nmrafd 20

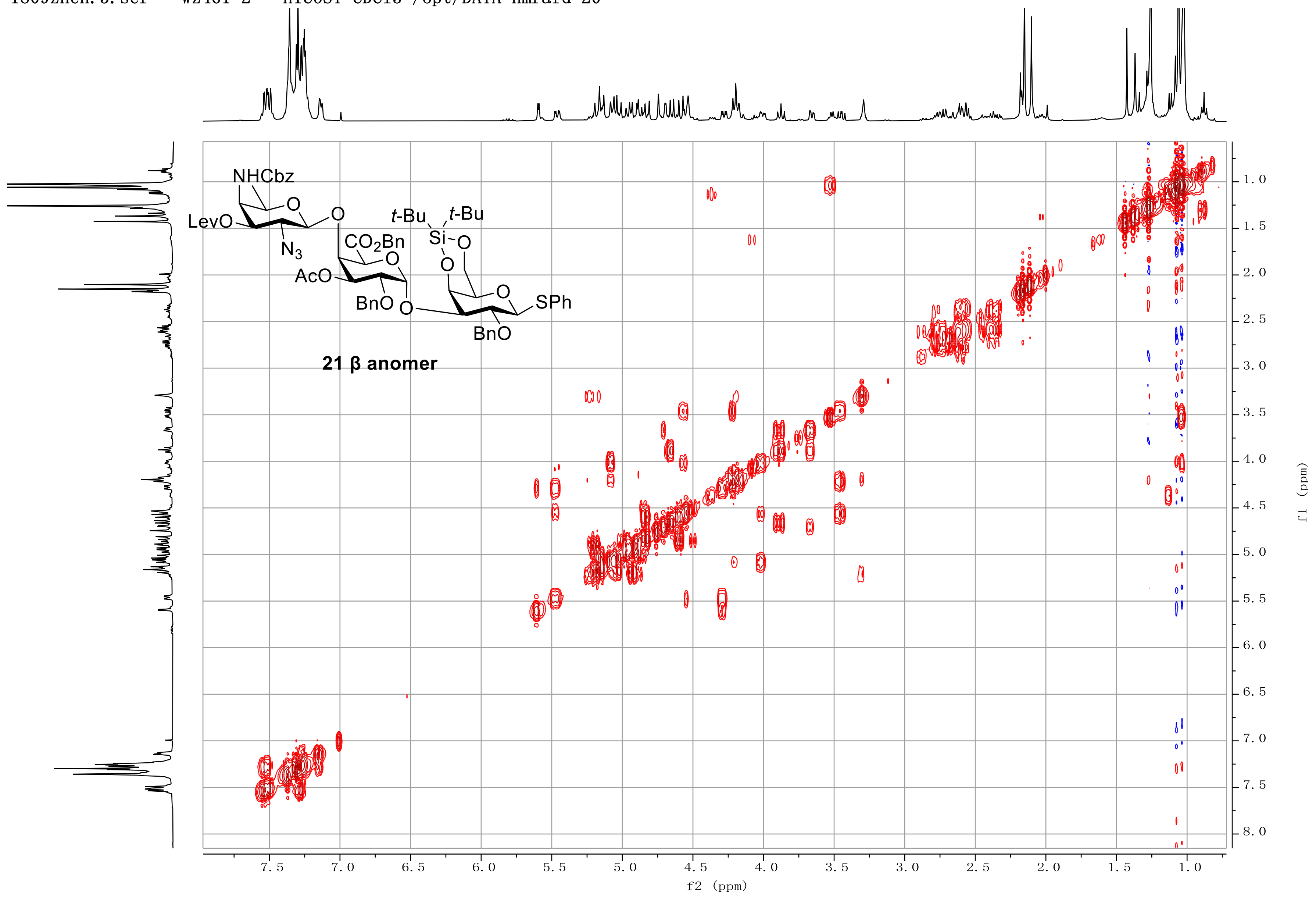

zhen1808biosyn.12.ser — wz498-3 — bbo-c13-HSQC CDCl3 /opt/topspin2.1 nmrafd 15

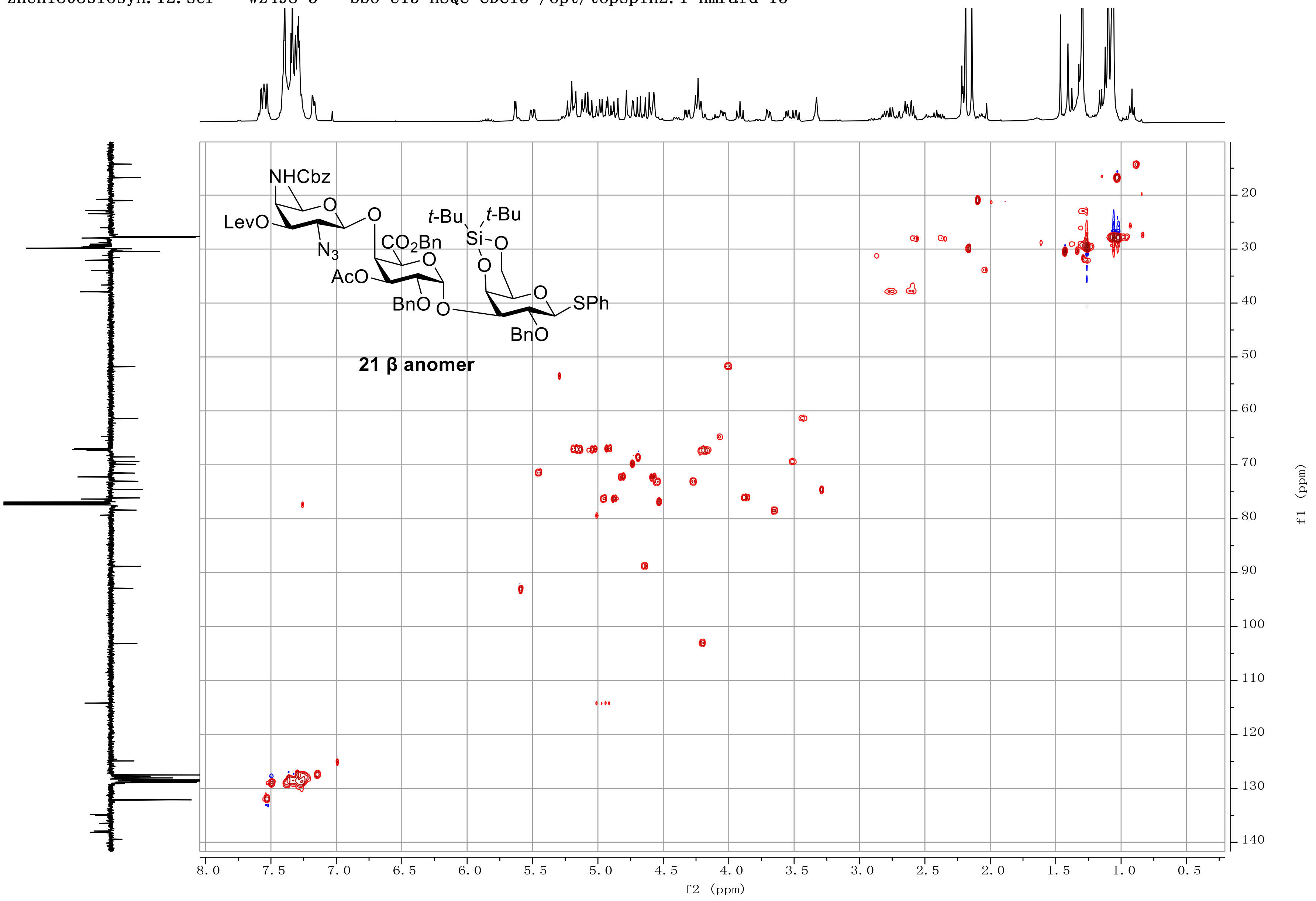

zhen1910biosyn.98.fid — wz503, size — bbo-h1 CDCl3 /opt/topspin2.1 nmrafd 10

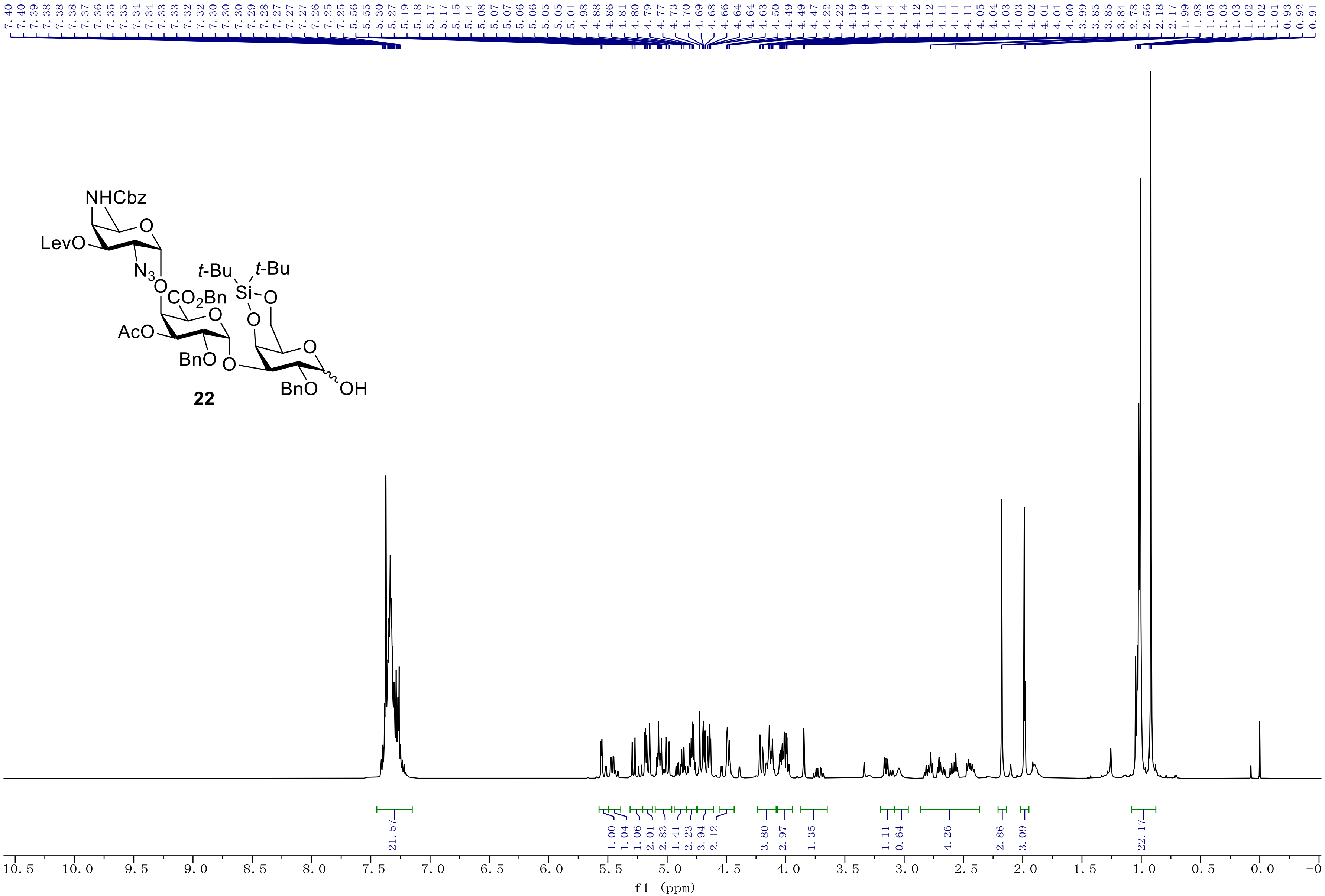

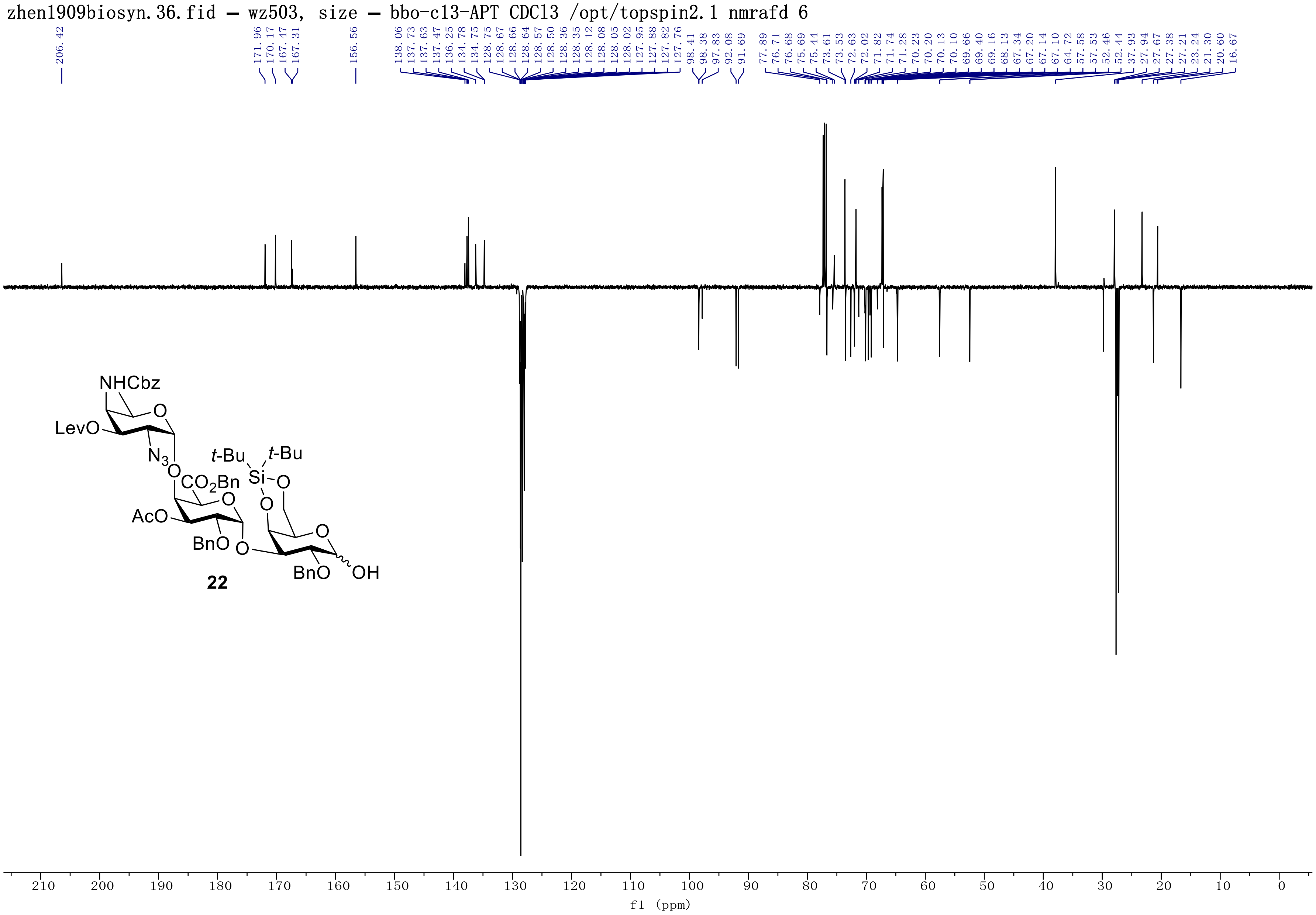
zhen1909biosyn.36.fid — wz503, size — bbo-c13-APT CDCl3 /opt/topspin2.1 nmrafd 6
206.42
171.96
170.17
167.47
167.31
156.56
138.06
137.73
137.63
137.47
136.25
134.78
134.75
128.75
128.67
128.66
128.64
128.57
128.50
128.36
128.35
128.12
128.08
128.05
128.02
127.95
127.88
127.82
127.76
98.41
98.38
97.83
92.08
91.69
77.89
76.71
76.68
75.69
75.44
73.61
73.53
72.63
72.02
71.82
71.74
71.28
70.23
70.20
70.13
70.10
69.66
69.40
69.16
68.13
67.34
67.20
67.14
67.10
64.72
57.58
57.53
52.46
52.44
37.93
27.94
27.67
27.38
27.21
23.24
21.30
20.60
16.67
NHCbz
LevO
O
N3
CO2Bn
AcO
BnO
t-Bu
t-Bu
Si
OH
22
210 200 190 180 170 160 150 140 130 120 110 100 90 80 70 60 50 40 30 20 10 0
f1 (ppm)

zhen1909biosyn.34.ser — wz503, size — bbo-h1-cosy CDCl3 /opt/topspin2.1 nmrafd 6

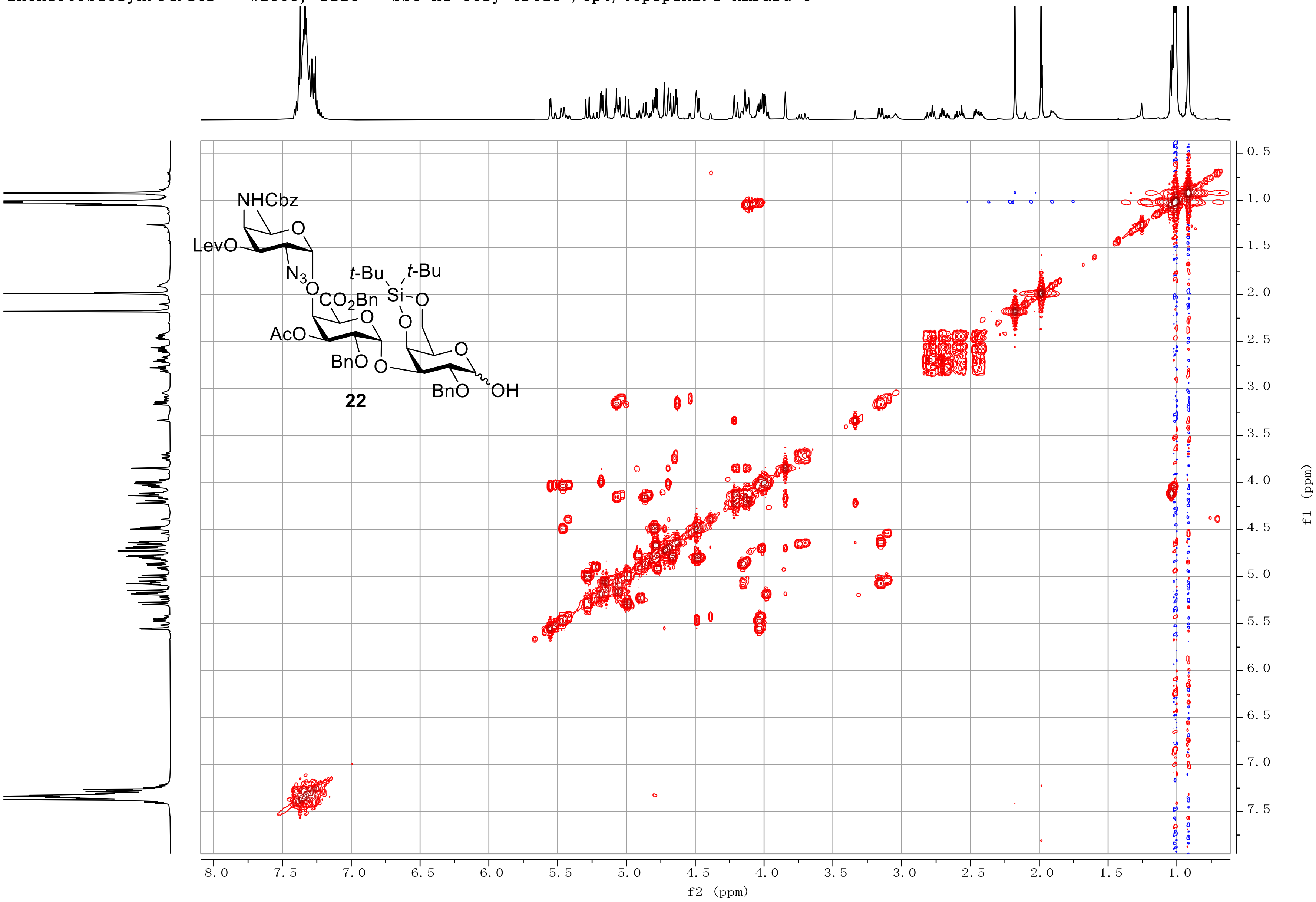

zhen1909biosyn.35.ser — wz503, size — bbo-c13-HSQC CDCl3 /opt/topspin2.1 nmrafd 6

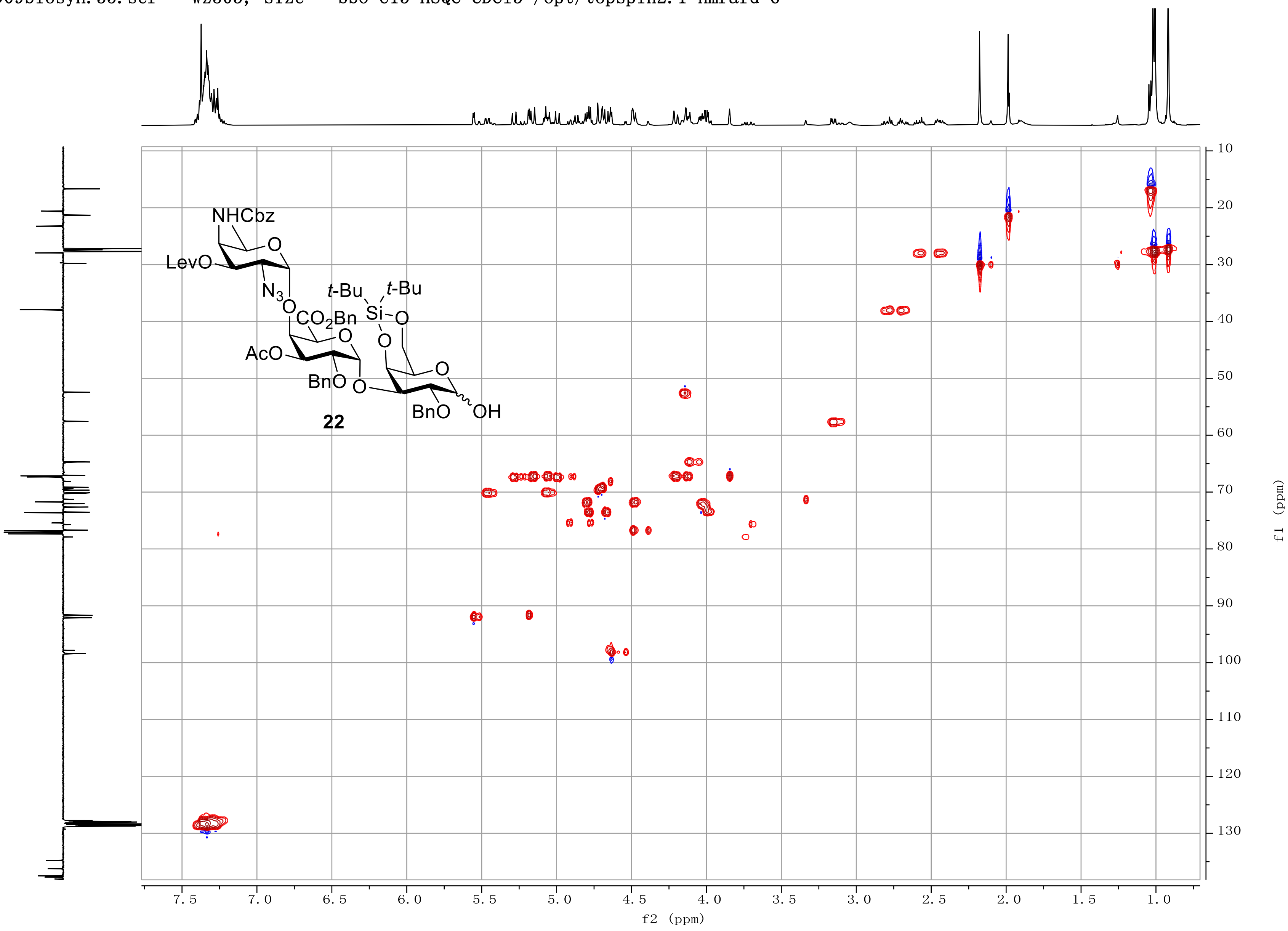

zhen1909biosyn.37.ser — wz503, size — bbo-c13-HMBC CDCl3 /opt/topspin2.1 nmrafd 6

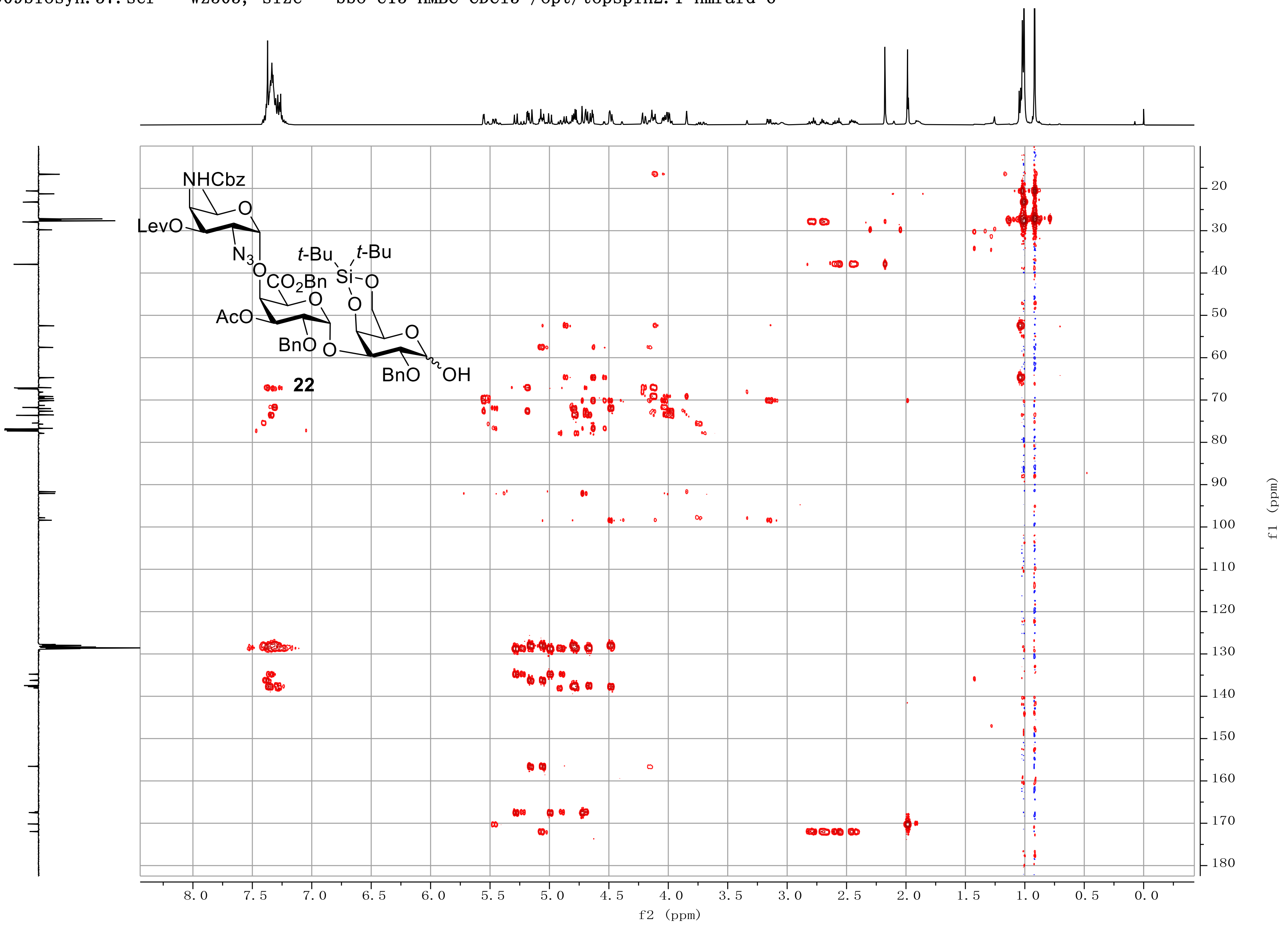

1911zhen.1.fid — wz504-F — h1 Acetone /opt/DATA nmrafd 19

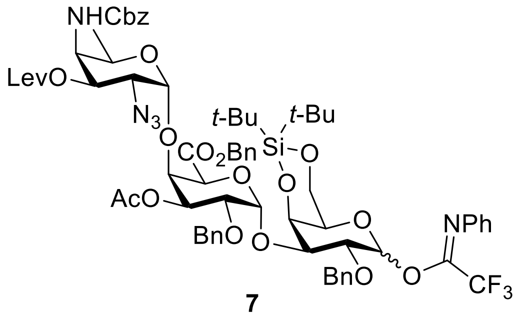

7

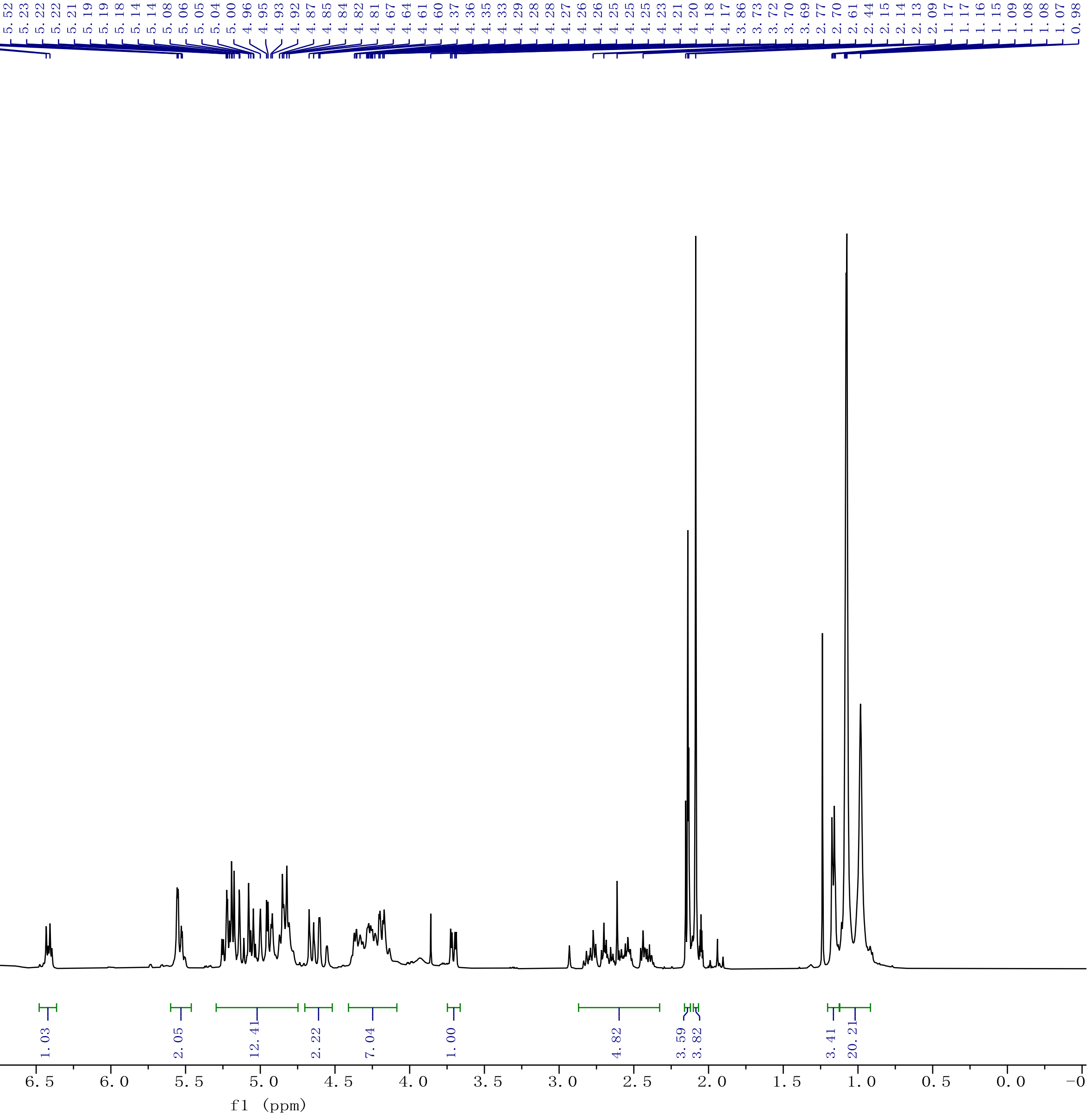

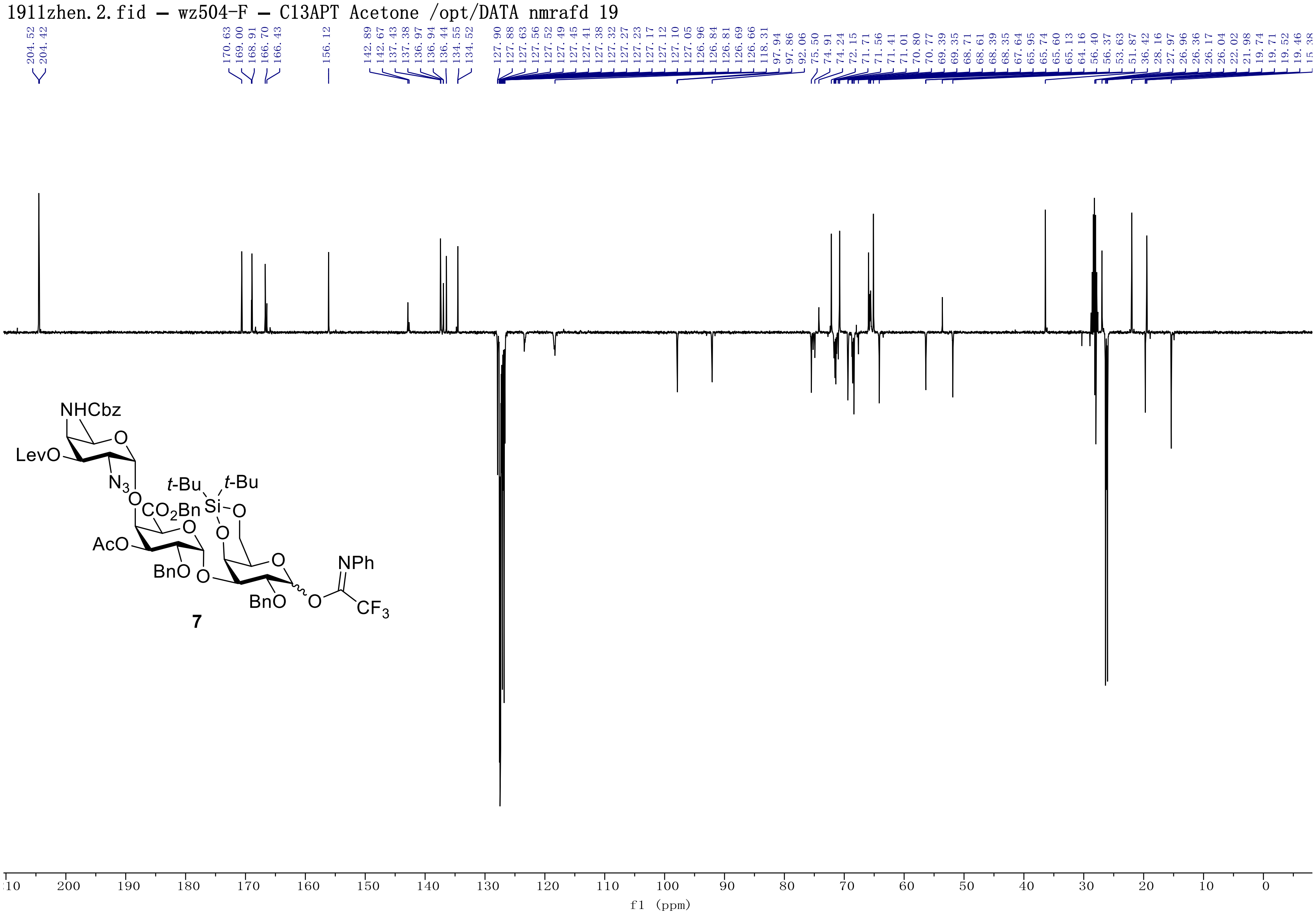
1911zhen.2.fid — wz504-F — C13APT Acetone /opt/DATA nmrafd 19
204.52
204.42
170.63
169.00
168.91
166.70
166.43
156.12
142.89
142.67
137.43
137.38
136.97
136.94
136.44
134.55
134.52
127.90
127.88
127.63
127.56
127.52
127.49
127.45
127.41
127.38
127.32
127.27
127.23
127.17
127.12
127.10
127.05
126.96
126.84
126.81
126.69
126.66
118.31
97.94
97.86
92.06
75.50
74.91
74.24
72.15
71.71
71.56
71.41
71.01
70.80
70.77
69.39
69.35
68.71
68.61
68.39
68.35
67.64
65.95
65.74
65.60
65.13
64.16
56.40
56.37
53.63
51.87
36.42
28.16
27.97
26.96
26.36
26.17
26.04
22.02
21.98
19.74
19.71
19.52
19.46
NHCbz
LevO
O
N_3
t-Bu
t-Bu
Si
CO_2Bn
AcO
BnO
NPh
CF_3
7
200
190
180
170
160
150
140
130
120
110
100
90
80
70
60
50
40
30
20
10
0
f1 (ppm)

1911zhen.3.ser — wz504-F — h1COSY Acetone /opt/DATA nmrafd 19

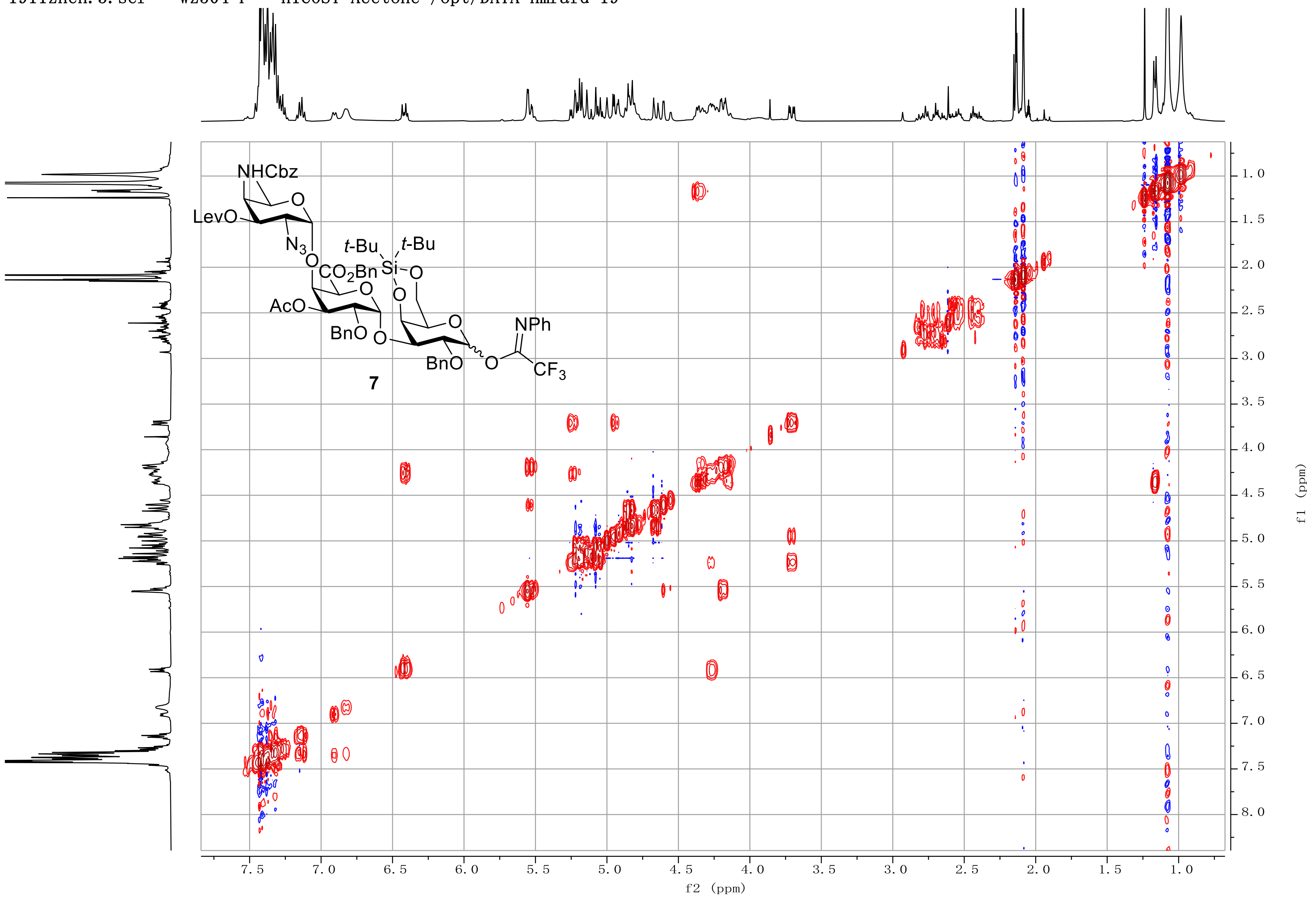

1911zhen.4.ser — wz504-F — c13HSQC Acetone /opt/DATA nmrafd 19

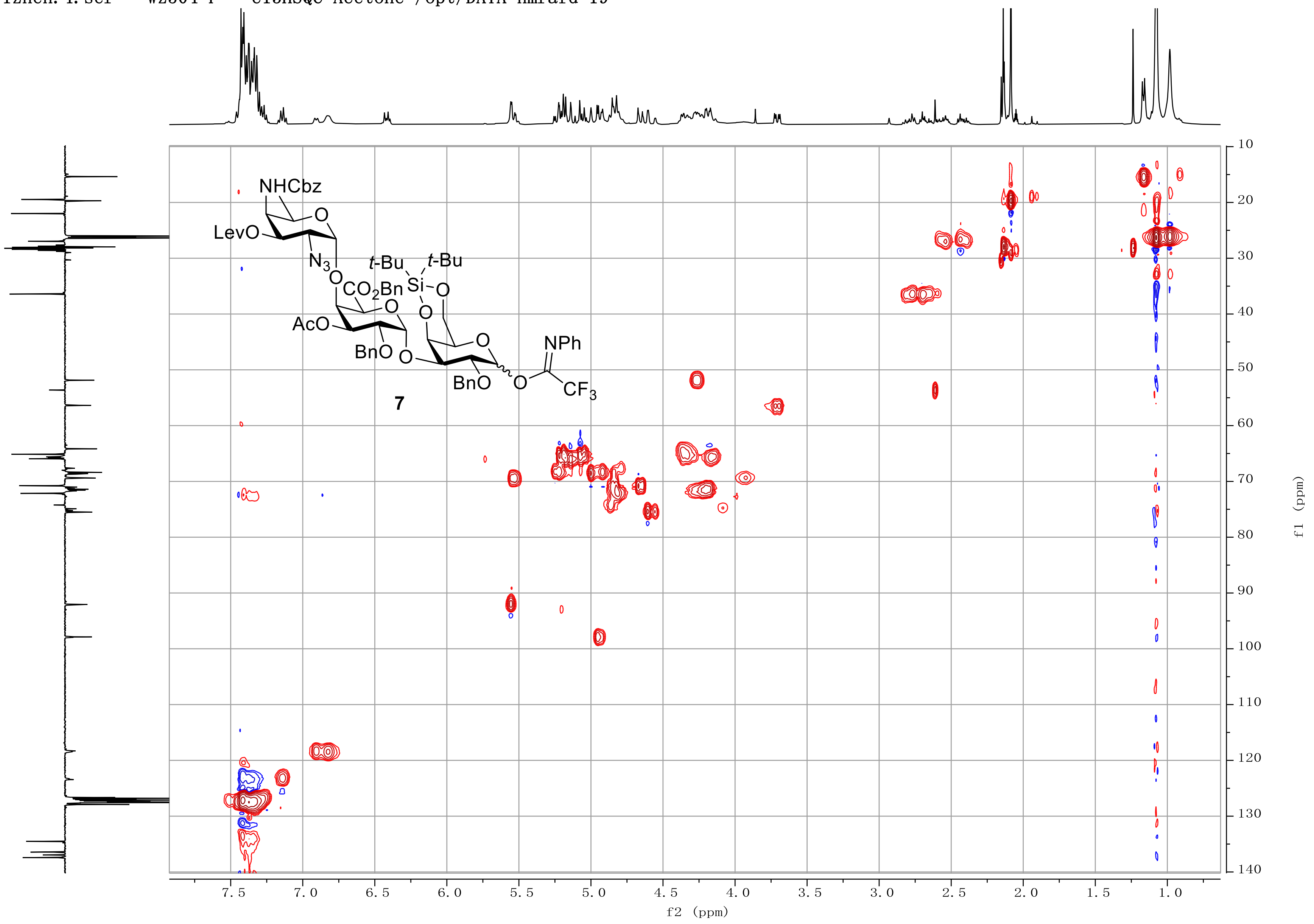

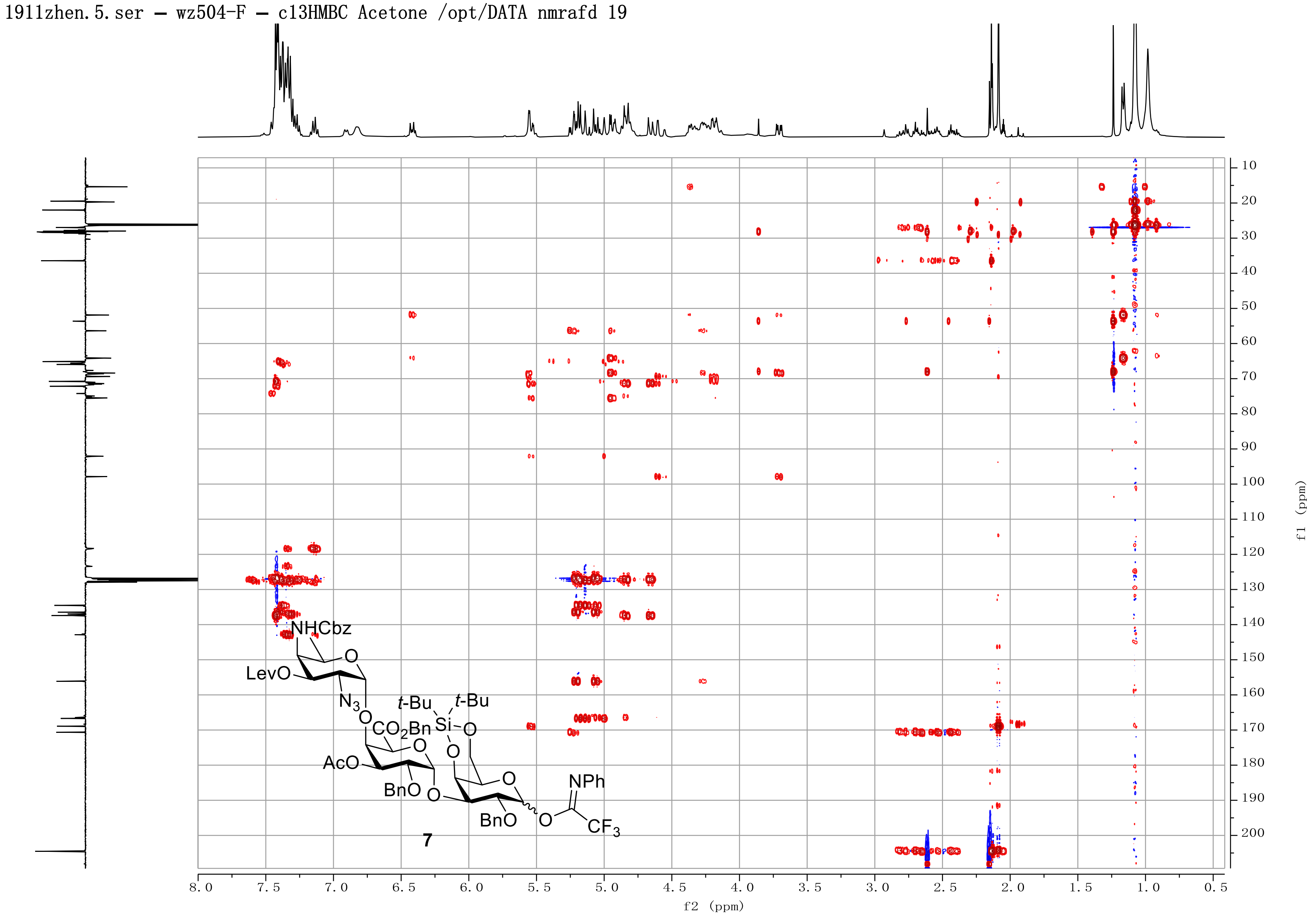
1911zhen.5.ser — wz504-F — c13HMBC Acetone /opt/DATA nmrafd 19
NHCbz
LevO
O
N3
t-Bu
t-Bu
Si
O
CO2Bn
AcO
BnO
O
BnO
NPh
CF3
7
f2 (ppm)
f1 (ppm)
8.0
7.5
7.0
6.5
6.0
5.5
5.0
4.5
4.0
3.5
3.0
2.5
2.0
1.5
1.0
0.5
10
20
30
40
50
60
70
80
90
100
110
120
130
140
150
160
170
180
190
200

zhen0318.9.fid — wz461; — 1H, bbi-av400

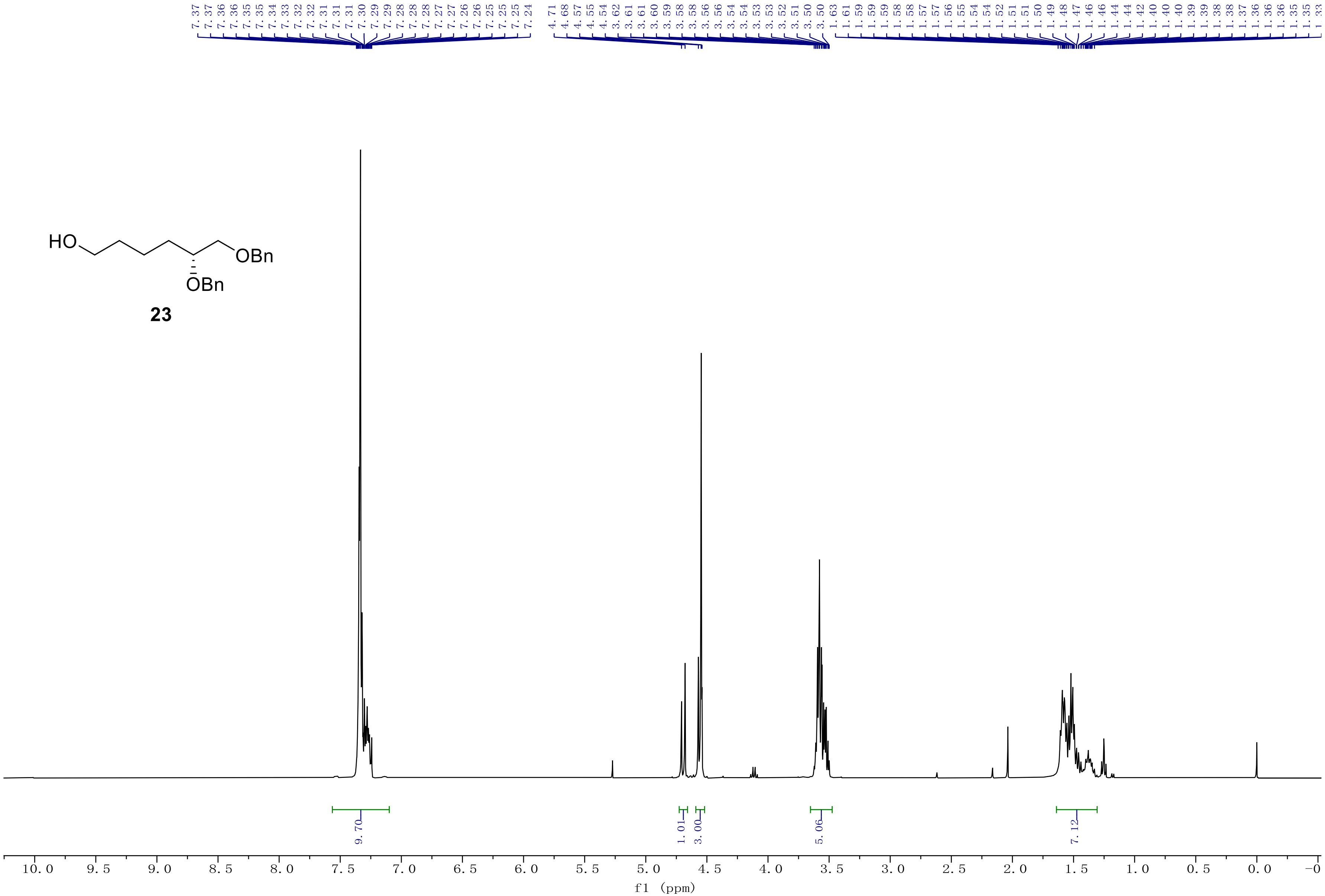

23

zhen0318.11.fid — wz461; — 13C-APT, av400-bbi, ns=500

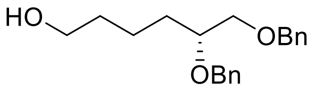

23

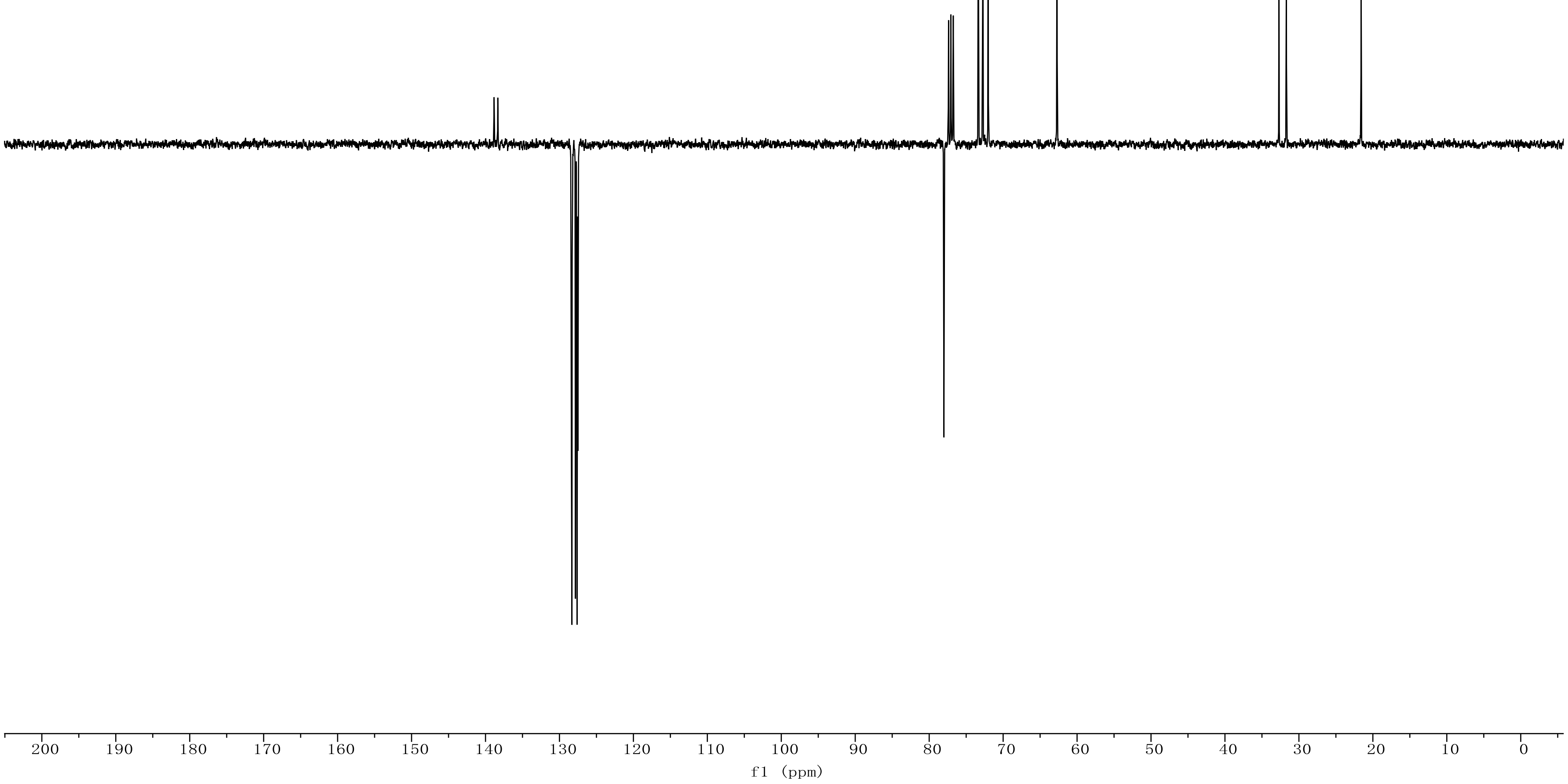

zhen0318.13.ser — wz461; — BBI-1H-COSY

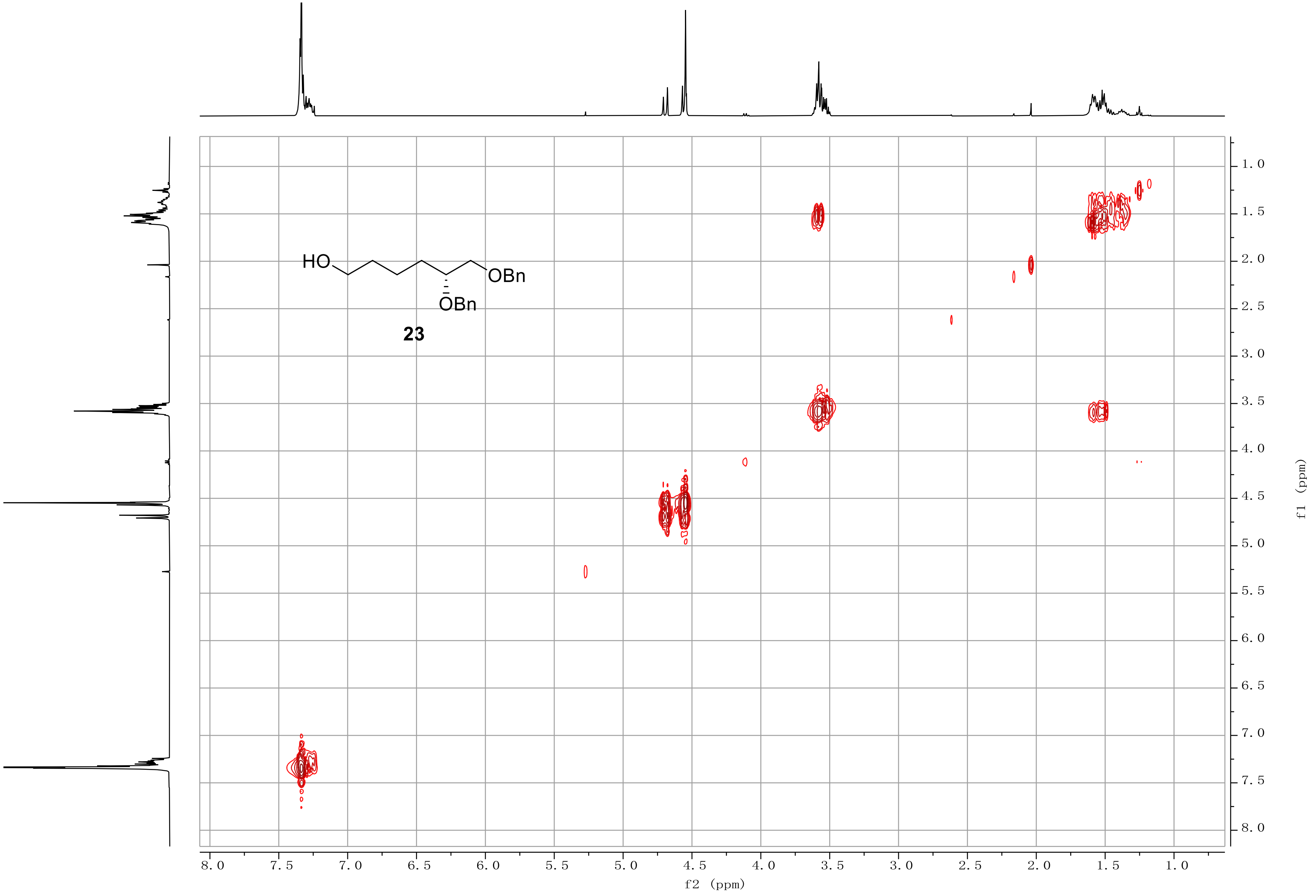

zhen0318.12.ser — wz461; — hsqc, av400, bbi,

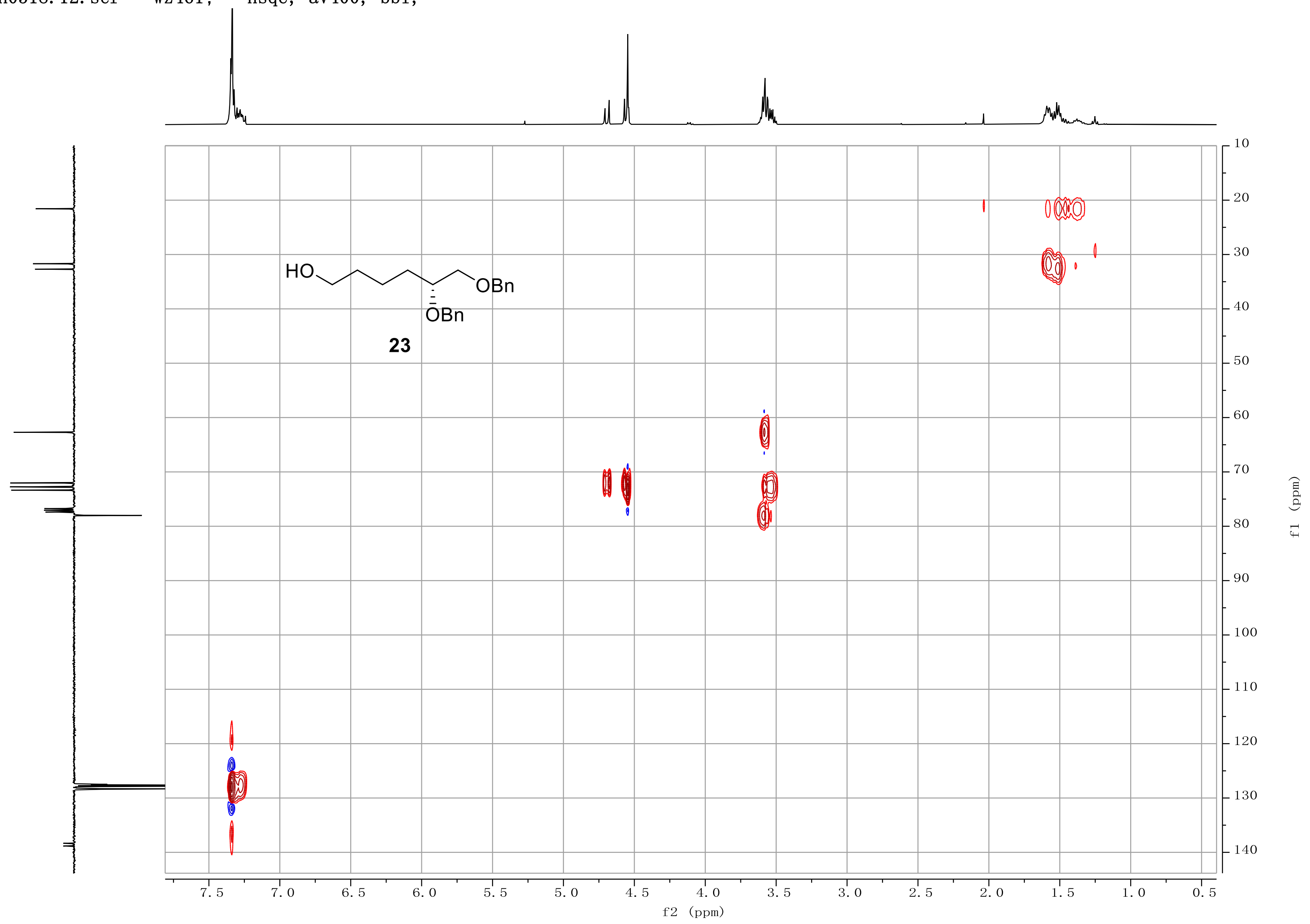

zhen1808biosyn.77.fid — wz506 — bbo-h1 CDCl3 /opt/topspin2.1 nmrafd 4

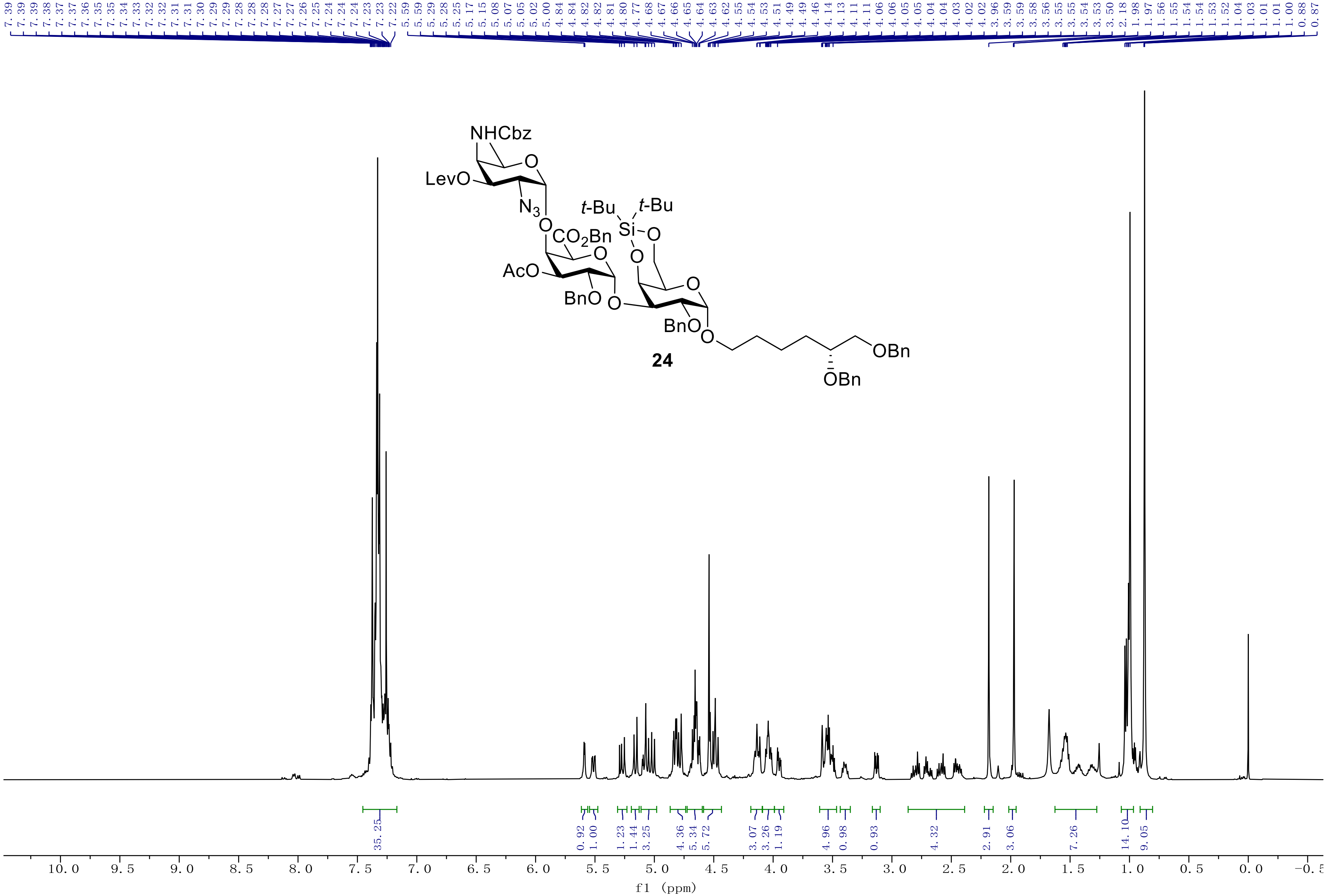

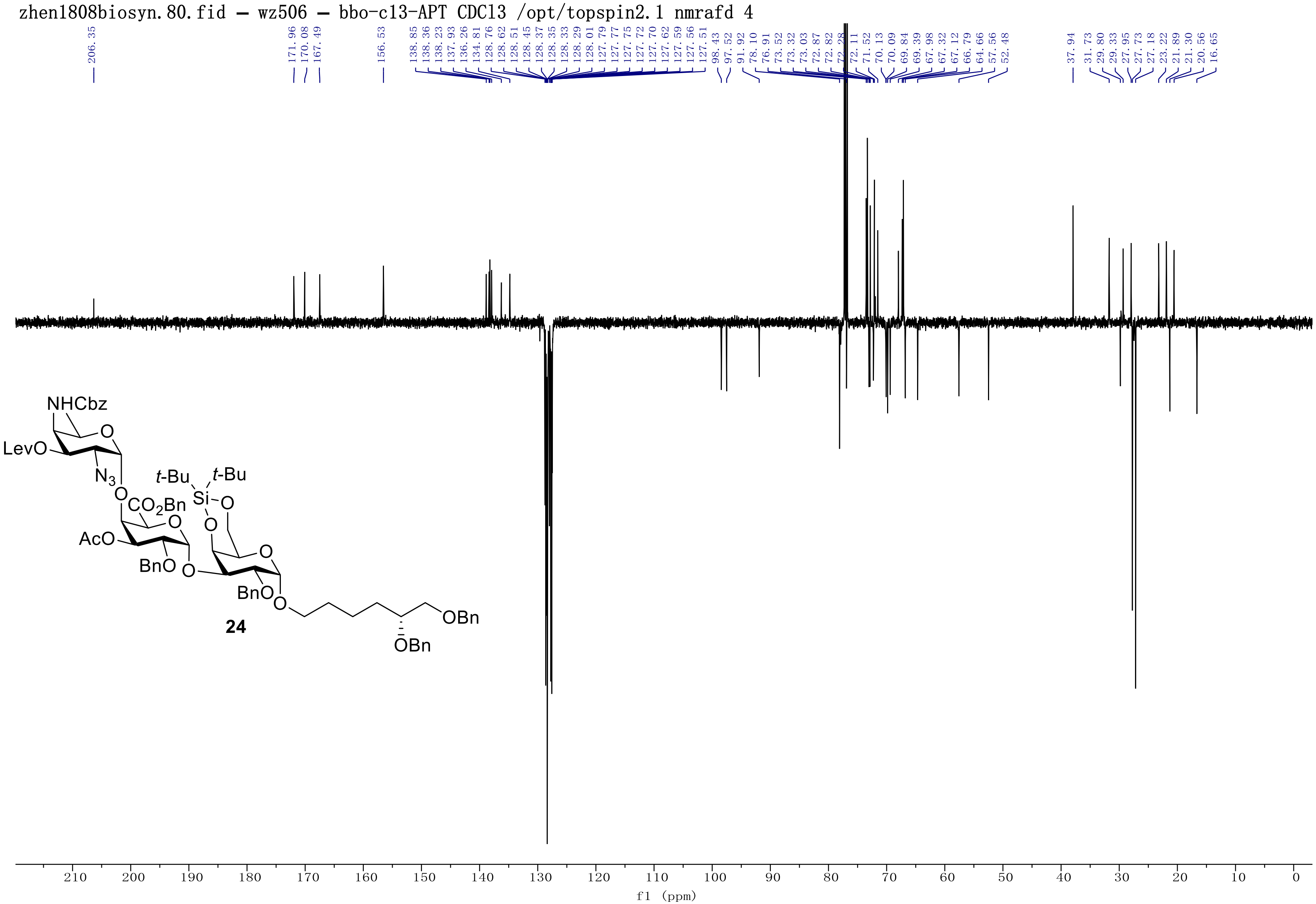
zhen1808biosyn.80.fid — wz506 — bbo-c13-APT CDCl3 /opt/topspin2.1 nmrafd 4
206.35
171.96
170.08
167.49
156.53
138.85
138.36
138.23
137.93
136.26
134.81
128.76
128.62
128.51
128.45
128.37
128.35
128.33
128.29
128.01
127.79
127.77
127.75
127.72
127.70
127.62
127.59
127.56
127.51
98.43
97.52
91.92
78.10
76.91
73.52
73.32
73.03
72.87
72.82
72.28
72.11
71.52
70.13
70.09
69.84
69.39
67.98
67.32
67.12
66.79
64.66
57.56
52.48
37.94
31.73
29.80
29.33
27.95
27.73
27.18
23.22
21.89
21.30
20.56
16.65
NHCbz
LevO
N3
O
t-Bu
t-Bu
Si
CO2Bn
AcO
BnO
BnO
OBn
OBn
24
210 200 190 180 170 160 150 140 130 120 110 100 90 80 70 60 50 40 30 20 10 0
f1 (ppm)

zhen1808biosyn.78.ser — wz506 — bbo-h1-cosy CDCl3 /opt/topspin2.1 nmrafd 4

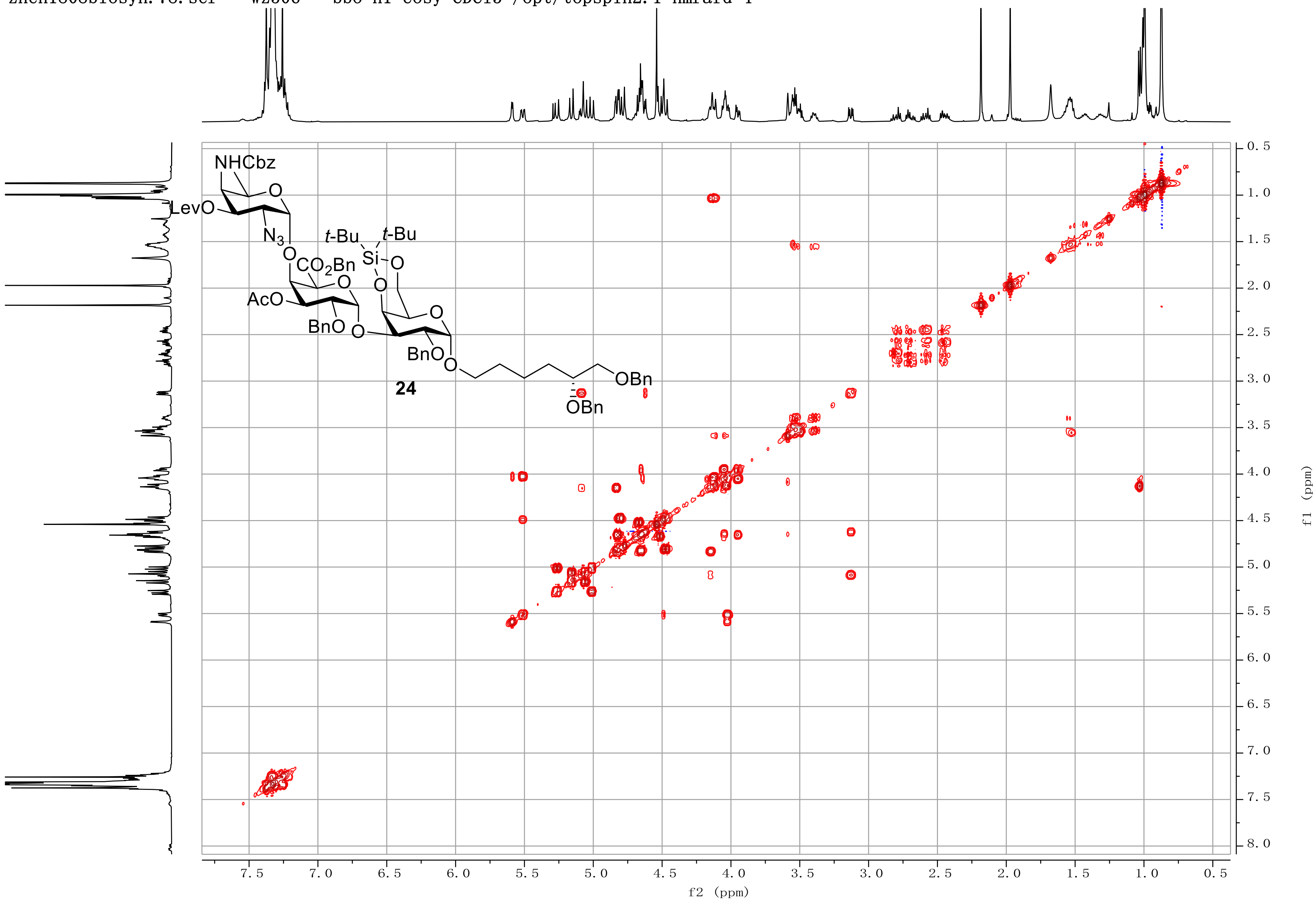

zhen1808biosyn.79.ser — wz506 — bbo-c13-HSQC CDCl3 /opt/topspin2.1 nmrafd 4

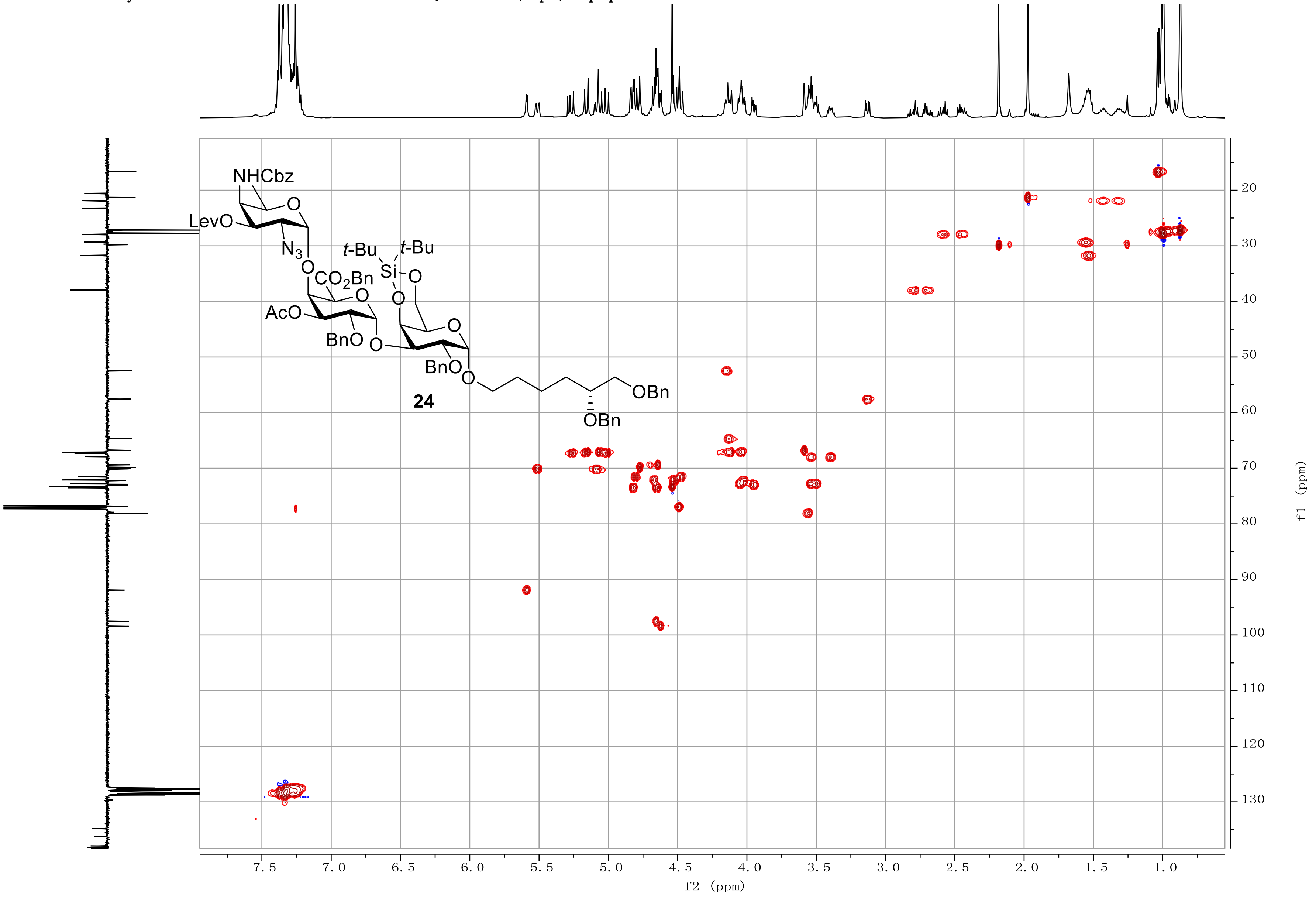

zhen1808biosyn.81.ser — wz506 — bbo-c13-HMBC CDCl3 /opt/topspin2.1 nmrafd 4

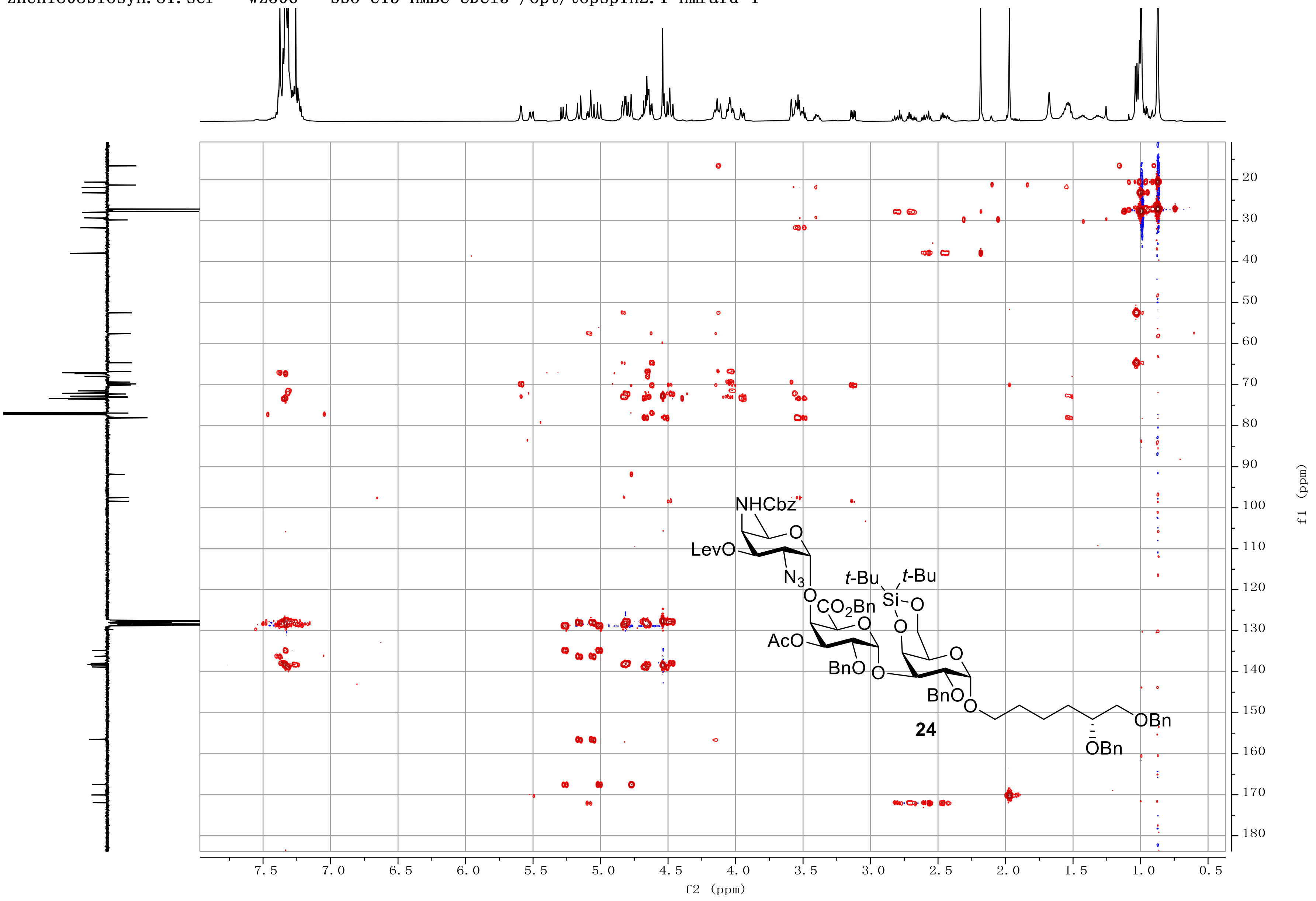

1810zhen.18.fid — wz513-1, 300mg — h1 CDCl3 /opt/DATA nmrafd 15

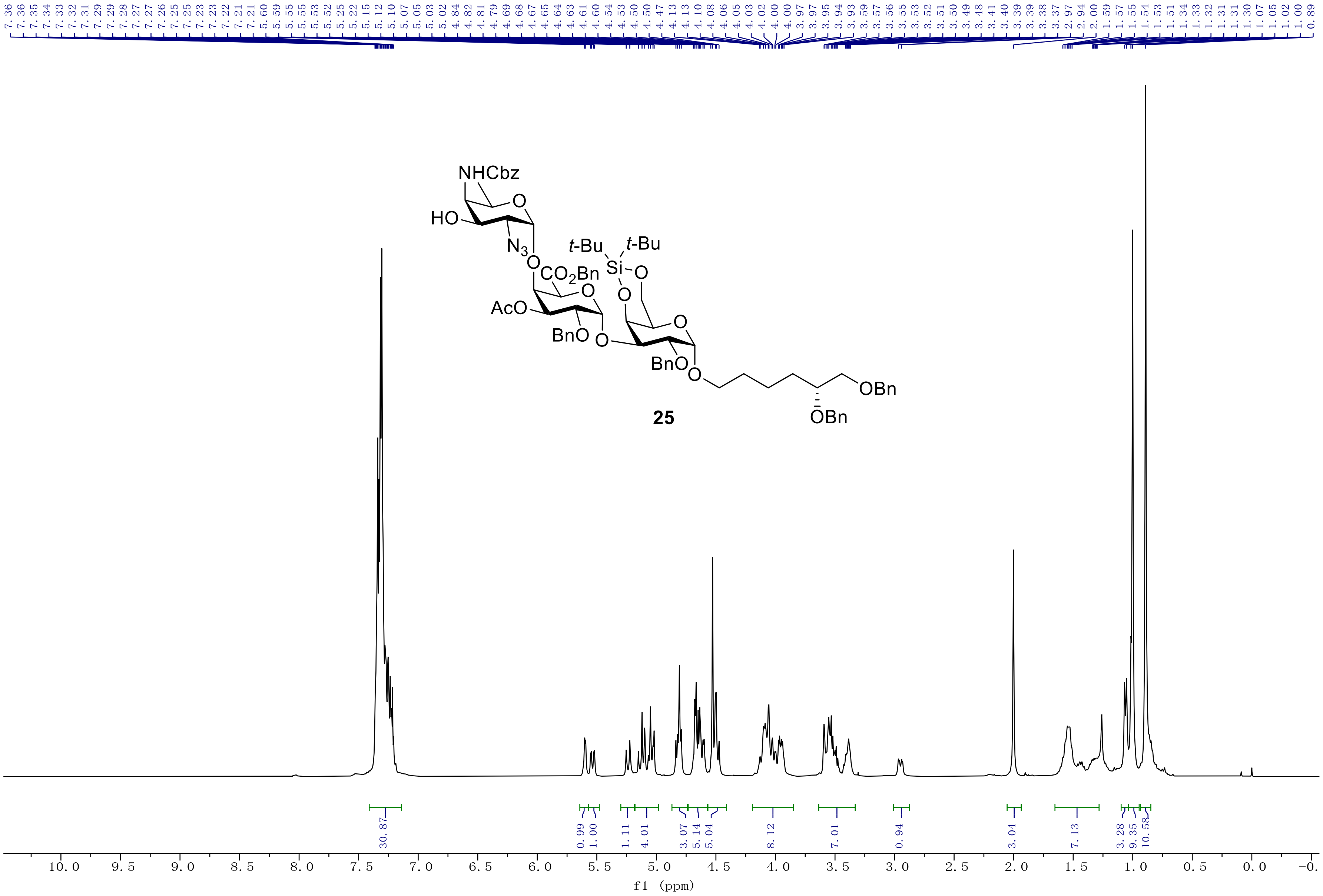

1810zhen.19.fid — wz513-1, 300mg — C13APT CDCl3 /opt/DATA nmrafd 15

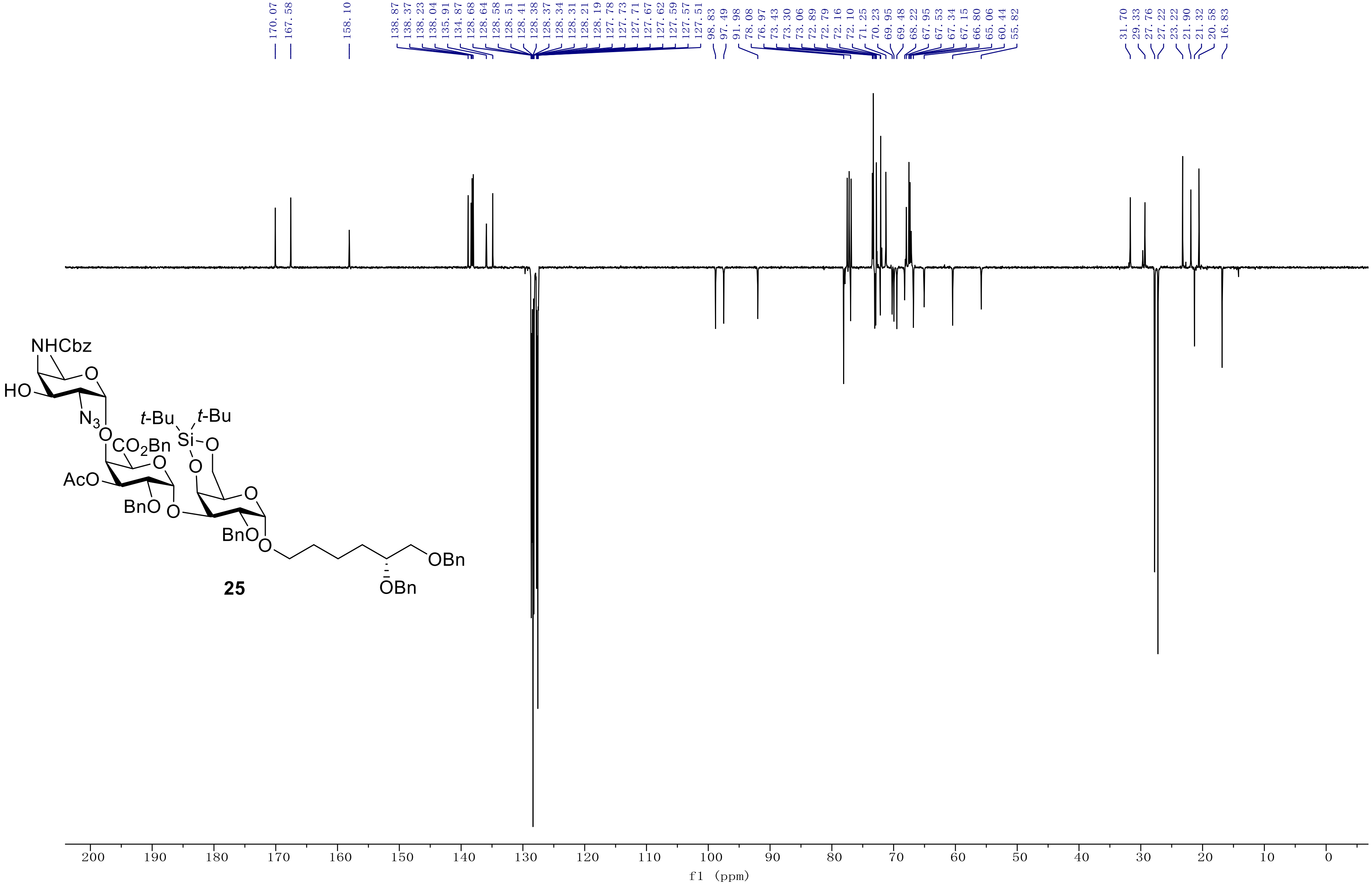

1810zhen.20.ser — wz513-1, 300mg — h1COSY CDCl3 /opt/DATA nmrafd 15

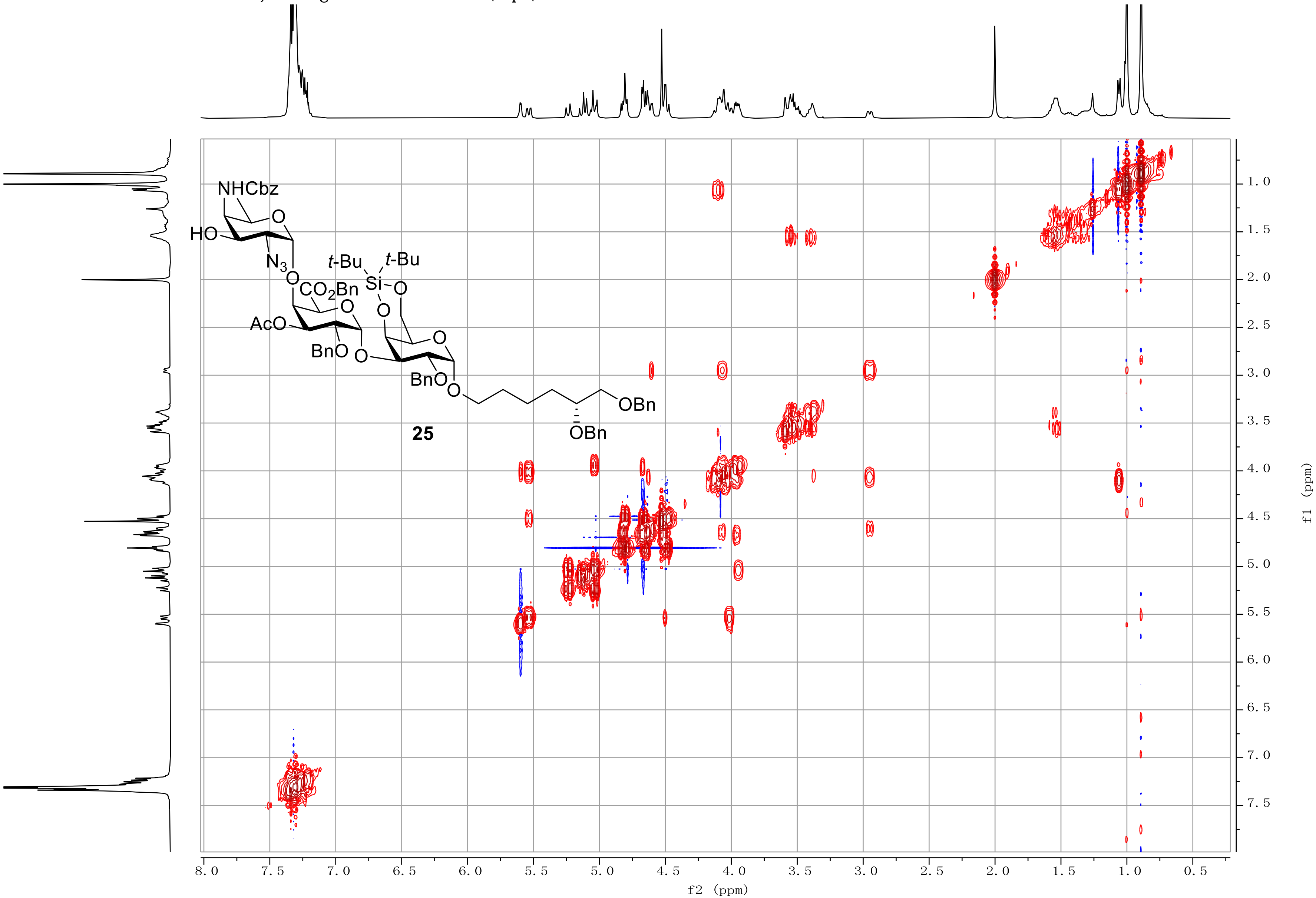

1810zhen.21.ser — wz513-1, 300mg — c13HSQC CDCl3 /opt/DATA nmrafd 15

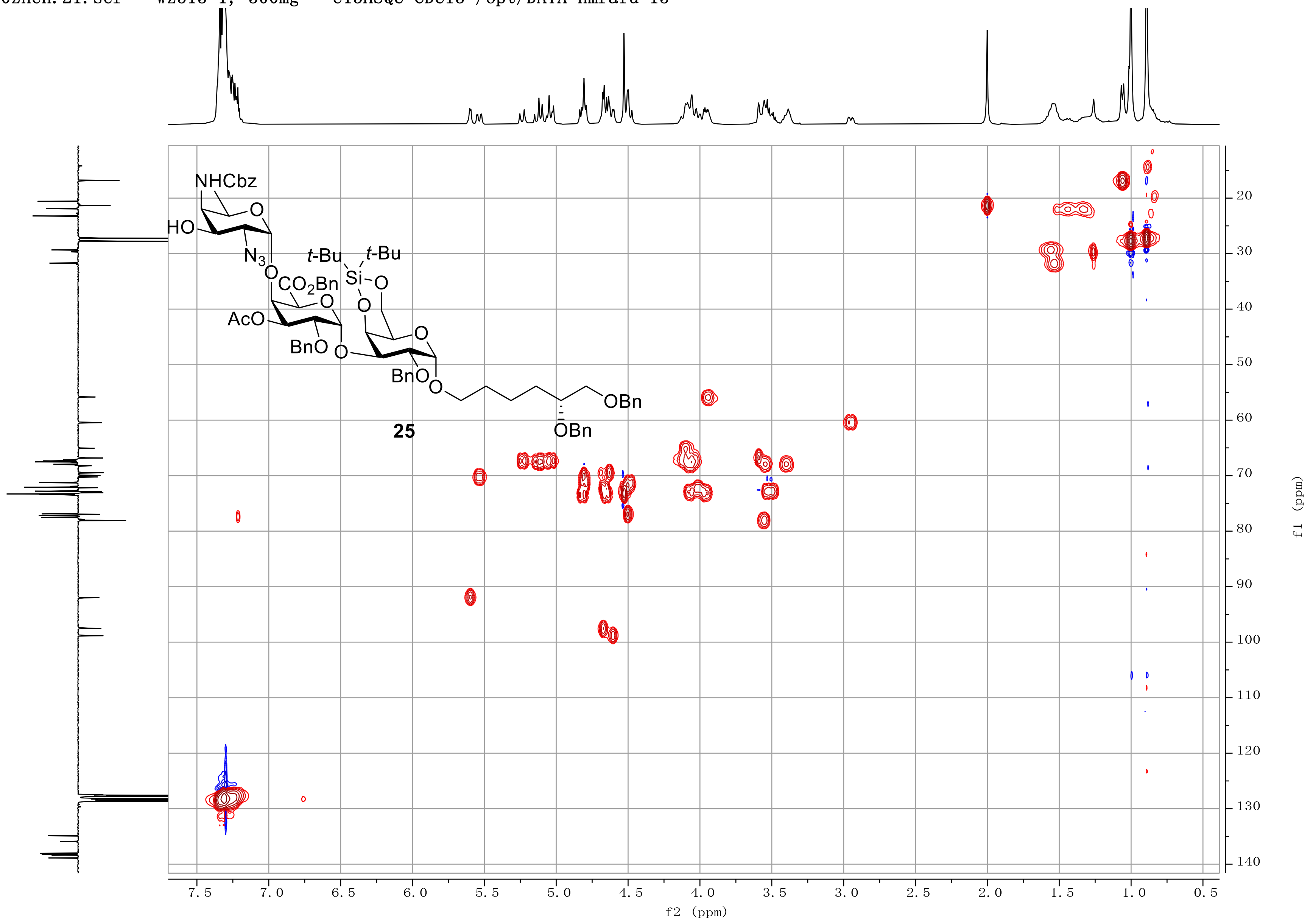

1810zhen.22.ser — wz513-1, 300mg — c13HMBC CDCl3 /opt/DATA nmrafd 15

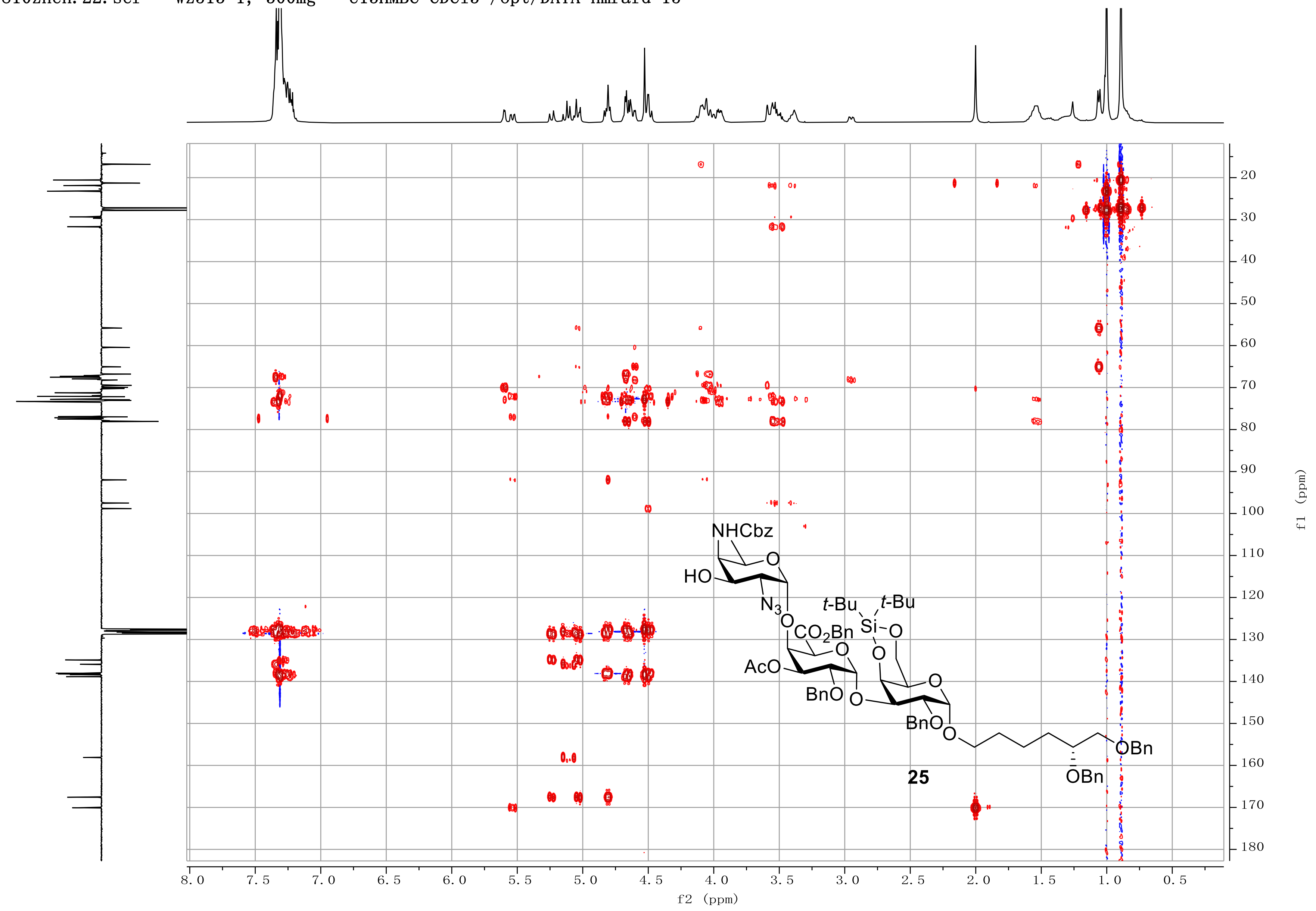

1810zhen.23.ser — wz513-1, 300mg — c13HMBCipvGATED CDCl3 /opt/DATA nmrafd 15

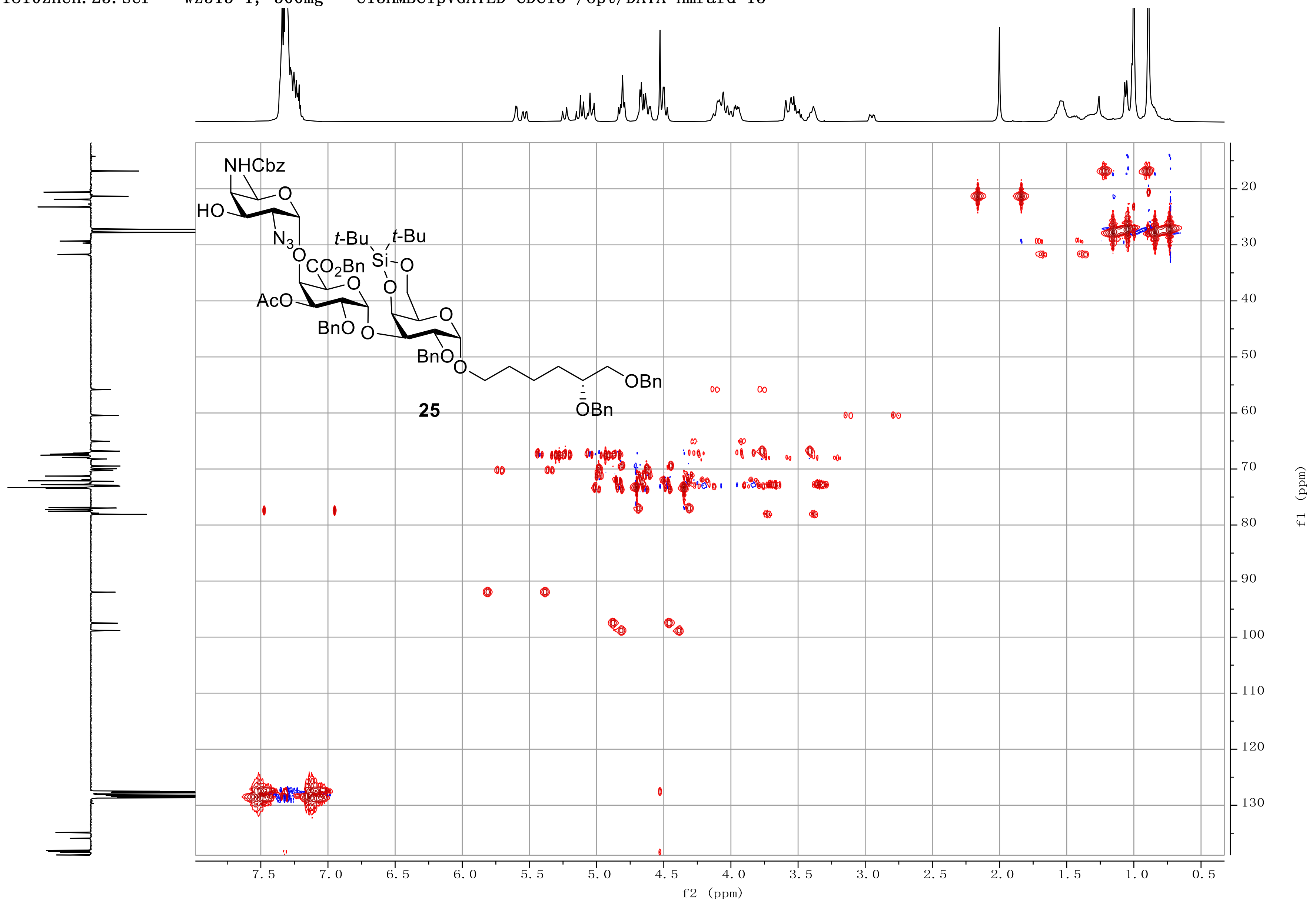

zhen1901biosyn.34.fid — wz514-4, 100mg — bbo-h1 CDCl3 /opt/topspin2.1 nmrafd 10

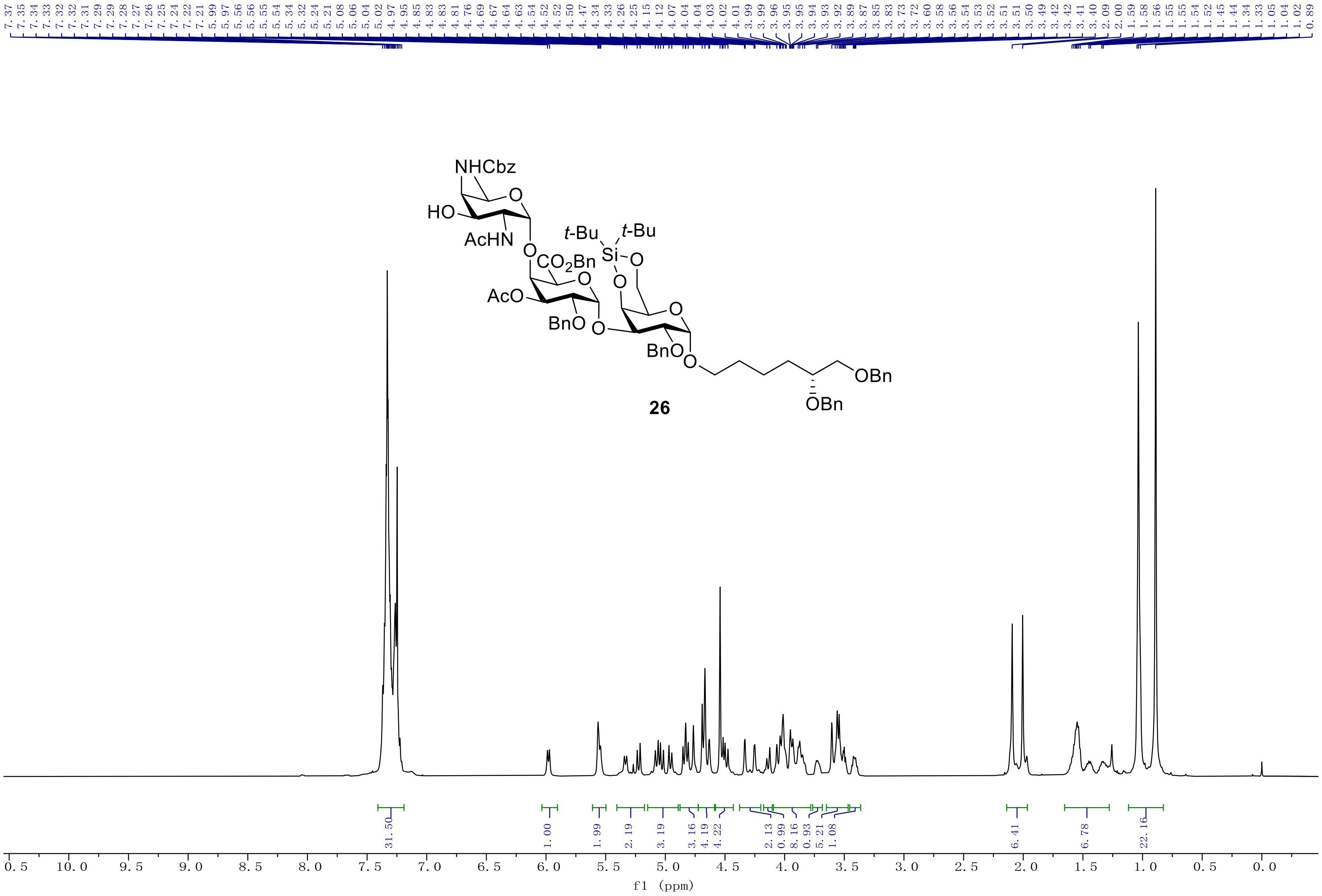

zhen1901biosyn.37.fid — wz514-4, 100mg — bbo-c13-APT CDCl3 /opt/topspin2.1 nmrafd 10

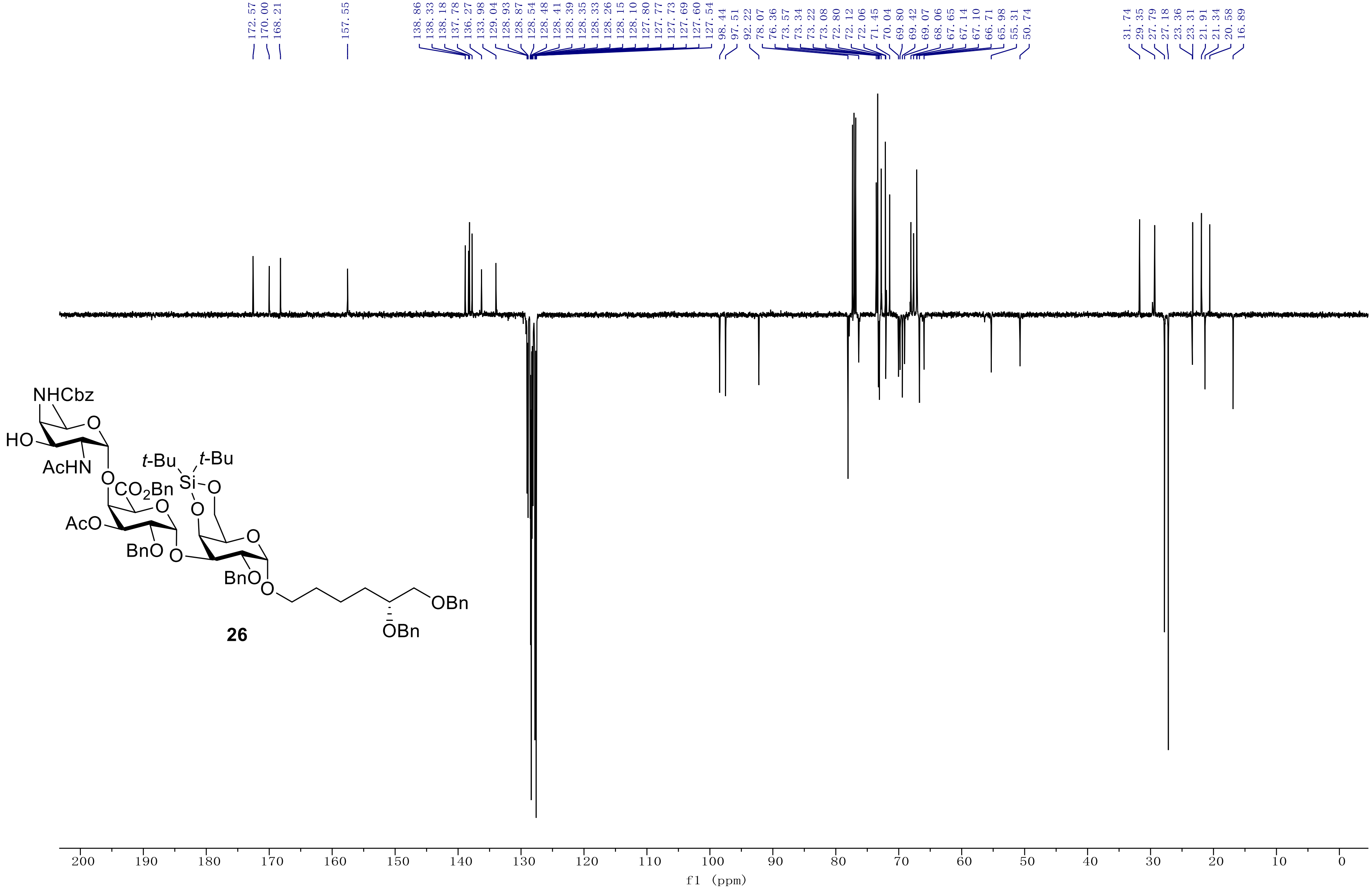

zhen1901biosyn.35.ser — wz514-4, 100mg — bbo-h1-cosy CDCl3 /opt/topspin2.1 nmrafd 10

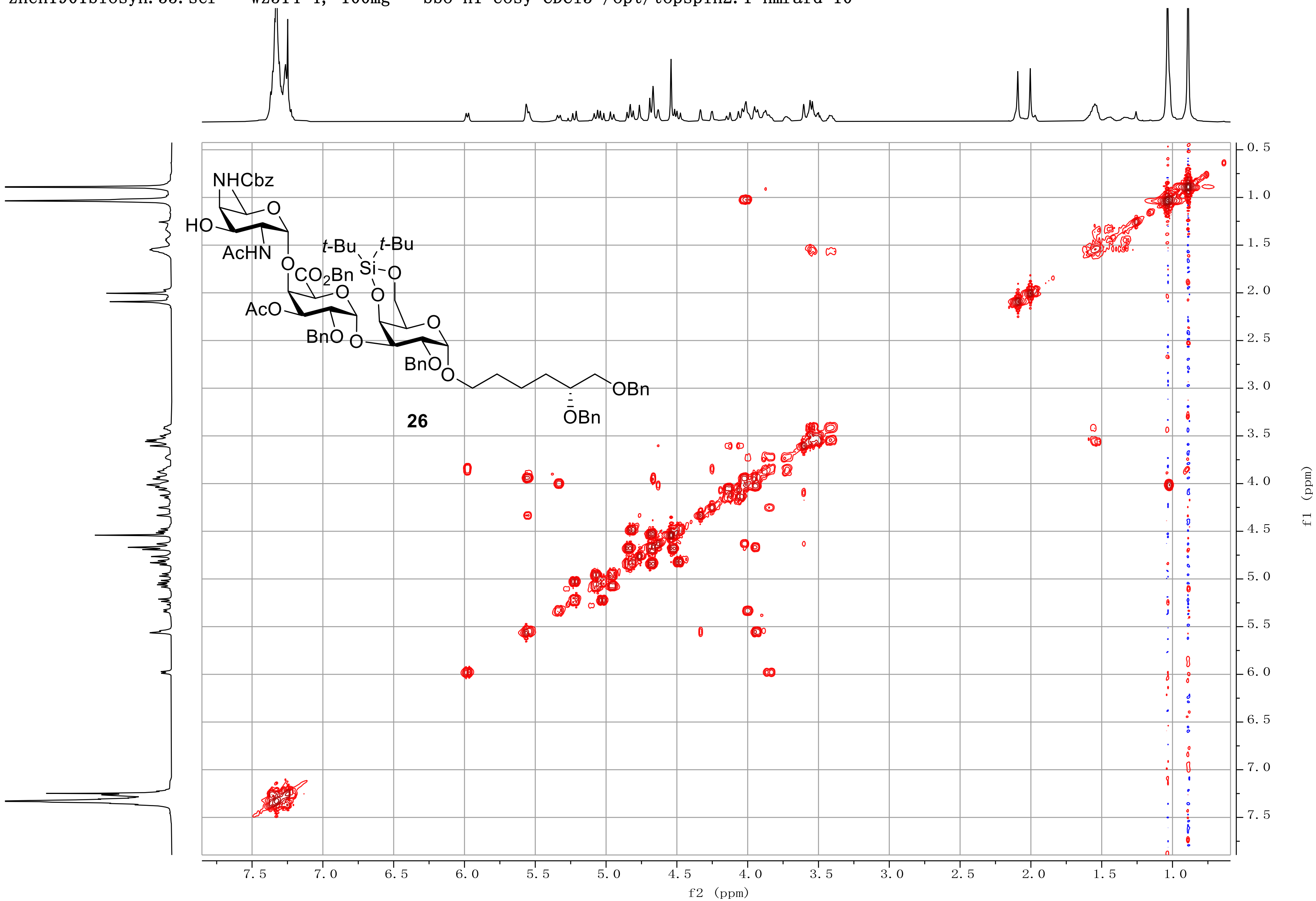

zhen1901biosyn.36.ser — wz514-4, 100mg — bbo-c13-HSQC CDCl3 /opt/topspin2.1 nmrafd 10

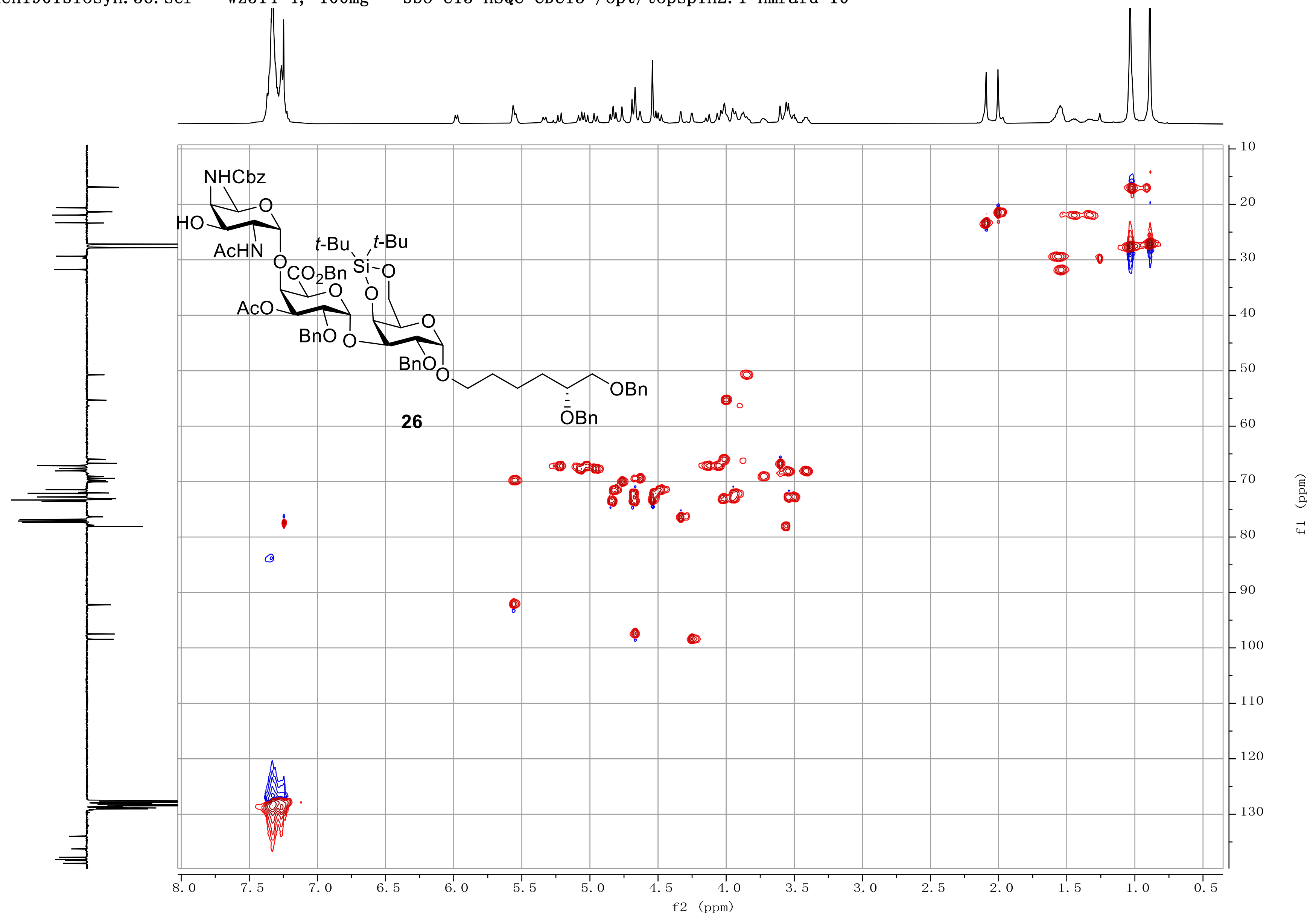

zhen1901biosyn.38.ser — wz514-4, 100mg — bbo-c13-HMBC CDCl3 /opt/topspin2.1 nmrafd 10

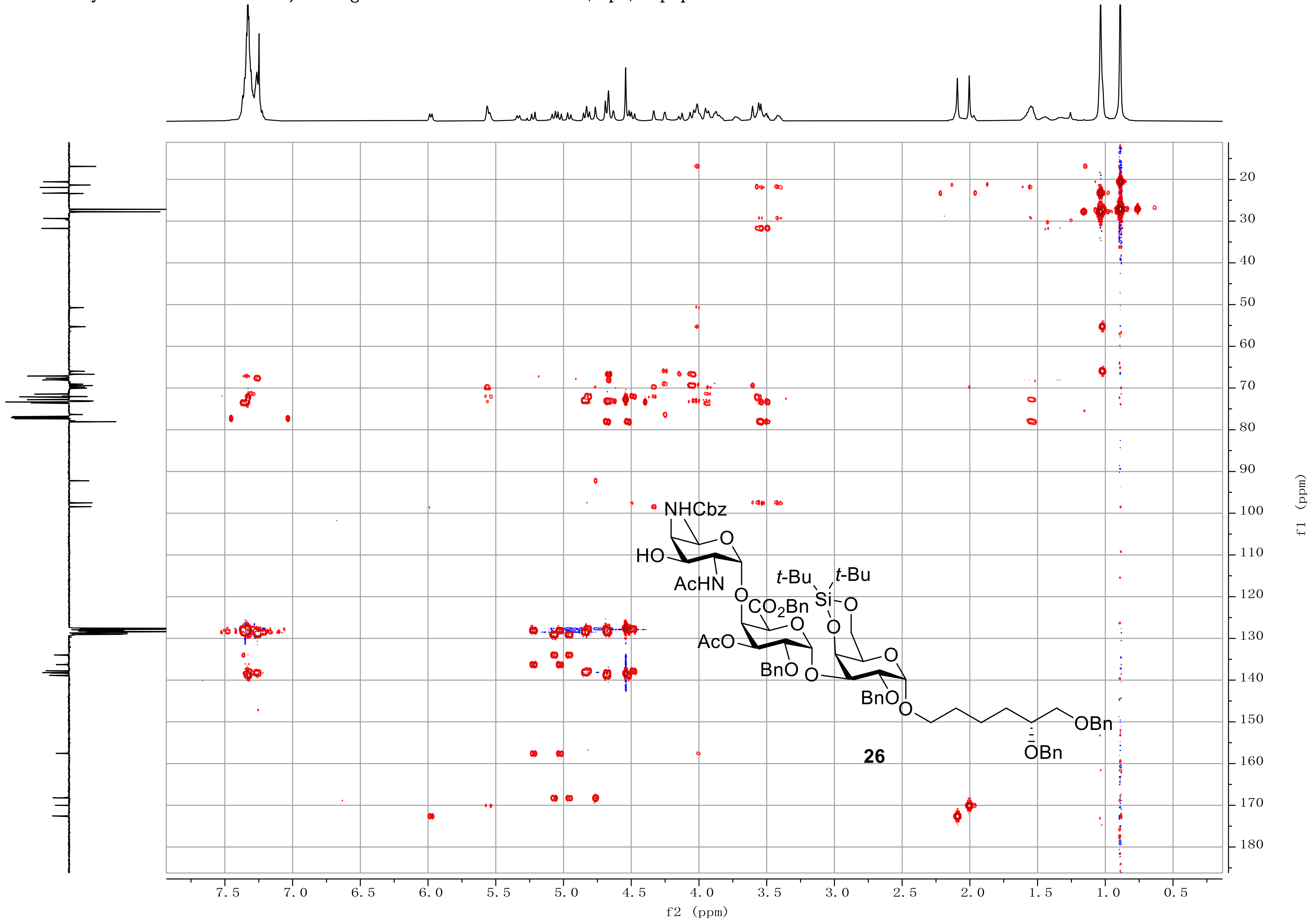

zhen1901biosyn.39.ser — wz514-4, 100mg — bbo-c13-hmbc-ipv-gated CDCl3 /opt/topspin2.1 nmrafd 10

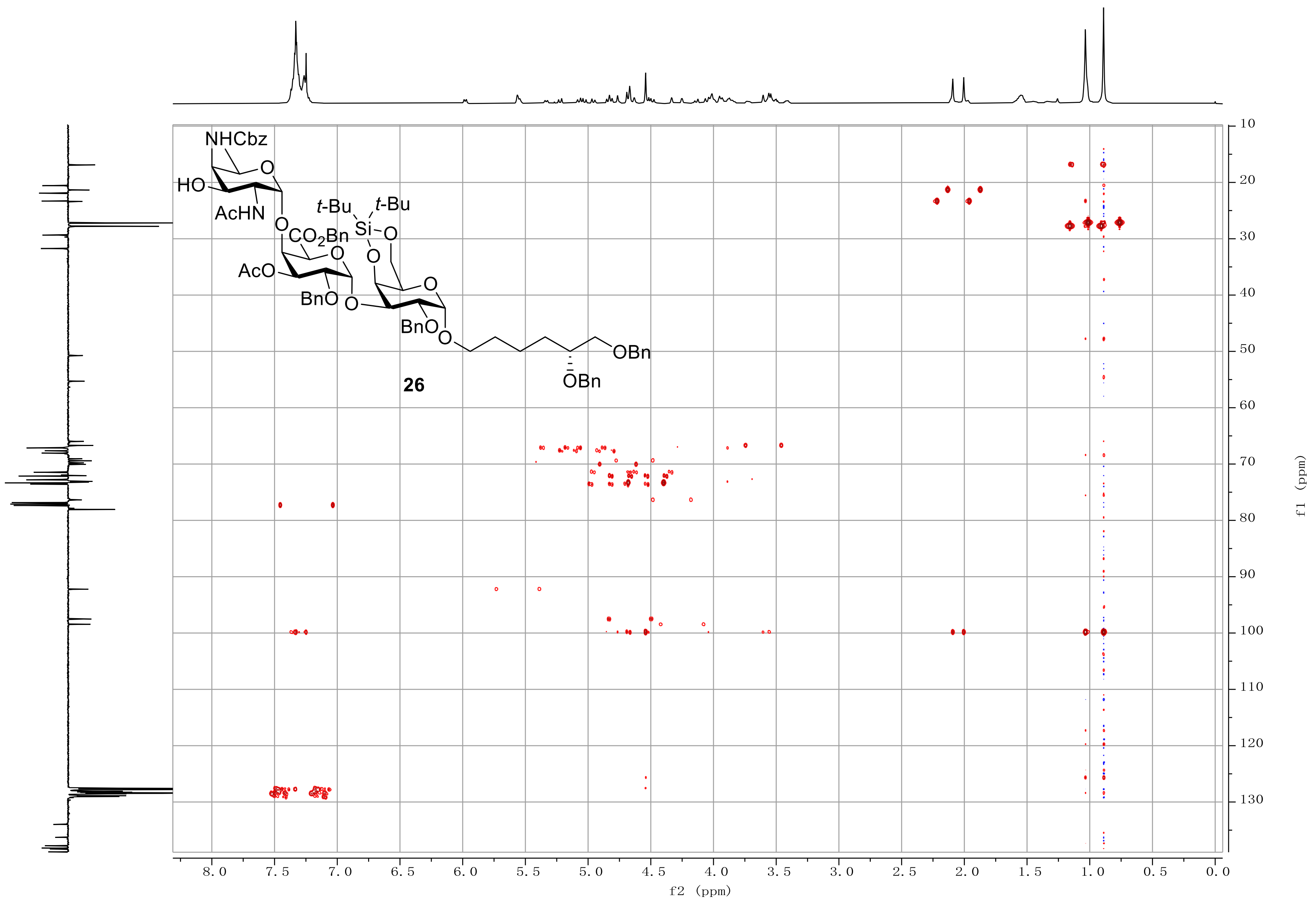

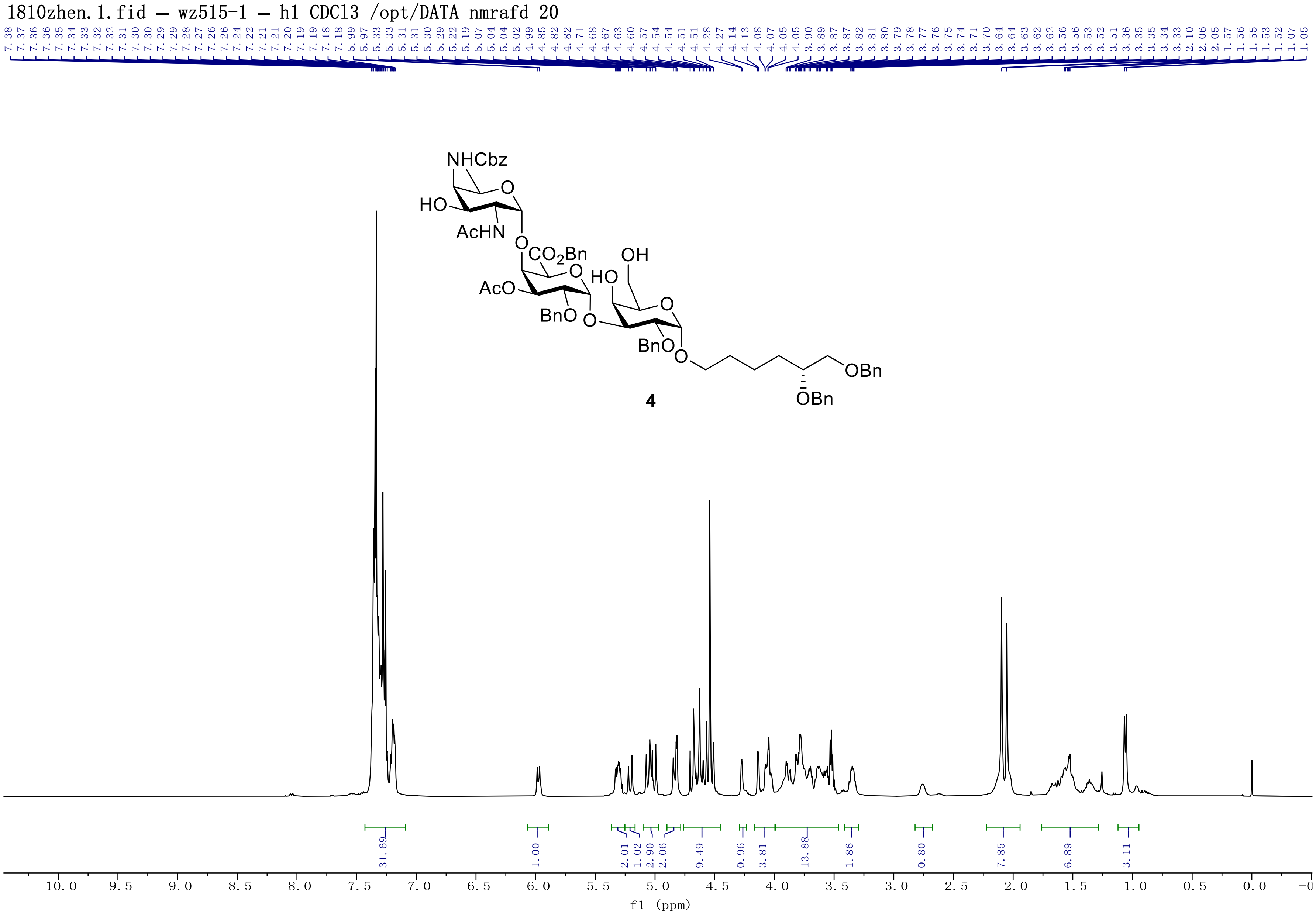
1810zhen.1.fid — wz515-1 — h1 CDCl3 /opt/DATA nmrafd 20
7.38 7.37 7.36 7.36 7.35 7.34 7.33 7.32 7.32 7.31 7.30 7.30 7.29 7.29 7.28 7.27 7.26 7.26 7.24 7.22 7.21 7.21 7.20 7.19 7.19 7.18 7.18 5.99 5.97 5.33 5.33 5.31 5.31 5.30 5.29 5.22 5.19 5.07 5.04 5.04 5.02 4.99 4.85 4.82 4.82 4.71 4.68 4.67 4.63 4.60 4.57 4.54 4.54 4.54 4.51 4.28 4.27 4.14 4.13 4.08 4.07 4.05 4.05 3.90 3.89 3.87 3.82 3.81 3.80 3.79 3.78 3.77 3.76 3.75 3.74 3.71 3.70 3.64 3.63 3.62 3.56 3.56 3.53 3.52 3.51 3.36 3.35 3.34 3.33 2.10 2.06 2.05 1.57 1.56 1.55 1.53 1.52 1.07 1.05
NHCbz
HO
AcHN
O
CO2Bn
AcO
BnO
OH
HO
BnO
OBn
OBn
4
31.69
1.00
2.01
1.02
2.90
2.06
9.49
0.96
3.81
13.88
1.86
0.80
7.85
6.89
3.11
10.0 9.5 9.0 8.5 8.0 7.5 7.0 6.5 6.0 5.5 5.0 4.5 4.0 3.5 3.0 2.5 2.0 1.5 1.0 0.5 0.0
f1 (ppm)

1901zhen.27.fid — wz515-2, 80mg — C13APT CDCl3 /opt/DATA nmrafd 15

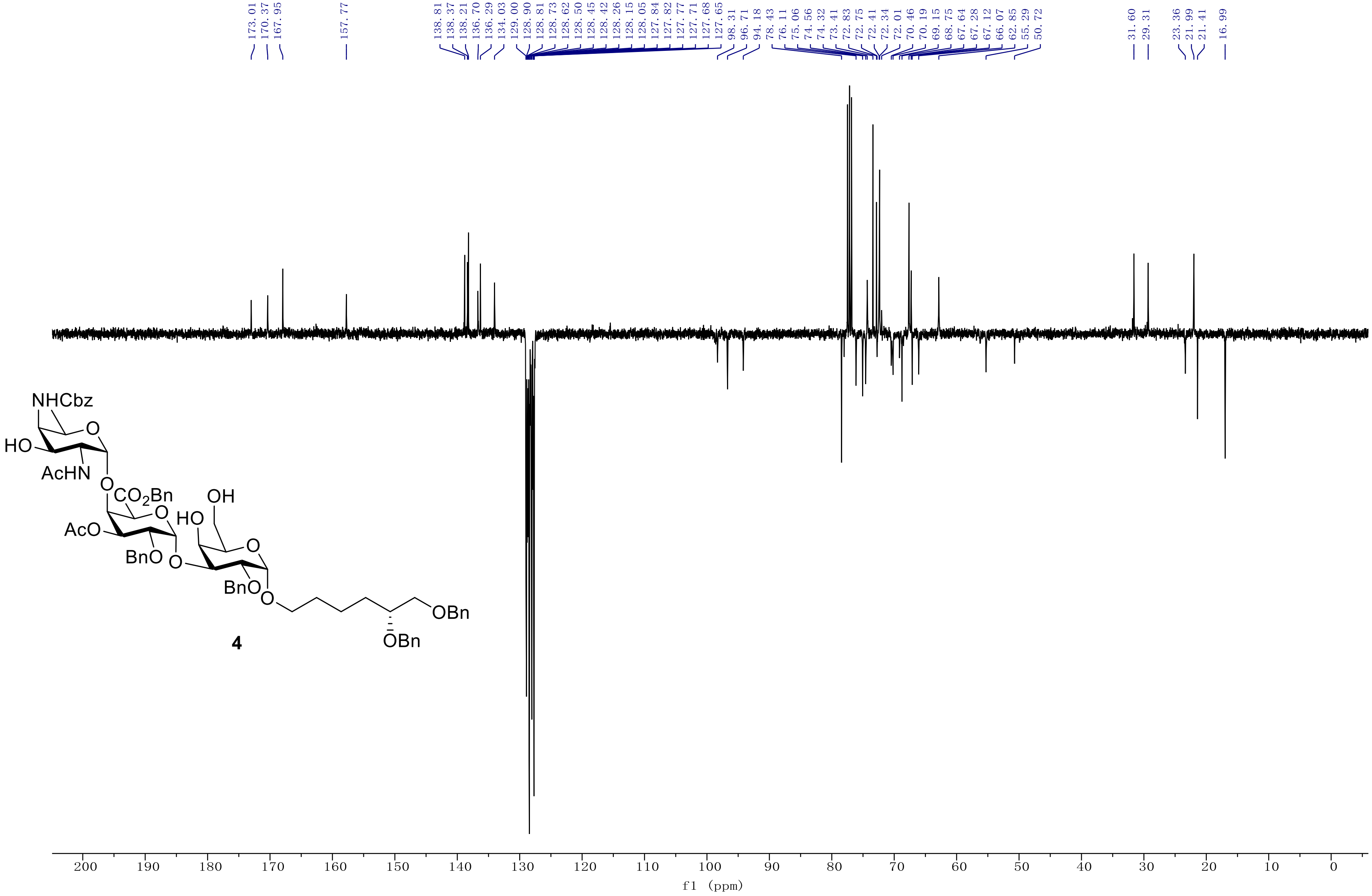

1901zhen.28.ser — wz515-2, 80mg — h1COSY CDCl3 /opt/DATA nmrafd 15

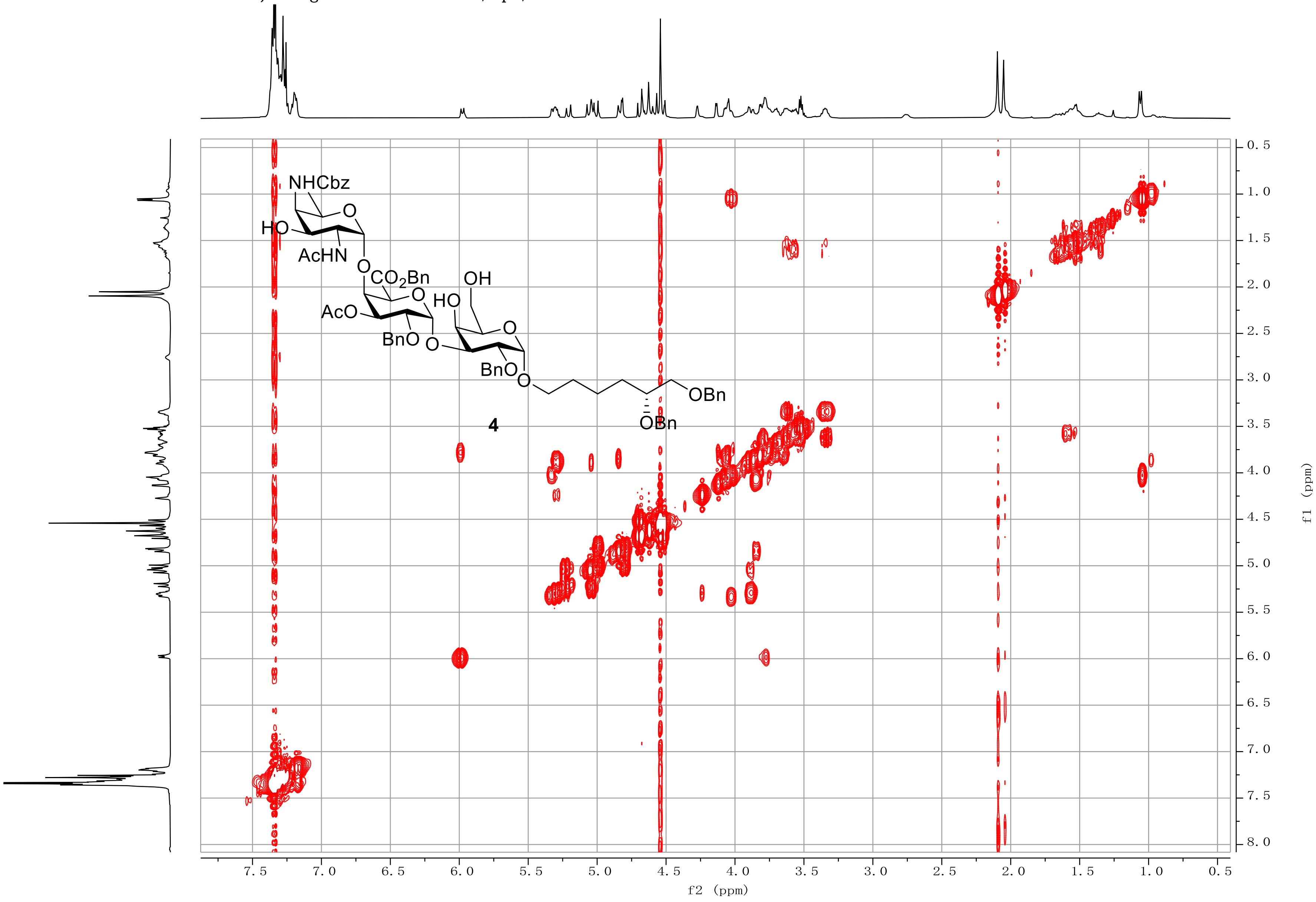

1901zhen.29.ser — wz515-2, 80mg — c13HSQC CDCl3 /opt/DATA nmrafd 15

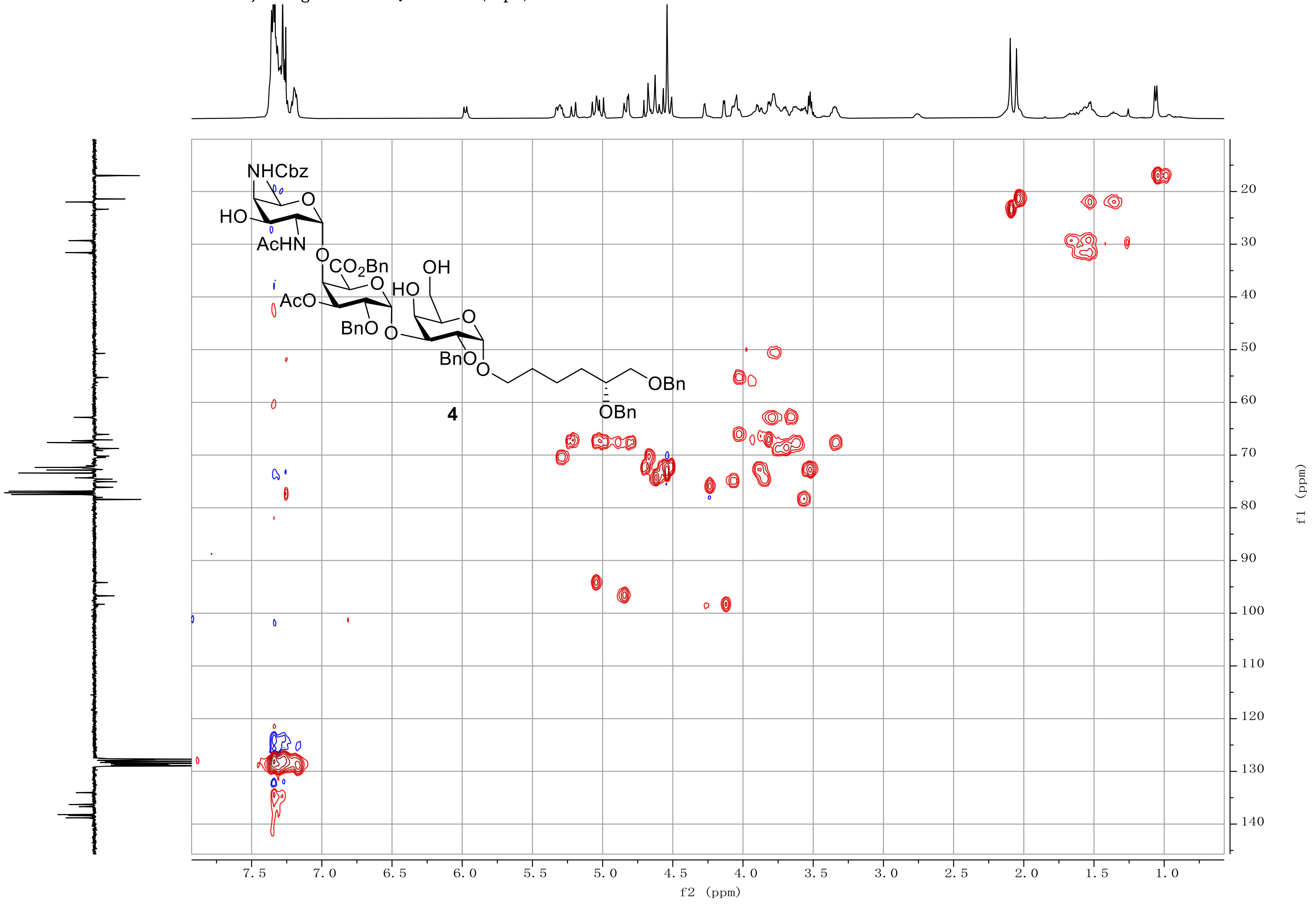

zhen1810biosyn.72.fid — wz516-3, 46 mg — bbo-h1 CDCl3 /opt/topspin2.1 nmrafd 16

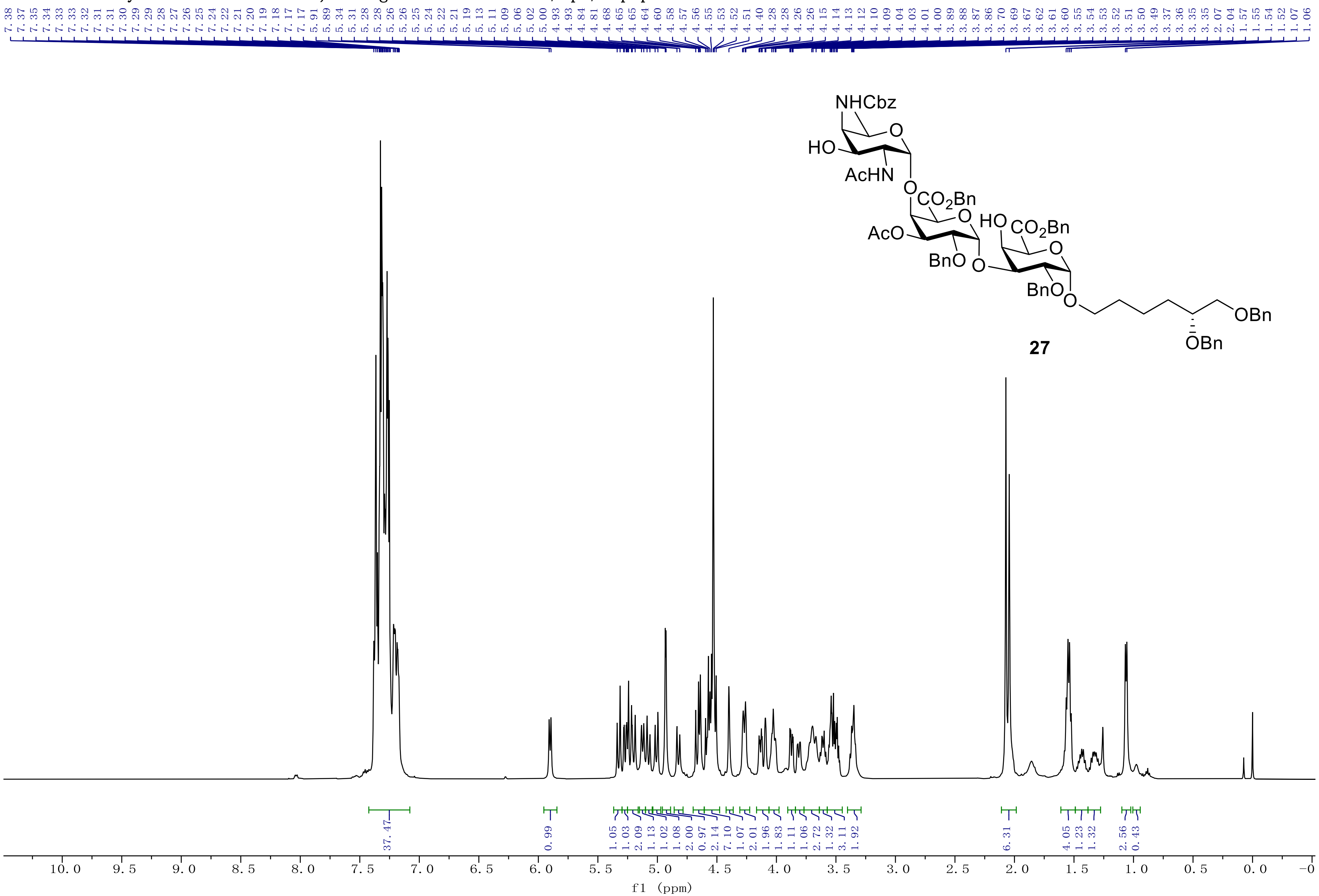

27

zhen1810biosyn.75.fid — wz516-3, 46 mg — bbo-c13-APT CDCl3 /opt/topspin2.1 nmrafd 16

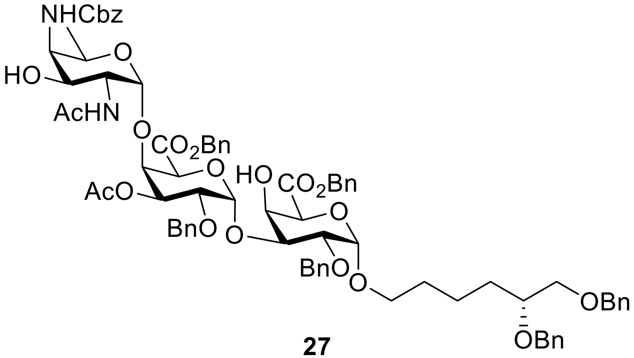

27

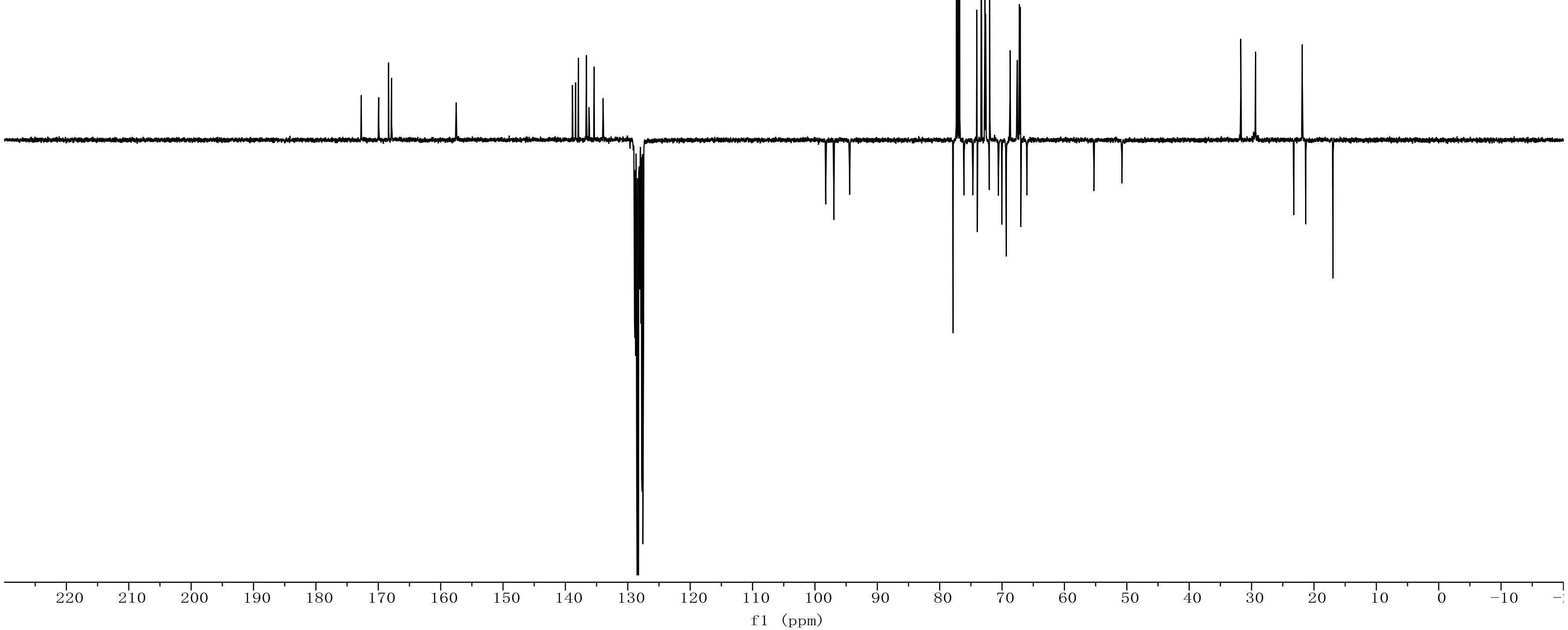

zhen1810biosyn.73.ser — wz516-3, 46 mg — bbo-h1-cosy CDCl3 /opt/topspin2.1 nmrafd 16

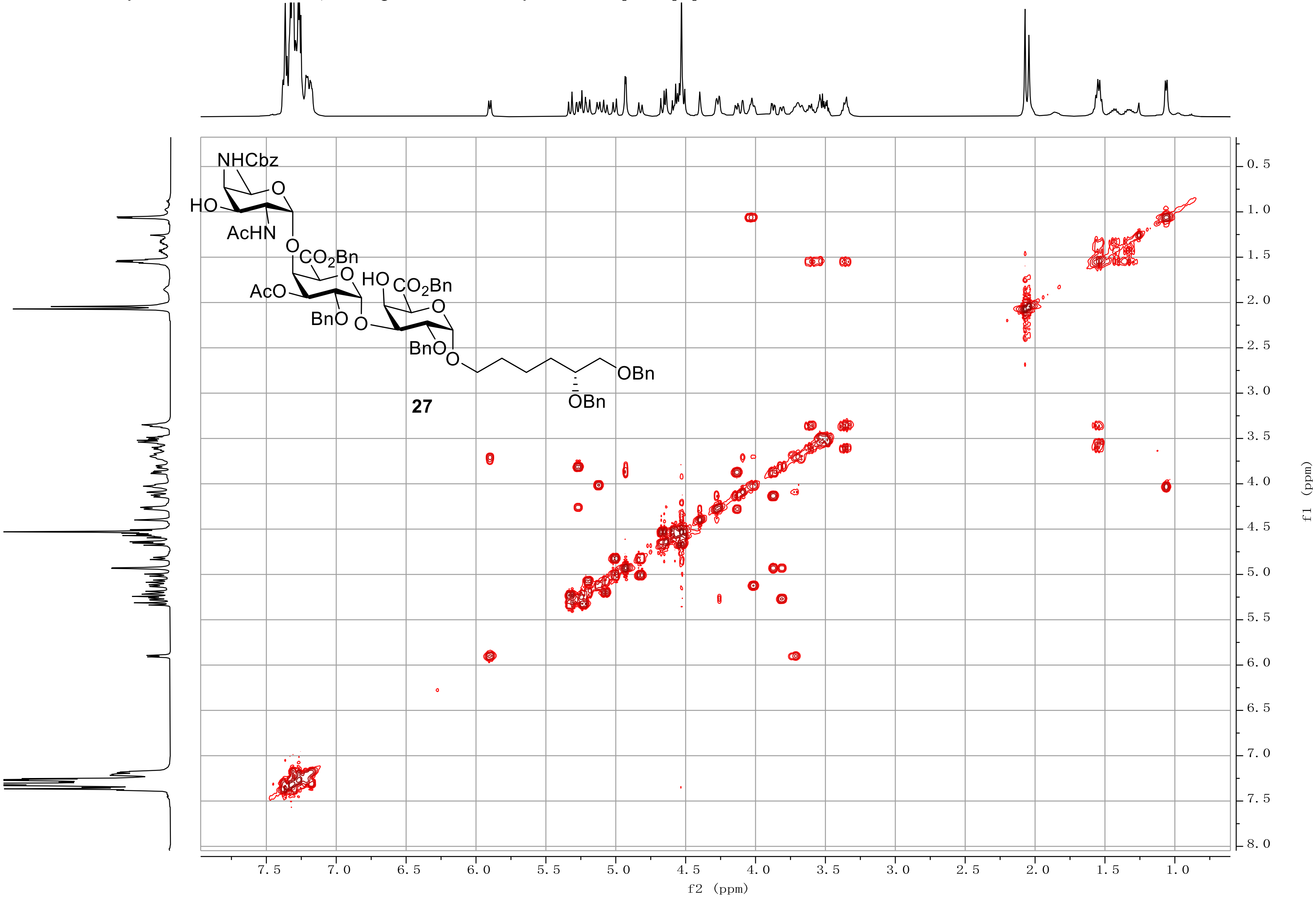

zhen1810biosyn.74.ser — wz516-3, 46 mg — bbo-c13-HSQC CDCl3 /opt/topspin2.1 nmrafd 16

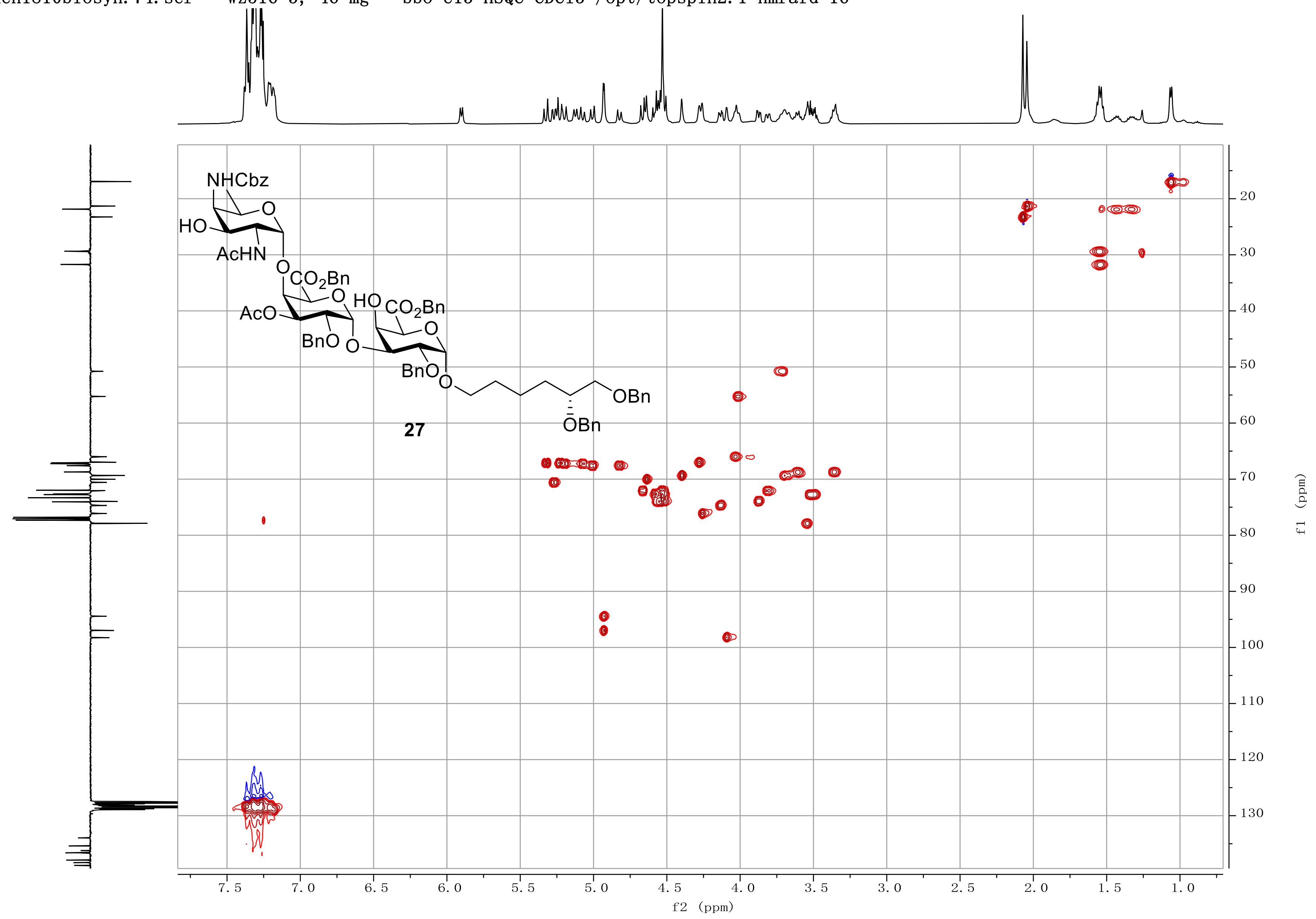

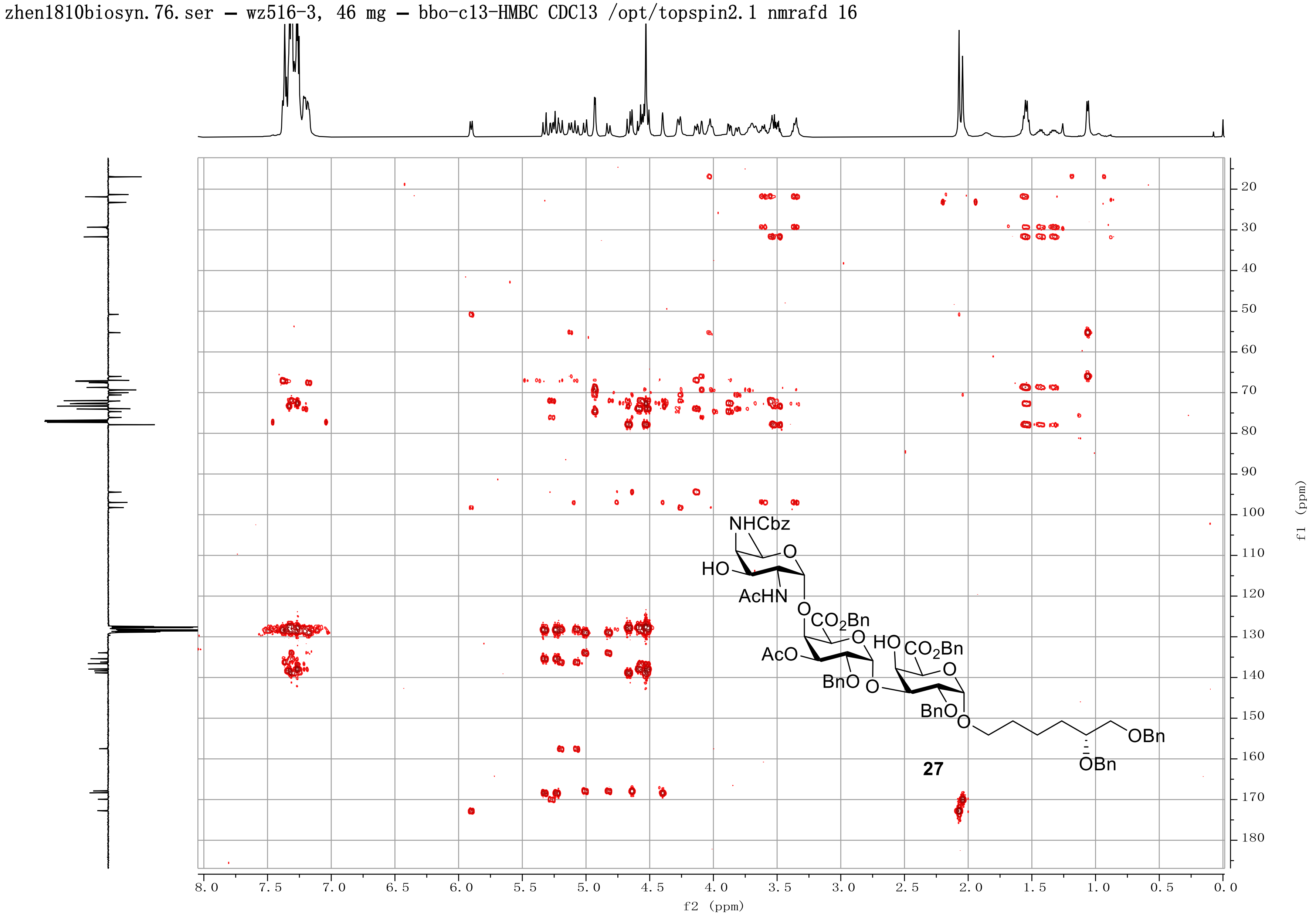
zhen1810biosyn.76.ser — wz516-3, 46 mg — bbo-c13-HMBC CDCl3 /opt/topspin2.1 nmrafd 16
NHCbz
HO
AcHN
CO2Bn
AcO
BnO
HO
CO2Bn
BnO
OBn
OBn
27
f2 (ppm)
f1 (ppm)

zhen1810biosyn.77.ser — wz516-3, 46 mg — bbo-c13-hmbc-ipv-gated CDCl3 /opt/topspin2.1 nmrafd 16

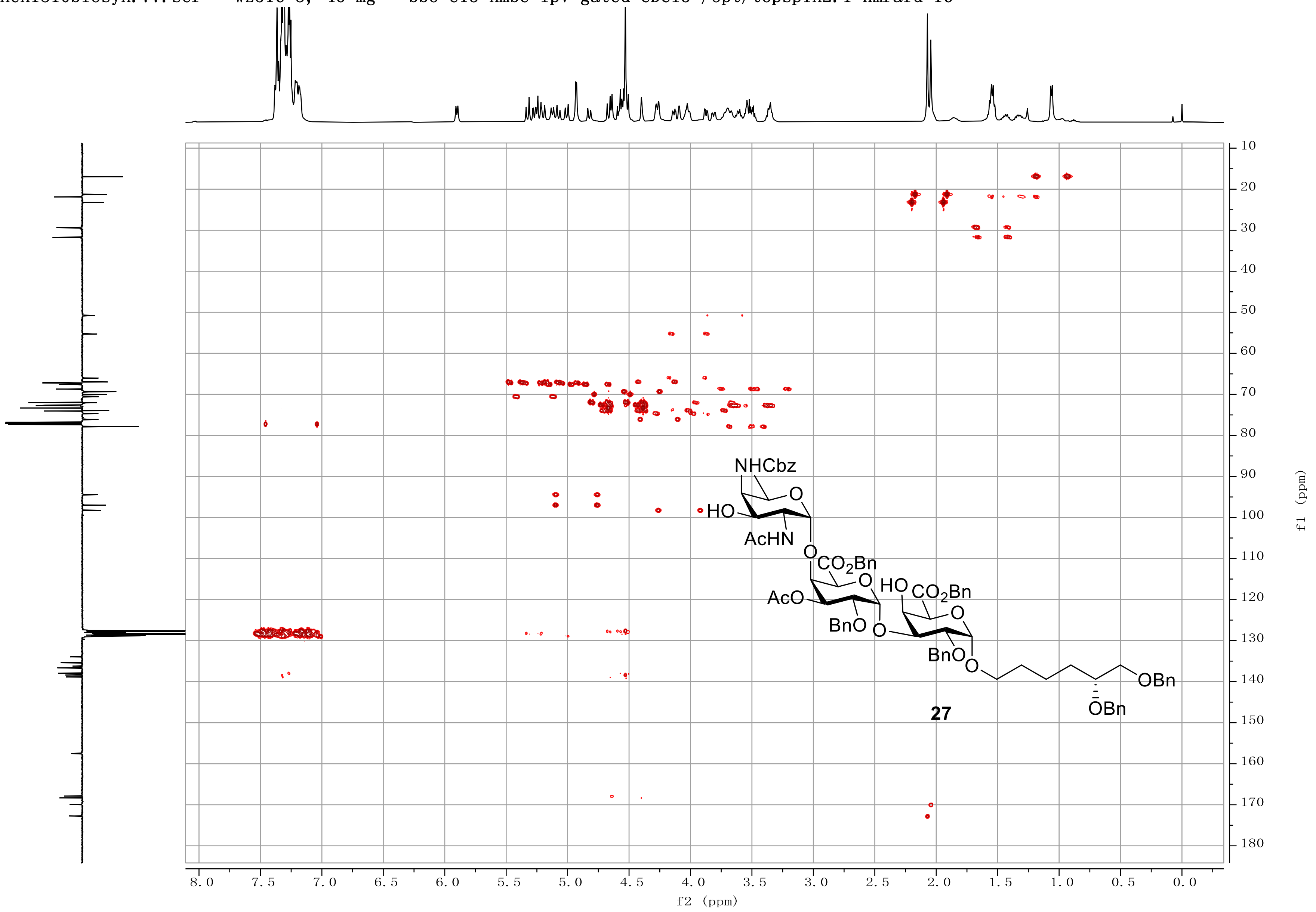

zhen1902biosyn.30.fid — wz517-E-2, 5mg — 1H, bbo, av500

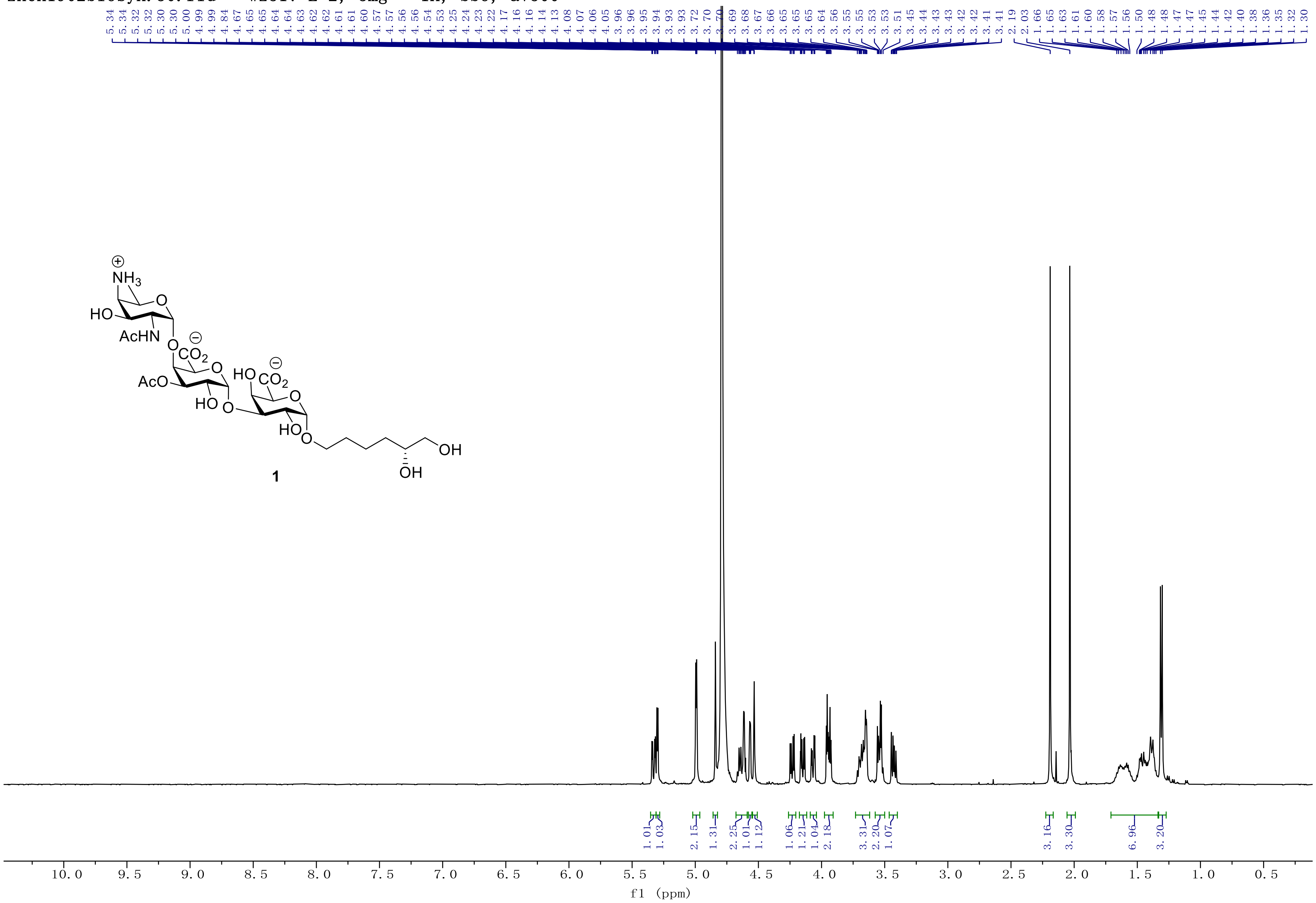

1902zhen.37.fid — wz517-E-2; 5mg — c13APT D2O /opt/DATA nmrafd 40

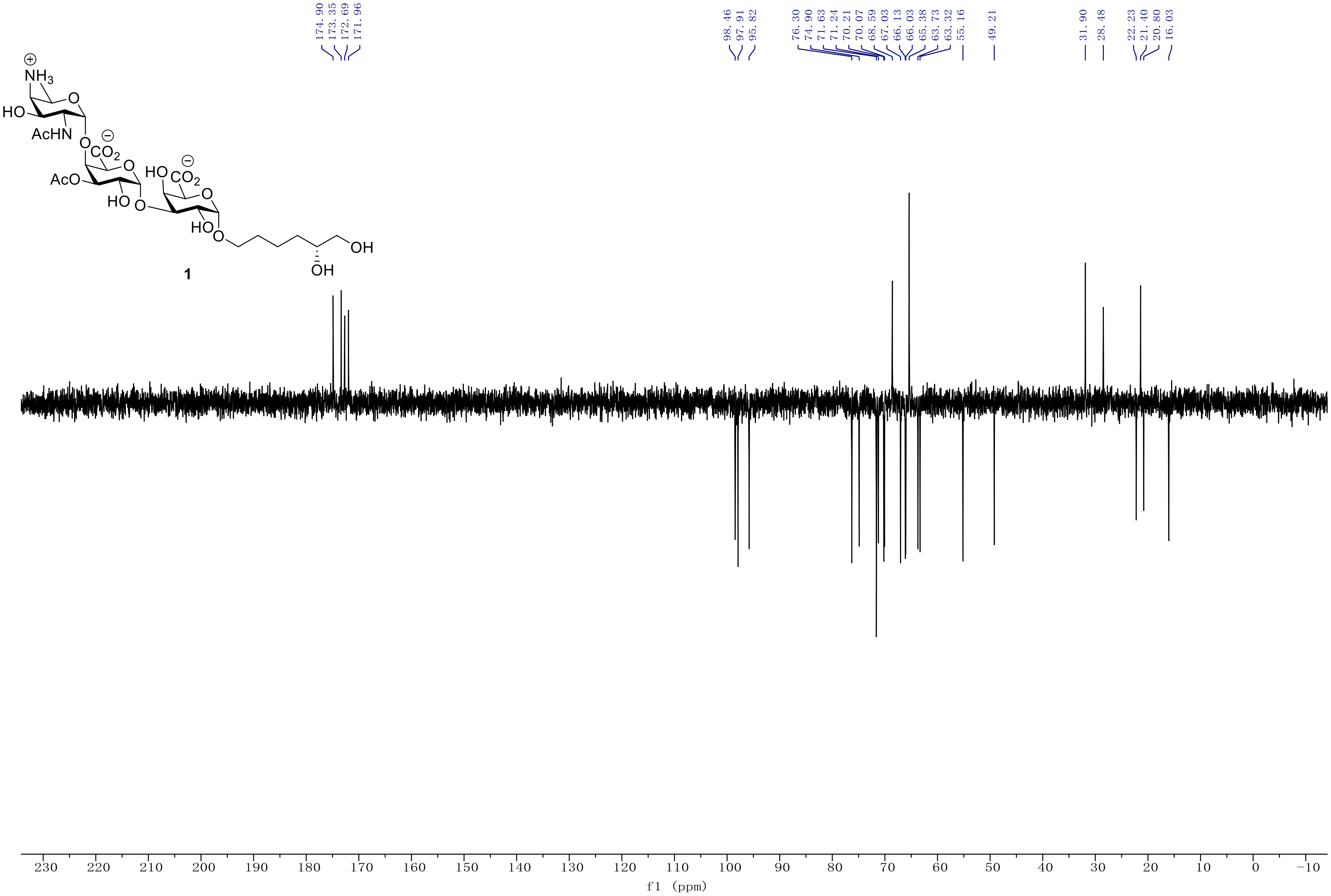

zhen1902biosyn.31.ser — wz517-E-2, 5mg — bbo-h1-cosy D20 /opt/topspin2.1 nmrafd 8

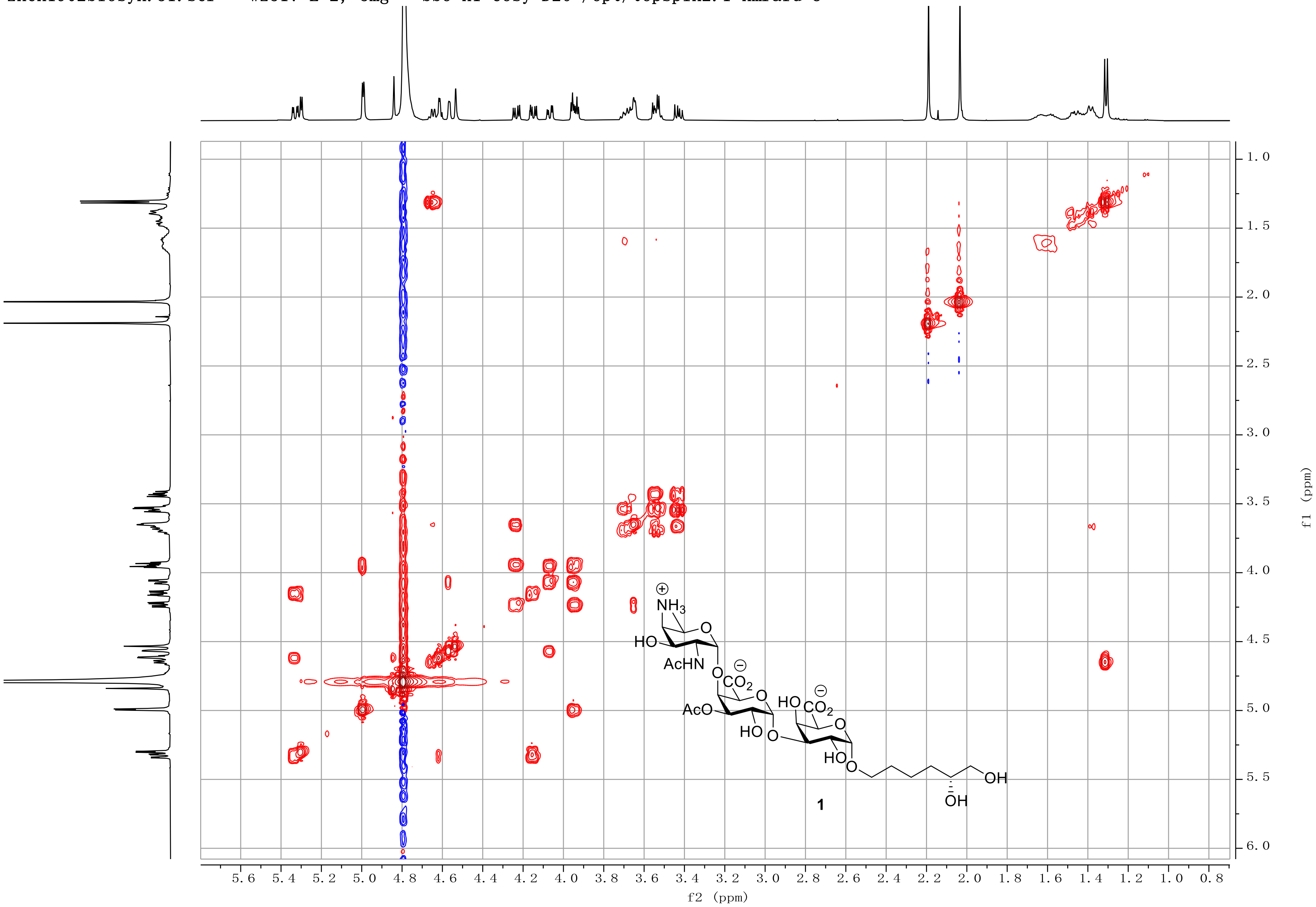

zhen1902biosyn.32.ser — wz517-E-2, 5mg — bbo-c13-HSQC D2O /opt/topspin2.1 nmrafd 8

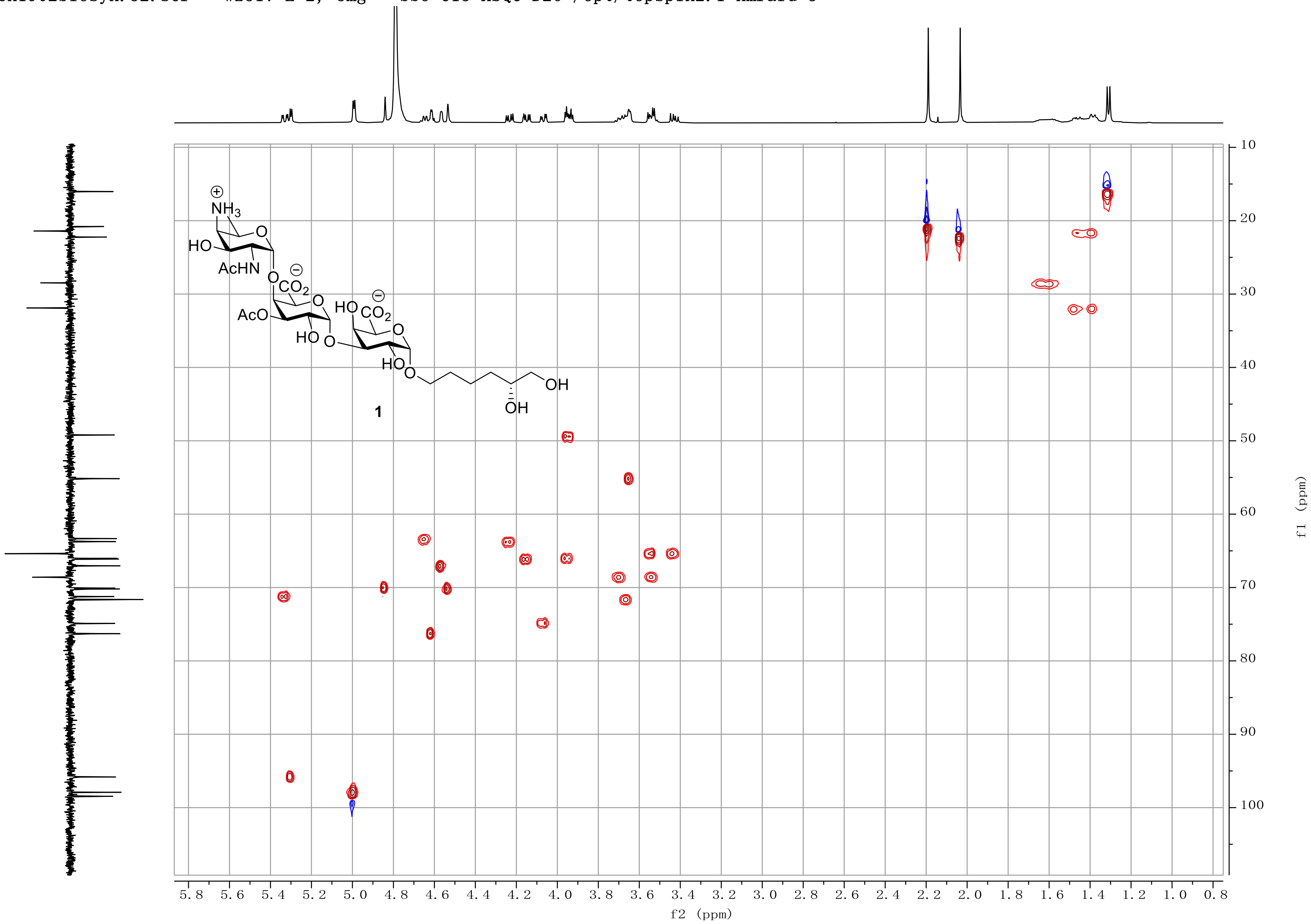

zhen1911biosyn.66.fid — wz538, Na+ — 1H, bbo, av500

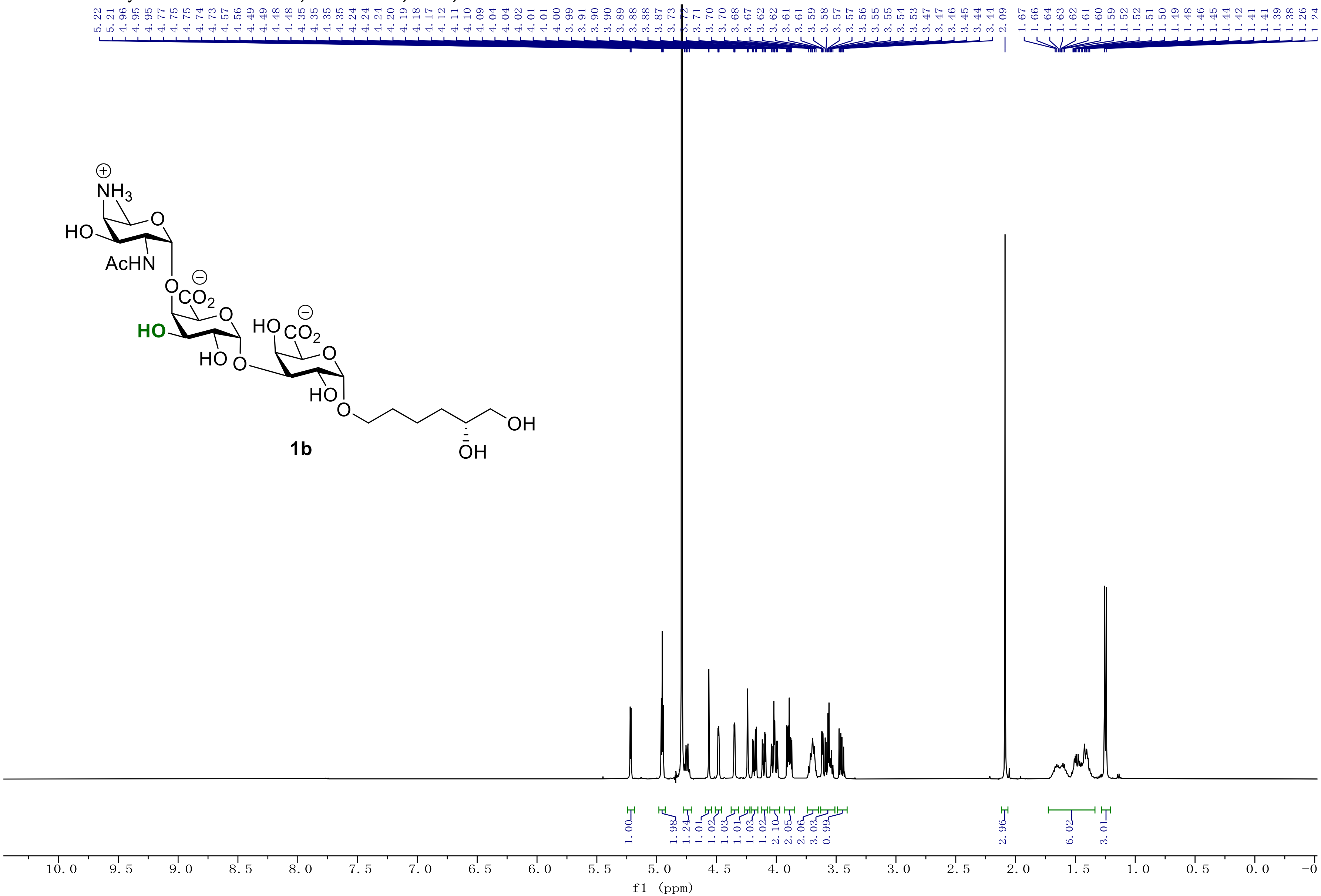

zhen1119biosyn.8.fid — 13C, APT, av850; wz538, 3-mer in D2O @ 298K

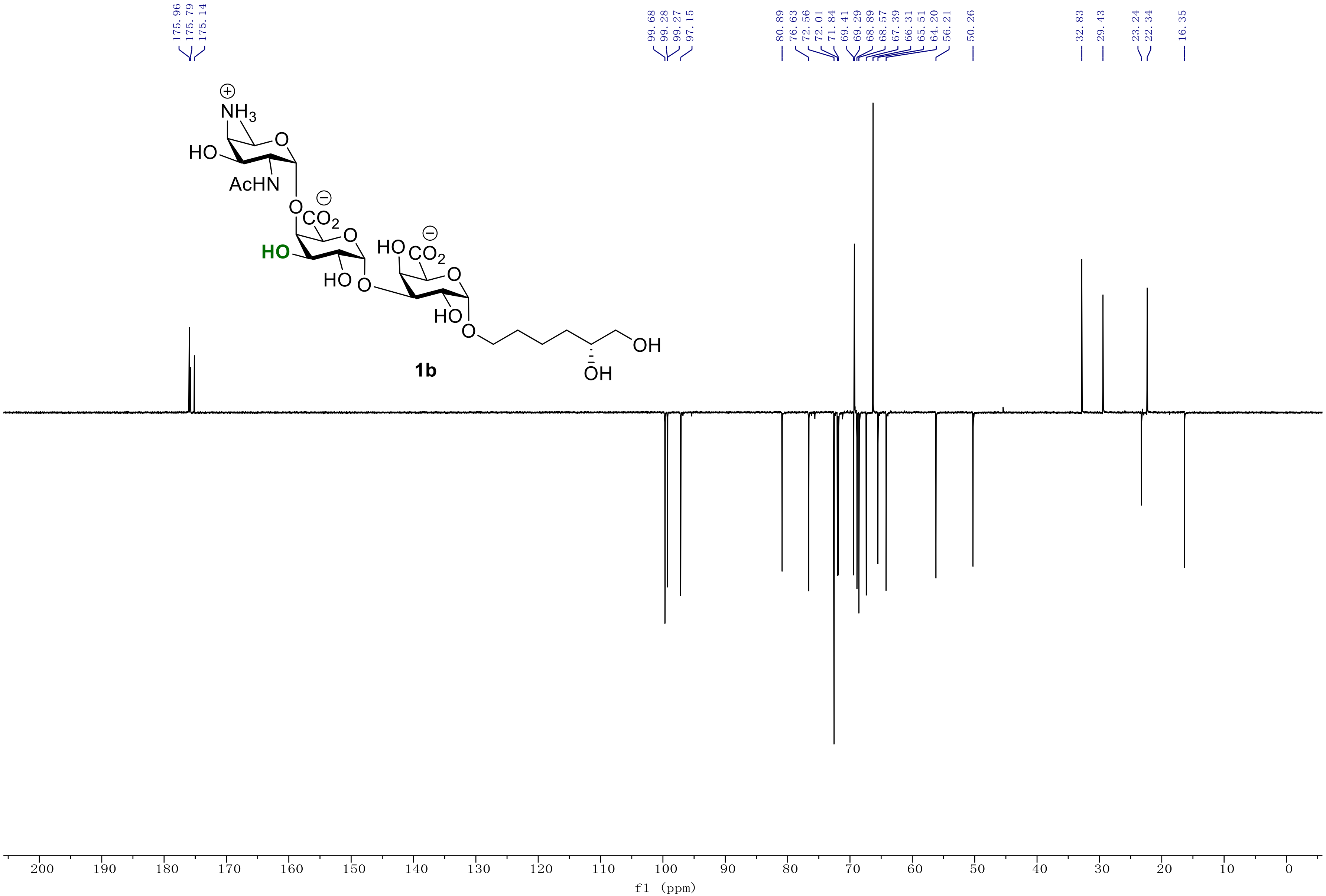

zhen1119biosyn.3.ser — 1H-1H cosy-presat, av850 , wz538, 3-mer in D2O @ 298K

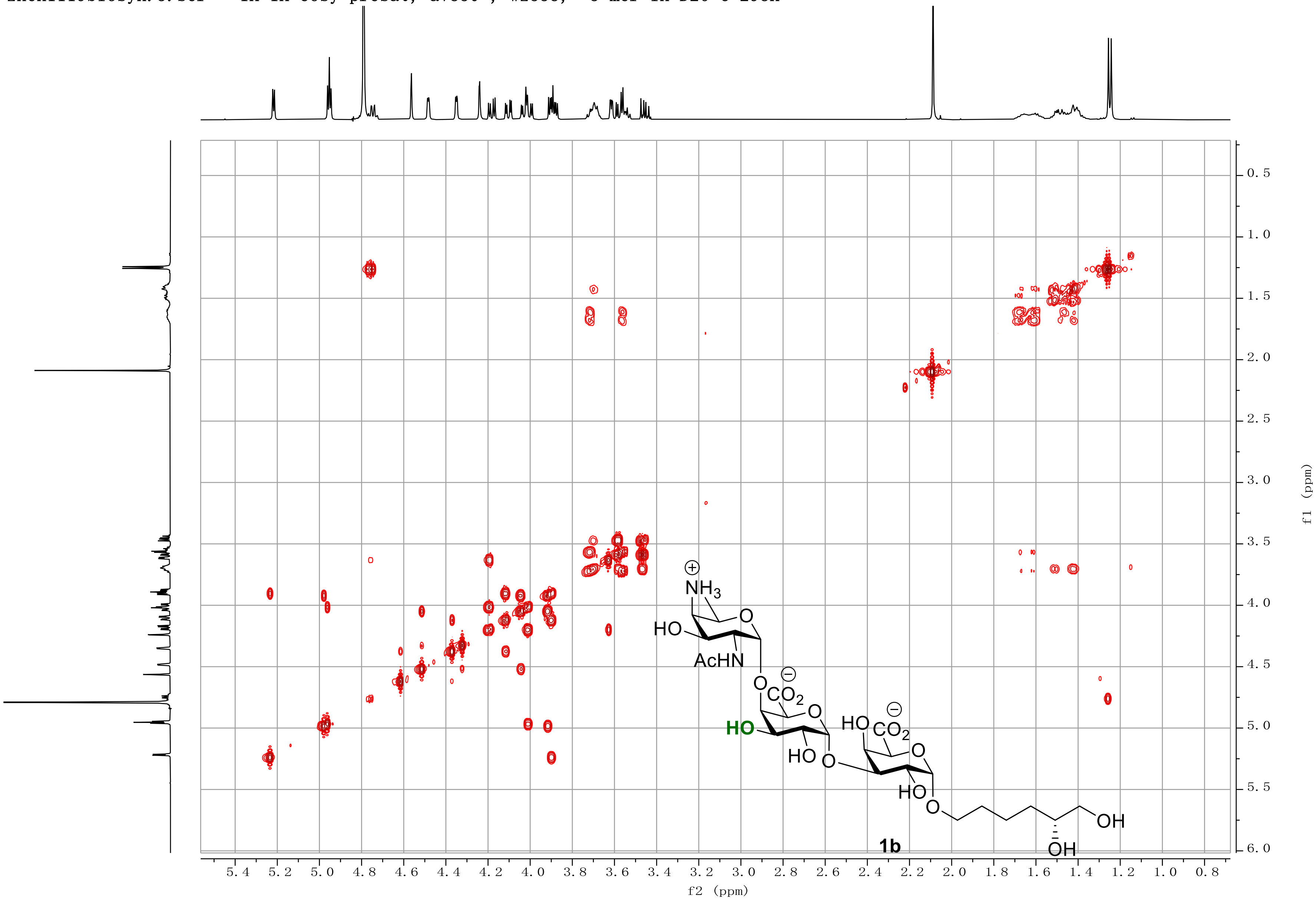

1b

zhen1119biosyn.4.ser — 1H-13C, hsqc-presat, wz538, 3-mer in D2O @ 298K

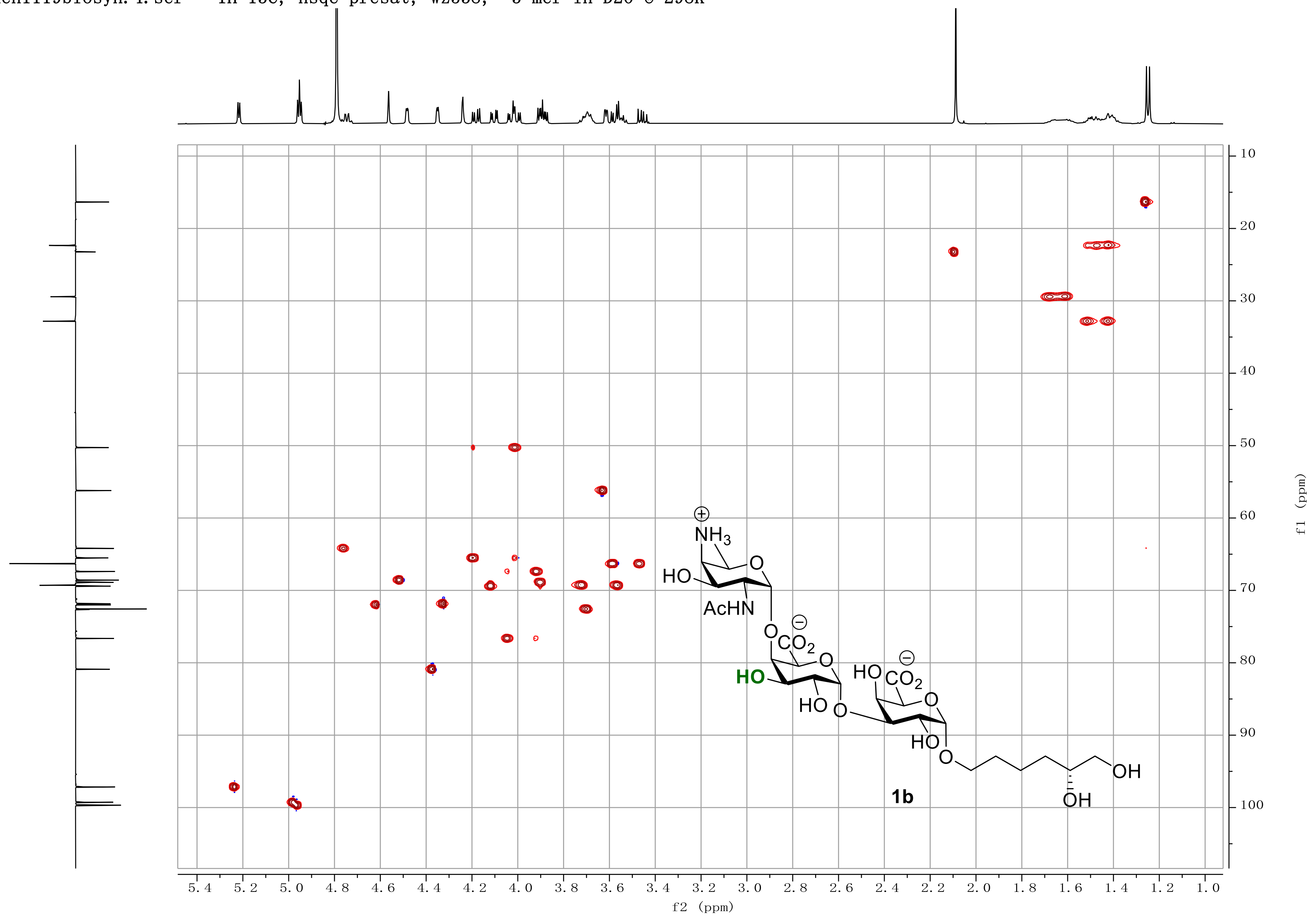

zhen1119biosyn.5.ser — 1H-13C, HMBC, av850; wz538, 3-mer in D2O @ 298K

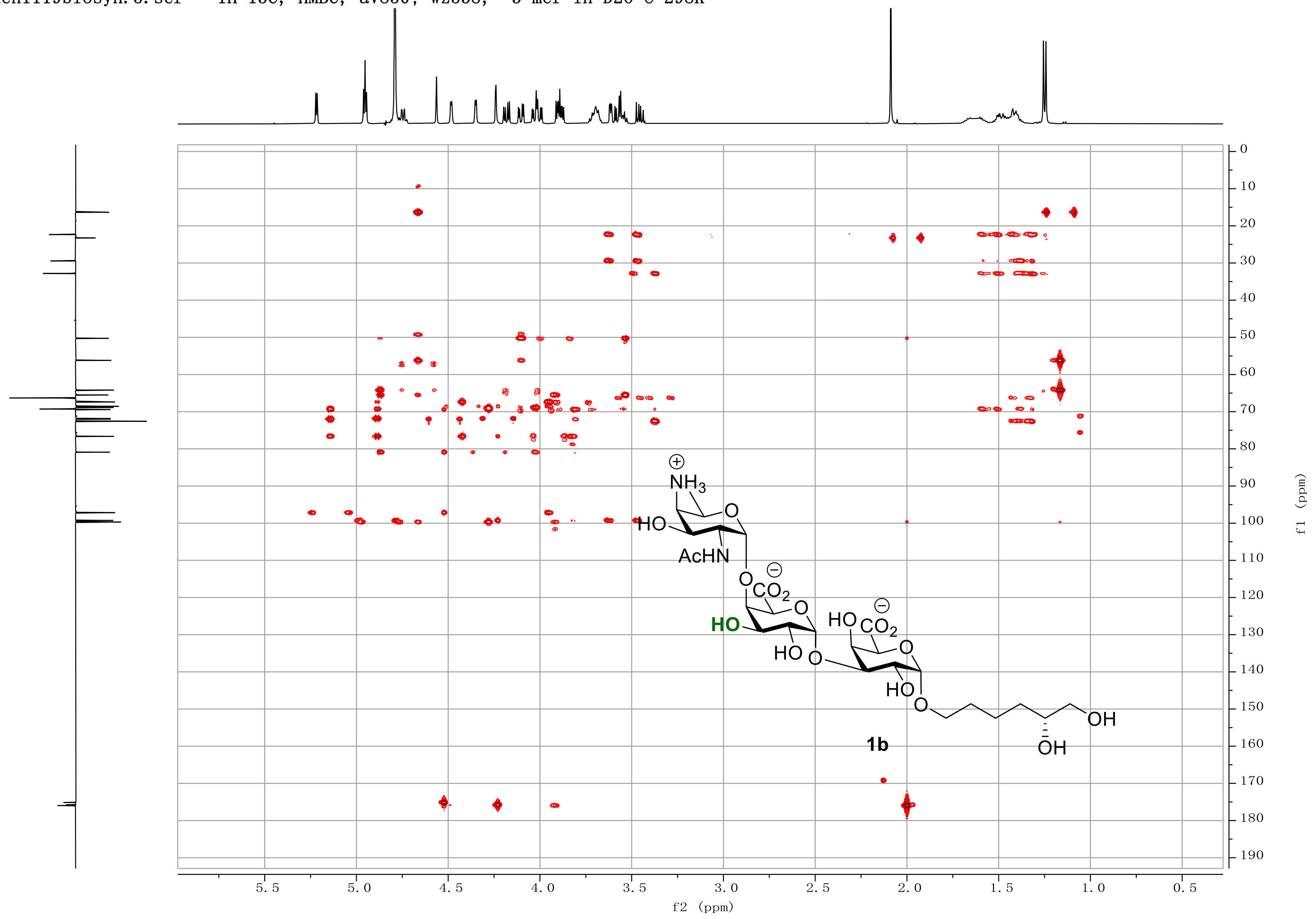

zhen1119biosyn.6.ser — 1H-13C, HMBC-gated-for-Direct-coupling; av850 , wz538, 3-mer in D2O @ 298K

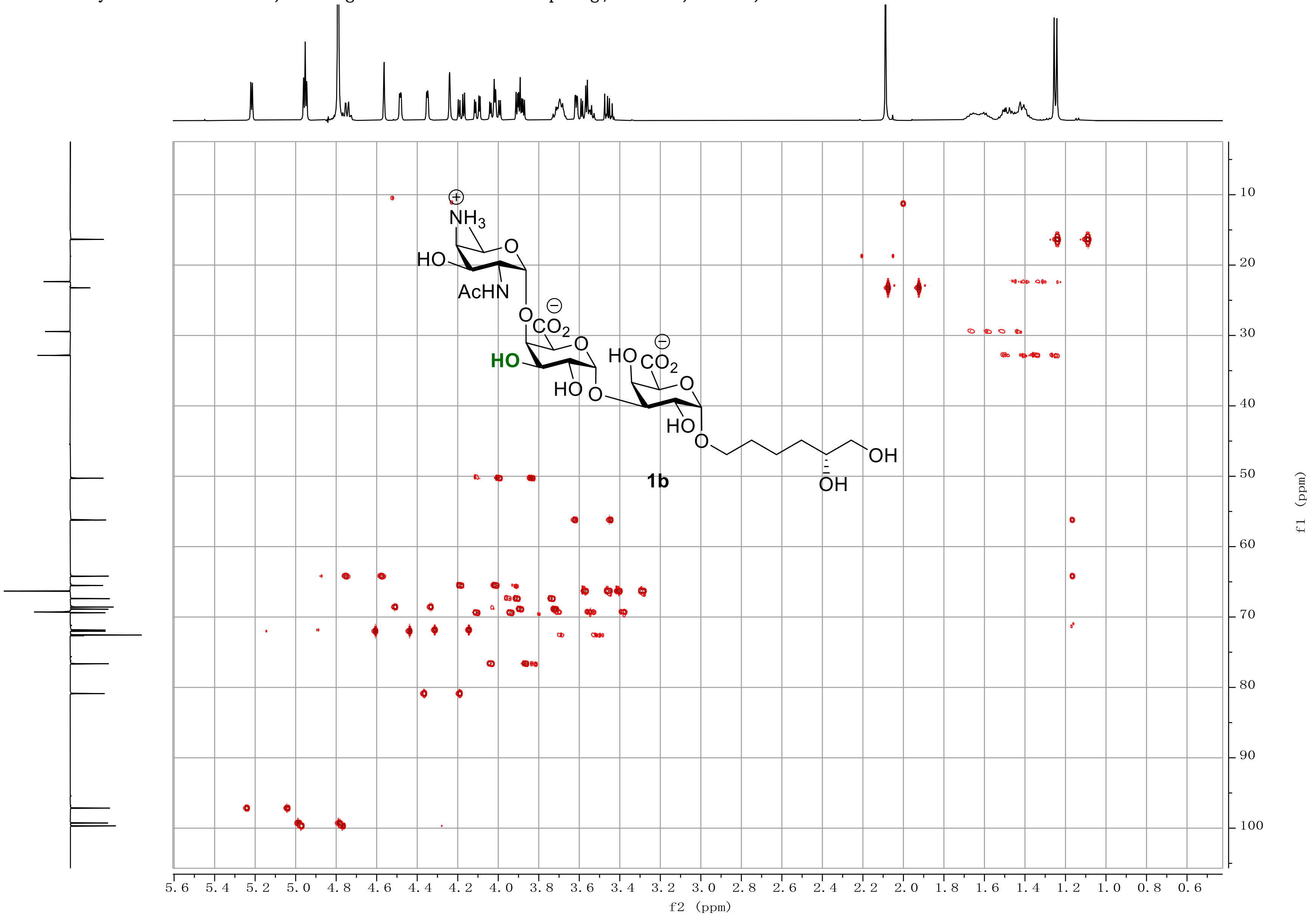

zhen1811biosyn.42.fid — wz522-4-1 — bbo-h1 CDCl3 /opt/topspin2.1 nmrafd 3

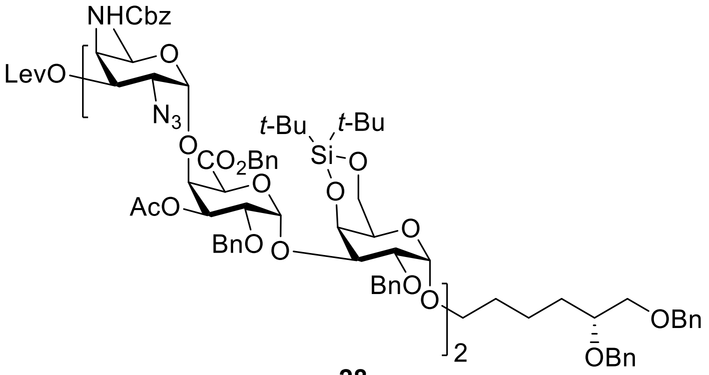

28

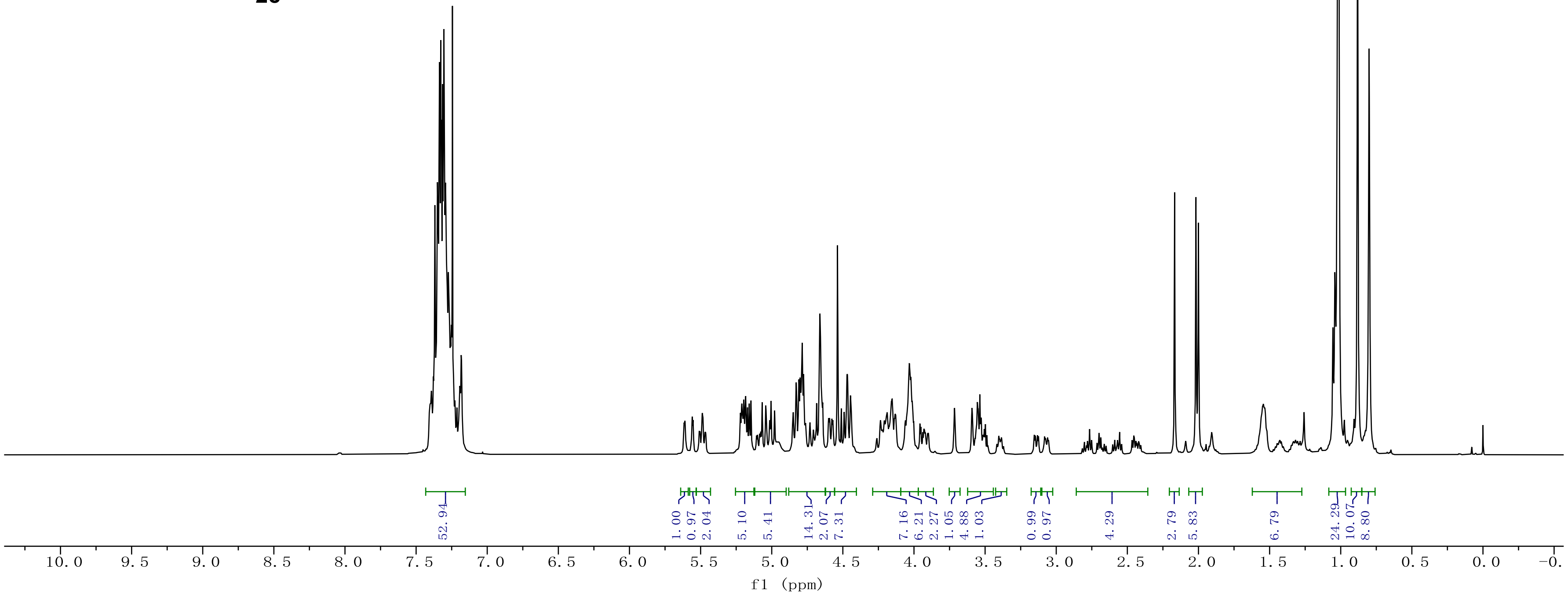

zhen1811biosyn.45.fid — wz522-4-1, size, 111mg — bbo-c13-APT CDCl3 /opt/topspin2.1 nmrafd 3

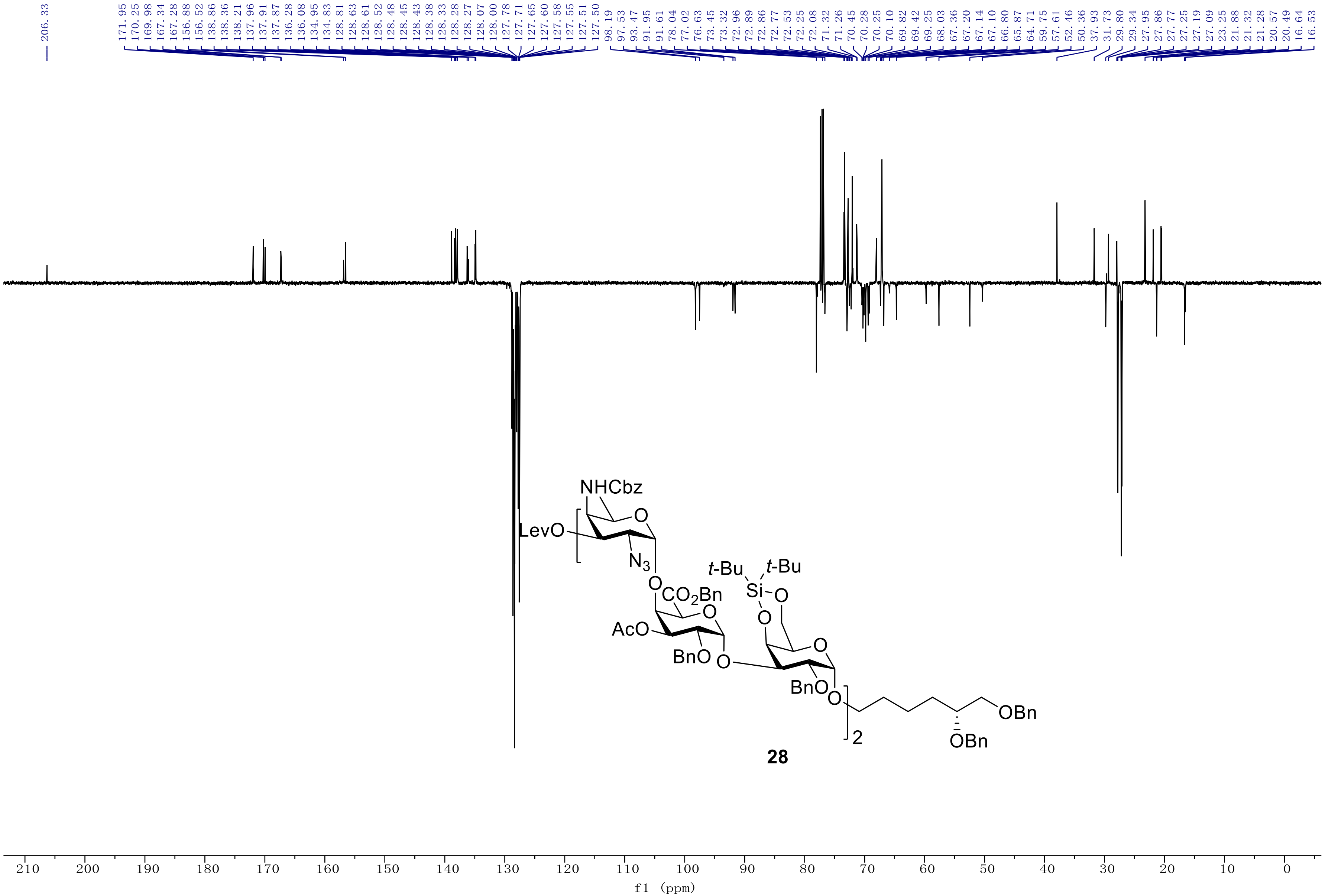

zhen1811biosyn.43.ser — wz522-4-1, size, 111mg — bbo-h1-cosy CDCl3 /opt/topspin2.1 nmrafd 3

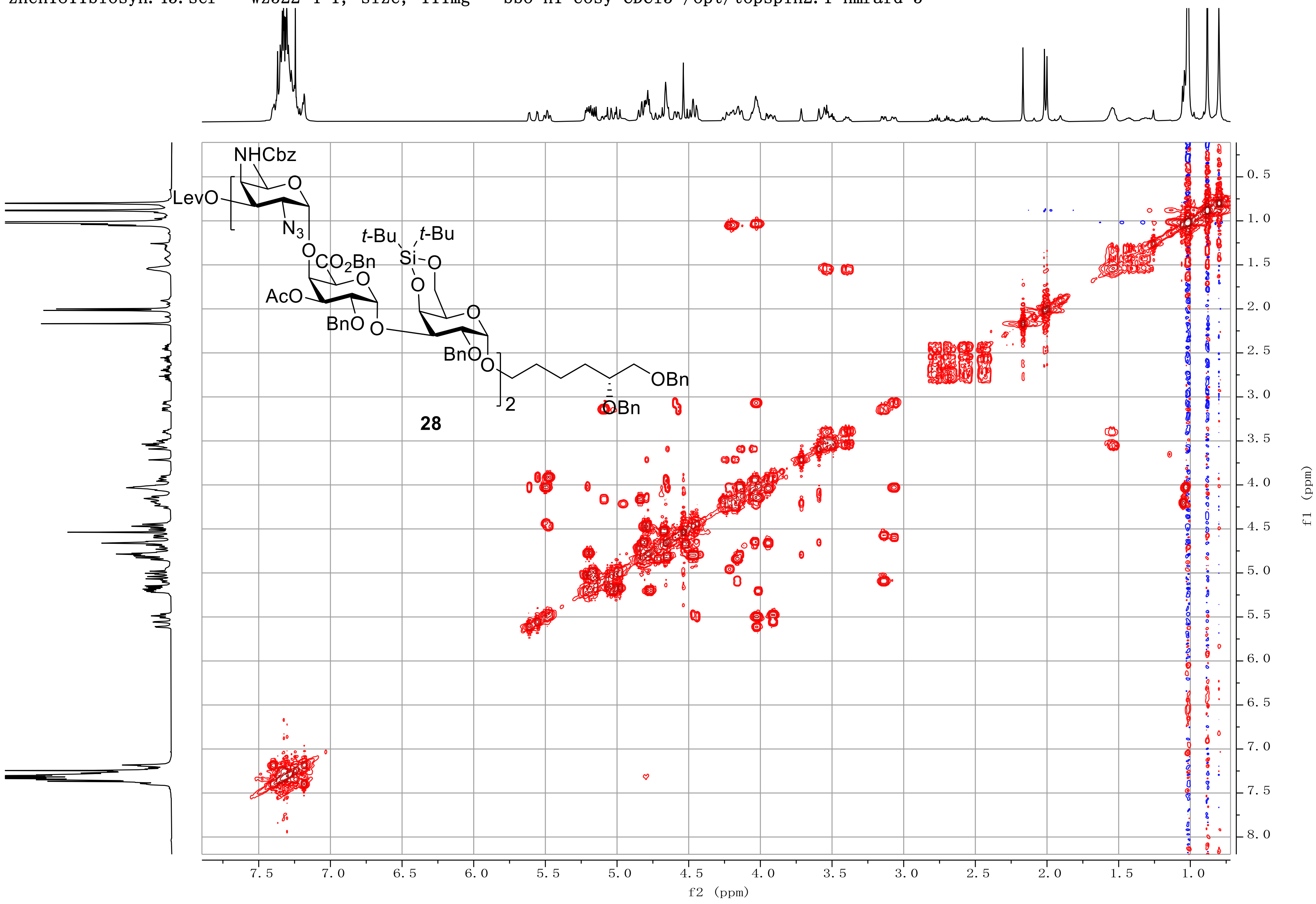

zhen1811biosyn.44.ser — wz522-4-1, size, 111mg — bbo-c13-HSQC CDCl3 /opt/topspin2.1 nmrafd 3

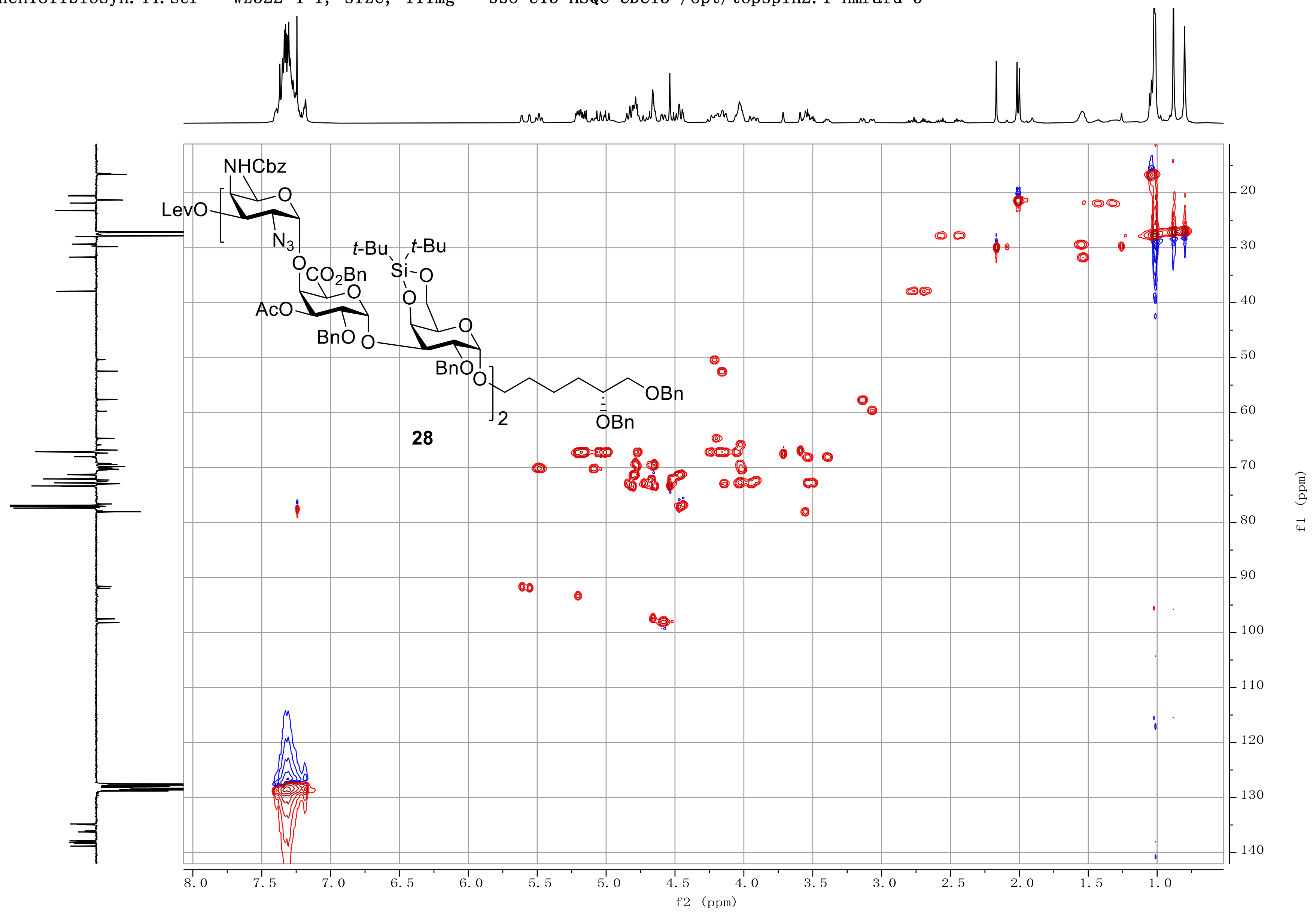

zhen1811biosyn.46.ser — wz522-4-1, size, 111mg — bbo-c13-HMBC CDCl3 /opt/topspin2.1 nmrafd 3

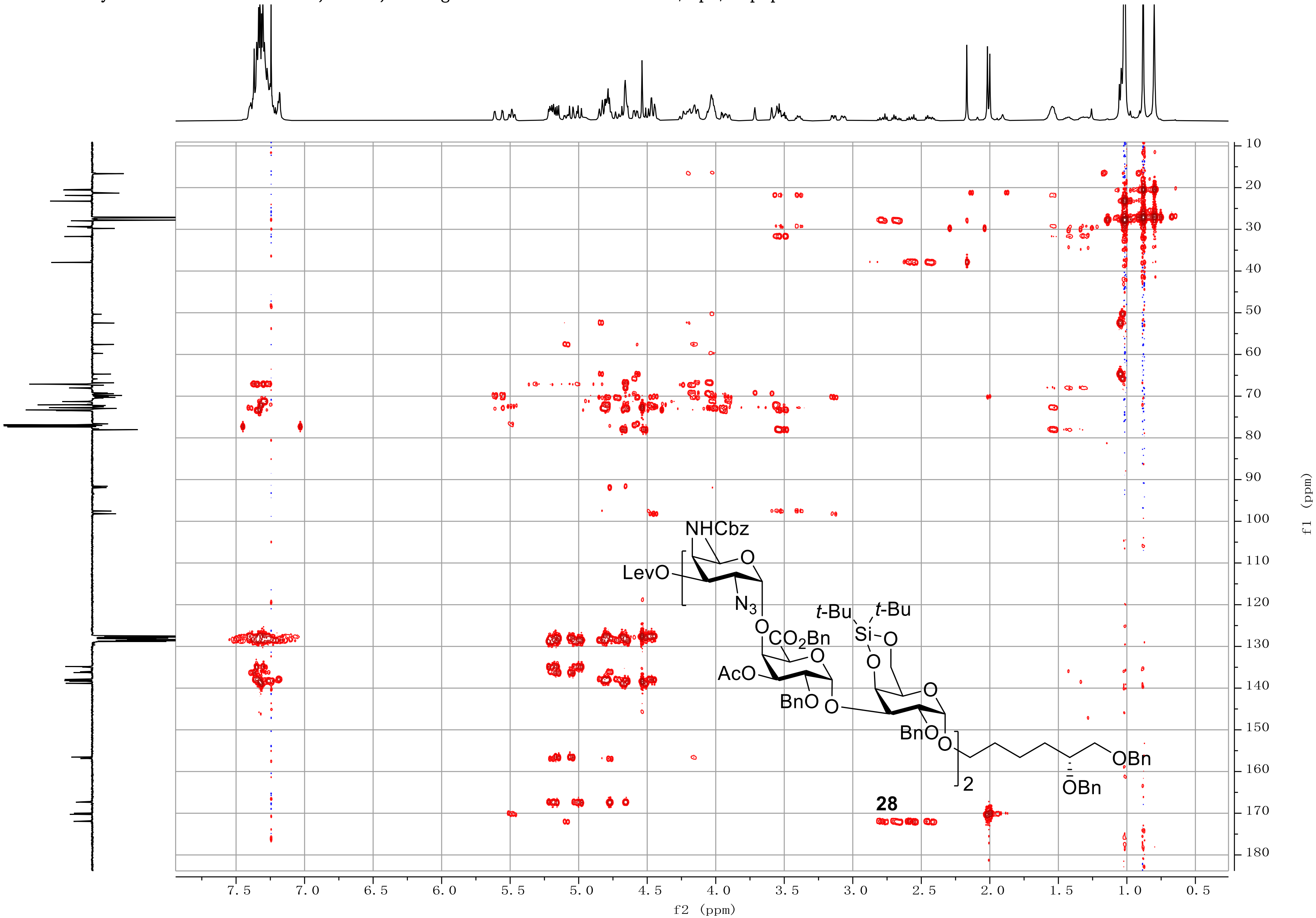

zhen1811biosyn.47.ser — wz522-4-1, size, 111mg — bbo-c13-hmbc-ipv-gated CDCl3 /opt/topspin2.1 nmrafd 3

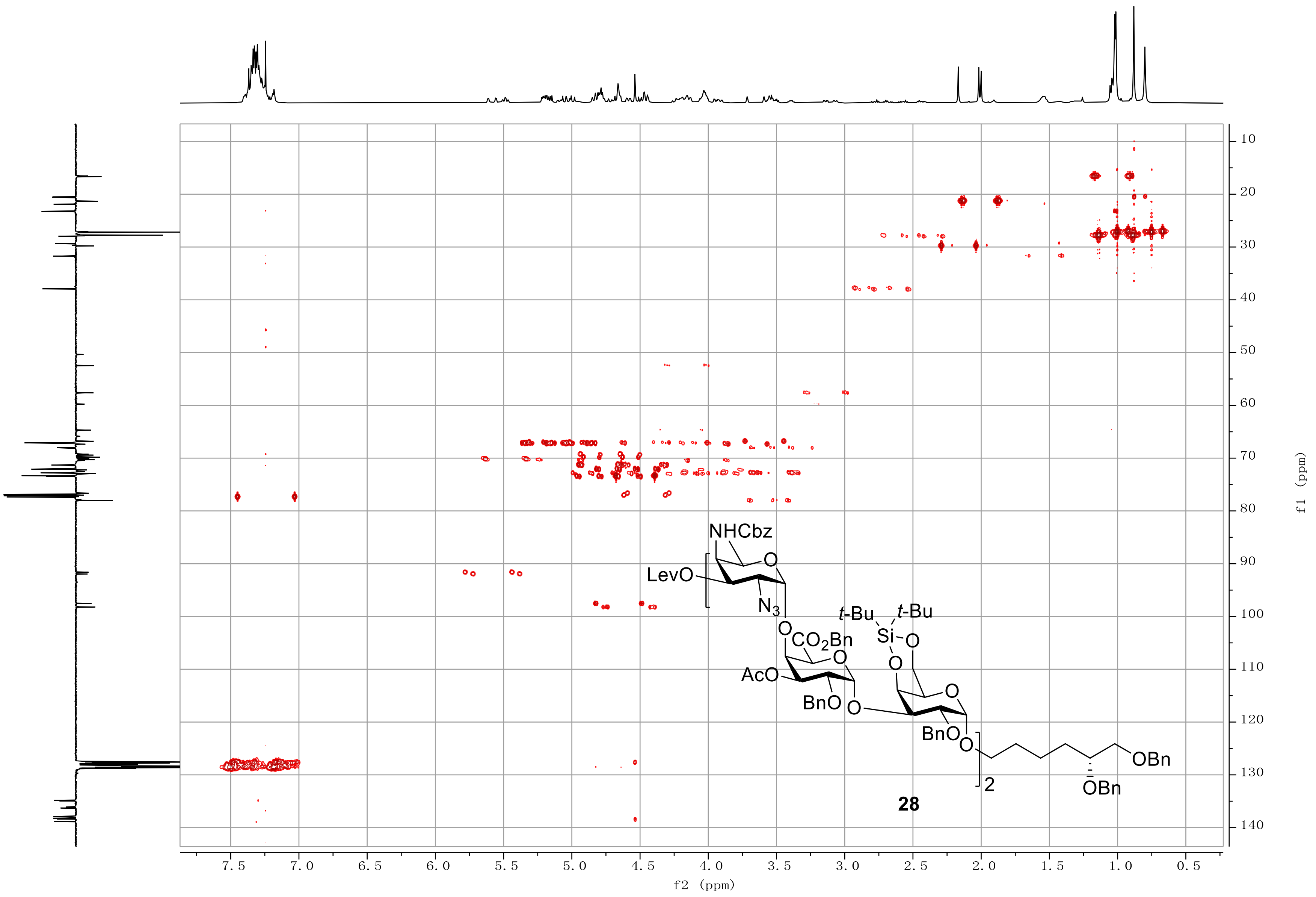

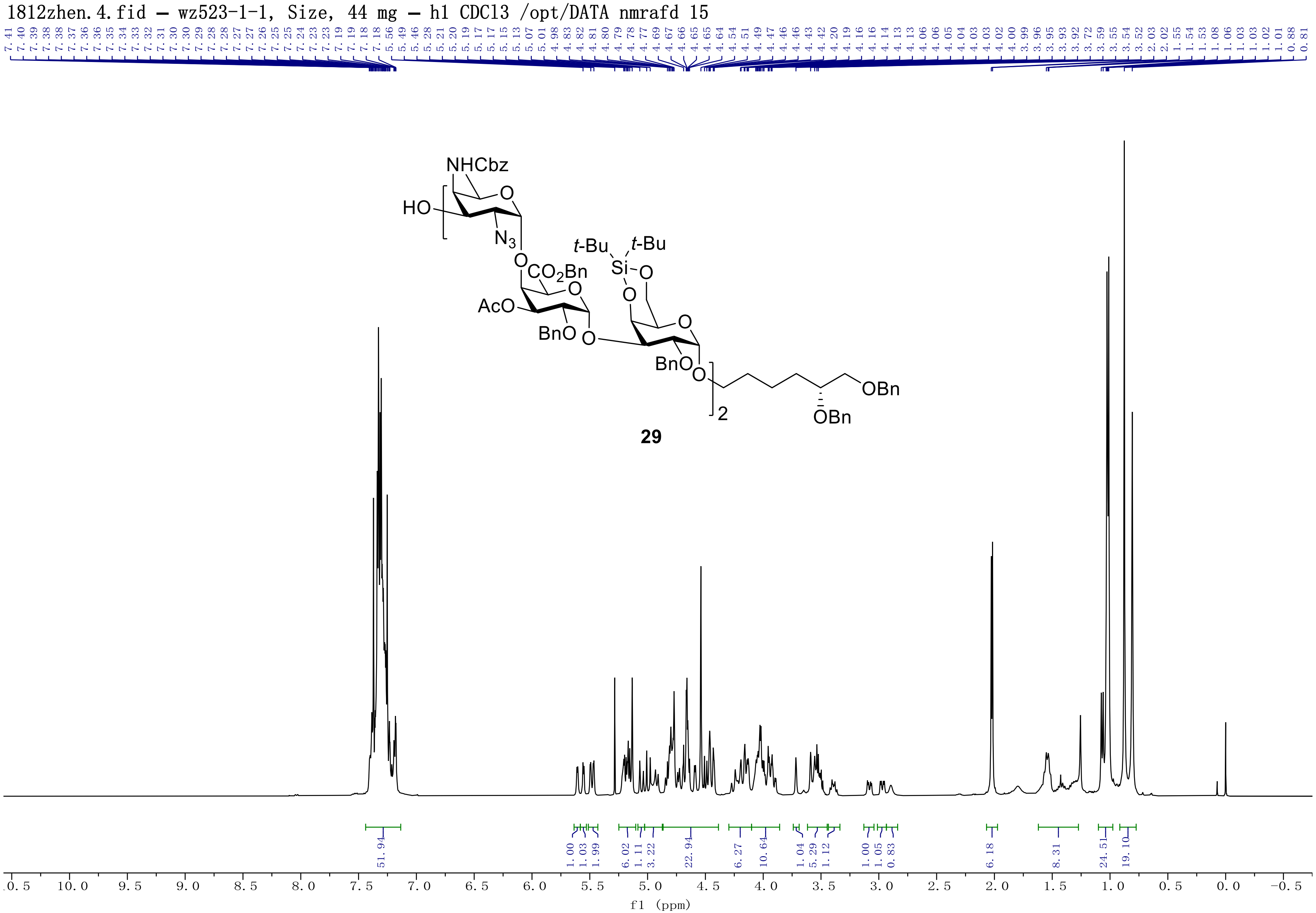
1812zhen.4.fid — wz523-1-1, Size, 44 mg — h1 CDCl3 /opt/DATA nmrafd 15
7.41 7.40 7.39 7.38 7.38 7.37 7.36 7.36 7.35 7.34 7.33 7.32 7.31 7.30 7.30 7.29 7.28 7.28 7.27 7.27 7.26 7.25 7.25 7.24 7.23 7.23 7.19 7.19 7.18 7.18 5.56 5.49 5.46 5.28 5.21 5.20 5.19 5.17 5.17 5.15 5.13 5.07 5.01 4.98 4.83 4.82 4.81 4.80 4.79 4.78 4.77 4.69 4.67 4.66 4.65 4.65 4.64 4.54 4.51 4.49 4.47 4.46 4.46 4.43 4.42 4.20 4.19 4.16 4.16 4.14 4.13 4.13 4.06 4.06 4.05 4.04 4.03 4.03 4.02 4.00 3.99 3.96 3.95 3.93 3.92 3.72 3.59 3.55 3.54 3.52 2.03 2.02 1.55 1.54 1.53 1.08 1.06 1.03 1.03 1.02 1.01 0.88 0.81
NHCbz
HO
N3
O
CO2Bn
AcO
BnO
t-Bu
t-Bu
Si
BnO
2
OBn
OBn
29
51.94
1.00 1.03 1.99
6.02 1.11 3.22
22.94
6.27 10.64
1.04 5.29 1.12
1.00 1.05 0.83
6.18
8.31
24.51 19.10
10.5 10.0 9.5 9.0 8.5 8.0 7.5 7.0 6.5 6.0 5.5 5.0 4.5 4.0 3.5 3.0 2.5 2.0 1.5 1.0 0.5 0.0 -0.5
f1 (ppm)

1812zhen.5.fid — wz523-1-1, Size, 44 mg — C13APT CDCl3 /opt/DATA nmrafd 15

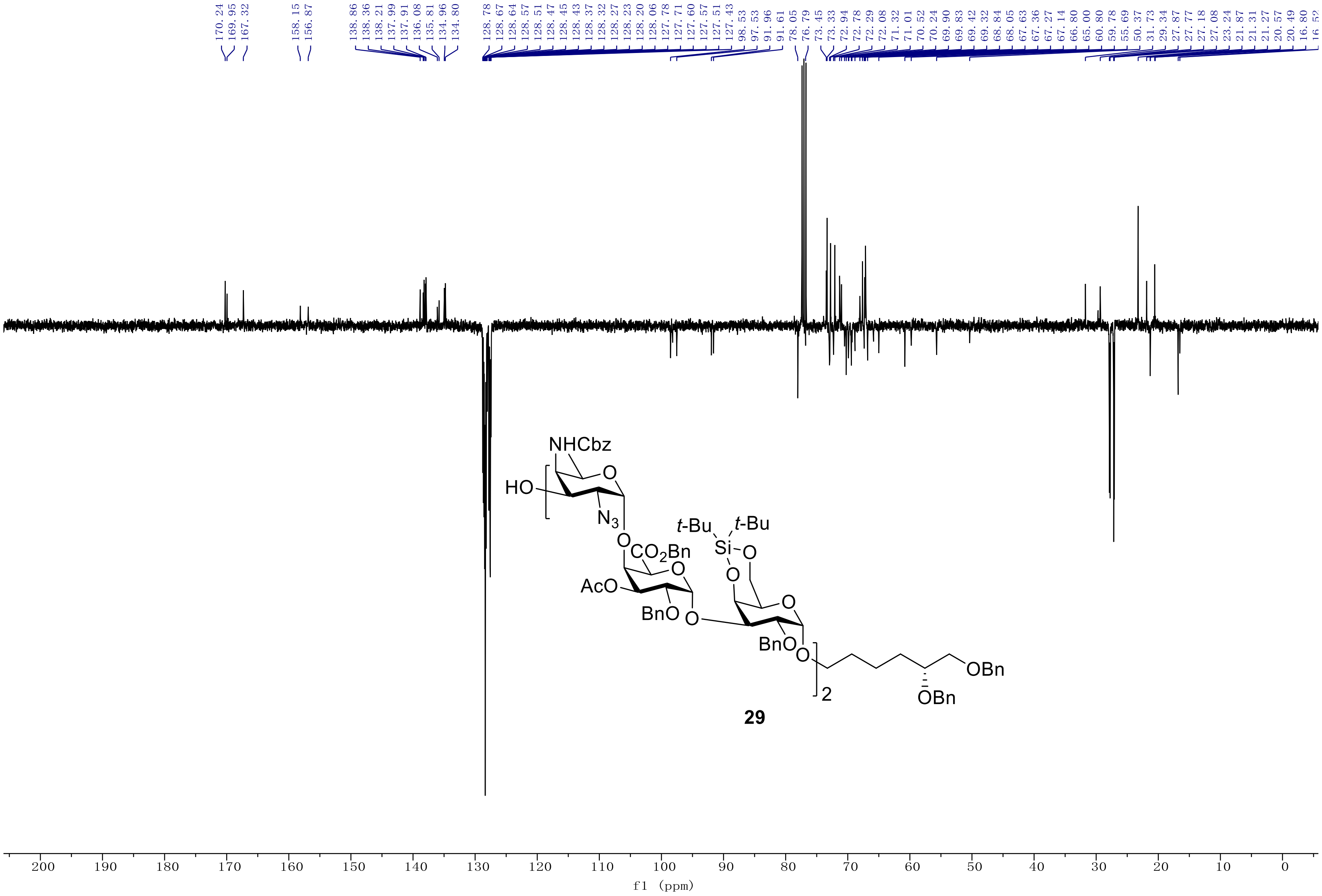

1812zhen.6.ser — wz523-1-1, Size, 44 mg — h1COSY CDCl3 /opt/DATA nmrafd 15

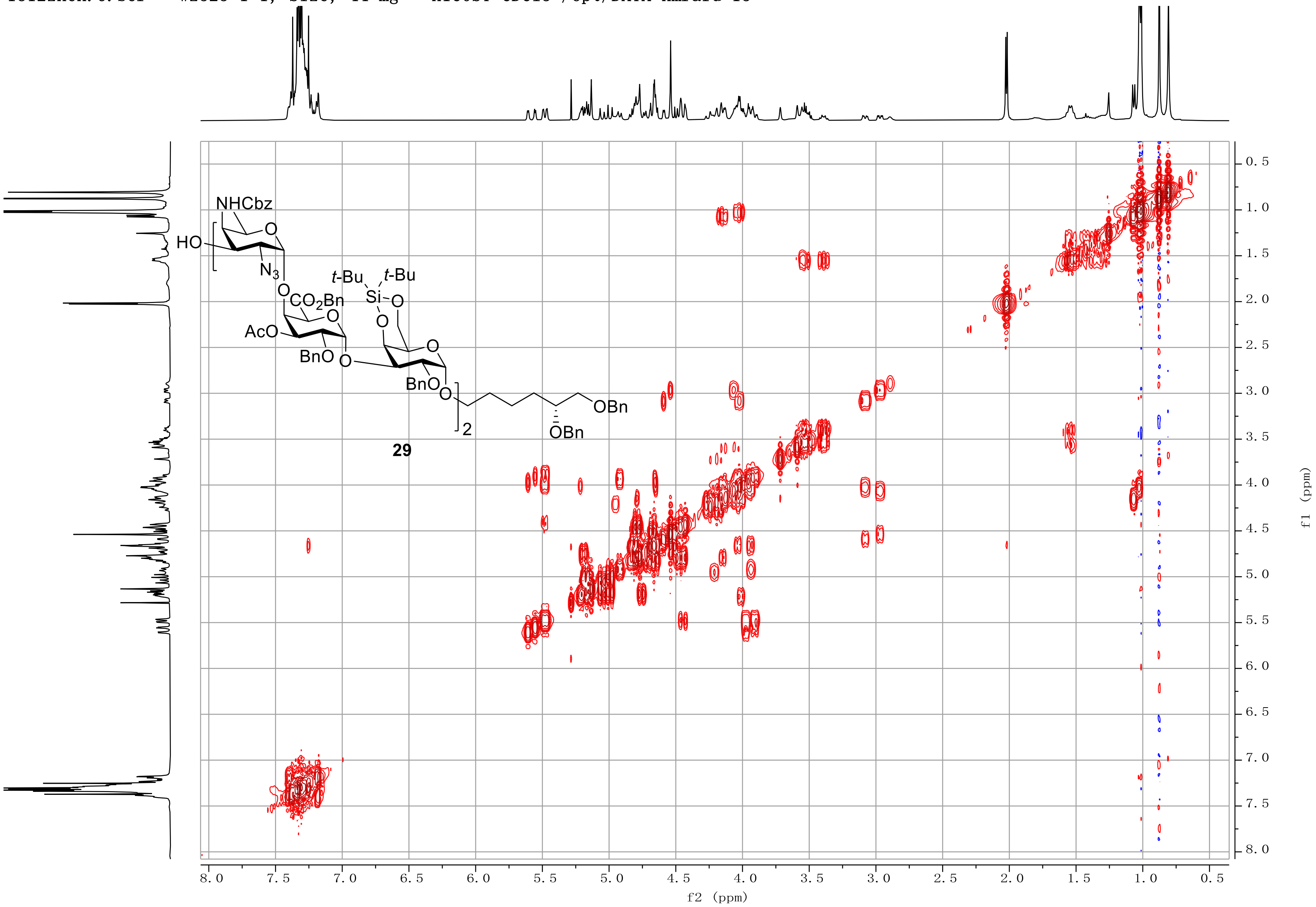

1812zhen.7.ser — wz523-1-1, Size, 44 mg — c13HSQC CDCl3 /opt/DATA nmrafd 15

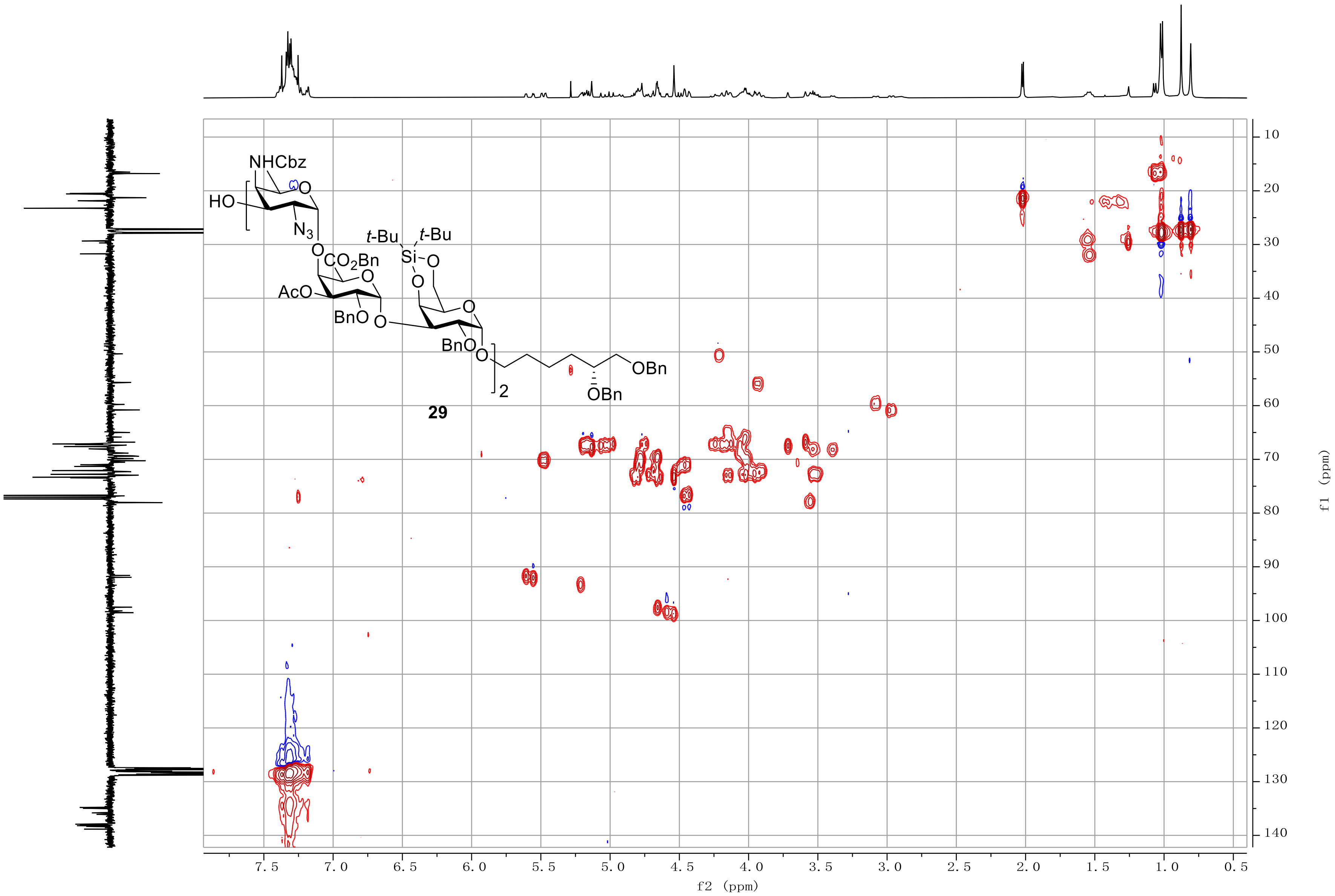

Biosyn022019Zhen.1.fid — 1H, av600, txi, ns =32 , 298K WZ 525-B in CDCl3;

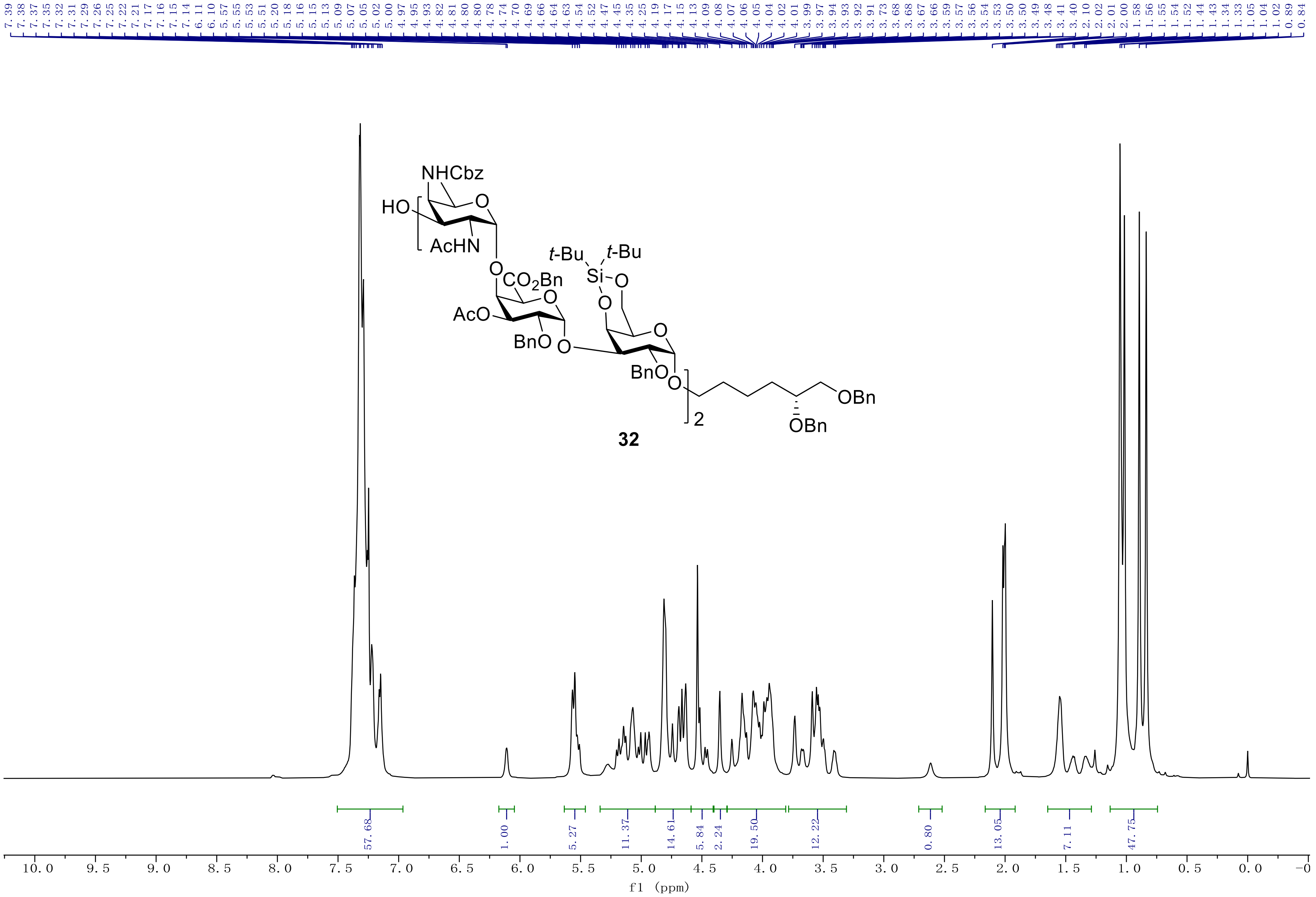

Biosyn022019Zhen.9.fid — 13C-APT, av600; WZ 525-B in CDCl3 @ 298K — ns =12384 — Lb=4, efp

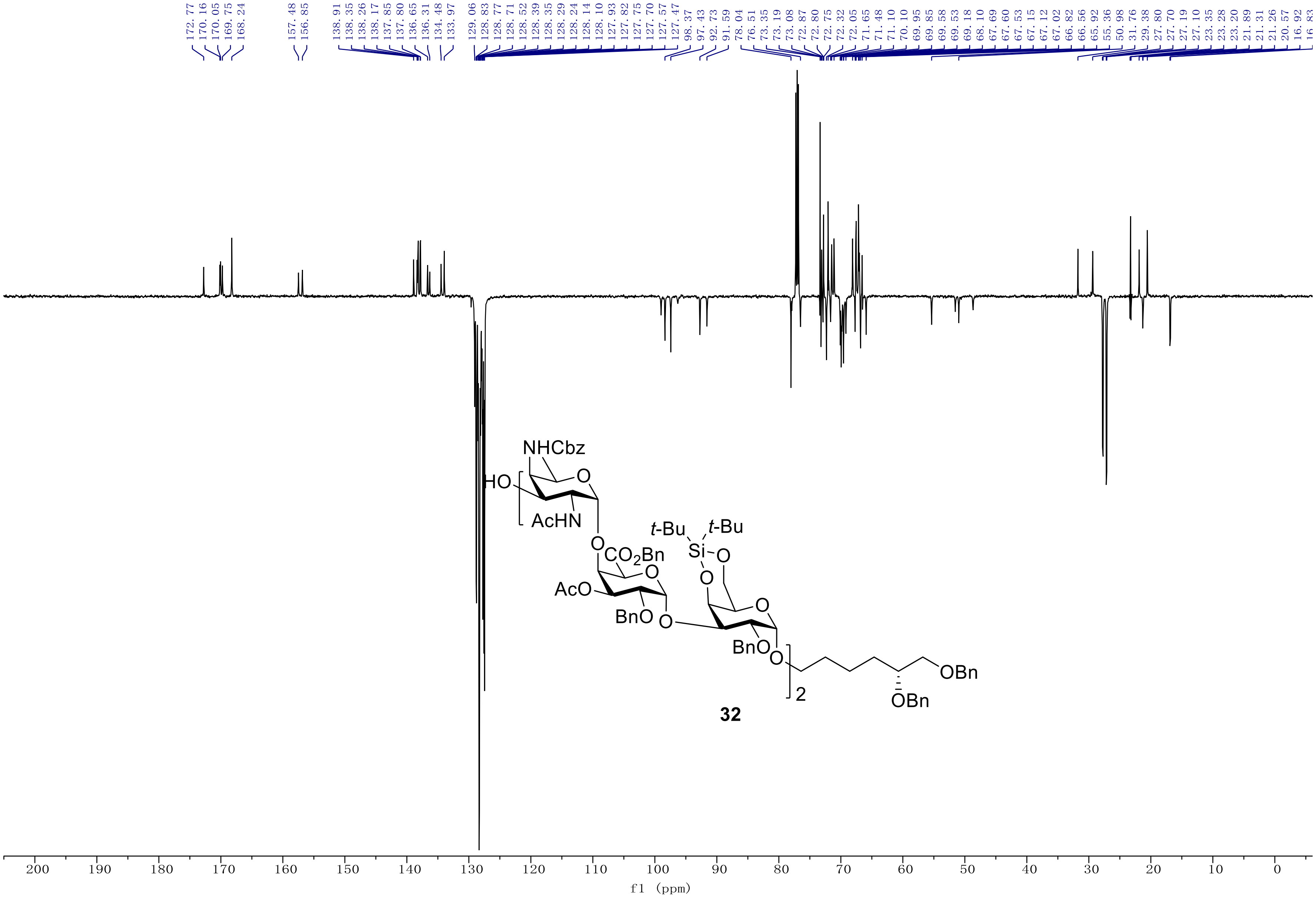

Biosyn022019Zhen.2.ser — 1H cosy, WZ 525-B in CDCl3 @ 298K

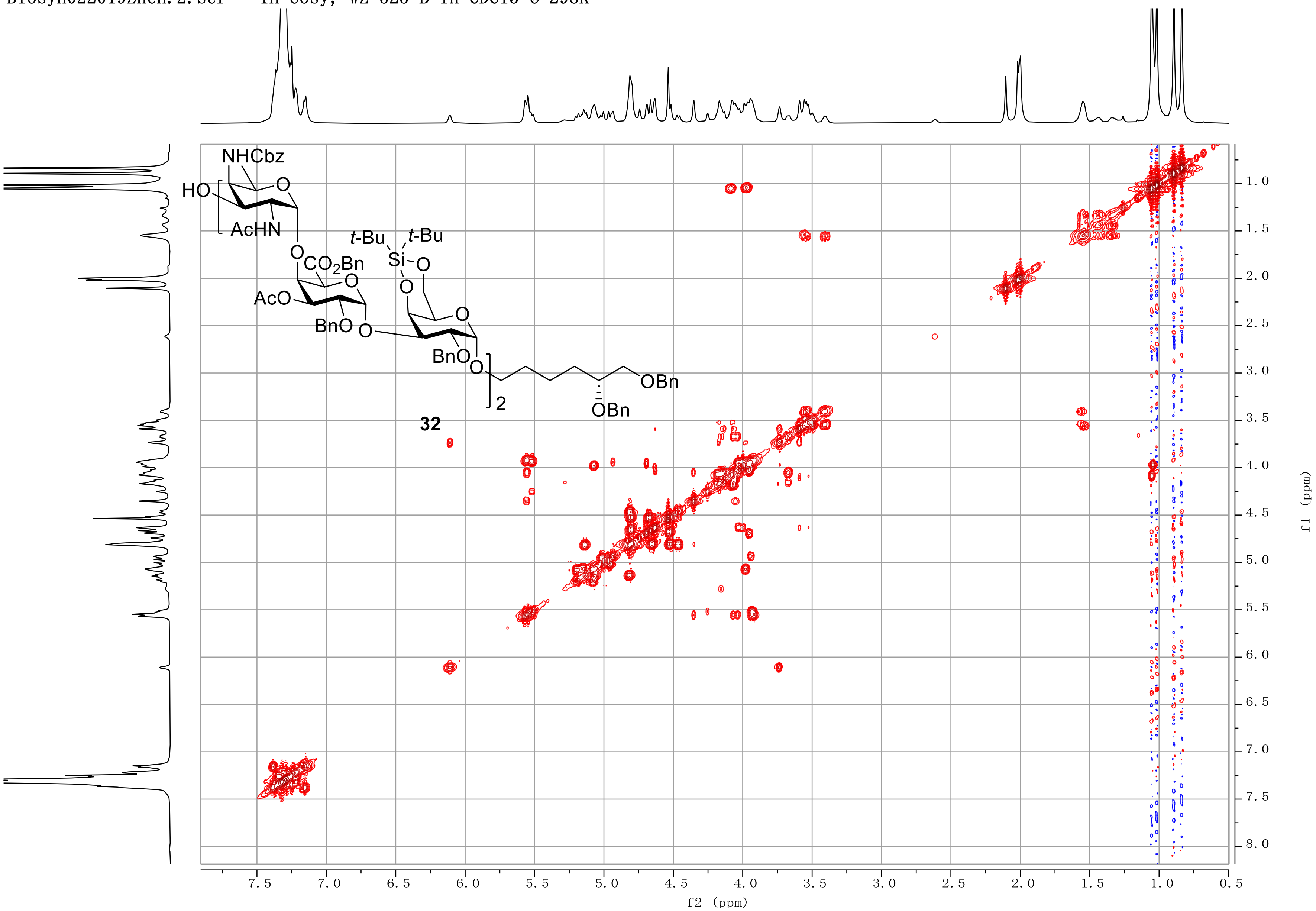

Biosyn022019Zhen.3.ser — hsqc, WZ 525-B in CDCl3 @ 298K

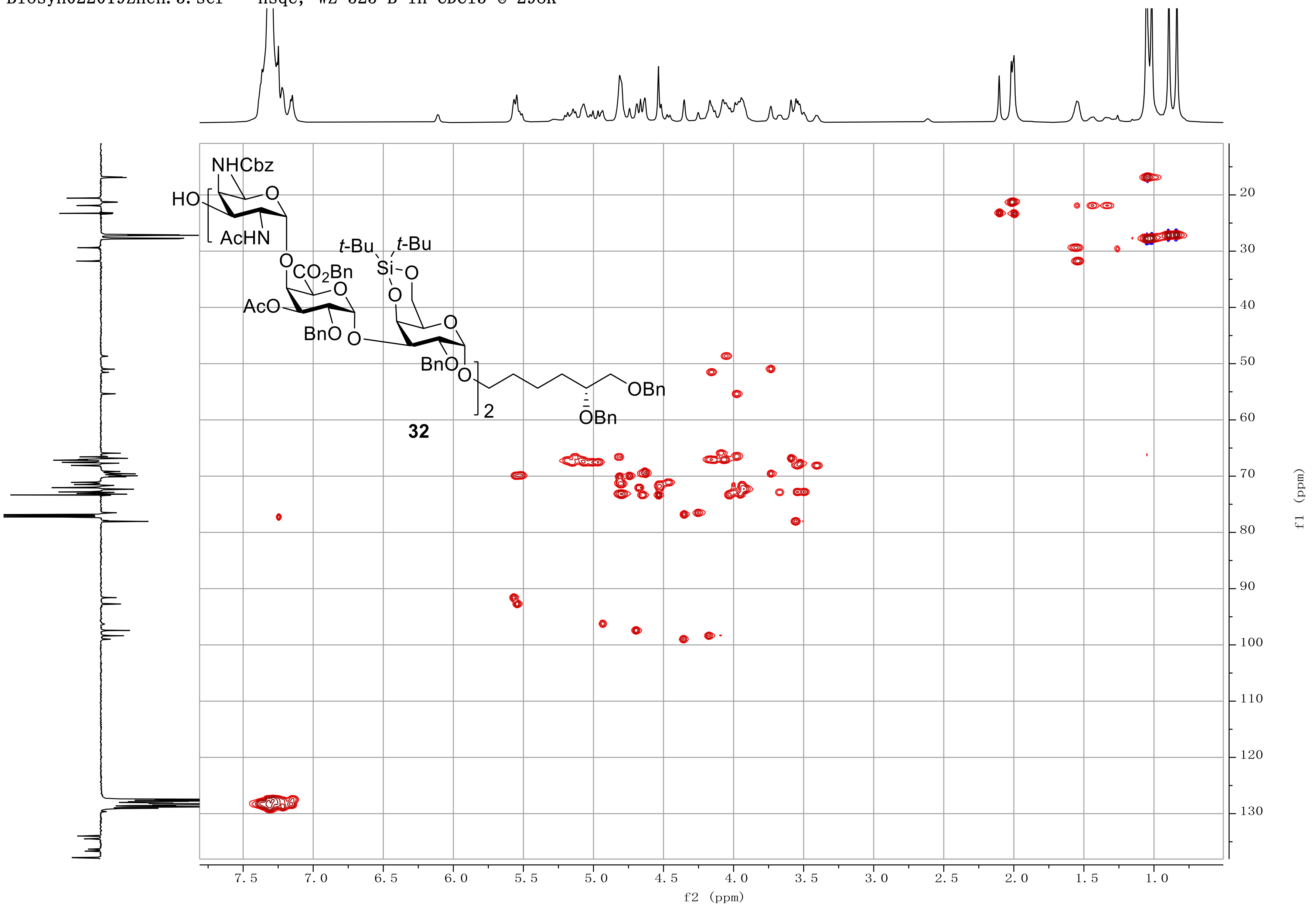

Biosyn022019Zhen.8.ser — hmbcNEW; WZ 525-B in CDCl3 @ 298K — with more number of scans

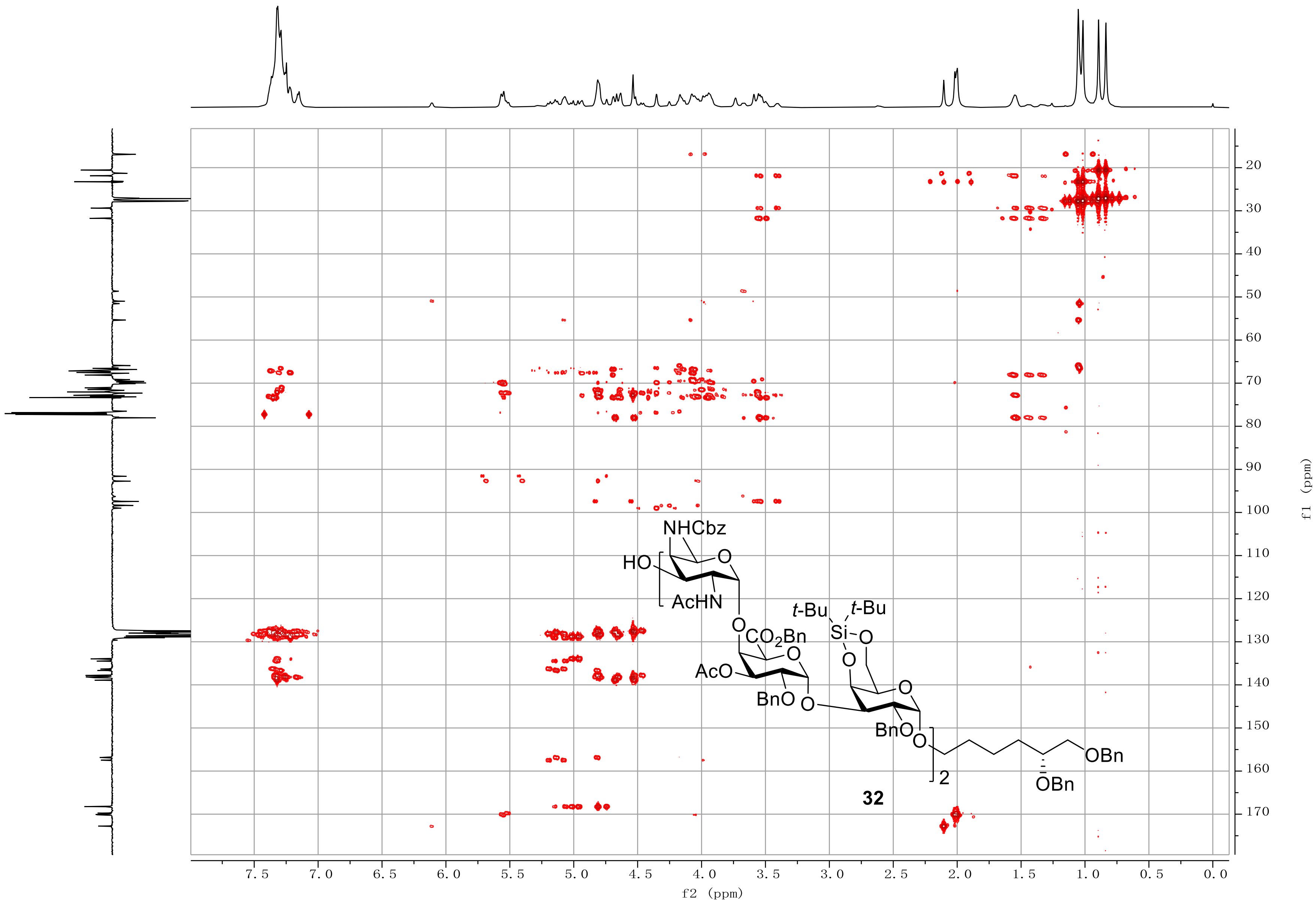

Biosyn022019Zhen.5.ser — c-hmbc for direct 1H13C ipv GATED; WZ 525-B in CDCl3 @ 298K

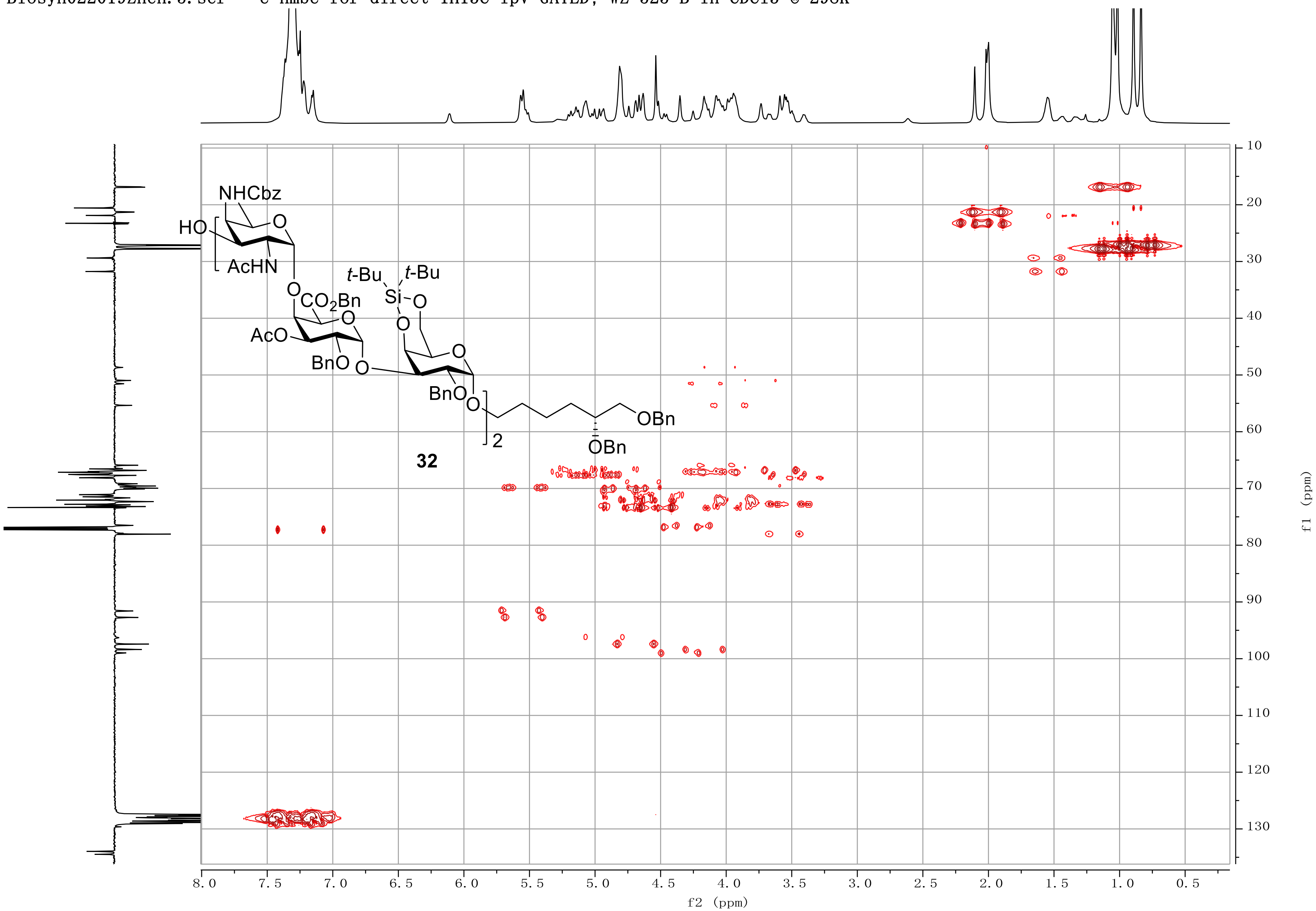

zhen1906biosyn.45.fid — wz526-c, size, 98mg — bbo-h1 CDCl3 /opt/topspin2.1 nmrafd 5

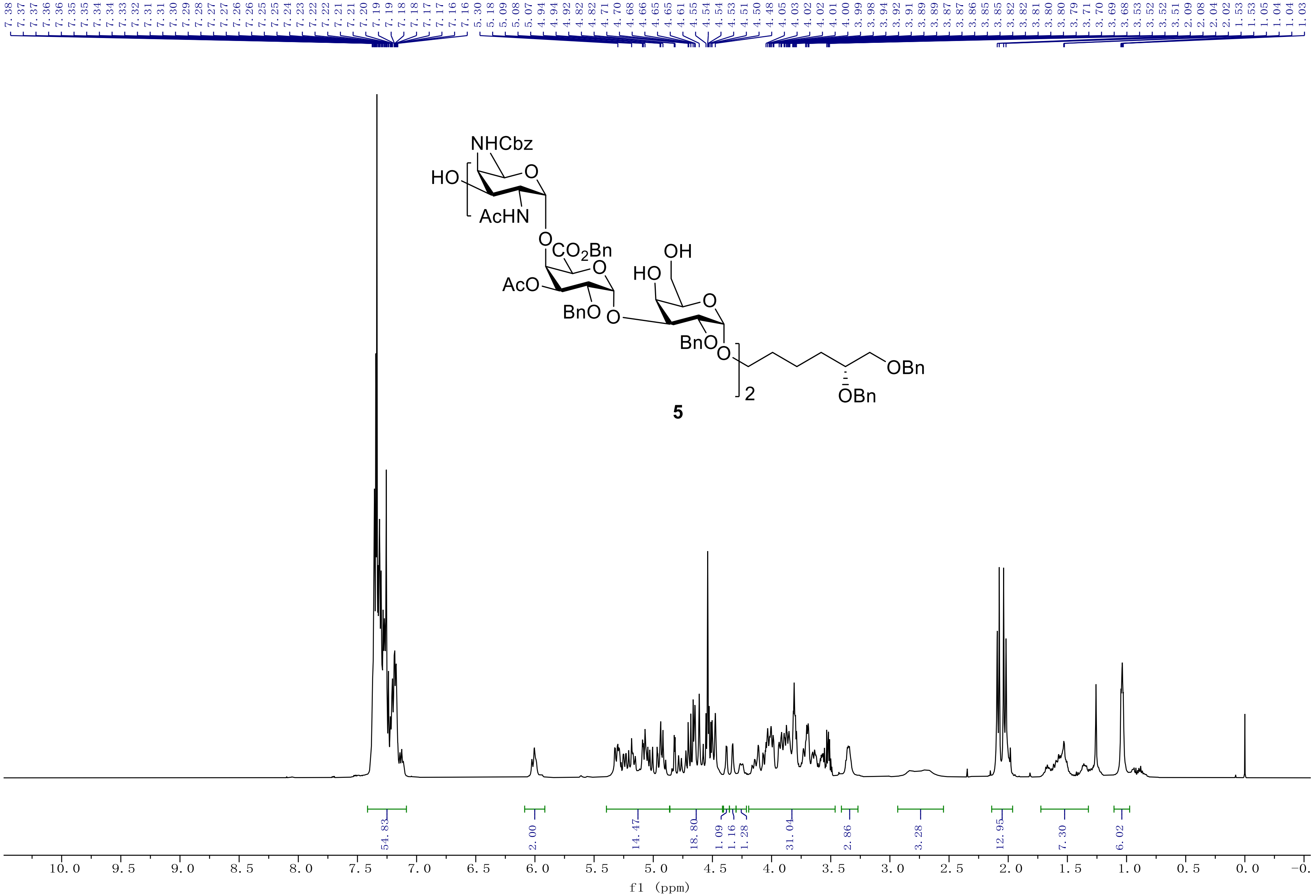

zhen1906biosyn.48.fid — wz526-c, size, 98mg — bbo-c13-APT CDCl3 /opt/topspin2.1 nmrafd 5

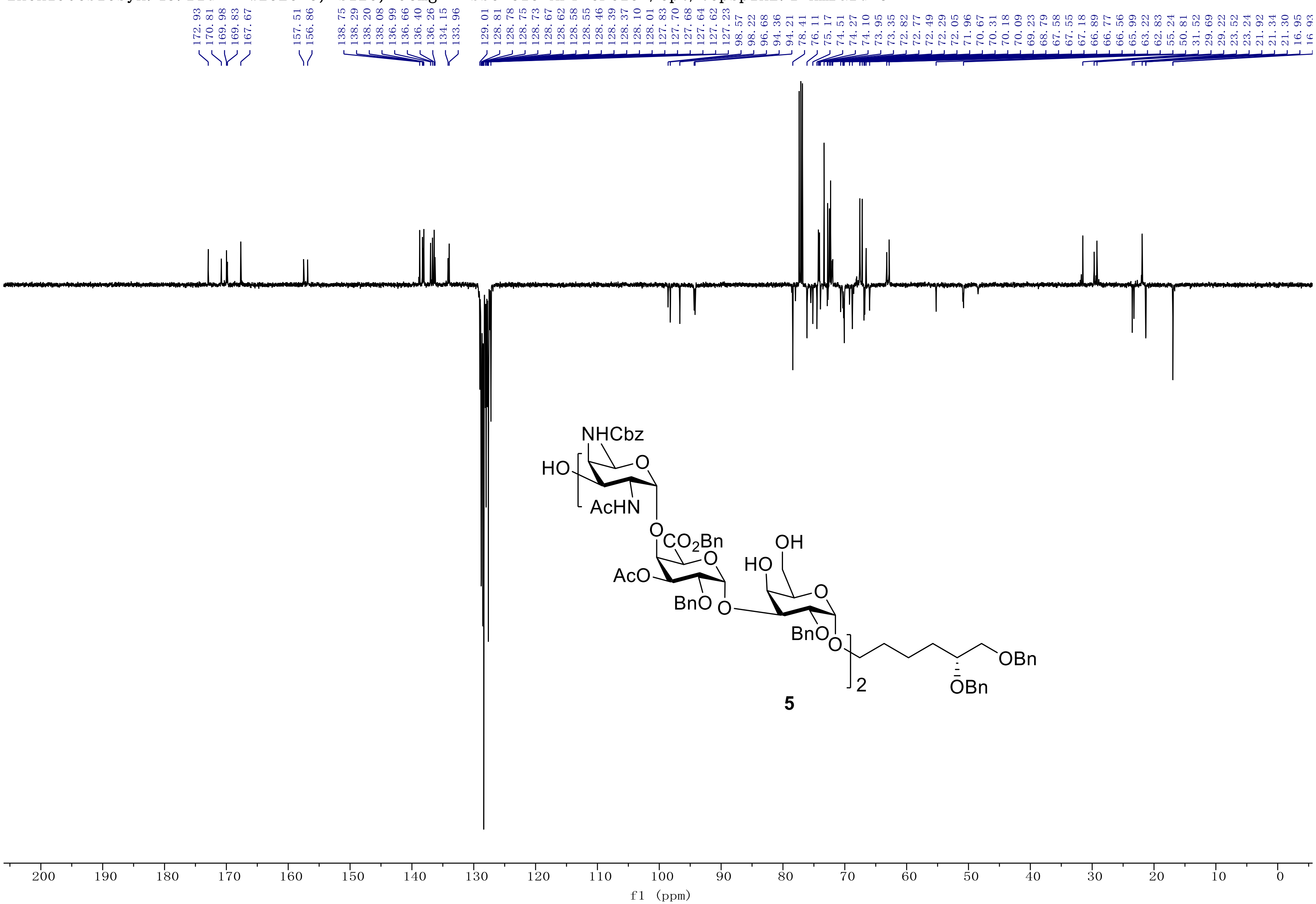

zhen1906biosyn.46.ser — wz526-c, size, 98mg — bbo-h1-cosy CDCl3 /opt/topspin2.1 nmrafd 5

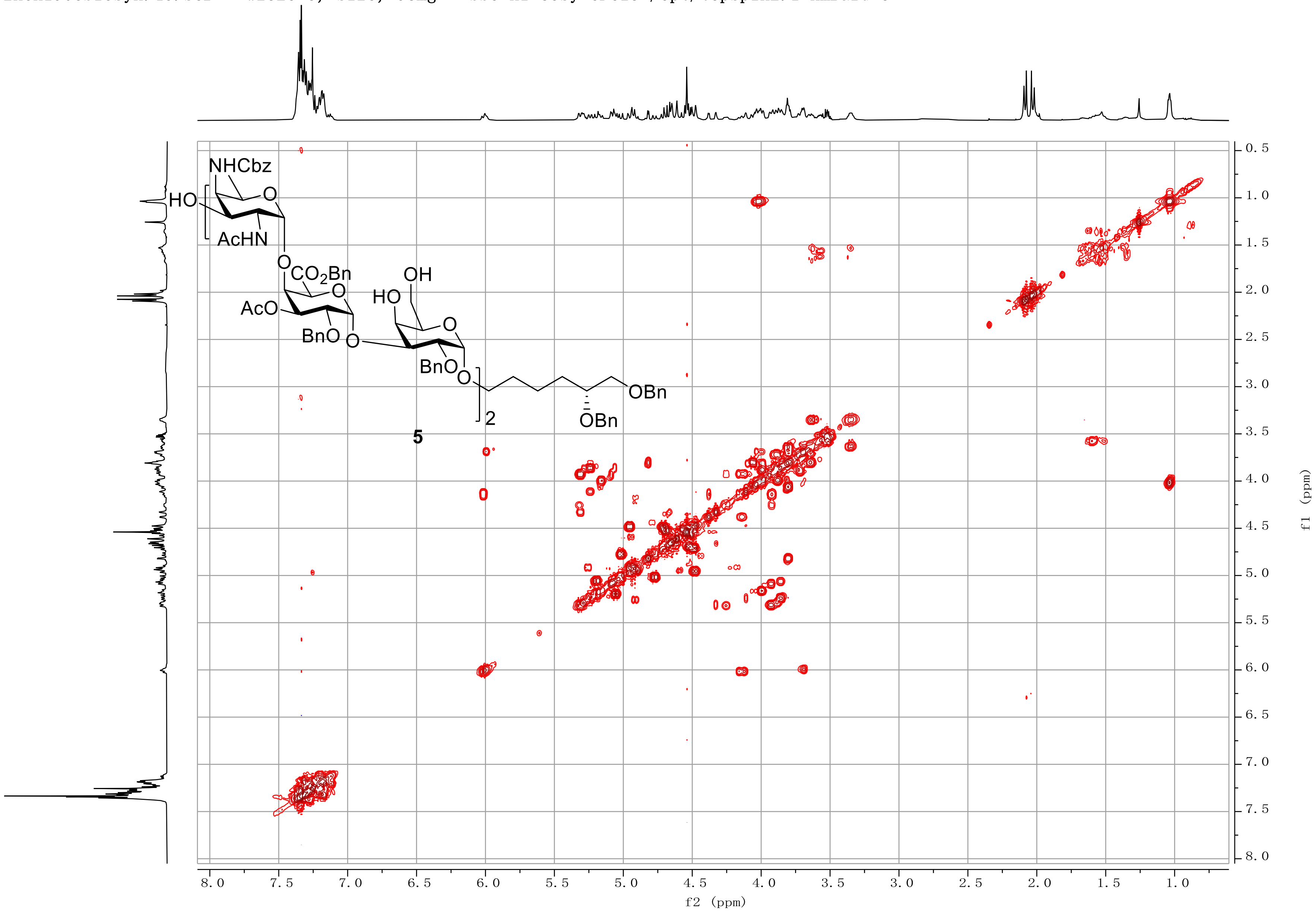

zhen1906biosyn.47.ser — wz526-c, size, 98mg — bbo-c13-HSQC CDCl3 /opt/topspin2.1 nmrafd 5

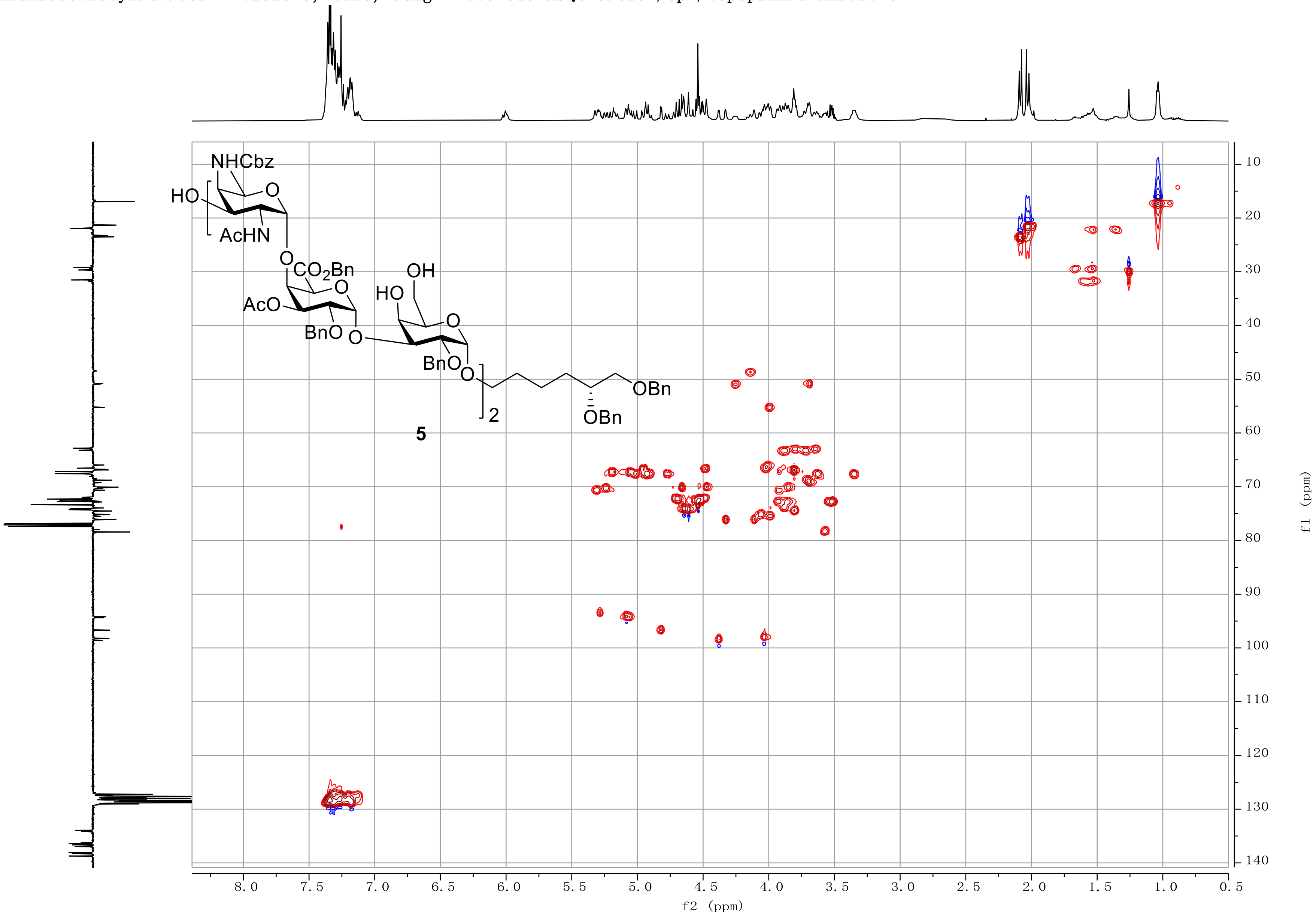

zhen1906biosyn.49.ser — wz526-c, size, 98mg — bbo-c13-HMBC CDCl3 /opt/topspin2.1 nmrafd 5

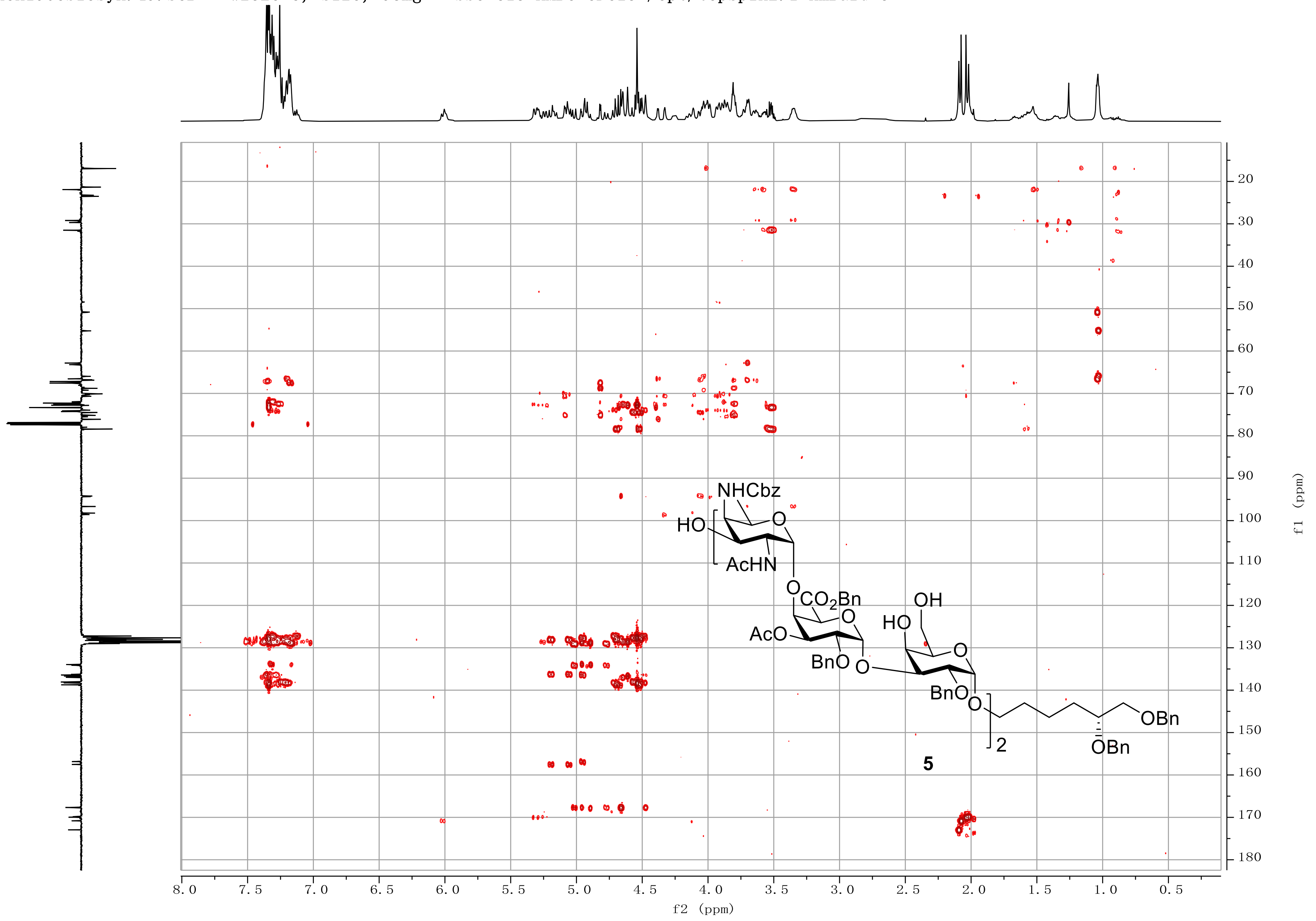

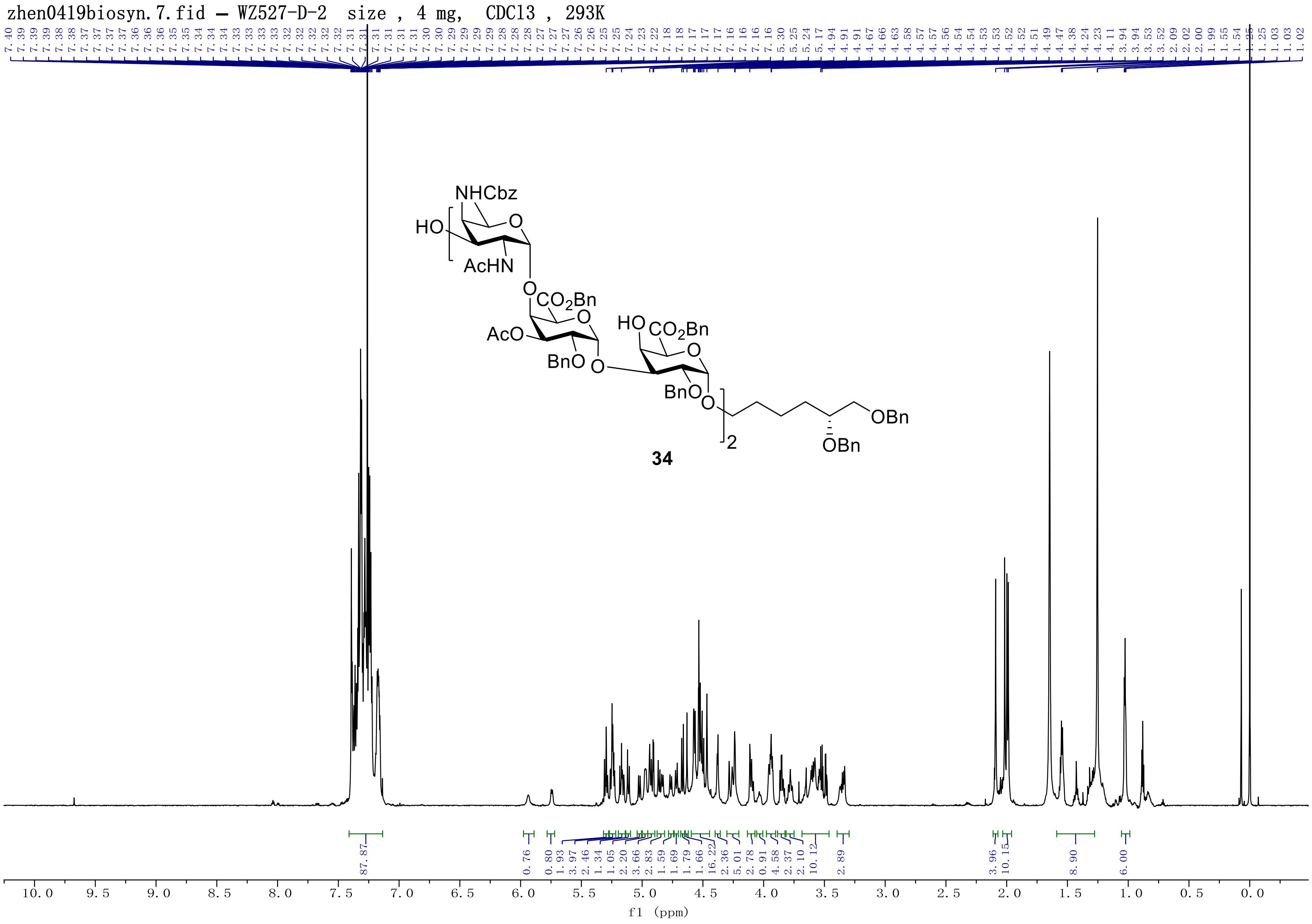
zhen0419biosyn.7.fid — WZ527-D-2 size , 4 mg, CDCl3 , 293K
NHCbz
HO
AcHN
CO2Bn
AcO
BnO
HO
CO2Bn
BnO
2
OBn
OBn
34
f1 (ppm)

zhen0419biosyn.13.fid — WZ527-D-2 size , 4 mg, CDCl3 , 293K , APT

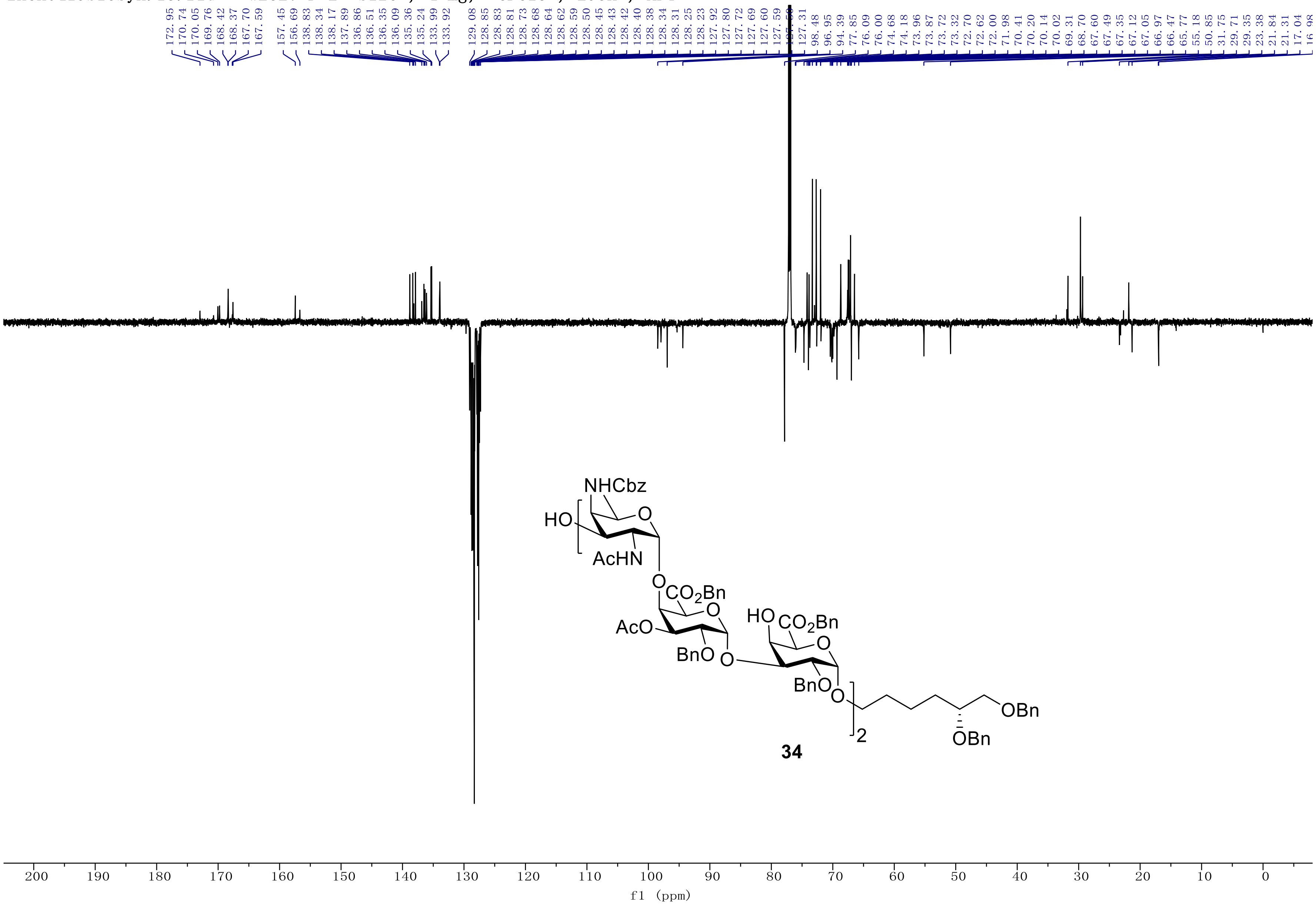

34

zhen0419biosyn.8.ser — WZ527-D-2 size , 4 mg, CDCl3 , 293K, cosy

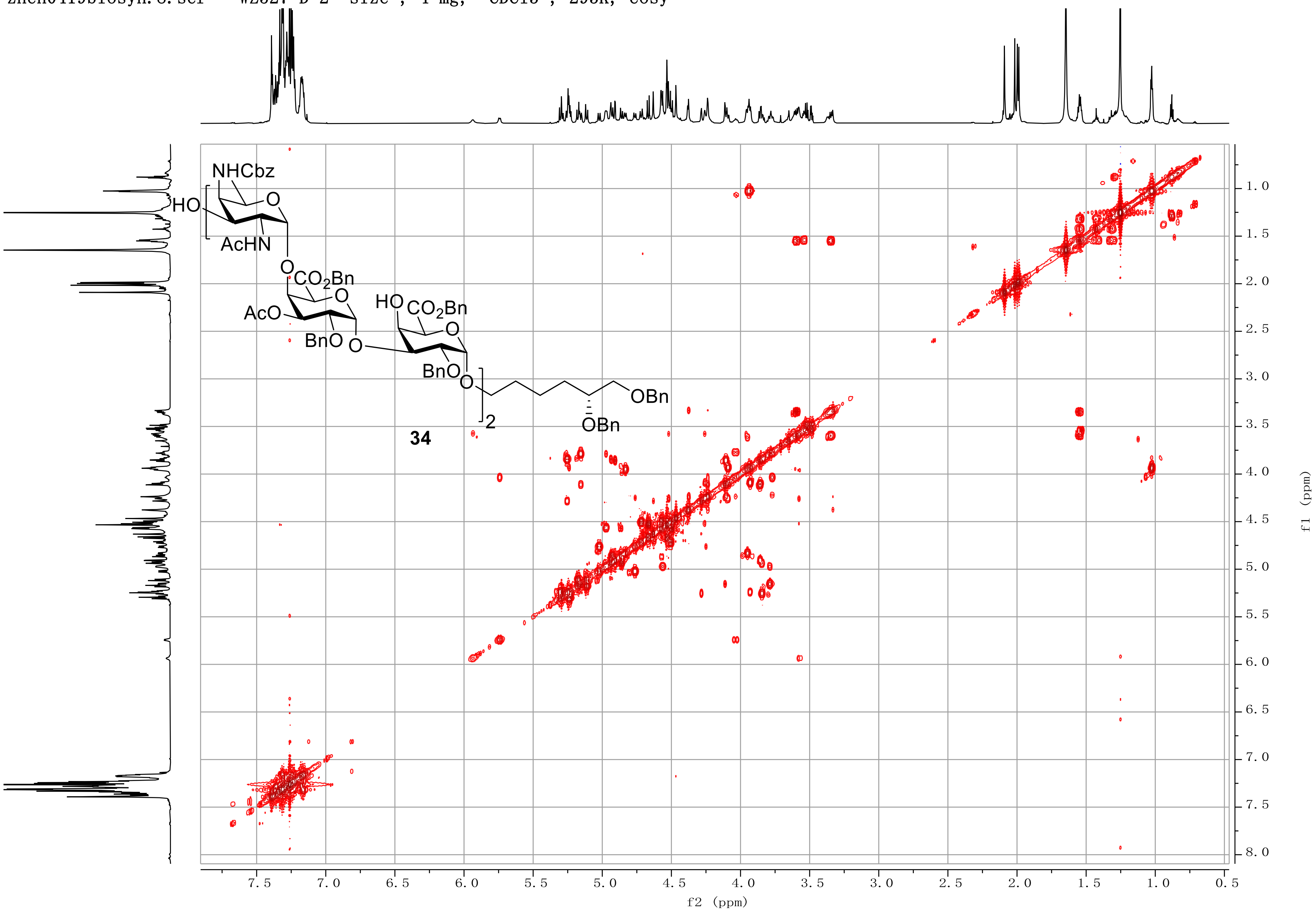

zhen0419biosyn.9.ser — WZ527-D-2 size , 4 mg, CDCl3 , 293K, hsqc

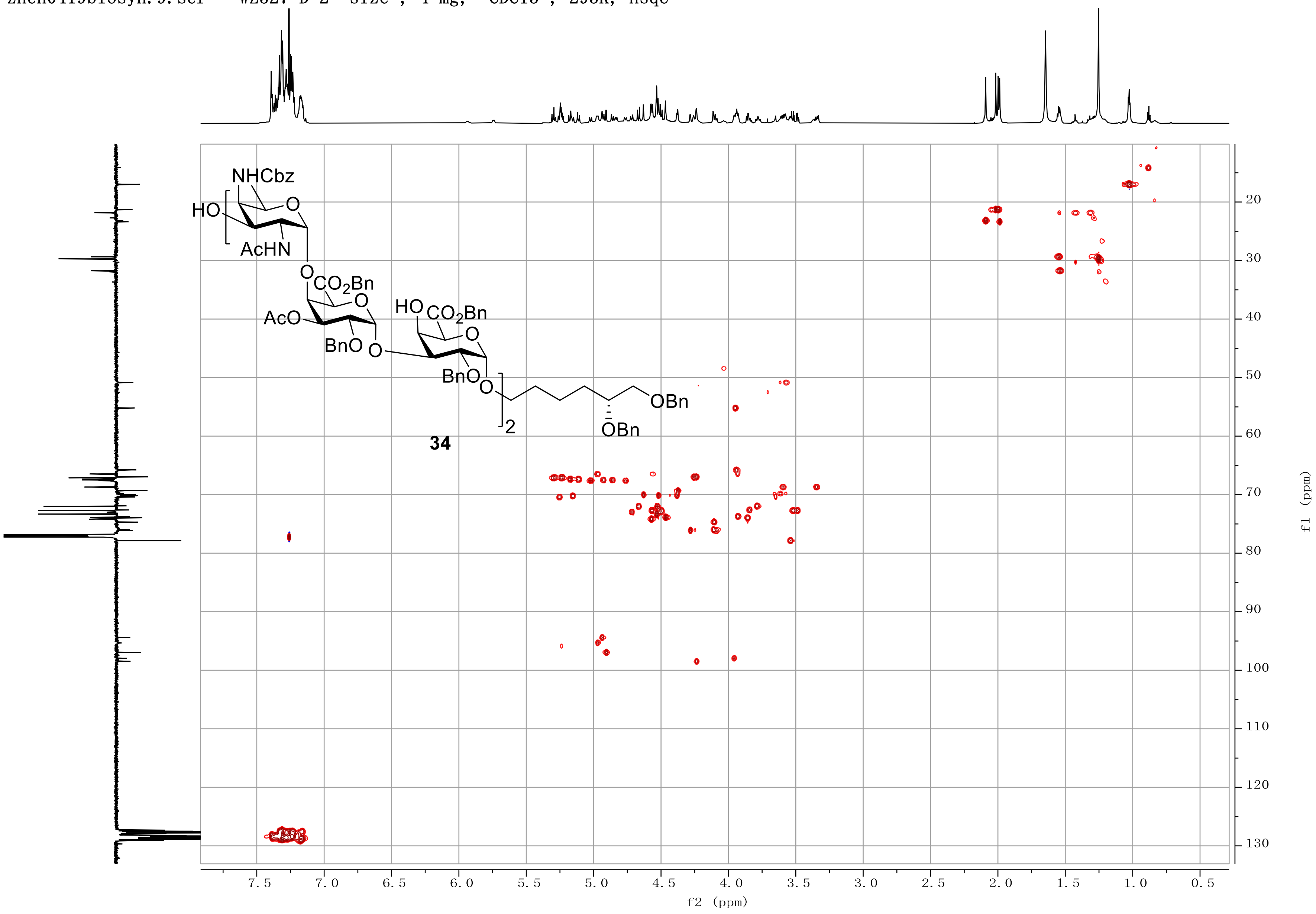

zhen0419biosyn.11.ser — WZ527-D-2 size , 4 mg, CDCl3 , 293K , c-hmbc

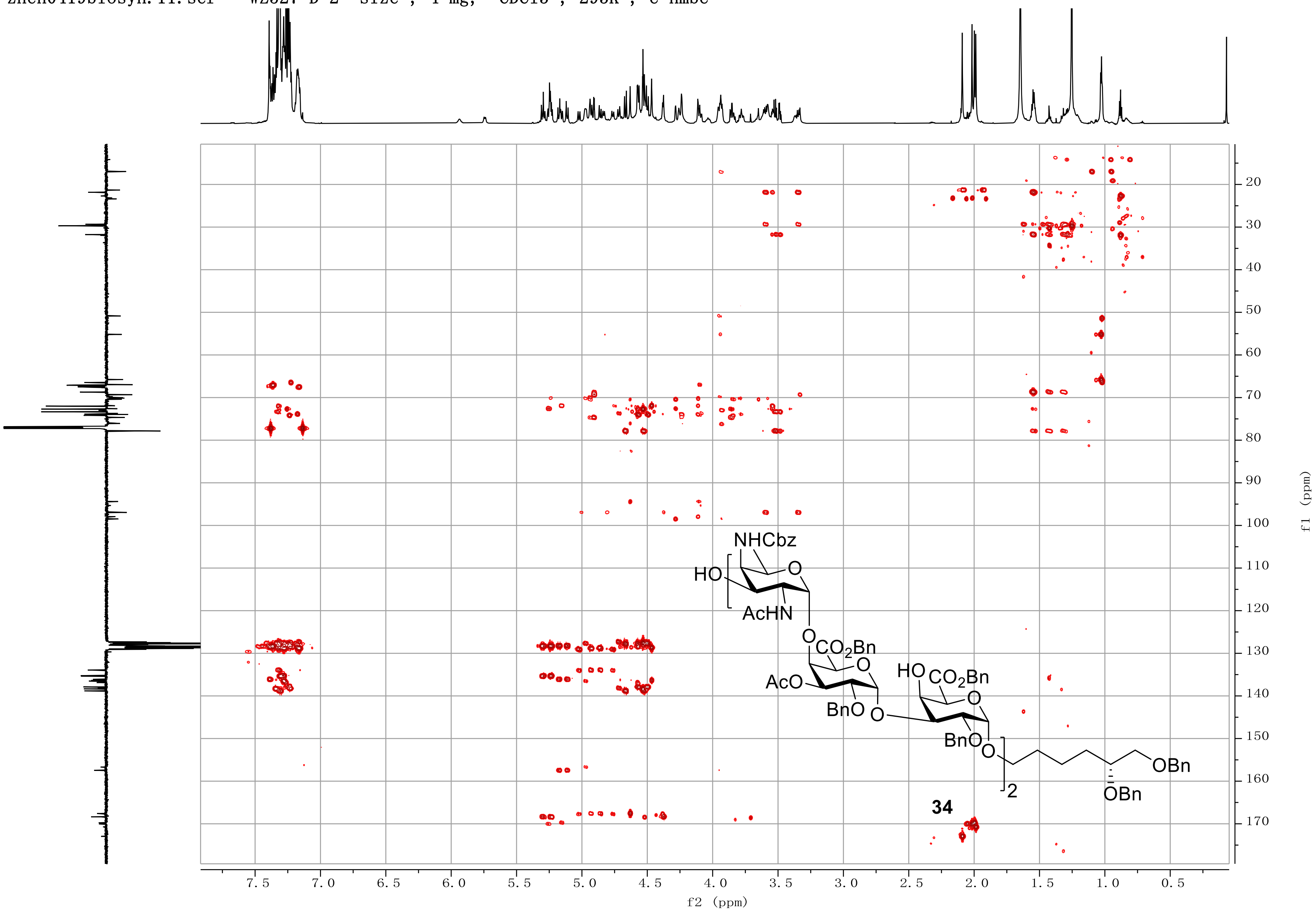

zhen0419biosyn.12.ser — 1H-13C, HMBC-gated-for-Direct-coupling; av850 , WZ527-D-2 size , 4 mg, CDCl3 , 293K

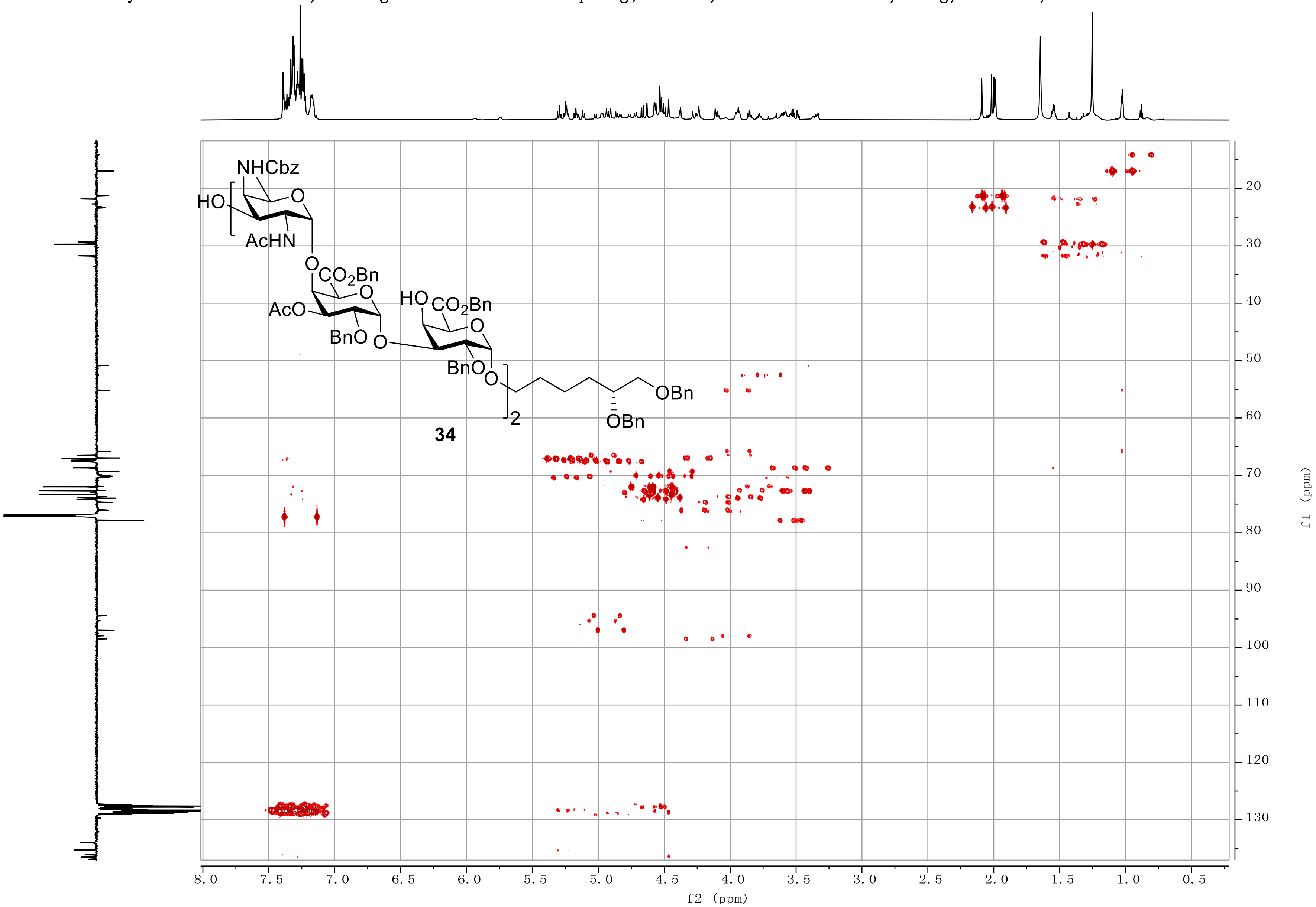

zhen0419biosyn.1.fid — WZ527-C-2 in CDCl3 @ 298K — h1-protonzg30 CDCl3 /opt/topspin3.2 nmrafd 24

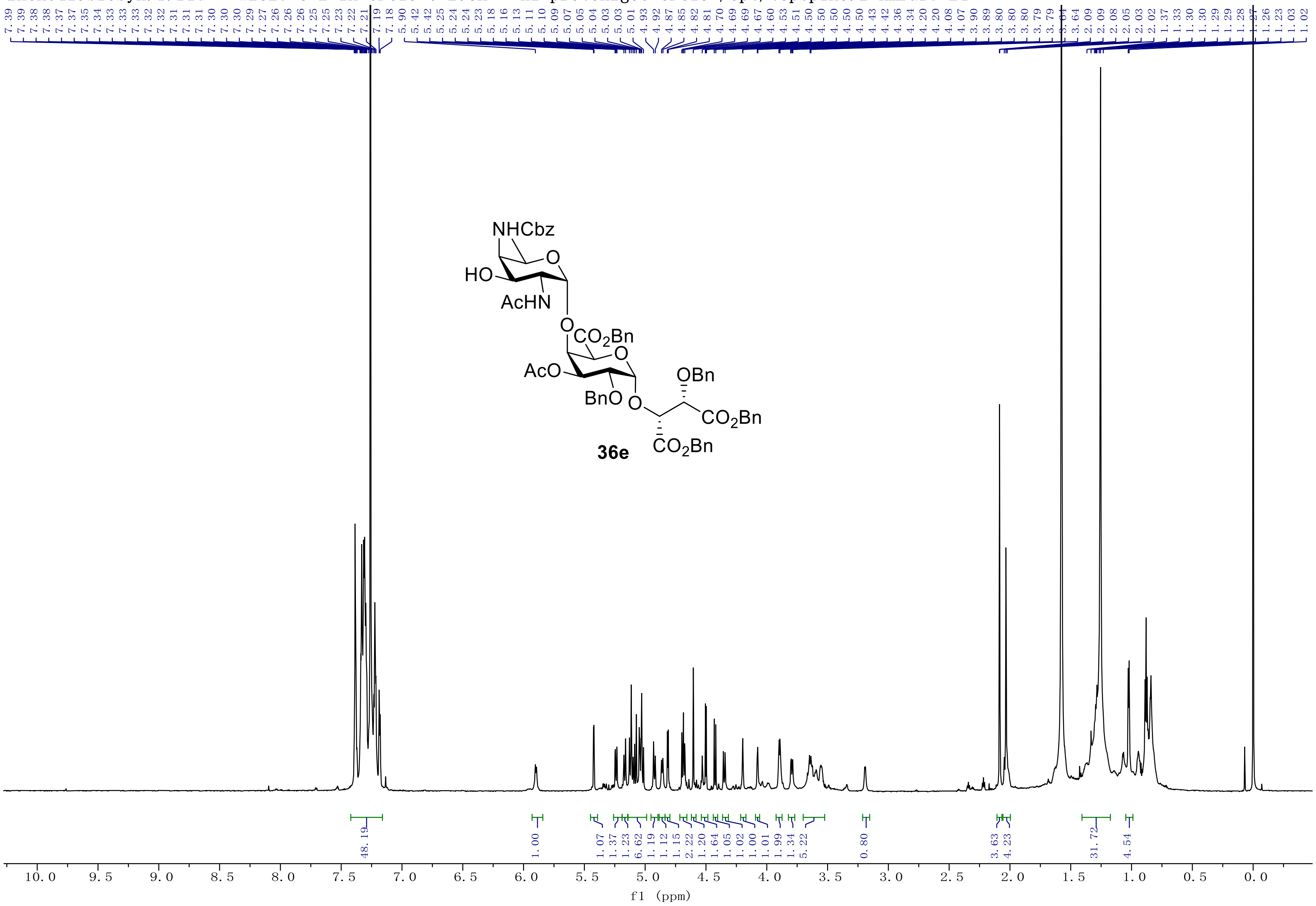

zhen0419biosyn.6.fid — WZ527-C-2 in CDCl3 @ 298K — c-APT CDCl3 /opt/topspin3.2 nmrafd 24

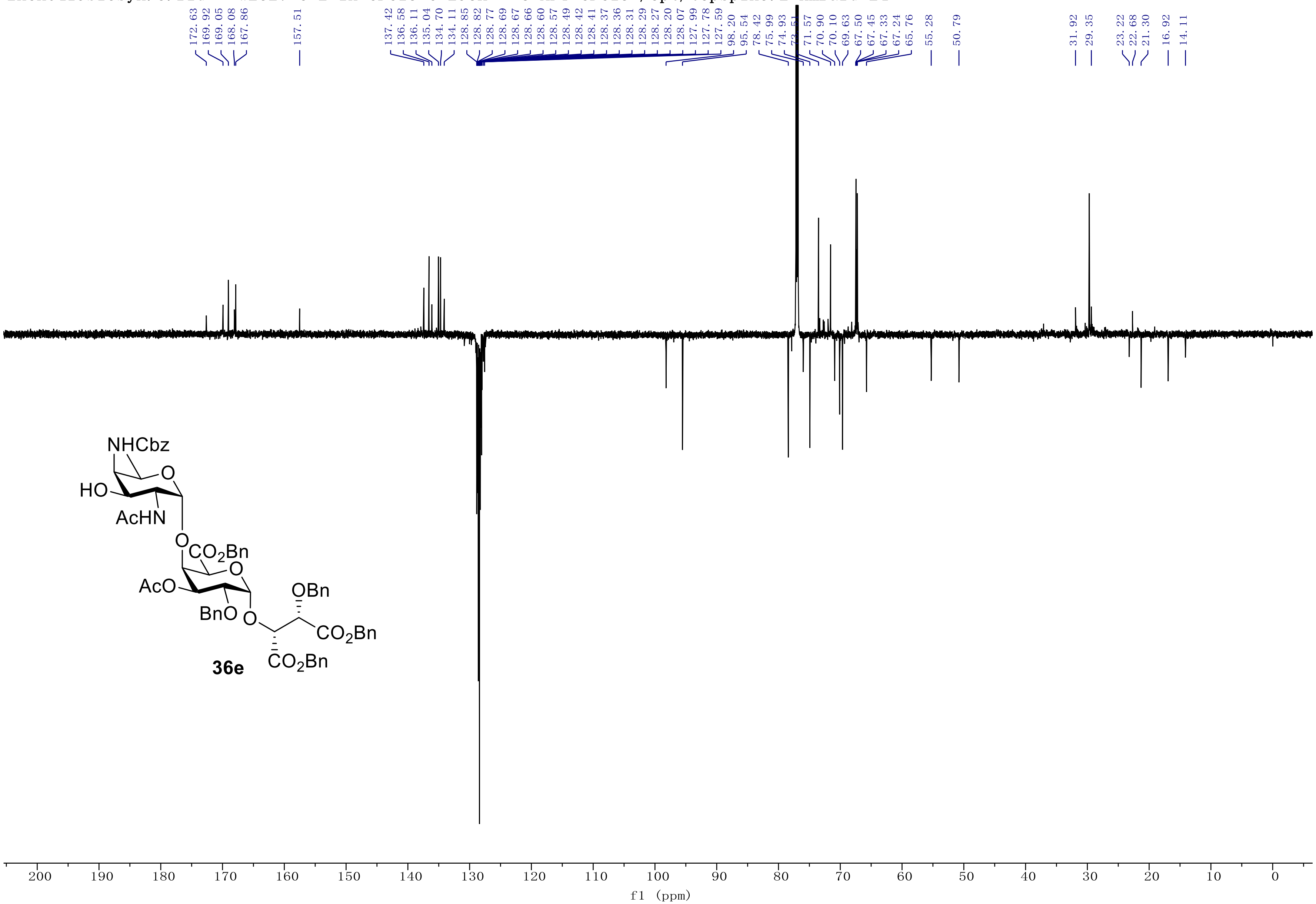

zhen0419biosyn.2.ser — WZ527-C-2 in CDCl3 @ 298K — h1-cosygp CDCl3 /opt/topspin3.2 nmrafd 24

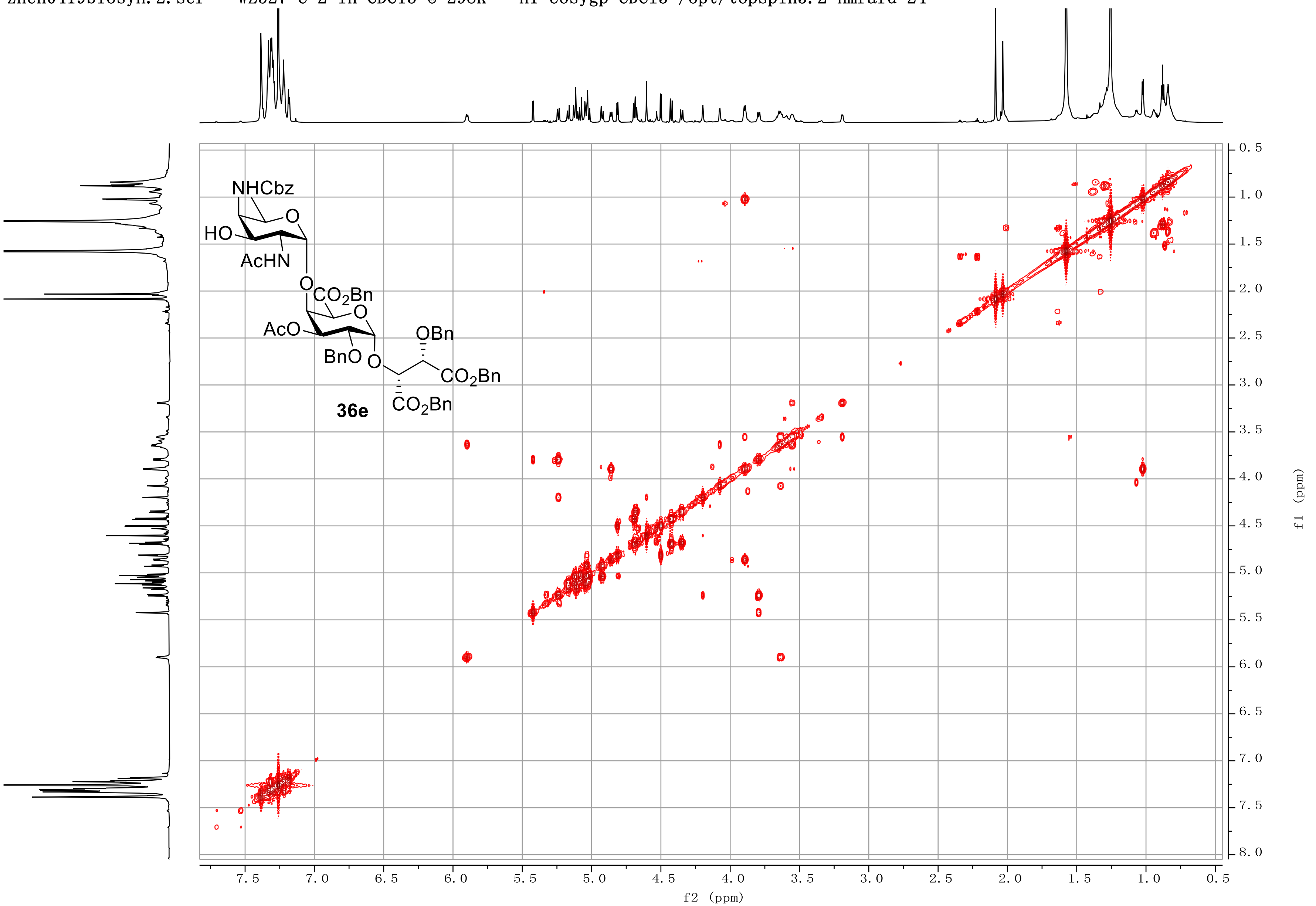

zhen0419biosyn.3.ser — WZ527-C-2 in CDCl3 @ 298K — c-hsqcgp CDCl3 /opt/topspin3.2 nmrafd 24

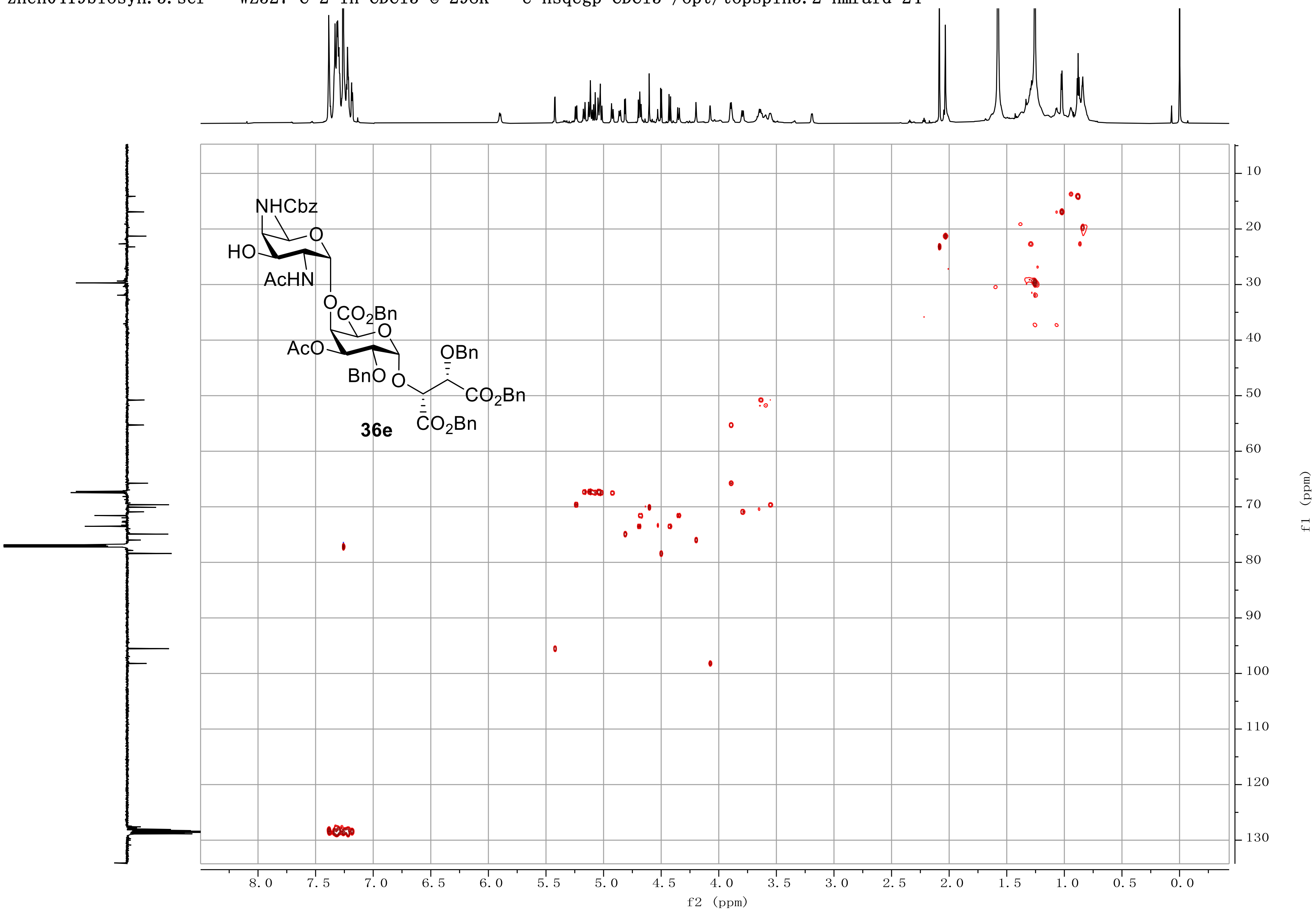

zhen0419biosyn.5.ser — WZ527-C-2 in CDCl3 @ 298K — c-hmbcgp CDCl3 /opt/topspin3.2 nmrafd 24

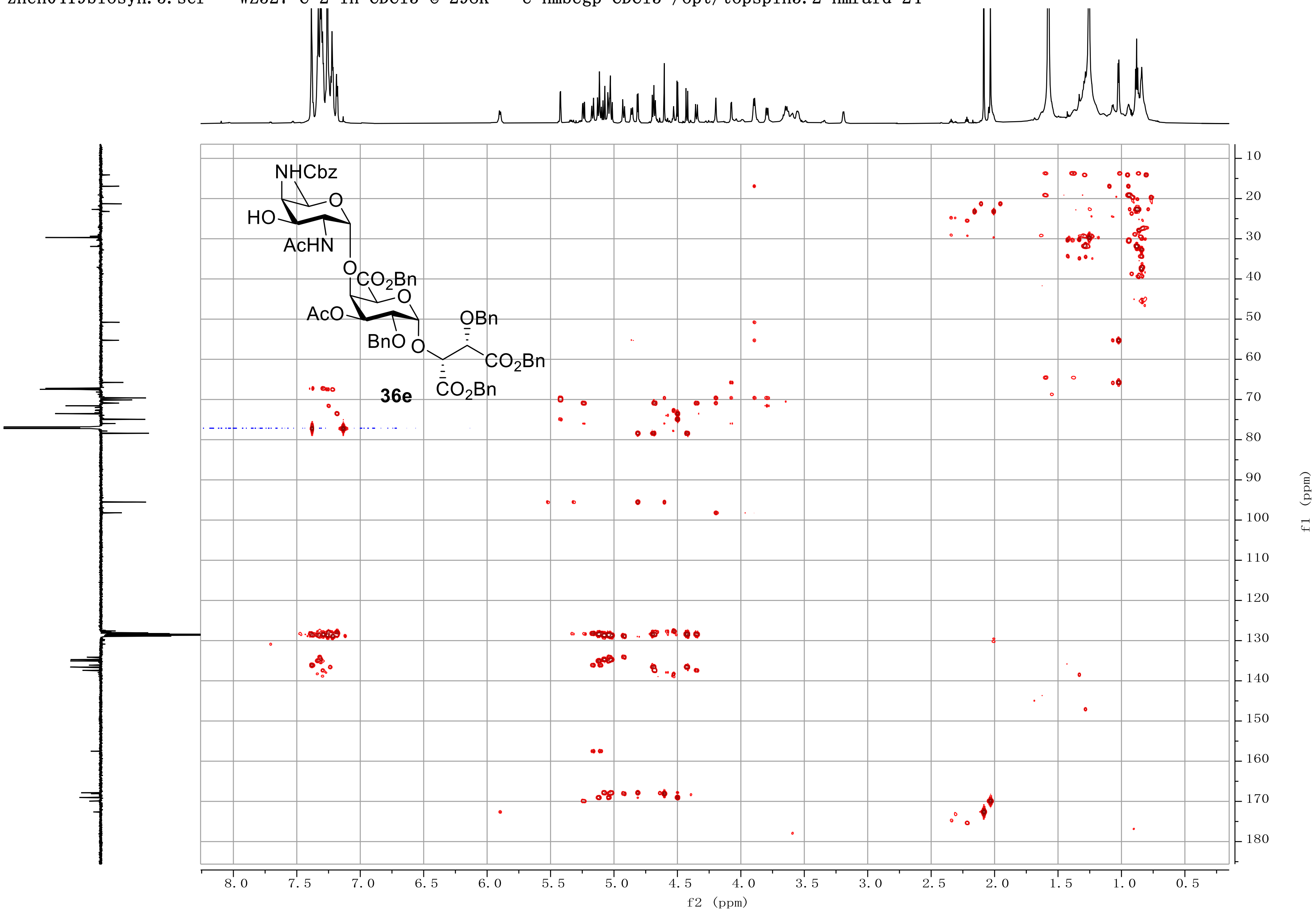

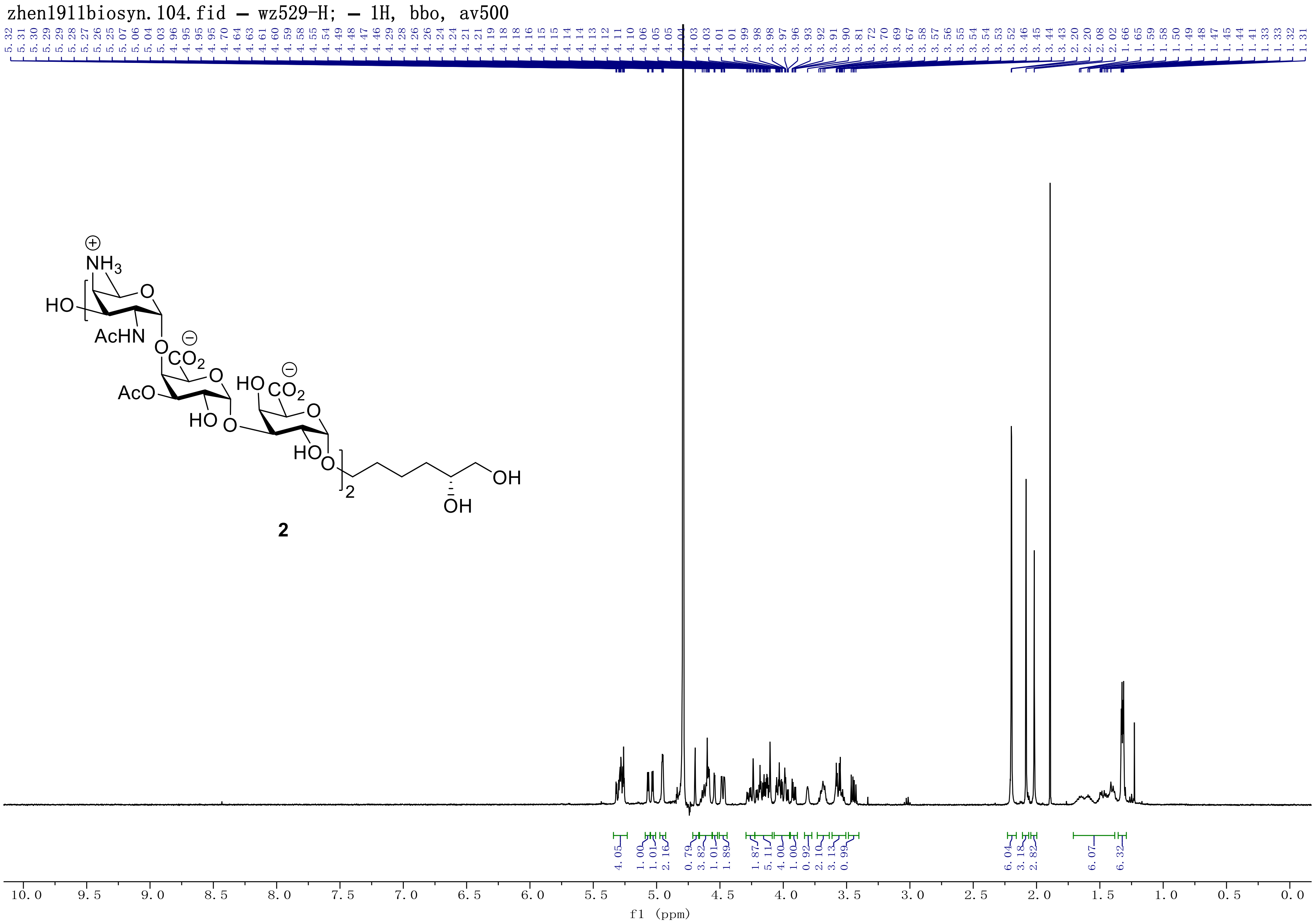

zhen1911biosyn.104.fid — wz529-H; — 1H, bbo, av500
5.32 5.31 5.30 5.29 5.29 5.28 5.27 5.26 5.25 5.07 5.06 5.04 5.03 4.96 4.95 4.95 4.70 4.64 4.63 4.61 4.60 4.59 4.58 4.55 4.54 4.49 4.48 4.47 4.46 4.29 4.28 4.26 4.24 4.24 4.21 4.19 4.18 4.16 4.15 4.15 4.14 4.13 4.12 4.11 4.10 4.06 4.05 4.05
4.03 4.03 4.01 4.01 3.99 3.98 3.98 3.97 3.96 3.93 3.92 3.91 3.90 3.81 3.72 3.70 3.69 3.67 3.58 3.57 3.56 3.55 3.54 3.54 3.53 3.52 3.46 3.45 3.44 3.43 2.20 2.20 2.08 2.02 1.66 1.65 1.59 1.58 1.50 1.49 1.48 1.47 1.45 1.44 1.41 1.33 1.33 1.32 1.31
NH3
HO
AcHN
CO2
AcO
HO
HO
CO2
HO
2
OH
OH
2
4.05 1.00 1.01 2.16 0.79 3.82 1.01 1.89 1.87 5.11 4.00 1.00 0.92 2.10 3.13 0.99 6.04 3.18 2.82 6.07 6.32
10.0 9.5 9.0 8.5 8.0 7.5 7.0 6.5 6.0 5.5 5.0 4.5 4.0 3.5 3.0 2.5 2.0 1.5 1.0 0.5 0.0
f1 (ppm)

zhen1119biosyn.17.fid — wz-529-G 3.6mg in D2O @ 293K — stopped at 09:30, from 02:15, run 7h 15min.

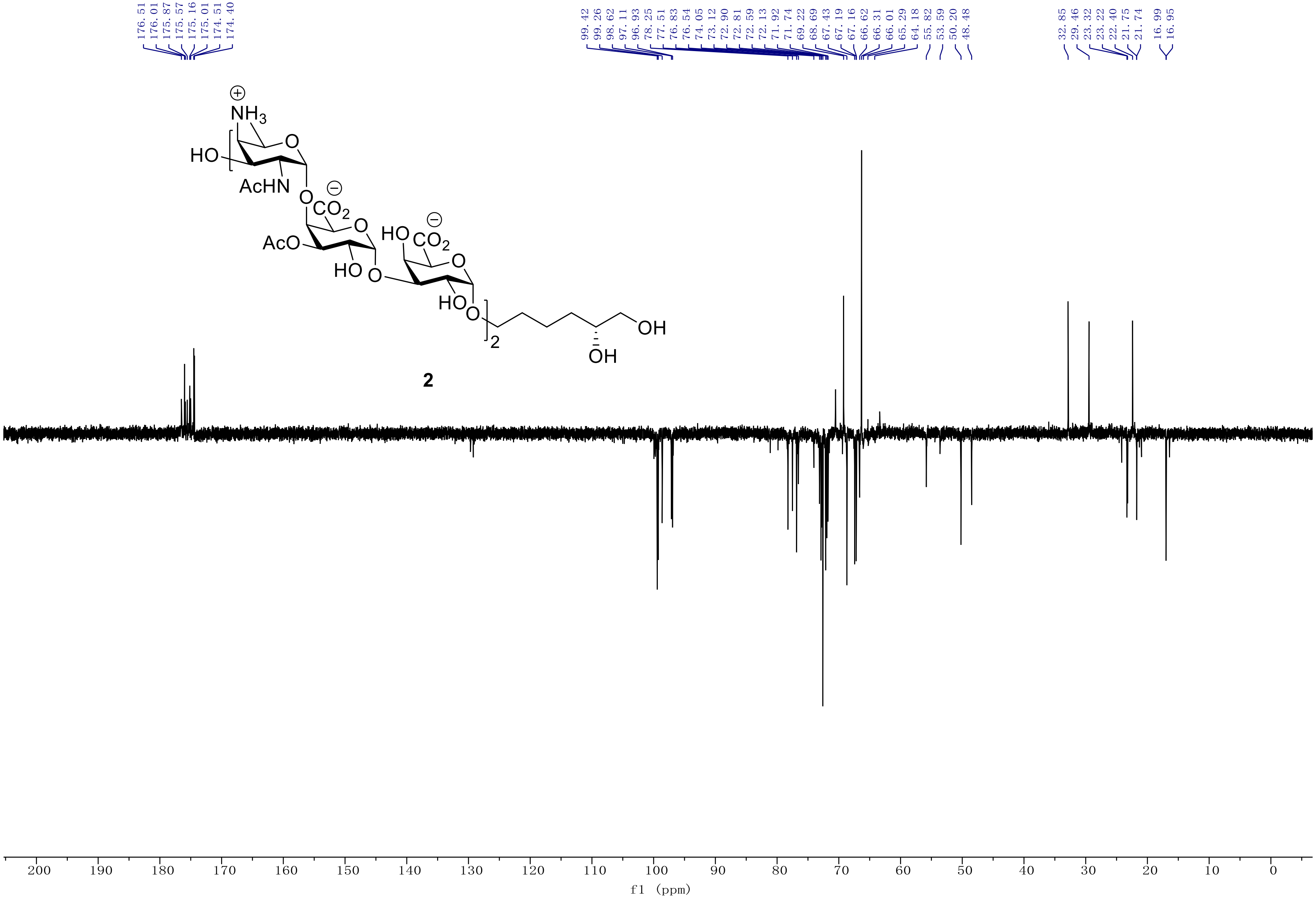

zhen1119biosyn.13.ser — wz-529-G 3.6mg in D2O @ 293K

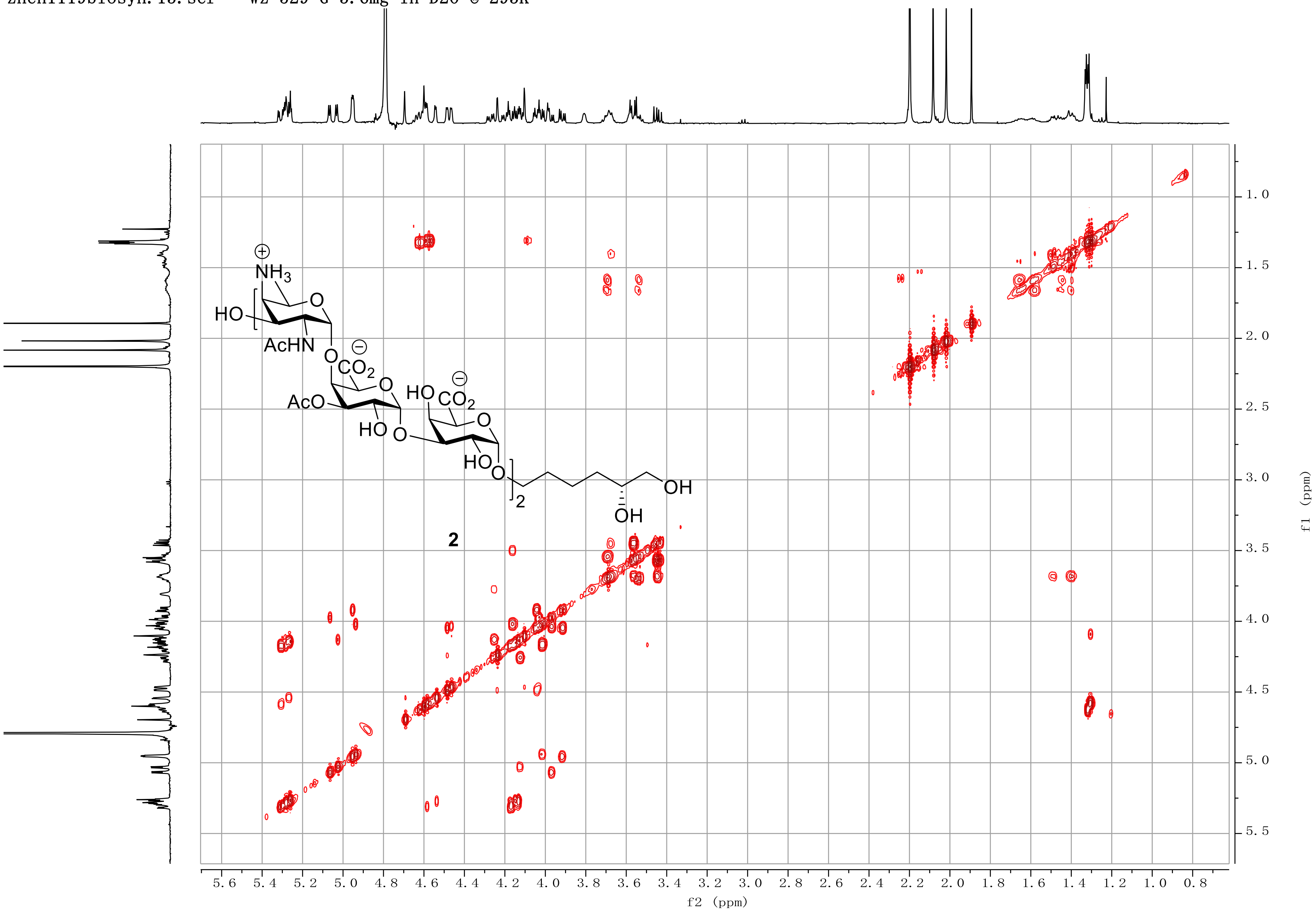

zhen1119biosyn.14.ser — wz-529-G 3.6mg in D2O @ 293K

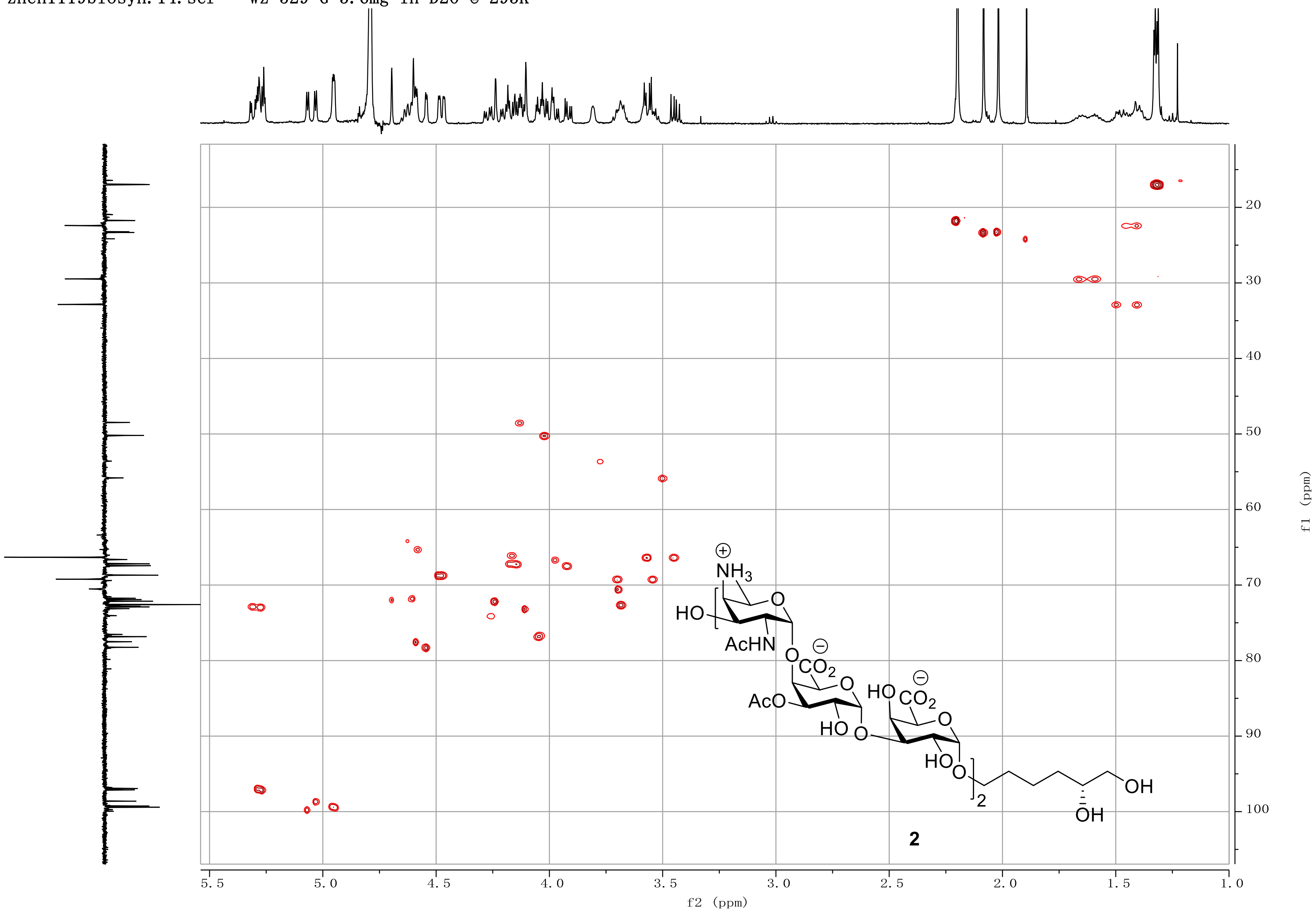

2

1906zhen.7.fid — wz530-Pure — h1 CDCl3 /opt/DATA nmrafd 9

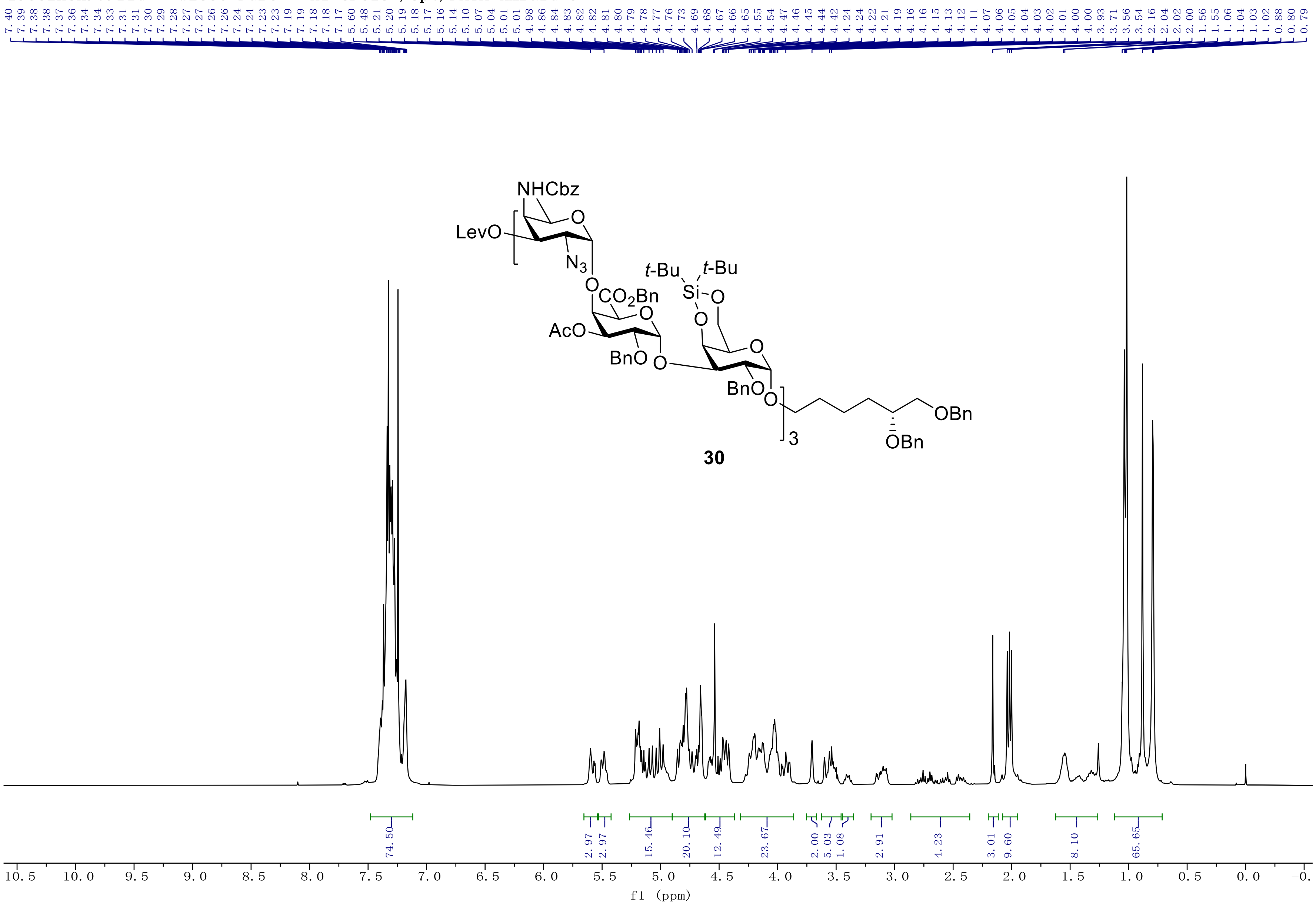

1906zhen.8.fid — wz530-Pure — C13APT CDCl3 /opt/DATA nmrafd 9

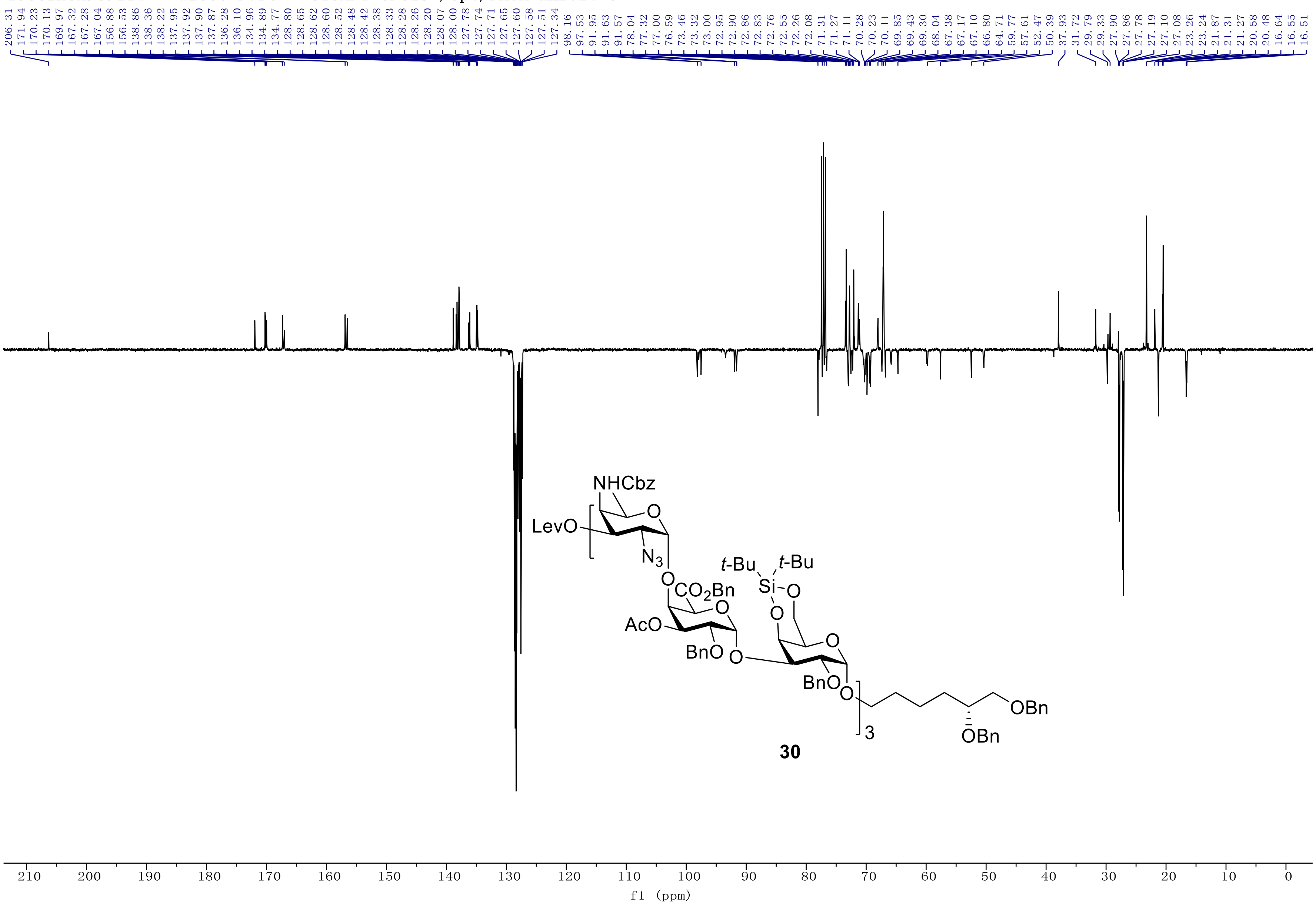

1906zhen.9.ser — wz530-Pure — h1COSY CDCl3 /opt/DATA nmrafd 9

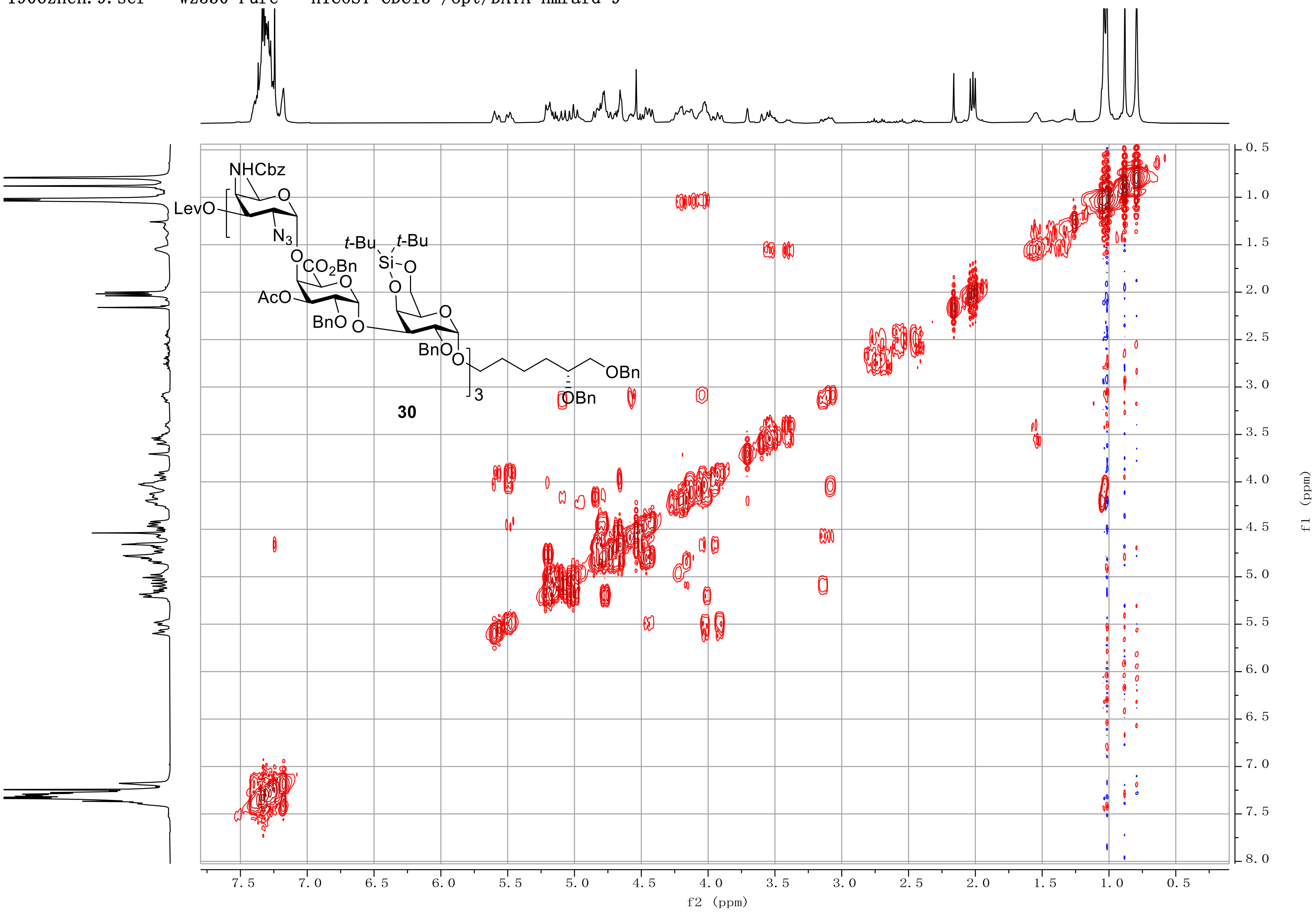

1906zhen.10.ser — wz530-Pure — c13HSQC CDCl3 /opt/DATA nmrafd 9

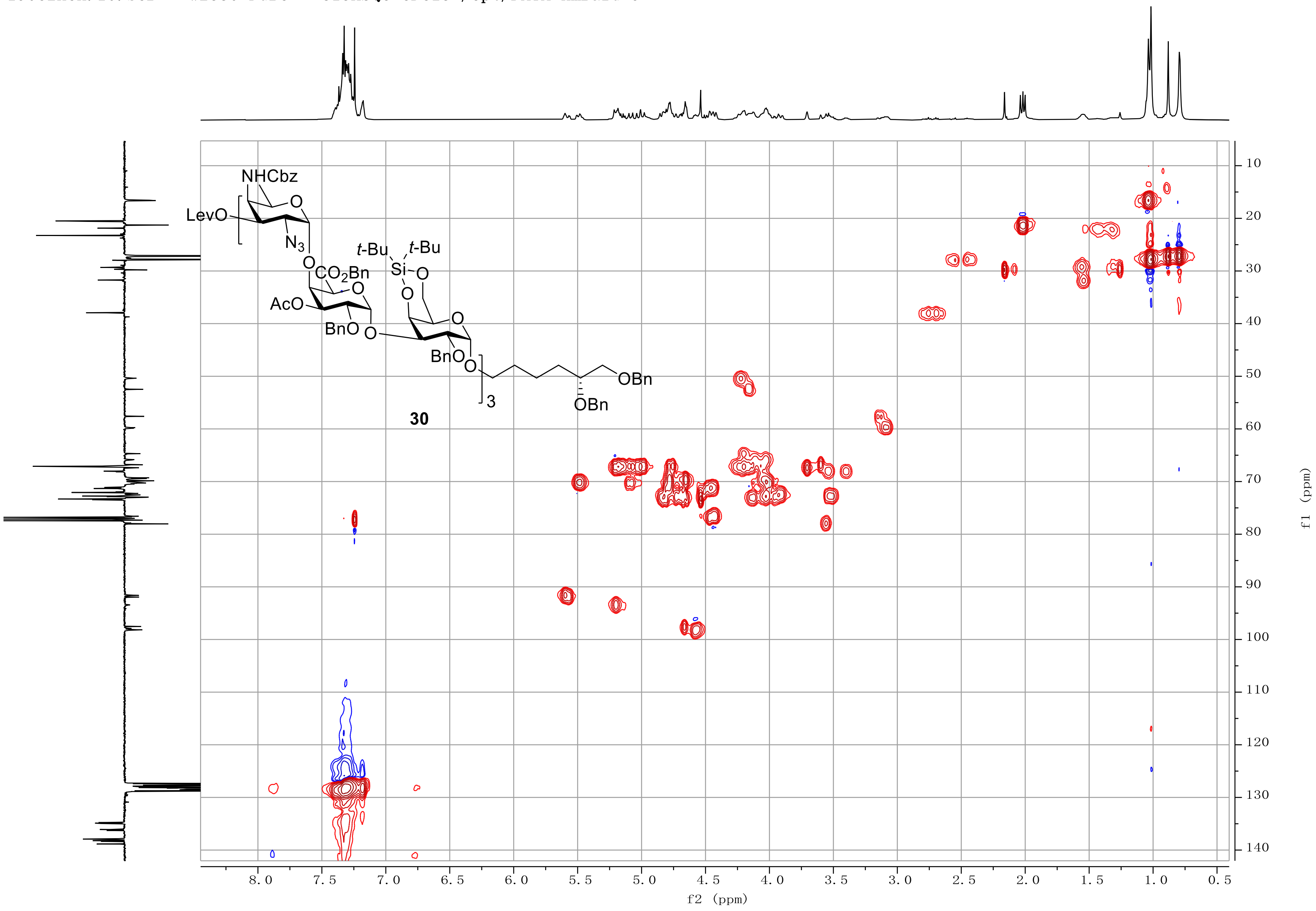

1906zhen.11.ser — wz530-Pure — c13HMBC CDCl3 /opt/DATA nmrafd 9

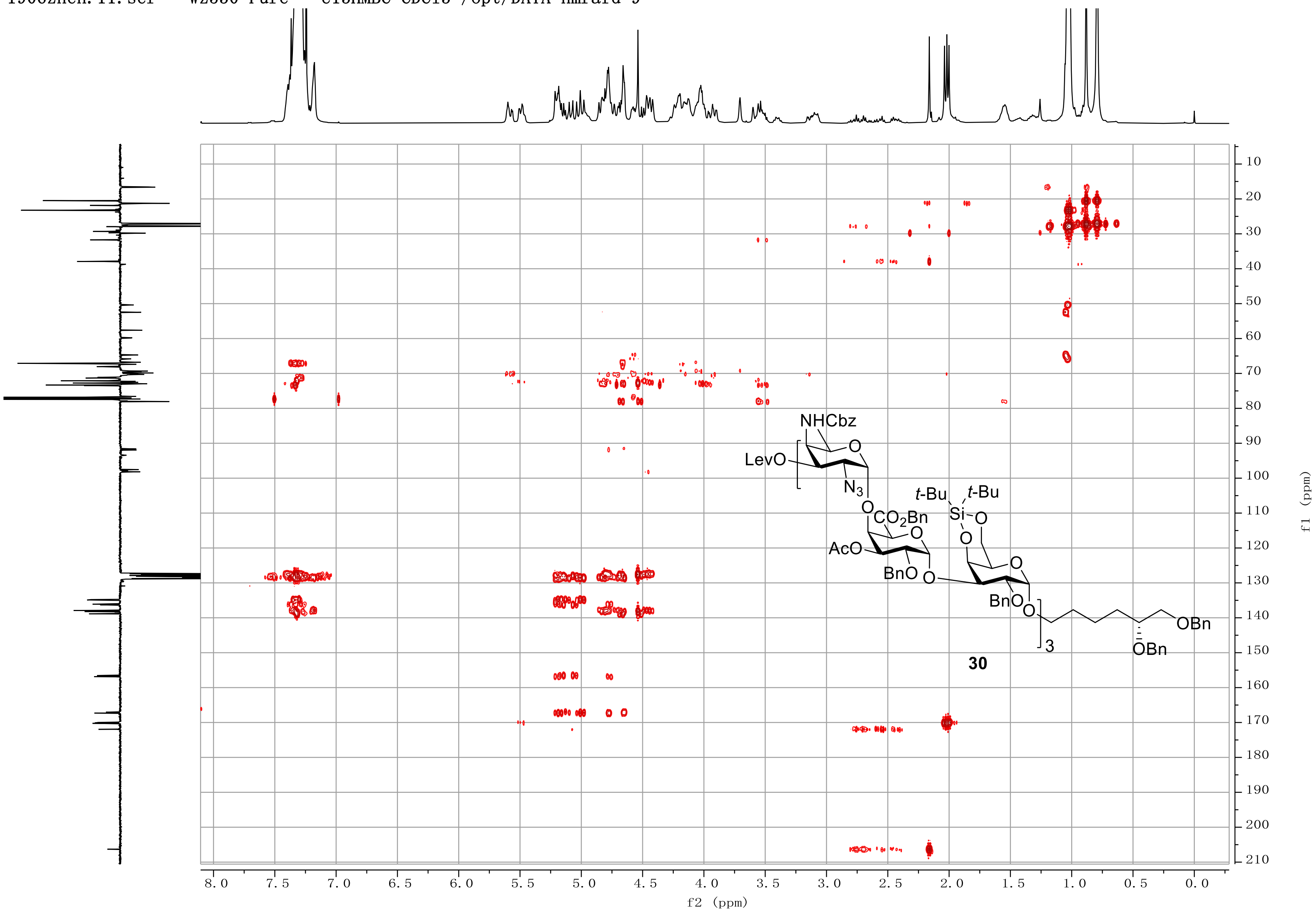

1907zhen.45.fid — wz533 — h1 CDCl3 /opt/DATA nmrafd 14

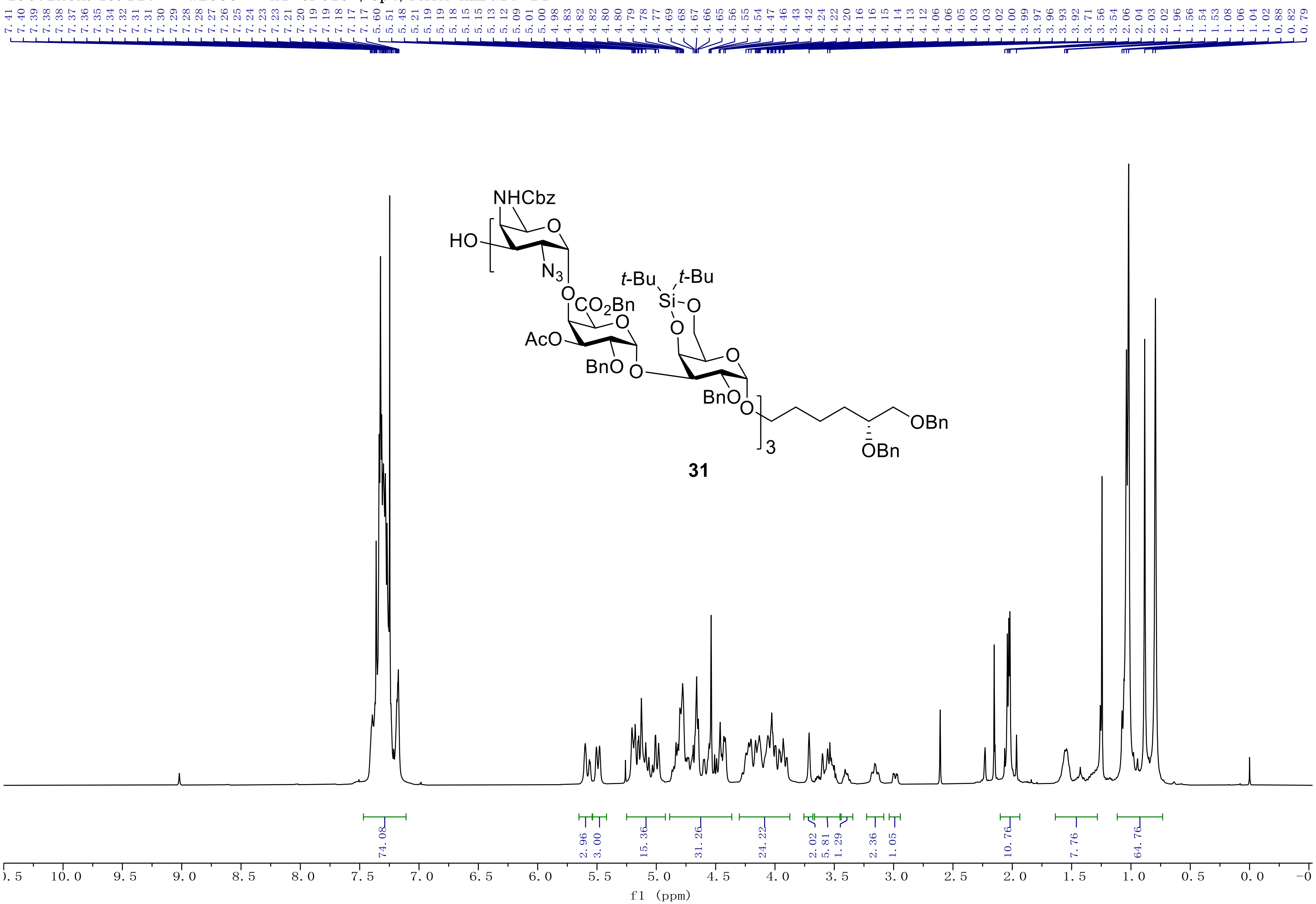

1907zhen.46.fid — wz533 — C13APT CDCl3 /opt/DATA nmrafd 14

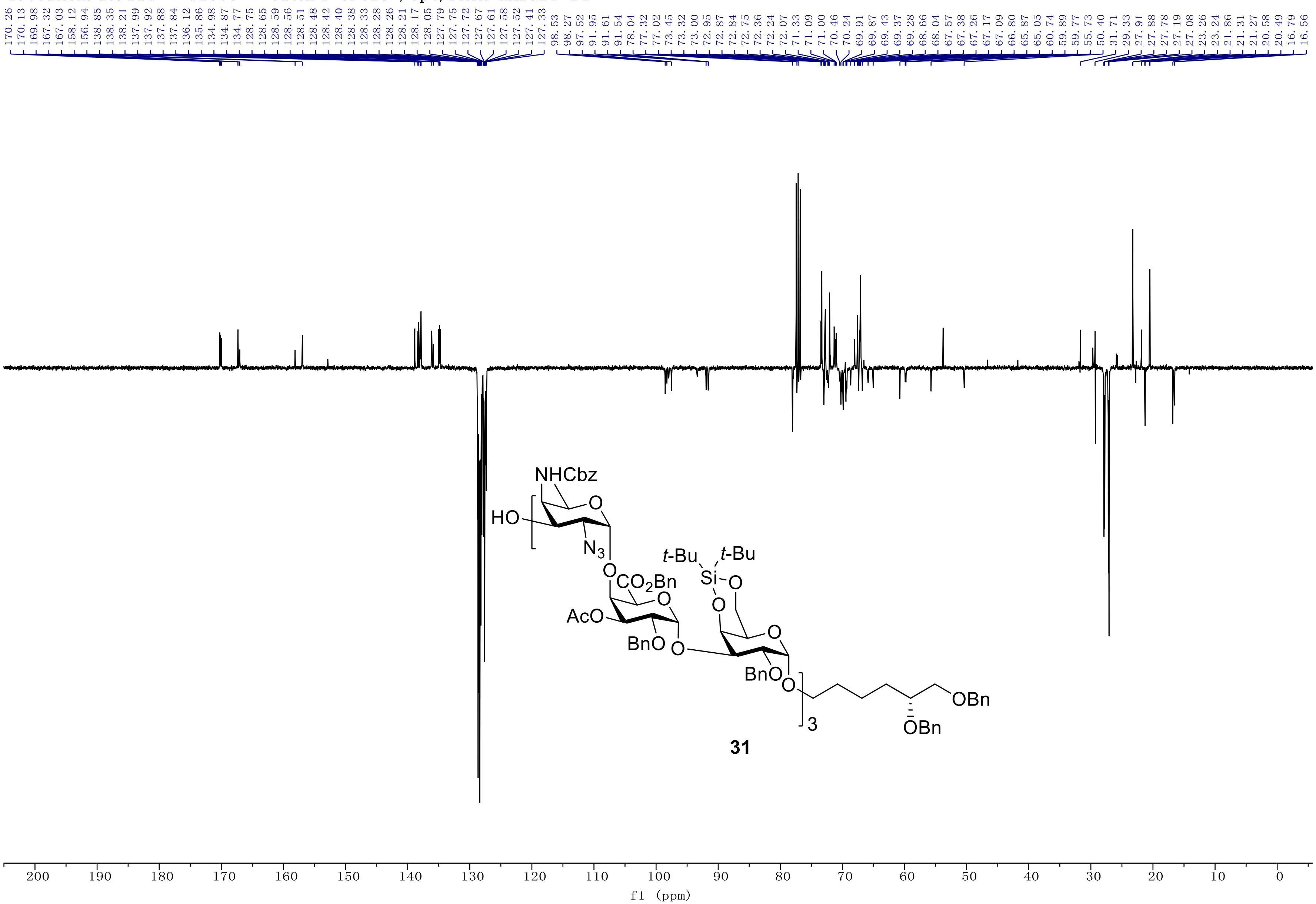

1907zhen.47.ser — wz533 — h1COSY CDCl3 /opt/DATA nmrafd 14

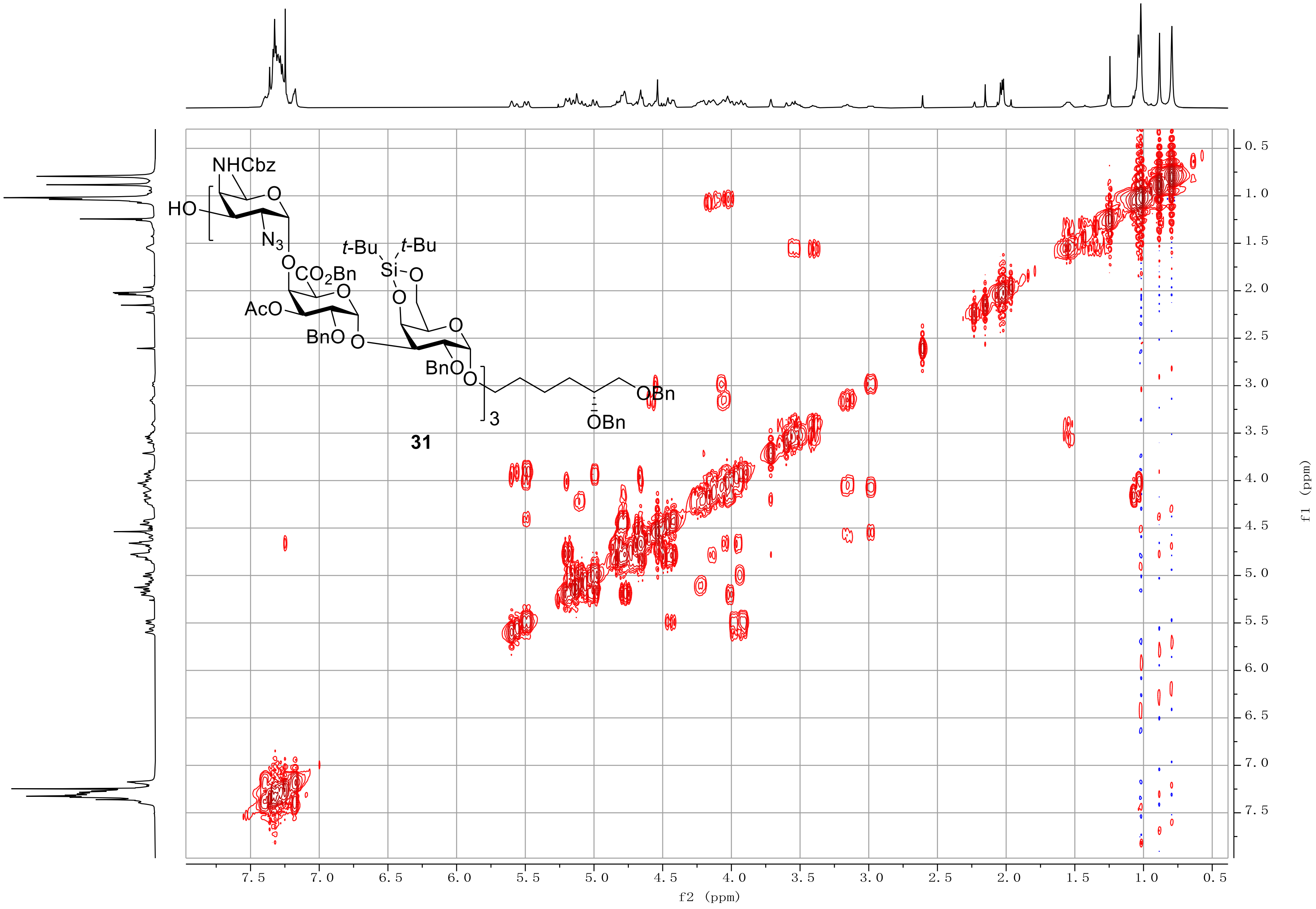

1907zhen.48.ser — wz533 — c13HSQC CDCl3 /opt/DATA nmrafd 14

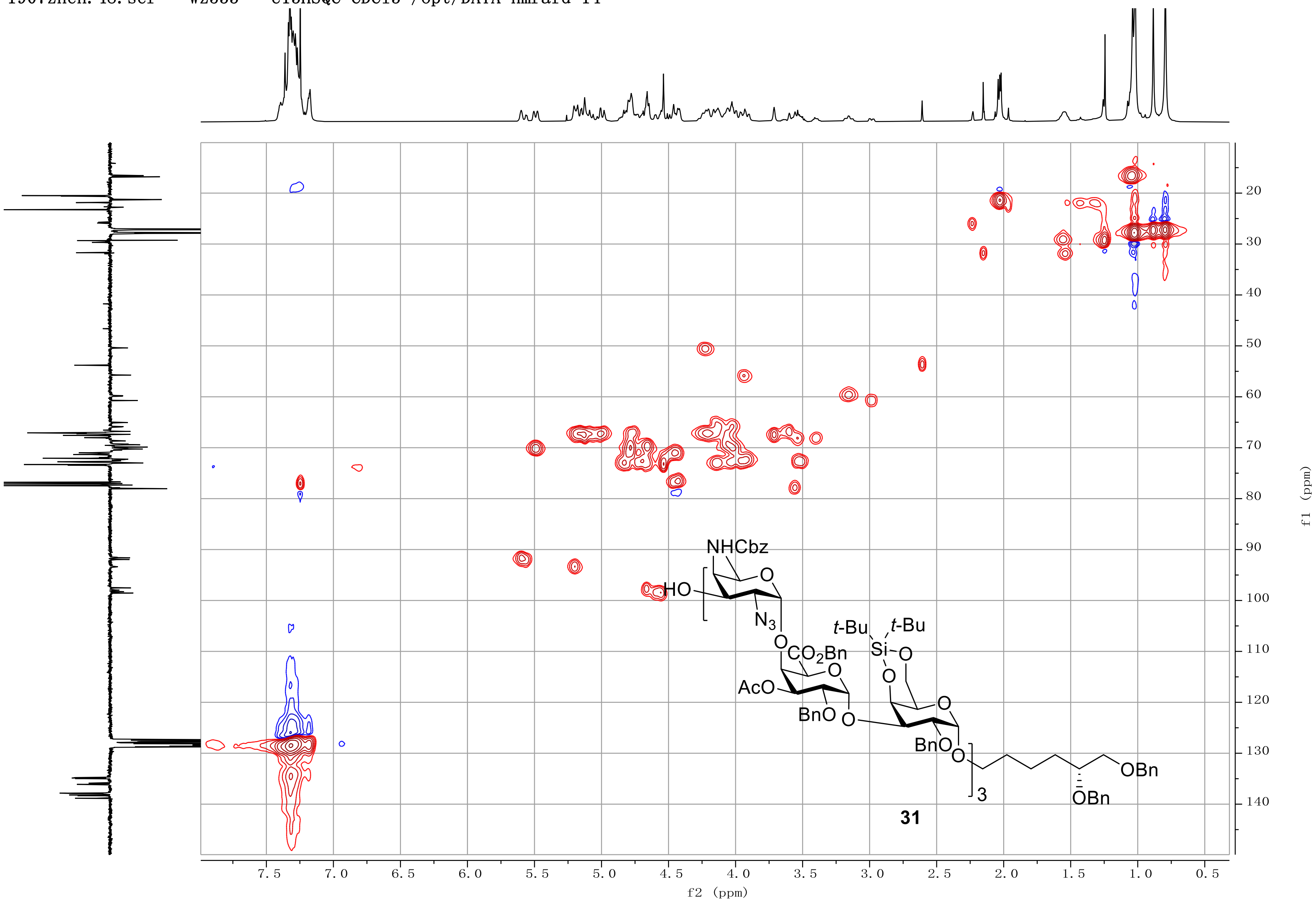

1907zhen.49.ser — wz533 — c13HMBC CDCl3 /opt/DATA nmrafd 14

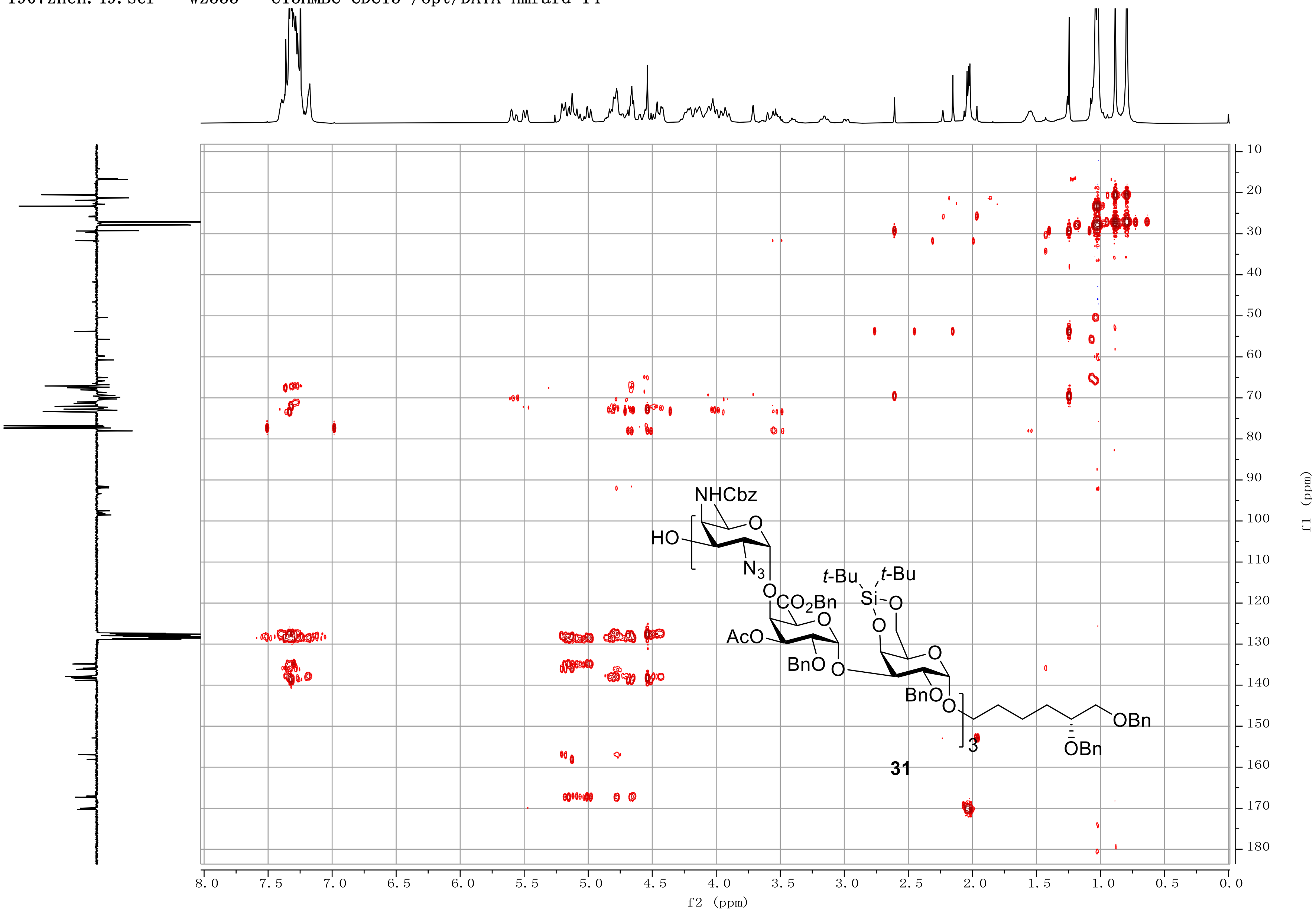

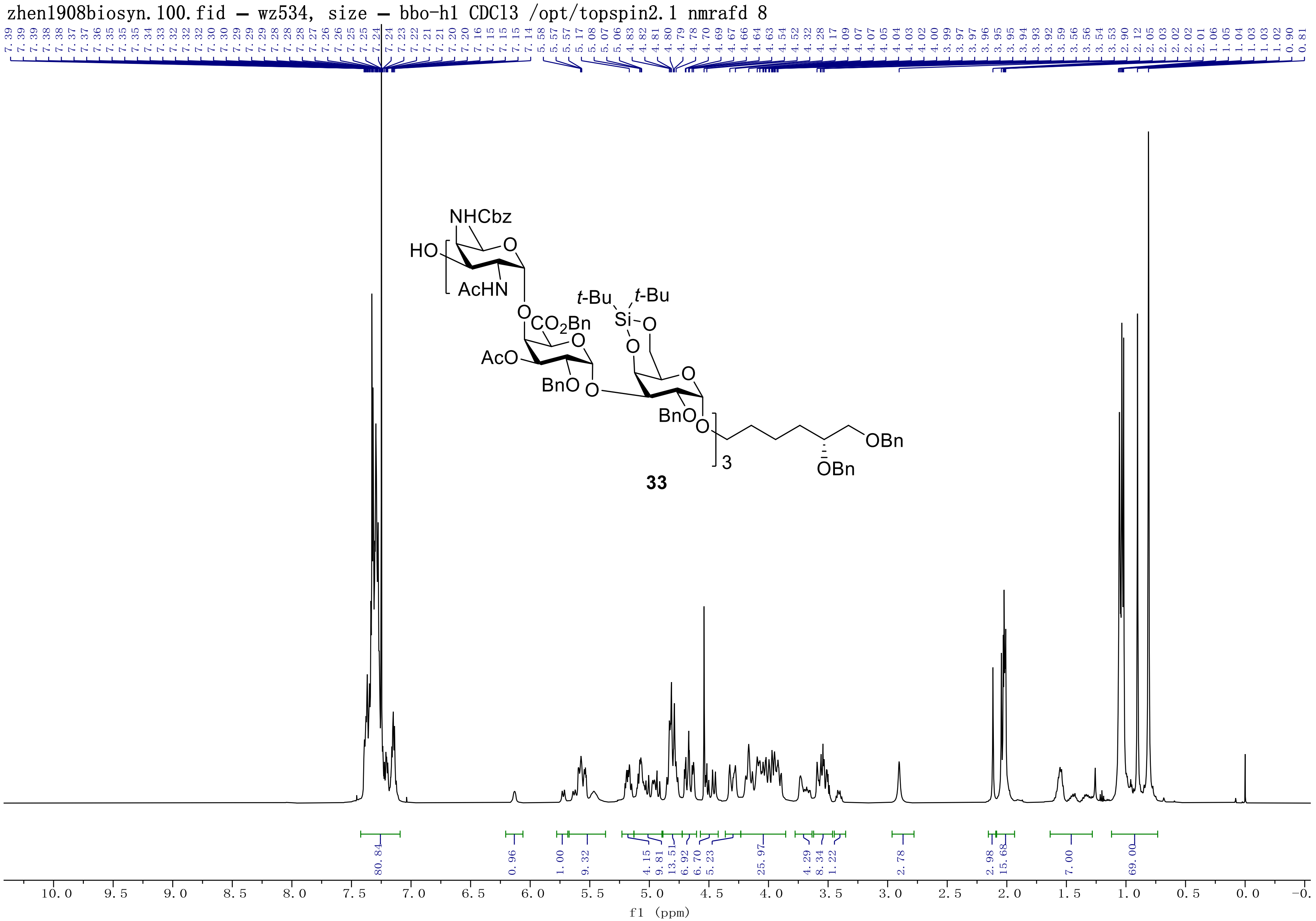
zhen1908biosyn.100.fid — wz534, size — bbo-h1 CDCl3 /opt/topspin2.1 nmrafd 8
NHCbz
HO
AcHN
O
CO2Bn
AcO
BnO
t-Bu
t-Bu
Si
BnO
3
OBn
OBn
33
f1 (ppm)

zhen1908biosyn.103.fid — wz534, size — bbo-c13-APT CDCl3 /opt/topspin2.1 nmrafd 8

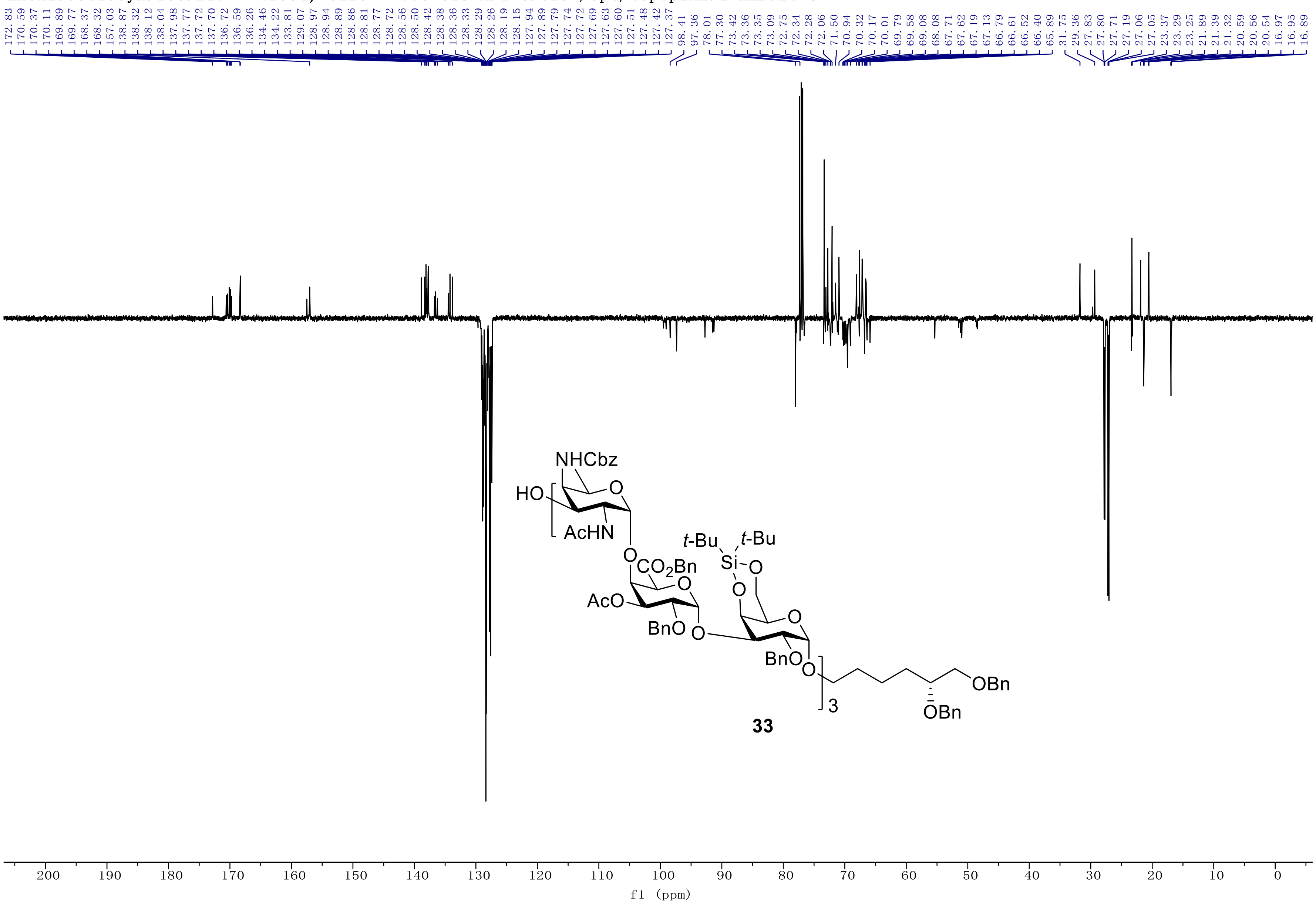

zhen1908biosyn.101.ser — wz534, size — bbo-h1-cosy CDCl3 /opt/topspin2.1 nmrafd 8

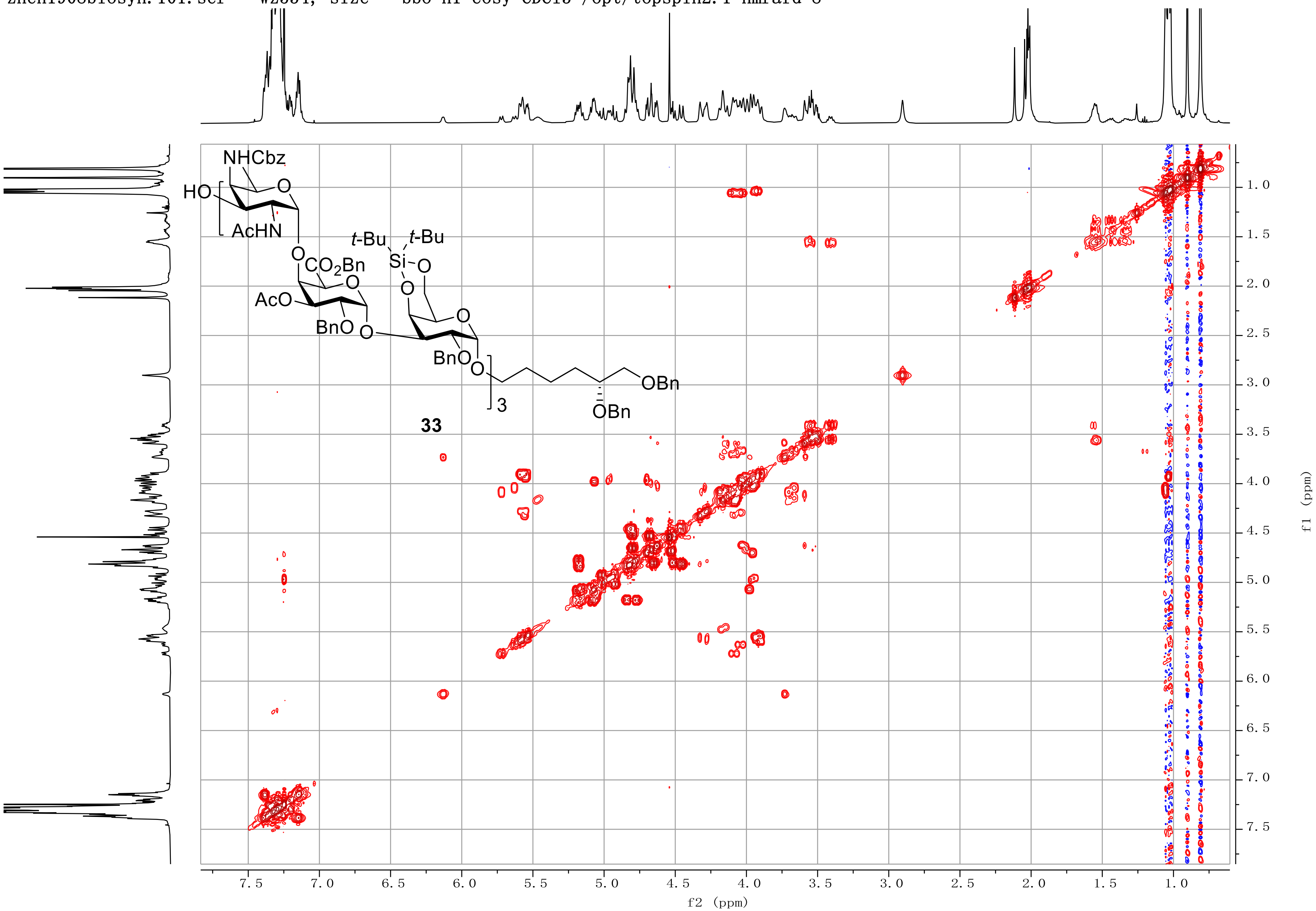

zhen1908biosyn.102.ser — wz534, size — bbo-c13-HSQC CDCl3 /opt/topspin2.1 nmrafd 8

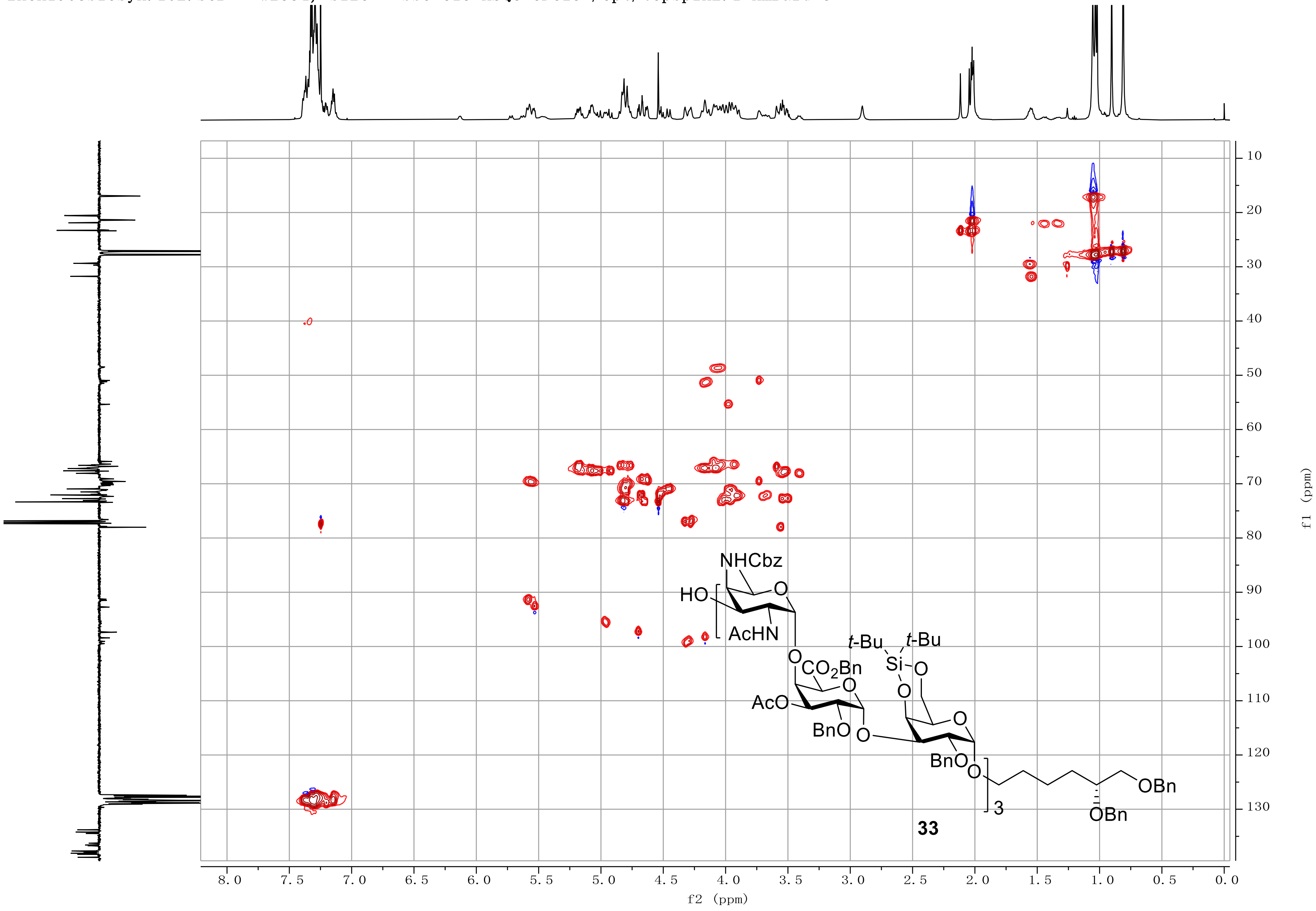

zhen1908biosyn.104.ser — wz534, size — bbo-c13-HMBC CDCl3 /opt/topspin2.1 nmrafd 8

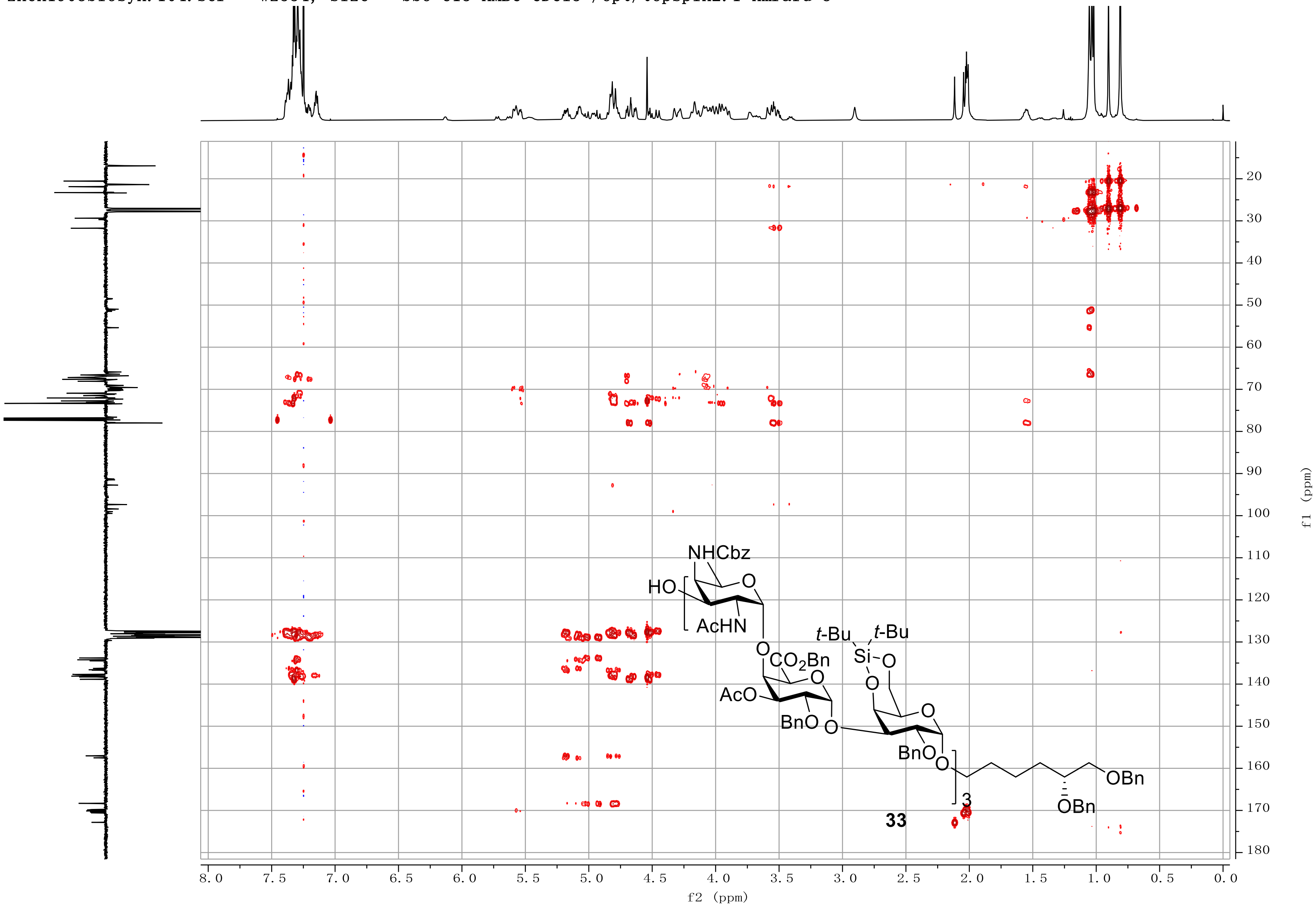

zhen1908biosyn.121.fid — wz535, size, 86mg — bbo-h1 CDCl3 /opt/topspin2.1 nmrafd 7

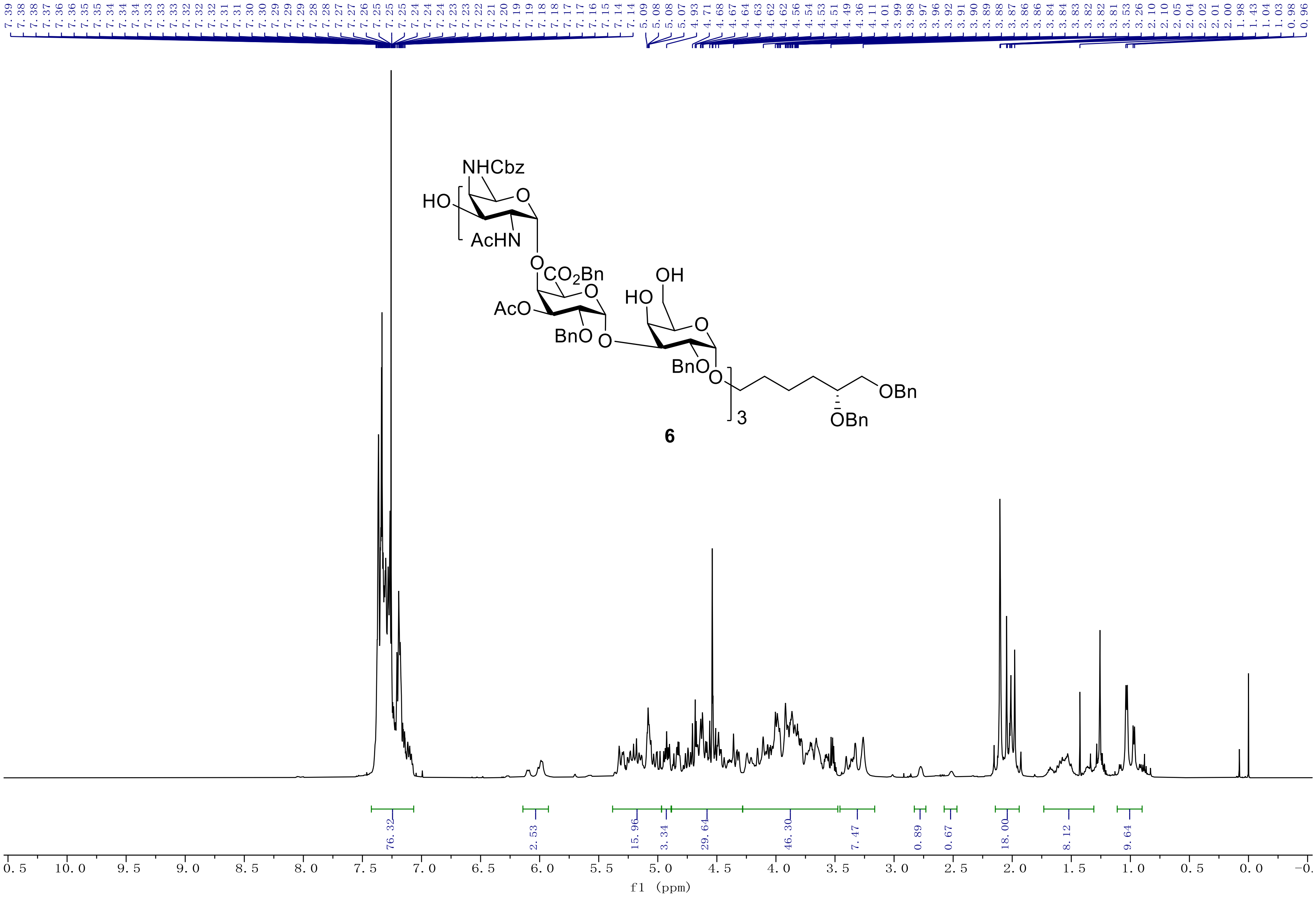

zhen1908biosyn.123.fid — wz535, size, 86mg — bbo-c13-APT CDCl3 /opt/topspin2.1 nmrafd 7

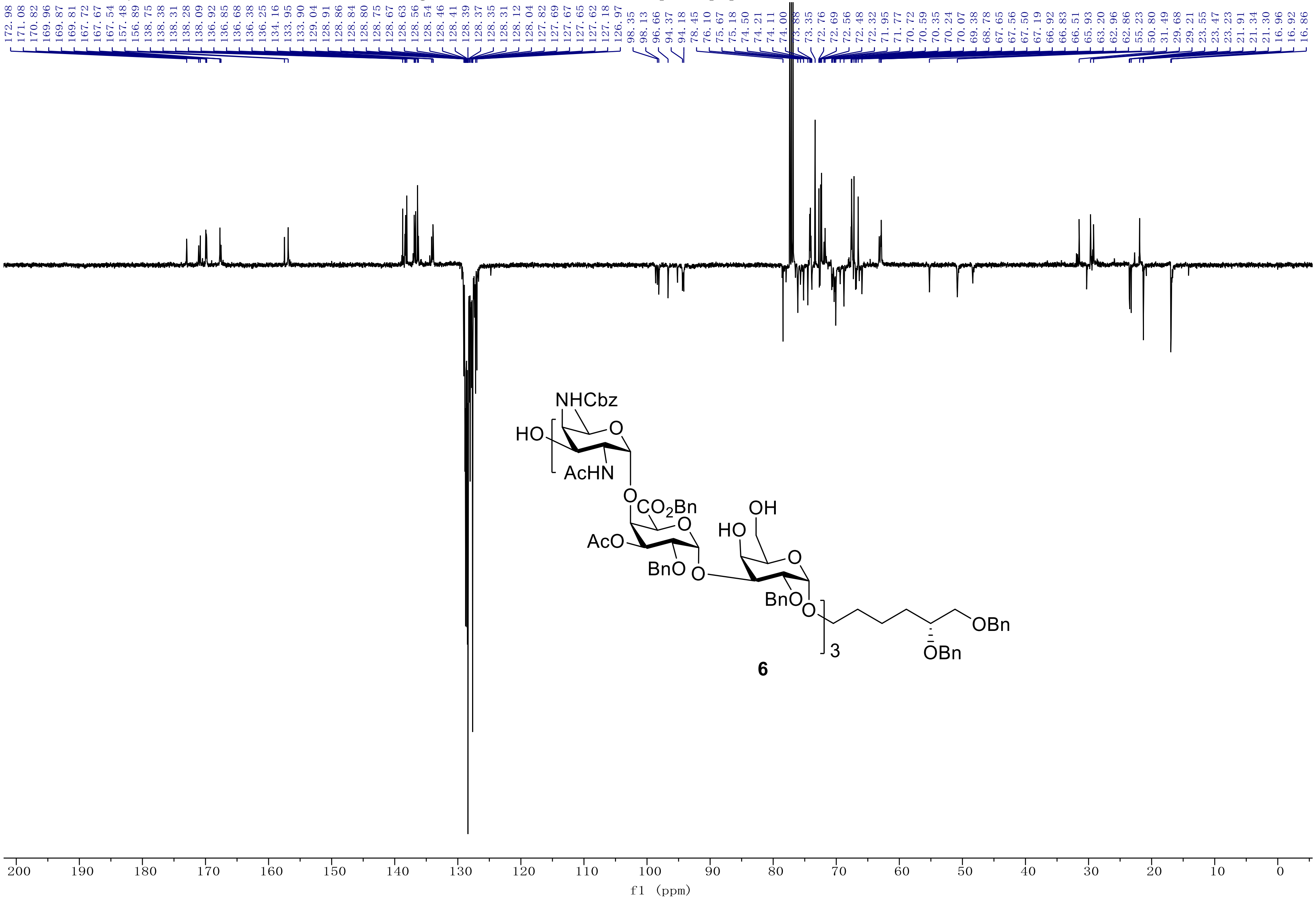

zhen1908biosyn.111.ser — wz535, 86mg — bbo-h1-cosy CDCl3 /opt/topspin2.1 nmrafd 10

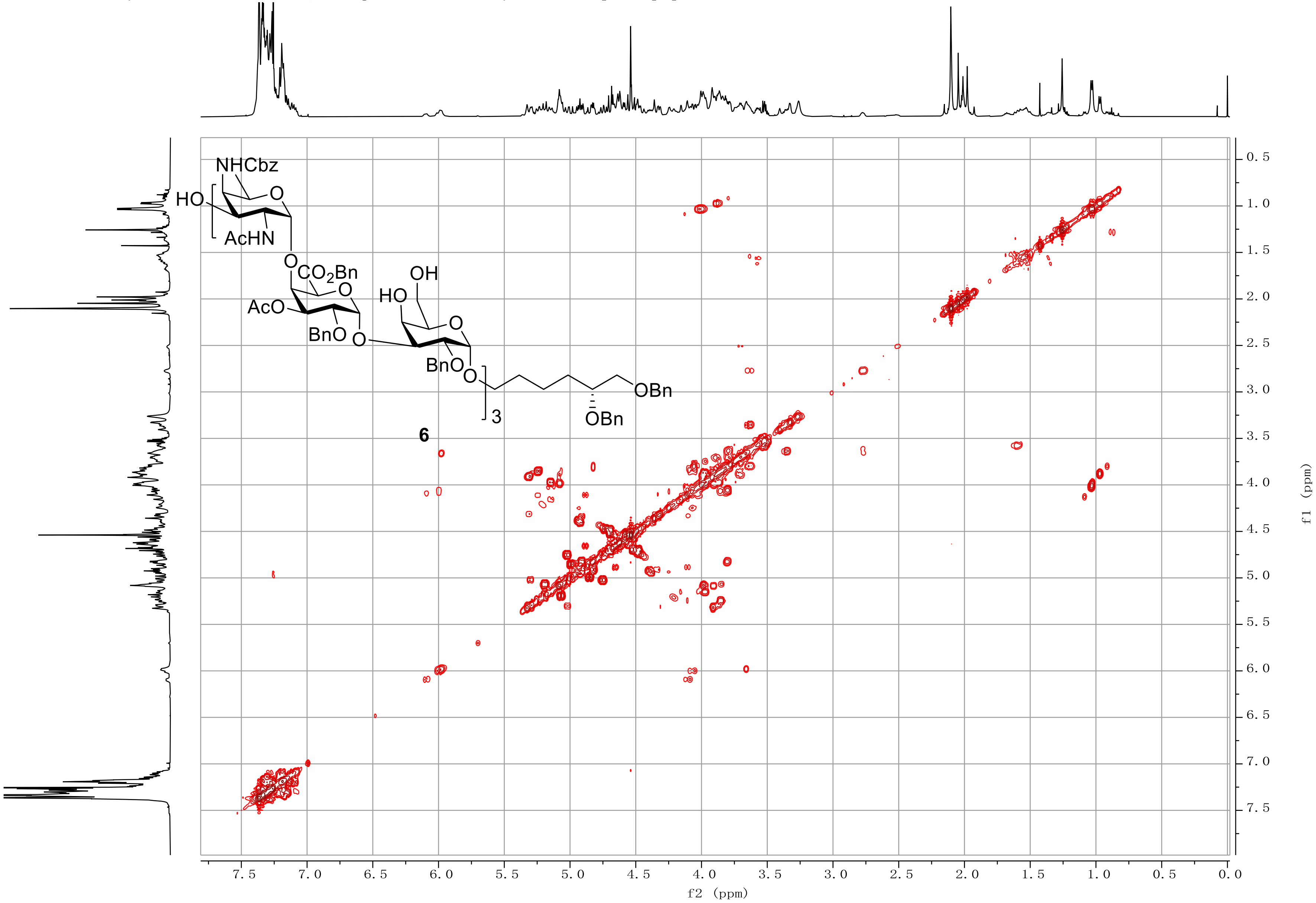

zhen1908biosyn.112.ser — wz535, 86mg — bbo-c13-HSQC CDCl3 /opt/topspin2.1 nmrafd 10

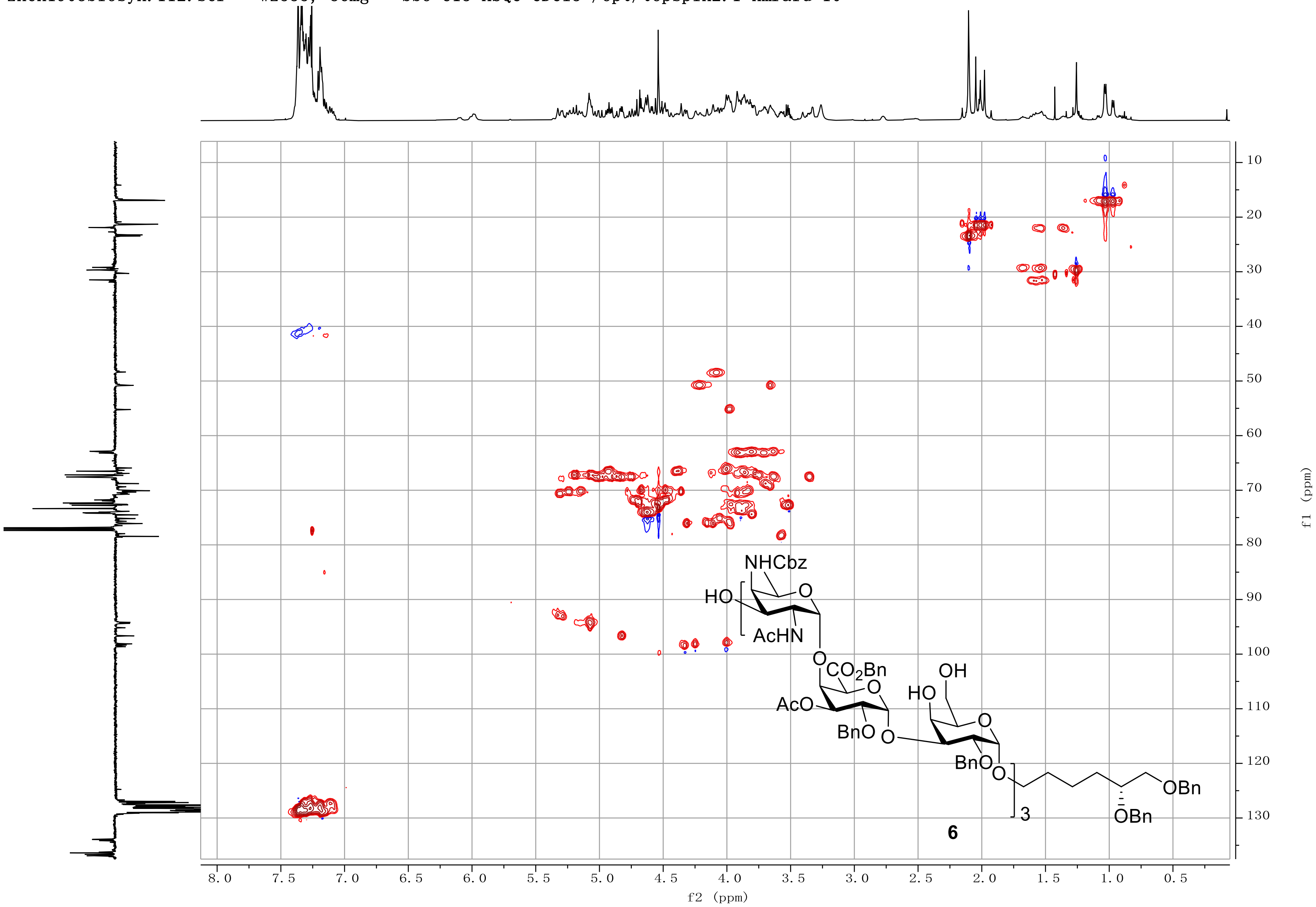

zhen1908biosyn.122.ser — wz535, size, 86mg — bbo-c13-HMBC CDCl3 /opt/topspin2.1 nmrafd 7

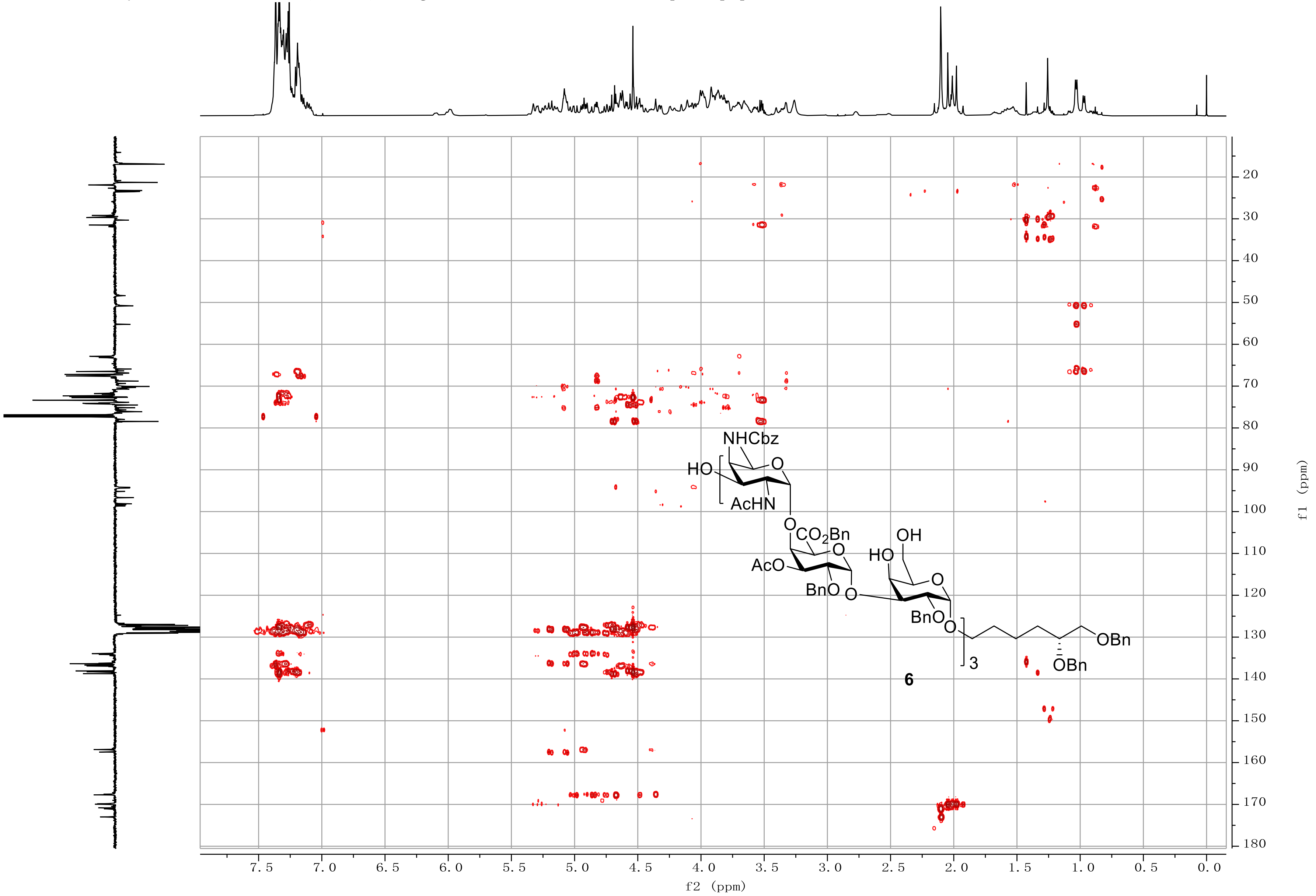

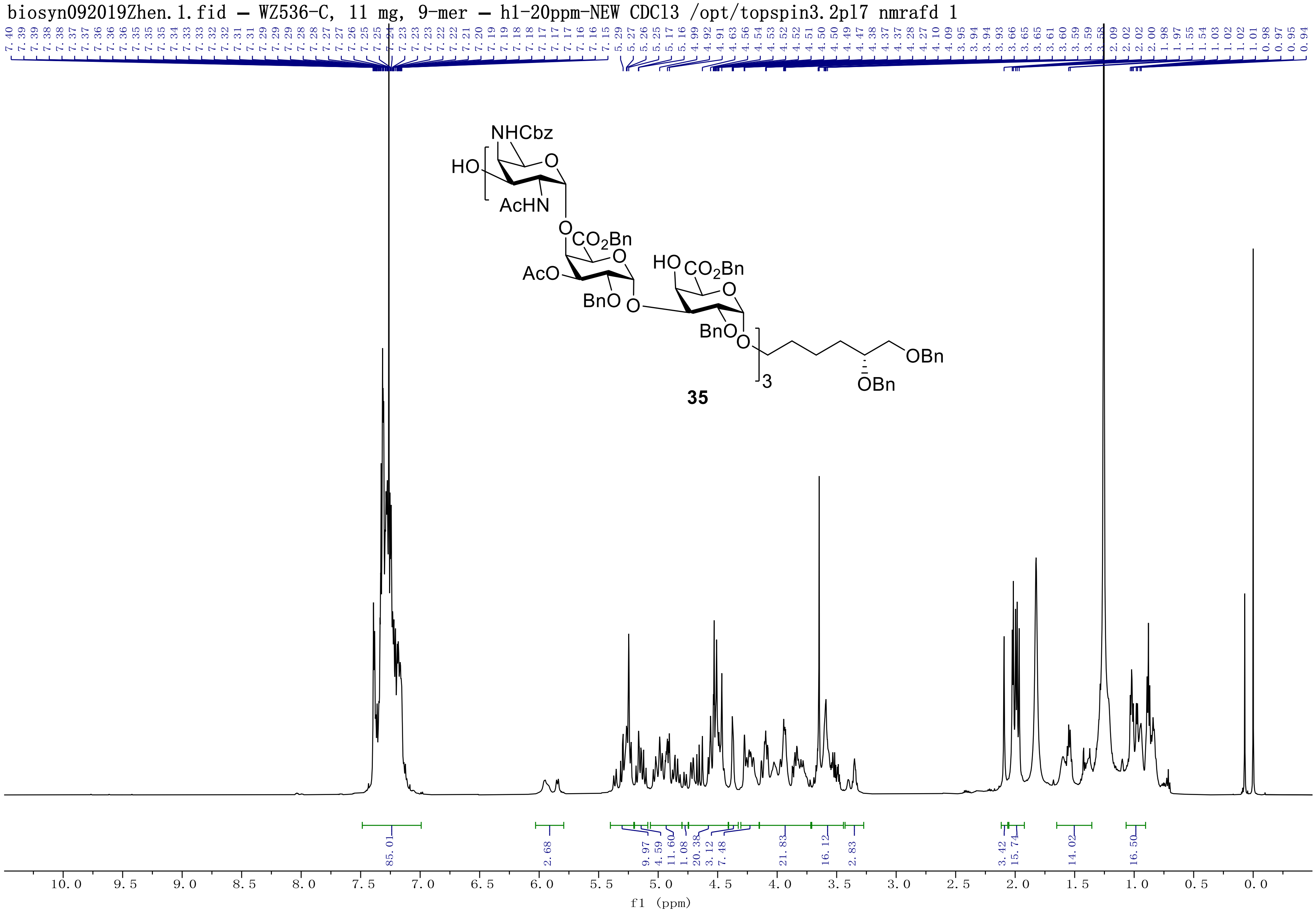
biosyn092019Zhen.1.fid — WZ536-C, 11 mg, 9-mer — h1-20ppm-NEW CDCl3 /opt/topspin3.2pl7 nmrafd 1
7.40 7.39 7.39 7.38 7.38 7.37 7.37 7.36 7.36 7.36 7.35 7.35 7.35 7.34 7.33 7.33 7.32 7.32 7.31 7.31 7.29 7.29 7.29 7.28 7.28 7.27 7.27 7.26 7.25 7.25 7.24 7.23 7.23 7.23 7.22 7.22 7.21 7.20 7.19 7.19 7.18 7.18 7.17 7.17 7.17 7.17 7.16 7.16 7.15 5.29 5.27 5.26 5.25 5.17 5.16 4.99 4.92 4.91 4.63 4.56 4.54 4.53 4.52 4.52 4.51 4.50 4.50 4.49 4.47 4.38 4.37 4.37 4.28 4.27 4.10 4.09 3.95 3.94 3.94 3.93 3.66 3.65 3.61 3.60 3.59 3.59 3.58 2.09 2.02 2.02 2.00 1.98 1.97 1.55 1.54 1.03 1.02 1.01 0.98 0.97 0.95 0.94
NHCbz
HO
O
AcHN
O
CO2Bn
O
AcO
BnO
O
HO
CO2Bn
O
BnO
O
3
OBn
OBn
35
85.01
2.68
9.97
4.59
11.60
1.08
20.38
3.12
7.48
21.83
16.12
2.83
3.42
15.74
14.02
16.50
10.0 9.5 9.0 8.5 8.0 7.5 7.0 6.5 6.0 5.5 5.0 4.5 4.0 3.5 3.0 2.5 2.0 1.5 1.0 0.5 0.0
f1 (ppm)

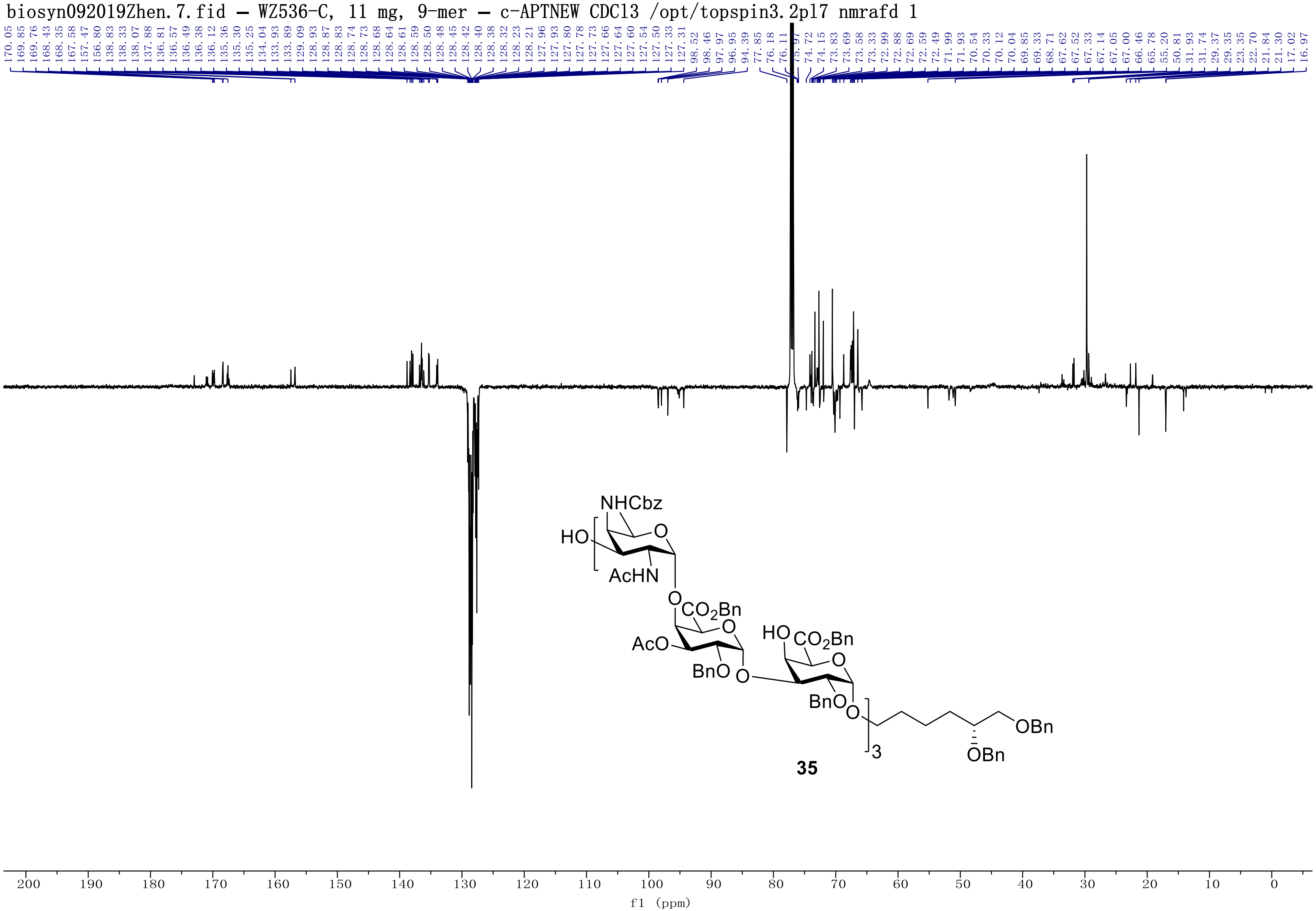
biosyn092019Zhen.7.fid — WZ536-C, 11 mg, 9-mer — c-APTNEW CDCl3 /opt/topspin3.2pl7 nmrafd 1
170.05 169.85 169.76 168.43 168.35 167.58 157.47 156.80 138.83 138.33 138.07 137.88 136.81 136.57 136.49 136.38 136.12 135.36 135.30 135.25 134.04 133.93 133.89 129.09 128.93 128.87 128.83 128.74 128.73 128.68 128.64 128.61 128.59 128.50 128.48 128.45 128.42 128.40 128.38 128.32 128.23 128.21 127.96 127.93 127.80 127.78 127.73 127.66 127.64 127.60 127.54 127.50 127.33 127.31 98.52 98.46 97.97 96.95 94.39 77.85 76.18 76.11 75.97 74.72 74.15 73.83 73.69 73.58 73.33 72.99 72.88 72.69 72.59 72.49 71.99 71.93 70.54 70.33 70.12 70.04 69.85 69.33 68.71 67.62 67.52 67.33 67.14 67.05 67.00 66.46 65.78 55.20 50.81 31.93 31.74 29.37 29.35 23.35 22.70 21.84 21.30 17.02 16.97
NHCbz
HO
O
AcHN
O
CO2Bn
AcO
O
BnO
HO
CO2Bn
O
BnO
O
3
OBn
OBn
35
200 190 180 170 160 150 140 130 120 110 100 90 80 70 60 50 40 30 20 10 0
f1 (ppm)

biosyn092019Zhen.2.ser — WZ536-C, 11 mg, 9-mer — h1-cosygpNEW CDCl3 /opt/topspin3.2pl7 nmrafd 1

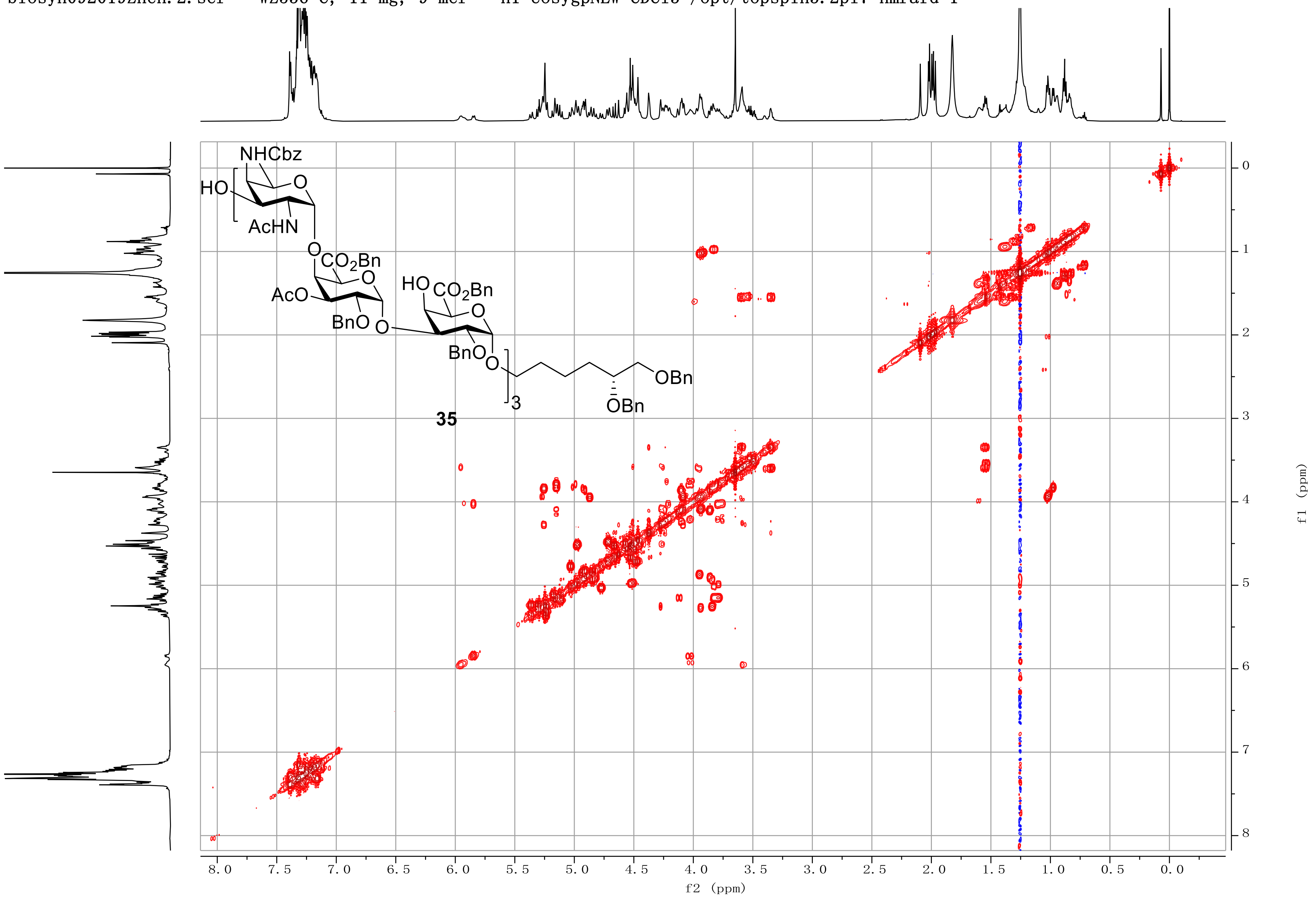

biosyn092019Zhen.4.ser — WZ536-C, 11 mg, 9-mer — c-HSQC CDCl3 /opt/topspin3.2pl7 nmrafd 1

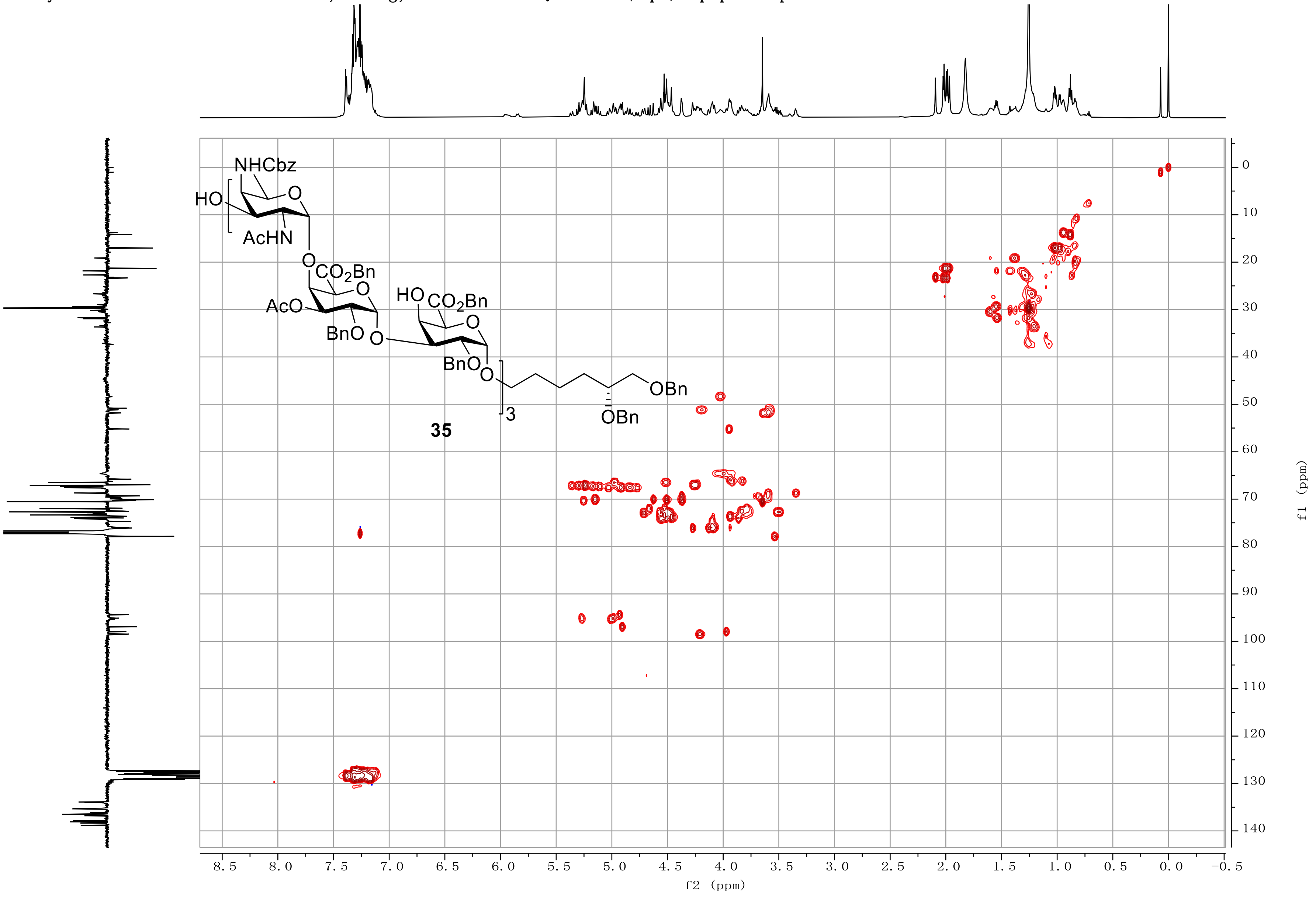

biosyn092019Zhen.5.ser — WZ536-C, 11 mg, 9-mer — c-hmbcNEW CDCl3 /opt/topspin3.2pl7 nmrafd 1

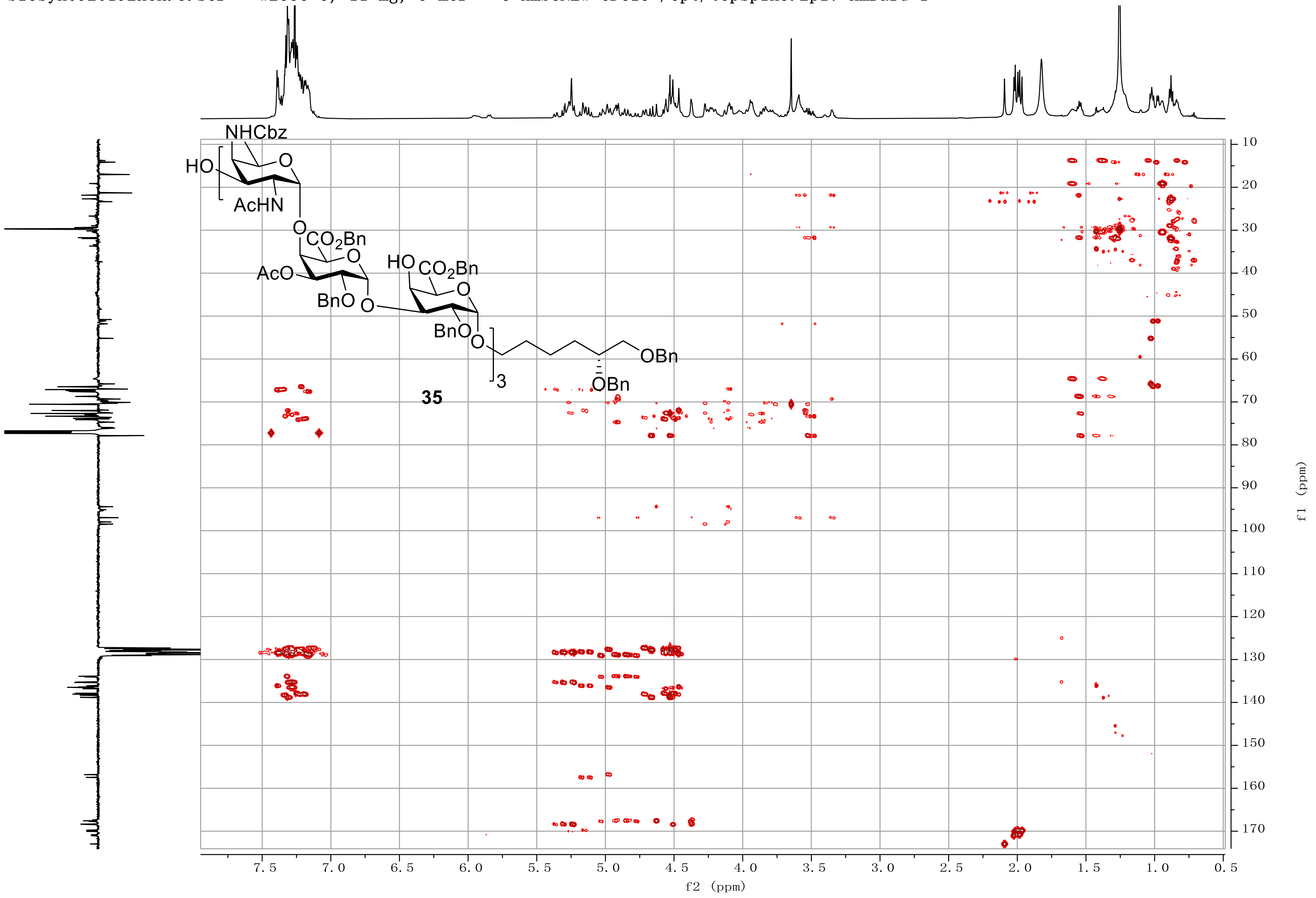

biosyn092019Zhen.6.ser — WZ536-C, 11 mg, 9-mer — c-hmbcDIRECTgatedNEW CDCl3 /opt/topspin3.2pl7 nmrafd 1

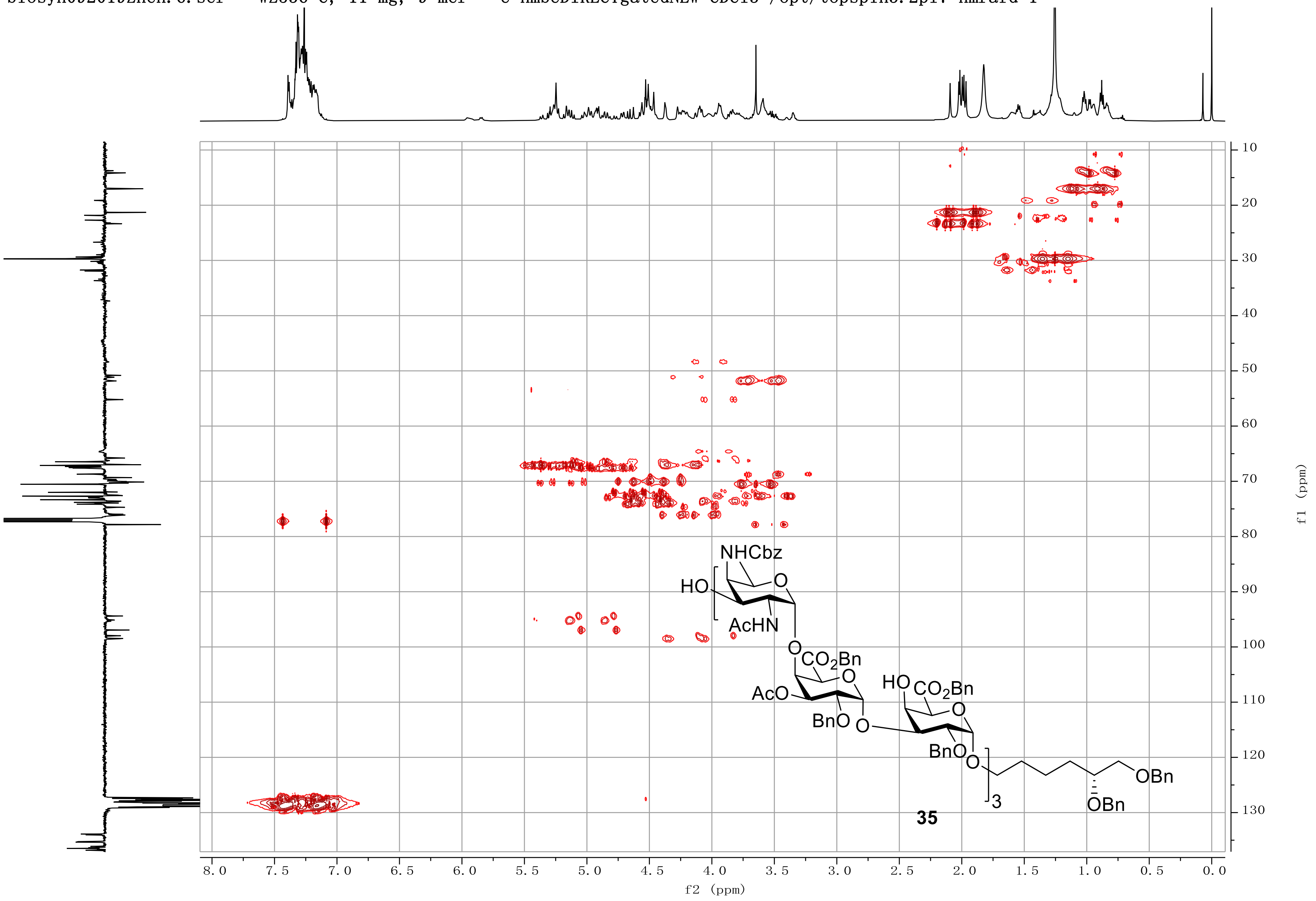

biosyn112019zhen.1.fid — wz537-b — h1-20ppm-NEW D2O /opt/topspin3.2pl7 nmrafd 60

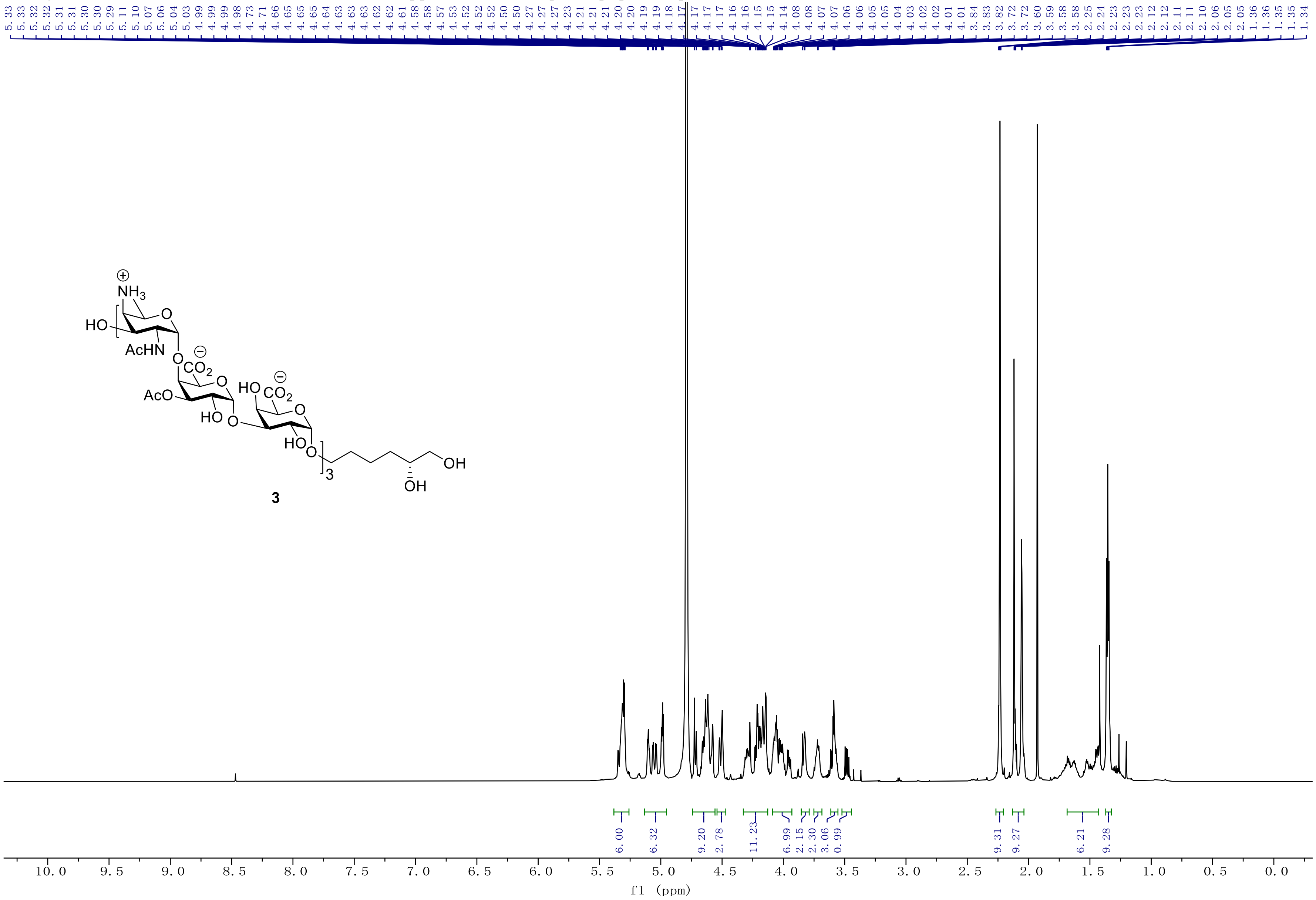

biosyn112019zhen.8.fid — wz537-b — c-APT-pl12-pl13-d2o D2O /opt/topspin3.2pl7 nmrafd 60

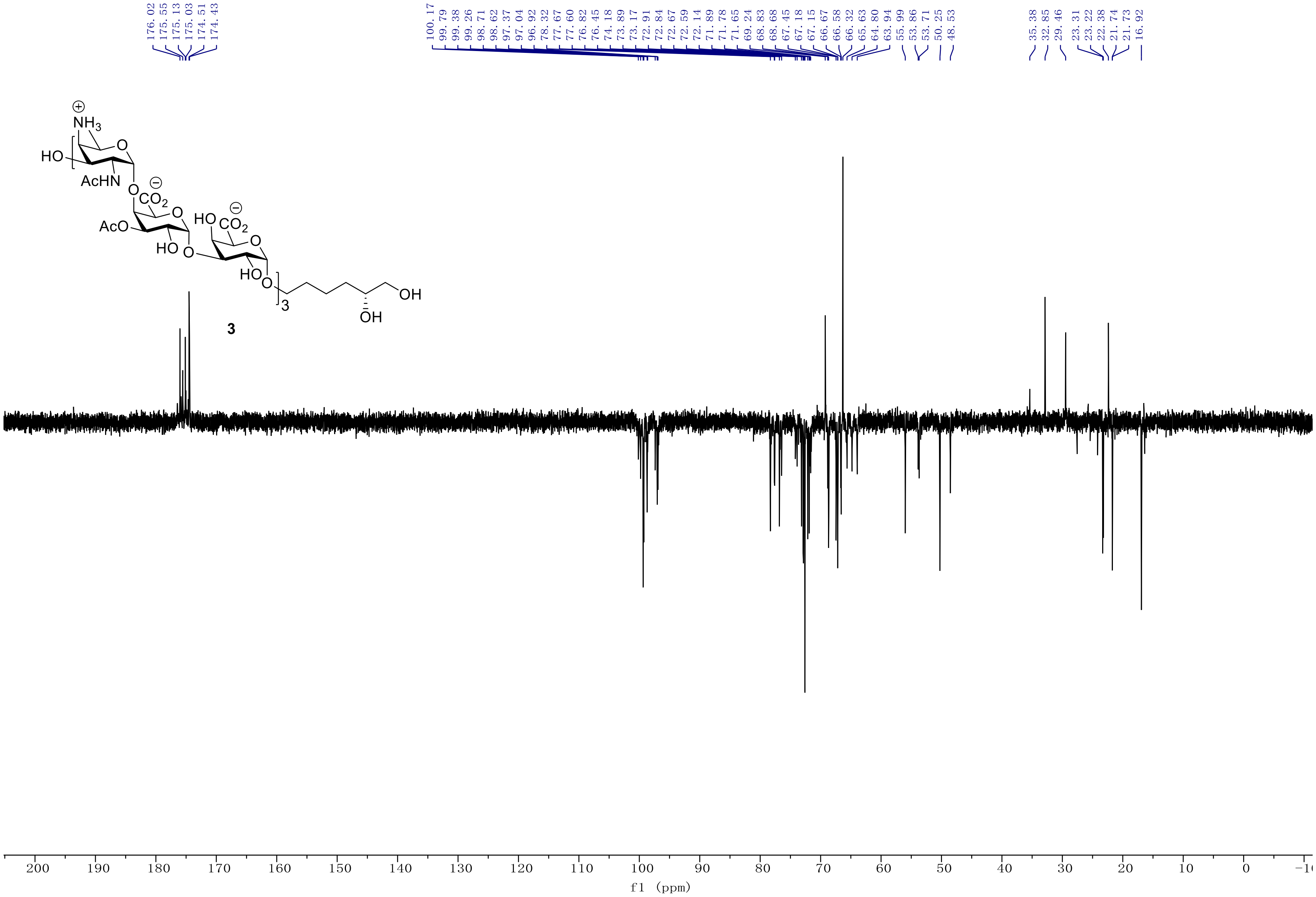

biosyn112019zhen.3.ser — wz537-b — h1-cosygpprNEW D2O /opt/topspin3.2pl7 nmrafd 60

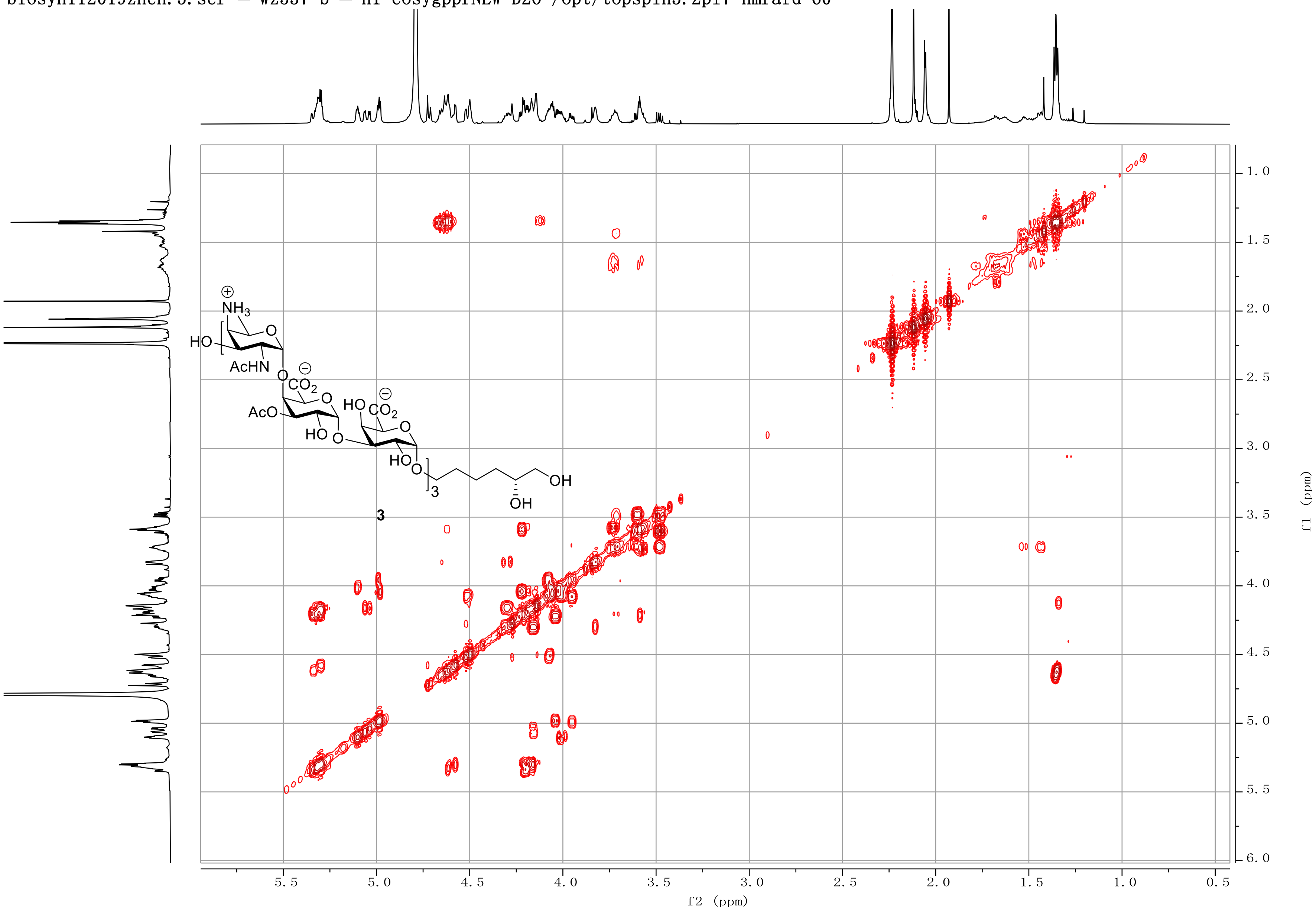

biosyn112019zhen.4.ser — wz537-b — c-HSQC-ct-D20 D20 /opt/topspin3.2pl7 nmrafd 60

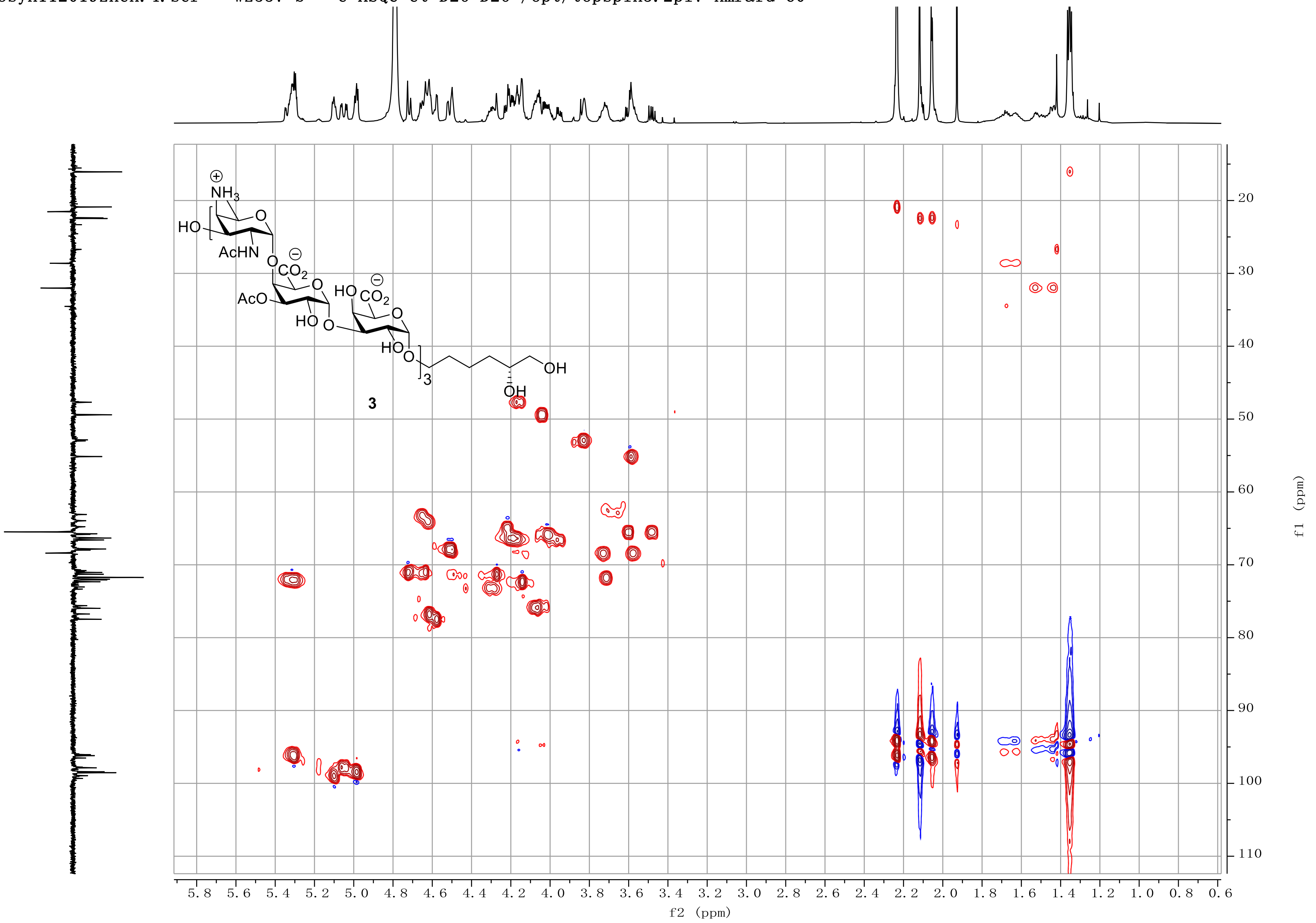

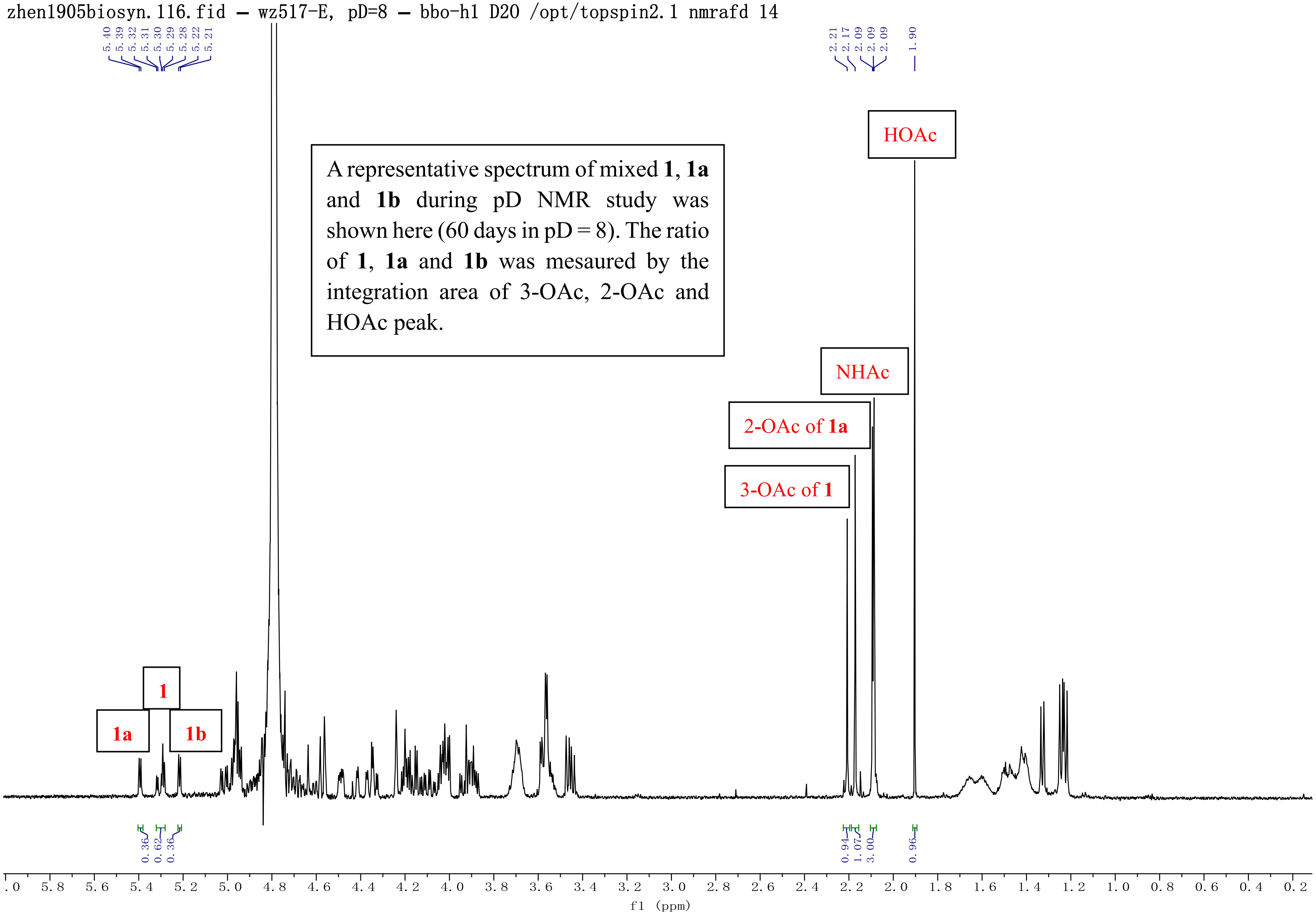
zhen1905biosyn.116.fid — wz517-E, pD=8 — bbo-h1 D20 /opt/topspin2.1 nmrafd 14
A representative spectrum of mixed **1**, **1a** and **1b** during pD NMR study was shown here (60 days in pD = 8). The ratio of **1**, **1a** and **1b** was mesaured by the integration area of 3-OAc, 2-OAc and HOAc peak.
HOAc
NHAc
2-OAc of **1a**
3-OAc of **1**
1
1a
1b
5.40 5.39 5.32 5.31 5.30 5.29 5.28 5.22 5.21
2.21 2.17 2.09 2.09 2.09
1.90
0.36 0.62 0.36
0.94 1.07 3.00
0.96
f1 (ppm)
